# Kinetic Resolution of Racemic Amines via Palladium/Chiral Phosphoric Acid-Catalyzed Intramolecular Allylation: Stereodivergent Access to Chiral 1,3-Disubstituted Isoindolines

**DOI:** 10.1021/acs.joc.5c00568

**Published:** 2025-06-12

**Authors:** Bing-Syuan Wu, Yu-Ming Lin, Cheng-Che Tsai

**Affiliations:** Department of Chemistry, 34890 Tunghai University, Taichung 40704, Taiwan

## Abstract

This article presents the kinetic resolution (KR) of racemic amines through stereoselective intramolecular allylation with palladium as a catalyst and chiral phosphoric acid as a cocatalyst. The KR reactions have high selectivity factors, producing chiral 1,3-disubstituted isoindolines (DSIs) and recovering enantioenriched amines. Subsequent palladium-catalyzed allylation of the enantioenriched amines, with an achiral Brønsted acid (BA) cocatalyst, yields *cis*-DSIs with favorable stereocontrol. Notably, this study is the first to investigate the effect of using a BA catalyst on the diastereodivergence of allylation reactions, suggesting a stereodivergent strategy for accessing all possible stereoisomers of chiral DSIs, relying on a single chiral source.

## Graphical abstract

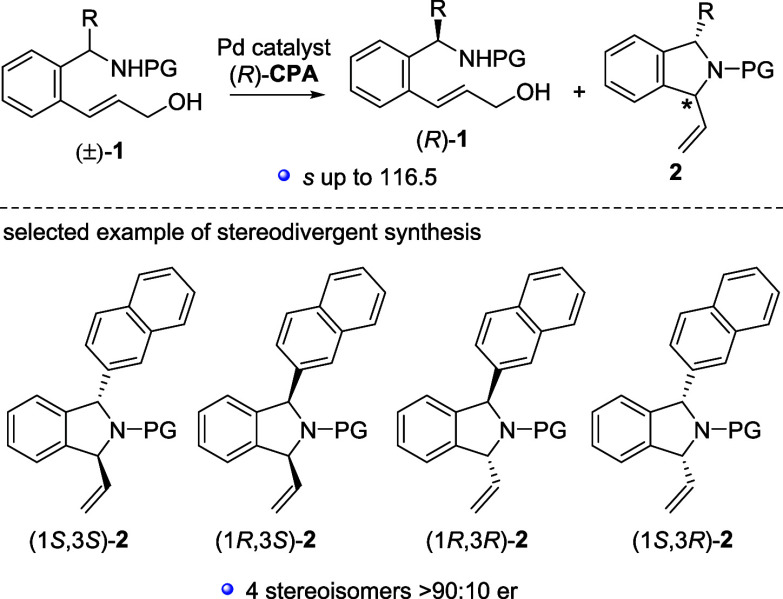

## Introduction

Isoindolines (i.e., 2,3-dihydro-1*H*-isoindoles) are key structural components of numerous biologically active compounds (Scheme 1a);^1^ for example, isoindoline 1-carboxylic acid derivatives, which are [*c*]-fused bicyclic proline analogs, serve as valuable building blocks in the synthesis of bioactive compounds, including agonists of peroxisome proliferator-activated receptor δ (**I**),^2^ inhibitors of heat shock protein 90 (**II**),^3^ and inverse agonists of retinoic acid receptor-related orphan receptor C2 (AZD0284, **III**).^4^ Notably, 1,3-disubstituted isoindoline (DSI) motifs act as core structures that considerably influence the pharmaceutical properties of some bioactive molecules. For instance, DSI frameworks were integrated into the molecular structure of the natural product gephyrotoxin, yielding a new series of selective muscarinic acetylcholine receptor modulators (**IV**).^5^ In addition, abeorphine (**V**), a structurally rigid five-membered analog of apomorphine, was identified as an orally active dopamine agonist with prolonged activity.^6^ Moreover, compound **VI** was identified as functional endothelin-A-selective receptor antagonists, and both the *cis*- and *trans*-stereoisomers were found to be active.^7^

**Scheme 1:**
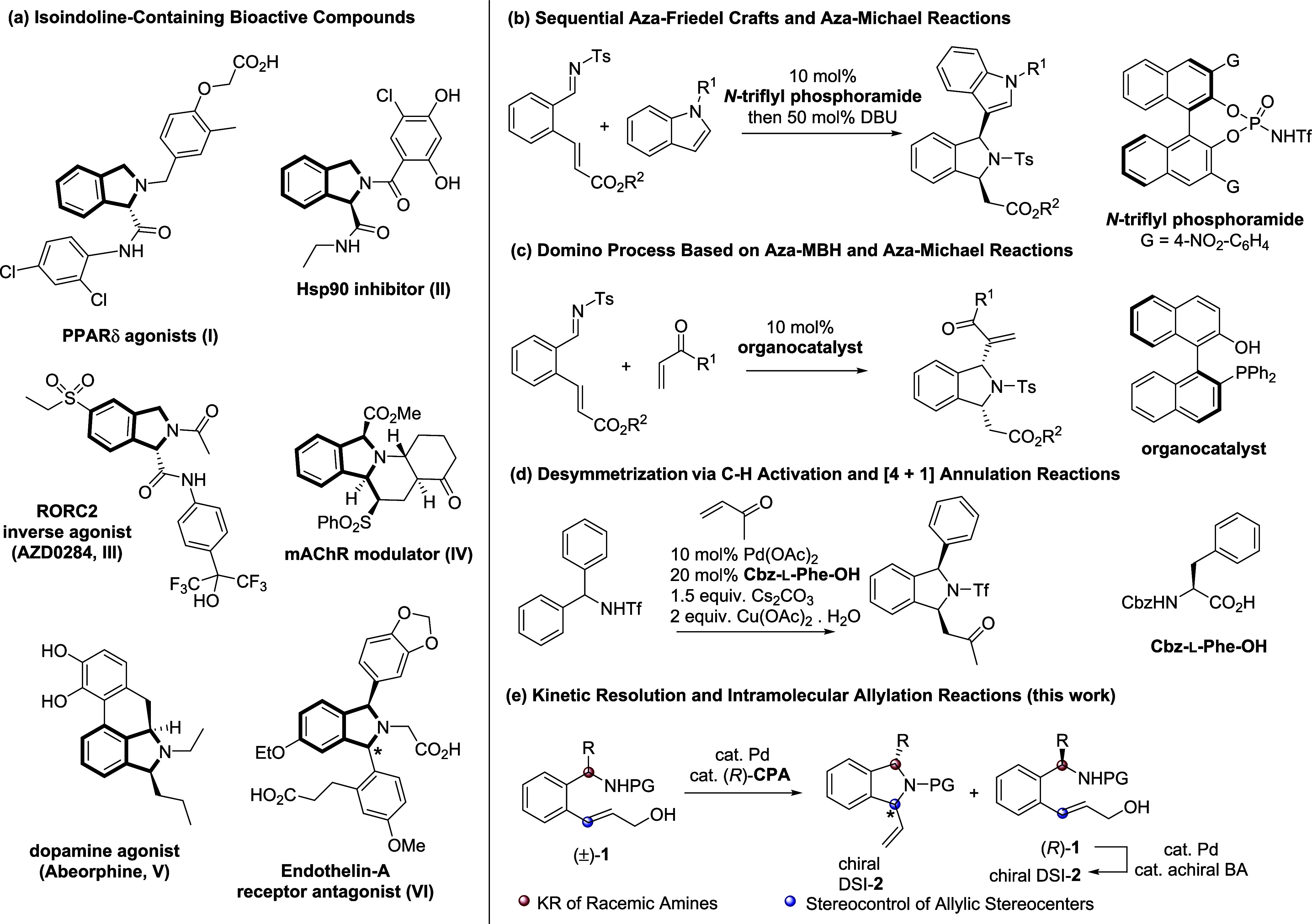
Chiral 1,3-Disubsituted Isoindolines (DSIs) and Their Asymmetric Catalytic Syntheses

The production of DSIs, especially the asymmetric synthesis of chiral DSIs, has become a prominent research focus in synthetic and medical chemistry.^8–10^ Enders et al. developed a one-pot method for chiral DSI synthesis, with their method involving a chiral *N*-triflyl phosphoramide-catalyzed aza-Friedel–Crafts reaction between indoles and imines, followed by a DBU-catalyzed intramolecular aza-Michael reaction (Scheme 1b).^11^ The Sasai group demonstrated the synthesis of chiral DSIs through a domino reaction by using a chiral organocatalyst incorporating Brønsted acid (BA) and Lewis base groups (Scheme 1c). This organocatalyst promotes a cascade aza-Morita–Baylis–Hillman reaction, followed by an intramolecular aza-Michael reaction.^12^ Dethe et al. engineered the palladium (Pd)-catalyzed desymmetrization of diarylmethyltriflamides through enantioselective C–H activation and [4 + 1] annulation reactions, offering a distinct approach for producing chiral DSIs (Scheme 1d).^13^

In this study, chiral DSIs **2** can be synthesized through a cyclization process that involves Pd/chiral phosphoric acid (CPA)-catalyzed kinetic resolution (KR) of racemic amines **1**, followed by an intramolecular allylic amination reaction (Scheme 1e).^14–16^ Given that the stereochemistry of isoindoline skeletons influences their bioactivity,^3,4,17^ the development of an asymmetric methodology that allows selective access to chiral *cis*- and *trans*-DSIs would be highly desirable.^7,18^ We investigate stereocontrol during the formation of the allylic stereocenter (i.e., *cis*–*trans* selectivity) through allylation of enantioenriched amine **1**, which is recovered from the KR reaction, by using an achiral BA as the cocatalyst. Notably, this study is the first to examine how a BA cocatalyst affects the diastereodivergence of Pd-catalyzed allylation reactions.^18^

## Results and Discussion

The synthesis of chiral DSIs through Pd-catalyzed cyclization reactions was investigated by using different solvents and different CPA derivatives as cocatalysts (Tables 1 and Table S1). Initially, we explored the KR of racemic phenylsulfonyl amine **1aa** by using 10% Pd­(PPh_3_)_4_ and 10% **CPA1** [*G* = 2,4,6-(Cy)_3_C_6_H_2_] as catalysts in CH_2_Cl_2_ at room temperature. The reaction resulted in the formation of the desired DSI product **2aa**, which had a conversion (Conv.) rate of 48% and a diastereomeric ratio (dr) of 1:1 (Entry 1, Table 1). The enantiomeric ratio (er) of recovered (*R*)-**1aa** was 78:22, which corresponded to a selectivity factor (s-factor) of 7.1.

**Table 1:**
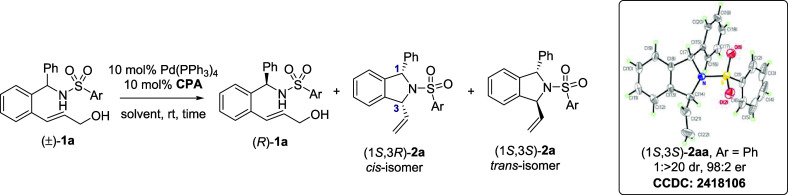
Optimization Table for DSI Synthesis and X-ray Crystallographic Structure of (1*S*,3*S*)-**2aa** with Thermal Ellipsoids at a 50% Probability Level^a^

Entry	Ar	CPA	solvent	time	Conv.	recovery of **1**	er of **1**	yield of **2**	dr of **2** (*cis*/*trans*)	er of *cis*-**2**	er of *trans*-**2**	s*-*factor
1	Ph (**1aa**)	**CPA1**	DCM	2 h	48%	-	78.1:21.9	-	1:1	-	-	7.1
2	Ph (**1aa**)	**-**	DCM	2 h	N.R.^f^	-	-	-	-	-	-	-
3^b^	Ph (**1aa**)	**CPA1**	DCM	2 h	N.R.^f^	-	-	-	-	-	-	-
4	Ph (**1aa**)	**CPA1**	CHCl_3_	3 h	65%	-	92.6:7.4	-	1.6:1	-	-	6.8
5	Ph (**1aa**)	**CPA1**	DCE	2 h 10 min	31%	34%	66.8:33.2	27%	1:1.2	90.2:9.8	89.6:10.4	9.6
6	Ph (**1aa**)	**CPA1**	benzene	1 h 10 min	75%	-	83.0:17.0	-	1:1.5	-	-	2.8
7	Ph (**1aa**)	**CPA1**	ether	3 h	74%	-	67.0:33.0	-	1.8:1	-	-	1.7
8	Ph (**1aa**)	**CPA2**	DCE	1 h 40 min	<5%	-		-		-	-	-
9	Ph (**1aa**)	**CPA3**	DCE	2 h	19%	-	54.0:46.0	-	1.1:1	-	-	2.2
10	Ph (**1aa**)	**CPA4**	DCE	3 h 30 min	42%	-	70.9:29.1	-	1.3:1	-	-	5.6
11	Ph (**1aa**)	**CPA5**	DCE	6 h	41%	-	69.0:31.0	-	1.1:1	-	-	4.9
12	Ph (**1aa**)	**CPA6**	DCE	9 h 30 min	13%	-	50.1:49.9	-	1.3:1	-	-	1.0
13	Ph (**1aa**)	**CPA7**	DCE	14 h	47%	-	58.6:41.4	-	2.0:1	-	-	1.7
14	4-*t-*Bu-C_6_H_4_ (**1ab**)	**CPA1**	DCE	6 h 15 min	60%	41%	94.2:5.8	22%	1:2.0^h^	84.5:15.5	84.2:15.8	10.9
15	4-F-C_6_H_4_ (**1ac**)	**CPA1**	DCE	4 h 30 min	31%	47%	69.4:30.6	23%	1:1.2^h^	91.1:8.9	90.8:9.2	20.0
16	4-CF_3_–C_6_H_4_ (**1ad**)	**CPA1**	DCE	1 h 55 min	52%	31%	88.4:11.6	25%	1.2:1	89.1:10.9	87.3:12.7	13.4
17	2-CF_3_–C_6_H_4_ (**1ae**)	**CPA1**	DCE	20 min	35%	64%	60.8:39.2	16%	1:1.2	80.1:19.9	79.3:20.7	2.9
18	2-CH_3_–C_6_H_4_ (**1af**)	**CPA1**	DCE	4 h 40 min	22%^g^	67%	62.5:37.5	17%	1:2.0	93.2:6.8	92.6:7.4	21.2
19	2,4,6-(CH_3_)_3_–C_6_H_2_ (**1ag**)	**CPA1**	DCE	4 h	N.R^f^	-	-	-	-	-		-
20	3-CF_3_–C_6_H_4_ (**1ah**)	**CPA1**	DCE	3 h	42%	56%	81.2:18.8	34%	1.2:1	92.4:7.6	92.7:7.3	25.5
21^c^	3-CF_3_–C_6_H_4_ (**1ah**)	**CPA1**	DCE	4 h 15 min	33%	-	70.5:29.5	-	1.1:1	91.3:8.7	91.1:8.9	16.3
22^d^	3-CF_3_–C_6_H_4_ (**1ah**)	**CPA1**	DCE	4 h 15 min	32%	-	70.0:30.0	-	1.6:1	93.9:6.1	91.9:9.1	18.2
23^e^	3-CF_3_–C_6_H_4_ (**1ah**)	**CPA1**	DCE	4 h	30%	-	66.9:33.1	-	1.1:1	90.5:9.5	90.8:9.2	11.7

a Reaction conditions: **1a** (1.0 equiv), Pd­(PPh_3_)_4_ (0.1 equiv), CPA (0.1 equiv), solvent (0.01 M), rt, in a nitrogen atmosphere. The Conv. of the KR reactions and the dr value of cyclized product **2a** were determined through ^1^H NMR spectroscopy. The er value was determined using a HPLC system equipped with a chiral column. The recovery of the starting material **1a** and the yield of the cyclized products **2a** were calculated from the weights of **1a** and **2a**, respectively, after column chromatography. The s-factor was calculated using the following equation: ln­[(1 – Conv.)­(1 – ee_s_)]/ln­[(1 – Conv.)­(1 + ee_s_)].

b In the absence of Pd­(PPh_3_)_4_.

c 5 mol % of Pd­(PPh_3_)_4_ and 5 mol % of **CPA1**.

d 5 mol % of Pd­(PPh_3_)_4_ and 10 mol % of **CPA1**.

e 10 mol % of Pd­(PPh_3_)_4_ and 5 mol % of **CPA1**.

f N.R. = No reaction.

g The Conv. value of the low-reactivity reactions (Conv. < 25%) was determined using the following equation: Conv. = ee_s_/(ee_s_ + ee_p_).

h Determined by HPLC analysis.
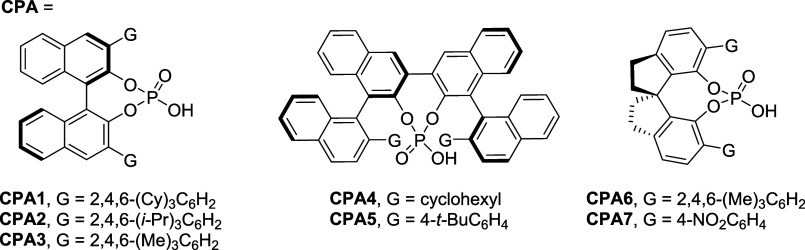

The absolute configurations of (*R*)-**1aa** and the *cis*-(1*S*,3*R*)-isomer in **2aa** were confirmed by comparing their high-performance liquid chromatography (HPLC) profiles with those of authentic compounds, which were prepared in accordance with a previously reported procedure.^9d^ The *trans*-isomer in **2aa** was recrystallized to achieve an er of 98:2 and a dr of 1:>20 and analyzed through single-crystal X-ray diffraction, which revealed its absolute configuration to be 1*S*,3*S*.

Control experiments conducted without Pd or CPA catalysts exhibited no reactivity, indicating that both catalysts were essential for the cyclization reaction (Entries 2 and 3, Table 1). Changing the chlorinated solvent to CHCl_3_ and 1,2-dichloroethane (DCE) resulted in an enhanced s-factor of 9.6 (Entries 4 and 5, Table 1). Further screening of aromatic and ethereal solvents (benzene and diethyl ether) and chiral catalysts [1,1′-bi-2-naphthol (BINOL)- and spiro[4.4]­nonane-1,6-diol (SPINOL)-based **CPA2–7**] did not improve the s-factor (Entries 6–13, Table 1).

We then examined the effect of various aryl groups (Ar) in the sulfonamide moiety in substrate **1** on the stereoselectivity of the KR reactions (**1ab**–**1ah**; Entries 14–20, Table 1). Introducing 4-substituents, such as 4-*tert*-butyl (**1ab**, Ar = 4-*t*-Bu-C_6_H_4_), 4-fluoro (**1ac**, Ar = 4-F-C_6_H_4_), and 4-trifluoromethyl (**1ad**, Ar = 4-CF_3_–C_6_H_4_) on the phenyl ring in the aryl sulfonamide moiety improved the s-factor to up to 20.0 (Entries 14–16, Table 1).

Sterically hindered 2-substituted and 2,4,6-trisubstituted aryl sulfonamide substrates were also evaluated under the standard reaction conditions (**1ae**–**1ag**; Entries 17–19, Table 1). The KR reaction of **1ae** (Ar = 2-CF_3_–C_6_H_4_) demonstrated high reactivity (conversion = 35% in 20 min) but low stereoselectivity (s-factor = 2.9; Entry 17). The enhanced reactivity could be attributed to the higher acidity of the sulfonamide proton, which facilitated deprotonation by the phosphate anion derived from CPA during the nucleophilic attack step of the cyclization reaction. By contrast, 2-methylphenyl (**1af**, Ar = 2-CH_3_–C_6_H_4_) and 2,4,6-trimethylphenyl (**1ag**, Ar = 2,4,6-(CH_3_)_3_–C_6_H_2_) exhibited reduced reactivity (Conv. < 22%); however, the KR reaction of **1af** exhibited a high s-factor of 21.2 (Entries 18 and 19, Table 1).The reduced reactivity of **1af** and **1ag** was likely caused by the diminished nucleophilicity of the hindered aryl sulfonamide group.

The stereoselectivity of the KR reaction was further enhanced by changing the position of the trifluoromethyl group to the 3-position on the phenyl ring within the Ar group (**1ah**, Ar = 3-CF_3_–C_6_H_4_; Entry 20, Table 1). The KR reaction of **1ah** exhibited a high s-factor of 25.5, resulting in the production of chiral DSI **2ah** (er, 92.4:7.6; dr, 1.2:1) and the recovery of chiral amine **1ah** (er, 81.2:18.8). The KR reaction was also performed under different catalyst ratios or loadings, with reduced s-factors ranging from 11.7 to 18.2 being obtained (Entries 21–23, Table 1). These results suggest that the ratio of Pd to CPA catalysts was 1:1, which aligned with the mechanism proposed in earlier reports.^15,16,19^

After determining the optimal conditions for the cyclization reaction, we examined the substrate scope (Tables 2 and S2). Initially, we evaluated the substrates containing a 4-substituted phenyl group at the R^1^ position, namely **1bh**–**1dh**. The allylation reactions of these substrates yielded the desired products, namely, **2bh** (R^1^ = 4-OCH_3_–C_6_H_4_), **2ch** (R^1^ = 4-F-C_6_H_4_), and **2dh** (R^1^ = 4-CF_3_–C_6_H_4_), with high s-factors ranging from 14.5 to 31.3 and yields ranging from 35% to 42%. Subsequently, the substituent at the R^1^ position was replaced with a 3-substituted phenyl group [3-OCH_3_–C_6_H_4_ (**1eh**) and 3-CH_3_–C_6_H_4_ (**1fh**)]. The two cyclization reactions yielded the DSI products **2eh** and **2fh**, with the corresponding s-factors being 22.2 and 48.5, respectively. The cyclization of substrates containing different aryl groups [3,5-(CF_3_)_2_C_6_H_3_, 1,3-benzodioxolyl, and 2-naphthyl, **1gh**–**1ih**] also proceeded smoothly, yielding moderate-to-excellent s-factors ranging from 6.1 to 116.5, dr values around 1:1.6, and Conv. values between 39% and 69%. The formation of the DSIs **2jh** and **2kh** with 5-methyl and 5-fluoro substituents resulted in favorable s-factors of 42.0 and 46.1, respectively. Finally, to assess the limitations of the adopted methodology, testing was conducted for substrate **1lh**, which has a methyl group at the R^1^ position. The reaction successfully yielded the desired DSI product **2lh** with a high er value of 90:10 and a dr value of 5.5:1 (s-factor = 13.6, conversion = 17%).

**Table 2:**
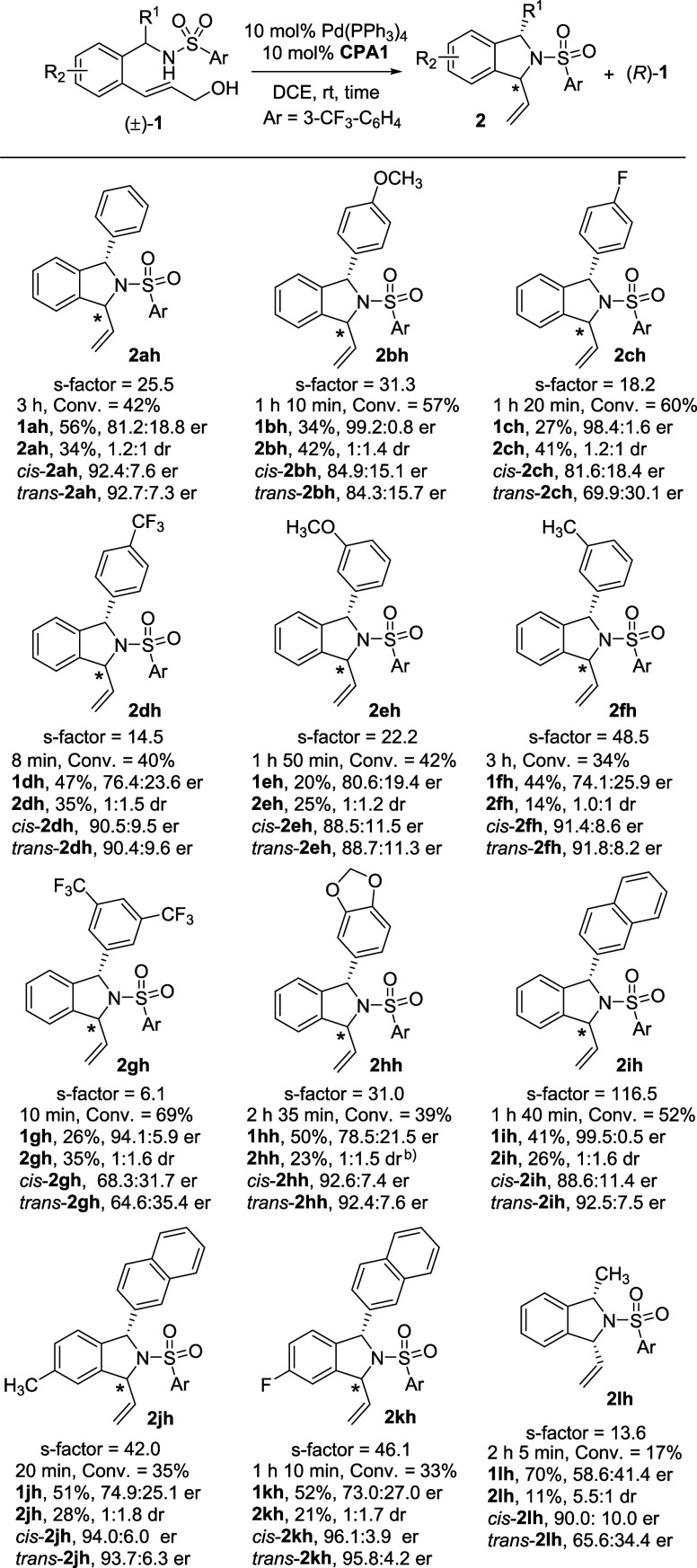
Substrate Scope in DSI Synthesis^a^

a Reaction conditions: **1** (1.0 equiv), Pd­(PPh_3_)_4_ (0.1 equiv), **CPA1** (0.1 equiv), DCE (0.01 M), rt, in a nitrogen atmosphere. The Conv. of the KR reactions and the dr of cyclized product **2** were determined through ^1^H NMR spectroscopy. The er was determined using a HPLC system equipped with a chiral column. The recovery of the starting material **1** and the yield of the cyclized products **2** were calculated from the weights of **1** and **2**, respectively, after column chromatography. The s-factor was calculated using the following equation: ln­[(1 – Conv.)­(1 – ee_s_)]/ln­[(1 – Conv.)­(1 + ee_s_)].

b The dr value was determined by HPLC analysis.

Stereodivergent allylation reactions have emerged as a powerful strategy for selectively obtaining all possible stereoisomeric products with two chiral centers.^20^ In previous reports, a combination of chiral ligands and other chiral components (e.g., chiral amines and chiral metal cocatalysts) was required to independently control the formation of each chiral center during the allylation reaction. In this study, we employed a new approach that requires only enantiomeric CPA catalysts as a chiral source for stereodivergent reactions (Scheme 2; see Supporting Information for further details).

**Scheme 2:**
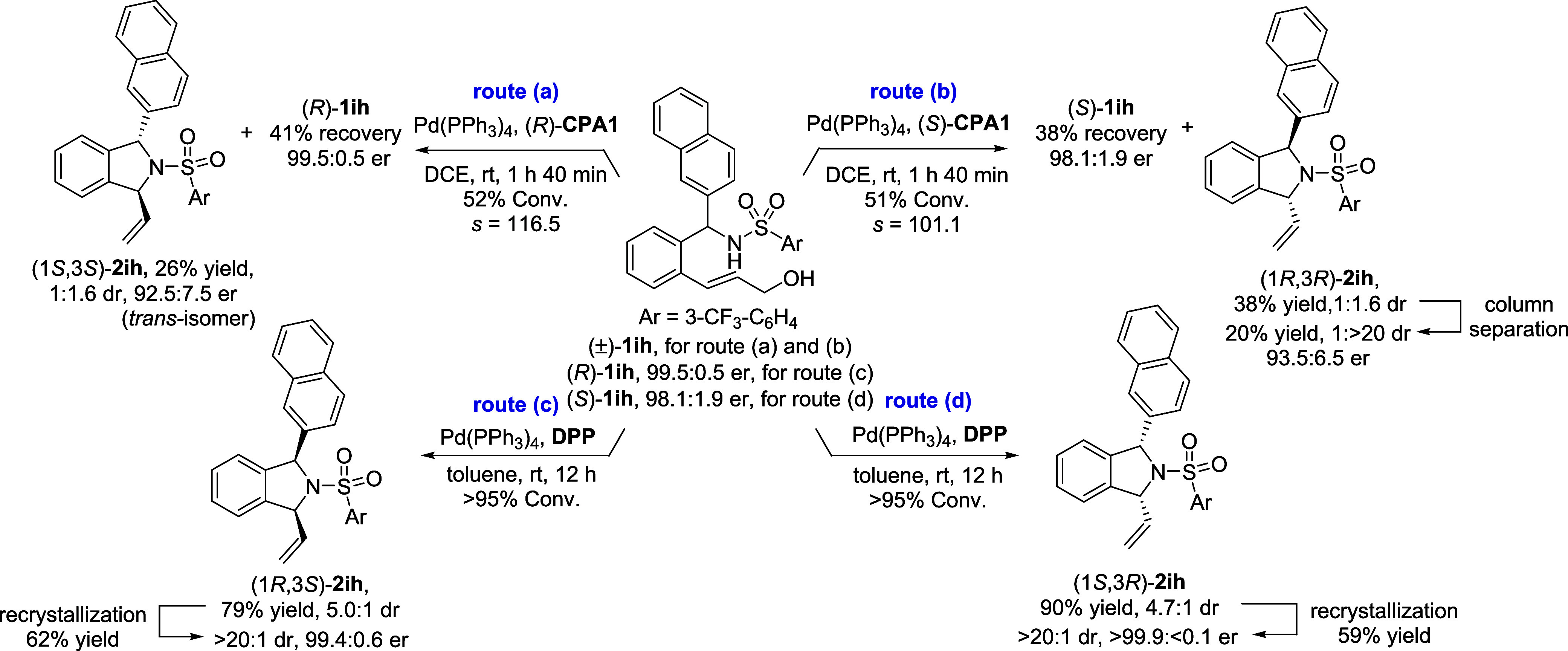
Stereodivergent Synthesis of DSIs

The chiral *trans*-DSIs, (1*S*,3*S*)- and (1*R*,3*R*)-**2ih**, were obtained as the major stereoisomer through the Pd-catalyzed KR reactions of racemic **1ih** by using enantiomeric (*R*)- and (*S*)-**CPA1** as a cocatalyst, respectively [route (a) and (b), Scheme 2]. The enantioenriched amines (*R*)- and (*S*)-**1ih**, recovered from the KR reactions, were subjected to Pd-catalyzed allylation with diphenyl phosphate (DPP), employed as an achiral BA cocatalyst, to afford *cis*-DSIs [routes (c) and (d), Scheme 2].^21^ Notably, all diastereoisomeric *cis*- and *trans*-DSIs **2ih** could be further separated through SiO_2_ column chromatography or recrystallization. Thus, all possible stereoisomers were independently obtained at high optical purities by using the proposed method (all er >90:10).

We propose that Pd/BA-catalyzed DSI synthesis involves four distinct steps (Scheme 3a).^15,19^ (1) Substrate **1** reacts with Pd­(PPh_3_)_2_ and the BA catalyst. The BA catalyst interacts with both the sulfonamide and hydroxyl groups in the substrate, leading to the formation of the Pd(0)−π–olefin complex **I**. (2) Complex **I** undergoes dehydroxylation to generate a Pd­(II)−π–allyl intermediate **II**. (3) The phosphate derived from the BA catalyst functions as a weak base and facilitates the intramolecular nucleophilic attack of the sulfonamide group in intermediate **II**, resulting in the formation of intermediate **III**. (4) Decomplexation of intermediate **III** produces the desired DSI product **2**, with the simultaneous regeneration of Pd and the BA catalyst.

**Scheme 3:**
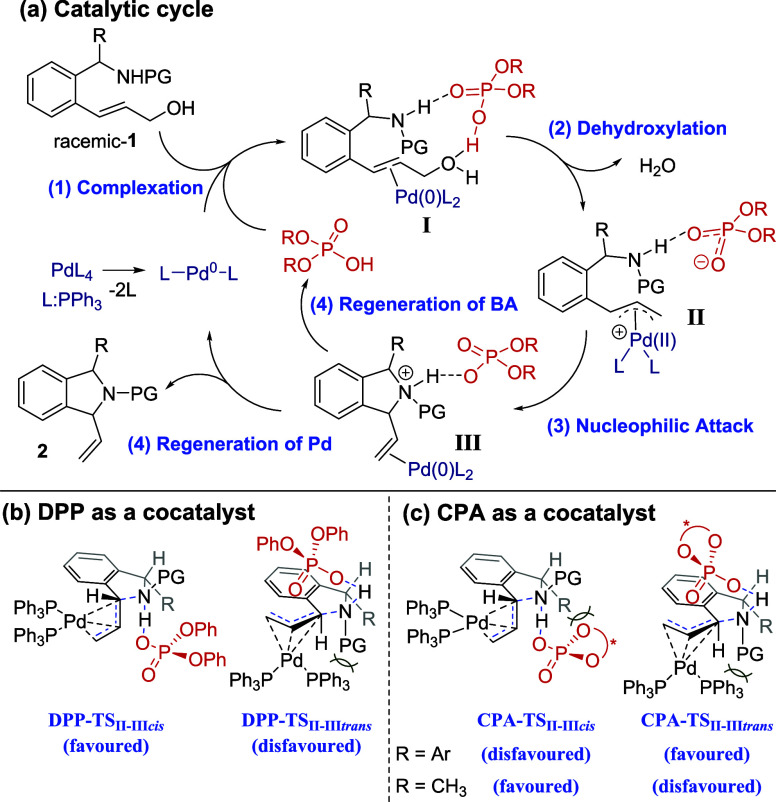
Proposed Catalytic Cycle and Stereochemical Model for the Pd/Brønsted Acid–Catalyzed Reaction

In steps (1) and (2) of the KR process, in which (*R*)-CPA is used as a BA cocatalyst, the (*R*)-**1** enantiomer is selectively excluded for the formation of intermediate **II**, resulting in the recovery of (*R*)-**1** at the end of the reaction.

Scheme 3b,c depicts stereochemical models of transition states (TSs), which rationalize the *cis*–*trans* selectivity during the nucleophilic attack of sulfonamide in step (3). Scheme 3b illustrates the observed *cis*-selectivity of the allylation reaction with DPP as a cocatalyst. The **DPP-TS**_**II–II** *cis*_ pathway is preferred due to the unfavorable steric repulsion between the protecting group (PG) and the Pd-bound ligand in **DPP-TS**_**II–III** *trans*_. Scheme 3c presents the TSs for the allylation reaction involving CPA as a bulkier BA cocatalyst. With a bulky Ar group (e.g., **2gh-2kh**) as the R substituent, **CPA-TS**_**II–III** *trans*_ is preferred due to the stronger steric repulsion between the CPA backbone and the Ar group compared with the interaction between the PG and the ligand in **CPA-TS**_**II–III** *cis*_. By contrast, with a smaller CH_3_ group (e.g., **2lh**) as the R substituent, the steric interaction is dominant in **CPA-TS**_**II–III** *trans*_, favoring the formation of the *cis*-isomer.

## Conclusions

In summary, we conducted the KR reaction of racemic amines through a Pd/CPA-catalyzed stereoselective intramolecular allylation. The reaction has a favorable selectivity factor and yields chiral 1,3-DSIs. The recovered enantioenriched amines are readily converted into chiral *cis*-DSIs through allylation by using combined achiral Pd and BA catalysts. After investigating the effect of the BA catalyst on stereochemical outcomes, we applied Pd/BA-catalyzed allylation to the stereodivergent synthesis of all four possible stereoisomeric DSIs, with a pair of enantiomeric CPA catalysts as the sole chiral source. The proposed stereodivergent synthesis strategy is expected to be applicable to a variety of asymmetric KR reactions, the diastereoselectivity of which can be switched by adjusting cocatalysts, ligands, or solvents.^18^ Consequently, the production of stereoisomeric bioactive molecules will be considerably accelerated, enabling further investigations regarding the effects of chirality on the bioactivity of these molecules.

## Supplementary Material



## Data Availability

The data underlying this study are available in the published article and its Supporting Information.

## Notes

NMR spectra and HPLC data for all new compound (PDF)
