# Supplementary material for: Kinetic Resolution of Racemic Amines via Palladium/Chiral Phosphoric Acid-Catalyzed Intramolecular Allylation: Stereodivergent Access to Chiral 1,3-Disubstituted Isoindolines
Source: J Org Chem. 2025 Jun 12;90(25):8578–84. doi: 10.1021/acs.joc.5c00568 (PMC12210262; doi:10.1021/acs.joc.5c00568)
Supplement: Supplementary file 1 [file jo5c00568_si_001.pdf]

Supporting Information for

Kinetic Resolution of Racemic Amines via Palladium/Chiral Phosphoric Acid-Catalyzed Intramolecular Allylation: Stereodivergent Access to Chiral 1,3-Disubstituted Isoindolines

Bing-Syuan Wu, Yu-Ming Lin, and Cheng-Che Tsai*

Department of Chemistry, Tunghai University, Taichung City 40704, Taiwan

Email: chengchetsai@thu.edu.tw

Table of Contents

1. General Information S3

2. Synthesis of Substrates **1** S4

3. Optimization for Kinetic Resolution Reactions S9

4. Substrate Scope in DSI Synthesis S10

5. Determination of the Absolute Configuration of Chiral Compounds in the KR Reactions ... S29

6. Stereodivergent Synthesis of DSIs S31

7. References S33

8. X-Ray Crystallographic data of Compound *trans*-**2aa** S34

9. NMR Sepctra S41

10. HPLC Data S170

1. General Information

Unless otherwise noted, all reactions were performed in a round-bottom flask with rubber septa and were stirred with Teflon-coated magnetic stir bars. Dry Et_2O, THF, CH_2Cl_2, and toluene were obtained by passing these previously degassed solvents through activated alumina columns. Dry $CHCl_3$ and 1,2-dichloroethane (DCE) were distilled from calcium hydride. All reagents were obtained from commercial sources and used without further purification. Thin-layer chromatography (TLC) analysis of reaction mixture was performed on Merck silica gel 60 F254 plates and visualized through ultraviolet (UV) irradiation and staining with ceric ammonium molybdate or $KMnO_4$. Column chromatography was carried out using KM3 Silica Gel with a particle size of 45–75 μm. Volatile solvents were removed under reduced pressure with a rotary evaporator and dried on a high-vacuum Schlenk line. Nuclear magnetic resonance (NMR) spectra were recorded using Bruker Ascend 400 MHz spectrometers. The NMR data are reported as chemical shift (multiplicity, coupling constants where applicable, number of hydrogens). Chemical shifts are reported in parts per million and relative to the residual solvent signal ($CDCl_3$: δ_H 7.26 and δ_C 77.0, CD_3OD: δ_H 3.31 and δ_C 49.0). High-resolution mass spectra (HRMS) were recorded by an Impact HD Q-TOF mass spectrometer (Bruker, Germany). The optical rotation was determined on a KRUSS P3000 Polarimeter at 25 °C and is reported as $[\alpha]_D^{25}$, concentration (g/100 mL), and solvent. Unless otherwise noted, chiral phosphoric acids (CPAs) were purchased from BLD Pharmatech and used without further purification. **CPA4** and **CPA5** and were synthesized according to a literature procedure.[1]

2. Synthesis of Substrates 1

2.1 General procedure A for the synthesis of racemic substrate 1aa-1ah

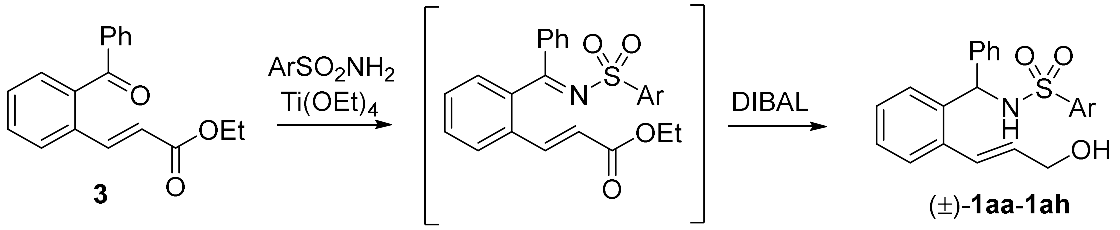

The following preparation of (±)-**1aa** is representative. $Ti(OEt)_4$ (1.5 mL, 7.13 mmol, 4.0 equiv.) and benzenesulfonamide (336 mg, 2.14 mmol, 1.2 equiv.) were added to a solution of compound **3**[2] (500 mg, 1.78 mmol, 1.0 equiv.) in dry toluene (3.6 mL, 0.5 M). After 16 h of stirring at reflux, the reaction mixture was cooled to room temperature and filtered through a celite pad with EtOAc as an eluent. The filtrate was concentrated to afford sulfonyl imine as a crude product, which was used for the next step without further purification.

DIBAL (3.0 mL, 1.2 M in toluene, 3.57 mmol, 2.0 equiv.) was added to a solution of the sulfonyl imine in dry Et_2O (8.9 mL, 0.2 M) at 0 °C. The reaction mixture was stirred at 0 °C for 2 hours, then treated with Brine and filtered through a Celite pad using EtOAc as the eluent. The filtrate was concentrated and purified by column chromatography [EtOAc:hexanes, 1:1 (v/v)], affording compound (±)-**1aa** (301 mg, 45% over 2 steps) as a yellow solid.

(*E*)-*N*-{[2-(3-Hydroxyprop-1-en-1-yl)phenyl](phenyl)methyl}benzenesulfonamide [(±)-**1aa**]

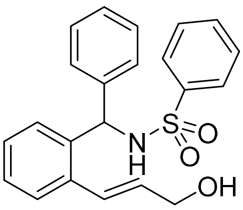

R_f = 0.57 [EtOAc:hexanes, 1:1 (v/v)]. Mp 82−83 °C. ^1H NMR ($CDCl_3$, 400 MHz) δ 7.59 (d, *J* = 7.4 Hz, 2H), 7.41 (t, *J* = 7.4 Hz, 1H), 7.33−7.25 (m, 3H), 7.19−7.15 (m, 4H), 7.10−7.04 (m, 3H), 6.95 (d, *J* = 7.7 Hz, 1H), 6.76 (d, *J* = 15.6 Hz, 1H), 6.08 (dt, *J* = 15.6, 5.4 Hz, 1H), 5.96 (d, *J* = 7.7 Hz, 1H), 5.48 (s, 1H), 4.22 (d, *J* = 5.4 Hz, 2H). $^{13}C\{^1H\}$ NMR ($CDCl_3$, 101 MHz) δ 140.2, 139.5, 136.9, 135.9, 132.6, 132.3, 128.6, 128.4, 128.1, 128.0, 127.7, 127.6, 127.5, 127.3, 127.2, 126.9, 63.4, 58.4. HRMS (ESI) m/z: $[M + Na]^+$ Calcd for $C_{22}H_{21}NNaO_3S$ 402.1134; Found 402.1134.

2.2 General procedure B for the synthesis of racemic substrates 1bh-1kh, 1ai, and 1aj

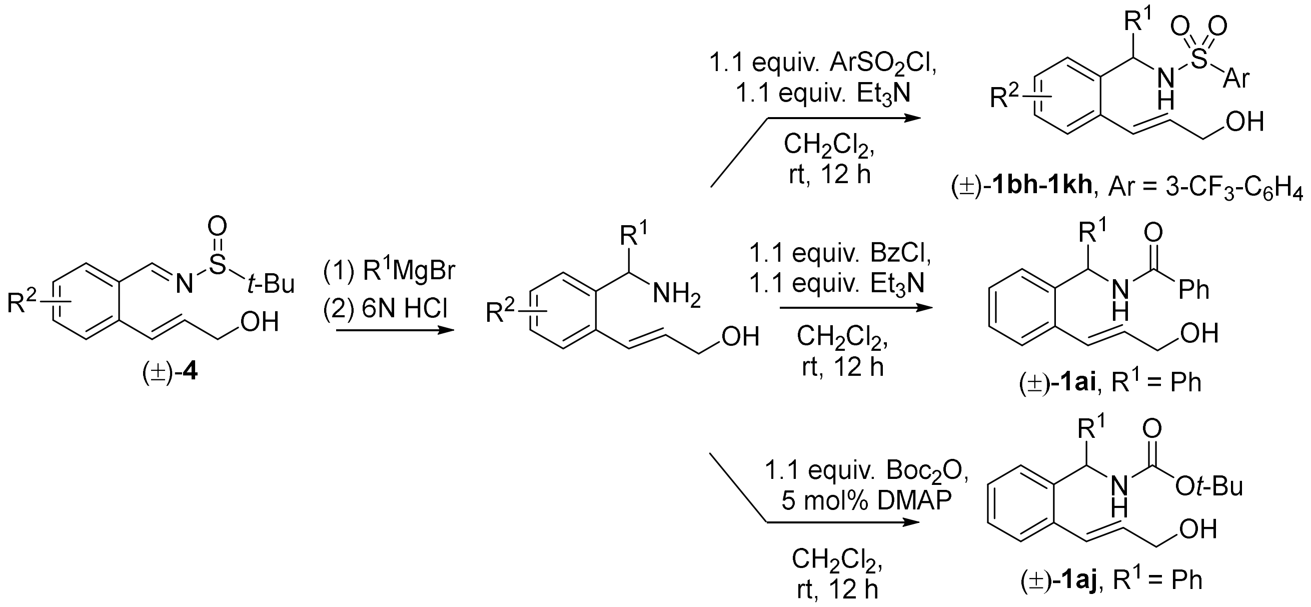

The following preparation of (±)-**1eh** is representative. Grignard reagent (13.11 mmol, 4.0 equiv.) was slowly added to a solution of (±)-sulfinyl imine **4**[3] (870 mg, 3.28 mmol, 1.0 equiv.) in dry THF (11 mL, 0.3 M) at 0 °C. The reaction mixture was stirred at 0 °C for 2 hours, then concentrated under reduced pressure. The residue was dissolved in EtOAc and washed with H_2O. The organic layer was dried over $MgSO_4$, concentrated, and purified by column chromatography [EtOAc:hexanes, 1:1 (v/v)], affording sulfinyl amine.

6N HCl (10.9 mL, 65.57 mmol, 20.0 equiv.) was slowly added to a solution of the sulfinyl amine in THF (33 mL, 0.1 M) at 0 ºC. After 1 h of stirring at 0 ºC, the reaction mixture was concentrated, redissolved in CH_2Cl_2, and neutralized with NaOH (10 M aq.). The organic layers were concentrated under reduced pressure to afford crude desulfinylation product, which was used for next step without further purification.

Et_3N (0.5 mL, 3.61 mmol, 1.1 equiv.) and 3-$CF_3C_6H_4SO_2Cl$ (0.6 mL, 3.61 mmol, 1.1 equiv.) was added to a solution of the desulfinylation product in CH_2Cl_2 (33 mL, 0.1 M). After 12 h of stirring at room temperature, the reaction mixture was concentrated under reduced pressure and purified using column chromatography, yielding compound (±)-**1eh** (336 mg, 20% over 3 steps) as a brown oil.

(*E*)-*N*-{[2-(3-Hydroxyprop-1-en-1-yl)phenyl](3-methoxyphenyl)methyl}-3-(trifluoromethyl)benzenesulfonamide [(±)-**1eh**]

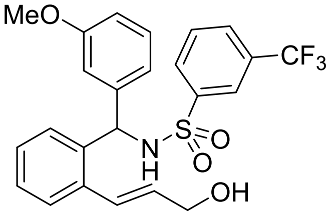

R_f = 0.59 [EtOAc:hexanes, 1:1 (v/v)]. ^{1}H NMR ($CDCl_3$, 400 MHz) δ 7.77 – 7.74 (m, 2H), 7.62 (d, *J* = 7.8 Hz, 1H), 7.39 (t, *J* = 7.8 Hz, 1H), 7.31 (d, *J* = 7.6 Hz, 1H), 7.16 (t, *J* = 7.4 Hz, 1H), 7.11 (t, *J* = 7.9 Hz, 1H), 7.03 (t, *J* = 7.6 Hz, 1H), 6.89 (d, *J* = 7.8 Hz, 1H), 6.82 (d, *J* = 15.6 Hz, 1H), 6.72 (dd, *J* = 8.2, 2.3 Hz, 1H), 6.67 (d, *J* = 7.9 Hz, 1H), 6.63 (s, 1H), 6.10 (dt, *J* = 15.6, 5.3 Hz, 1H), 5.98 (d, *J* = 7.9 Hz, 1H), 5.61 (s, 1H), 4.28 (d, *J* = 5.3 Hz, 2H), 3.67 (s, 3H).

$^{13}C\{^1H\}$ NMR ($CDCl_3$, 101 MHz) δ 159.7, 141.5, 140.3, 136.02, 135.98, 132.7, 131.0 (q, $J_{C\text{–}F}$ = 33.4 Hz), 130.0, 129.6, 129.3, 128.8 (q, $J_{C\text{–}F}$ = 3.0 Hz), 128.3, 128.1, 127.6, 127.4, 127.3, 123.9 (q, $J_{C\text{–}F}$ = 3.7 Hz), 123.0 (q, $J_{C\text{–}F}$ = 272.8 Hz), 119.6, 113.2, 112.9, 63.3, 58.7, 55.1. ^{19}F NMR ($CDCl_3$, 376 MHz) δ –62.82 (s). HRMS (ESI) m/z: $[M + Na]^+$ Calcd for $C_{24}H_{22}F_3NNaO_4S$ 500.1114; Found 500.1109.

(*E*)-*N*-{[2-(3-Hydroxyprop-1-en-1-yl)phenyl](phenyl)methyl)}benzamide [(±)-**1ai**]

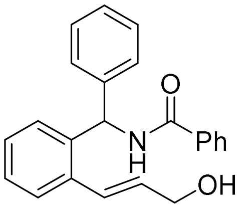

Through a reaction sequence involving Grignard addition, desulfinylation, and benzoylation of (±)-sulfinyl imine **4** (377 mg, 1.42 mmol, 1.0 equiv.), (±)-**1ai** was obtained as a colorless oil (50 mg, 10% over 3 steps). R_f = 0.39 [EtOAc:hexanes, 1:1 (v/v)]. ^1H NMR ($CDCl_3$, 400 MHz) δ 7.80 (d, *J* = 7.5 Hz, 2H), 7.53 – 7.41 (m, 4H), 7.36 – 7.22 (m, 7H), 7.14 (d, *J* = 7.5 Hz, 1H), 6.92 (d, *J* = 15.6 Hz, 1H), 6.75 – 6.70 (m, 2H), 6.17 (dt, *J* = 15.6, 5.3 Hz, 1H), 4.21 (d, *J* = 4.0 Hz, 2H). $^{13}C\{^1H\}$ NMR ($CDCl_3$, 101 MHz) δ 166.4, 140.8, 138.5, 136.7, 134.0, 132.4, 131.8, 128.7, 128.2, 128.0, 127.8, 127.51, 127.47, 127.4, 127.3, 127.0, 63.5, 54.3. HRMS (ESI) m/z: $[M + Na]^+$ Calcd for $C_{23}H_{21}NNaO_2$ 366.1465; Found 366.1464.

tert-Butyl (*E*)-{[2-(3-hydroxyprop-1-en-1-yl)phenyl](phenyl)methyl}carbamate [(±)-**1aj**]

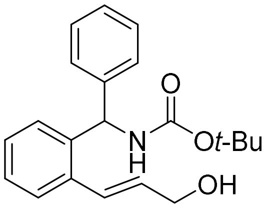

Through a reaction sequence involving Grignard addition, desulfinylation, and Boc protection of (±)-sulfinyl imine **4** (169 mg, 0.64 mmol, 1.0 equiv.), (±)-**1aj** was obtained as a colorless oil (23 mg, 11% over 3 steps). R_f = 0.81 [EtOAc:hexanes, 1:1 (v/v)]. ^1H NMR ($CDCl_3$, 400 MHz) δ 7.42 – 7.39 (m, 1H), 7.25 – 7.13 (m, 8H), 6.82 (d, *J* = 15.5 Hz, 1H), 6.25 (s, 1H), 6.05 – 5.97 (m, 1H), 5.24 (d, *J* = 10.5 Hz, 1H), 4.57 (t, *J* = 8.8 Hz, 2H), 1.42 (d, *J* = 5.7 Hz, 9H). $^{13}C\{^1H\}$ NMR ($CDCl_3$, 101 MHz) δ 156.2, 153.4, 141.4, 139.1, 135.5, 135.4, 131.7, 131.6, 128.6, 128.3, 127.8, 127.3, 127.2, 126.4, 82.2, 67.6, 67.5, 55.5, 27.7. HRMS (ESI) m/z: $[M + Na]^+$ Calcd for $C_{21}H_{25}NNaO_3$ 362.1727; Found 362.1720.

2.3 Operation procedure for the synthesis of racemic substrate 1lh

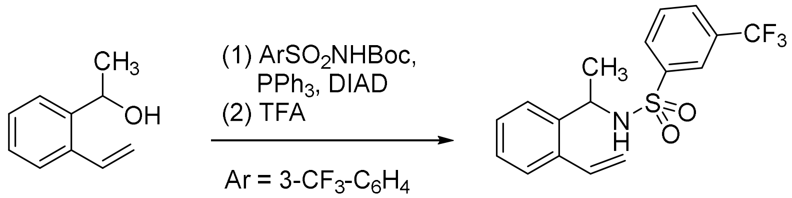

(±)-**5** (±)-**6**

PPh_3 (15.081 g, 57.50 mmol, 1.3 equiv.), $3\text{-}CF_3C_6H_4SO_2NHBoc$ (17.140 g, 48.65 mmol, 1.1 equiv.), and DIAD (9.6 mL, 48.65 mmol, 1.1 equiv.) were added to a solution of alcohol (±)-**5**[4] (6.555 g, 44.23 mmol, 1.0 equiv.) in dry toluene (442 mL, 0.1 M). After 6 h of stirring at room temperature, the reaction mixture was concentrated and purified using column chromatography [EtOAc:hexanes, 1 : 10 (v/v)], yielding Boc-protected sulfonamide product.

TFA (27 mL, 353.83 mmol, 8.0 equiv.) was added to a solution of the Boc-protected sulfonamide in dry CH_2Cl_2 (442 mL, 0.1 M) at 0 °C. After 1 h of stirring at 0 °C, the reaction mixture was concentrated and purified using column chromatography [EtOAc:hexanes, 1 : 5 (v/v)], yielding sulfonamide product (±)-**6** (7.356 g, 47% over 2 steps) as a white solid.

3-(Trifluoromethyl)-*N*-[1-(2-vinylphenyl)ethyl]benzenesulfonamide [(±)-**6**]

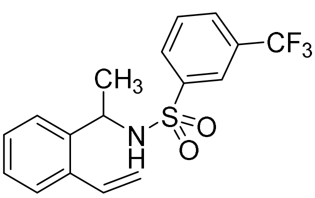

R_f = 0.40 [EtOAc:hexanes, 1:3 (v/v)]. Mp 103−104 °C. ^{1}H NMR ($CDCl_3$, 400 MHz) δ 7.83 (s, 1H), 7.79 (d, *J* = 7.9 Hz, 1H), 7.63 (d, *J* = 7.8 Hz, 1H), 7.40 (t, *J* = 7.8 Hz, 1H), 7.23 (d, *J* = 7.7 Hz, 1H), 7.09−6.98 (m, 3H), 6.91 (dd, *J* = 17.2, 10.9 Hz, 1H), 5.47 (dd, *J* = 17.2, 1.3 Hz, 1H), 5.47 (s, 1H), 5.30 (dd, *J* = 10.9, 1.3 Hz, 1H), 4.93 (q, *J* = 6.9 Hz, 1H), 1.46 (d, *J* = 6.9 Hz, 3H). ^{13}C{^{1}H} NMR ($CDCl_3$, 101 MHz) δ 141.5, 138.0, 135.7, 133.5, 131.1 (q, J_{C-F} = 33.4 Hz), 130.1, 129.3, 128.8 (q, J_{C-F} = 3.4 Hz), 128.0, 127.7, 126.7, 125.5, 124.0 (q, J_{C-F} = 3.8 Hz), 123.1 (q, J_{C-F} = 272.8 Hz), 117.7, 50.0, 23.5. ^{19}F NMR ($CDCl_3$, 376 MHz) δ –62.85 (s). HRMS (ESI) m/z: [M + Na]$^+$ Calcd for $C_{17}H_{16}F_3NNaO_2S$ 378.0746; Found 378.0747.

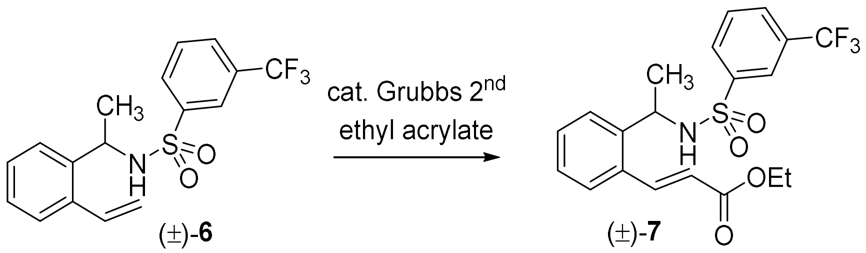

Ethyl acrylate (8.9 mL, 81.60 mmol, 10.0 equiv.) and Grubbs Catalyst, 2nd Generation (256 mg, 0.41 mmol, 0.05 equiv.) were added to a solution of compound (±)-**6** (2.900 g, 8.16 mmol, 1.0 equiv.) in dry toluene (82 mL, 0.1 M). After 10 h of stirring at room temperature, the reaction mixture was concentrated and purified using column chromatography [EtOAc:hexanes, 1 : 5 (v/v)]; consequently, compound (±)-**6** (2.394 g, 83%) was recovered and product (±)-**7** [311 mg, 9% (51%, based on recovered starting material)] was obtained as a brown oil.

Ethyl (*E*)-3-{2-[1-((3-(trifluoromethyl)phenyl)sulfonamido)ethyl]phenyl}acrylate [(±)-**7**]

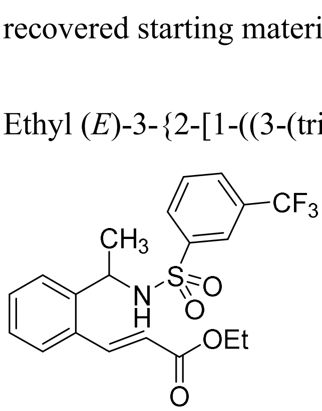

R_f = 0.39 [EtOAc:hexanes, 1:3 (v/v)]. ^{1}H NMR (CD_3OD, 400 MHz) δ 8.02 (dd, *J* = 15.7, 6.4 Hz, 1H), 7.79−7.67 (m, 3H), 7.51−7.46 (m, 1H), 7.40 −7.36 (m, 1H), 7.19−7.15 (m, 1H), 7.12−7.08 (m, 2H), 6.27 (dd, *J* = 15.7, 5.9 Hz, 1H), 4.94−4.89 (m, 2H), 4.27 (q, *J* = 7.1 Hz, 2H), 1.41− 1.32 (m, 6H). ^{13}C{^{1}H} NMR ($CDCl_3$, 101 MHz) δ 166.6, 141.6, 140.7, 139.9, 132.1, 131.1 (q, J_{C-F} = 33.3 Hz), 130.1, 130.0, 129.4, 128.8, 127.8, 127.0, 126.5 (q, J_{C-F} = 3.4 Hz), 123.9 (q, J_{C-F} = 3.6 Hz), 123.0 (q, J_{C-F} = 272.9 Hz), 121.3 (q, J_{C-F} = 4.7 Hz), 60.7, 50.4, 23.7, 14.2. ^{19}F NMR ($CDCl_3$, 376 MHz) δ –62.86 (s). HRMS (ESI) m/z: [M + Na]$^+$ Calcd for $C_{20}H_{20}F_3NNaO_4S$ 450.0957; Found 450.0956.

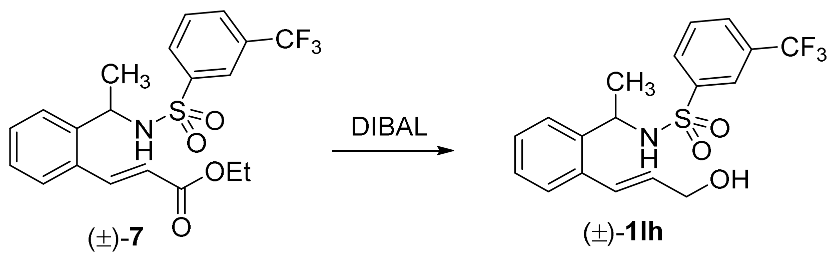

DIBAL (1.3 mL, 1.2 M in toluene, 1.54 mmol, 1.5 equiv.) was added to a solution of (±)-**7** (438 mg, 1.02 mmol, 1.0 equiv.) in dry Et_2O (10 mL, 0.1 M) at 0 °C. The reaction mixture was stirred at 0 °C for 2 hours, then quenched with saturated aqueous $NaHCO_3$ and filtered through a Celite pad using EtOAc as the eluent. The filtrate was concentrated and purified by column chromatography [EtOAc:hexanes, 1:1 (v/v)], affording compound (±)-**1lh** (108 mg, 27%) as a colorless oil.

(*E*)-*N*-{1-[2-(3-Hydroxyprop-1-en-1-yl)phenyl]ethyl}-3-(trifluoromethyl)benzenesulfonamide [(±)-**1lh**]

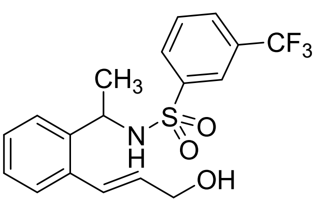

R_f = 0.53 [EtOAc:hexanes, 1:1 (v/v)]. ^{1}H NMR ($CDCl_3$, 400 MHz) δ 7.81 (s, 1H), 7.76 (d, *J* = 8.1 Hz, 1H), 7.63 (d, *J* = 7.7 Hz, 1H), 7.41 (t, *J* = 7.9 Hz, 1H), 7.23 (d, *J* = 7.7 Hz, 1H), 7.10−7.06 (m, 1H), 7.00 (d, *J* = 4.0 Hz, 2H), 6.87 (d, *J* = 15.6 Hz, 1H), 6.08 (dt, *J* = 15.6, 5.2 Hz, 1H), 5.28 (d, *J* = 6.5 Hz, 1H), 4.96 (q, *J* = 6.9 Hz, 1H), 4.33 (dd, *J* = 5.2, 0.7 Hz, 2H), 1.95 (s, 1H), 1.45 (d, *J* = 6.9 Hz, 3H). $^{13}C\{^1H\}$ NMR ($CDCl_3$, 101 MHz) δ 141.7, 137.9, 135.1, 132.5, 131.1 (q, $J_{C\text{–}F}$ = 33.5 Hz), 130.0, 129.4, 128.8 (q, $J_{C\text{–}F}$ = 3.4 Hz), 127.80, 127.77, 127.1, 127.0, 125.7, 123.9 (q, $J_{C\text{–}F}$ = 3.7 Hz), 123.1 (q, $J_{C\text{–}F}$ = 272.9 Hz), 63.4, 50.3, 23.1. ^{19}F NMR ($CDCl_3$, 376 MHz) δ –62.80 (s). HRMS (ESI) m/z: $[M + Na]^+$ Calcd for $C_{18}H_{18}F_3NNaO_3S$ 408.0852; Found 408.0852.

3. Optimization for Kinetic Resolution Reactions

Table S1. Screening of Protecting Groups, CPA Catalysts, and Solvents[a]

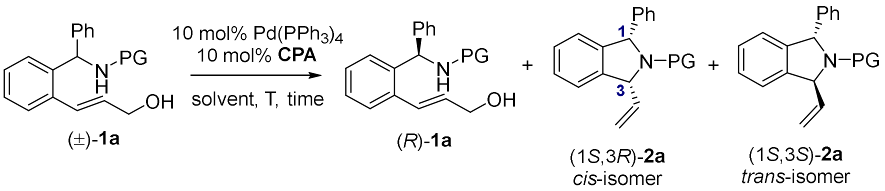

Entry	PG	**CPA**	solvent	T	time	Conv.[b]	er of **1aa**[c]	dr of **2aa** (*cis*/*trans*)[b]	er of *cis*-**2aa**[c]	er of *trans*-**2aa**[c]	*s*-factor[d]
1	SO_2Ph (**1aa**)	**CPA1**	DCM	rt	2 h	48%	78.1:21.9	1.0:1	-		7.1
2	SO_2Ph (**1aa**)	**CPA1**	$CHCl_3$	rt	3 h	65%	92.6:7.4	1.6:1	-		6.8
3	SO_2Ph (**1aa**)	**CPA1**	DCE	rt	2 h 10 min	31%	66.8:33.2	1:1.2	90.2:9.8	89.6:10.4	9.6
4	SO_2Ph (**1aa**)	**CPA1**	ether	rt	3 h	74%	67.0:33.0	1.8:1	-	-	1.7
5	SO_2Ph (**1aa**)	**CPA1**	benzene	rt	1 h 10 min	75%	83.0:17.0	1:1.5	-	-	2.8
6	SO_2Ph (**1aa**)	**CPA1**	toluene	0 °C	1 h 30 min	31%	60.0:40.0	2.0:1	-	-	3.1
7	SO_2Ph (**1aa**)	**CPA2**	DCE	rt	1 h 40 min	<5%	-	-	-	-	-
8	SO_2Ph (**1aa**)	**CPA3**	DCE	rt	2 h	19%	54.0:46.0	1.1:1	-	-	2.2
9	SO_2Ph (**1aa**)	**CPA8**	DCE	rt	1 h 50 min	26%	59.7:40.3	2.0:1	-	-	4.2
10	SO_2Ph (**1aa**)	**CPA9**	DCE	rt	5 h 10 min	24%	54.0:46.0	1.8:1	-	-	1.8
11	SO_2Ph (**1aa**)	**CPA10**	DCE	rt	7 h	21%	58.5:41.5	1:1.3	-	-	5.4
12	SO_2Ph (**1aa**)	**CPA11**	DCE	rt	7 h	41%	56.3:43.7	1.5:1	-	-	1.6
13	SO_2Ph (**1aa**)	**CPA4**	DCE	rt	3 h 30 min	42%	70.9:29.1	1.3:1		-	5.6
14	SO_2Ph (**1aa**)	**CPA5**	DCE	rt	6 h	41%	69.0:31.0	1.1:1	-	-	4.9
15	SO_2Ph (**1aa**)	**CPA6**	DCE	rt	9 h 30 min	13%	50.1:49.9	1.3:1	-	-	1.0
16	SO_2Ph (**1aa**)	**CPA7**	DCE	rt	14 h	47%	58.6:41.4	2.0:1	-	-	1.7
17	SO_2Ph (**1aa**)	**CPA12**	DCE	rt	19 h 30 min	29%	50.7:49.3	1.2:1	-	-	1.1
18	SO_2Ph (**1aa**)	**CPA13**	DCE	rt	14 h	14%	52.3:47.7	1:1.2	-	-	1.9
19	Bz (**1ai**)	**CPA1**	DCE	rt	3 h	<5%	-	-	-	-	-
20	Boc (**1aj**)	**CPA1**	DCE	rt	3 h	-[e]	-	-	-	-	-

[a] Reaction condition: **1a** (1.0 equiv.), $Pd(PPh_3)_4$ (0.1 equiv.), **CPA** (0.1 equiv.), solvent (0.01 M), in a nitrogen atmosphere. [b] Determined by proton nuclear magnetic resonance (^1H NMR) spectroscopy of the crude reaction mixture. [c] Determined by HPLC equipped with a chiral column. [d] s-factor = ln[(1 – Conv.)(1 − ee_s)]/ln[(1 − Conv.)(1 + ee_s)]. [e] Complex mixture. Bz = benzoyl. Boc = *t*-butyloxycarbonyl.

CPA =

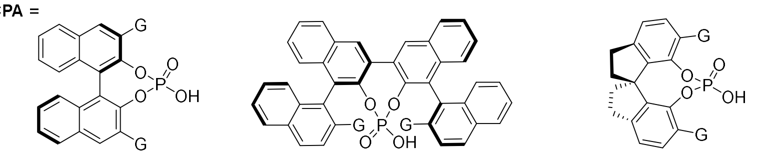

CPA1, G = 2,4,6-$(Cy)_3C_6H_2$
CPA2, G = 2,4,6-$(i\text{-}Pr)_3C_6H_2$
CPA3, G = 2,4,6-$(Me)_3C_6H_2$
CPA8, G = 4-t-BuC_6H_4
CPA9, G = 4-PhC_6H_4
CPA10, G = C_6H_5
CPA11, G = $SiPh_3$

CPA4, G = cyclohexyl
CPA5, G = 4-t-BuC_6H_4

CPA6, G = 2,4,6-$(Me)_3C_6H_2$
CPA7, G = 4-$NO_2C_6H_4$
CPA12, G = 2-naphthyl
CPA13, G = phenanthrenyl

4. Substrate Scope in DSI Synthesis

Table S2. Substrate Scope[a]

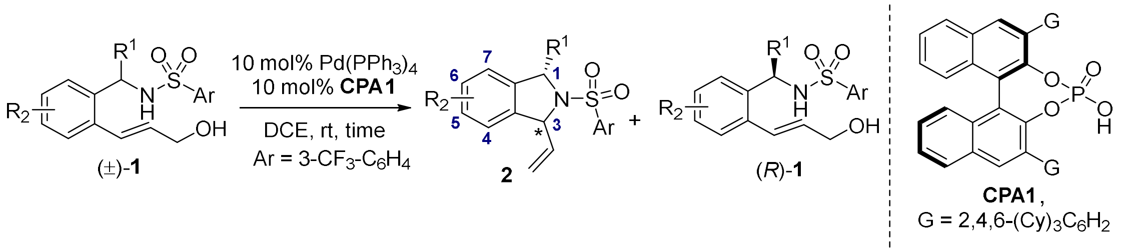

Entry	(±)-1	R[1]	R[2]	time	Conv.[b]	er of 1[c]	recovery of 1 (%)[d]	dr of 2[b] (*cis*/*trans*)	yield of 2 (%)[d]	er of *cis*-2[c]	er of *trans*-2[c]	*s*-factor[e]
1	**1ah**	Ph	H	3 h	42%	81.2:18.8	56	1.2:1	34	92.4:7.6	92.7:7.3	25.5
2	**1bh**	4-OCH$_3$C$_6$H$_4$	H	1 h 10 min	57%	99.2:0.8	34	1:1.4	42	84.9:15.1	84.3:15.7	31.3
3	**1ch**	4-FC$_6$H$_4$	H	1 h 30 min	60%	98.4:1.6	27	1.2:1	41	81.6:18.4	69.9:30.1	18.2
4	**1dh**	4-CF$_3$C$_6$H$_4$	H	8 min	40%	76.4:23.6	47	1:1.5	35	90.5:9.5	90.4:9.6	14.5
5	**1eh**	3-OCH$_3$C$_6$H$_4$	H	1 h 50 min	42%	80.6:19.4	20	1:1.2	25	88.5:11.5	88.7:11.3	22.2
6	**1fh**	3-CH$_3$C$_6$H$_4$	H	3 h	34%	74.1:25.9	44	1.0:1	14	91.4:8.6	91.8:8.2	48.5
7	**1gh**	3,5-(CF$_3$)$_2$C$_6$H$_3$	H	10 min	69%	94.1:5.9	26	1:1.6	35	68.3:31.7	64.6:35.4	6.1
8	**1hh**	Bod[f]	H	2 h 35 min	39%	78.5:21.5	50	1:1.5	23	92.6:7.4	92.4:7.6	31.0
9	**1ih**	2-naphthyl	H	1 h 40 min	52%	99.5:0.5	41	1:1.6	26	88.6:11.4	92.5:7.5	116.5
10	**1jh**	2-naphthyl	5-CH$_3$	20 min	35%	74.9:25.1	51	1:1.8	28	94.0:6.0	93.7:6.3	42.0
11[g]	**1jh**	2-naphthyl	5-CH$_3$	1 h	<5%	-	-	-	-	-	-	-
12	**1kh**	2-naphthyl	5-F	1 h 10 min	33%	73.0:27.0	52	1:1.7	21	96.1:3.9	95.8:4.2	46.1
13	**1lh**	CH$_3$	H	2 h 5 min	17%	58.6:41.4	70	5.5:1	11	90.0:10.0	65.6:34.4	13.6

[a] Reaction condition: **1** (1.0 equiv.), Pd(PPh$_3$)$_4$ (0.1 equiv.), **CPA1** (0.1 equiv.), DCE (0.01 M), in a nitrogen atmosphere. [b] Determined by proton nuclear magnetic resonance (^{1}H NMR) spectroscopy. [c] Determined by HPLC equipped with a chiral column. [d] Calculated from the weights of **1** and **2**, respectively, after column chromatography. [e] s-factor = ln[(1 – Conv.)(1 − ee$_s$)]/ln[(1 − Conv.)(1 + ee$_s$)]. [f] Bod = 1,3-benzodioxolyl. [g] 3Å molecular sieves (300 wt%) were used as an additive in the reaction.

General procedure C for the DSI synthesis via a kinetic resolution reaction

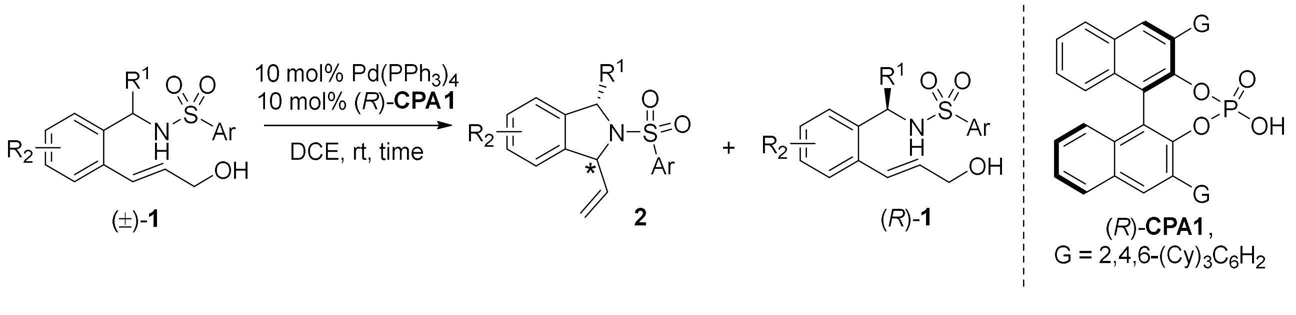

The following reaction of (±)-**1aa** is representative. (±)-**1aa** (30.0 mg, 0.08 mmol, 1 equiv.), $Pd(PPh_3)_4$ (9.1 mg, 0.01 mmol, 0.1 equiv.), and (*R*)-**CPA1** (7.9 mg, 0.01 mmol, 0.1 equiv.) were added to a flame-dried Schlenk tube. The Schlenk tube was evacuated under vacuum, filled with nitrogen, and then charged with dry 1,2-dichloroethane (7.9 mL, 0.01 M) at room temperature. The reaction progress was monitored using crude ^{1}H NMR, and upon reaching the desired conversion, the reaction mixture was filtered through a silica gel pad using EtOAc as the eluent. The filtrate was concentrated and purified by column chromatography [EtOAc:hexanes, 1:3 (v/v)], recovering enantioenriched compound **1** and producing cyclized product **2**.

(*R*,*E*)-*N*-{[2-(3-Hydroxyprop-1-en-1-yl)phenyl](phenyl)methyl}benzenesulfonamide [(*R*)-**1aa**]

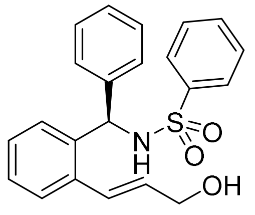

Following the general procedure C, the reaction of (±)-**1aa** (30.0 mg) resulted in the recovery of (*R*)-**1aa** (10.1 mg, 34%). HPLC analysis: Daicel Chiralcel OD−H (absorbance at 254 nm, mobile phase: *n*-hexane/ isopropanol = 91:9, flow rate 1.0 mL/min), er = 66.8:33.2 [tr (major, 66.8%) = 23.0 min, tr (minor, 33.2%) = 34.1 min]. $[\alpha]_D^{25}$= +99.0 (c 0.001, $CHCl_3$).

1-Phenyl-2-(phenylsulfonyl)-3-vinylisoindoline (**2aa**)

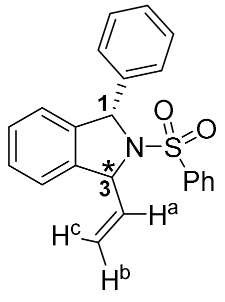

Following the general procedure C, the reaction of (±)-**1aa** (30.0 mg) afforded **2aa** [7.9 mg, 27%, 1:1.2 dr (*cis*/*trans*)] as a colorless oil. R_f = 0.44 [EtOAc:hexanes, 1:8 (v/v)]. δ 7.58 (d, *J* = 7.4 Hz, 2H), 7.45—7.10 (m, 22H), 6.98 (d, *J* = 7.2 Hz, 2H), 6.90 (d, *J* = 7.6 Hz, 2H), 6.12 (d, *J* = 2.6 Hz, 1H, H^1 of *cis*-**2aa**), 6.08 (s, 1H, H^1 of *trans*-**2aa**), 6.05—5.88 (m, 2H, H^a of *cis*- and *trans*-**2aa**), 5.61 (d, *J* = 8.0 Hz, 1H, H^3 of *trans*-**2aa**), 5.50 (d, *J* = 16.9 Hz, 1H, H^c of *trans*-**2aa**), 5.47 (d, *J* = 16.9 Hz, 1H, H^c of *cis*-**2aa**), 5.42 (dd, *J* = 8.6, 2.4 Hz, 1H, H^3 of *cis*-**2aa**), 5.31 (d, *J* = 10.1 Hz, 1H, H^b of *cis*-**2aa**), 5.28 (d, *J* = 10.1 Hz, 1H, H^b of *trans*-**2aa**). ^{13}C{^{1}H} NMR ($CDCl_3$, 101 MHz) δ 141.6, 140.5, 140.4, 140.2, 139.9, 139.7, 139.1, 138.3, 138.0, 137.5, 132.3, 131.7, 128.6, 128.5, 128.4, 128.2, 128.05, 127.99, 127.96, 127.8, 127.5, 127.0, 123.6, 123.35, 123.25, 117.6, 116.1, 70.0, 69.5, 69.1, 68.5. HRMS (ESI) m/z: $[M + Na]^+$ Calcd for $C_{22}H_{19}NNaO_2S$ 384.1029; Found 384.1026. HPLC analysis: Daicel Chiralcel IC (absorbance at 220 nm, mobile phase: *n*-hexane/ isopropanol = 92:8, flow rate 1.0 mL/min), er of *cis*-**2aa** = 90.2:9.8 [tr (major, 40.3%) = 32.2 min, tr (minor, 4.4%) = 34.4 min], er of *trans*-**2aa** = 89.6:10.4 [tr (minor 5.8%) = 19.3 min, tr (major, 49.5%) = 37.7 min]. $[\alpha]_D^{25}$= +101.3 (c 0.001, CH_3OH).

(R,E)-4-(*tert*-Butyl)-*N*-{[2-(3-hydroxyprop-1-en-1-yl)phenyl](phenyl)methyl}benzenesulfonamide [(R)-**1ab**]

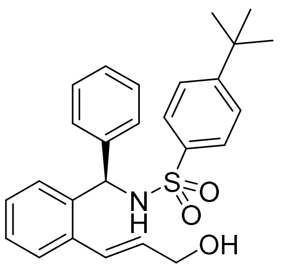

Following the general procedure C, the reaction of (±)-**1ab** (30.0 mg) resulted in the recovery of (R)-**1ab** (12.3 mg, 41%) as a yellow solid. R_f = 0.71 [EtOAc:hexanes, 1:1 (v/v)]. Mp 65−66 °C. ^{1}H NMR ($CDCl_3$, 400 MHz) δ 7.49 (d, J = 8.3 Hz, 2H), 7.32 (d, J = 7.5 Hz, 1H), 7.26−7.23 (m, 3H), 7.18−7.14 (m, 3H), 7.08−7.02 (m, 3H), 6.89 (d, J = 7.6 Hz, 1H), 6.80 (d, J = 15.6 Hz, 1H), 6.11 (dt, J = 15.6, 5.3 Hz, 1H), 5.95 (d, J = 7.6 Hz, 1H), 5.21 (d, J = 7.6 Hz, 1H), 4.26 (s, 2H), 1.93 (s, 1H), 1.28 (s, 9H). ^{13}C{^{1}H} NMR ($CDCl_3$, 101 MHz) δ 156.1, 139.4, 137.0, 136.9, 136.0, 132.6, 128.4, 128.0, 127.8, 127.54, 127.46, 127.4, 127.2, 126.8, 125.6, 63.5, 58.4, 35.0, 31.0. HRMS (ESI) m/z: $[M + Na]^+$ Calcd for $C_{26}H_{29}NNaO_3S$ 458.1760; Found 458.1757. HPLC analysis: Daicel Chiralcel IC (absorbance at 254 nm, mobile phase: *n*-hexane/ isopropanol = 75:25, flow rate 1.0 mL/min), er = 94.2:5.8 [tr (major, 94.2%) = 10.8 min, tr (minor, 5.8%) = 13.9 min]. $[\alpha]_D^{25}$= +24.8 (c 0.001, $CHCl_3$).

2-{[4-(*tert*-Butyl)phenyl]sulfonyl}-1-phenyl-3-vinylisoindoline (**2ab**)

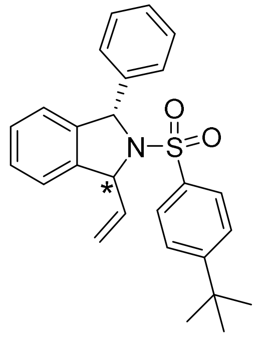

Following the general procedure C, the reaction of (±)-**1ab** (30.0 mg) afforded **2ab** [6.3 mg, 22%, 1:2.0 dr (*cis*/*trans*)] as a yellow oil. R_f= 0.49 [EtOAc:hexanes, 1:8 (v/v)]. ^{1}H NMR ($CDCl_3$, 400 MHz) δ 7.53 (d, J = 8.5 Hz, 2H), 7.32−7.14 (m, 20H), 7.07 (t, J = 7.7 Hz, 1H), 6.95 (d, J = 7.3 Hz, 1H), 6.89 (d, J = 7.2 Hz, 1H), 6.88 (d, J = 5.8 Hz, 1H), 6.09 (d, J = 2.4 Hz, 1H), 6.02 (s, 1H), 6.05−5.95 (m, 2H), 5.59 (d, J = 8.0 Hz, 1H), 5.50 (d, J = 17.0 Hz, 1H), 5.49−5.45 (m, 2H), 5.31 (d, J = 9.9 Hz, 1H), 5.30 (d, J = 9.9 Hz, 1H), 1.28 (s, 9H), 1.27 (s, 9H). ^{13}C{^{1}H} NMR ($CDCl_3$, 101 MHz) δ 156.1, 155.3, 141.8, 140.8, 140.4, 140.1, 139.3, 138.6, 138.1, 137.6, 137.4, 136.4, 128.6, 128.4, 128.1, 128.0, 127.9, 127.8, 127.7, 127.4, 126.9, 125.6, 125.2, 123.6, 123.3, 117.4, 115.9, 69.8, 69.5, 69.2, 68.4, 35.0, 34.9, 31.0. HRMS (ESI) m/z: $[M + H]^+$ Calcd for $C_{26}H_{28}NO_2S$ 418.1835; Found 418.1836. HPLC analysis: Daicel Chiralcel AD−H (absorbance at 220 nm, mobile phase: *n*-hexane/ isopropanol = 98:2, flow rate 1.0 mL/min), er of *cis*-**2ab** = 84.5:15.5 [tr (minor, 5.2%) = 27.0 min, tr (major, 28.4%) = 28.9 min], er of *trans*-**2ab** = 84.2:15.8 [tr (minor, 10.5%) = 46.4 min, tr (major, 55.9%) = 55.9 min]. $[\alpha]_D^{25}$= +262.3 (c 0.001, $CHCl_3$).

(*R*,*E*)-4-Fluoro-*N*-{[2-(3-hydroxyprop-1-en-1-yl)phenyl](phenyl)methyl}benzenesulfonamide [(*R*)-**1ac**]

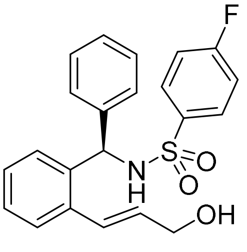

Following the general procedure C, the reaction of (±)-**1ac** (30.0 mg) resulted in the recovery of (*R*)-**1ac** (14.0 mg, 47%) as a yellow solid. R_f = 0.62 [EtOAc:hexanes, 1:1 (v/v)]. Mp 99−100 ºC. ^{1}H NMR ($CDCl_3$, 400 MHz) δ 7.59−7.55 (m, 2H), 7.35 (d, *J* = 7.8 Hz, 1H), 7.22−7.19 (m, 4H), 7.11−7.06 (m, 3H), 6.95−6.90 (m, 3H), 6.81 (d, *J* = 15.6 Hz, 1H), 6.13 (dt, *J* = 15.6, 5.4 Hz, 1H), 6.01 (d, *J* = 7.5 Hz, 1H), 5.24 (d, *J* = 7.5 Hz, 1H), 4.27 (dd, *J* = 5.4, 1.6 Hz, 2H). ^{13}C{^{1}H} NMR ($CDCl_3$, 101 MHz) δ 164.8 (d, $J_{C–F}$ = 254.5 Hz), 139.2, 136.6, 136.40, 136.36, 136.0, 132.7, 129.7 (d, $J_{C–F}$ = 9.4 Hz), 128.6, 128.2, 128.0, 127.7 (d, $J_{C–F}$ = 8.5 Hz), 127.6, 127.4, 127.3, 115.7 (d, $J_{C–F}$ = 22.6 Hz), 63.4, 58.5. ^{19}F NMR ($CDCl_3$, 376 MHz) δ –105.61 to –105.65 (m). HRMS (ESI) m/z: $[M + H]^+$ Calcd for $C_{22}H_{21}FNO_3S$ 398.1221; Found 398.1215. HPLC analysis: Daicel Chiralcel OD−H (absorbance at 254 nm, mobile phase: *n*-hexane/ isopropanol = 85:15, flow rate 1.0 mL/min), er = 69.4:30.6 [tr (major, 69.4%) = 11.5 min, tr (minor, 30.6%) = 19.2 min]. $[\alpha]_D^{25}$= +157.1 (c 0.001, $CHCl_3$).

2-[(4-Fluorophenyl)sulfonyl]-1-phenyl-3-vinylisoindoline (**2ac**)

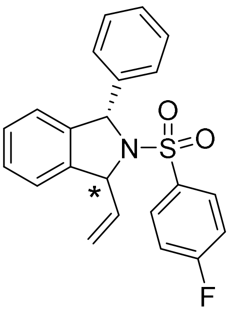

Following the general procedure C, the reaction of (±)-**1ac** (30.0 mg) afforded **2ac** [6.6 mg, 23%, 1:1.2 dr (*cis*/*trans*)] as a colorless oil. R_f = 0.47 [EtOAc:hexanes, 1:8 (v/v)]. ^{1}H NMR ($CDCl_3$, 400 MHz) δ 7.50−7.47 (m, 2H), 7.31−7.12 (m, 16H), 6.97−6.84 (m, 8H), 6.11 (s, 2H), 6.06−5.97 (m, 1H), 5.89−5.81 (m, 1H), 5.65 (d, *J* = 8.2 Hz, 1H), 5.51 (d, *J* = 17.0 Hz, 1H), 5.45 (d, *J* = 17.0 Hz, 1H), 5.38 (dd, *J* = 8.8, 2.0 Hz, 1H), 5.31 (d, *J* = 10.5 Hz, 1H), 5.28 (d, *J* = 10.2 Hz, 1H). ^{13}C{^{1}H} NMR ($CDCl_3$, 101 MHz) δ 164.7 (d, $J_{C–F}$ = 254.2 Hz), 164.5 (d, $J_{C–F}$ = 253.8 Hz), 141.4, 140.4, 140.2, 139.8, 139.1, 138.2, 138.0, 137.5, 136.4, 130.1 (d, $J_{C–F}$ = 9.3 Hz), 129.7 (d, $J_{C–F}$ = 9.3 Hz), 128.7, 128.6, 128.5, 128.3, 128.2, 128.1, 128.0, 123.6, 123.39, 123.35, 123.3, 118.0, 116.1, 115.5 (d, $J_{C–F}$ = 22.2 Hz), 115.3 (d, $J_{C–F}$ = 20.7 Hz), 70.0, 69.6, 69.1, 68.6. ^{19}F NMR ($CDCl_3$, 376 MHz) δ –105.99 to –106.02 (m), –106.61 to –106.63 (m). HRMS (ESI) m/z: $[M + Na]^+$ Calcd for $C_{22}H_{18}FNNaO_2S$ 402.0934; Found 402.0933. HPLC analysis: Daicel Chiralcel OD−H (absorbance at 220 nm, mobile phase: *n*-hexane/ isopropanol = 98:2, flow rate 1.0 mL/min), er of *cis*-**2ac** = 91.1:8.9 [tr (minor, 4.2%) = 13.1 min, tr (major, 43.0%) = 17.1 min], er of *trans*-**2ac** = 90.8:9.2 [tr (minor, 5.0%) = 11.7 min, tr (major, 47.9%) = 14.9 min]. $[\alpha]_D^{25}$= +227.3 (c 0.001, CH_3OH).

(R,E)-N-{[2-(3-Hydroxyprop-1-en-1-yl)phenyl](phenyl)methyl}-4-(trifluoromethyl) benzenesulfonamide [(R)-**1ad**]

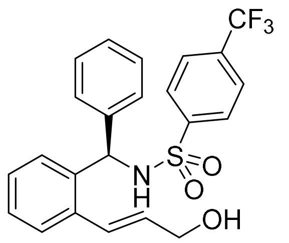

Following the general procedure C, the reaction of (±)-**1ad** (30.0 mg) resulted in the recovery of (R)-**1ad** (9.3 mg, 31%) as a yellow solid. R_f = 0.71 [EtOAc:hexanes, 1:1 (v/v)]. Mp 147−148 °C. ^{1}H NMR ($CDCl_3$, 400 MHz) δ 7.63 (d, J = 7.8 Hz, 2H), 7.46−7.44 (m, 2H), 7.31−7.30 (m, 1H), 7.20−7.14 (m, 4H), 7.10−7.08 (m, 2H), 7.03 (t, J = 7.6 Hz, 1H), 6.87 (d, J = 7.8 Hz, 1H), 6.79 (d, J = 15.5 Hz, 1H), 6.08 (dt, J = 15.5, 4.3 Hz, 1H), 6.00 (d, J = 8.1 Hz, 1H), 5.83−5.69 (m, 1H), 4.26 (s, 2H), 2.01 (s, 1H). ^{13}C{^{1}H} NMR ($CDCl_3$, 101 MHz) δ 143.7, 138.8, 136.2, 136.0, 133.78 (q, J_{C-F} = 32.9 Hz), 132.8, 128.6, 128.3, 128.2, 127.7, 127.6, 127.4, 127.33, 127.29, 125.6 (q, J_{C-F} = 3.6 Hz), 123.1 (q, J_{C-F} = 272.8 Hz), 63.3, 58.7. ^{19}F NMR ($CDCl_3$, 376 MHz) δ –63.23 (s). HRMS (ESI) m/z: [M + Na]$^+$ Calcd for $C_{23}H_{20}F_3NNaO_3S$ 470.1008; Found 470.1003. HPLC analysis: Daicel Chiralcel IC (absorbance at 254 nm, mobile phase: n-hexane/ isopropanol = 90:10, flow rate 1.0 mL/min), er = 88.4:11.6 [tr (major, 88.4%) = 13.9 min, tr (minor, 11.6%) = 18.8 min]. $[\alpha]_D^{25}$= +21.3 (c 0.001, $CHCl_3$).

1-Phenyl-2-{[4-(trifluoromethyl)phenyl]sulfonyl}-3-vinylisoindoline (**2ad**)

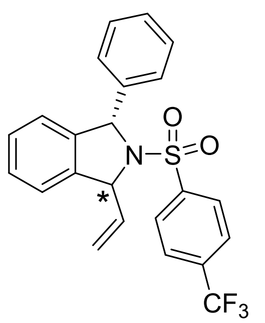

Following the general procedure C, the reaction of (±)-**1ad** (30.0 mg) afforded **2ad** [7.1 mg, 25%, 1.2:1 dr (*cis*/*trans*)] as a yellow oil. R_f = 0.53 [EtOAc:hexanes, 1:8 (v/v)]. ^{1}H NMR ($CDCl_3$, 400 MHz) δ 7.56−7.45 (m, 6H), 7.38−7.30 (m, 4H), 7.28−7.15 (m, 10H), 7.11 (t, J = 7.8 Hz, 2H), 6.95−6.91 (m, 4H), 6.18 (s, 1H), 6.15 (d, J = 2.6 Hz, 1H), 6.12−6.03 (m, 1H), 5.86−5.79 (m, 1H), 5.74 (d, J = 8.4 Hz, 1H), 5.58 (d, J = 16.6 Hz, 1H), 5.50 (d, J = 17.0 Hz, 1H), 5.45 (dd, J = 8.6, 2.4 Hz, 1H), 5.36 (d, J = 10.6 Hz, 1H), 5.34 (d, J = 10.7 Hz, 1H). ^{13}C{^{1}H} NMR ($CDCl_3$, 101 MHz) δ 144.0, 143.7, 140.9, 140.2, 139.8, 139.6, 139.0, 137.8, 137.7, 137.3, 133.34 (q, J = 33.6 Hz, 1H), 133.29 (q, J = 32.9 Hz, 1H), 128.82, 128.77, 128.7, 128.5, 128.30, 128.27, 128.2, 128.1, 127.8, 127.4, 125.4 (q, J = 3.7 Hz, 1H), 125.3 (q, J = 3.6 Hz, 1H), 123.6, 123.39, 123.35, 123.27, 123.25 (q, J = 272.7 Hz, 1H), 123.2 (q, J = 272.9 Hz, 1H), 118.6, 116.2, 70.0, 69.6, 69.4, 68.7. ^{19}F NMR ($CDCl_3$, 376 MHz) δ –63.17 (s). HRMS (ESI) m/z: [M + H]$^+$ Calcd for $C_{23}H_{19}F_3NO_2S$ 430.1083; Found 430.1083. HPLC analysis: Daicel Chiralcel OD−H (absorbance at 220 nm, mobile phase: n-hexane/ isopropanol = 97:3, flow rate 1.0 mL/min), er of *cis*-**2ad** = 89.1:10.9 [tr (minor, 8.1%) = 8.4 min, tr (major, 66.3%) = 11.1 min], er of *trans*-**2ad** = 87.3:12.7 [tr (minor, 3.3%) = 7.7 min, tr (major, 22.3%) = 9.7 min]. $[\alpha]_D^{25}$= +108.7 (c 0.001, $CHCl_3$).

(*R*,*E*)-*N*-{[2-(3-Hydroxyprop-1-en-1-yl)phenyl](phenyl)methyl}-2-(trifluoromethyl)benzenesulfonamide [(*R*)-**1ae**]

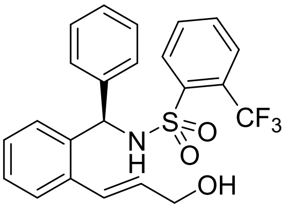

Following the general procedure C, the reaction of (±)-**1ae** (30.0 mg) resulted in the recovery of (*R*)-**1ae** (12.8 mg, 64%) as a yellow oil. R_f = 0.68 [EtOAc:hexanes, 1:1 (v/v)]. ^{1}H NMR ($CDCl_3$, 400 MHz) δ 7.81 (d, *J* = 7.9 Hz, 1H), 7.70 (d, *J* = 7.7 Hz, 1H), 7.51 (t, *J* = 7.6 Hz, 1H), 7.37 (t, *J* = 7.2 Hz, 1H), 7.33 (d, *J* = 6.8 Hz, 1H), 7.20−7.14 (m, 4H), 7.09−7.06 (m, 2H), 7.04 (td, *J* = 7.6, 1.3 Hz, 1H), 6.87 (dd, *J* = 7.8, 0.6 Hz, 1H), 6.80 (d, *J* = 15.6 Hz, 1H), 6.12 (dt, *J* = 15.6, 5.4 Hz, 1H), 6.04 (d, *J* = 8.2 Hz, 1H), 5.40 (d, *J* = 8.2 Hz, 1H), 4.26 (dd, *J* = 5.4, 1.6 Hz, 2H). $^{13}C\{^1H\}$ NMR ($CDCl_3$, 101 MHz) δ 139.0, 136.2, 136.1, 132.8, 132.3, 132.0, 131.3, 128.5, 128.2, 127.9, 127.8, 127.7, 127.53, 127.46, 127.30, 127.26, 127.0 (q, $J_{C\text{–}F}$ = 32.8 Hz), 122.9 (q, $J_{C\text{–}F}$ = 273.7 Hz), 63.4, 58.7. ^{19}F NMR ($CDCl_3$, 376 MHz) δ –57.98 (s). HRMS (ESI) m/z: $[M + Na]^+$ Calcd for $C_{23}H_{20}F_3NNaO_3S$ 470.1008; Found 470.1010. HPLC analysis: Daicel Chiralcel IC (absorbance at 254 nm, mobile phase: *n*-hexane/ isopropanol = 88:12, flow rate 1.0 mL/min), er = 60.8:39.2 [tr (major, 60.8%) = 18.6 min, tr (minor, 39.2%) = 20.3 min]. $[\alpha]_D^{25}$= +46.9 (c 0.001, $CHCl_3$).

1-Phenyl-2-{[2-(trifluoromethyl)phenyl]sulfonyl}-3-vinylisoindoline (**2ae**)

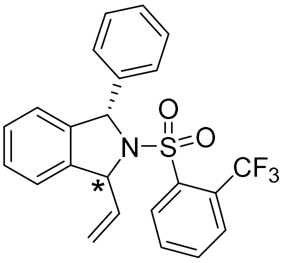

Following the general procedure C, the reaction of (±)-**1ae** (30.0 mg) afforded **2ae** [3.1 mg, 16%, 1:1.2 dr (*cis*/*trans*)] as a yellow oil. R_f = 0.54 [EtOAc:hexanes, 1:8 (v/v)]. ^{1}H NMR ($CDCl_3$, 400 MHz) δ 7.75 (d, *J* = 8.0 Hz, 1H), 7.65 (d, *J* = 7.9 Hz, 3H), 7.51 (t, *J* = 7.5 Hz, 1H), 7.42−7.28 (m, 6H), 7.22−7.21 (m, 8H), 7.05−6.98 (m, 6H), 6.79 (d, *J* = 7.9 Hz, 1H), 6.22 (s, 1H), 6.10 (d, *J* = 1.7 Hz, 1H), 6.06−5.95 (m, 2H), 5.82 (dd, *J* = 8.2, 2.0 Hz, 1H), 5.72 (d, *J* = 8.0 Hz, 1H), 5.40 (d, *J* = 17.0 Hz, 1H), 5.38 (d, *J* = 17.0 Hz, 1H), 5.22 (d, *J* = 10.0 Hz, 1H), 5.15 (d, *J* = 10.0 Hz, 1H). $^{13}C\{^1H\}$ NMR ($CDCl_3$, 101 MHz) δ 141.2, 140.8, 140.3, 139.8, 139.5, 139.0, 137.9, 137.8, 137.5, 132.5, 132.2, 131.8, 131.7, 131.4, 130.7, 128.7, 128.44, 128.37, 128.3, 128.2, 128.13, 128.06, 128.0, 127.9, 127.5 (q, $J_{C\text{–}F}$ = 6.3 Hz), 127.0 (q, $J_{C\text{–}F}$ = 33.0 Hz), 123.7, 123.5, 123.4, 123.1, 122.7 (q, $J_{C\text{–}F}$ = 274.1 Hz), 122.5 (q, $J_{C\text{–}F}$ = 274.5 Hz), 118.0, 116.8, 70.0, 69.7, 69.4, 68.6. ^{19}F NMR ($CDCl_3$, 376 MHz) δ –57.15 (s), –57.89 (s). HRMS (ESI) m/z: $[M + H]^+$ Calcd for $C_{23}H_{19}F_3NO_2S$ 430.1083; Found 430.1084. HPLC analysis: Daicel Chiralcel AD−H (absorbance at 220 nm, mobile phase: *n*-hexane/ isopropanol = 95:5, flow rate 1.0 mL/min), er of *cis*-**2ae** = 80.1:19.9 [tr (minor, 9.0%) = 10.2 min, tr (major, 36.2%) = 11.6 min], er of *trans*-**2ae** = 79.3:20.7 [tr (major, 43.5%) = 22.0 min, tr (minor, 11.3%) = 41.1 min]. $[\alpha]_D^{25}$= +129.0 (c 0.001, $CHCl_3$).

(R,E)-N-{[2-(3-Hydroxyprop-1-en-1-yl)phenyl](phenyl)methyl}-2-methylbenzenesulfonamide [(R)-**1af**]

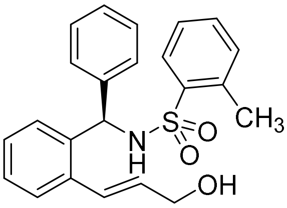

Following the general procedure C, the reaction of (±)-**1af** (30.0 mg) resulted in the recovery of (R)-**1af** (20.1 mg, 67%) as a yellow solid. R_f = 0.62 [EtOAc:hexanes, 1:1 (v/v)]. Mp 140−141 °C. ^{1}H NMR ($CDCl_3$, 400 MHz) δ 7.77 (d, J = 7.8 Hz, 1H), 7.36−7.33 (m, 2H), 7.21−7.06 (m, 9H), 6.69 (d, J = 7.6 Hz, 1H), 6.68 (d, J = 15.6 Hz, 1H), 6.10 (dt, J = 15.6, 5.4 Hz, 1H), 5.85 (d, J = 7.0 Hz, 1H), 5.16 (d, J = 7.0 Hz, 1H), 4.21 (s, 2H), 2.44 (s, 3H), 1.73 (s, 1H). ^{13}C{^{1}H} NMR ($CDCl_3$, 101 MHz) δ 139.9, 138.1,137.2, 136.9, 135.7, 132.6, 132.2, 129.5, 128.5, 128.1, 127.8, 127.69, 127.66, 127.6, 127.2, 126.0, 63.4, 58.3, 20.2. HRMS (ESI) m/z: $[M + Na]^+$ Calcd for $C_{23}H_{23}NNaO_3S$ 416.1291; Found 416.1292. HPLC analysis: Daicel Chiralcel OD−H (absorbance at 254 nm, mobile phase: n-hexane/ isopropanol = 91:9, flow rate 1.0 mL/min), er = 62.5:37.5 [tr (major, 62.5%) = 21.2 min, tr (minor, 37.5%) = 24.9 min]. $[\alpha]_D^{25}$= +69.3 (c 0.001, $CHCl_3$)

1-Phenyl-2-(o-tolylsulfonyl)-3-vinylisoindoline (**2af**)

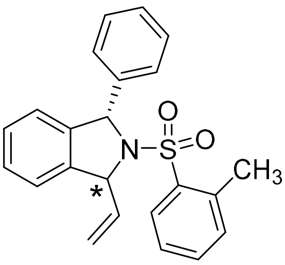

Following the general procedure C, the reaction of (±)-**1af** (30.0 mg) afforded **2af** [4.9 mg, 17%, 1:2.0 dr (*cis*/*trans*)] as a yellow oil. R_f = 0.47 [EtOAc:hexanes, 1:8 (v/v)]. ^{1}H NMR ($CDCl_3$, 400 MHz) δ 7.85 (d, J = 7.8 Hz, 1H), 7.51 (d, J = 7.9 Hz, 1H), 7.34−7.04 (m, 21H), 6.95 (t, J = 7.6 Hz, 1H), 6.91 (d, J = 7.5 Hz, 1H), 6.82 (d, J = 7.4 Hz, 1H), 6.12 (s, 1H), 6.05 (d, J = 2.4 Hz, 1H), 5.97−5.86 (m, 2H), 5.76 (dd, J = 8.4, 2.4 Hz, 1H), 5.68 (d, J = 8.0 Hz, 1H), 5.36 (d, J = 16.9 Hz, 1H), 5.24 (d, J = 16.9 Hz, 1H), 5.12 (d, J = 10.0 Hz, 1H), 5.11 (d, J = 10.2 Hz, 1H), 2.57 (s, 3H), 2.36 (s, 3H). ^{13}C{^{1}H} NMR ($CDCl_3$, 101 MHz) δ 141.6, 141.08, 140.13, 139.6, 138.9, 138.2, 138.0, 137.8, 137.7, 137.3, 136.9, 133.0, 132.4, 132.1, 131.7, 130.1, 130.0, 128.5, 128.4, 128.2, 128.1, 128.0, 127.9, 127.6, 125.7, 125.5, 123.7, 123.5, 123.4, 123.2, 117.2, 116.7, 69.5, 69.3, 69.2, 68.0, 20.3, 20.0. (ESI) m/z: $[M + H]^+$ Calcd for $C_{23}H_{22}NO_2S$ 376.1366; Found 376.1364. HPLC analysis: Daicel Chiralcel AD−H (absorbance at 220 nm, mobile phase: n-hexane/ isopropanol = 95:5, flow rate 1.0 mL/min), er of *cis*-**2af** = 93.2:6.8 [tr (minor, 2.7%) = 12.5 min, tr (major, 37.1%) = 16.3 min], er of *trans*-**2af** = 92.6:7.4 [tr (major, 55.8%) = 23.4 min, tr (minor, 4.6%) = 33.2 min]. $[\alpha]_D^{25}$= +81.6 (c 0.001, $CHCl_3$).

(*R*,*E*)-*N*-{[2-(3-Hydroxyprop-1-en-1-yl)phenyl](phenyl)methyl}-3-(trifluoromethyl)benzenesulfonamide [(*R*)-**1ah**]

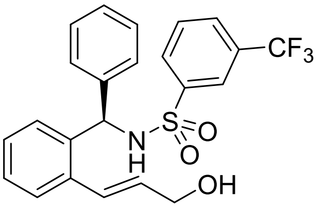

Following the general procedure C, the reaction of (±)-**1ah** (30.0 mg) resulted in the recovery of (*R*)-**1ah** (16.8 mg, 56%) as a yellow oil. R_f = 0.63 [EtOAc:hexanes, 1:1 (v/v)]. ^{1}H NMR ($CDCl_3$, 400 MHz) δ 7.77−7.74 (m, 2H), 7.62 (d, *J* = 7.6 Hz, 1H), 7.39 (t, *J* = 7.6 Hz, 1H), 7.32 (d, *J* = 7.5 Hz, 1H), 7.20−7.15 (m, 4H), 7.11−7.09 (m, 2H), 7.04 (d, *J* = 7.4 Hz, 1H), 6.88 (d, *J* = 7.7 Hz, 1H), 6.81 (d, *J* = 15.6 Hz, 1H), 6.12 (dt, *J* = 15.6, 5.1 Hz, 1H), 6.02 (d, *J* = 7.0 Hz, 1H), 5.41 (d, *J* = 7.0 Hz, 1H), 4.28 (s, 2H), 1.92 (s, 1H). ^{13}C{^{1}H} NMR ($CDCl_3$, 101 MHz) δ 141.5, 138.8, 136.0, 135.9, 132.6, 130.9 (q, $J_{C\text{–}F}$ = 33.3 Hz), 130.0, 129.3, 128.7 (q, $J_{C\text{–}F}$ = 3.2 Hz), 128.5, 128.24, 128.17, 127.6, 127.5, 127.3, 127.2, 123.9 (q, $J_{C\text{–}F}$ = 3.8 Hz), 123.0 (q, $J_{C\text{–}F}$ = 273.0 Hz), 63.2, 58.8. ^{19}F NMR ($CDCl_3$, 376 MHz) δ –62.78 (s). HRMS (ESI) m/z: $[M + Na]^+$ Calcd for $C_{23}H_{20}F_3NNaO_3S$ 470.1008; Found 470.1013. HPLC analysis: Daicel Chiralcel OD−H (absorbance at 254 nm, mobile phase: *n*-hexane/ isopropanol = 90:10, flow rate 1.0 mL/min), er = 81.2:18.8 [tr (major, 81.2%) = 12.4 min, tr (minor, 18.8%) = 19.5 min]. $[\alpha]_D^{25}$= +29.8 (c 0.002, $CHCl_3$).

1-Phenyl-2-{[3-(trifluoromethyl)phenyl]sulfonyl}-3-vinylisoindoline (**2ah**)

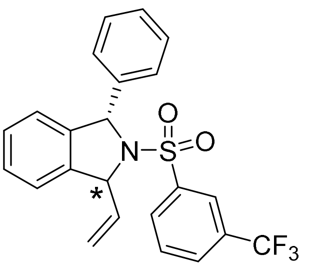

Following the general procedure C, the reaction of (±)-**1ah** (30.0 mg) afforded **2ah** [9.8 mg, 34%, 1.2:1 dr (*cis*/*trans*)] as a colorless oil. R_f = 0.50 [EtOAc:hexanes, 1:8 (v/v)]. ^{1}H NMR ($CDCl_3$, 400 MHz) δ 7.66−7.57 (m, 4H), 7.50−7.45 (m, 2H), 7.38−7.28 (m, 6H), 7.25−7.08 (m, 10H), 6.95−6.89 (m, 4H), 6.20 (s, 1H), 6.13 (d, *J* = 2.6 Hz, 1H), 6.08−5.99 (m, 1H), 5.81−5.71 (m, 2H), 5.60−5.54 (m, 1H), 5.48 (d, *J* = 17.0 Hz, 1H), 5.42 (dd, *J* = 8.6, 2.4 Hz, 1H), 5.35 (d, *J* = 10.0 Hz, 1H), 5.31 (d, *J* = 9.1 Hz, 1H). ^{13}C{^{1}H} NMR ($CDCl_3$, 101 MHz) δ 142.0, 141.3, 140.7, 140.2, 139.7, 139.6, 138.8, 137.7, 137.4, 133.8, 133.6, 130.9 (q, $J_{C\text{–}F}$ = 33.2 Hz), 130.4, 130.1, 129.03, 128.99, 128.8, 128.6, 128.53, 128.5 (q, $J_{C\text{–}F}$ = 2.4 Hz), 128.5 (q, $J_{C\text{–}F}$ = 4.1 Hz), 124.6 (q, $J_{C\text{–}F}$ = 3.8 Hz), 124.2 (q, $J_{C\text{–}F}$ = 3.9 Hz), 123.6, 123.4, 123.3, 123.2, 123.1 (q, $J_{C\text{–}F}$ = 273.5 Hz), 118.8, 116.4, 70.0, 69.6, 69.4, 68.7. ^{19}F NMR ($CDCl_3$, 376 MHz) δ –62.61 (s), –62.81 (s). HRMS (ESI) m/z: $[M + H]^+$ Calcd for $C_{23}H_{19}F_3NO_2S$ 430.1083; Found 430.1084. HPLC analysis: Daicel Chiralcel OD−H (absorbance at 220 nm, mobile phase: *n*-hexane/ isopropanol = 98:2, flow rate 0.8 mL/min), er of *cis*-**2ah** = 92.4:7.6 [tr (minor, 4.0%) = 11.4 min, tr (major, 48.3%) = 15.3 min], er of *trans*-**2ah** = 92.7:7.3 [tr (minor, 3.5%) = 9.9 min, tr (major, 44.2%) = 12.2 min]. $[\alpha]_D^{25}$= +183.1 (c 0.001, $CHCl_3$).

(*R*,*E*)-*N*-{[2-(3-Hydroxyprop-1-en-1-yl)phenyl](4-methoxyphenyl)methyl}-3-(trifluoromethyl)benzenesulfonamide [(*R*)-**1bh**]

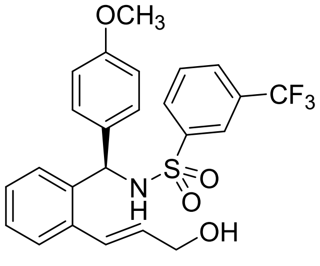

Following the general procedure C, the reaction of (±)-**1bh** (30.0 mg) resulted in the recovery of (*R*)-**1bh** (10.1 mg, 34%) as a yellow oil. R_f = 0.57 [EtOAc:hexanes, 1:1 (v/v)]. ^{1}H NMR ($CDCl_3$, 400 MHz) δ 7.74−7.73 (m, 2H), 7.61 (d, *J* = 7.9 Hz, 1H), 7.38 (t, *J* = 8.0 Hz, 1H), 7.29 (d, *J* = 7.7 Hz, 1H), 7.15 (t, *J* = 7.4 Hz, 1H), 7.05−7.00 (m, 3H), 6.91 (d, *J* = 7.1 Hz, 1H), 6.77 (d, *J* = 15.6 Hz, 1H), 6.71 (d, *J* = 8.7 Hz, 2H), 6.08 (dt, *J* = 15.6, 5.3 Hz, 1H), 5.96 (d, *J* = 7.8 Hz, 1H), 5.71 (s, 1H), 4.26 (d, *J* = 5.3 Hz, 2H), 3.73 (s, 3H). ^{13}C{^{1}H} NMR ($CDCl_3$, 101 MHz) δ 158.9, 141.5, 136.2, 135.9, 132.5, 130.9 (q, $J_{C\text{–}F}$ = 32.7 Hz), 130.8, 130.0, 129.3, 128.7 (q, $J_{C\text{–}F}$ = 3.2 Hz), 128.6, 128.1, 128.0, 127.5, 127.4, 127.2, 123.9 (q, $J_{C\text{–}F}$ = 3.6 Hz), 123.0 (q, $J_{C\text{–}F}$ = 272.8 Hz), 113.8, 63.2, 58.3, 55.1. ^{19}F NMR ($CDCl_3$, 376 MHz) δ –62.78 (s). HRMS (ESI) m/z: $[M + Na]^+$ Calcd for $C_{24}H_{22}F_3NNaO_4S$ 500.1114; Found 500.1114. HPLC analysis: Daicel Chiralcel AD−H (absorbance at 254 nm, mobile phase: *n*-hexane/ isopropanol = 88:12, flow rate 1.0 mL/min), er = 99.2:0.8 [tr (minor, 0.8%) = 18.6 min, tr (major, 99.2%) = 22.0 min]. $[\alpha]_D^{25}$= +207.9 (c 0.001, $CHCl_3$).

1-(4-Methoxyphenyl)-2-{[3-(trifluoromethyl)phenyl]sulfonyl}-3-vinylisoindoline (**2bh**)

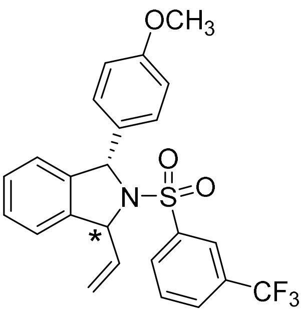

Following the general procedure C, the reaction of (±)-**1bh** (30.0 mg) afforded **2bh** [12.1 mg, 42%, 1:1.4 dr (*cis*/*trans*)] as a colorless oil. R_f = 0.51 [EtOAc:hexanes, 1:8 (v/v)]. ^{1}H NMR ($CDCl_3$, 400 MHz) δ 7.65−7.28 (m, 10H), 7.24−7.15 (m, 4H), 7.04 (d, *J* = 8.6 Hz, 2H), 6.89 (t, *J* = 6.9 Hz, 2H), 6.84 (d, *J* = 8.5 Hz, 2H), 6.73 (d, *J* = 8.6 Hz, 2H), 6.60 (d, *J* = 8.6 Hz, 2H), 6.18 (s, 1H), 6.10 (d, *J* = 2.0 Hz, 1H), 6.08−6.01 (m, 1H), 5.76−5.67 (m, 2H), 5.59−5.53 (m, 1H), 5.46 (d, *J* = 17.0 Hz, 1H), 5.39 (dd, *J* = 8.5, 2.3 Hz, 1H), 5.34 (d, *J* = 10.0 Hz, 1H), 5.28 (d, *J* = 8.5 Hz, 1H), 3.77 (s, 3H), 3.75 (s, 3H). ^{13}C{^{1}H} NMR ($CDCl_3$, 101 MHz) δ 159.49, 159.47, 142.3, 141.5, 140.4, 139.8, 139.0, 137.8, 137.7, 137.4, 132.8, 131.5, 130.80 (q, $J_{C\text{–}F}$ = 32.9 Hz), 130.78 (q, $J_{C\text{–}F}$ = 33.3 Hz), 130.4, 130.1, 130.0, 129.6, 129.0, 128.8, 128.62, 128.58 (q, $J_{C\text{–}F}$ = 3.4 Hz), 128.4 (q, $J_{C\text{–}F}$ = 3.3 Hz), 128.2, 128.1, 124.5 (q, $J_{C\text{–}F}$ = 3.2 Hz), 124.1 (q, $J_{C\text{–}F}$ = 3.9 Hz), 123.6, 123.4, 123.3, 123.2, 123.1 (q, $J_{C\text{–}F}$ = 272.8 Hz), 118.7, 116.2, 113.8, 113.5, 69.4, 69.1, 68.6, 55.2, 55.0. ^{19}F NMR ($CDCl_3$, 376 MHz) δ –62.81 (s), –62.88 (s). HRMS (ESI) m/z: $[M + H]^+$ Calcd for $C_{24}H_{21}F_3NO_3S$ 460.1189; Found 460.1186. HPLC analysis: Daicel Chiralcel AD−H (absorbance at 220 nm, mobile phase: *n*-hexane/ isopropanol = 95:5, flow rate 1.0 mL/min), er of *cis*-**2bh** = 84.9:15.1 [tr (minor, 6.1%) = 11.9 min, tr (major, 34.2%) = 22.5 min], er of *trans*-**2bh** = 84.3:15.7 [tr (major, 50.3%) = 18.4 min, tr (minor, 9.3%) = 25.2 min]. $[\alpha]_D^{25}$= +181.8 (c 0.001, $CHCl_3$).

(R,E)-N-{(4-Fluorophenyl)[2-(3-hydroxyprop-1-en-1-yl)]methyl}-3-(trifluoromethyl)benzenesulfonamide [(R)-**1ch**]

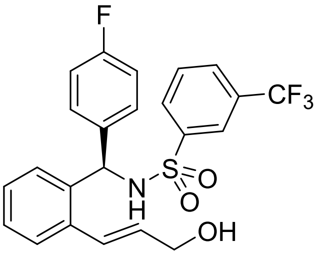

Following the general procedure C, the reaction of (±)-**1ch** (30.0 mg) resulted in the recovery of (R)-**1ch** (8.2 mg, 27%) as a yellow oil. R_f = 0.66 [EtOAc:hexanes, 1:1 (v/v)]. ^{1}H NMR ($CDCl_3$, 400 MHz) δ 7.75 (s, 1H), 7.74 (d, J = 8.5 Hz, 1H), 7.64 (d, J = 7.8 Hz, 1H), 7.40 (t, J = 7.8 Hz, 1H), 7.31 (d, J = 7.6 Hz, 1H), 7.17 (t, J = 7.5 Hz, 1H), 7.11−7.03 (m, 3H), 6.91−6.84 (m, 2H), 6.85 (d, J = 7.7 Hz, 1H), 6.75 (d, J = 15.7 Hz, 1H), 6.09 (dt, J = 15.7, 5.1 Hz, 1H), 5.99 (d, J = 7.9 Hz, 1H), 5.61 (s, 1H), 4.28 (d, J = 5.1 Hz, 2H). ^{13}C{^{1}H} NMR ($CDCl_3$, 101 MHz) δ 162.1 (d, J_{C-F} = 247.4 Hz), 141.4, 136.0, 135.8, 134.63, 134.60, 133.0, 131.1 (q, J_{C-F} = 33.4 Hz), 130.0, 129.4, 129.1 (d, J_{C-F} = 8.1 Hz), 128.9 (q, J_{C-F} = 3.2 Hz), 128.4, 128.0, 127.7, 127.5, 127.1, 124.0 (q, J_{C-F} = 3.7 Hz), 123.0 (q, J_{C-F} = 272.9 Hz), 115.5 (d, J_{C-F} = 21.7 Hz), 63.2, 58.2. ^{19}F NMR ($CDCl_3$, 376 MHz) δ –62.84 (s), –113.04 to –113.12 (m). HRMS (ESI) m/z: [M + Na]$^+$ Calcd for $C_{23}H_{19}F_4NNaO_3S$ 488.0914; Found 488.0911. HPLC analysis: Daicel Chiralcel AD−H (absorbance at 254 nm, mobile phase: n-hexane/ isopropanol = 88:12, flow rate 1.0 mL/min), er = 98.4:1.6 [tr (minor, 1.6%) = 13.3 min, tr (major, 98.4%) = 14.5 min]. $[\alpha]_D^{25}$= +12.2 (c 0.001, $CHCl_3$).

1-(4-Fluorophenyl)-2-{[3-(trifluoromethyl)phenyl]sulfonyl}-3-vinylisoindoline (**2ch**)

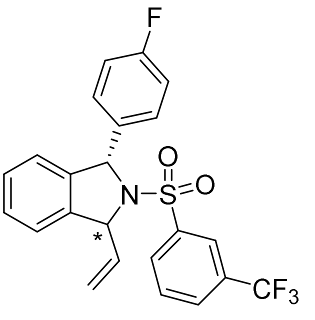

Following the general procedure C, the reaction of (±)-**1ch** (30.0 mg) afforded **2ch** [11.9 mg, 41%, 1.2:1 dr (cis/$trans$)] as a colorless oil. R_f = 0.59 [EtOAc:hexanes, 1:8 (v/v)]. ^{1}H NMR ($CDCl_3$, 400 MHz) δ 7.65−7.63 (m, 3H), 7.64 (d, J = 8.8 Hz, 1H), 7.55−7.51 (m, 2H), 7.44−7.39 (m, 2H), 7.33−7.29 (m, 2H), 7.25−7.22 (m, 2H), 7.17 (d, J = 7.6 Hz, 2H), 7.14−7.10 (m, 2H), 6.95−6.87 (m, 6H), 6.80 (t, J = 8.5 Hz, 2H), 6.18 (s, 1H), 6.12 (d, J = 2.6 Hz, 1H), 6.06−5.97 (m, 1H), 5.78−5.69 (m, 2H), 5.60−5.53 (m, 1H), 5.48 (d, J = 17.0 Hz, 1H), 5.41 (dd, J = 8.3, 2.2 Hz, 1H), 5.34 (d, J = 9.9 Hz, 1H), 5.31 (d, J = 9.0 Hz, 1H). ^{13}C{^{1}H} NMR ($CDCl_3$, 101 MHz) δ 162.51 (d, J_{C-F} = 247.6 Hz), 162.48 (d, J_{C-F} = 248.3 Hz), 141.9, 141.4, 139.9, 139.3, 138.6, 137.7, 137.6, 137.4, 136.70, 136.67, 135.7, 135.6, 131.0 (q, J_{C-F} = 33.3 Hz), 130.43, 130.35, 130.1, 130.0, 129.1, 128.9, 128.8 (q, J_{C-F} = 3.6 Hz), 128.7, 128.6 (q, J_{C-F} = 3.3 Hz), 128.43, 128.37, 124.5 (q, J_{C-F} = 3.9 Hz), 124.1 (q, J_{C-F} = 3.9 Hz), 123.5, 123.42, 123.36, 123.3, 123.1 (q, J_{C-F} = 273.3 Hz), 118.9, 116.6, 115.5 (d, J_{C-F} = 21.3 Hz), 115.3 (d, J_{C-F} = 21.3 Hz), 69.3, 69.1, 68.8, 68.6. ^{19}F NMR ($CDCl_3$, 376 MHz) δ –62.88 (s), –62.97 (s), –113.14 to –113.15 (m), –113.58 to –113.64 (m). HRMS (ESI) m/z: [M + H]$^+$ Calcd for $C_{23}H_{18}F_4NO_2S$ 448.0989; Found 448.0990. HPLC analysis: Daicel Chiralcel OD−H (absorbance at 220 nm, mobile phase: n-hexane/ isopropanol = 99:1, flow rate 1.0 mL/min), er of cis-**2ch** = 81.6:18.4 [tr (major, 28.8%) = 13.2 min, tr (minor, 12.4%) = 14.7 min], er of $trans$-**2ch** = 69.6:30.1 [tr (major, 47.9%) = 11.6 min, tr (minor, 10.8%) = 12.4 min]. $[\alpha]_D^{25}$= +184.9 (c 0.001, $CHCl_3$).

(*R*,*E*)-*N*-{[2-(3-Hydroxyprop-1-en-1-yl)](4-(trifluoromethyl)methyl}-3-(trifluoromethyl)benzenesulfonamide [(*R*)-**1dh**]

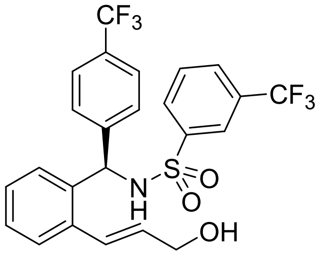

Following the general procedure C, the reaction of (±)-**1dh** (30.0 mg) resulted in the recovery of (*R*)-**1dh** (14.1 mg, 47%) as a colorless oil. R_f = 0.75 [EtOAc:hexanes, 1:1 (v/v)]. ^{1}H NMR ($CDCl_3$, 400 MHz) δ 7.77 − 7.74 (m, 2H), 7.64 (d, *J* = 7.7 Hz, 1H), 7.46 (d, *J* = 8.1 Hz, 2H), 7.40 (t, *J* = 7.8 Hz, 1H), 7.33 (d, *J* = 7.6 Hz, 1H), 7.27 − 7.25 (m, 2H), 7.20 (t, *J* = 7.7 Hz, 1H), 7.07 (t, *J* = 7.7 Hz, 1H), 6.82 (d, *J* = 7.9 Hz, 1H), 6.77 (d, *J* = 15.7 Hz, 1H), 6.11 (dt, *J* = 15.7, 5.0 Hz, 1H), 6.06 (d, *J* = 7.7 Hz, 1H), 5.74 (d, *J* = 7.7 Hz, 1H), 4.29 (d, *J* = 5.0 Hz, 2H). $^{13}C\{^1H\}$ NMR ($CDCl_3$, 101 MHz) δ 142.9, 141.3, 136.1, 135.3, 133.4, 131.3 (q, J_{C-F} = 33.5 Hz), 130.04 (q, J_{C-F} = 32.7 Hz), 130.02, 129.5, 129.1 (q, J_{C-F} = 3.4 Hz), 128.8, 128.1, 127.8, 127.7, 127.6, 126.8, 125.5 (q, J_{C-F} = 3.6 Hz), 124.0 (q, J_{C-F} = 3.6 Hz), 123.8 (q, J_{C-F} = 272.2 Hz), 122.9 (q, J_{C-F} = 273.1 Hz), 63.2, 58.4. ^{19}F NMR ($CDCl_3$, 376 MHz) δ –62.75 (s), –62.95 (s). HRMS (ESI) m/z: $[M + Na]^+$ Calcd for $C_{24}H_{19}F_6NNaO_3S$ 538.0882; Found 538.0872. HPLC analysis: Daicel Chiralcel AD−H (absorbance at 254 nm, mobile phase: *n*-hexane/ isopropanol = 88:12, flow rate 1.0 mL/min), er = 76.4:23.6 [tr (major, 74.6%) = 11.2 min, tr (minor, 23.6%) = 13.6 min]. $[\alpha]_D^{25}$= +28.4 (c 0.001, $CHCl_3$).

1-[4-(Trifluoromethyl)phenyl]-2-{[3-(trifluoromethyl)phenyl]sulfonyl}-3-vinylisoindoline (**2dh**)

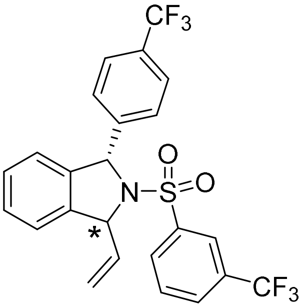

Following the general procedure C, the reaction of (±)-**1dh** (30.0 mg) afforded **2dh** [10.1 mg, 35%, 1:1.5 dr (*cis*/*trans*)] as a colorless oil. R_f = 0.41 [EtOAc:hexanes, 1:8 (v/v)]. ^{1}H NMR ($CDCl_3$, 400 MHz) δ 7.70 − 7.63 (m, 5H), 7.49 (d, *J* = 8.1 Hz, 3H), 7.42 − 7.39 (m, 4H), 7.36 − 7.22 (m, 6H), 7.19 (d, *J* = 7.6 Hz, 2H), 7.12 (d, *J* = 8.0 Hz, 2H), 6.88 (d, *J* = 7.0 Hz, 2H), 6.89 − 6.86 (m, 2H), 6.22 (s, 1H), 6.16 (d, *J* = 2.5 Hz, 1H), 6.00 − 5.91 (m, 1H), 5.84 − 5.73 (m, 2H), 5.58 (d, *J* = 16.5 Hz, 1H), 5.51 (d, *J* = 17.1 Hz, 1H), 5.49 (dd, *J* = 8.5, 2.5 Hz, 1H), 5.35 (d, *J* = 9.5 Hz, 2H). $^{13}C\{^1H\}$ NMR ($CDCl_3$, 101 MHz) δ 144.8, 144.2, 141.55, 141.45, 139.4, 138.7, 138.2, 137.7, 137.5, 137.4, 131.2 (q, J_{C-F} = 33.4 Hz), 130.5 (q, J_{C-F} = 32.5 Hz), 130.42 (q, J_{C-F} = 33.0 Hz), 130.38, 130.1, 129.2, 129.0, 128.9, 128.8 (q, J_{C-F} = 3.6 Hz), 128.7, 128.6, 128.5, 125.6 (q, J_{C-F} = 3.5 Hz), 125.4 (q, J_{C-F} = 3.5 Hz), 124.6 (q, J_{C-F} = 3.7 Hz), 124.3 (q, J_{C-F} = 3.7 Hz), 123.83 (q, J_{C-F} = 272.3 Hz), 123.77 (q, J_{C-F} = 273.0 Hz), 123.6, 123.5, 123.4, 123.3, 123.0 (q, J_{C-F} = 272.2 Hz), 119.1, 117.0, 69.7, 69.3, 68.9, 68.8. ^{19}F NMR ($CDCl_3$, 376 MHz) δ –62.76 (s), –62.77 (s), –62.96 (s), –63.06 (s). HRMS (ESI) m/z: $[M + H]^+$ Calcd for $C_{24}H_{18}F_6NO_2S$ 498.0957; Found 498.0938. HPLC analysis: Daicel Chiralcel AD−H (absorbance at 220 nm, mobile phase: *n*-hexane/ isopropanol = 98:2, flow rate 1.0 mL/min), er of *cis*-**2dh** = 90.5:9.5 [tr (minor, 3.9%) = 13.3 min, tr (major, 36.9%) = 16.0 min], er of *trans*-**2dh** = 90.4:9.6 [tr (major, 53.5%) = 14.8 min, tr (minor, 5.7%) = 25.6 min]. $[\alpha]_D^{25}$= +196.2 (c 0.003, $CHCl_3$).

(R,E)-N-{[2-(3-Hydroxyprop-1-en-1-yl)phenyl](3-methoxyphenyl)methyl}-3-(trifluoromethyl)benzenesulfonamide [(R)-**1eh**]

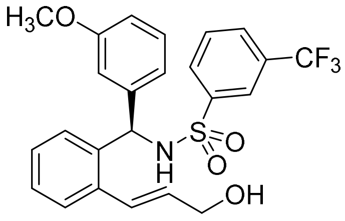

Following the general procedure C, the reaction of (±)-**1eh** (30.0 mg) resulted in the recovery of (R)-**1eh** (6.1 mg, 20%). HPLC analysis: Daicel Chiralcel AD−H (absorbance at 254 nm, mobile phase: n-hexane/ isopropanol = 88:12, flow rate 1.0 mL/min), er = 80.6:19.4 [tr (minor, 19.4%) = 17.3 min, tr (major, 80.6%) = 23.4 min]. $[\alpha]_D^{25}$= +278.7 (c 0.001, $CHCl_3$).

1-(3-Methoxyphenyl)-2-{[3-(trifluoromethyl)phenyl]sulfonyl}-3-vinylisoindoline (**2eh**)

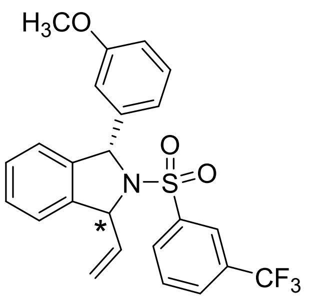

Following the general procedure C, the reaction of (±)-**1eh** (30.0 mg) afforded **2eh** [7.2 mg, 25%, 1:1.2 dr (cis/$trans$)] as a colorless oil. R_f = 0.49 [EtOAc:hexanes, 1:8 (v/v)]. ^{1}H NMR ($CDCl_3$, 400 MHz) δ 7.68 (s, 1H), 7.68 −7.52 (m, 5H), 7.39 (t, J = 7.8 Hz, 2H), 7.32−7.27 (m, 2H), 7.25−7.20 (m, 2H), 7.17−7.12 (m, 3H), 7.03 (t, J = 8.0 Hz, 1H), 6.93 (d, J = 7.2 Hz, 1H), 6.91 (d, J = 7.1 Hz, 1H), 6.77−6.74 (m, 2H), 6.70 (dd, J = 8.2, 2.4 Hz, 1H), 6.61 (t, J = 1.8 Hz, 1H), 6.58 (d, J = 7.6 Hz, 1H), 6.38 (s, 1H), 6.15 (s, 1H), 6.08 (d, J = 2.7 Hz, 1H), 6.05−5.99 (m, 1H), 5.82−5.74 (m, 2H), 5.61−5.54 (m, 1H), 5.48 (d, J = 17.0 Hz, 1H), 5.42 (dd, J = 8.7, 2.4 Hz, 1H), 5.34 (d, J = 9.9 Hz, 1H), 5.32 (d, J = 9.3 Hz, 1H), 3.66 (s, 3H), 3.16 (s, 3H). ^{13}C{^{1}H} NMR ($CDCl_3$, 101 MHz) δ 159.7, 159.4, 142.15, 142.08, 141.5, 141.2, 140.1, 139.6, 138.9, 137.8, 137.7, 137.3, 130.89 (q, J_{C-F} = 33.3 Hz), 130.85 (q, J_{C-F} = 33.6 Hz), 130.5, 130.2, 129.6, 129.3, 129.0, 128.8, 128.7, 128.5 (q, J_{C-F} = 3.7 Hz), 128.3, 128.2, 124.6 (q, J_{C-F} = 3.9 Hz), 124.2 (q, J_{C-F} = 3.9 Hz), 123.5, 123.4, 123.3, 123.2 (q, J_{C-F} = 269.9 Hz), 123.1 (q, J_{C-F} = 270.5 Hz), 121.0, 120.5, 118.8, 116.3, 114.4, 113.9, 113.4, 113.3, 69.9, 69.52, 69.48, 68.7, 55.0. ^{19}F NMR ($CDCl_3$, 376 MHz) δ –62.62 (s), –62.83 (s). HRMS (ESI) m/z: $[M + Na]^+$ Calcd for $C_{24}H_{20}F_3NNaO_3S$ 482.1008; Found 482.0996. HPLC analysis: Daicel Chiralcel AD−H (absorbance at 220 nm, mobile phase: n-hexane/ isopropanol = 95:5, flow rate 1.0 mL/min), er of cis-**2eh** = 88.5:11.5 [tr (minor, 5.1%) = 10.7 min, tr (major, 39.2%) = 26.2 min], er of $trans$-**2eh** = 88.7:11.3 [tr (major, 49.4%) = 16.7 min, tr (minor, 6.3%) = 23.3 min]. $[\alpha]_D^{25}$= +194.4 (c 0.001, $CHCl_3$).

(*R*,*E*)-*N*-{[2-(3-Hydroxyprop-1-en-1-yl)phenyl](m-tolyl)methyl}-3-(trifluoromethyl)benzenesulfonamide [(*R*)-**1fh**]

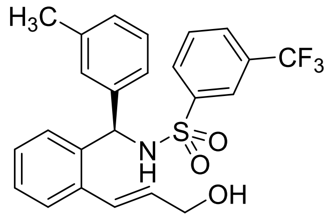

Following the general procedure C, the reaction of (±)-**1fh** (30.0 mg) resulted in the recovery of (*R*)-**1fh** (13.1 mg, 44%) as a yellow oil. R_f = 0.66 [EtOAc:hexanes, 1:1 (v/v)]. ^{1}H NMR (CD_3OD, 400 MHz) δ 7.84 (d, *J* = 7.8 Hz, 1H), 7.77 (s, 1H), 7.70 (d, *J* = 7.9 Hz, 1H), 7.51 (t, *J* = 7.8 Hz, 1H), 7.35 (d, *J* = 7.8 Hz, 1H), 7.12 (td, *J* = 7.3, 2.3 Hz, 1H), 7.08−7.03 (m, 3H), 6.97 (d, *J* = 7.5 Hz, 1H), 6.89−6.84 (m, 3H), 6.12 (dt, *J* = 15.6, 5.4 Hz, 1H), 5.95 (s, 1H), 4.20 (dd, *J* = 5.4, 1.5 Hz, 2H), 2.19 (s, 3H). ^{13}C{^{1}H} NMR ($CDCl_3$, 101 MHz) δ 141.5, 138.5, 138.3, 136.2, 136.1, 132.8, 131.1 (q, $J_{C\text{–}F}$ = 33.4 Hz), 130.1, 129.2, 128.8 (q, $J_{C\text{–}F}$ = 3.4 Hz), 128.5, 128.4, 128.3, 128.1, 128.0, 127.6, 127.5, 127.3, 124.4, 124.0, (q, $J_{C\text{–}F}$ = 3.7 Hz), 123.0 (q, $J_{C\text{–}F}$ = 273.0 Hz), 63.4, 58.7, 21.2. ^{19}F NMR ($CDCl_3$, 376 MHz) δ –62.80 (s). HRMS (ESI) m/z: [M + Na]$^+$ Calcd for $C_{24}H_{22}F_3NNaO_3S$ 484.1165; Found 484.1159. HPLC analysis: Daicel Chiralcel AD−H (absorbance at 254 nm, mobile phase: *n*-hexane/ isopropanol = 88:12, flow rate 1.0 mL/min), er = 74.1:25.9 [tr (minor, 25.9%) = 11.4 min, tr (major, 74.1%) = 13.9 min]. $[\alpha]_D^{25}$= +38.2 (c 0.001, $CHCl_3$).

1-(*m*-Tolyl)-2-{[3-(trifluoromethyl)phenyl]sulfonyl}-3-vinylisoindoline (**2fh**)

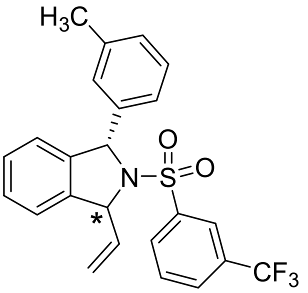

Following the general procedure C, the reaction of (±)-**1fh** (30.0 mg) afforded **2fh** [4.0 mg, 14%, 1:1 dr (*cis*/*trans*)] as a colorless oil. R_f = 0.49 [EtOAc:hexanes, 1:8 (v/v)]. ^{1}H NMR ($CDCl_3$, 400 MHz) δ 7.65−7.60 (m, 4H), 7.49−4.47 (m, 2H), 7.36 (t, *J* = 7.8 Hz, 2H), 7.32−7.28 (m, 2H), 7.25 −7.16 (m, 4H), 7.08 (t, *J* = 7.4 Hz, 1H), 7.03−6.90 (m, 7H), 6.77 (d, *J* = 6.6 Hz, 1H), 6.63 (s, 1H), 6.14 (s, 1H), 6.10−6.02 (m, 2H), 5.83−5.74 (m, 2H), 5.61−5.55 (m, 1H), 5.48 (d, *J* = 17.0 Hz, 1H), 5.44 (dd, *J* = 8.7, 2.6 Hz, 1H), 5.36−5.31 (m, 2H), 2.20 (s, 3H), 2.10 (s, 3H). ^{13}C{^{1}H} NMR ($CDCl_3$, 101 MHz) δ 142.1, 141.5, 140.42, 140.37, 139.8, 139.4, 139.0, 138.2, 137.9, 137.8, 137.7, 137.3, 130.8 (q, $J_{C\text{–}F}$ = 33.6 Hz), 130.5, 130.1, 129.1, 129.0, 128.9, 128.8, 128.6, 128.4, 128.2, 128.1, 125.9, 125.3, 124.53 (q, $J_{C\text{–}F}$ = 4.0 Hz), 124.1, 123.6, 123.4, 123.3, 123.23, 123.15 (q, $J_{C\text{–}F}$ = 274.1 Hz), 118.80, 118.76, 116.2, 70.0, 69.6, 69.5, 68.7, 21.2, 21.1. ^{19}F NMR ($CDCl_3$, 376 MHz) δ –62.64 (s), –62.83 (s). HRMS (ESI) m/z: [M +Na]$^+$ Calcd for $C_{24}H_{20}F_3NNaO_2S$ 466.1059; Found 466.1056. HPLC analysis: Daicel Chiralcel OD−H (absorbance at 220 nm, mobile phase: *n*-hexane/ isopropanol = 99:1, flow rate 1.0 mL/min), er of *cis*-**2fh** = 91.4:8.6 [tr (minor, 4.4%) = 11.4 min, tr (major, 46.9%) = 13.6 min], er of *trans*-**2fh** = 91.8:8.2 [tr (minor, 4.0%) = 9.6 min, tr (major, 44.7%) = 10.5 min]. $[\alpha]_D^{25}$= +169.8 (c 0.001, $CHCl_3$).

(R,E)-N-{[3,5-Bis(trifluoromethyl)phenyl][2-(3-hydroxyprop-1-en-1-yl)phenyl]methyl}-3-(trifluoromethyl)benzenesulfonamide [(R)-**1gh**]

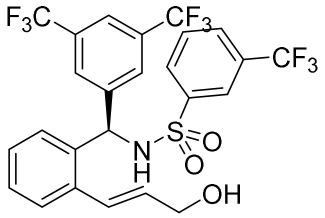

Following the general procedure C, the reaction of (±)-**1gh** (30.0 mg) resulted in the recovery of (R)-**1gh** (7.8 mg, 26%) as a yellow oil. R_f = 0.77 [EtOAc:hexanes, 1:1 (v/v)]. ^{1}H NMR ($CDCl_3$, 400 MHz) δ 7.79 – 7.75 (m, 2H), 7.71 (s, 1H), 7.66 (d, J = 7.8 Hz, 1H), 7.58 (s, 2H), 7.44 (t, J = 7.8 Hz, 1H), 7.37 (d, J = 7.8 Hz, 1H), 7.26 – 7.23 (m, 1H), 7.10 (t, J = 7.4 Hz, 1H), 6.84 (d, J = 15.6 Hz, 1H), 6.72 (d, J = 7.6 Hz, 1H), 6.19 – 6.13 (m, 2H), 5.86 (d, J = 7.3 Hz, 1H), 4.32 (d, J = 4.8 Hz, 2H). ^{13}C{^{1}H} NMR ($CDCl_3$, 101 MHz) δ 141.8, 141.1, 136.3, 134.7, 133.9, 131.9 (q, J_{C-F} = 33.6 Hz), 131.5 (q, J_{C-F} = 33.8 Hz), 129.8, 129.7, 129.4 (q, J_{C-F} = 3.2 Hz), 129.3, 128.1, 127.99, 127.96, 127.4, 126.4, 123.8 (q, J_{C-F} = 3.7 Hz), 122.9 (q, J_{C-F} = 273.0 Hz), 122.8 (q, J_{C-F} = 273.3 Hz), 121.8 (q, J_{C-F} = 3.6 Hz), 63.1, 58.1. ^{19}F NMR ($CDCl_3$, 376 MHz) δ –62.90 (s), –63.07 (s). HRMS (ESI) m/z: $[M + Na]^+$ Calcd for $C_{25}H_{18}F_9NNaO_3S$ 606.0756; Found 606.0753. HPLC analysis: Daicel Chiralcel OD−H (absorbance at 254 nm, mobile phase: n-hexane/ isopropanol = 94:6, flow rate 1.0 mL/min), er = 94.1:5.9 [tr (major, 94.1%) = 9.1 min, tr (minor, 5.9%) = 12.9 min. $[\alpha]_D^{25}$= +282.1 (c 0.001, $CHCl_3$).

1-[3,5-Bis(trifluoromethyl)phenyl]-2-{[3-(trifluoromethyl)phenyl]sulfonyl}-3-vinylisoindoline (**2gh**)

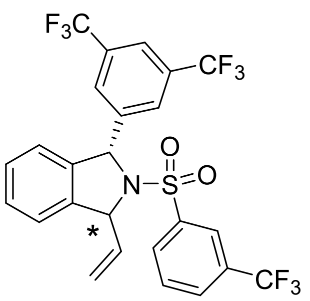

Following the general procedure C, the reaction of (±)-**1gh** (30.0 mg) afforded **2gh** [10.1 mg, 35%, 1:1.6 dr (cis/$trans$)] as a yellow oil. R_f = 0.63 [EtOAc:hexanes, 1:8 (v/v)]. ^{1}H NMR ($CDCl_3$, 400 MHz) δ 7.73 – 7.56 (m, 10H), 7.47 – 7.35 (m, 6H), 7.30 – 7.21 (m, 4H), 6.89 – 6.85 (m, 2H), 6.27 (s, 1H), 6.21 (d, J = 2.7 Hz, 1H), 6.02 – 5.93 (m, 1H), 5.89 – 5.77 (m, 2H), 5.62 (d, J = 16.3 Hz, 1H), 5.59 (dd, J = 8.6, 2.6 Hz, 1H), 5.54 (d, J = 17.0 Hz, 1H), 5.44 (d, J = 9.4 Hz, 1H), 5.36 (d, J = 10.0 Hz, 1H). ^{13}C{^{1}H} NMR ($CDCl_3$, 101 MHz) δ 143.5, 143.0, 141.5, 141.3, 138.4, 138.0, 137.9, 137.8, 137.3, 137.2, 132.0 (q, J_{C-F} = 33.4 Hz), 131.8 (q, J_{C-F} = 33.6 Hz), 131.0 (q, J_{C-F} = 33.3 Hz), 130.2, 129.8, 129.5, 129.34 (q, J_{C-F} = 3.6 Hz), 129.29, 129.14, 129.10, 128.3, 127.9, 124.3 (q, J_{C-F} = 3.8 Hz), 124.2, 123.9, 123.8, 123.3, 122.9 (q, J_{C-F} = 273.1 Hz), 122.8 (q, J_{C-F} = 272.7 Hz), 122.3 (q, J_{C-F} = 3.9 Hz), 122.2 (q, J_{C-F} = 3.6 Hz), 119.7, 117.3, 69.9, 68.8, 68.7, 68.5. ^{19}F NMR ($CDCl_3$, 376 MHz) δ –62.86 (s), –62.97 (s), –62.99 (s), –63.15 (s). HRMS (ESI) m/z: $[M + H]^+$ Calcd for $C_{25}H_{17}F_9NO_2S$ 566.0831; Found 566.0830. HPLC analysis: Daicel Chiralcel AD−H (absorbance at 220 nm, mobile phase: n-hexane/ isopropanol = 99:1, flow rate 1.0 mL/min), er of cis-**2gh** = 68.3:31.7 [tr (minor, 10.8%) = 6.4 min, tr (major, 23.2%) = 11.4 min], er of $trans$-**2gh** = 64.6:35.4 [tr (major, 42.6%) = 7.9 min, tr (minor, 23.4%) = 12.2 min]. $[\alpha]_D^{25}$= +178.2 (c 0.001, $CHCl_3$).

(R,E)-N-{Benzo[d][1,3]dioxol-5-yl[2-(3-hydroxyprop-1-en-1-yl)phenyl]methyl}-3-(trifluoromethyl)benzenesulfonamide [(R)-**1hh**]

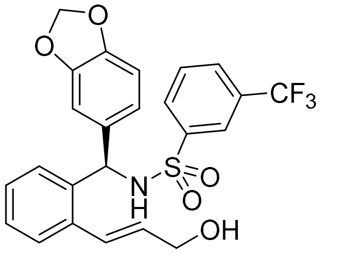

Following the general procedure C, the reaction of (±)-**1hh** (30.0 mg) resulted in the recovery of (R)-**1hh** (15.1 mg, 50%) as a brown oil. R_f = 0.53 [EtOAc:hexanes, 1:1 (v/v)]. ^{1}H NMR ($CDCl_3$, 400 MHz) δ 7.77−7.75 (m, 2H), 7.63 (d, J = 7.7 Hz, 1H), 7.41 (t, J = 7.8 Hz, 1H), 7.30 (d, J = 7.6 Hz, 1H), 7.15 (t, J = 7.4 Hz, 1H), 7.04 (t, J = 7.6 Hz, 1H), 6.91 (d, J = 7.7 Hz, 1H), 6.79 (d, J = 15.7 Hz, 1H), 6.62−6.55 (m, 3H), 6.09 (dt, J = 15.7, 5.3 Hz, 1H), 5.92−5.88 (m, 3H), 5.57 (d, J = 7.7 Hz, 1H), 4.28 (d, J = 5.3 Hz, 2H). ^{13}C{^{1}H} NMR ($CDCl_3$, 101 MHz) δ 147.9, 147.2, 141.5, 136.0, 135.9, 132.8, 132.6, 131.1 (q, $J_{C\text{–}F}$ = 33.4 Hz), 130.1, 129.3, 128.8 (q, $J_{C\text{–}F}$ = 3.2 Hz), 128.3, 127.8, 127.6, 127.4, 127.3, 124.0 (q, $J_{C\text{–}F}$ = 3.8 Hz), 123.0 (q, $J_{C\text{–}F}$ = 273.0 Hz), 120.9, 108.1, 107.9, 101.2, 63.3, 58.5. ^{19}F NMR ($CDCl_3$, 376 MHz) δ –62.84 (s). HRMS (ESI) m/z: $[M + Na]^+$ Calcd for $C_{24}H_{20}F_3NNaO_5S$ 514.0906; Found 514.0905. HPLC analysis: Daicel Chiralcel AD−H (absorbance at 254 nm, mobile phase: n-hexane/ isopropanol = 88:12, flow rate 1.0 mL/min), er = 78.5:21.5 [tr (minor, 21.5%) = 24.4 min, tr (major, 78.5%) = 31.5 min]. $[\alpha]_D^{25}$= +264.9 (c 0.002, $CHCl_3$).

1-{Benzo[d][1,3]dioxol-5-yl}-2-{[3-(trifluoromethyl)phenyl]sulfonyl}-3-vinylisoindoline (**2hh**)

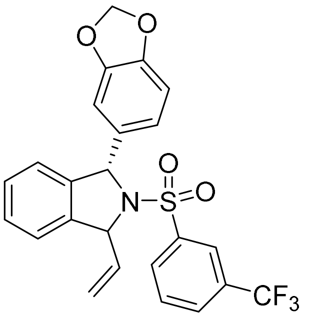

Following the general procedure C, the reaction of (±)-**1hh** (30.0 mg) afforded **2hh** [6.5 mg, 23%, 1:1.5 dr (cis/$trans$)] as a colorless oil. R_f = 0.31 [EtOAc:hexanes, 1:8 (v/v)]. ^{1}H NMR ($CDCl_3$, 400 MHz) δ 7.75−7.29 (m, 10H), 7.25−7.15 (m, 4H), 7.25−7.15 (m, 6H), 6.40 (s, 1H), 6.12 (s, 2H), 6.07−5.98 (m, 2H), 5.89 (d, J = 6.2 Hz, 2H), 5.85 (d, J = 6.4 Hz, 2H), 5.77−5.68 (m, 2H), 5.58−5.53 (m, 1H), 5.47 (d, J = 17.0 Hz, 1H), 5.41 (d, J = 8.2 Hz, 1H), 5.33 (d, J = 10.0 Hz, 1H), 5.30 (d, J = 9.0 Hz, 1H). ^{13}C{^{1}H} NMR ($CDCl_3$, 101 MHz) δ 147.9, 147.6, 142.2, 141.6, 140.1, 139.5, 138.9, 137.7, 137.3, 134.6, 133.4, 130.9 (q, $J_{C\text{–}F}$ = 33.2 Hz), 130.5, 130.2, 129.0, 128.8, 128.7, 128.53, 128.51 (q, $J_{C\text{–}F}$ = 3.0 Hz), 128.3, 128.2, 124.5 (q, $J_{C\text{–}F}$ = 4.1 Hz), 124.2 (q, $J_{C\text{–}F}$ = 4.0 Hz), 123.5, 123.4, 123.3, 123.2 (q, $J_{C\text{–}F}$ = 273.5 Hz), 123.1 (q, $J_{C\text{–}F}$ = 271.8 Hz), 122.8, 122.3, 118.8, 116.3, 108.4, 108.1, 107.9, 107.7, 101.2, 69.7, 69.4, 69.3, 68.6. ^{19}F NMR ($CDCl_3$, 376 MHz) δ –62.84 (s), –62.92 (s). HRMS (ESI) m/z: $[M + H]^+$ Calcd for $C_{24}H_{19}F_3NO_4S$ 474.0981; Found 474.0980. HPLC analysis: Daicel Chiralcel AD−H (absorbance at 220 nm, mobile phase: n-hexane/ isopropanol = 95:5, flow rate 1.0 mL/min), er of cis-**2hh** = 92.6:7.4 [tr (minor, 3.0%) = 18.4 min, tr (major, 37.0%) = 30.5 min], er of $trans$-**2hh** = 92.4:7.6 [tr (major, 55.5%) = 28.8 min, tr (minor, 4.6%) = 41.5 min]. $[\alpha]_D^{25}$= +276.9 (c 0.001, $CHCl_3$).

(*R,E*)-*N*-{[2-(3-Hydroxyprop-1-en-1-yl)phenyl](naphthalen-2-yl)methyl}-3-(trifluoromethyl)benzenesulfonamide [(*R*)-**1ih**]

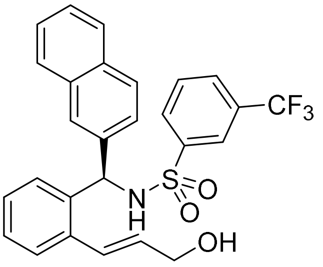

Following the general procedure C, the reaction of (±)-**1ih** (30.0 mg) resulted in the recovery of (*R*)-**1ih** (12.3 mg, 41%) as a white solid. R_f = 0.65 [EtOAc:hexanes, 1:1 (v/v)]. Mp 112−113 °C. ^{1}H NMR ($CDCl_3$, 400 MHz) δ 7.80 (s, 1H), 7.77−7.72 (m, 2H), 7.69 (d, *J* = 8.6 Hz, 1H), 7.61 (d, *J* = 7.2 Hz, 1H), 7.53 (d, *J* = 7.7 Hz, 1H), 7.48 (s, 1H), 7.46−7.42 (m, 2H), 7.35 (d, *J* = 7.8 Hz, 1H), 7.31−7.24 (m, 2H), 7.19 (t, *J* = 7.5 Hz, 1H), 7.05 (t, *J* = 7.6 Hz, 1H), 6.92 (d, *J* = 7.8 Hz, 1H), 6.87 (d, *J* = 15.6 Hz, 1H), 6.19 (d, *J* = 7.8 Hz, 1H), 6.12 (dt, *J* = 15.6, 5.3 Hz, 1H), 5.58 (d, *J* = 7.8 Hz, 1H), 4.27 (d, *J* = 5.3 Hz, 2H). ^{13}C{^{1}H} NMR ($CDCl_3$, 101 MHz) δ 141.5, 136.2, 136.1, 136.0, 132.9, 132.6, 131.0 (q, $J_{C\text{–}F}$ = 33.4 Hz), 130.0, 129.2, 128.7 (q, $J_{C\text{–}F}$ = 3.2 Hz), 128.5, 128.4, 128.3, 127.9, 127.6, 127.5, 127.4, 126.4, 126.35, 126.31, 125.2, 123.9 (q, $J_{C\text{–}F}$ = 3.8 Hz), 123.0 (q, $J_{C\text{–}F}$ = 272.9 Hz), 63.3, 58.9. ^{19}F NMR ($CDCl_3$, 376 MHz) δ –62.82 (s). HRMS (ESI) m/z: $[M + Na]^+$ Calcd for $C_{27}H_{22}F_3NNaO_3S$ 520.1165; Found 520.1166. HPLC analysis: Daicel Chiralcel AD−H (absorbance at 254 nm, mobile phase: *n*-hexane/ isopropanol = 88:12, flow rate 1.0 mL/min), er = 99.5:0.5 [tr (minor, 0.5%) = 18.8 min, tr (major, 99.5%) = 21.7 min]. $[\alpha]_D^{25}$= +292.7 (c 0.001, $CHCl_3$).

1-(Naphthalen-2-yl)-2-{[3-(trifluoromethyl)phenyl]sulfonyl}-3-vinylisoindoline (**2ih**)

Following the general procedure C, the reaction of (±)-**1ih** (30.0 mg) afforded **2ih** [7.5 mg, 26%, 1:1.6 dr (*cis*/*trans*)] as a white solid. R_f = 0.61 [EtOAc:hexanes, 1:8 (v/v)]. Mp 198−199 °C. ^{1}H NMR ($CDCl_3$, 400 MHz) δ 7.80−7.68 (m, 7H), 7.59−7.30 (m, 12H), 7.24−6.88 (m, 10H), 6.72 (dd, *J* = 8.6, 1.5 Hz, 1H), 6.35 (s, 1H, H^1 of *trans*-**2ih**), 6.27 (d, *J* = 2.5 Hz, 1H, H^1 of *cis*-**2ih**), 6.12−6.03 (m, 1H, H^a of *cis*-**2ih**), 5.91−5.79 (m, 2H, H^3 and H^a of *trans*-**2ih**), 5.61 (d, *J* = 16.0 Hz, 1H, H^c of *trans*-**2ih**), 5.55 (dd, *J* = 9.2, 2.4 Hz, 1H, H^3 of *cis*-**2ih**), 5.52 (d, *J* = 17.2 Hz, 1H, H^c of *cis*-**2ih**), 5.37−5.34 (m, 2H, H^b of *cis*- and *trans*-**2ih**). ^{13}C{^{1}H} NMR ($CDCl_3$, 101 MHz) δ 141.9, 141.3, 140.1, 139.5, 138.8, 137.8, 137.7, 137.6, 137.5, 136.8, 133.0, 132.95, 132.87, 132.8, 130.8 (q, $J_{C\text{–}F}$ = 33.0 Hz), 130.7 (q, $J_{C\text{–}F}$ = 33.7 Hz), 130.4, 130.1, 128.8 (q, $J_{C\text{–}F}$ = 3.1 Hz), 128.71, 128.66, 128.5 (q, $J_{C\text{–}F}$ = 3.3 Hz), 128.4, 128.3, 128.24, 128.19, 128.0, 127.9, 127.62, 127.60 ,126.43, 126.40, 125.2, 125.0, 124.5 (q, $J_{C\text{–}F}$ = 3.6 Hz), 124.1 (q, $J_{C\text{–}F}$ = 3.9 Hz), 123.7, 123.6, 123.4, 123.3, 122.9 (q, $J_{C\text{–}F}$ = 272.8 Hz), 118.9, 116.5, 70.1, 69.8, 69.7, 68.8. ^{19}F NMR ($CDCl_3$, 376 MHz) δ –63.11 (s), –63.19 (s). HRMS (ESI) m/z: $[M + Na]^+$ Calcd for $C_{27}H_{20}F_3NNaO_2S$ 502.1059; Found 502.1061. HPLC analysis: Daicel Chiralcel AD−H (absorbance at 220 nm, mobile phase: *n*-hexane/ isopropanol = 98:2, flow rate 1.0 mL/min), er of *cis*-**2ih** = 88.6:11.4 [tr (minor, 3.4%) = 22.1 min, tr (major, 26.7%) = 41.0 min], er of *trans*-**2ih** = 92.5:7.5 [tr (major, 64.6%) = 29.5 min, tr (minor, 5.2%) = 47.3 min]. $[\alpha]_D^{25}$= +131.1 (c 0.001, $CHCl_3$).

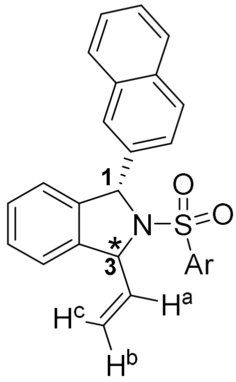

Ar = 3=CF_3-C_6H_4

(*R*,*E*)-N-{[2-(3-Hydroxyprop-1-en-1-yl)-4-methylphenyl](naphthalen-2-yl)methyl}-3-(trifluoromethyl)benzenesulfonamide [(*R*)-**1jh**]

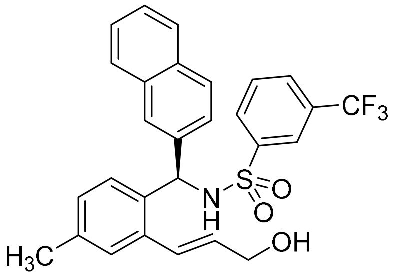

Following the general procedure C, the reaction of (±)-**1jh** (30.0 mg) resulted in the recovery of (*R*)-**1jh** (15.2 mg, 51%) as a yellow oil. R_f = 0.75 [EtOAc:hexanes, 1:1 (v/v)]. ^{1}H NMR ($CDCl_3$, 400 MHz) δ 7.77−7.74 (m, 3H), 7.67 (d, *J* = 8.6 Hz, 1H), 7.60 (d, *J* = 7.2 Hz, 1H), 7.53 (d, *J* = 7.8 Hz, 1H), 7.49 (s, 1H), 7.47−7.41 (m, 2H), 7.31−7.23 (m, 2H), 7.15 (s, 1H), 6.86−6.78 (m, 3H), 6.15−6.07 (m, 2H), 5.59 (s, 1H), 4.25 (d, *J* = 4.8 Hz, 2H), 2.27 (s, 3H), 1.98 (s, 1H). ^{13}C{^{1}H} NMR ($CDCl_3$, 101 MHz) δ 141.5, 138.0, 136.3, 135.8, 133.1, 132.8, 132.5, 132.4, 130.8 (q, $J_{C\text{–}F}$ = 33.4 Hz), 130.0, 129.1, 128.5, 128.3, 127.9, 127.8, 127.5, 126.3, 126.2, 126.1, 125.2, 123.8 (q, $J_{C\text{–}F}$ = 3.7 Hz), 123.0 (q, $J_{C\text{–}F}$ = 272.9 Hz), 63.2, 58.7, 20.9. ^{19}F NMR ($CDCl_3$, 376 MHz) δ –62.94 (s). HRMS (ESI) m/z: $[M + Na]^+$ Calcd for $C_{28}H_{24}F_3NNaO_3S$ 534.1321; Found 534.1323. HPLC analysis: Daicel Chiralcel AD−H (absorbance at 254 nm, mobile phase: *n*-hexane/ isopropanol = 85:15, flow rate 1.0 mL/min), er = 74.9:25.1 [tr (minor, 25.1%) = 12.6 min, tr (major, 74.9%) = 16.7 min]. $[\alpha]_D^{25}$= +46.1 (c 0.002, $CHCl_3$).

5-Methyl-1-(naphthalen-2-yl)-2-{[3-(trifluoromethyl)phenyl]sulfonyl}-3-vinylisoindoline (**2jh**)

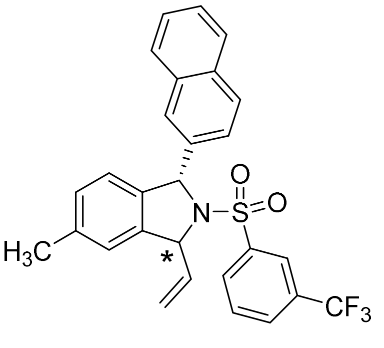

Following the general procedure C, the reaction of (±)-**1jh** (30.0 mg) afforded **2jh** [8.2 mg, 28%, 1:1.8 dr (*cis*/*trans*)] as a colorless oil. R_f = 0.46 [EtOAc:hexanes, 1:8 (v/v)]. ^{1}H NMR ($CDCl_3$, 400 MHz) δ 7.79−7.69 (m, 5H), 7.68 (s, 2H), 7.58 (d, *J* = 8.5 Hz, 1H), 7.54−7.39 (m, 9H), 7.34 (d, *J* = 7.8 Hz, 1H), 7.15 (t, *J* = 7.8 Hz, 1H), 7.07−6.97 (m, 6H), 6.79 (d, *J* = 8.1 Hz, 1H), 6.77 (d, *J* = 8.8 Hz, 1H), 6.71 (d, *J* = 8.6 Hz, 1H), 6.30 (s, 1H), 6.23 (s, 1H), 6.10−6.01 (m, 1H), 5.89−5.80 (m, 1H), 5.74 (d, *J* = 8.6 Hz, 1H), 5.60 (d, *J* = 16.8 Hz, 1H), 5.54−5.49 (m, 2H), 5.36 (d, *J* = 10.0 Hz, 1H), 5.35 (d, *J* = 9.8 Hz, 1H), 2.35 (s, 6H). ^{13}C{^{1}H} NMR ($CDCl_3$, 101 MHz) δ 141.8, 141.4, 138.9, 138.4, 138.3, 137.6, 137.3, 137.0, 136.6, 133.0, 132.92, 132.88, 132.8, 130.74 (q, $J_{C\text{–}F}$ = 33.2 Hz), 130.67 (q, $J_{C\text{–}F}$ = 33.2 Hz), 130.4, 130.1, 129.8, 129.7, 128.83, 128.78, 128.6, 128.5 (q, $J_{C\text{–}F}$ = 3.3 Hz), 128.3 (q, $J_{C\text{–}F}$ = 3.4 Hz), 128.2, 128.1, 128.0, 127.8, 127.6, 126.4, 126.3, 125.2, 125.0, 124.5 (q, $J_{C\text{–}F}$ = 3.7 Hz), 124.0 (q, $J_{C\text{–}F}$ = 3.9 Hz), 123.75, 123.67, 123.4, 123.3, 122.9 (q, $J_{C\text{–}F}$ = 273.9 Hz), 118.8, 116.3, 69.9, 69.7, 68.8, 21.3. ^{19}F NMR ($CDCl_3$, 376 MHz) δ –63.11 (s), –63.18 (s). HRMS (ESI) m/z: $[M + Na]^+$ Calcd for $C_{28}H_{22}F_3NNaO_2S$ 516.1216; Found 516.1201. HPLC analysis: Daicel Chiralcel IC (absorbance at 220 nm, mobile phase: *n*-hexane/ isopropanol = 93:7, flow rate 0.8 mL/min), er of *cis*-**2jh** = 94.0:6.0 [tr (major, 33.9%) = 11.1 min, tr (minor, 2.2%) = 11.6 min], er of *trans*-**2jh** = 93.7:6.3 [tr (minor, 4.0%) = 9.4 min, tr (major, 59.9%) = 9.8 min]. $[\alpha]_D^{25}$= +122.0 (c 0.001, $CHCl_3$).

(*R*,*E*)-*N*-{[4-Fluoro-2-(3-hydroxyprop-1-en-1-yl)phenyl](naphthalen-2-yl)methyl}-3-(trifluoromethyl)benzenesulfonamide [(*R*)-**1kh**]

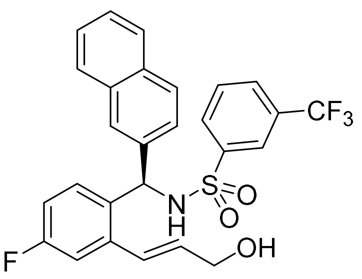

Following the general procedure C, the reaction of (±)-**1kh** (26.0 mg) resulted in the recovery of (*R*)-**1kh** (13.5 mg, 52%) as a colorless oil. R_f = 0.75 [EtOAc:hexanes, 1:1 (v/v)]. ^{1}H NMR ($CDCl_3$, 400 MHz) δ 7.79 (s, 1H), 7.75−7.70 (m, 2H), 7.66 (d, *J* = 8.6 Hz, 1H), 7.58 (d, *J* = 7.9 Hz, 1H), 7.52 (d, *J* = 7.9 Hz, 1H), 7.47−7.40 (m, 3H), 7.27 (t, *J* = 7.9 Hz, 1H), 7.21 (dd, *J* = 8.5, 1.6 Hz, 1H), 7.01 (dd, *J* = 9.8, 2.6 Hz, 1H), 6.91 (dd, *J* = 8.6, 5.7 Hz, 1H), 6.83 (d, *J* = 15.6 Hz, 1H), 6.72 (td, *J* = 8.2, 2.7 Hz, 1H), 6.15−6.05 (m, 3H), 4.24 (d, *J* = 4.8 Hz, 2H). $^{13}C\{^1H\}$ NMR ($CDCl_3$, 101 MHz) δ 162.3 (d, J_{C-F} = 247.6 Hz), 141.4, 138.5 (d, J_{C-F} = 7.9 Hz), 135.8, 133.9, 132.8, 132.6, 132.0, 131.9, 131.1 (q, J_{C-F} = 33.5 Hz), 130.4 (d, J_{C-F} = 8.5 Hz), 130.0, 129.3, 128.8 (q, J_{C-F} = 3.3 Hz), 128.6, 127.8, 127.5, 126.5 (d, J_{C-F} = 3.3 Hz), 126.2, 126.0, 125.0, 123.9 (q, J_{C-F} = 3.7 Hz), 122.9 (q, J_{C-F} = 273.0 Hz), 114.2 (d, J_{C-F} = 21.5 Hz), 113.8 (d, J_{C-F} = 22.2 Hz), 62.9, 58.4. ^{19}F NMR ($CDCl_3$, 376 MHz) δ –62.95 (s), –113.68 to –113.71 (m). HRMS (ESI) m/z: $[M + Na]^+$ Calcd for $C_{27}H_{21}F_4NNaO_3S$ 538.1070; Found 538.1065. HPLC analysis: Daicel Chiralcel AD−H (absorbance at 254 nm, mobile phase: *n*-hexane/ isopropanol = 88:12, flow rate 1.0 mL/min), er = 73.0:27.0 [tr (minor, 27.0%) = 19.0 min, tr (major, 73.0%) = 23.9 min]. $[\alpha]_D^{25}$= +61.1 (c 0.001, $CHCl_3$).

5-Fluoro-1-(naphthalen-2-yl)-2-{[3-(trifluoromethyl)phenyl]sulfonyl}-3-vinylisoindoline (**2kh**)

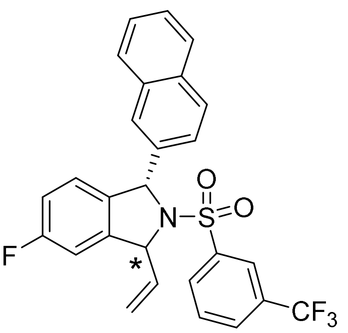

Following the general procedure C, the reaction of (±)-**1kh** (26.0 mg) afforded **2kh** [5.3 mg, 21%, 1:1.7 dr (*cis*/*trans*)] as a colorless oil. R_f = 0.41 [EtOAc:hexanes, 1:8 (v/v)]. ^{1}H NMR ($CDCl_3$, 400 MHz) δ 7.80−7.70 (m, 5H), 7.65 (d, *J* = 9.1 Hz, 2H), 7.42 (d, *J* = 8.5 Hz, 1H), 7.51−7.45 (m, 8H), 7.42 (d, *J* = 8.5 Hz, 1H), 7.34 (d, *J* = 7.8 Hz, 1H), 7.15 (t, *J* = 7.9 Hz, 1H), 7.06 (t, *J* = 7.8 Hz, 1H), 6.96−6.82 (m, 7H), 6.71 (dd, *J* = 8.2, 1.5 Hz, 1H), 6.31 (s, 1H), 6.22 (s, 1H), 6.12−6.03 (m, 1H), 5.88−5.76 (m, 2H), 5.62 (dd, *J* = 16.6, 1.0 Hz, 1H), 5.54−5.50 (m, 2H), 5.39 (d, *J* = 10.0 Hz, 1H), 5.38 (d, *J* = 8.7 Hz, 1H). $^{13}C\{^1H\}$ NMR ($CDCl_3$, 101 MHz) δ 163.0 (d, J_{C-F} = 247.1 Hz), 162.9 (d, J_{C-F} = 247.2 Hz), 141.8, 141.1, 139.9 (d, J_{C-F} = 8.6 Hz), 139.6 (d, J_{C-F} = 8.4 Hz), 138.3, 137.3, 137.1, 136.5, 135.7, 135.1, 133.1, 133.0, 132.8, 132.7, 130.8 (q, J_{C-F} = 33.1 Hz), 130.8 (q, J_{C-F} = 33.6 Hz), 130.3, 130.1, 128.9, 128.8, 128.6 (q, J_{C-F} = 3.4 Hz), 128.4 (q, J_{C-F} = 3.2 Hz), 128.3, 128.2, 128.0, 127.9, 127.6, 126.6 (d, J_{C-F} = 8.4 Hz), 125.3, 125.2, 125.1, 125.0, 124.8, 124.5 (q, J_{C-F} = 3.6 Hz), 124.1 (q, J_{C-F} = 3.7 Hz), 123.4 (q, J_{C-F} = 275.5 Hz), 122.9 (q, J_{C-F} = 273.0 Hz), 119.6, 117.0, 116.4 (d, J_{C-F} = 23.0 Hz), 116.3 (d, J_{C-F} = 23.0 Hz), 110.5 (d, J_{C-F} = 23.9 Hz), 110.3 (d, J_{C-F} = 23.7 Hz), 69.5, 69.4, 69.3, 68.5. ^{19}F NMR ($CDCl_3$, 376 MHz) δ –63.12 (s), –63.21 (s), –113.01 to –113.06 (m), –113.17 to –113.23 (m). HRMS (ESI) m/z: $[M + H]^+$ Calcd for $C_{27}H_{20}F_4NO_2S$ 498.1145; Found 498.1138. HPLC analysis: Daicel Chiralcel AD−H (absorbance at 220 nm, mobile phase: *n*-hexane/ isopropanol = 98:2, flow rate 1.0 mL/min), er of *cis*-**2kh** = 96.1:3.9 [tr (minor, 1.2%) = 21.2 min, tr (major, 30.4%) = 38.5 min], er of *trans*-**2kh** = 95.8:4.2 [tr (major, 65.6%) = 25.4 min, tr (minor, 2.8%) = 28.4 min]. $[\alpha]_D^{25}$= +173.9 (c 0.001, $CHCl_3$).

(*R*,*E*)-*N*-{1-[2-(3-hydroxyprop-1-en-1-yl)phenyl]ethyl}-3-(trifluoromethyl)benzenesulfonamide [(*R*)-**1lh**]

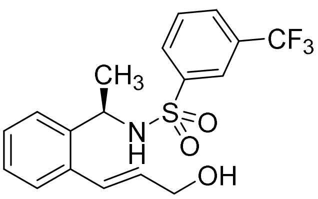

Following the general procedure C, the reaction of (±)-**1lh** (30.0 mg) resulted in the recovery of (*R*)-**1lh** 21.0 mg (70%). HPLC analysis: Daicel Chiralcel AD−H (absorbance at 254 nm, mobile phase: *n*-hexane/ isopropanol = 88:12, flow rate 1.0 mL/min), er = 58.6:41.4 [tr (minor, 41.4%) = 12.9 min, tr (major, 58.6%) = 14.2 min]. $[\alpha]_D^{25}$= +47.4 (c 0.002, $CHCl_3$).

1-Methyl-2-{[3-(trifluoromethyl)phenyl]sulfonyl}-3-vinylisoindoline (**2lh**)

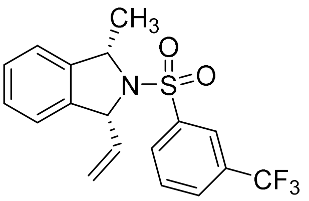

Following the general procedure C, the reaction of (±)-**1lh** (30.0 mg) afforded **2lh** [3.1 mg, 11%, 5.5:1 dr (*cis*/*trans*)] as a colorless oil. R_f = 0.38 [EtOAc:hexanes, 1:8 (v/v)]. ^{1}H NMR ($CDCl_3$, 400 MHz) Major isomer: δ 8.14 (s, 1H), 8.05 (d, *J* = 8.0 Hz, 1H), 7.79 (d, *J* = 7.8 Hz, 1H), 7.62 (t, *J* = 7.9 Hz, 1H), 7.30 − 7.24 (m, 2H), 7.18 − 7.03 (m, 2H), 5.89 − 5.80 (m, 1H5.41 (d, *J* = 17.1 Hz, 1H), 5.32 (d, *J* = 7.6 Hz, 1H), 5.21 (d, *J* = 10.1 Hz, 1H), 5.07 (q, *J* = 6.4 Hz, 1H), 1.64 (d, *J* = 6.4 Hz, 3H). ^{13}C{^{1}H} NMR ($CDCl_3$, 101 MHz) Major isomer: δ 140.4, 140.3, 138.8, 137.3, 131.6 (q, $J_{C\text{–}F}$ = 33.4 Hz), 130.5, 129.8, 129.3 (q, $J_{C\text{–}F}$ = 3.5 Hz), 128.4, 128.0, 124.6 (q, $J_{C\text{–}F}$ = 3.7 Hz), 123.4, 123.2 (q, $J_{C\text{–}F}$ = 272.8 Hz), 122.3, 116.8, 68.4, 62.2, 24.9. ^{19}F NMR ($CDCl_3$, 376 MHz) δ –62.85 (s), δ –62.86 (s). HRMS (ESI) m/z: $[M + H]^+$ Calcd for $C_{18}H_{17}F_3NO_2S$ 368.0927; Found 368.0926. HPLC analysis: Daicel Chiralcel OD−H (absorbance at 210 nm, mobile phase: *n*-hexane/ isopropanol = 99.4:0.6, flow rate 1.0 mL/min), er of *cis*-**2lh** = 90.0:10.0 [tr (major, 75.7%) = 14.5 min, tr (minor, 8.4%) = 15.5 min], er of *trans*-**2lh** = 65.6:34.4 [tr (major, 10.5%) = 13.3 min, tr (minor, 5.5%) = 17.8 min]. $[\alpha]_D^{25}$= +161.3 (c 0.001, CH_3OH).

5. Determination of the Absolute Configuration of Chiral Compounds in the KR Reactions

The absolute configuration of (*R*)-**1aa** was confirmed by comparing its HPLC profile with that of the authentic (*R*)-**1aa**, which was synthesized from (*S*s,*R*)-**8** using a procedure reported in the literature.[3]

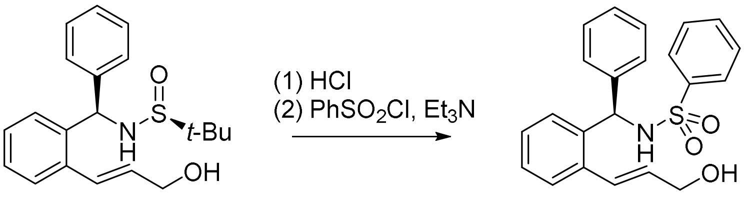

(S_s,*R*)-**8**, >20:1 dr

(*R*)-**1aa**, 98.4:1.6 er, 40% yield, over 2 steps

(*R*,*E*)-*N*-{[2-(3-Hydroxyprop-1-en-1-yl)phenyl](phenyl)methyl}benzenesulfonamide [(*R*)-**1aa**]

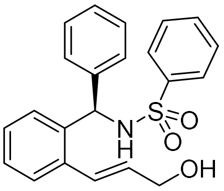

The ^{1}H NMR is identical to the one previously reported on page S4. HPLC analysis: Daicel Chiralcel OD−H (absorbance at 254 nm, mobile phase: *n*-hexane/ isopropanol = 91:9, flow rate 1.0 mL/min), er = 98.4:1.6 [tr (major, 98.4%) = 24.4 min, tr (minor, 1.6%) = 37.7 min].

The absolute configurations of *cis*-(1*S*,3*R*)-isomers in **2aa**, **2ih**, and **2lh** were confirmed by comparing their NMR spectra and HPLC profiles with those of the authentic (1*S*,3*R*)-**2aa**, **2ih**, and **2lh**. These authentic compounds were synthesized from the configurationally known compounds (*R*s,1*S*,3*R*)-**9a**, **9b**, and **9c**, respectively, using a procedure reported in the literature.[3]

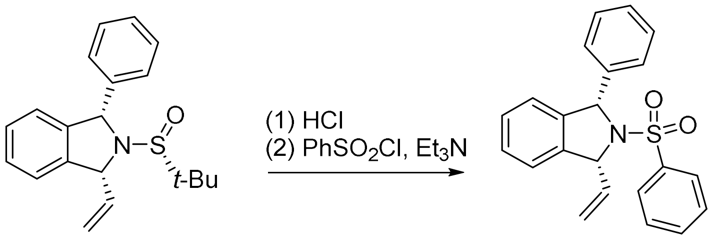

(R_s,1*S*,3*R*)-**9a**, >20:1 dr

(1*S*,3*R*)-**2aa**, 99.4:0.6 er, >20:1 dr 79% yield, over 2 steps

(1*S*,3*R*)-1-Phenyl-2-(phenylsulfonyl)-3-vinylisoindoline [(1*S*,3*R*)-**2aa**]

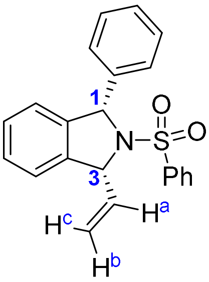

^{1}H NMR ($CDCl_3$, 400 MHz) δ 7.37 (t, *J* = 7.3 Hz, 1H), 7.29−7.27 (m, 3H), 7.23−7.18 (m, 4H), 7.16−7.10 (m, 3H), 6.98−6.97 (m, 2H), 6.90 (d, *J* = 7.5 Hz, 1H), 6.12 (d, *J* = 2.5 Hz, 1H, H^1), 6.04−5.95 (m, 1H, H^a), 5.46 (d, *J* = 17.0 Hz, 1H, H^c), 5.42 (d, *J* = 8.9 Hz, 1H, H^3), 5.31 (d, *J* = 9.9 Hz, 1H, H^b). ^{13}C{^{1}H} NMR ($CDCl_3$, 101 MHz) δ 140.6, 140.5, 140.3, 139.1, 138.1, 131.7, 128.64, 128.58, 128.3, 128.0, 127.9, 127.1, 123.39, 123.36, 116.1, 70.0, 69.1. The ^{1}H and ^{13}C NMR spectra are identical to those of the *cis*-isomer in **2aa** reported on page S10. HPLC analysis: Daicel Chiralcel IC (absorbance at 220 nm, mobile phase: *n*-hexane/ isopropanol = 92:8, flow rate 1.0 mL/min), er = 99.4:0.6 [tr (major, 99.4%) = 32.3 min, tr (minor, 0.6%) = 35.6 min].

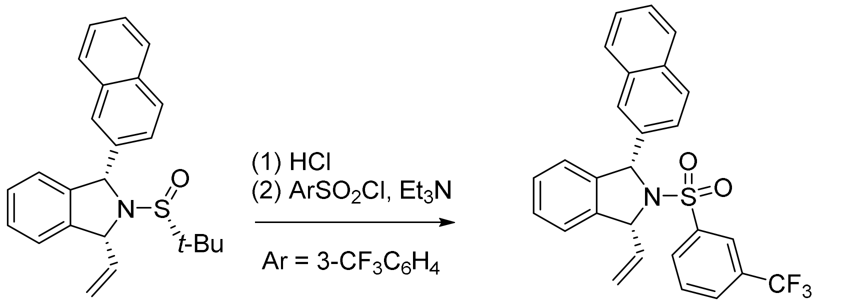

(R_s,1*S*,3*R*)-**9b**, >20:1 dr

(1*S*,3*R*)-**2ih**, >99:<1 er, >20:1 dr
85% yield, over 2 steps

(1*S*,3*R*)-1-(Naphthalen-2-yl)-2-{[3-(trifluoromethyl)phenyl]sulfonyl}-3-vinylisoindoline [(1*S*,3*R*)-**2ih**]

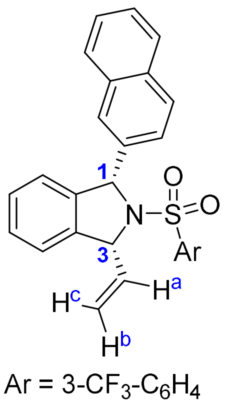

^{1}H NMR ($CDCl_3$, 400 MHz) δ 7.75 – 7.70 (m, 2H), 7.68 (s, 1H), 7.55 (s, 1H), 7.49 – 7.40 (m, 4H), 7.36 – 7.30 (m, 2H), 7.23 – 7.19 (m, 2H), 7.06 (t, *J* = 7.9 Hz, 1H), 6.89 (d, *J* = 7.7 Hz, 1H), 6.73 (d, *J* = 8.5 Hz, 1H), 6.27 (d, *J* = 2.1 Hz, 1H, H^1), 6.12 – 6.03 (m, 1H, H^a), 5.56 (dd, *J* = 8.4, 1.1 Hz, 1H, H^3), 5.52 (d, *J* = 17.5 Hz, 1H, H^c), 5.36 (d, *J* = 10.0 Hz, 1H, H^b). The ^{1}H NMR spectrum is identical to that of the *cis*-isomer in **2ih** reported on page S25. HPLC analysis: Daicel Chiralcel AD–H (absorbance at 220 nm, mobile phase: *n*-hexane/ isopropanol = 98:2, flow rate 1.0 mL/min), er = > 99.9:< 0.1 [tr (major, 99.3%) = 39.7 min].

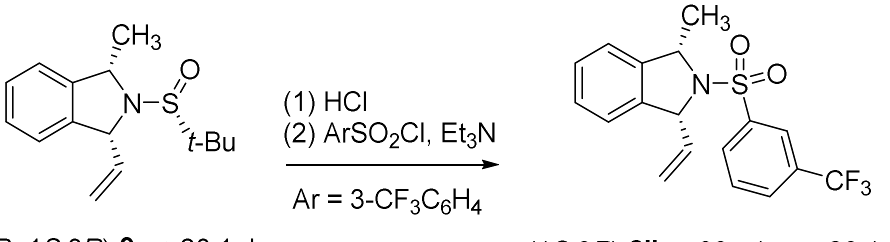

(R_s,1*S*,3*R*)-**9c**, >20:1 dr

(1*S*,3*R*)-**2lh**, >99:<1 er, >20:1 dr
57% yield, over 2 steps

(1*S*,3*R*)-1-Methyl-2-{[3-(trifluoromethyl)phenyl]sulfonyl}-3-vinylisoindoline [(1*S*,3*R*)-**2lh**]

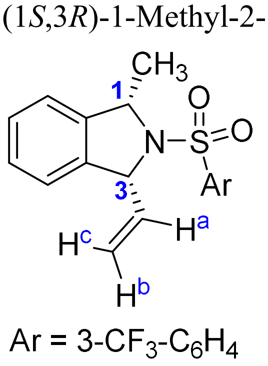

^{1}H NMR ($CDCl_3$, 400 MHz) δ 8.14 (s, 1H), 8.05 (d, *J* = 7.8 Hz, 1H), 7.79 (d, *J* = 7.9 Hz, 1H), 7.62 (t, *J* = 7.8 Hz, 1H), 7.30 – 7.27 (m, 2H), 7.15 – 7.09 (m, 2H), 5.89 – 5.80 (m, 1H, H^a), 5.41 (d, *J* = 16.9 Hz, 1H, H^c), 5.32 (d, *J* = 7.6 Hz, 1H, H^3), 5.21 (d, *J* = 9.9 Hz, 1H, H^b), 5.08 (q, *J* = 6.5 Hz, 1H, H^1), 1.63 (d, *J* = 6.4 Hz, 3H). The ^{1}H NMR spectrum is identical to that of the *cis*-isomer in **2lh** reported on page S28. HPLC analysis: Daicel Chiralcel OD-H (absorbance at 220 nm, mobile phase: *n*-hexane/ isopropanol = 99.4:0.6, flow rate 1.0 mL/min), er = >99.9:<0.1 [tr (major, 99.9%) = 15.2 min].

6. Stereodivergent Synthesis of DSIs

6.1 Synthesis of (1*R*,3*R*)-2ih through cyclization reaction using (*S*)-CPA1 as the BA catalyst

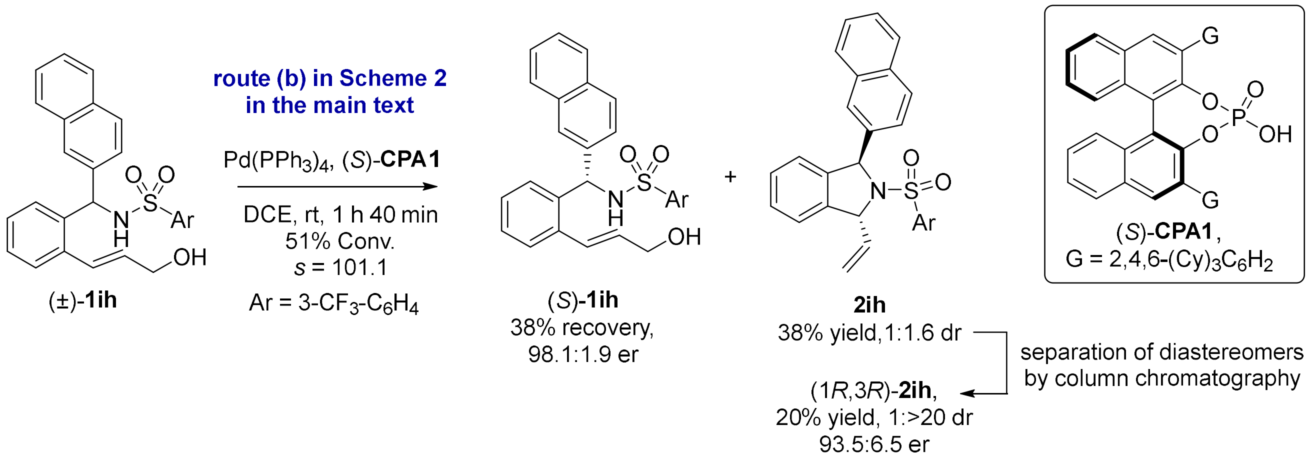

(*S*,*E*)-*N*-{[2-(3-hydroxyprop-1-en-1-yl)phenyl](naphthalen-2-yl)methyl}-3-(trifluoromethyl)benzenesulfonamide [(*S*)-**1ih**]

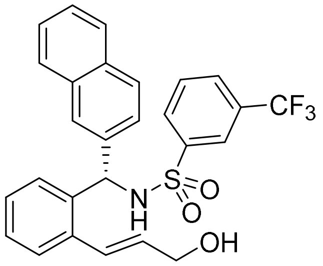

Following the adapted general procedure C with (*S*)-**CPA1** as the catalyst, the reaction with (±)-**1ih** (30.0 mg) resulted in the recovery of (*S*)-**1ih** (11.4 mg, 38%). HPLC analysis: Daicel Chiralcel AD−H (absorbance at 254 nm, mobile phase: *n*-hexane/ isopropanol = 88:12, flow rate 1.0 mL/min), er = 98.1:1.9 [tr (major, 98.1%) = 17.9 min, tr (minor, 1.9%) = 20.9 min].

(1*R*,3*R*)-1-(Naphthalen-2-yl)-2-{[3-(trifluoromethyl)phenyl]sulfonyl}-3-vinylisoindoline [(1*R*,3*R*)-**2ih**]

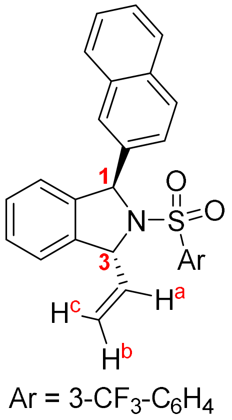

Following the adapted general procedure C with (*S*)-**CPA1** as the catalyst, the reaction of (±)-**1ih** (30.0 mg) afforded **2ih** [11.1 mg, 38%, 1:1.6 dr (*cis*/*trans*)]. The diastereomers in **2ih** were further separated by column chromatography to afford (1*R*,3*R*)-**2ih** (5.8 mg, 20%). R_f = 0.65 [EtOAc:hexanes, 1:8 (v/v)]. ^{1}H NMR ($CDCl_3$, 400 MHz) δ 7.80−7.74 (m, 3H), 7.67 (s, 1H), 7.58 (d, *J* = 8.5 Hz, 1H), 7.52−7.46 (m, 4H), 7.32 (t, *J* = 7.6 Hz, 1H), 7.24−7.19 (m, 2H), 7.15 (t, *J* = 7.8 Hz, 1H), 6.98 (dd, *J* = 8.5, 1.6 Hz, 1H), 6.91 (d, *J* = 7.6 Hz, 1H), 6.35 (s, 1H, H^1), 5.90−5.79 (m, 2H, H^a and H^3), 5.61 (d, *J* = 15.9 Hz, 1H, H^c), 5.36 (d, *J* = 10.0 Hz, 1H, H^b). ^{13}C{^{1}H} NMR ($CDCl_3$, 101 MHz) δ 141.8, 139.4, 137.7, 137.6, 137.5, 133.0, 132.9, 130.8 (q, $J_{C\text{–}F}$ = 33.4 Hz), 130.4, 128.8, 128.71, 128.66, 128.5 (q, $J_{C\text{–}F}$ = 3.3 Hz), 128.4, 128.0, 127.9, 127.6, 126.43, 126.40, 125.0, 124.5 (q, $J_{C\text{–}F}$ = 3.7 Hz), 123.7, 123.3, 122.9 (q, $J_{C\text{–}F}$ = 274.0 Hz), 118.9, 69.8, 68.8. ^{19}F NMR ($CDCl_3$, 376 MHz) δ –63.18 (s). The ^{1}H, ^{13}C, ^{19}F NMR spectra are identical to those of the *trans*-isomer in **2ih** reported on page S25. HPLC analysis: Daicel Chiralcel AD−H (absorbance at 220 nm, mobile phase: *n*-hexane/ isopropanol = 98:2, flow rate 1.0 mL/min), er = 93.5:6.5 [tr (minor, 6.5%) = 30.5 min, tr (major, 93.5%) = 48.0 min]. $[\alpha]_D^{25}$ = +250.0 (c 0.001, $CHCl_3$).

6.2 Synthesis of (1*R*,3*S*)-2ih through cyclization reaction using DPP as the BA catalyst

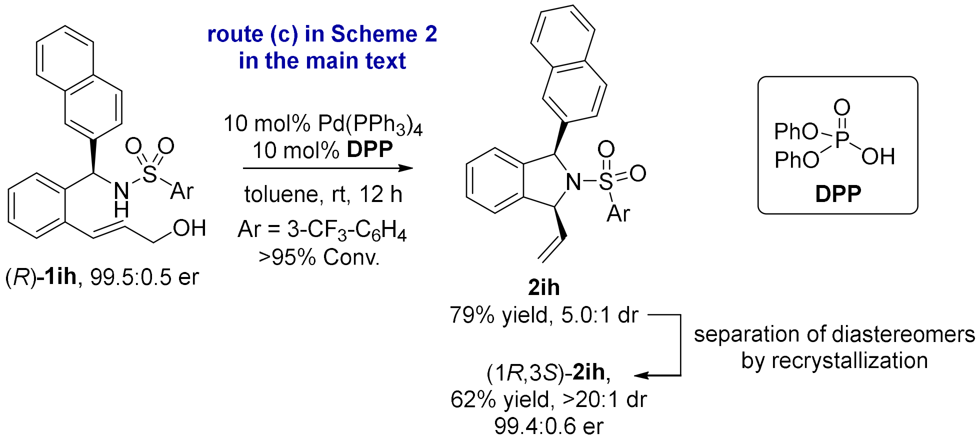

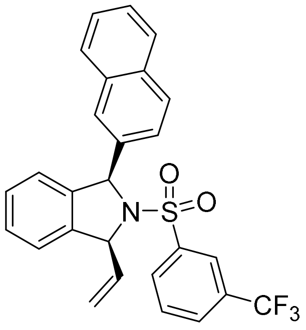

Following the adapted general procedure C with **DPP** as the catalyst, the reaction of (*R*)-**1ih** (11.5 mg) afforded **2ih** [8.8 mg, 79%, 5.0:1 dr (*cis*/*trans*)]. The diastereomers in **2ih** were further separated by recrystallization to afford (1*R*,3*S*)-**2ih** (6.9 mg, 62%). R_f = 0.61 [EtOAc:hexanes, 1:8 (v/v)]. ^{1}H NMR ($CDCl_3$, 400 MHz) δ 7.75—7.70 (m, 2H), 7.68 (s, 1H), 7.54 (s, 1H), 7.50—7.44 (m, 3H), 7.41 (d, *J* = 8.5 Hz, 1H), 7.36—7.30 (m, 2H), 7.23—7.19 (m, 2H), 7.06 (t, *J* = 7.8 Hz, 1H), 6.89 (d, *J* = 7.8 Hz, 1H), 6.72 (d, *J* = 8.4 Hz, 1H), 6.27 (d, *J* = 2.4 Hz, 1H), 6.12—6.03 (m, 1H), 5.55 (dd, *J* = 8.1, 1.9 Hz, 1H), 5.52 (d, *J* = 17.1 Hz, 1H), 5.36 (d, *J* = 9.9 Hz, 1H). ^{13}C{^{1}H} NMR ($CDCl_3$, 101 MHz) δ 141.4, 140.1, 138.8, 137.8, 136.8, 133.0, 132.8, 130.7 (q, $J_{C\text{–}F}$ = 33.3 Hz), 130.1, 128.8 (q, $J_{C\text{–}F}$ = 3.2 Hz), 128.3, 128.24, 128.19, 128.0, 127.6, 126.5, 126.4, 125.2, 124.1 (q, $J_{C\text{–}F}$ = 3.5 Hz), 123.6, 123.4, 122.9 (q, $J_{C\text{–}F}$ = 272.6 Hz), 116.4, 70.1, 69.7. ^{19}F NMR ($CDCl_3$, 376 MHz) δ –63.15 (s). The ^{1}H, ^{13}C, ^{19}F NMR spectra are identical to those of the *cis*-isomer in **2ih** reported on page S25. HPLC analysis: Daicel Chiralcel AD−H (absorbance at 220 nm, mobile phase: *n*-hexane/ isopropanol = 98:2, flow rate 1.0 mL/min), er = 99.4: 0.6 [tr (major, 97.7%) = 22.1 min, tr (minor, 0.6%) = 40.5 min].

6.3 Synthesis of (1*S*,3*R*)-2ih through cyclization reaction using DPP as the BA catalyst

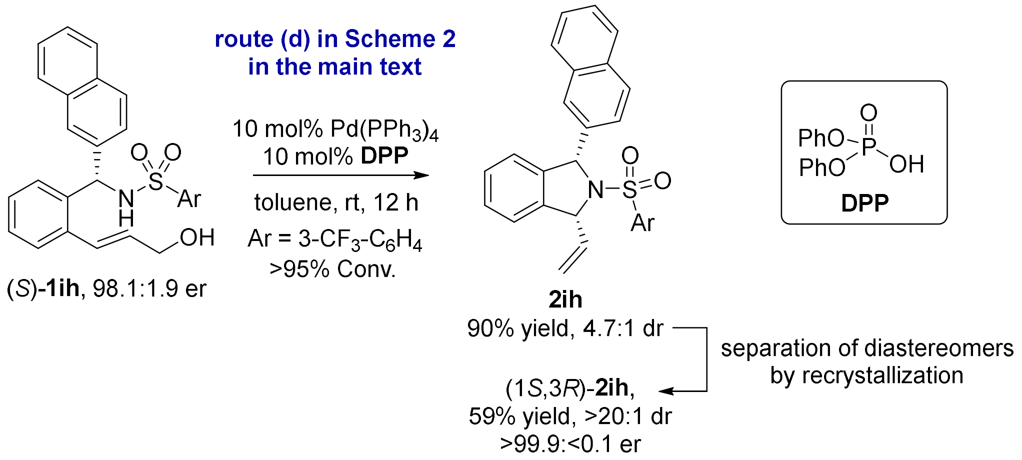

(1*S*,3*R*)-1-(Naphthalen-2-yl)-2-{[3-(trifluoromethyl)phenyl]sulfonyl}-3-vinylisoindoline [(1*S*,3*R*)-**2ih**]

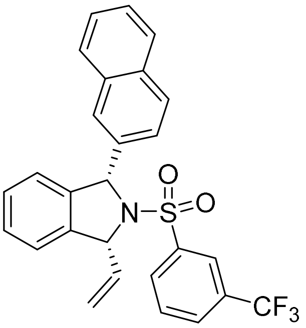

Following the adapted general procedure C with **DPP** as the catalyst, the reaction of (*S*)-**1ih** (13.3 mg) afforded **2ih** [11.5 mg, 90%, 4.7:1 dr (*cis*/*trans*)]. The diastereomers in **2ih** were further separated by recrystallization to afford (1*S*,3*R*)-**2ih** (7.2 mg, 59%). The ^{1}H NMR, ^{13}C NMR, and ^{19}F are identical with the those of (1*R*,3*S*)-isomer reported above. HPLC analysis: Daicel Chiralcel AD−H (absorbance at 220 nm, mobile phase: *n*-hexane/ isopropanol = 98:2, flow rate 1.0 mL/min), er = >99.9: <0.1 [tr (major, 99.7%) = 40.3 min].

7. References

(1) Chou, C.-T.; Lu, C.-W.; Wu, B.-S.; Tsai, C.-C. Synergistic Palladium/Chiral Phosphoric Acid−Catalyzed Kinetic Resolution via Stereoselective Intramolecular Substitution of Unactivated Allylic Alcohols. *J. Org. Chem.* **2023**, *88*, 5813−5826.

(2) Dethe, D. H.; Murhade, G. $FeCl_3$ Catalyzed Prins-Type Cyclization for the Synthesis of Highly Substituted Indenes: Application to the Total Synthesis of (±)-Jungianol and Epi-Jungianol. *Org. Lett.* **2013**, *15*, 429– 431.

(3) Hung, C.-T.; Lu, C.-W.; Huang, S.-H.; Lu, Y.-F.; Chou, H.- C.; Tsai, C.-C. Palladium/Bronsted-Acid-Catalyzed Diastereose-lective Cyclization with Chiral Sulfinamides as Nucleophiles. *J. Org. Chem*. **2021**, *86*, 12354−12366.

(4) Hemric, B. N.; Chen, A. W.; Wang, Q. Copper-Catalyzed Modular Amino Oxygenation of Alkenes: Access to Diverse 1,2-Amino Oxygen-Containing Skeletons. *J. Org. Chem.* **2019**, *84*, 1468– 1488.

8. X-Ray Crystallographic Data of Compound *trans*-2aa

Recrystallization. Compound **2aa** was dissolved in CH_2Cl_2 and *n*-hexane was added, leaving to slow evaporation overnight to yield colorless parallelepiped crystals. The enantiomeric ratio (er) of *trans*-**2aa** crystals was determined to be 98:2 based on HPLC analysis using a Daicel Chiralcel IC column. A suitable crystal (0.690 x 0.070 x 0.020 mm^3) was selected and mounted on a Gemini A Ultra diffractometer with Mo radiation (l = 0.71073 Å) for cell determination and subsequent data collection at 150 K. Using Olex29, the structure was solved with the SHELXL-2018/3 structure solution program using Intrinsic Phasing and refined with the SHELXL-2018/3 refinement package using Least Squares minimisation. The solved structure of compound *trans*-**2aa** has been deposited in The Cambridge Crystallographic Data Centre (CCDC: 2418106).

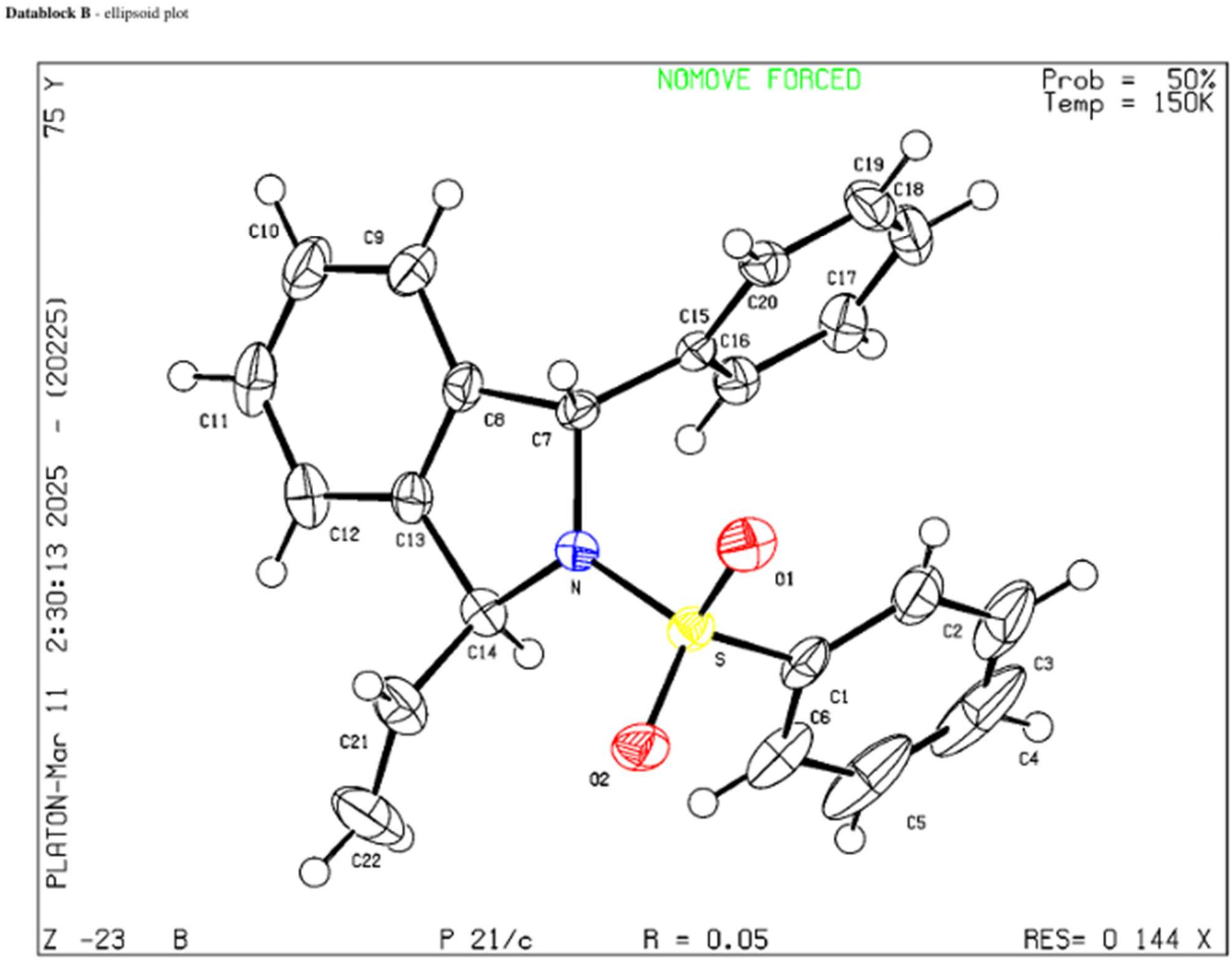

ORTEP drawing of *trans*-**2aa** showing thermal ellipsoids at the 50% probability level

Table S3. Crystal data and structure refinement for *trans*-**2aa**

Identification code	**B**	
Empirical formula	C22 H19 N O2 S	
Formula weight	361.44	
Temperature	150(2) K	
Wavelength	0.71073 Å	
Crystal system	Monoclinic	
Space group	$P2_1/c$	
Unit cell dimensions	a = 5.7255(2) Å	α= 90°.
	b = 27.5959(13) Å	β= 103.518(2)°.
	c = 12.0695(5) Å	γ = 90°.
Volume	1854.15(13) $Å^3$	
Z	4	
Density (calculated)	1.295 Mg/m^3	
Absorption coefficient	0.190 mm^{-1}	
F(000)	760	
Crystal size	0.690 x 0.070 x 0.020 mm^3	
Theta range for data collection	2.814 to 27.919°.	
Index ranges	-6<=h<=7, -36<=k<=36, -15<=l<=15	
Reflections collected	40267	
Independent reflections	4417 [R(int) = 0.0551]	
Completeness to theta = 25.242°	99.5 %	
Absorption correction	Semi-empirical from equivalents	
Max. and min. transmission	0.7456 and 0.6784	
Refinement method	Full-matrix least-squares on F^2	
Data / restraints / parameters	4417 / 0 / 235	
Goodness-of-fit on F^2	1.092	
Final R indices [I>2sigma(I)]	R1 = 0.0483, wR2 = 0.1149	
R indices (all data)	R1 = 0.0665, wR2 = 0.1267	
Extinction coefficient	n/a	
Largest diff. peak and hole	0.456 and -0.321 e.$Å^{-3}$	

Table S4. Atomic coordinates (x 10^4) and equivalent isotropic displacement parameters ($Å^2$x 10^3) for *trans*-**2aa**. U(eq) is defined as one third of the trace of the orthogonalized U^{ij} tensor.

	x	y	z	U(eq)
S	8874(1)	6497(1)	1735(1)	23(1)
O(1)	11238(2)	6456(1)	2448(1)	34(1)
O(2)	8572(3)	6545(1)	532(1)	35(1)
N	7440(2)	6016(1)	1962(1)	22(1)
C(1)	7477(3)	7002(1)	2200(2)	30(1)
C(2)	8595(5)	7226(1)	3211(2)	50(1)
C(3)	7407(8)	7615(1)	3582(3)	82(1)
C(4)	5178(8)	7765(1)	2953(4)	92(1)
C(5)	4120(5)	7538(1)	1957(3)	70(1)
C(6)	5261(4)	7156(1)	1557(2)	43(1)
C(7)	8155(3)	5742(1)	3053(1)	21(1)
C(8)	6162(3)	5374(1)	2884(1)	24(1)
C(9)	5936(4)	4997(1)	3616(2)	35(1)
C(10)	3917(4)	4702(1)	3329(2)	42(1)
C(11)	2172(4)	4784(1)	2342(2)	41(1)
C(12)	2392(3)	5164(1)	1615(2)	34(1)
C(13)	4424(3)	5457(1)	1903(1)	25(1)
C(14)	5028(3)	5889(1)	1255(1)	25(1)
C(15)	8349(3)	6038(1)	4125(1)	20(1)
C(16)	6391(3)	6298(1)	4316(1)	24(1)
C(17)	6595(3)	6557(1)	5314(2)	31(1)
C(18)	8726(4)	6555(1)	6142(2)	36(1)
C(19)	10677(3)	6296(1)	5960(2)	34(1)
C(20)	10484(3)	6041(1)	4953(1)	26(1)
C(21)	5036(4)	5766(1)	48(2)	38(1)
C(22)	3340(5)	5912(1)	-825(2)	58(1)

Table S5. Bond lengths [Å] and angles [°] for *trans*-**2aa**.

S-O(2)	1.4281(13)
S-O(1)	1.4286(13)
S-N	1.6166(14)
S-C(1)	1.7615(17)
N-C(14)	1.486(2)
N-C(7)	1.4913(19)
C(1)-C(2)	1.384(3)
C(1)-C(6)	1.390(3)
C(2)-C(3)	1.398(4)
C(2)-H(2B)	0.9500
C(3)-C(4)	1.387(5)
C(3)-H(3A)	0.9500
C(4)-C(5)	1.365(5)
C(4)-H(4A)	0.9500
C(5)-C(6)	1.385(3)
C(5)-H(5A)	0.9500
C(6)-H(6A)	0.9500
C(7)-C(8)	1.506(2)
C(7)-C(15)	1.512(2)
C(7)-H(7A)	1.0000
C(8)-C(13)	1.377(2)
C(8)-C(9)	1.389(2)
C(9)-C(10)	1.389(3)
C(9)-H(9A)	0.9500
C(10)-C(11)	1.382(3)
C(10)-H(10A)	0.9500
C(11)-C(12)	1.391(3)
C(11)-H(11A)	0.9500
C(12)-C(13)	1.393(2)
C(12)-H(12A)	0.9500
C(13)-C(14)	1.510(2)
C(14)-C(21)	1.496(2)
C(14)-H(14A)	1.0000
C(15)-C(20)	1.386(2)
C(15)-C(16)	1.395(2)
C(16)-C(17)	1.383(2)

C(16)-H(16A)	0.9500
C(17)-C(18)	1.385(3)
C(17)-H(17A)	0.9500
C(18)-C(19)	1.386(3)
C(18)-H(18A)	0.9500
C(19)-C(20)	1.387(3)
C(19)-H(19A)	0.9500
C(20)-H(20A)	0.9500
C(21)-C(22)	1.318(3)
C(21)-H(21A)	0.9500
C(22)-H(22C)	0.9500
C(22)-H(22A)	0.9500

O(2)-S-O(1)	119.45(8)
O(2)-S-N	107.62(7)
O(1)-S-N	106.23(7)
O(2)-S-C(1)	107.12(8)
O(1)-S-C(1)	107.97(9)
N-S-C(1)	107.99(7)
C(14)-N-C(7)	114.44(12)
C(14)-N-S	122.47(11)
C(7)-N-S	121.40(10)
C(2)-C(1)-C(6)	121.9(2)
C(2)-C(1)-S	118.90(17)
C(6)-C(1)-S	119.13(15)
C(1)-C(2)-C(3)	117.8(3)
C(1)-C(2)-H(2B)	121.1
C(3)-C(2)-H(2B)	121.1
C(4)-C(3)-C(2)	120.5(3)
C(4)-C(3)-H(3A)	119.7
C(2)-C(3)-H(3A)	119.7
C(5)-C(4)-C(3)	120.4(2)
C(5)-C(4)-H(4A)	119.8
C(3)-C(4)-H(4A)	119.8
C(4)-C(5)-C(6)	120.6(3)
C(4)-C(5)-H(5A)	119.7
C(6)-C(5)-H(5A)	119.7
C(5)-C(6)-C(1)	118.8(3)

C(5)-C(6)-H(6A)	120.6
C(1)-C(6)-H(6A)	120.6
N-C(7)-C(8)	100.41(12)
N-C(7)-C(15)	115.55(12)
C(8)-C(7)-C(15)	112.43(13)
N-C(7)-H(7A)	109.4
C(8)-C(7)-H(7A)	109.4
C(15)-C(7)-H(7A)	109.4
C(13)-C(8)-C(9)	121.10(16)
C(13)-C(8)-C(7)	112.06(14)
C(9)-C(8)-C(7)	126.80(16)
C(8)-C(9)-C(10)	118.34(18)
C(8)-C(9)-H(9A)	120.8
C(10)-C(9)-H(9A)	120.8
C(11)-C(10)-C(9)	120.74(18)
C(11)-C(10)-H(10A)	119.6
C(9)-C(10)-H(10A)	119.6
C(10)-C(11)-C(12)	120.84(17)
C(10)-C(11)-H(11A)	119.6
C(12)-C(11)-H(11A)	119.6
C(13)-C(12)-C(11)	118.28(18)
C(13)-C(12)-H(12A)	120.9
C(11)-C(12)-H(12A)	120.9
C(8)-C(13)-C(12)	120.70(17)
C(8)-C(13)-C(14)	111.79(14)
C(12)-C(13)-C(14)	127.50(16)
N-C(14)-C(21)	113.51(14)
N-C(14)-C(13)	100.57(13)
C(21)-C(14)-C(13)	112.20(14)
N-C(14)-H(14A)	110.1
C(21)-C(14)-H(14A)	110.1
C(13)-C(14)-H(14A)	110.1
C(20)-C(15)-C(16)	119.02(15)
C(20)-C(15)-C(7)	119.75(14)
C(16)-C(15)-C(7)	121.20(13)
C(17)-C(16)-C(15)	120.17(16)
C(17)-C(16)-H(16A)	119.9
C(15)-C(16)-H(16A)	119.9

C(16)-C(17)-C(18)	120.49(17)
C(16)-C(17)-H(17A)	119.8
C(18)-C(17)-H(17A)	119.8
C(17)-C(18)-C(19)	119.65(16)
C(17)-C(18)-H(18A)	120.2
C(19)-C(18)-H(18A)	120.2
C(20)-C(19)-C(18)	119.91(17)
C(20)-C(19)-H(19A)	120.0
C(18)-C(19)-H(19A)	120.0
C(15)-C(20)-C(19)	120.75(16)
C(15)-C(20)-H(20A)	119.6
C(19)-C(20)-H(20A)	119.6
C(22)-C(21)-C(14)	122.6(2)
C(22)-C(21)-H(21A)	118.7
C(14)-C(21)-H(21A)	118.7
C(21)-C(22)-H(22C)	120.0
C(21)-C(22)-H(22A)	120.0
H(22C)-C(22)-H(22A)	120.0

Symmetry transformations used to generate equivalent atoms:

9. NMR Spectra

^{1}H NMR of **1aa** ($CDCl_3$, 400 MHz)

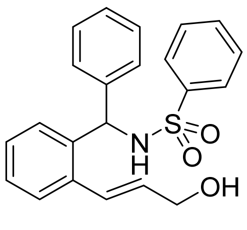

1aa

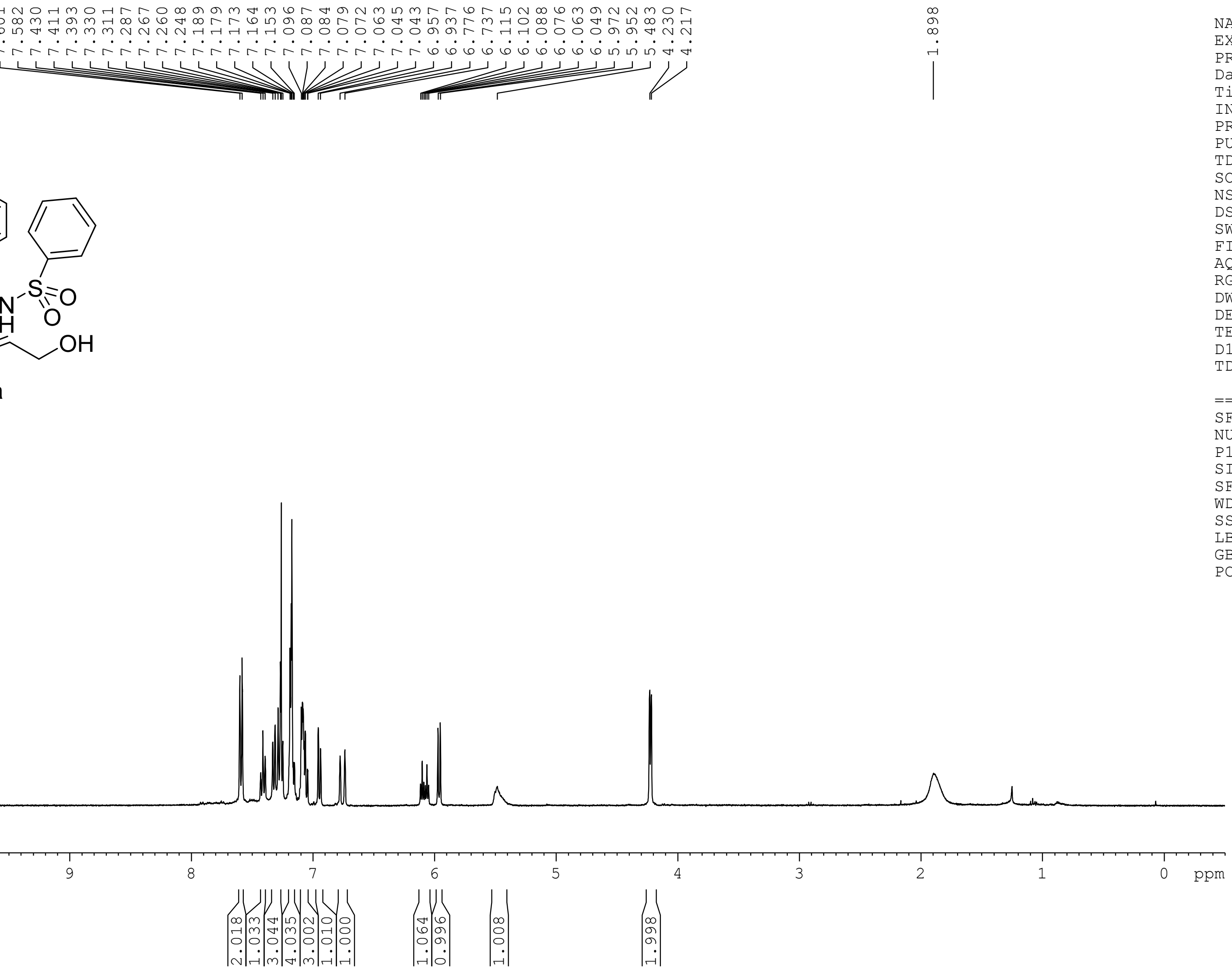

NAME	202308
EXPNO	162
PROCNO	1
Date_	20230809
Time	17.09
INSTRUM	spect
PROBHD	5 mm PABBO BB/
PULPROG	zg30
TD	32768
SOLVENT	CDCl3
NS	5
DS	0
SWH	8012.820 Hz
FIDRES	0.244532 Hz
AQ	2.0447731 sec
RG	137.93
DW	62.400 usec
DE	16.53 usec
TE	291.9 K
D1	2.00000000 sec
TD0	1
======== CHANNEL f1 ========	
SFO1	400.1324008 MHz
NUC1	1H
P1	14.00 usec
SI	16384
SF	400.1300096 MHz
WDW	EM
SSB	0
LB	0.00 Hz
GB	0
PC	1.00

$^{13}C\{^1H\}$ NMR of **1aa** ($CDCl_3$, 101 MHz)

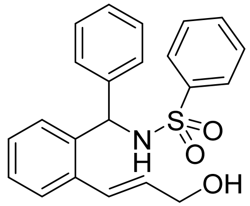

1aa

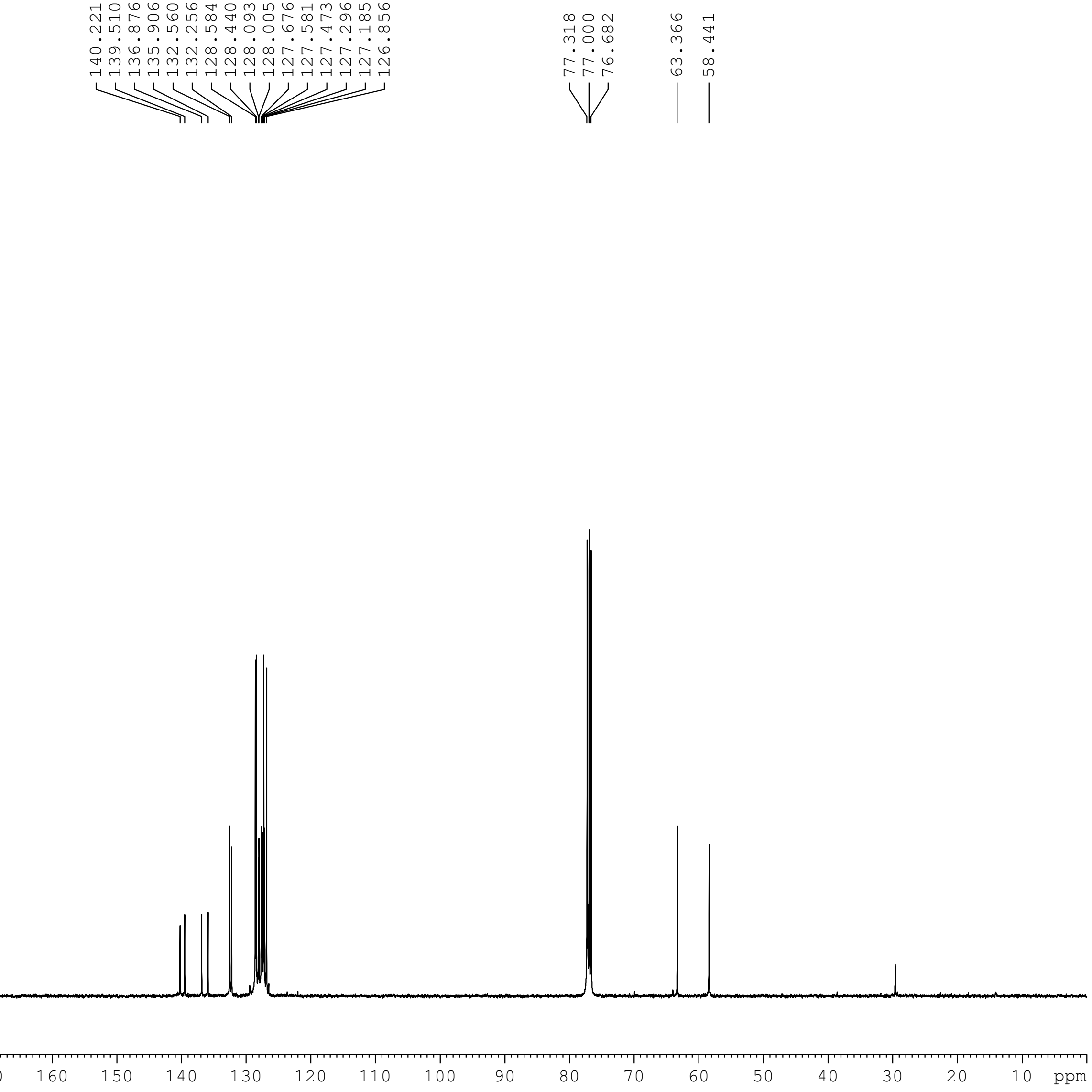


```
NAME                202307
EXPNO                  436
PROCNO                   1
Date_             20230729
Time                  0.23
INSTRUM              spect
PROBHD   5 mm PABBO BB/
PULPROG            zgpg30
TD                  32768
SOLVENT              CDCl3
NS                    4000
DS                       0
SWH             24038.461 Hz
FIDRES           0.733596 Hz
AQ              0.6816244 sec
RG                 205.92
DW                 20.800 usec
DE                   6.50 usec
TE                  291.9 K
D1             2.00000000 sec
D11            0.03000000 sec
TD0                      1

======== CHANNEL f1 ========
SFO1         100.6233329 MHz
NUC1                   13C
P1                   10.00 usec
SI                   32768
SF           100.6127757 MHz
WDW                     EM
SSB                      0
LB                    2.00 Hz
GB                       0
PC                    1.00
```

^{1}H NMR of **1ab** ($CDCl_3$, 400 MHz)

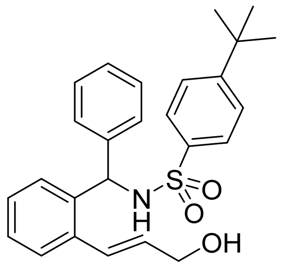

1ab

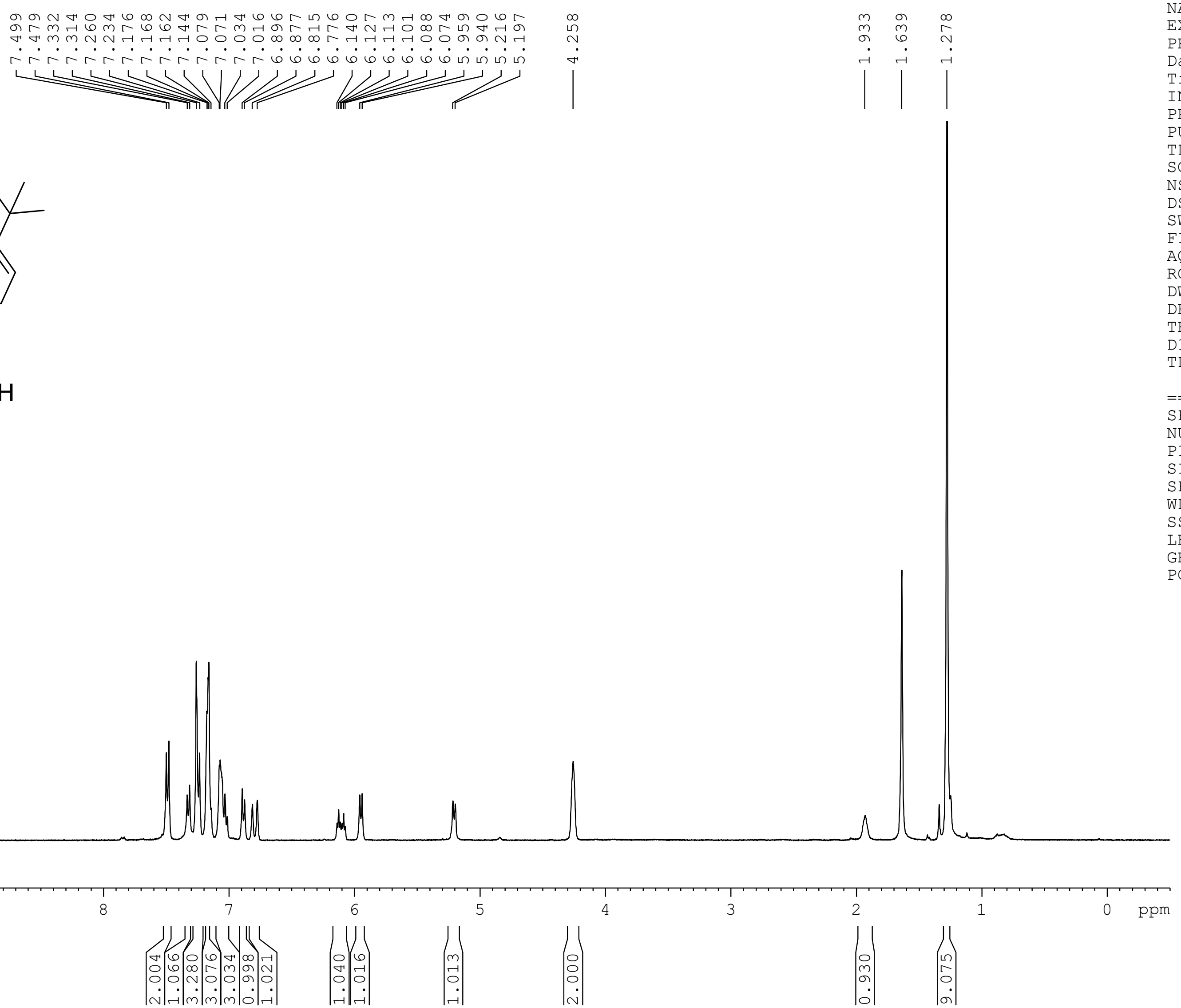

NAME 202304
EXPNO 103
PROCNO 1
Date_ 20230414
Time 19.56
INSTRUM spect
PROBHD 5 mm PABBO BB/
PULPROG zg30
TD 32768
SOLVENT CDCl3
NS 16
DS 0
SWH 8012.820 Hz
FIDRES 0.244532 Hz
AQ 2.0447731 sec
RG 205.92
DW 62.400 usec
DE 16.53 usec
TE 288.8 K
D1 2.00000000 sec
TD0 1

======== CHANNEL f1 ========
SFO1 400.1324008 MHz
NUC1 1H
P1 14.00 usec
SI 16384
SF 400.1300093 MHz
WDW EM
SSB 0
LB 0.00 Hz
GB 0
PC 1.00

$^{13}C\{^{1}H\}$ NMR of **1ab** ($CDCl_3$, 101 MHz)

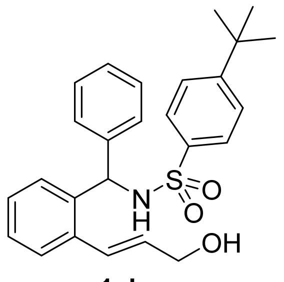

1ab

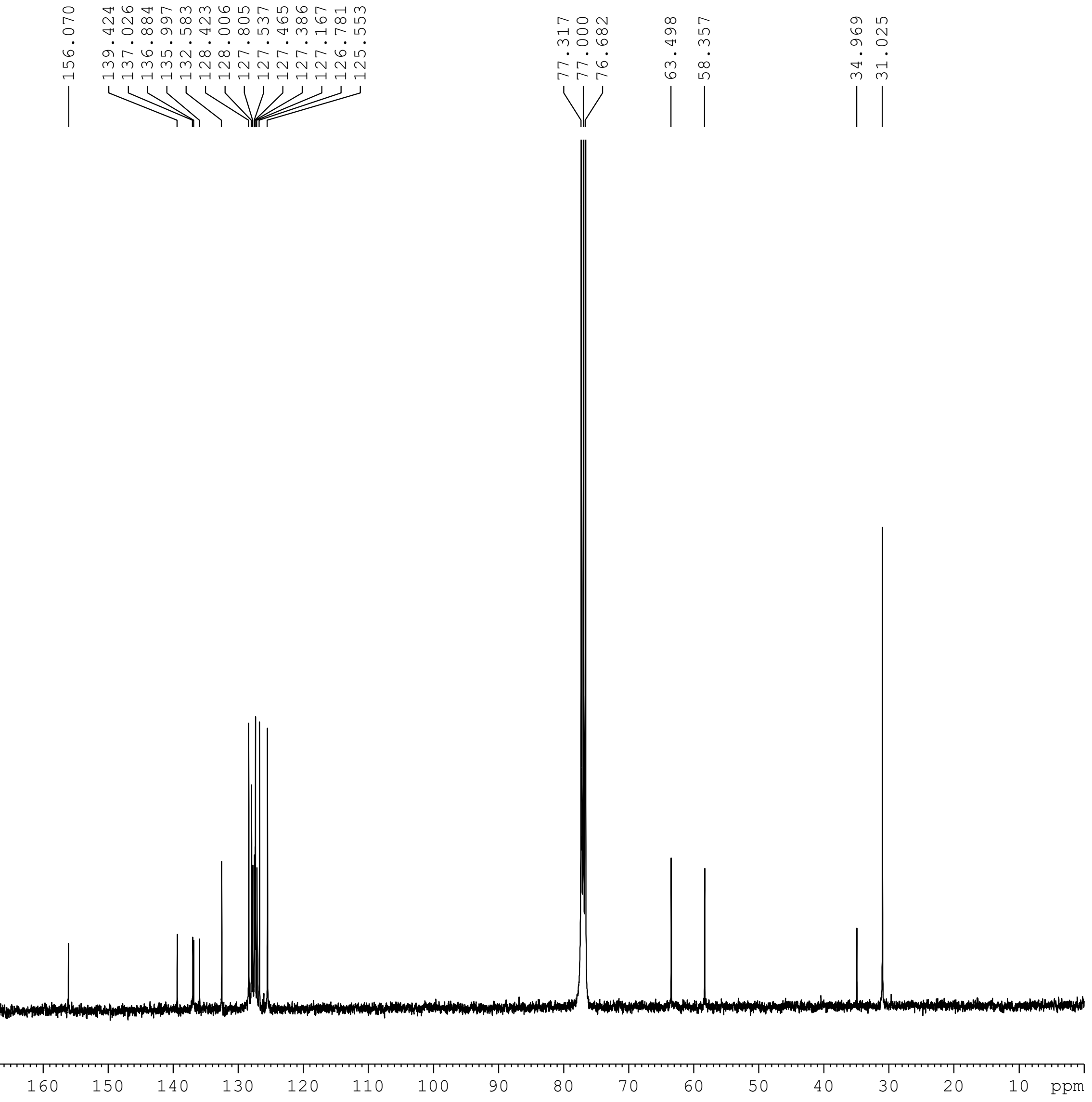

NAME	202304
EXPNO	128
PROCNO	1
Date_	20230417
Time	6.44
INSTRUM	spect
PROBHD	5 mm PABBO BB/
PULPROG	zgpg30
TD	32768
SOLVENT	CDCl3
NS	5000
DS	0
SWH	24038.461 Hz
FIDRES	0.733596 Hz
AQ	0.6816244 sec
RG	205.92
DW	20.800 usec
DE	6.50 usec
TE	290.0 K
D1	2.00000000 sec
D11	0.03000000 sec
TD0	1

======== CHANNEL f1 ========

SFO1	100.6233329 MHz
NUC1	13C
P1	10.00 usec
SI	32768
SF	100.6127732 MHz
WDW	EM
SSB	0
LB	2.00 Hz
GB	0
PC	1.00

^{1}H NMR of **1ac** ($CDCl_3$, 400 MHz)

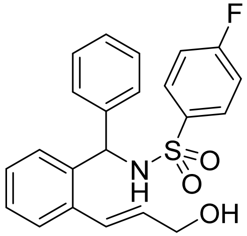

1ac

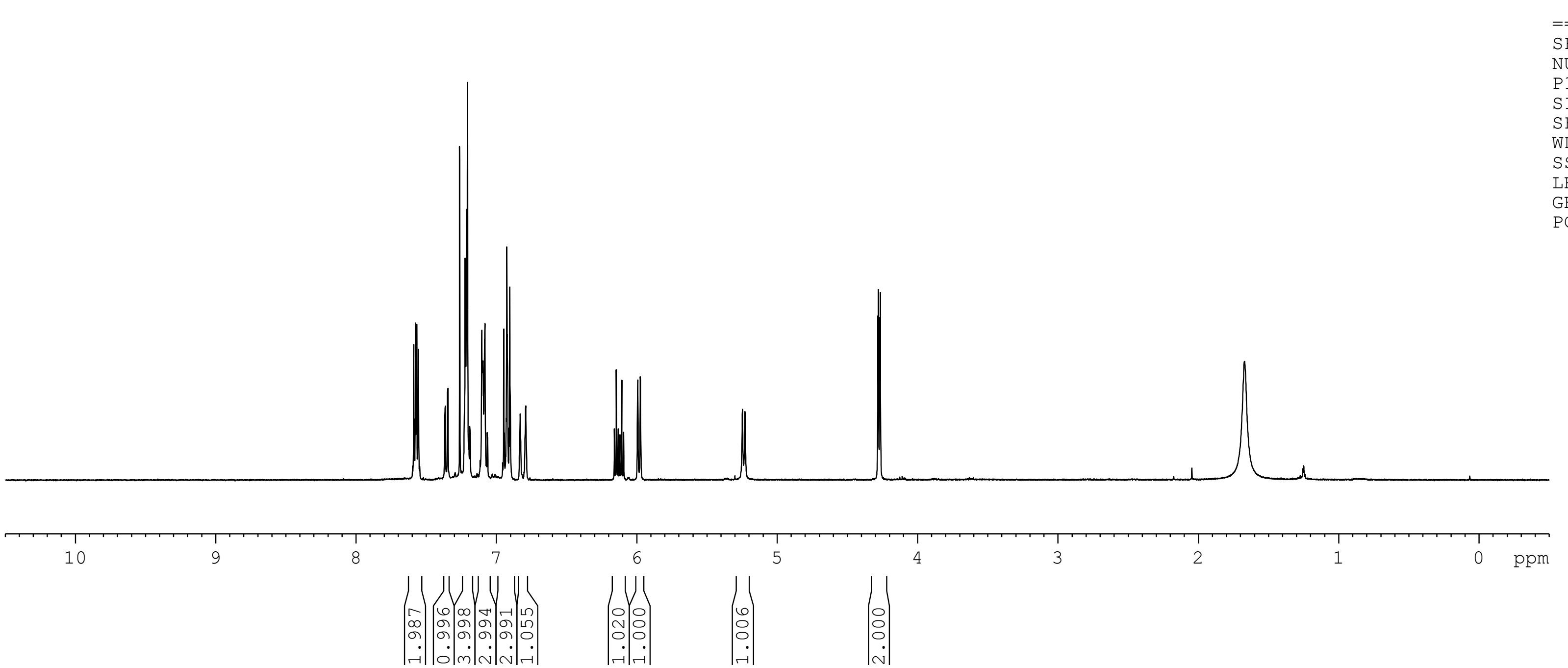

NAME	202307
EXPNO	158
PROCNO	1
Date_	20230712
Time	14.48
INSTRUM	spect
PROBHD	5 mm PABBO BB/
PULPROG	zg30
TD	32768
SOLVENT	CDCl3
NS	16
DS	0
SWH	8012.820 Hz
FIDRES	0.244532 Hz
AQ	2.0447731 sec
RG	205.92
DW	62.400 usec
DE	16.53 usec
TE	289.8 K
D1	2.00000000 sec
TD0	1

======== CHANNEL f1 ========

SFO1	400.1324008 MHz
NUC1	1H
P1	14.00 usec
SI	16384
SF	400.1300099 MHz
WDW	EM
SSB	0
LB	0.00 Hz
GB	0
PC	1.00

$^{13}C\{^1H\}$ NMR of **1ac** ($CDCl_3$, 101 MHz)

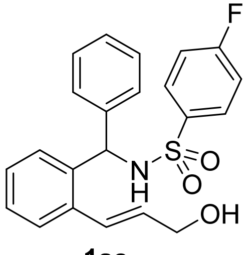

1ac

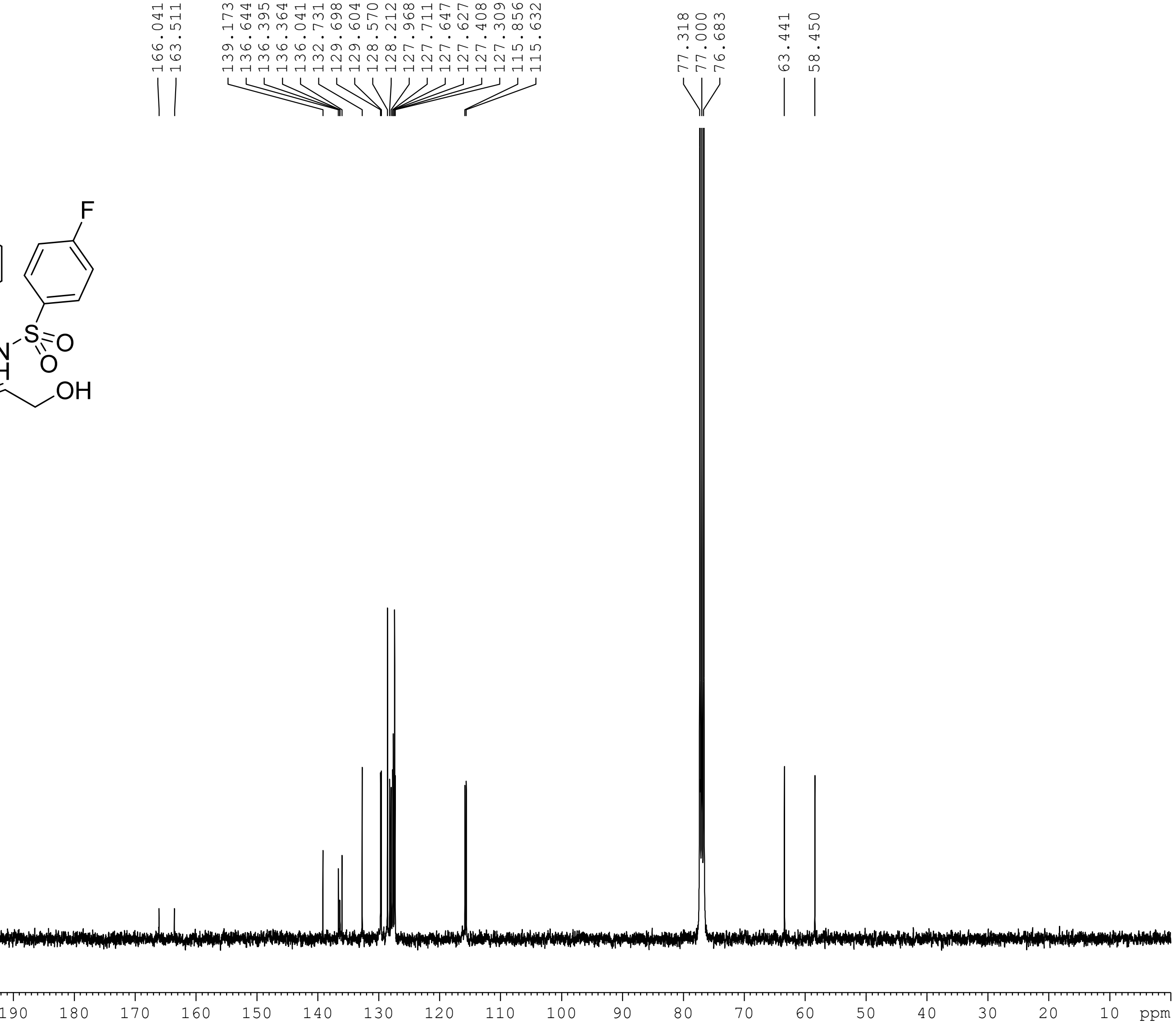

NAME	202307
EXPNO	159
PROCNO	1
Date_	20230713
Time	7.54
INSTRUM	spect
PROBHD	5 mm PABBO BB/
PULPROG	zgpg30
TD	32768
SOLVENT	CDCl3
NS	3500
DS	0
SWH	24038.461 Hz
FIDRES	0.733596 Hz
AQ	0.6816244 sec
RG	205.92
DW	20.800 usec
DE	6.50 usec
TE	289.9 K
D1	2.00000000 sec
D11	0.03000000 sec
TD0	1

======== CHANNEL f1 ========

SFO1	100.6233329 MHz
NUC1	13C
P1	10.00 usec
SI	32768
SF	100.6127722 MHz
WDW	EM
SSB	0
LB	2.00 Hz
GB	0
PC	1.00

^{19}F NMR of **1ac** ($CDCl_3$, 376 MHz)

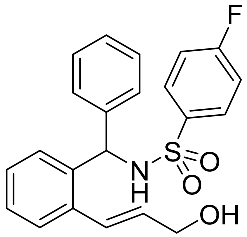

1ac

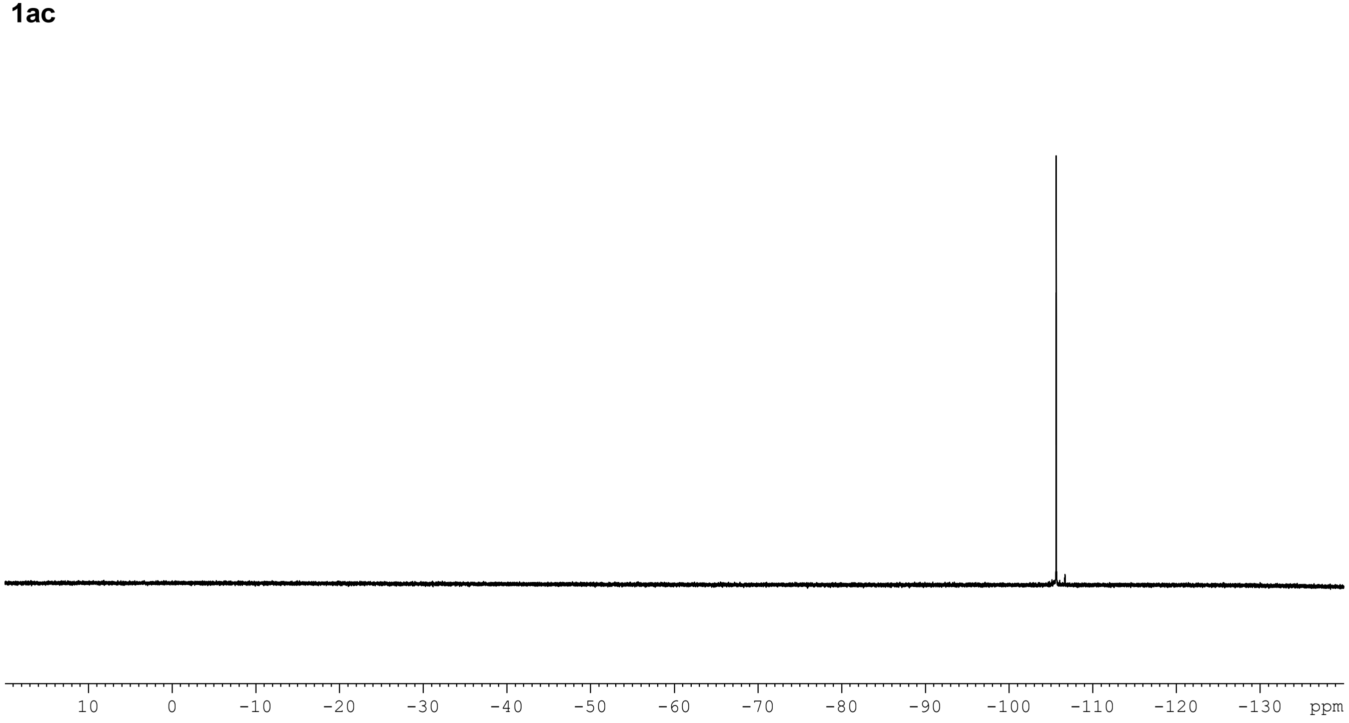

NAME	202307	
EXPNO	175	
PROCNO	1	
Date_	20230713	
Time	10.33	
INSTRUM	spect	
PROBHD	5 mm PABBO BB/	
PULPROG	zg30	
TD	131072	
SOLVENT	CDCl3	
NS	203	
DS	0	
SWH	89285.711	Hz
FIDRES	0.681196	Hz
AQ	0.7340532	sec
RG	205.92	
DW	5.600	usec
DE	6.50	usec
TE	289.7	K
D1	1.00000000	sec
TD0	1	

======== CHANNEL f1 ========

SFO1	376.4757776	MHz
NUC1	19F	
P1	15.00	usec
SI	65536	
SF	376.4983662	MHz
WDW	EM	
SSB	0	
LB	0.30	Hz
GB	0	
PC	1.00	

^{1}H NMR of **1ad** ($CDCl_3$, 400 MHz)

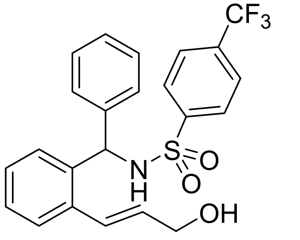

1ad

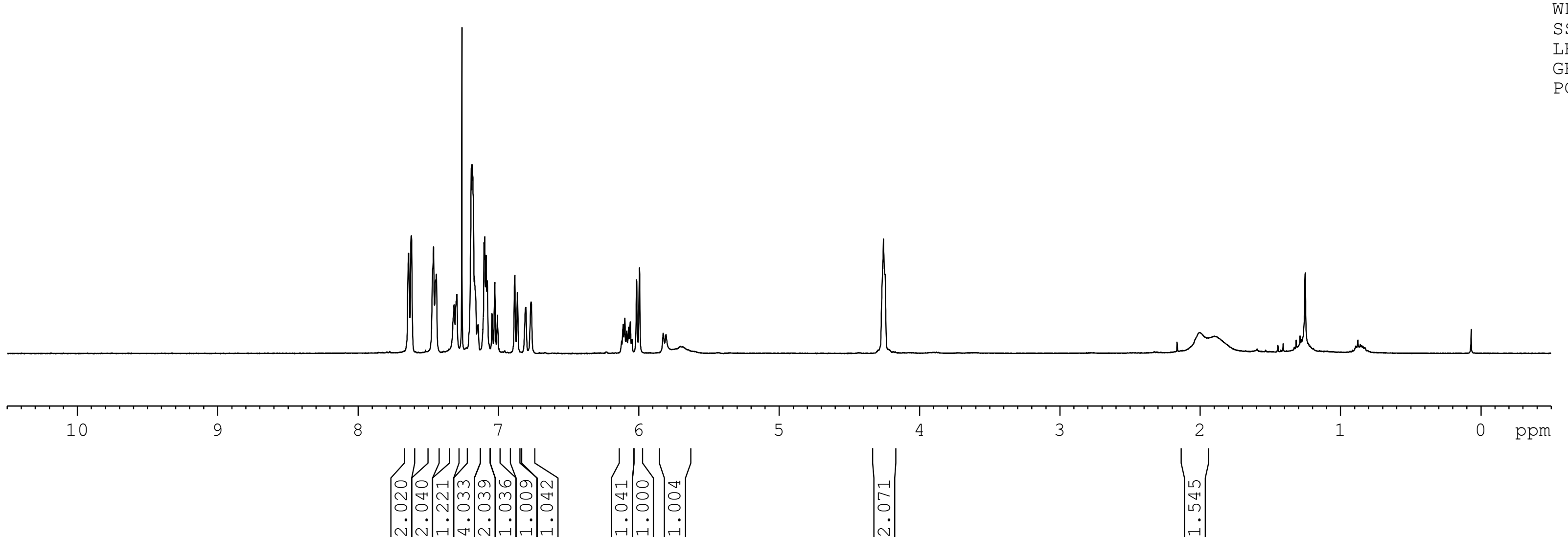

NAME	202308
EXPNO	138
PROCNO	1
Date_	20230808
Time	13.47
INSTRUM	spect
PROBHD	5 mm PABBO BB/
PULPROG	zg30
TD	32768
SOLVENT	CDCl3
NS	16
DS	0
SWH	8012.820 Hz
FIDRES	0.244532 Hz
AQ	2.0447731 sec
RG	137.93
DW	62.400 usec
DE	16.53 usec
TE	292.1 K
D1	2.00000000 sec
TD0	1

======== CHANNEL f1 ========

SFO1	400.1324008 MHz
NUC1	1H
P1	14.00 usec
SI	16384
SF	400.1300097 MHz
WDW	EM
SSB	0
LB	0.00 Hz
GB	0
PC	1.00

$^{13}C\{^1H\}$ NMR of **1ad** ($CDCl_3$, 101 MHz)

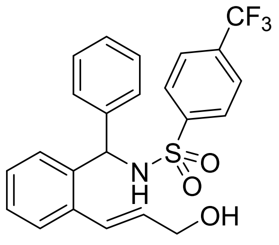

1ad

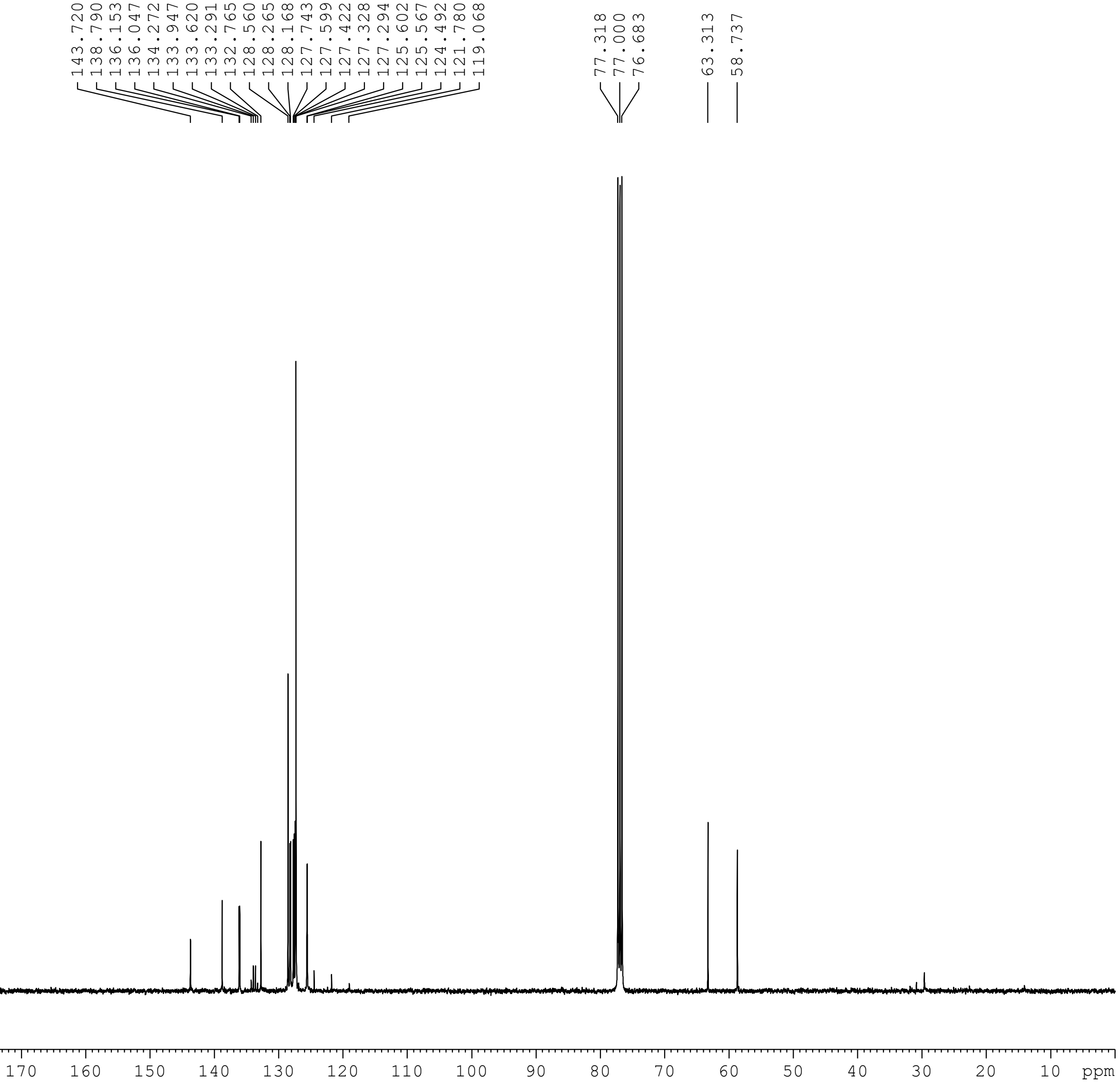

NAME	202308
EXPNO	101
PROCNO	1
Date_	20230804
Time	23.54
INSTRUM	spect
PROBHD	5 mm PABBO BB/
PULPROG	zgpg30
TD	32768
SOLVENT	CDCl3
NS	2033
DS	0
SWH	24038.461 Hz
FIDRES	0.733596 Hz
AQ	0.6816244 sec
RG	205.92
DW	20.800 usec
DE	6.50 usec
TE	293.6 K
D1	2.00000000 sec
D11	0.03000000 sec
TD0	1
======== CHANNEL f1 ========	
SFO1	100.6233329 MHz
NUC1	13C
P1	10.00 usec
SI	32768
SF	100.6127730 MHz
WDW	EM
SSB	0
LB	2.00 Hz
GB	0
PC	1.00

^{19}F NMR of **1ad** ($CDCl_3$, 376 MHz)

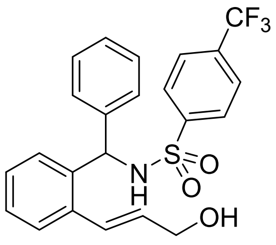

1ad

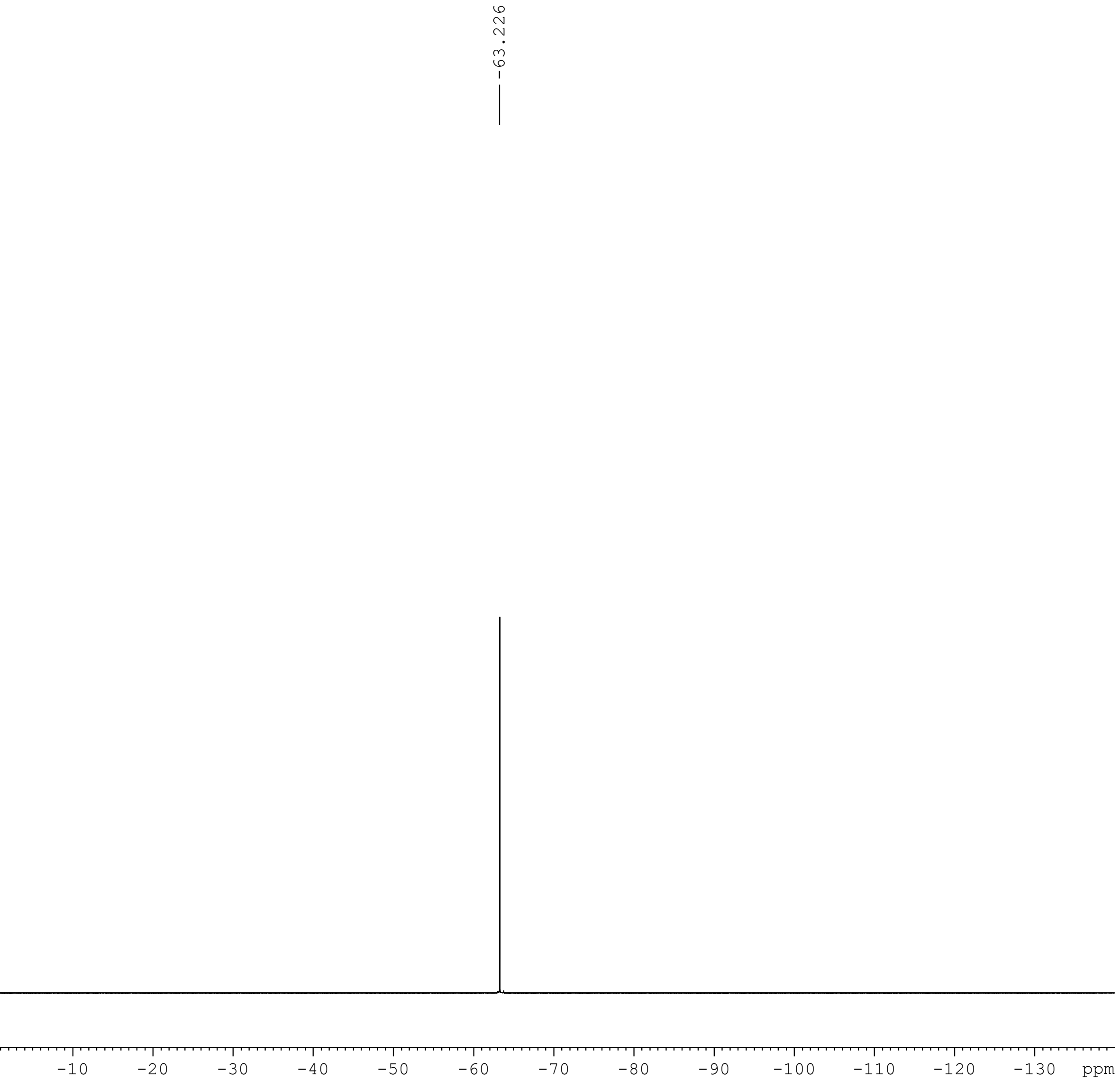


```
NAME              202308
EXPNO                 65
PROCNO                 1
Date_           20230802
Time               18.40
INSTRUM            spect
PROBHD   5 mm PABBO BB/
PULPROG           zgig30
TD                131072
SOLVENT            CDCl3
NS                    16
DS                     0
SWH            89285.711 Hz
FIDRES          0.681196 Hz
AQ             0.7340532 sec
RG                205.92
DW                 5.600 usec
DE                  6.50 usec
TE                 289.9 K
D1            1.00000000 sec
D11           0.03000000 sec
TD0                    1

======== CHANNEL f1 ========
SFO1         376.4757776 MHz
NUC1                 19F
P1                 15.00 usec
SI                 65536
SF           376.4983662 MHz
WDW                   EM
SSB                    0
LB                  0.30 Hz
GB                     0
PC                  1.00
```

^{1}H NMR of **1ae** ($CDCl_3$, 400 MHz)

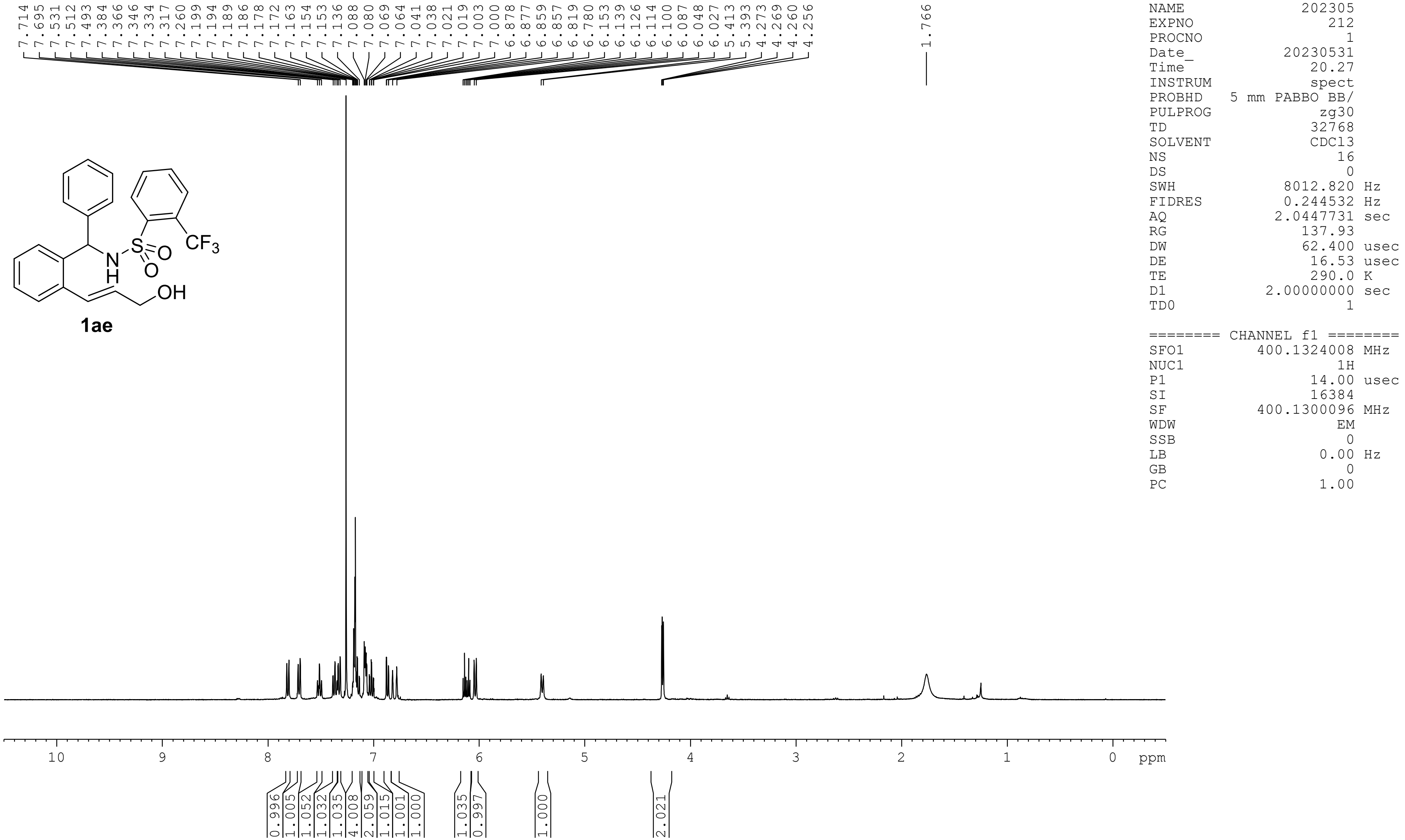

$^{13}C\{^1H\}$ NMR of **1ae** ($CDCl_3$, 101 MHz)

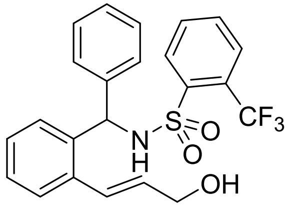

1ae

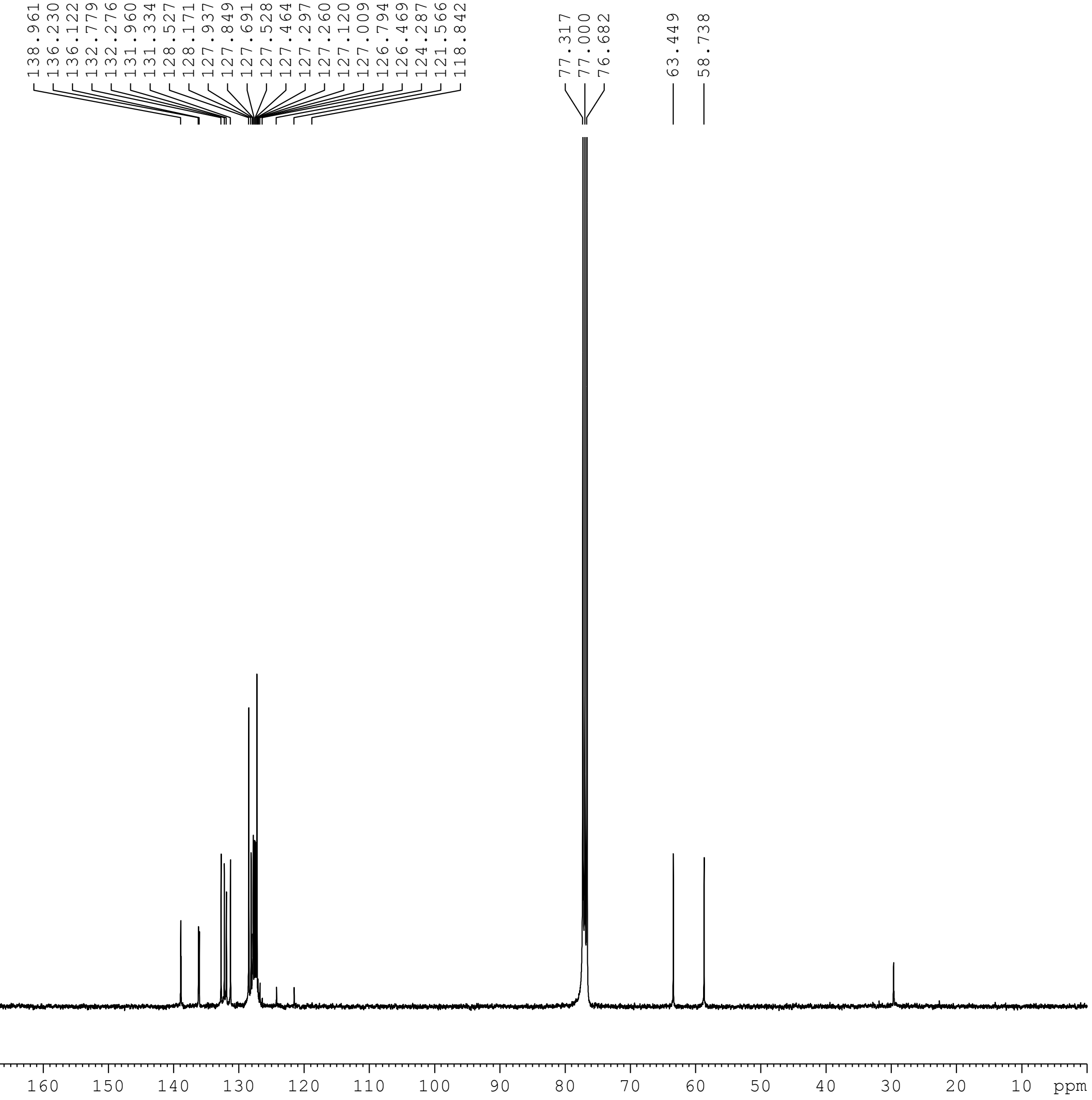

NAME	202307
EXPNO	438
PROCNO	1
Date_	20230729
Time	8.08
INSTRUM	spect
PROBHD	5 mm PABBO BB/
PULPROG	zgpg30
TD	32768
SOLVENT	CDCl3
NS	10000
DS	0
SWH	24038.461 Hz
FIDRES	0.733596 Hz
AQ	0.6816244 sec
RG	205.92
DW	20.800 usec
DE	6.50 usec
TE	291.4 K
D1	2.00000000 sec
D11	0.03000000 sec
TD0	1

======== CHANNEL f1 ========

SFO1	100.6233329 MHz
NUC1	13C
P1	10.00 usec
SI	32768
SF	100.6127732 MHz
WDW	EM
SSB	0
LB	2.00 Hz
GB	0
PC	1.00

^{19}F NMR of **1ae** ($CDCl_3$, 376 MHz)

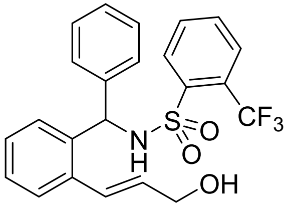

1ae

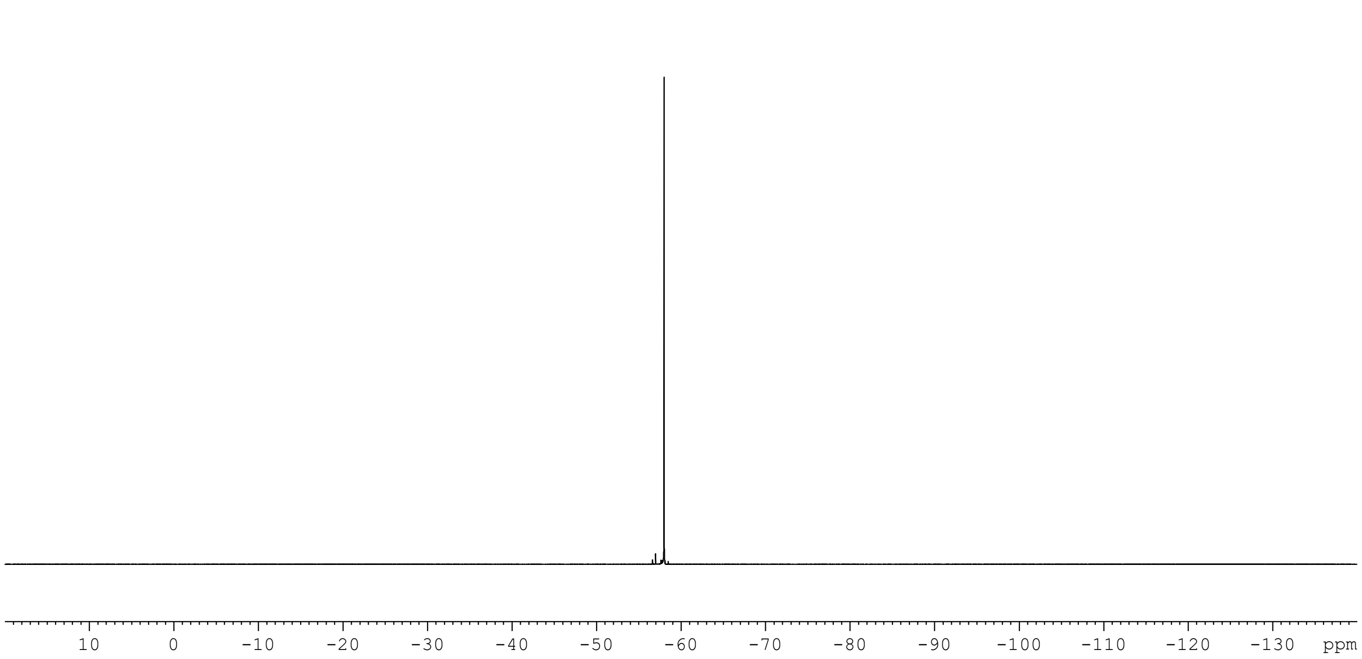


```
NAME              202307
EXPNO                174
PROCNO                 1
Date_           20230713
Time               10.26
INSTRUM            spect
PROBHD   5 mm PABBO BB/
PULPROG             zg30
TD                131072
SOLVENT            CDCl3
NS                    89
DS                     0
SWH           89285.711 Hz
FIDRES         0.681196 Hz
AQ            0.7340532 sec
RG                205.92
DW                 5.600 usec
DE                  6.50 usec
TE                 289.7 K
D1            1.00000000 sec
TD0                    1

======== CHANNEL f1 ========
SFO1        376.4757776 MHz
NUC1                 19F
P1                 15.00 usec
SI                 65536
SF          376.4983662 MHz
WDW                   EM
SSB                    0
LB                  0.30 Hz
GB                     0
PC                  1.00
```

^{1}H NMR of **1af** ($CDCl_3$, 400 MHz)

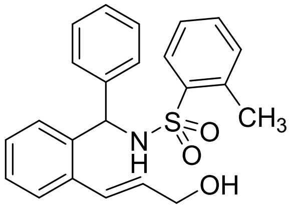

1af

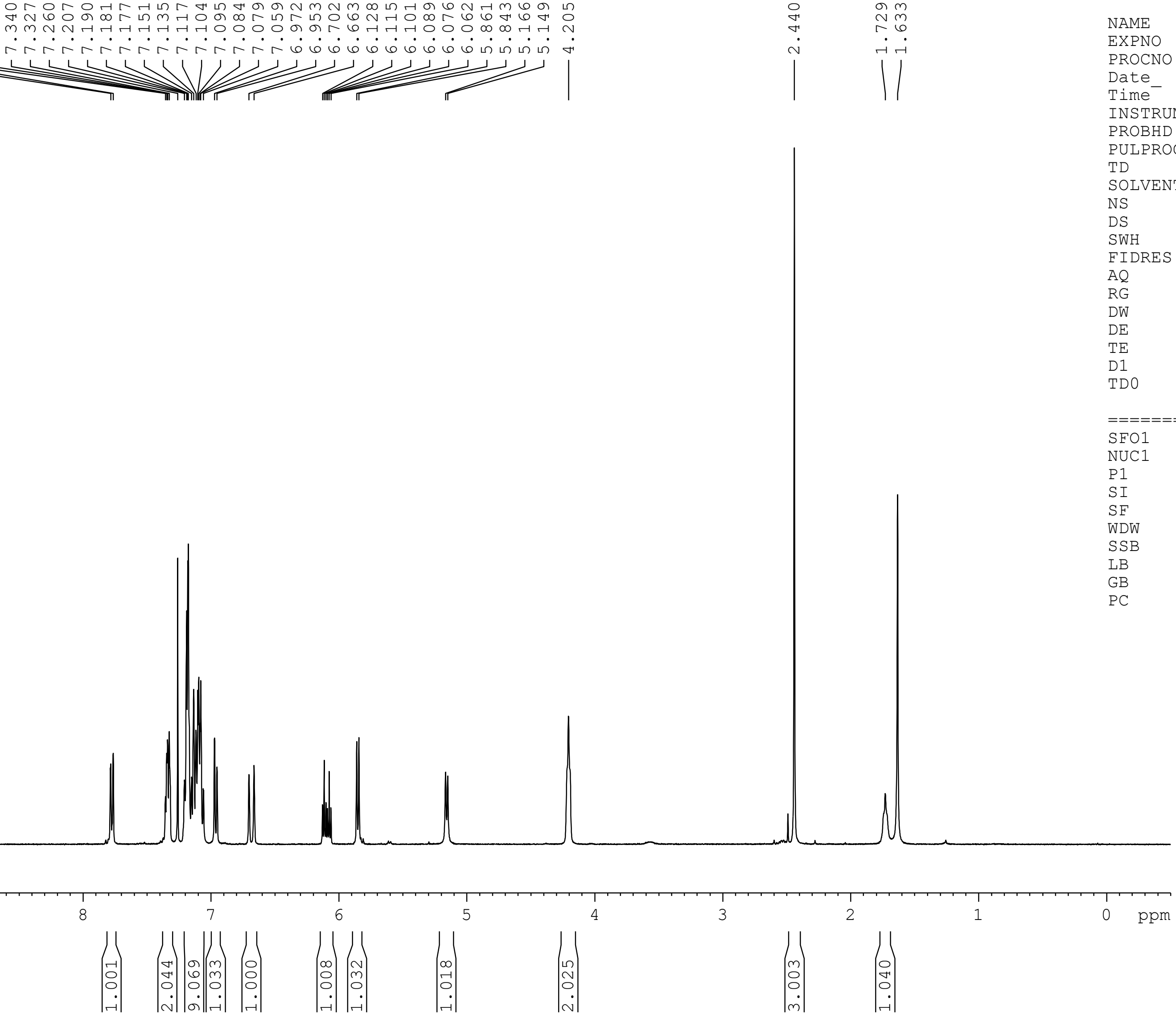

NAME	202405
EXPNO	213
PROCNO	1
Date_	20240511
Time	13.09
INSTRUM	spect
PROBHD	5 mm PABBO BB/
PULPROG	zg30
TD	32768
SOLVENT	CDCl3
NS	15
DS	0
SWH	8012.820 Hz
FIDRES	0.244532 Hz
AQ	2.0447731 sec
RG	205.92
DW	62.400 usec
DE	16.53 usec
TE	295.5 K
D1	2.00000000 sec
TD0	1

======== CHANNEL f1 ========

SFO1	400.1324008 MHz
NUC1	1H
P1	14.00 usec
SI	16384
SF	400.1300095 MHz
WDW	EM
SSB	0
LB	0.00 Hz
GB	0
PC	1.00

$^{13}C\{^1H\}$ NMR of **1af** ($CDCl_3$, 101 MHz)

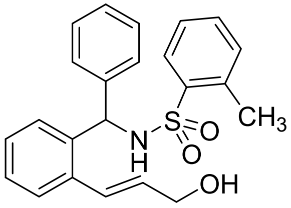

1af

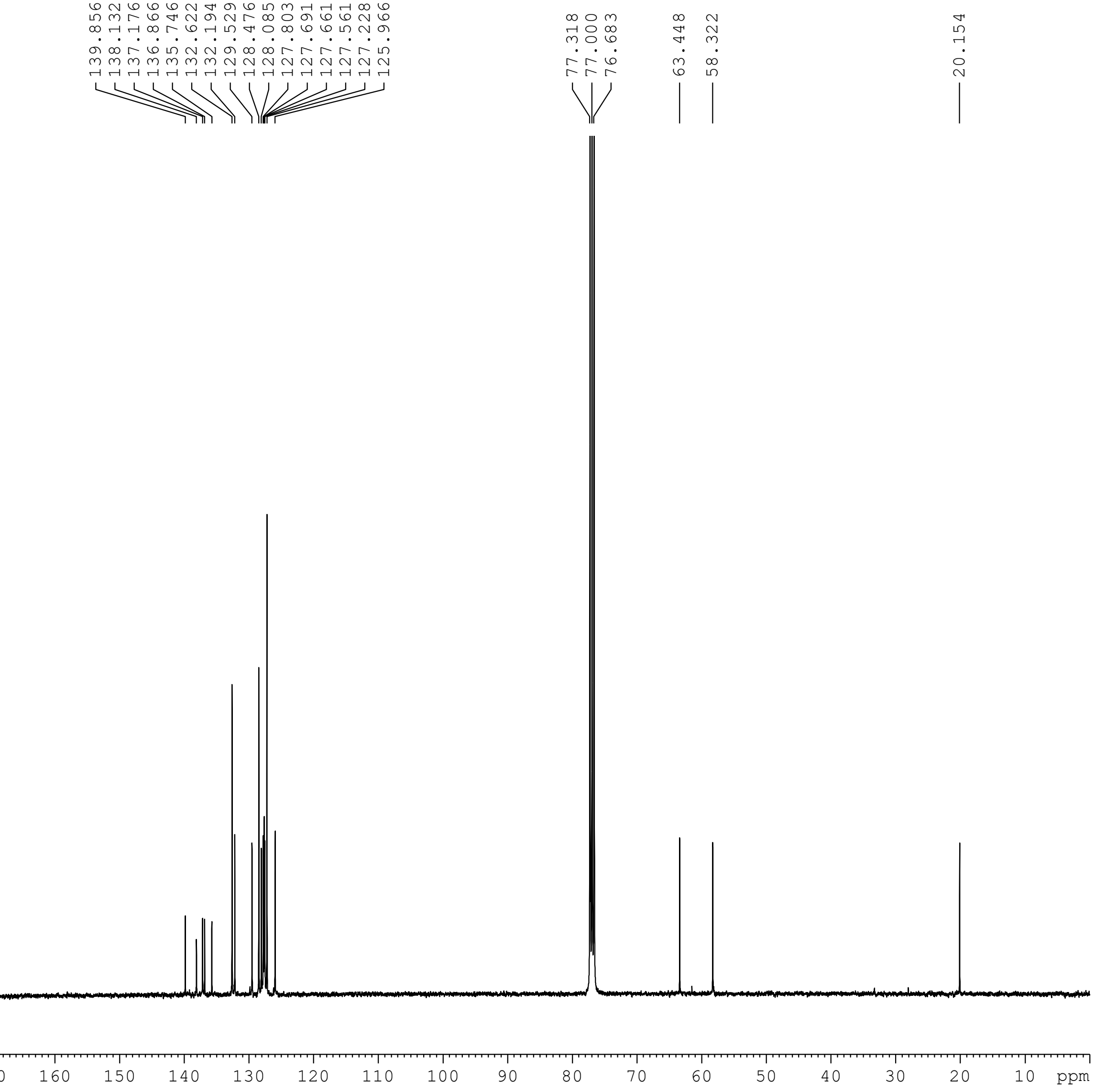

NAME	202405
EXPNO	216
PROCNO	1
Date_	20240512
Time	2.58
INSTRUM	spect
PROBHD	5 mm PABBO BB/
PULPROG	zgpg30
TD	32768
SOLVENT	CDCl3
NS	10000
DS	0
SWH	24038.461 Hz
FIDRES	0.733596 Hz
AQ	0.6816244 sec
RG	205.92
DW	20.800 usec
DE	6.50 usec
TE	296.5 K
D1	2.00000000 sec
D11	0.03000000 sec
TD0	1

======== CHANNEL f1 ========

SFO1	100.6233329 MHz
NUC1	13C
P1	10.00 usec
SI	32768
SF	100.6127714 MHz
WDW	EM
SSB	0
LB	2.00 Hz
GB	0
PC	1.00

^{1}H NMR of **1ag** ($CDCl_3$, 400 MHz)

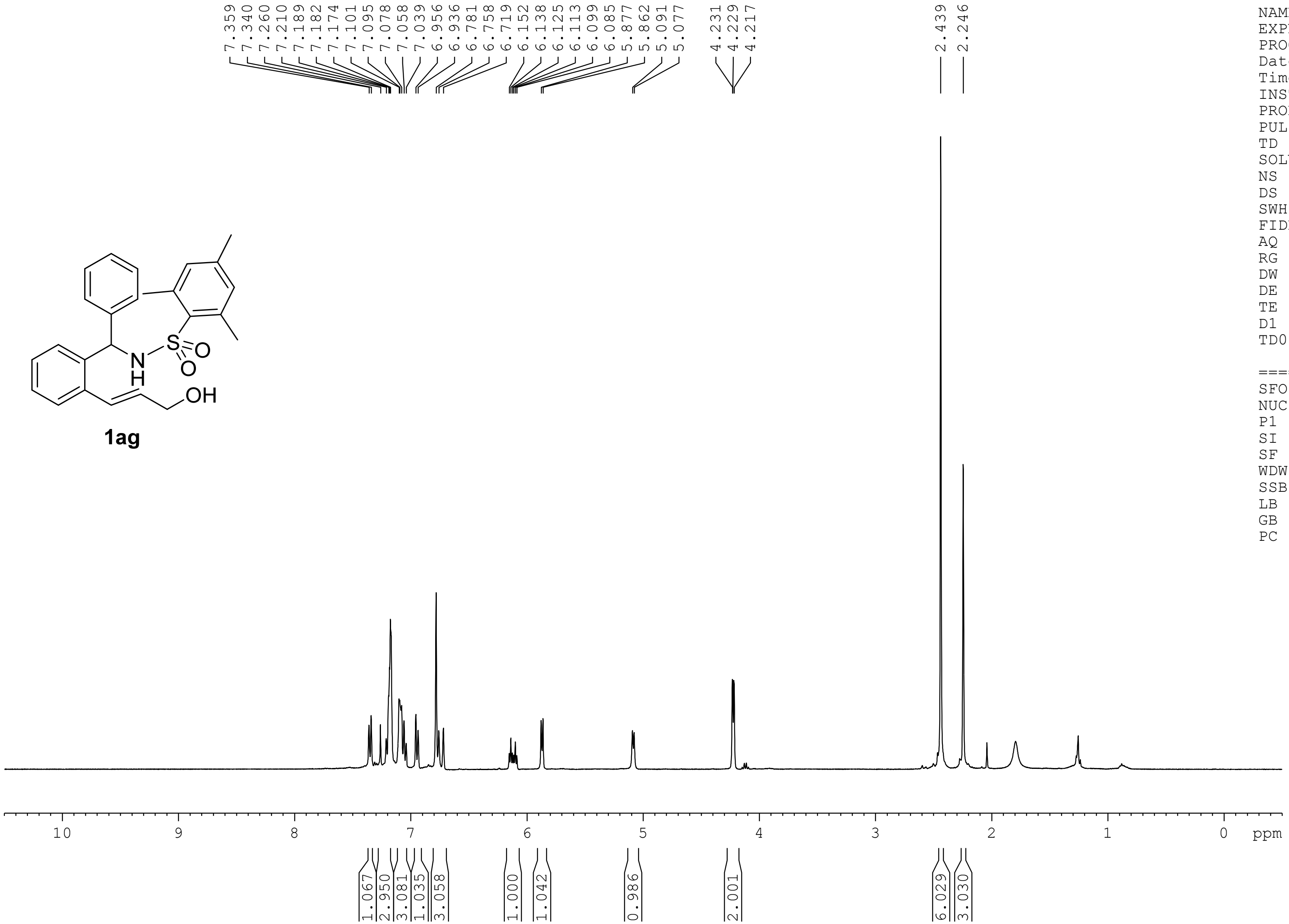


```
NAME              CCT113
EXPNO                422
PROCNO                 1
Date_           20250114
Time               15.36
INSTRUM            spect
PROBHD   5 mm PABBO BB/
PULPROG             zg30
TD                 32768
SOLVENT            CDCl3
NS                     8
DS                     0
SWH             8012.820 Hz
FIDRES          0.244532 Hz
AQ             2.0447731 sec
RG                 122.8
DW                62.400 usec
DE                 16.53 usec
TE                 297.1 K
D1            2.00000000 sec
TD0                    1

======== CHANNEL f1 ========
SFO1         400.1324008 MHz
NUC1                  1H
P1                 14.00 usec
SI                 16384
SF           400.1300097 MHz
WDW                   EM
SSB                    0
LB                  0.00 Hz
GB                     0
PC                  1.00
```

$^{13}C\{^{1}H\}$ NMR of **1ag** ($CDCl_3$, 101 MHz)

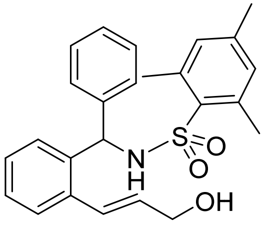

1ag

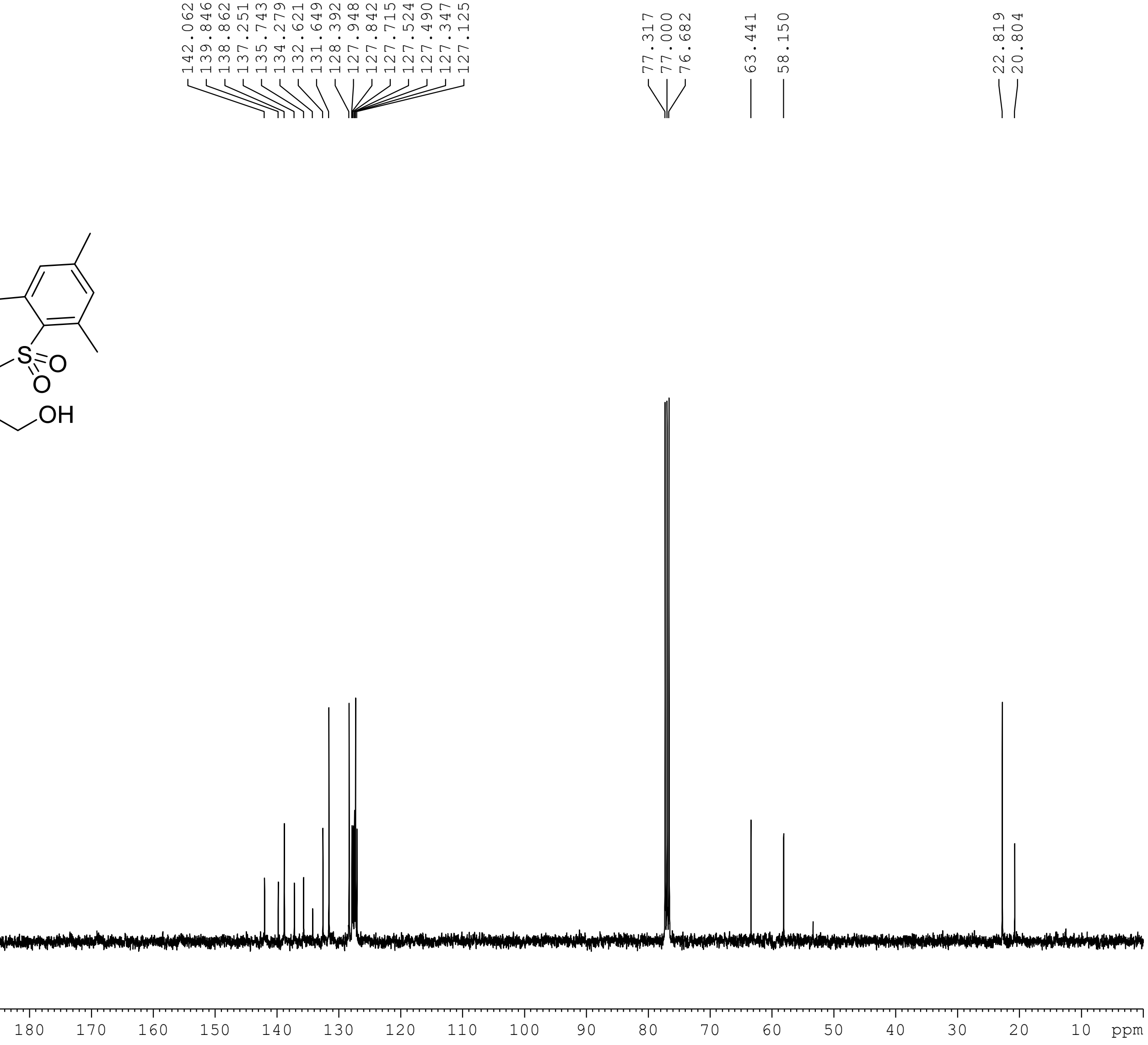

NAME	CCT113	
EXPNO	420	
PROCNO	1	
Date_	20250114	
Time	14.09	
INSTRUM	spect	
PROBHD	5 mm PABBO BB/	
PULPROG	zgpg30	
TD	32768	
SOLVENT	CDCl3	
NS	138	
DS	0	
SWH	24038.461	Hz
FIDRES	0.733596	Hz
AQ	0.6816244	sec
RG	205.92	
DW	20.800	usec
DE	6.50	usec
TE	297.6	K
D1	2.00000000	sec
D11	0.03000000	sec
TD0	1	

======== CHANNEL f1 ========

SFO1	100.6233329	MHz
NUC1	13C	
P1	10.00	usec
SI	32768	
SF	100.6127732	MHz
WDW	EM	
SSB	0	
LB	2.00	Hz
GB	0	
PC	1.00	

^{1}H NMR of **1ah** ($CDCl_3$, 400 MHz)

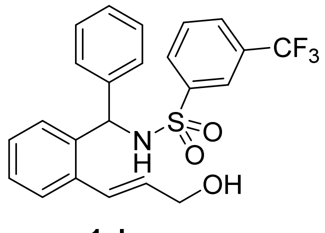

1ah

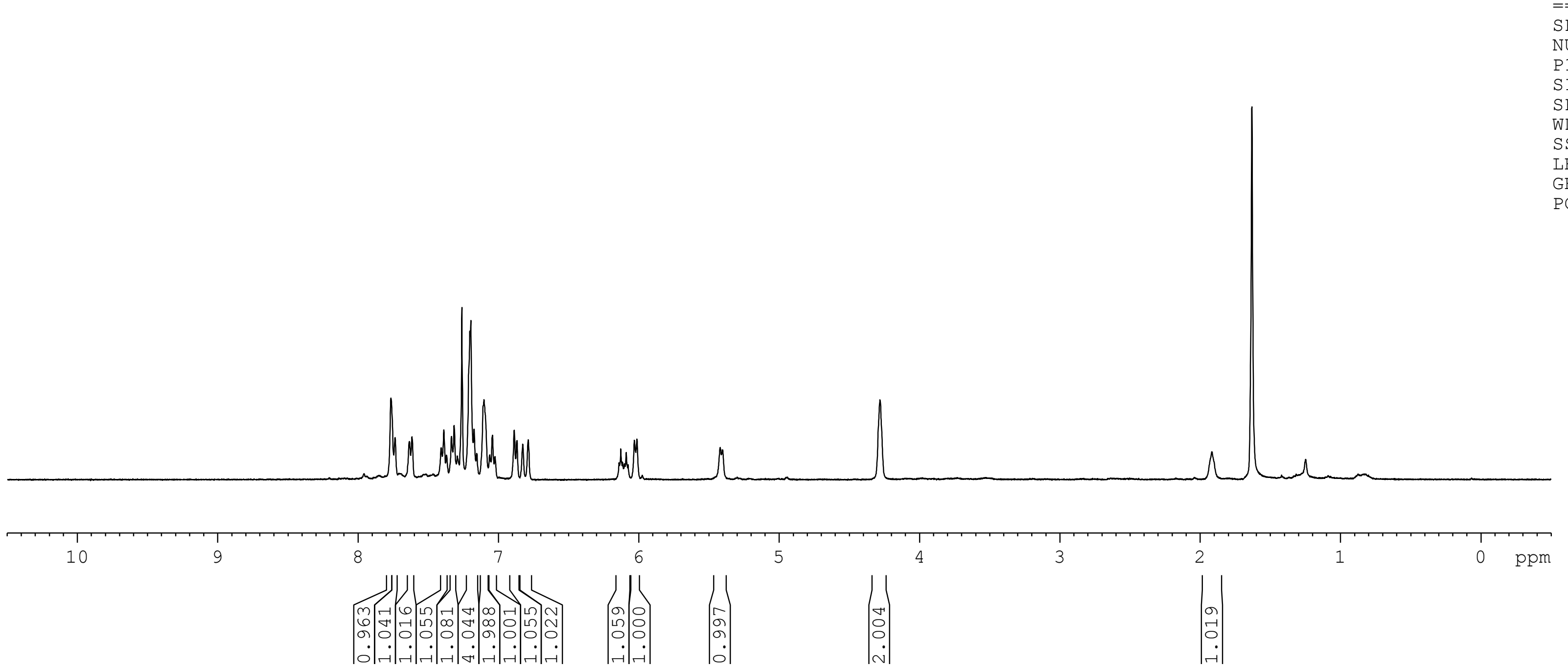

NAME	202304
EXPNO	102
PROCNO	1
Date_	20230414
Time	19.52
INSTRUM	spect
PROBHD	5 mm PABBO BB/
PULPROG	zg30
TD	32768
SOLVENT	CDCl3
NS	16
DS	0
SWH	8012.820 Hz
FIDRES	0.244532 Hz
AQ	2.0447731 sec
RG	205.92
DW	62.400 usec
DE	16.53 usec
TE	288.8 K
D1	2.00000000 sec
TD0	1

======== CHANNEL f1 ========

SFO1	400.1324008 MHz
NUC1	1H
P1	14.00 usec
SI	16384
SF	400.1300098 MHz
WDW	EM
SSB	0
LB	0.00 Hz
GB	0
PC	1.00

$^{13}C\{^{1}H\}$ NMR of **1ah** ($CDCl_3$, 101 MHz)

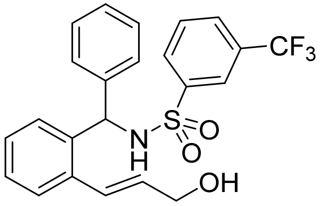

1ah

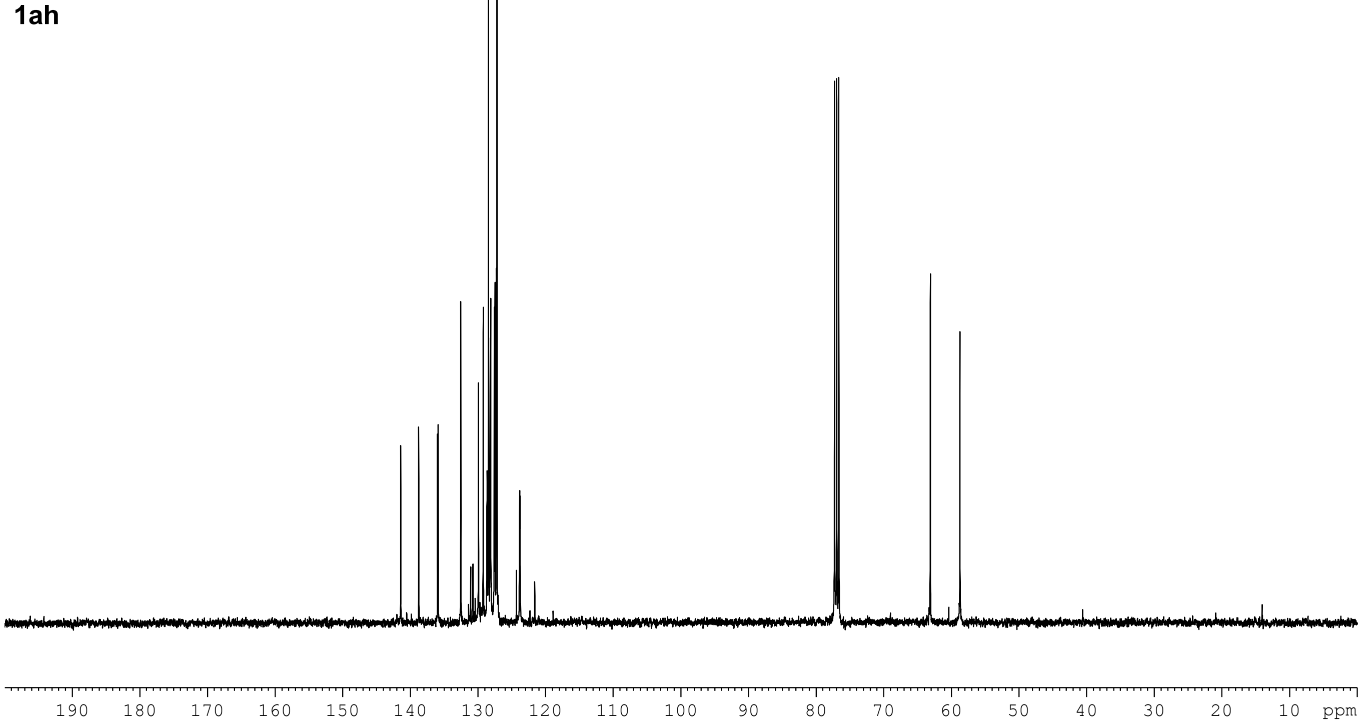

NAME	202405
EXPNO	318
PROCNO	1
Date_	20240518
Time	20.47
INSTRUM	spect
PROBHD	5 mm PABBO BB/
PULPROG	zgpg30
TD	32768
SOLVENT	CDCl3
NS	17000
DS	0
SWH	24038.461 Hz
FIDRES	0.733596 Hz
AQ	0.6816244 sec
RG	205.92
DW	20.800 usec
DE	6.50 usec
TE	295.8 K
D1	2.00000000 sec
D11	0.03000000 sec
TD0	1

======== CHANNEL f1 ========

SFO1	100.6233329 MHz
NUC1	13C
P1	10.00 usec
SI	32768
SF	100.6127776 MHz
WDW	EM
SSB	0
LB	2.00 Hz
GB	0
PC	1.00

^{19}F NMR of **1ah** ($CDCl_3$, 376 MHz)

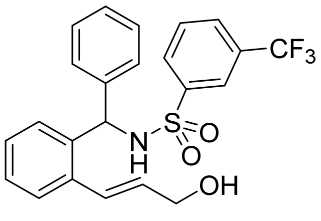

1ah

-62.775

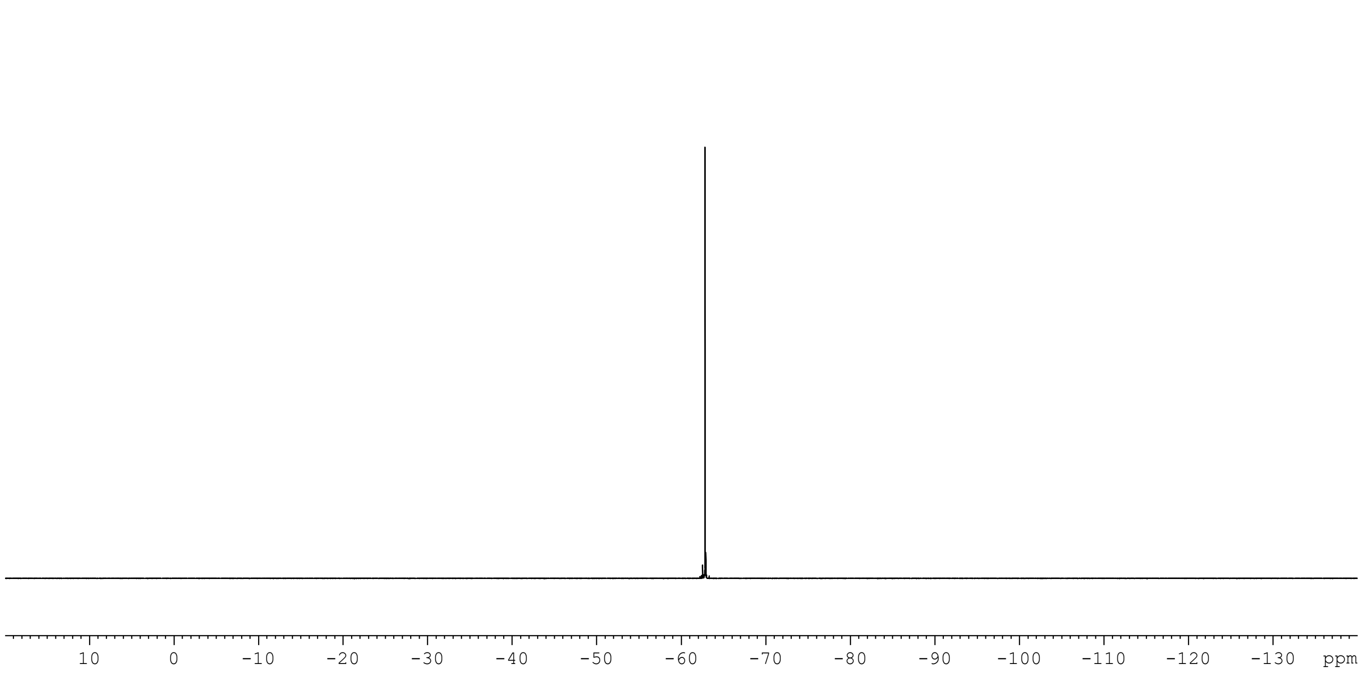


```
NAME               202308
EXPNO                  79
PROCNO                  1
Date_            20230803
Time                17.42
INSTRUM             spect
PROBHD   5 mm PABBO BB/
PULPROG              zg30
TD                 131072
SOLVENT             CDCl3
NS                     16
DS                      0
SWH            89285.711 Hz
FIDRES          0.681196 Hz
AQ             0.7340532 sec
RG                 205.92
DW                  5.600 usec
DE                   6.50 usec
TE                  290.9 K
D1             1.00000000 sec
TD0                     1

======== CHANNEL f1 ========
SFO1         376.4757776 MHz
NUC1                  19F
P1                  15.00 usec
SI                  65536
SF           376.4983662 MHz
WDW                    EM
SSB                     0
LB                   0.30 Hz
GB                      0
PC                   1.00
```

^{1}H NMR of **1ai** ($CDCl_3$, 400 MHz)

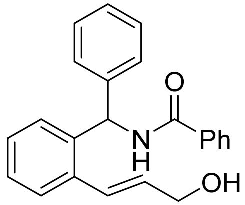

1ai

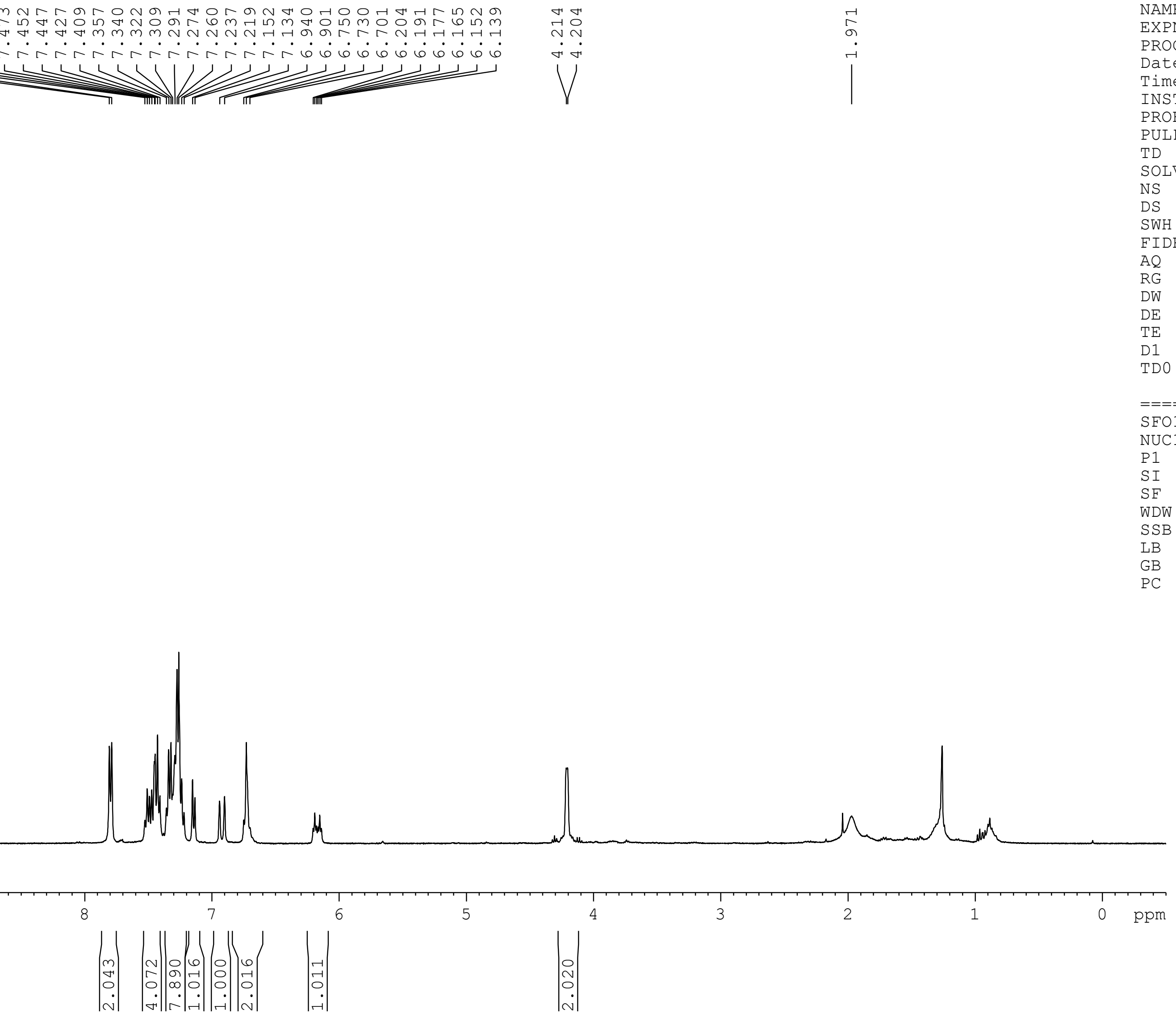

NAME CCT113
EXPNO 474
PROCNO 1
Date_ 20250414
Time 12.08
INSTRUM spect
PROBHD 5 mm PABBO BB/
PULPROG zg30
TD 32768
SOLVENT CDCl3
NS 6
DS 0
SWH 8012.820 Hz
FIDRES 0.244532 Hz
AQ 2.0447731 sec
RG 181.8
DW 62.400 usec
DE 16.53 usec
TE 296.2 K
D1 2.00000000 sec
TD0 1

======== CHANNEL f1 ========
SFO1 400.1324008 MHz
NUC1 1H
P1 14.00 usec
SI 16384
SF 400.1300092 MHz
WDW EM
SSB 0
LB 0.00 Hz
GB 0
PC 1.00

$^{13}C\{^{1}H\}$ NMR of **1ai** ($CDCl_3$, 101 MHz)

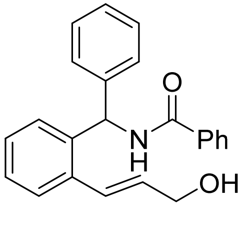

1ai

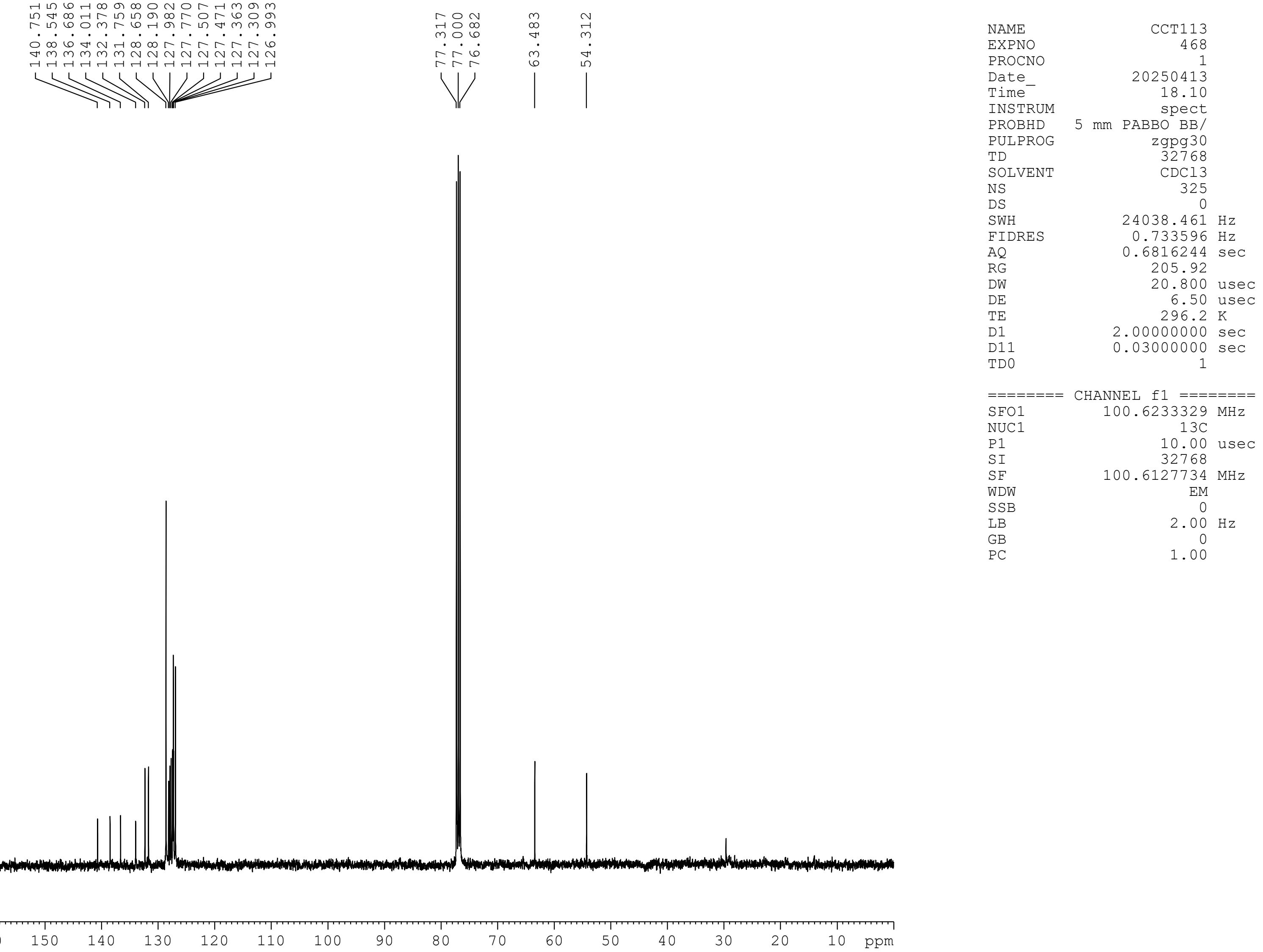

^{1}H NMR of **1aj** ($CDCl_3$, 400 MHz)

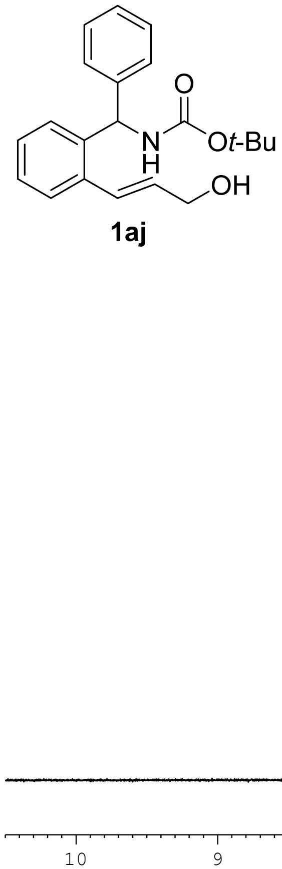

1aj

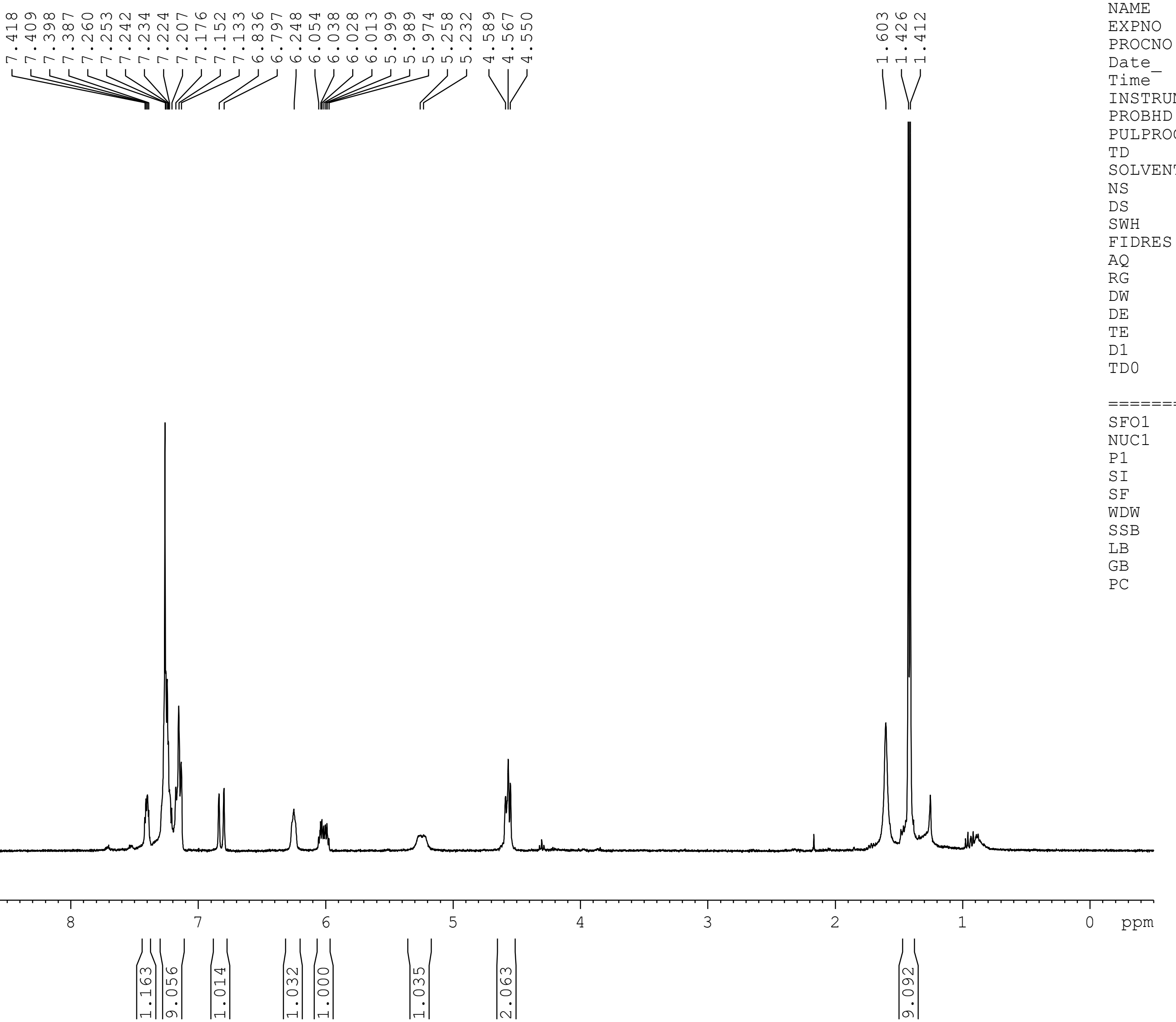


```
NAME              CCT113
EXPNO                477
PROCNO                 1
Date_           20250416
Time               16.55
INSTRUM            spect
PROBHD   5 mm PABBO BB/
PULPROG             zg30
TD                 32768
SOLVENT            CDCl3
NS                     7
DS                     0
SWH             8012.820 Hz
FIDRES          0.244532 Hz
AQ             2.0447731 sec
RG                205.92
DW                62.400 usec
DE                 16.53 usec
TE                 296.3 K
D1            2.00000000 sec
TD0                    1

======== CHANNEL f1 ========
SFO1         400.1324008 MHz
NUC1                  1H
P1                 14.00 usec
SI                 16384
SF           400.1300097 MHz
WDW                   EM
SSB                    0
LB                  0.00 Hz
GB                     0
PC                  1.00
```

$^{13}C\{^{1}H\}$ NMR of **1aj** ($CDCl_3$, 101 MHz)

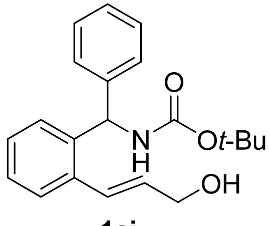

1aj

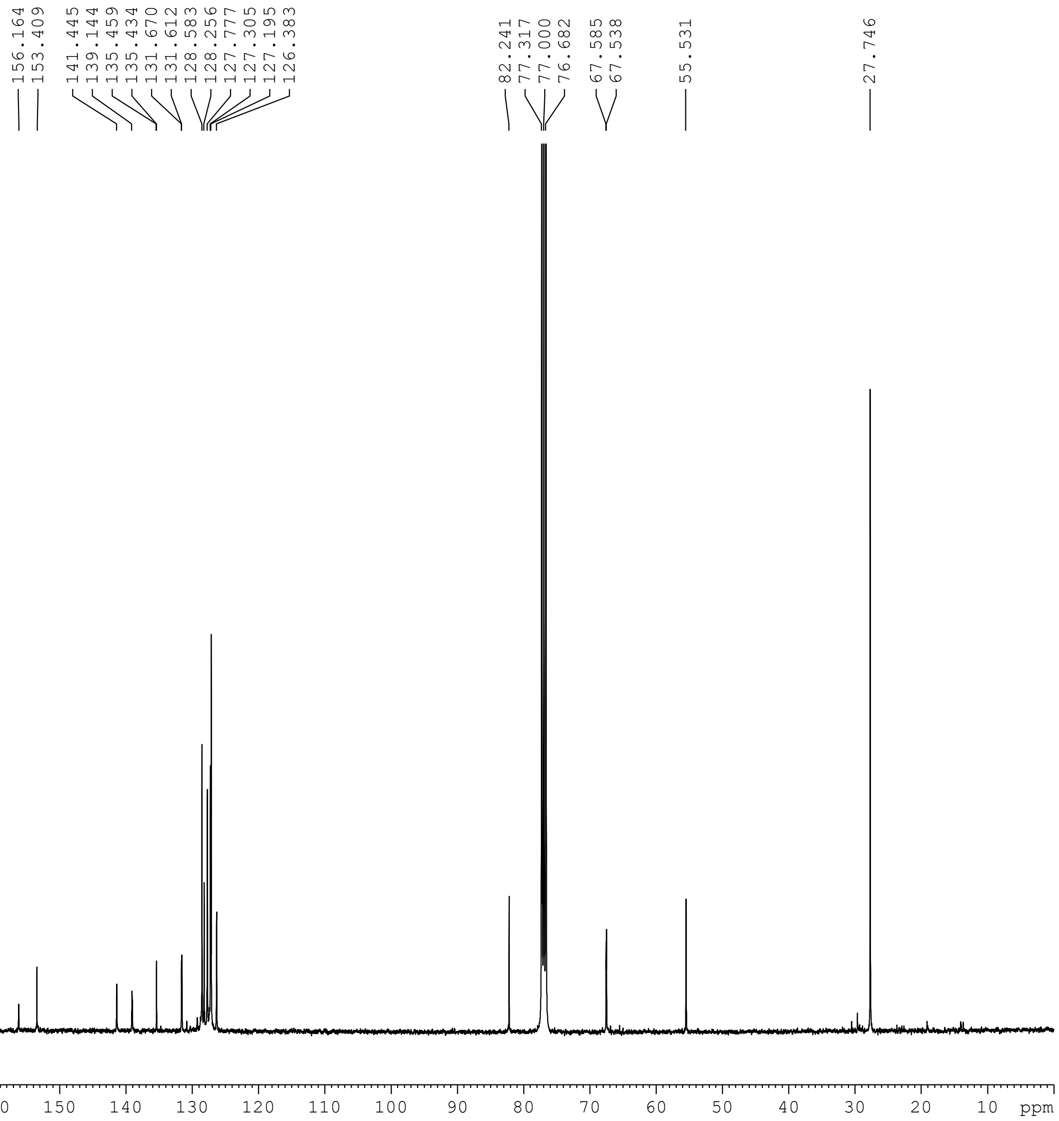

NAME	CCT113	
EXPNO	484	
PROCNO	1	
Date_	20250418	
Time	20.21	
INSTRUM	spect	
PROBHD	5 mm PABBO BB/	
PULPROG	zgpg30	
TD	32768	
SOLVENT	CDCl3	
NS	15315	
DS	0	
SWH	24038.461	Hz
FIDRES	0.733596	Hz
AQ	0.6816244	sec
RG	205.92	
DW	20.800	usec
DE	6.50	usec
TE	296.2	K
D1	2.00000000	sec
D11	0.03000000	sec
TD0	1	
======== CHANNEL f1 ========		
SFO1	100.6233329	MHz
NUC1	13C	
P1	10.00	usec
SI	32768	
SF	100.6127710	MHz
WDW	EM	
SSB	0	
LB	2.00	Hz
GB	0	
PC	1.00	

^{1}H NMR of **1bh** ($CDCl_3$, 400 MHz)

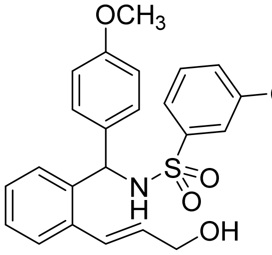

1bh

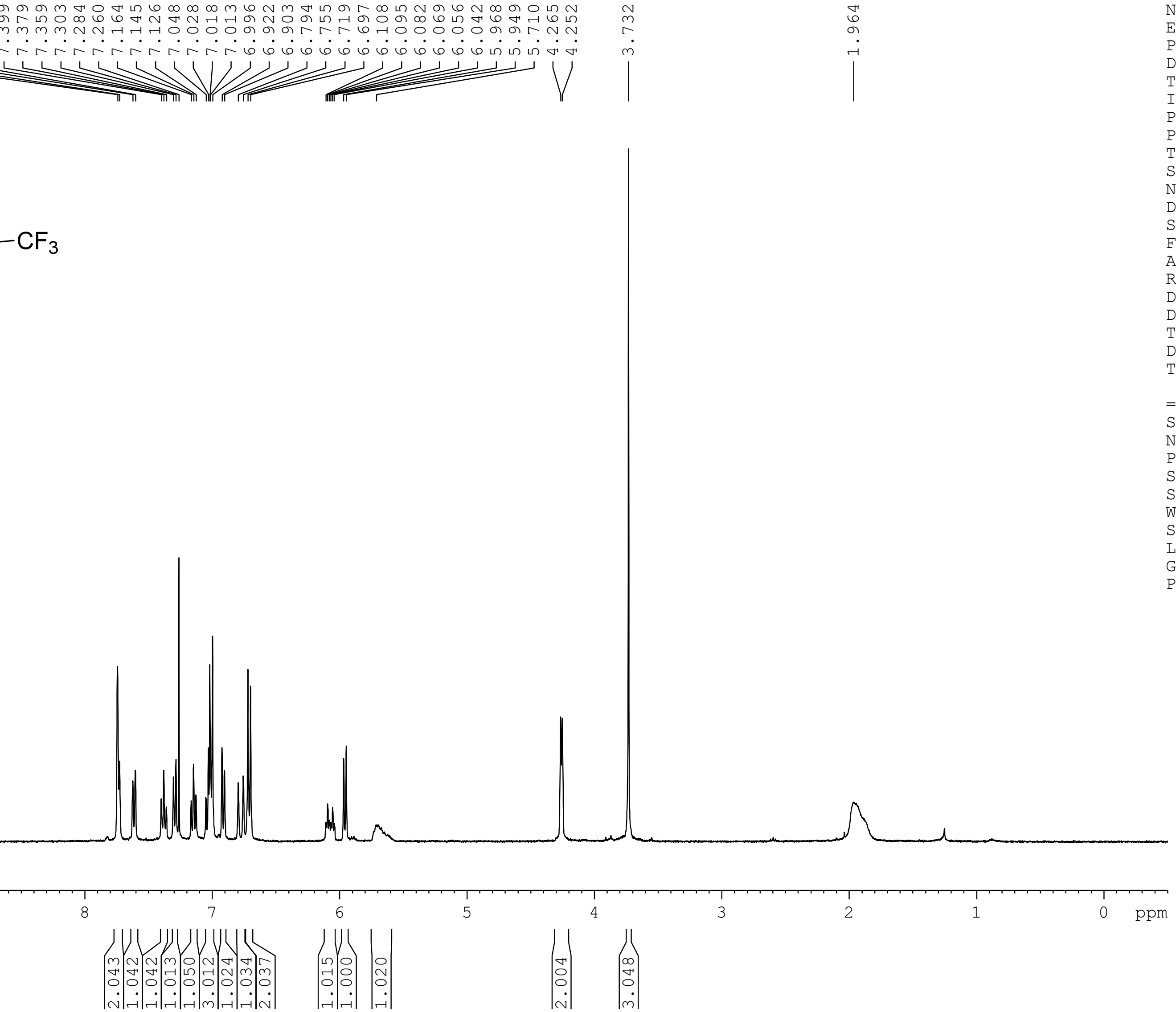

NAME	202408
EXPNO	42
PROCNO	1
Date_	20240802
Time	12.02
INSTRUM	spect
PROBHD	5 mm PABBO BB/
PULPROG	zg30
TD	32768
SOLVENT	CDCl3
NS	16
DS	0
SWH	8012.820 Hz
FIDRES	0.244532 Hz
AQ	2.0447731 sec
RG	137.93
DW	62.400 usec
DE	16.53 usec
TE	291.6 K
D1	2.00000000 sec
TD0	1

======== CHANNEL f1 ========

SFO1	400.1324008 MHz
NUC1	1H
P1	14.00 usec
SI	16384
SF	400.1300096 MHz
WDW	EM
SSB	0
LB	0.00 Hz
GB	0
PC	1.00

$^{13}C\{^{1}H\}$ NMR of **1bh** ($CDCl_3$, 101 MHz)

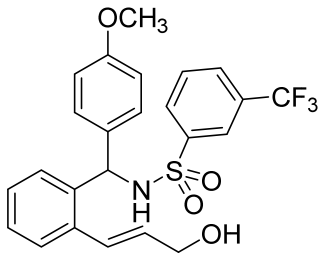

1bh

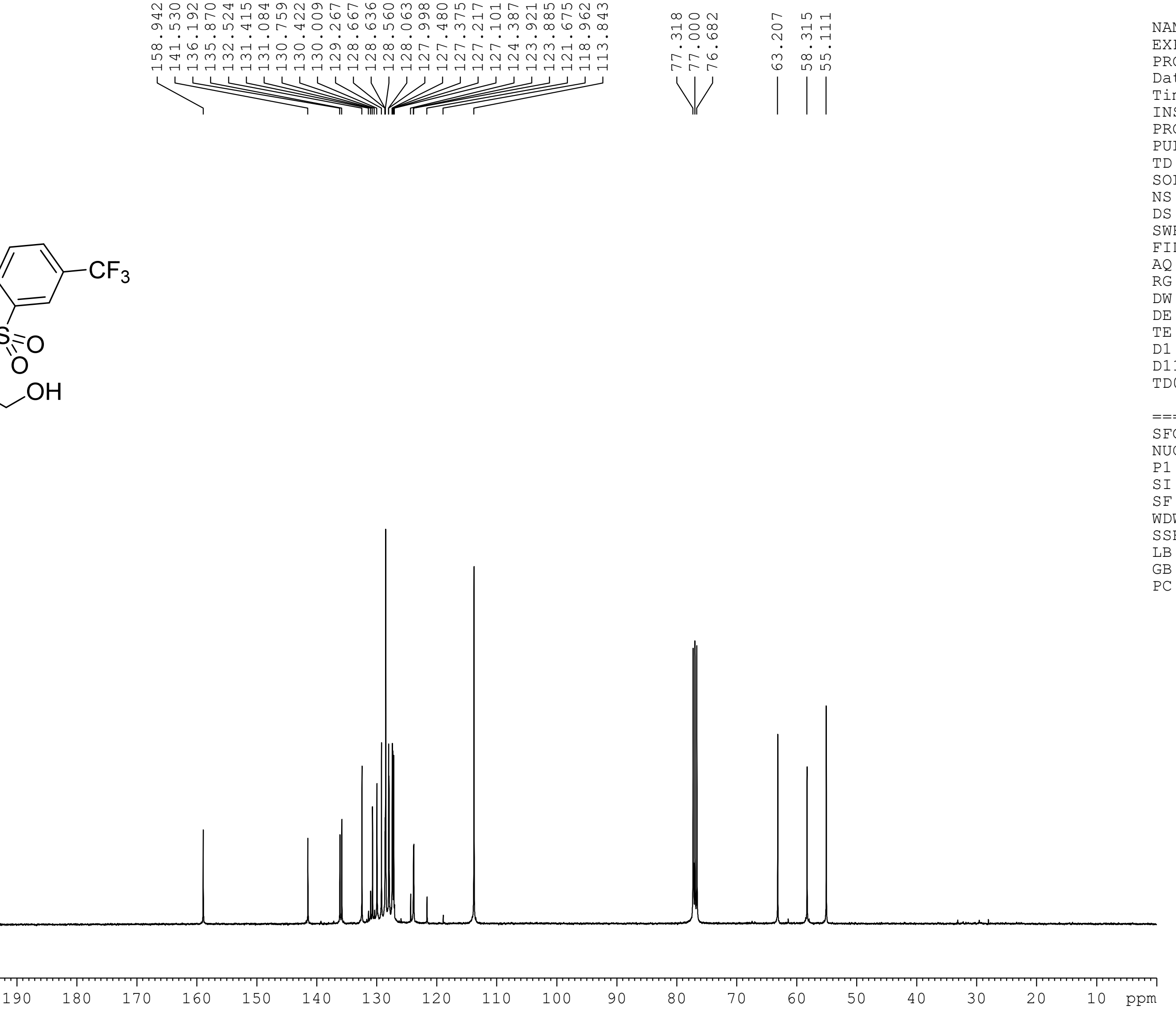

NAME 202407
EXPNO 605
PROCNO 1
Date_ 20240731
Time 1.58
INSTRUM spect
PROBHD 5 mm PABBO BB/
PULPROG zgpg30
TD 32768
SOLVENT CDCl3
NS 7500
DS 0
SWH 24038.461 Hz
FIDRES 0.733596 Hz
AQ 0.6816244 sec
RG 205.92
DW 20.800 usec
DE 6.50 usec
TE 297.2 K
D1 2.00000000 sec
D11 0.03000000 sec
TD0 1

======== CHANNEL f1 ========
SFO1 100.6233329 MHz
NUC1 13C
P1 10.00 usec
SI 32768
SF 100.6127769 MHz
WDW EM
SSB 0
LB 2.00 Hz
GB 0
PC 1.00

^{19}F NMR of **1bh** ($CDCl_3$, 376 MHz)

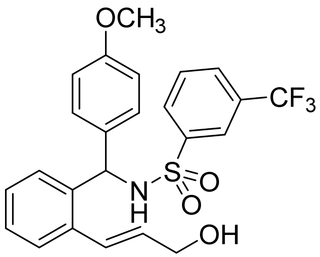

1bh

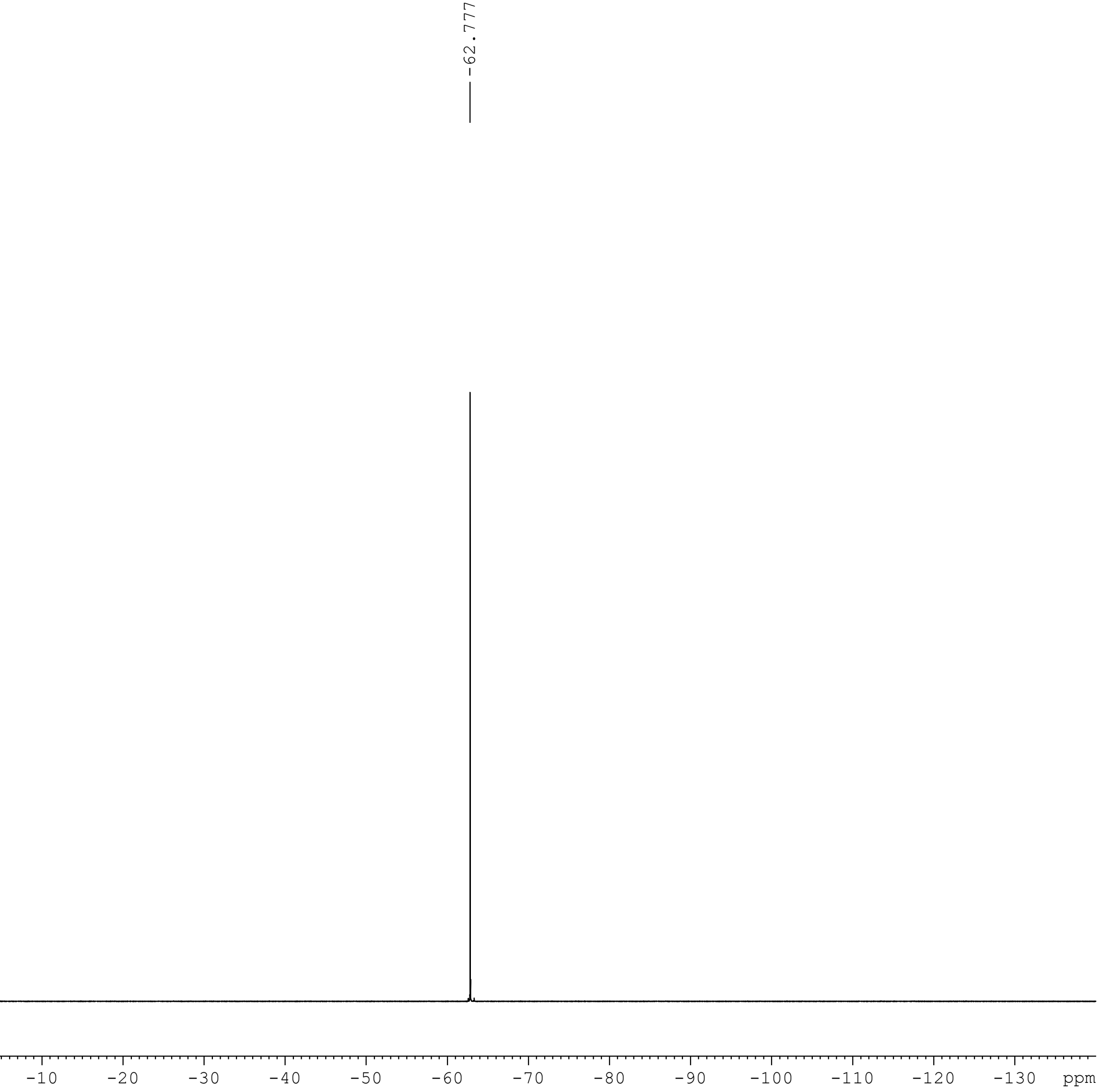


```
NAME              202407
EXPNO                574
PROCNO                 1
Date_           20240727
Time               16.30
INSTRUM            spect
PROBHD   5 mm PABBO BB/
PULPROG             zg30
TD                131072
SOLVENT            CDCl3
NS                     3
DS                     0
SWH           89285.711 Hz
FIDRES         0.681196 Hz
AQ            0.7340532 sec
RG                205.92
DW                 5.600 usec
DE                  6.50 usec
TE                 293.4 K
D1            1.00000000 sec
TD0                    1

======== CHANNEL f1 ========
SFO1         376.4757776 MHz
NUC1                 19F
P1                 15.00 usec
SI                 65536
SF           376.4983662 MHz
WDW                   EM
SSB                    0
LB                  0.30 Hz
GB                     0
PC                  1.00
```

^{1}H NMR of **1ch** ($CDCl_3$, 400 MHz)

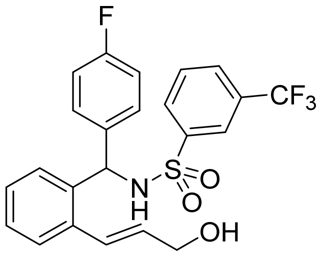

1ch

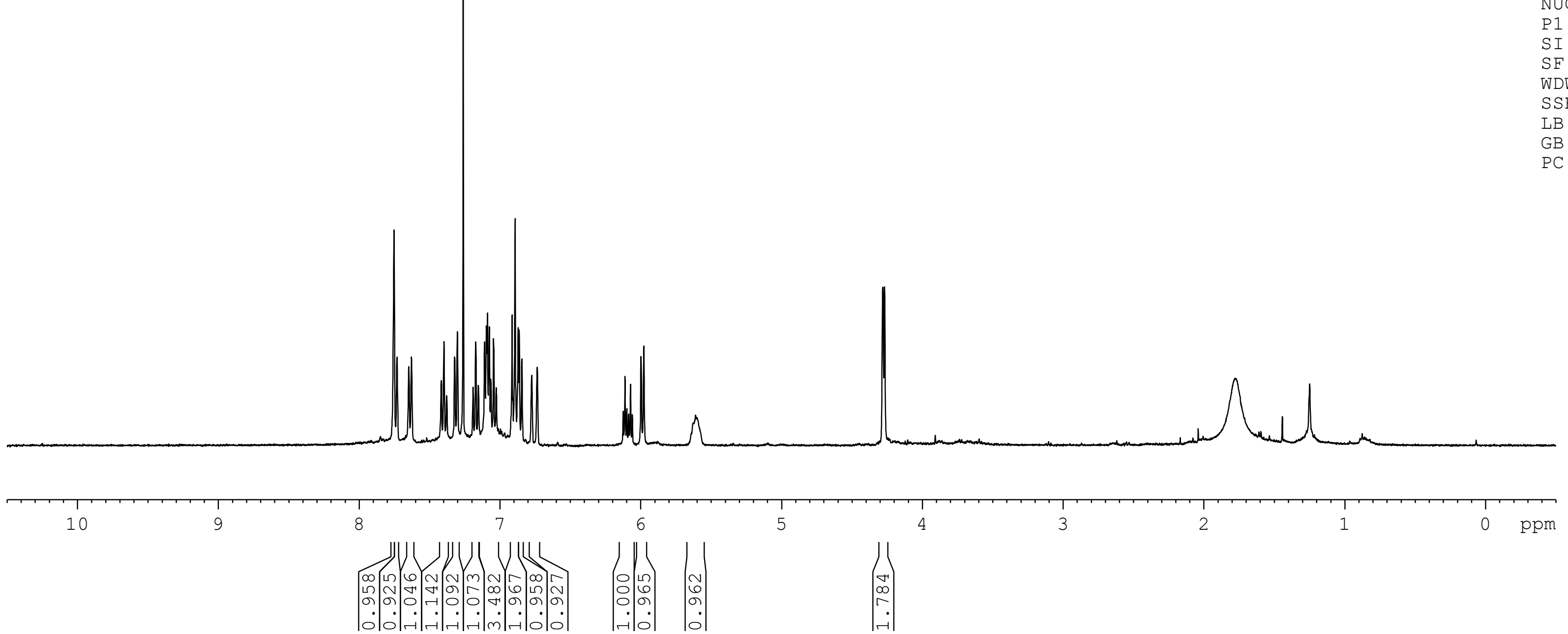


```
NAME               202407
EXPNO                 339
PROCNO                  1
Date_            20240716
Time                13.04
INSTRUM             spect
PROBHD   5 mm PABBO BB/
PULPROG              zg30
TD                  32768
SOLVENT             CDCl3
NS                      7
DS                      0
SWH             8012.820 Hz
FIDRES          0.244532 Hz
AQ             2.0447731 sec
RG                 205.92
DW                 62.400 usec
DE                  16.53 usec
TE                  291.0 K
D1            2.00000000 sec
TD0                     1

======== CHANNEL f1 ========
SFO1         400.1324008 MHz
NUC1                   1H
P1                  14.00 usec
SI                  16384
SF           400.1300096 MHz
WDW                    EM
SSB                     0
LB                   0.00 Hz
GB                      0
PC                   1.00
```

$^{13}C\{^{1}H\}$ NMR of **1ch** ($CDCl_3$, 101 MHz)

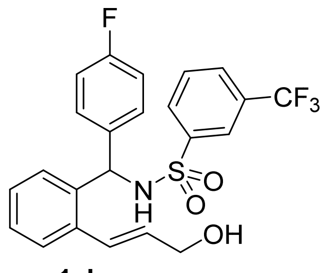

1ch

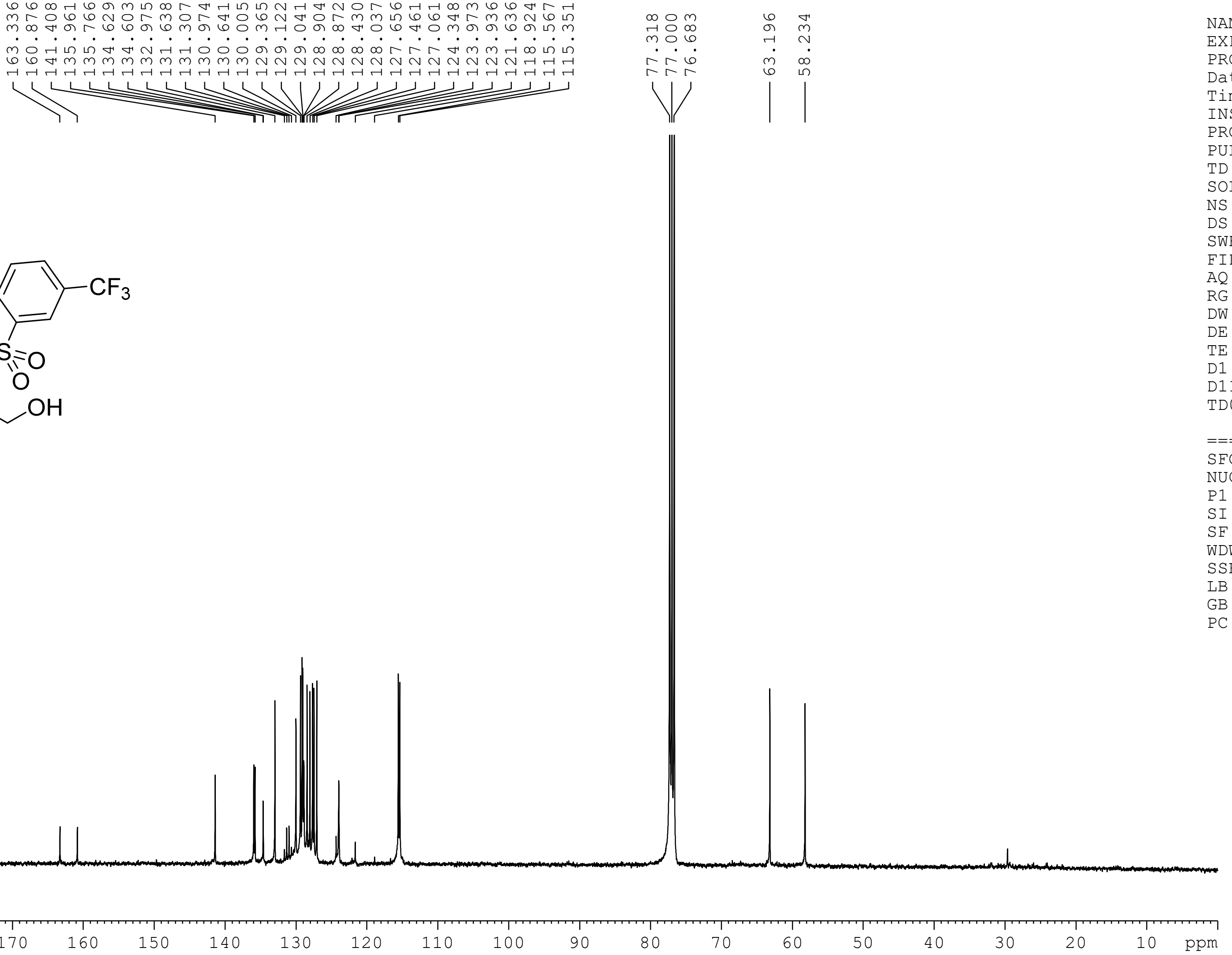

NAME	202407
EXPNO	229
PROCNO	1
Date_	20240711
Time	8.17
INSTRUM	spect
PROBHD	5 mm PABBO BB/
PULPROG	zgpg30
TD	32768
SOLVENT	CDCl3
NS	17058
DS	0
SWH	24038.461 Hz
FIDRES	0.733596 Hz
AQ	0.6816244 sec
RG	205.92
DW	20.800 usec
DE	6.50 usec
TE	292.8 K
D1	2.00000000 sec
D11	0.03000000 sec
TD0	1

======== CHANNEL f1 ========

SFO1	100.6233329 MHz
NUC1	13C
P1	10.00 usec
SI	32768
SF	100.6127730 MHz
WDW	EM
SSB	0
LB	2.00 Hz
GB	0
PC	1.00

^{19}F NMR of **1ch** ($CDCl_3$, 376 MHz)

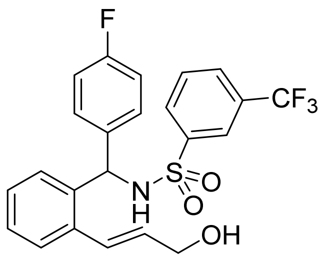

1ch

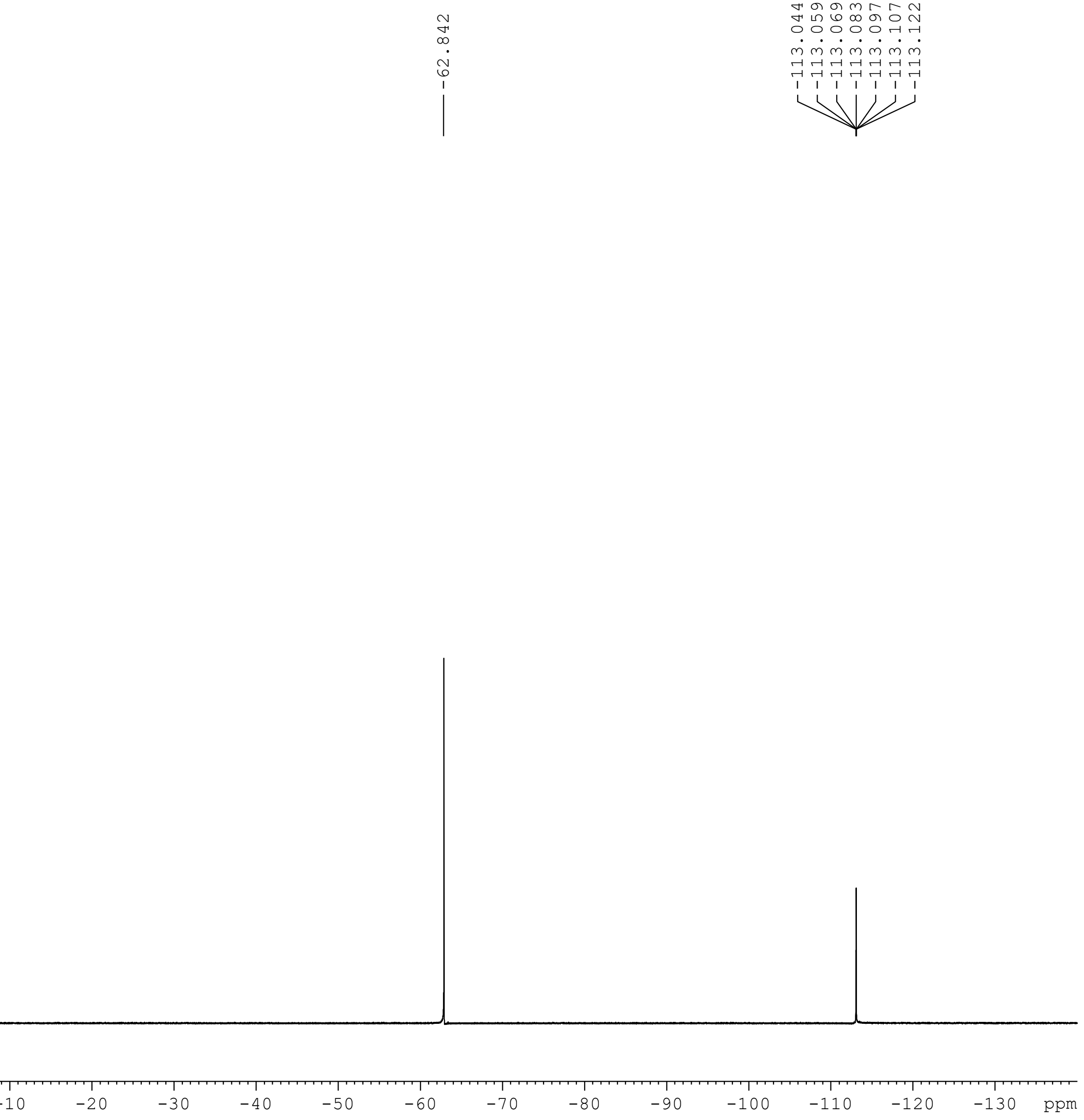

NAME	202408
EXPNO	293
PROCNO	1
Date_	20240815
Time	18.24
INSTRUM	spect
PROBHD	5 mm PABBO BB/
PULPROG	zg30
TD	131072
SOLVENT	CDCl3
NS	30
DS	0
SWH	89285.711 Hz
FIDRES	0.681196 Hz
AQ	0.7340532 sec
RG	205.92
DW	5.600 usec
DE	6.50 usec
TE	292.9 K
D1	1.00000000 sec
TD0	1

======== CHANNEL f1 ========

SFO1	376.4757776 MHz
NUC1	19F
P1	15.00 usec
SI	65536
SF	376.4983662 MHz
WDW	EM
SSB	0
LB	0.30 Hz
GB	0
PC	1.00

^{1}H NMR of **1dh** ($CDCl_3$, 400 MHz)

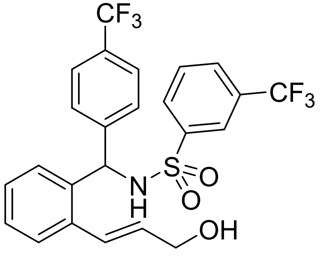

1dh

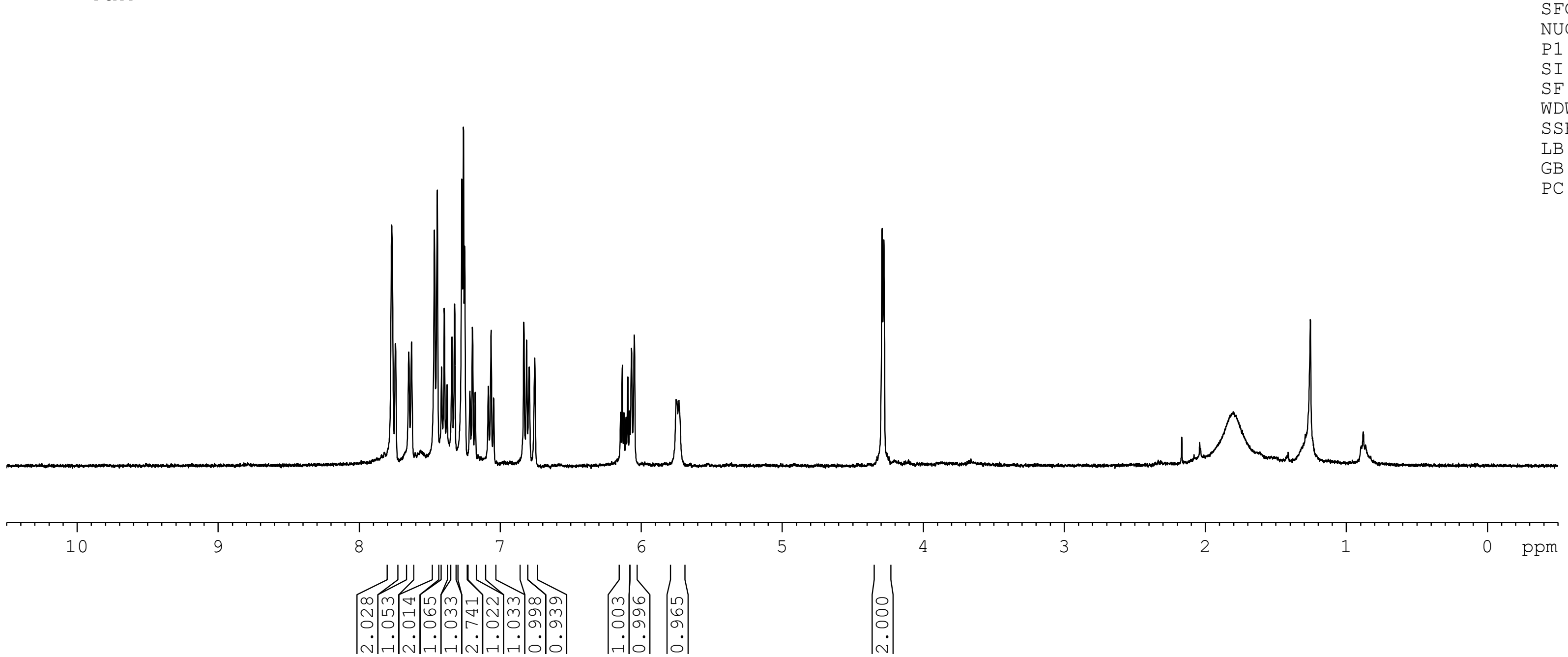

NAME	202408
EXPNO	413
PROCNO	1
Date_	20240826
Time	10.27
INSTRUM	spect
PROBHD	5 mm PABBO BB/
PULPROG	zg30
TD	32768
SOLVENT	CDCl3
NS	15
DS	0
SWH	8012.820 Hz
FIDRES	0.244532 Hz
AQ	2.0447731 sec
RG	205.92
DW	62.400 usec
DE	16.53 usec
TE	296.0 K
D1	2.00000000 sec
TD0	1

======== CHANNEL f1 ========

SFO1	400.1324008 MHz
NUC1	1H
P1	14.00 usec
SI	16384
SF	400.1300104 MHz
WDW	EM
SSB	0
LB	0.00 Hz
GB	0
PC	1.00

$^{13}C\{^{1}H\}$ NMR of **1dh** ($CDCl_3$, 101 MHz)

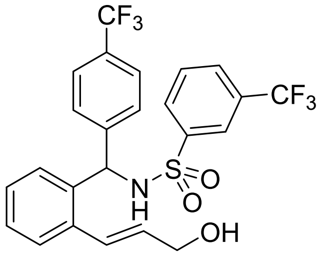

1dh

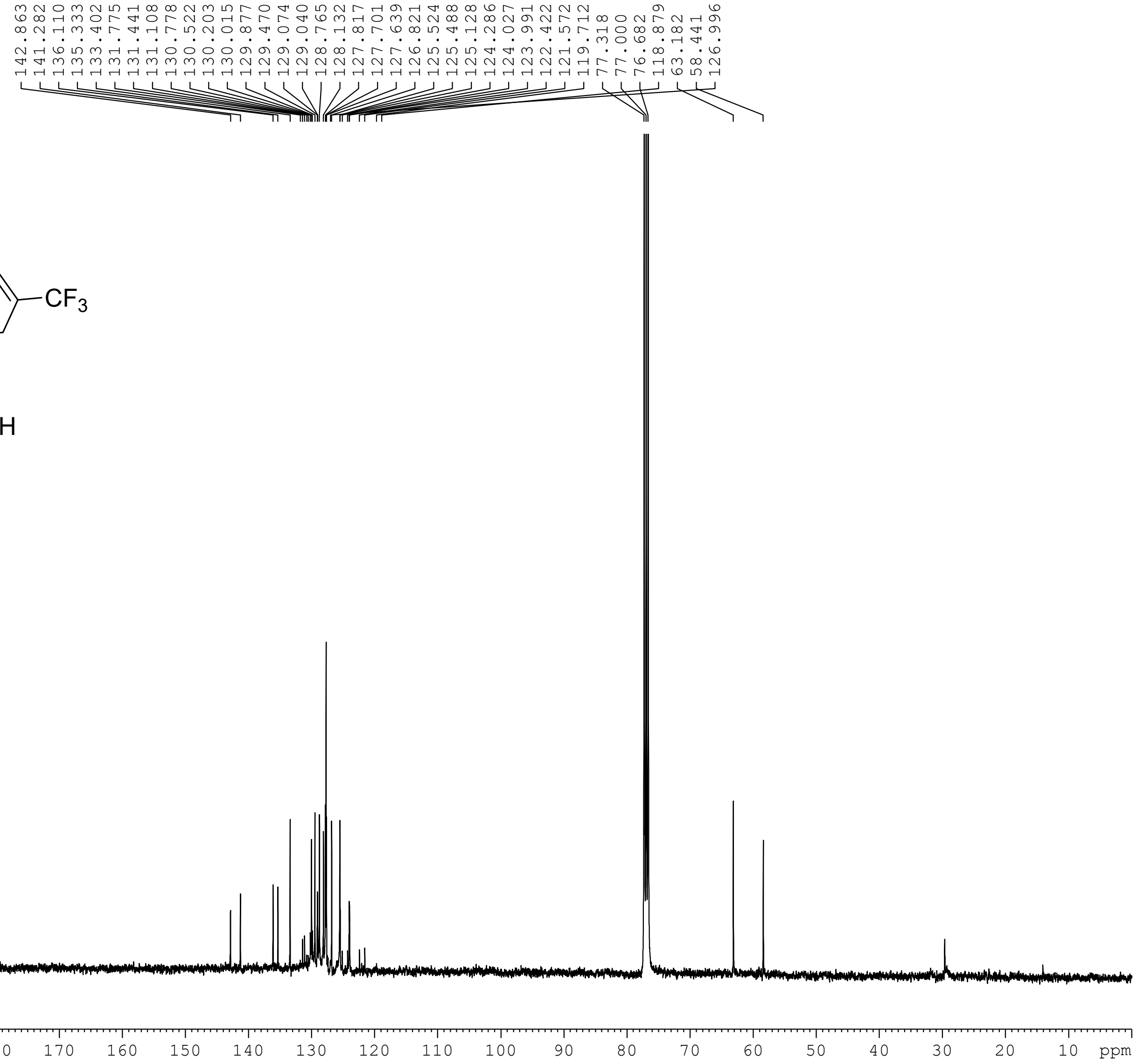


```
NAME              202408
EXPNO                481
PROCNO                 1
Date_           20240901
Time                5.25
INSTRUM            spect
PROBHD   5 mm PABBO BB/
PULPROG          zgpg30
TD                32768
SOLVENT            CDCl3
NS                10000
DS                     0
SWH           24038.461 Hz
FIDRES         0.733596 Hz
AQ            0.6816244 sec
RG                205.92
DW                20.800 usec
DE                  6.50 usec
TE                 292.2 K
D1            2.00000000 sec
D11           0.03000000 sec
TD0                    1

======== CHANNEL f1 ========
SFO1        100.6233329 MHz
NUC1                 13C
P1                 10.00 usec
SI                 32768
SF          100.6127721 MHz
WDW                   EM
SSB                    0
LB                  2.00 Hz
GB                     0
PC                  1.00
```

^{19}F NMR of **1dh** ($CDCl_3$, 376 MHz)

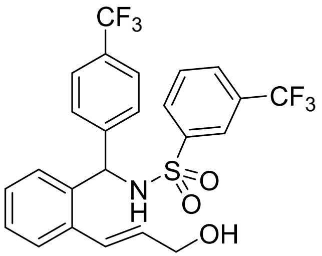

1dh

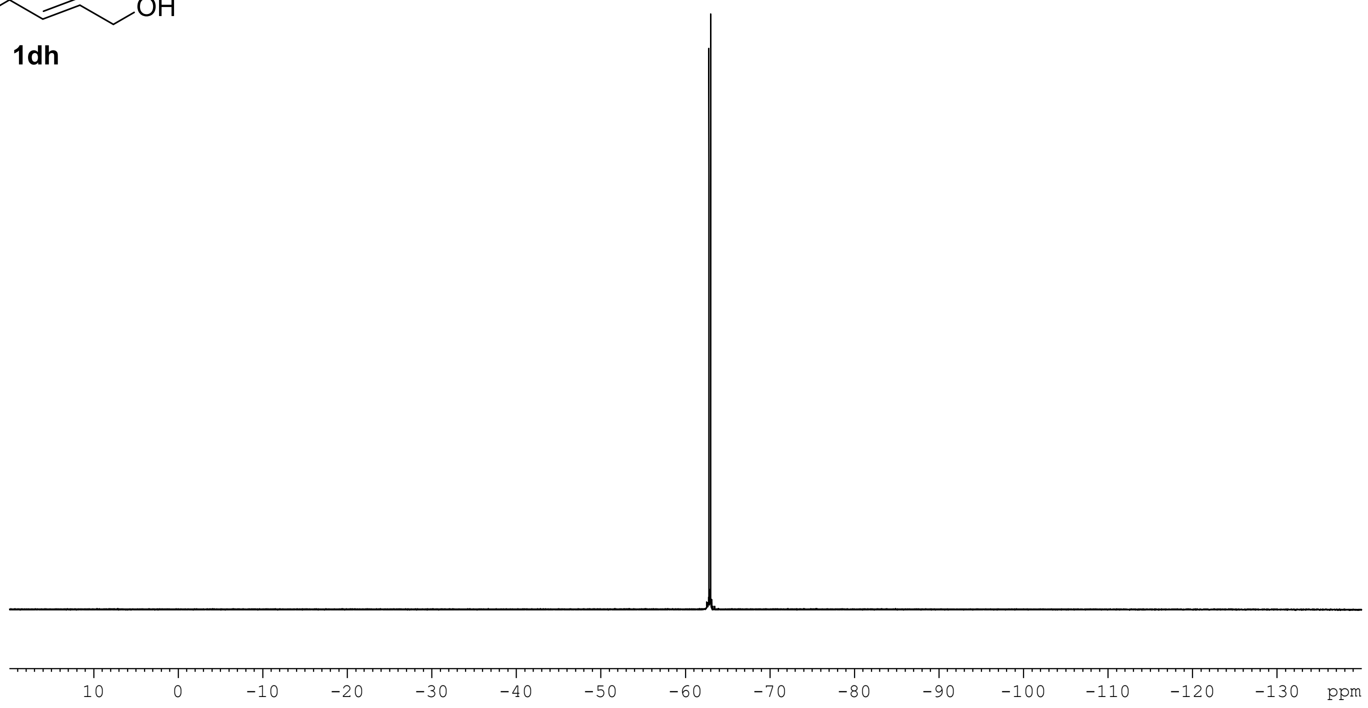


```
NAME              202408
EXPNO                414
PROCNO                 1
Date_           20240826
Time               10.35
INSTRUM            spect
PROBHD   5 mm PABBO BB/
PULPROG             zg30
TD                131072
SOLVENT            CDCl3
NS                    22
DS                     0
SWH           89285.711 Hz
FIDRES         0.681196 Hz
AQ            0.7340532 sec
RG                205.92
DW                 5.600 usec
DE                  6.50 usec
TE                 296.2 K
D1            1.00000000 sec
TD0                    1

======== CHANNEL f1 ========
SFO1         376.4757776 MHz
NUC1                 19F
P1                 15.00 usec
SI                 65536
SF           376.4983662 MHz
WDW                   EM
SSB                    0
LB                  0.30 Hz
GB                     0
PC                  1.00
```

^{1}H NMR of **1eh** ($CDCl_3$, 400 MHz)

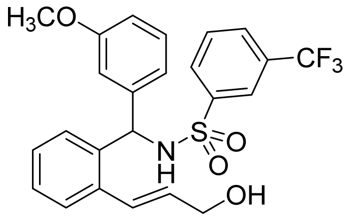

1eh

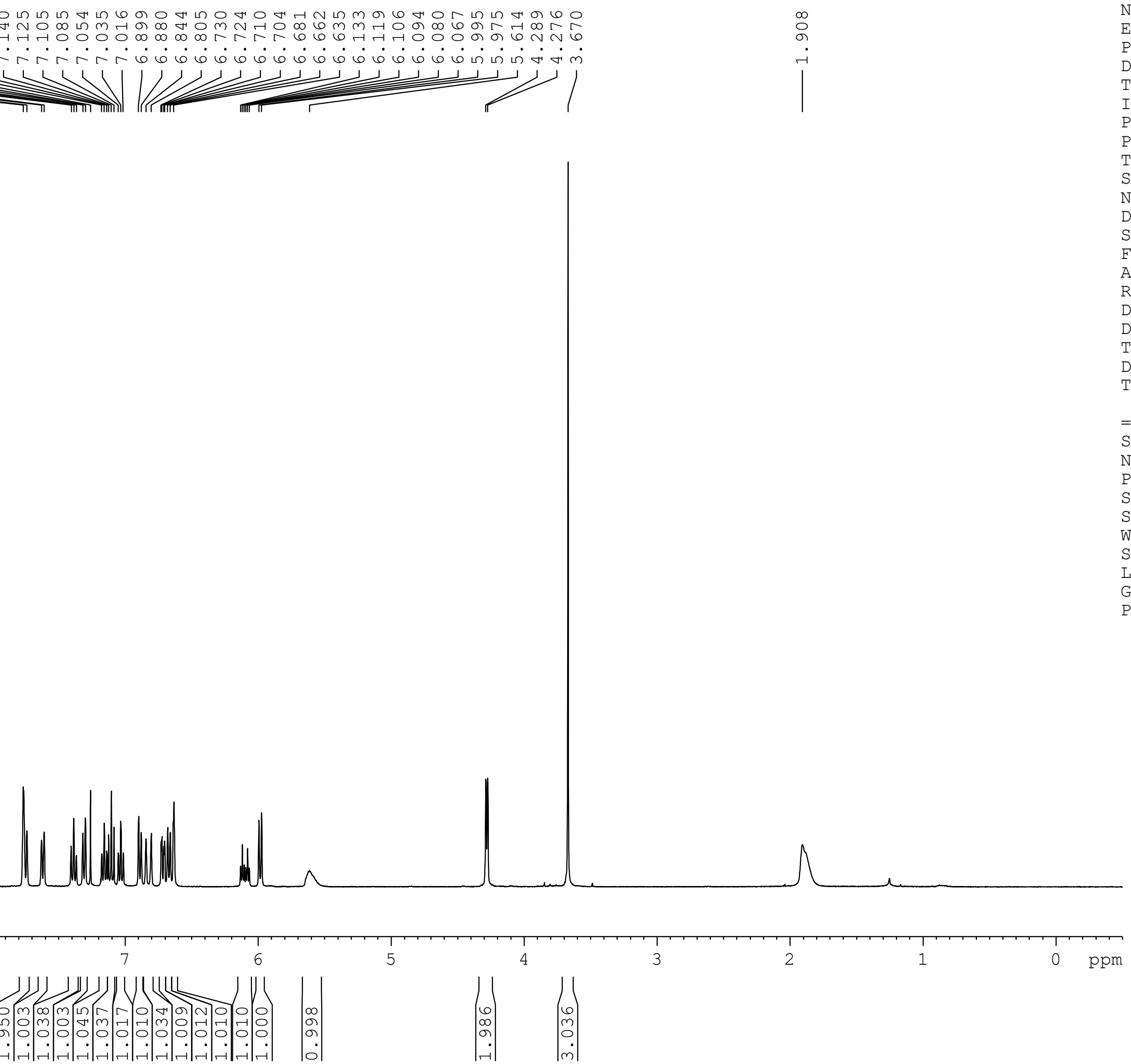

NAME	202408
EXPNO	38
PROCNO	1
Date_	20240802
Time	11.39
INSTRUM	spect
PROBHD	5 mm PABBO BB/
PULPROG	zg30
TD	32768
SOLVENT	CDCl3
NS	12
DS	0
SWH	8012.820 Hz
FIDRES	0.244532 Hz
AQ	2.0447731 sec
RG	181.8
DW	62.400 usec
DE	16.53 usec
TE	291.5 K
D1	2.00000000 sec
TD0	1
======== CHANNEL f1 ========	
SFO1	400.1324008 MHz
NUC1	1H
P1	14.00 usec
SI	16384
SF	400.1300102 MHz
WDW	EM
SSB	0
LB	0.00 Hz
GB	0
PC	1.00

$^{13}C\{^1H\}$ NMR of **1eh** ($CDCl_3$, 101 MHz)

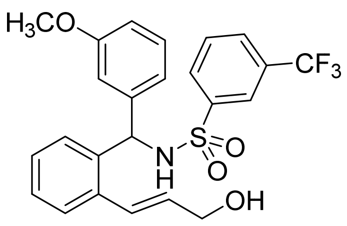

1eh

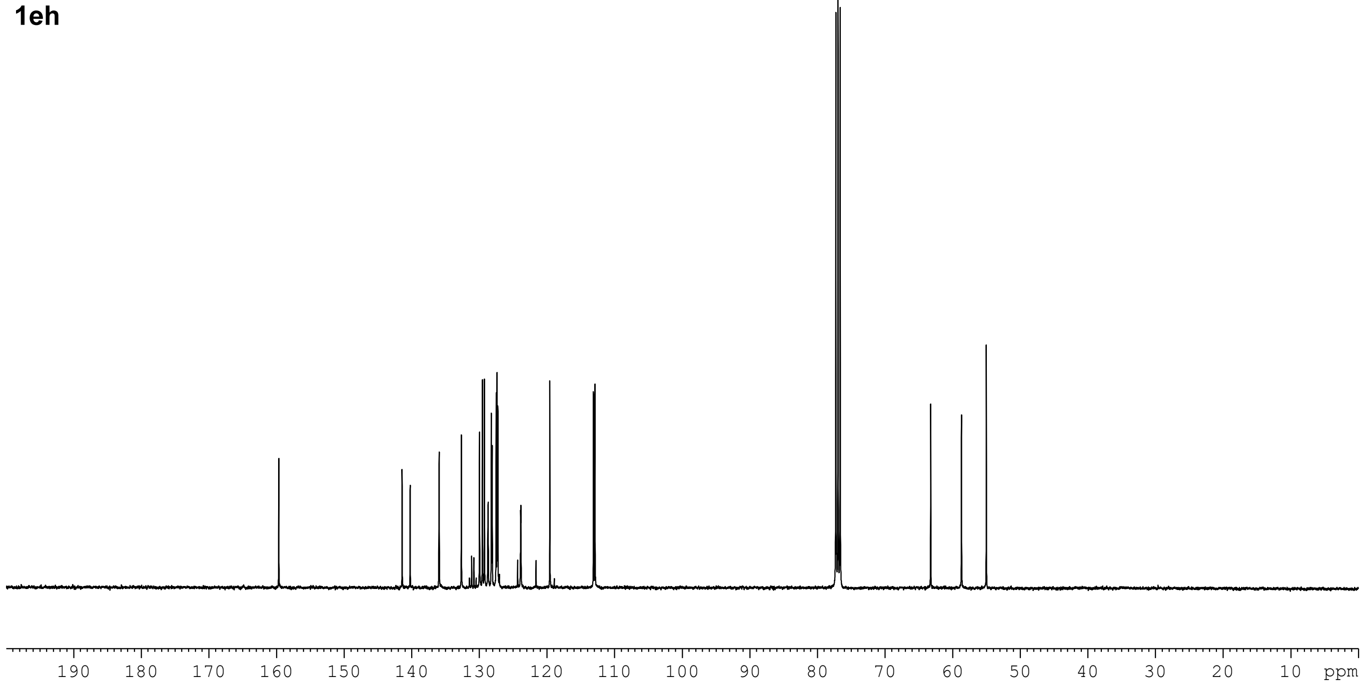

NAME	202408	
EXPNO	33	
PROCNO	1	
Date_	20240801	
Time	23.30	
INSTRUM	spect	
PROBHD	5 mm PABBO BB/	
PULPROG	zgpg30	
TD	32768	
SOLVENT	CDCl3	
NS	3800	
DS	0	
SWH	24038.461	Hz
FIDRES	0.733596	Hz
AQ	0.6816244	sec
RG	205.92	
DW	20.800	usec
DE	6.50	usec
TE	293.3	K
D1	2.00000000	sec
D11	0.03000000	sec
TD0	1	

======== CHANNEL f1 ========

SFO1	100.6233329	MHz
NUC1	13C	
P1	10.00	usec
SI	32768	
SF	100.6127742	MHz
WDW	EM	
SSB	0	
LB	2.00	Hz
GB	0	
PC	1.00	

^{19}F NMR of **1eh** ($CDCl_3$, 376 MHz)

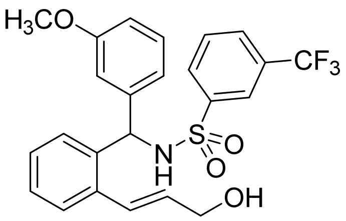

1eh

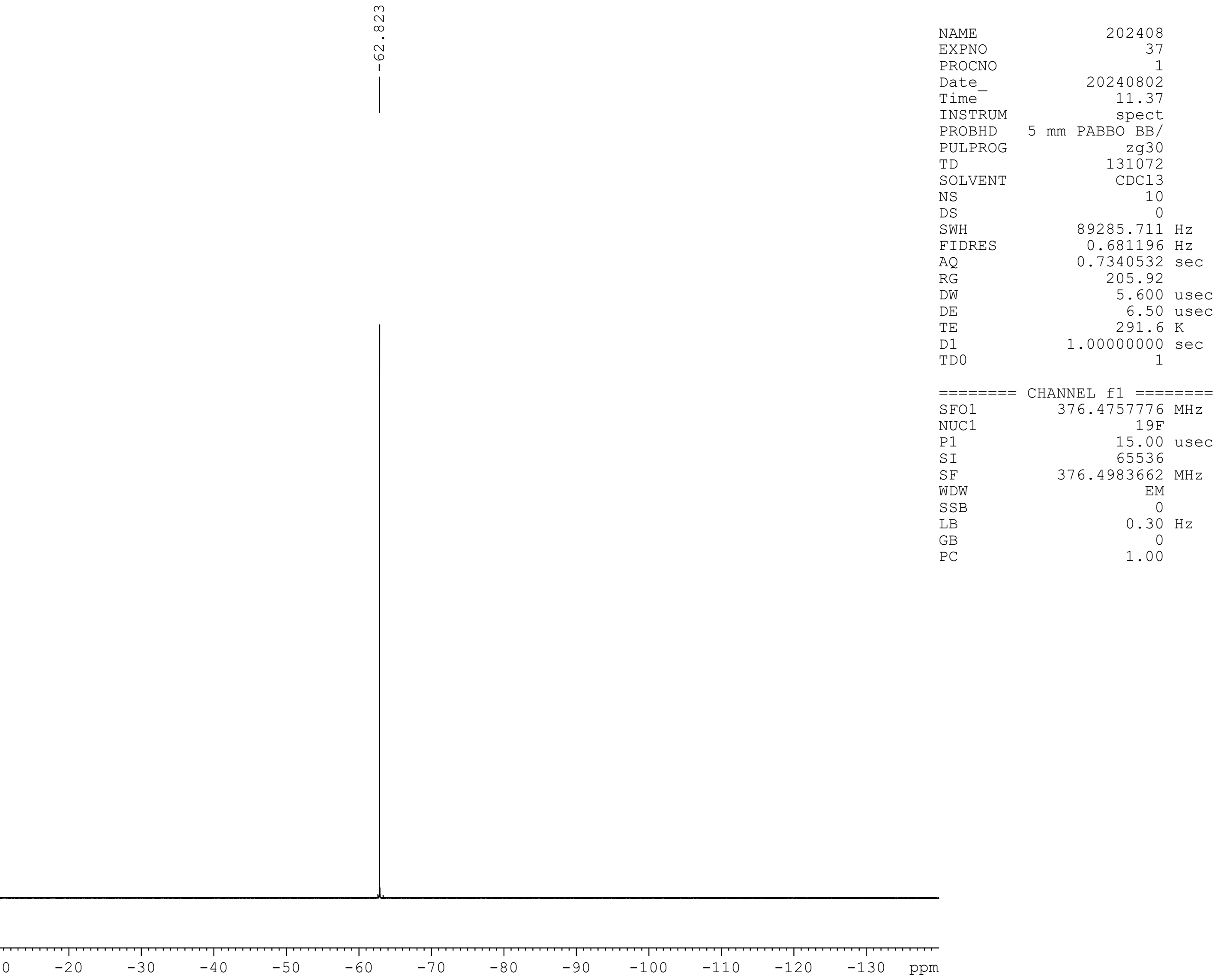

^{1}H NMR of **1fh** (CD_3OD, 400 MHz)

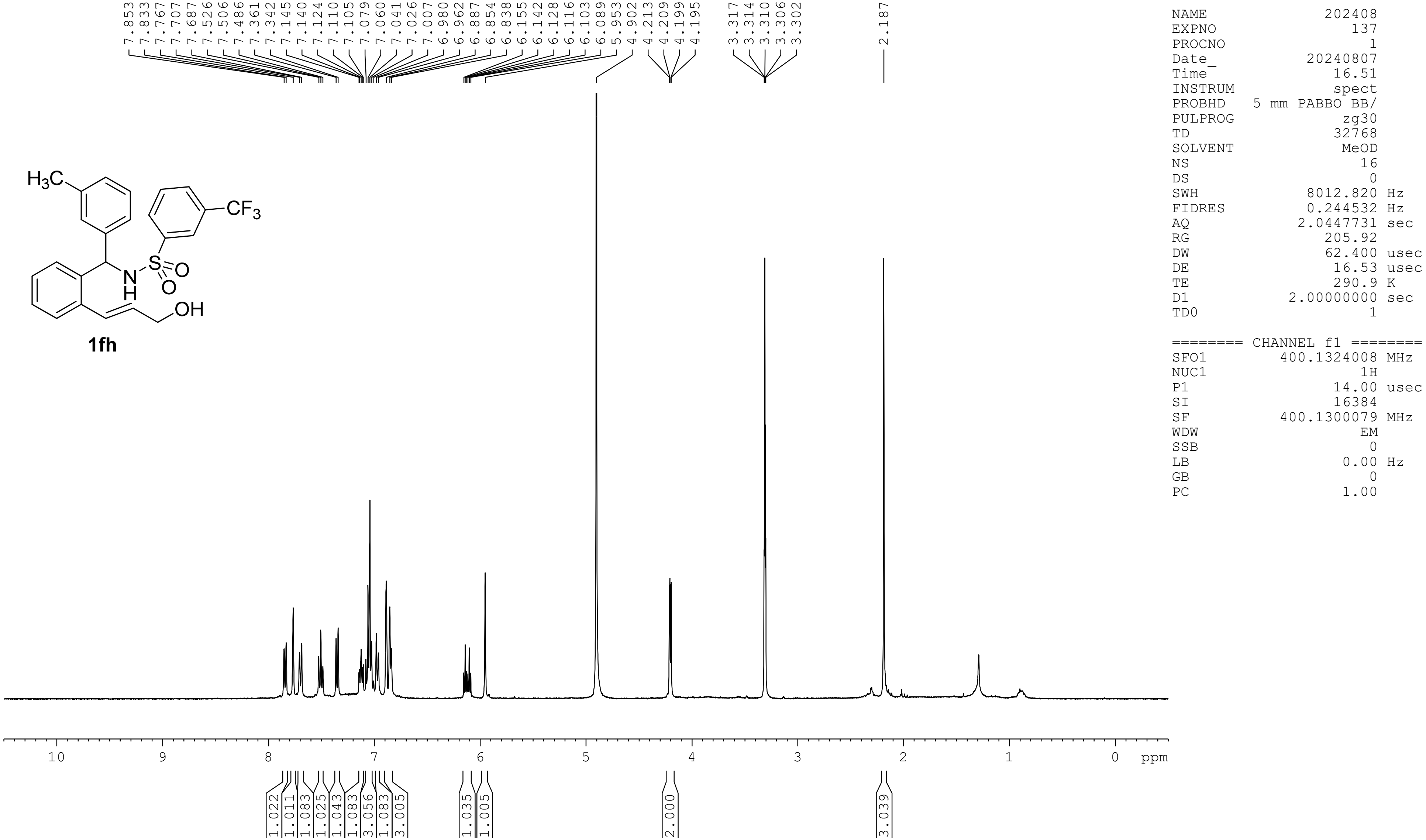

$^{13}C\{^1H\}$ NMR of **1fh** ($CDCl_3$, 101 MHz)

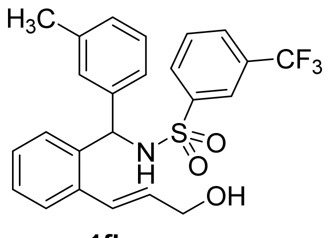

1fh

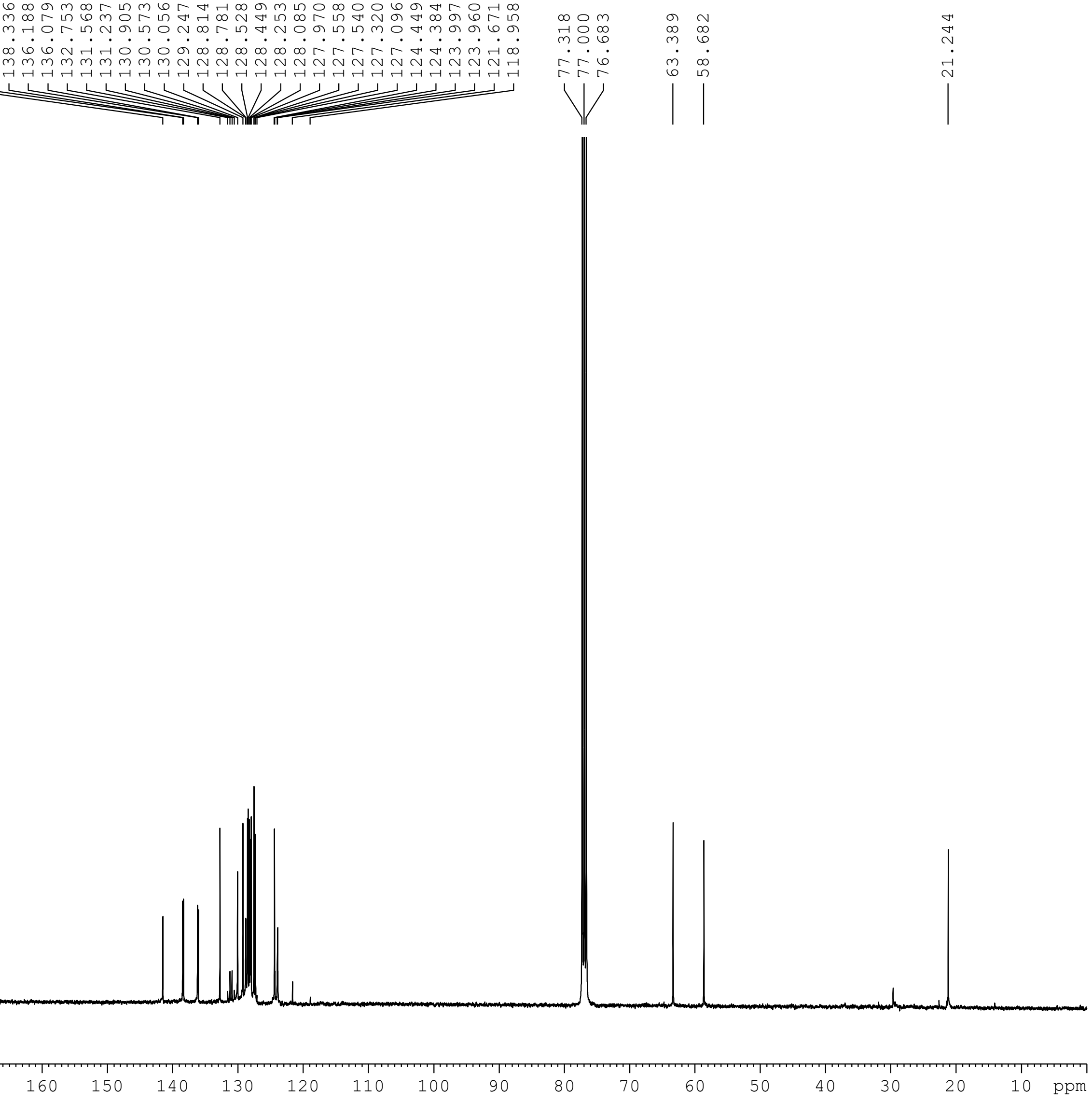

NAME	202408
EXPNO	61
PROCNO	1
Date_	20240802
Time	20.07
INSTRUM	spect
PROBHD	5 mm PABBO BB/
PULPROG	zgpg30
TD	32768
SOLVENT	CDCl3
NS	10987
DS	0
SWH	24038.461 Hz
FIDRES	0.733596 Hz
AQ	0.6816244 sec
RG	205.92
DW	20.800 usec
DE	6.50 usec
TE	291.1 K
D1	2.00000000 sec
D11	0.03000000 sec
TD0	1
======== CHANNEL f1 ========	
SFO1	100.6233329 MHz
NUC1	13C
P1	10.00 usec
SI	32768
SF	100.6127730 MHz
WDW	EM
SSB	0
LB	2.00 Hz
GB	0
PC	1.00

^{19}F NMR of **1fh** ($CDCl_3$, 376 MHz)

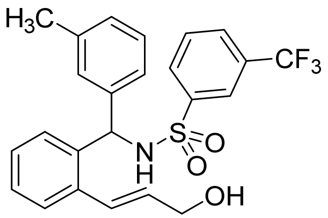

1fh

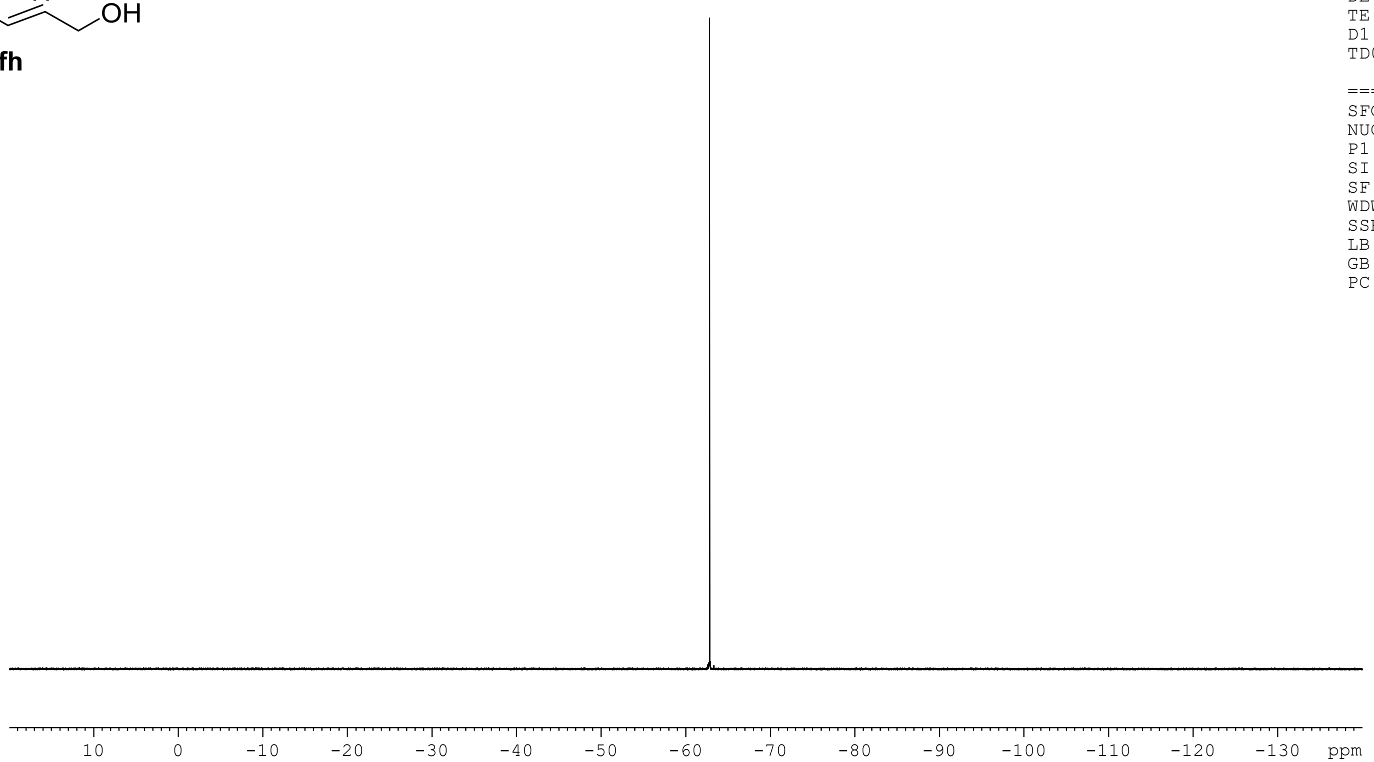

NAME	202408	
EXPNO	60	
PROCNO	1	
Date_	20240802	
Time	19.05	
INSTRUM	spect	
PROBHD	5 mm PABBO BB/	
PULPROG	zg30	
TD	131072	
SOLVENT	CDCl3	
NS	3	
DS	0	
SWH	89285.711	Hz
FIDRES	0.681196	Hz
AQ	0.7340532	sec
RG	205.92	
DW	5.600	usec
DE	6.50	usec
TE	290.9	K
D1	1.00000000	sec
TD0	1	

======== CHANNEL f1 ========

SFO1	376.4757776	MHz
NUC1	19F	
P1	15.00	usec
SI	65536	
SF	376.4983662	MHz
WDW	EM	
SSB	0	
LB	0.30	Hz
GB	0	
PC	1.00	

^{1}H NMR of **1gh** ($CDCl_3$, 400 MHz)

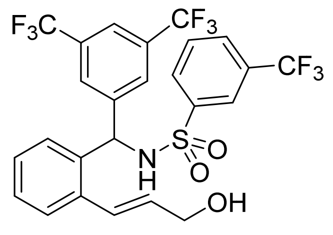

1gh

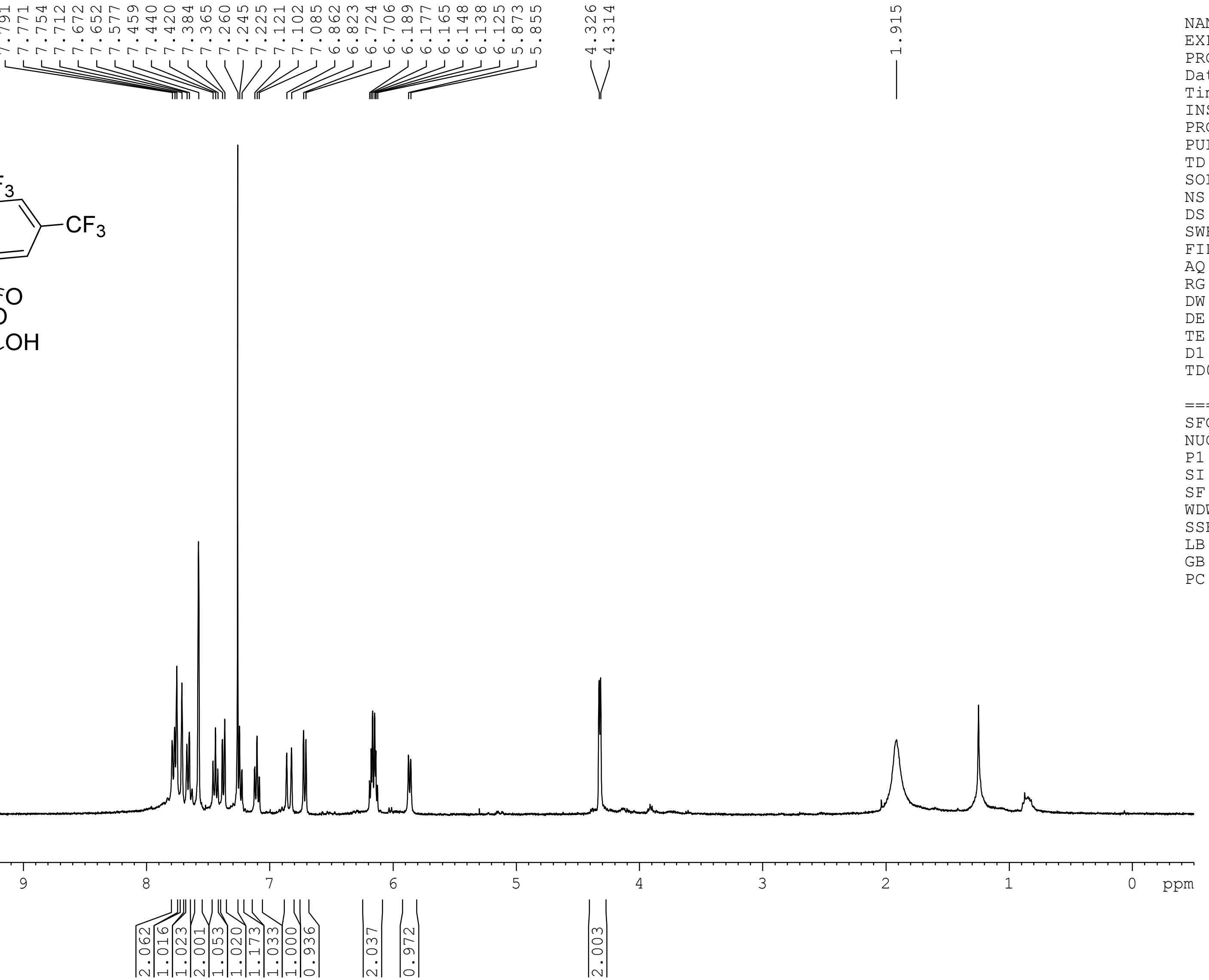

NAME 202407
EXPNO 399
PROCNO 1
Date_ 20240718
Time 17.12
INSTRUM spect
PROBHD 5 mm PABBO BB/
PULPROG zg30
TD 32768
SOLVENT CDCl3
NS 4
DS 0
SWH 8012.820 Hz
FIDRES 0.244532 Hz
AQ 2.0447731 sec
RG 205.92
DW 62.400 usec
DE 16.53 usec
TE 291.5 K
D1 2.00000000 sec
TD0 1

======== CHANNEL f1 ========
SFO1 400.1324008 MHz
NUC1 1H
P1 14.00 usec
SI 16384
SF 400.1300101 MHz
WDW EM
SSB 0
LB 0.00 Hz
GB 0
PC 1.00

$^{13}C\{^1H\}$ NMR of **1gh** ($CDCl_3$, 101 MHz)

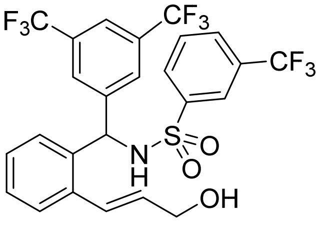

1gh

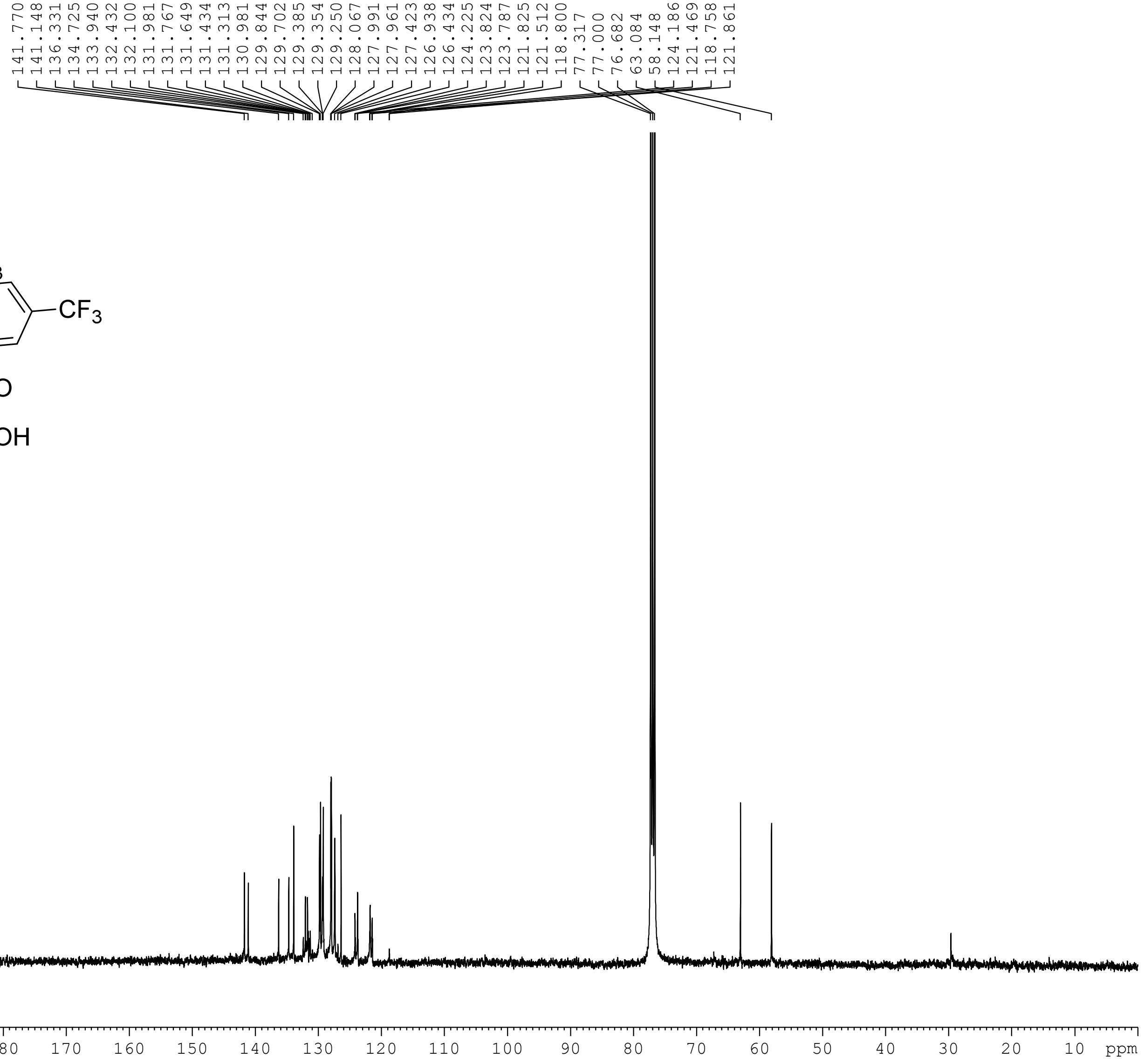

NAME	202407
EXPNO	411
PROCNO	1
Date_	20240719
Time	9.01
INSTRUM	spect
PROBHD	5 mm PABBO BB/
PULPROG	zgpg30
TD	32768
SOLVENT	CDCl3
NS	7500
DS	0
SWH	24038.461 Hz
FIDRES	0.733596 Hz
AQ	0.6816244 sec
RG	205.92
DW	20.800 usec
DE	6.50 usec
TE	293.3 K
D1	2.00000000 sec
D11	0.03000000 sec
TD0	1

======== CHANNEL f1 ========

SFO1	100.6233329 MHz
NUC1	13C
P1	10.00 usec
SI	32768
SF	100.6127719 MHz
WDW	EM
SSB	0
LB	2.00 Hz
GB	0
PC	1.00

^{19}F NMR of **1gh** ($CDCl_3$, 376 MHz)

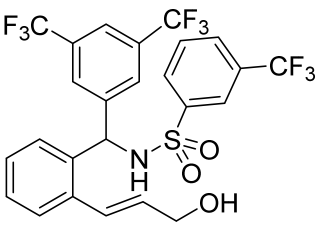

1gh

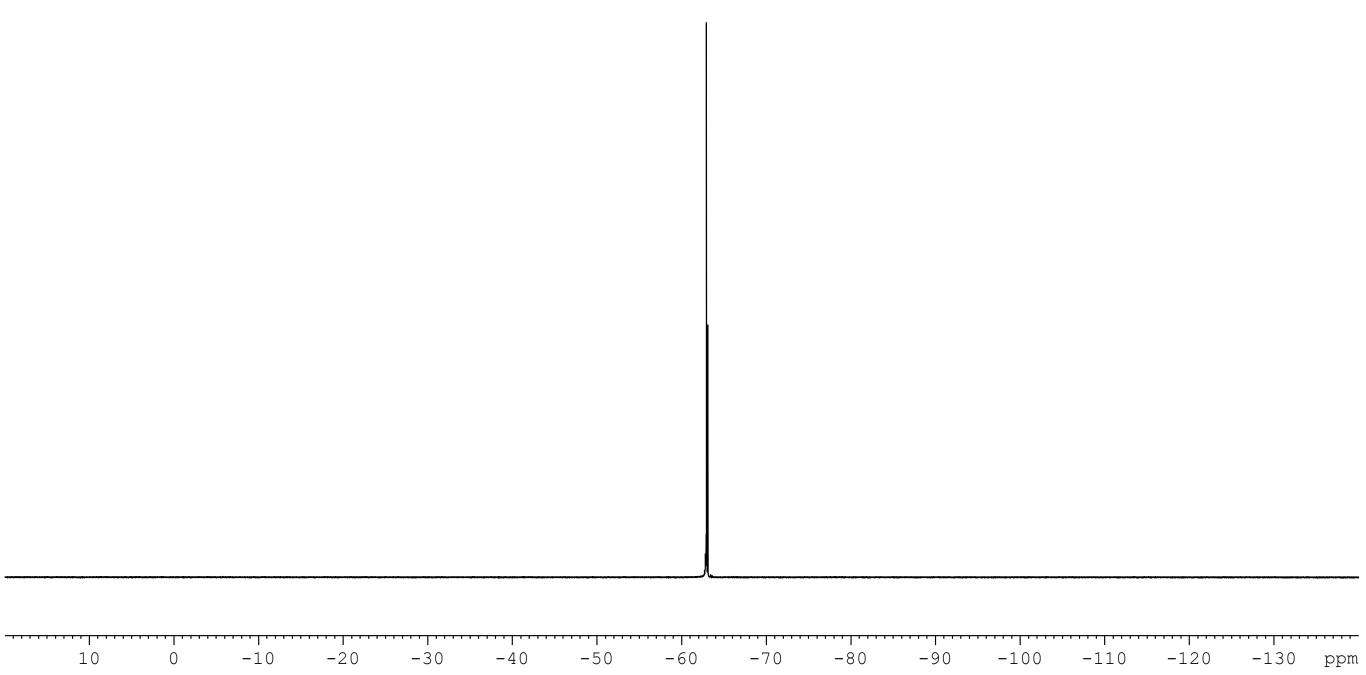

NAME	202407	
EXPNO	400	
PROCNO	1	
Date_	20240718	
Time	17.15	
INSTRUM	spect	
PROBHD	5 mm PABBO BB/	
PULPROG	zg30	
TD	131072	
SOLVENT	CDCl3	
NS	5	
DS	0	
SWH	89285.711	Hz
FIDRES	0.681196	Hz
AQ	0.7340532	sec
RG	205.92	
DW	5.600	usec
DE	6.50	usec
TE	291.5	K
D1	1.00000000	sec
TD0	1	
======== CHANNEL f1 ========		
SFO1	376.4757776	MHz
NUC1	19F	
P1	15.00	usec
SI	65536	
SF	376.4983662	MHz
WDW	EM	
SSB	0	
LB	0.30	Hz
GB	0	
PC	1.00	

^{1}H NMR of **1hh** ($CDCl_3$, 400 MHz)

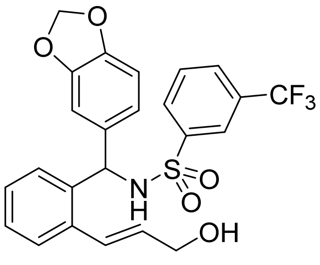

1hh

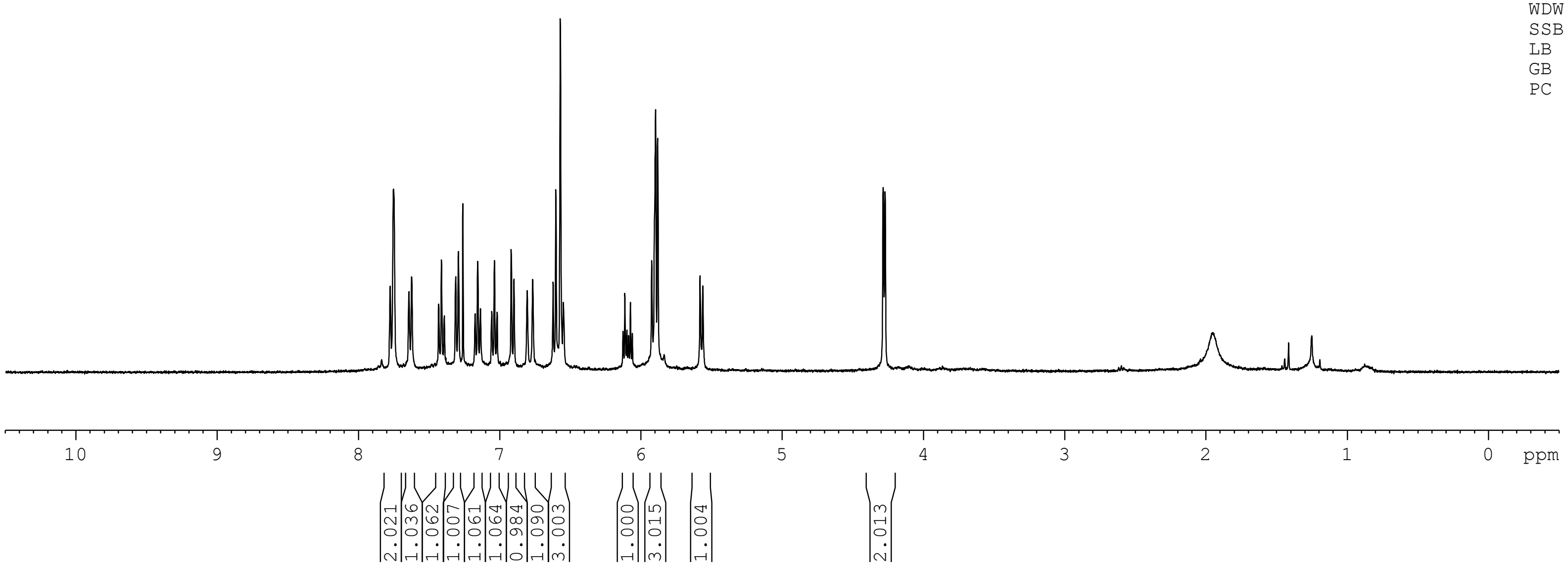

NAME	202408
EXPNO	173
PROCNO	1
Date_	20240808
Time	21.37
INSTRUM	spect
PROBHD	5 mm PABBO BB/
PULPROG	zg30
TD	32768
SOLVENT	CDCl3
NS	4
DS	0
SWH	8012.820 Hz
FIDRES	0.244532 Hz
AQ	2.0447731 sec
RG	181.8
DW	62.400 usec
DE	16.53 usec
TE	291.1 K
D1	2.00000000 sec
TD0	1

======== CHANNEL f1 ========

SFO1	400.1324008 MHz
NUC1	1H
P1	14.00 usec
SI	16384
SF	400.1300102 MHz
WDW	EM
SSB	0
LB	0.00 Hz
GB	0
PC	1.00

$^{13}C\{^{1}H\}$ NMR of **1hh** ($CDCl_3$, 101 MHz)

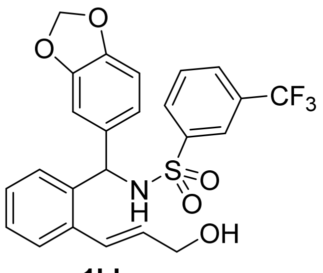

1hh

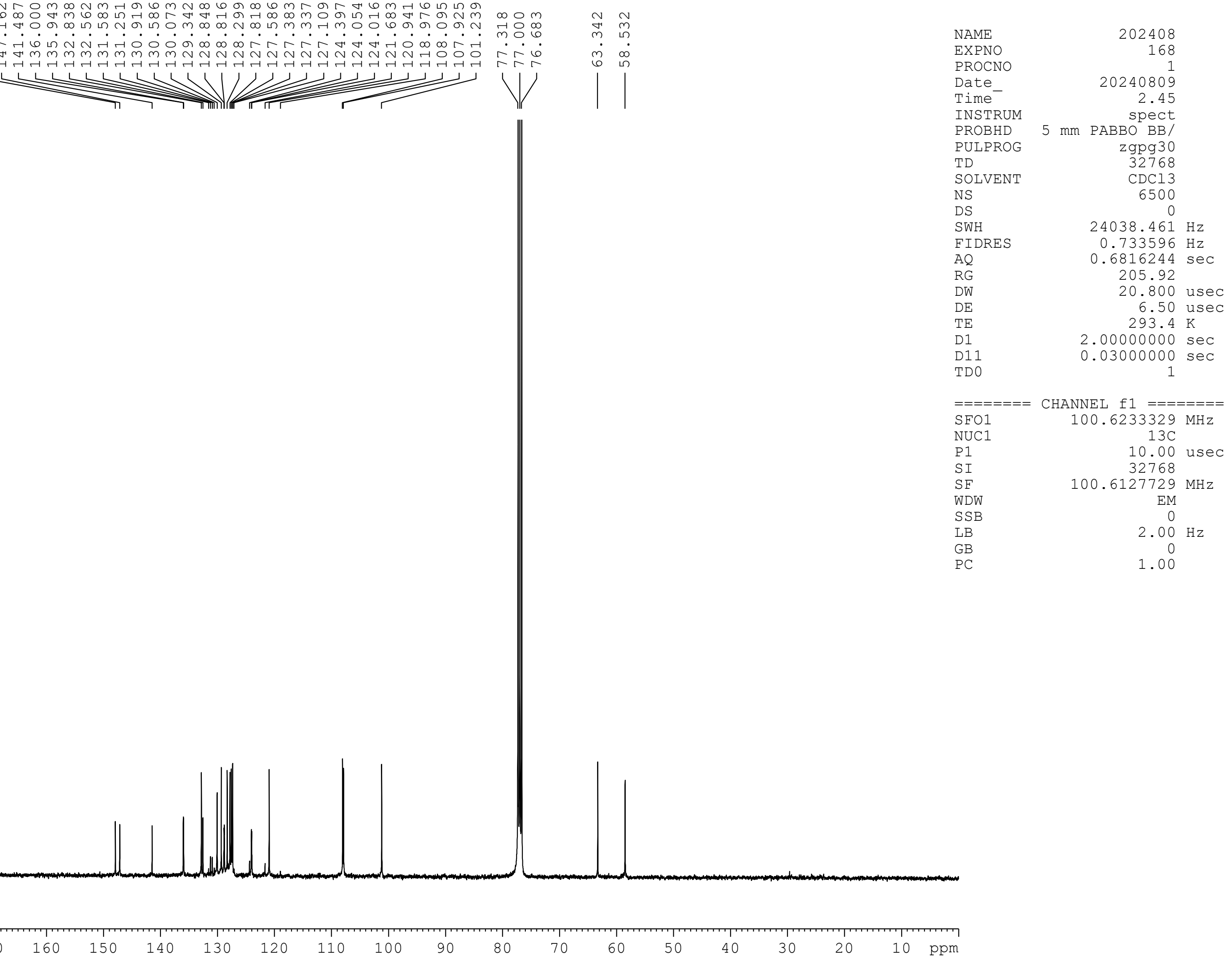

^{19}F NMR of **1hh** ($CDCl_3$, 376 MHz)

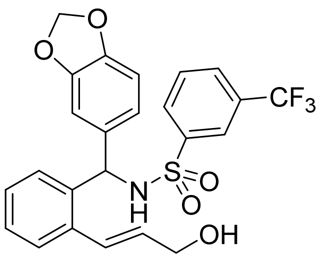

1hh

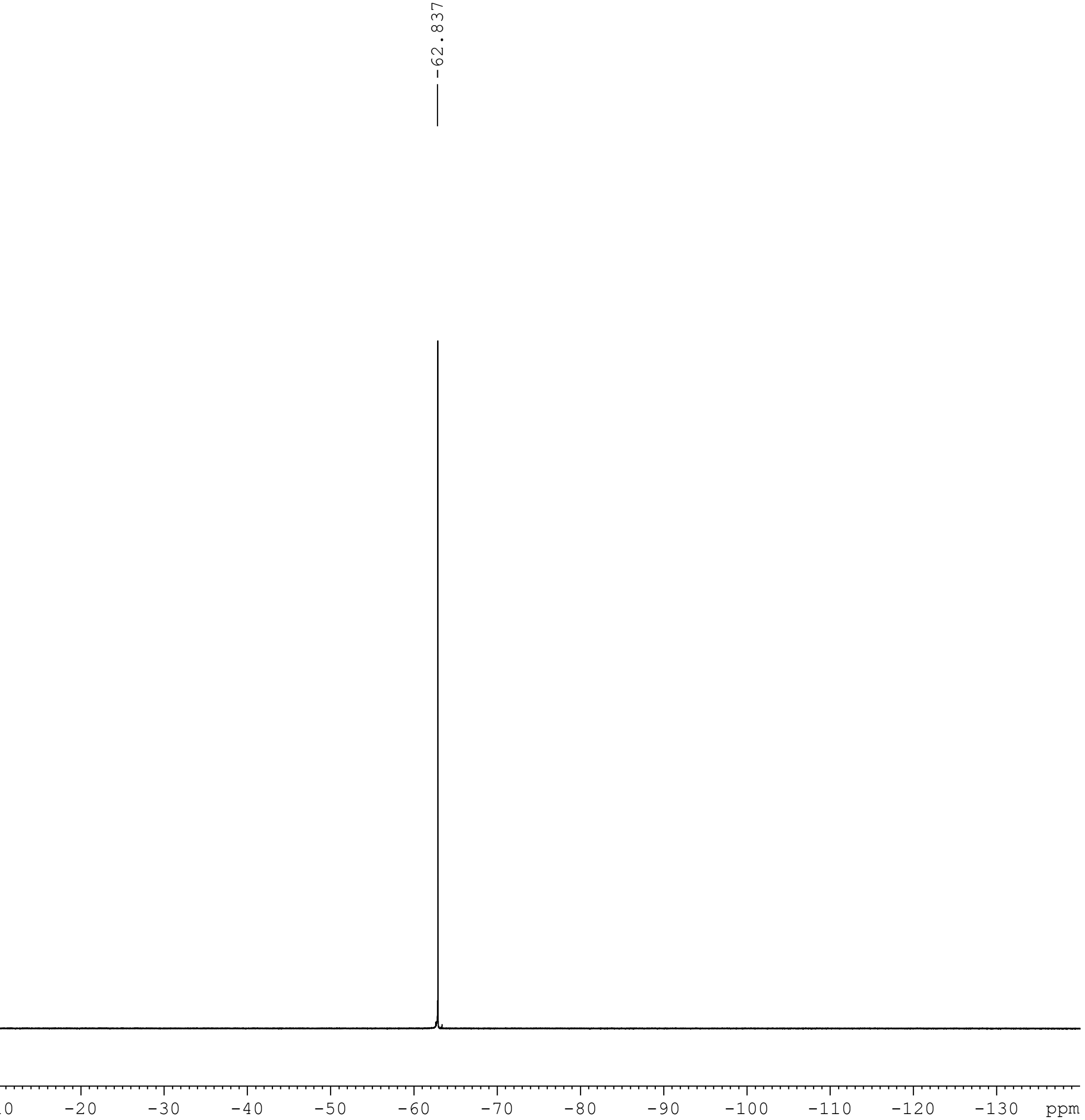

NAME	202408
EXPNO	167
PROCNO	1
Date_	20240808
Time	21.46
INSTRUM	spect
PROBHD	5 mm PABBO BB/
PULPROG	zg30
TD	131072
SOLVENT	CDCl3
NS	20
DS	0
SWH	89285.711 Hz
FIDRES	0.681196 Hz
AQ	0.7340532 sec
RG	205.92
DW	5.600 usec
DE	6.50 usec
TE	291.1 K
D1	1.00000000 sec
TD0	1

======== CHANNEL f1 ========

SFO1	376.4757776 MHz
NUC1	19F
P1	15.00 usec
SI	65536
SF	376.4983662 MHz
WDW	EM
SSB	0
LB	0.30 Hz
GB	0
PC	1.00

^{1}H NMR of **1ih** ($CDCl_3$, 400 MHz)

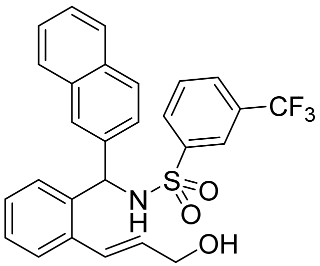

1ih

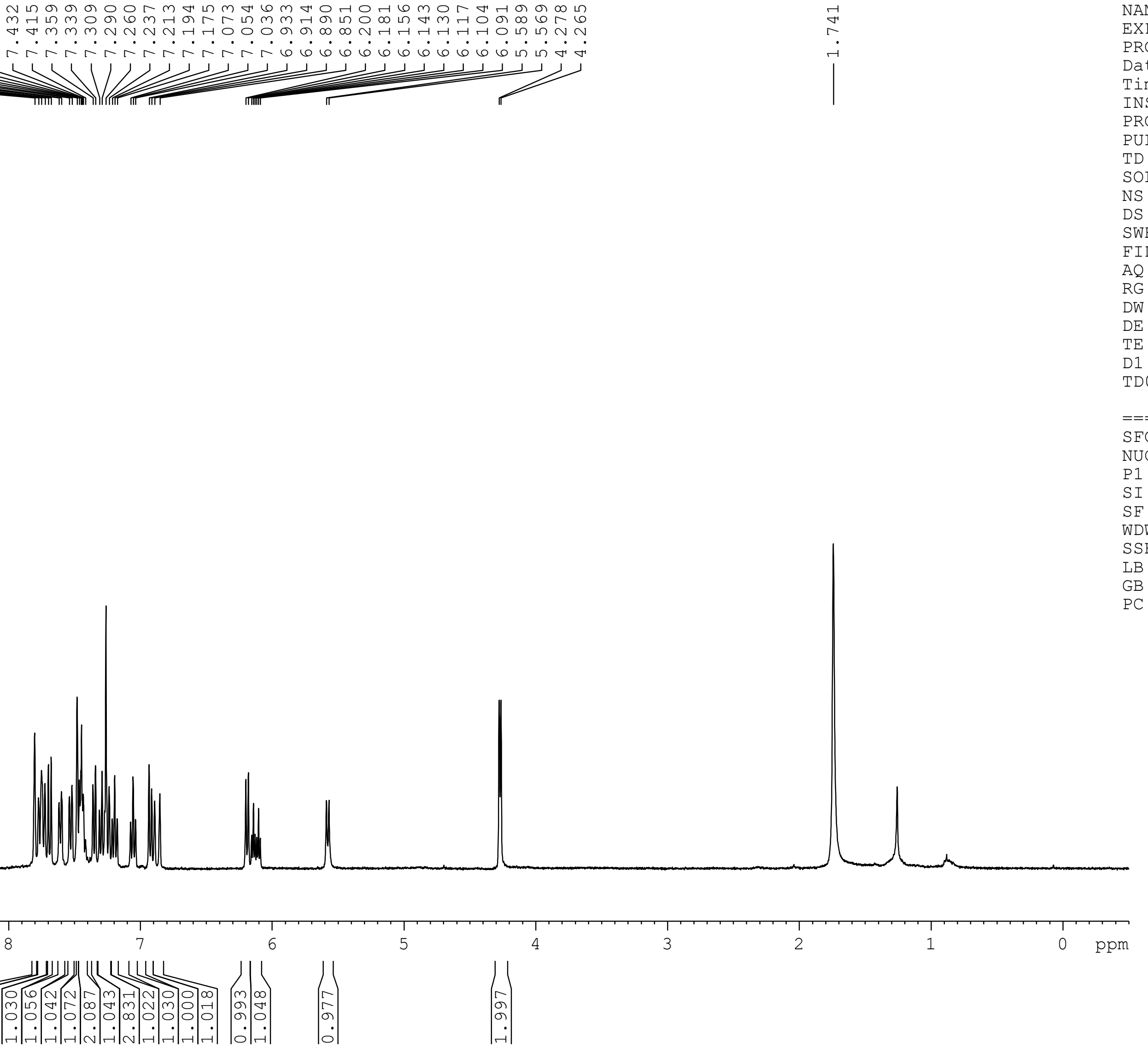

NAME 202408
EXPNO 3
PROCNO 1
Date_ 20240801
Time 10.54
INSTRUM spect
PROBHD 5 mm PABBO BB/
PULPROG zg30
TD 32768
SOLVENT CDCl3
NS 14
DS 0
SWH 8012.820 Hz
FIDRES 0.244532 Hz
AQ 2.0447731 sec
RG 205.92
DW 62.400 usec
DE 16.53 usec
TE 295.9 K
D1 2.00000000 sec
TD0 1

======== CHANNEL f1 ========
SFO1 400.1324008 MHz
NUC1 1H
P1 14.00 usec
SI 16384
SF 400.1300091 MHz
WDW EM
SSB 0
LB 0.00 Hz
GB 0
PC 1.00

$^{13}C\{^{1}H\}$ NMR of **1ih** ($CDCl_3$, 101 MHz)

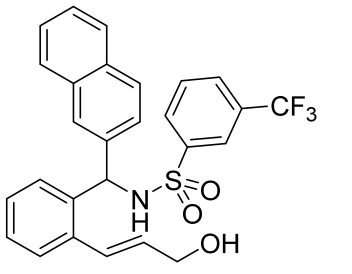

1ih

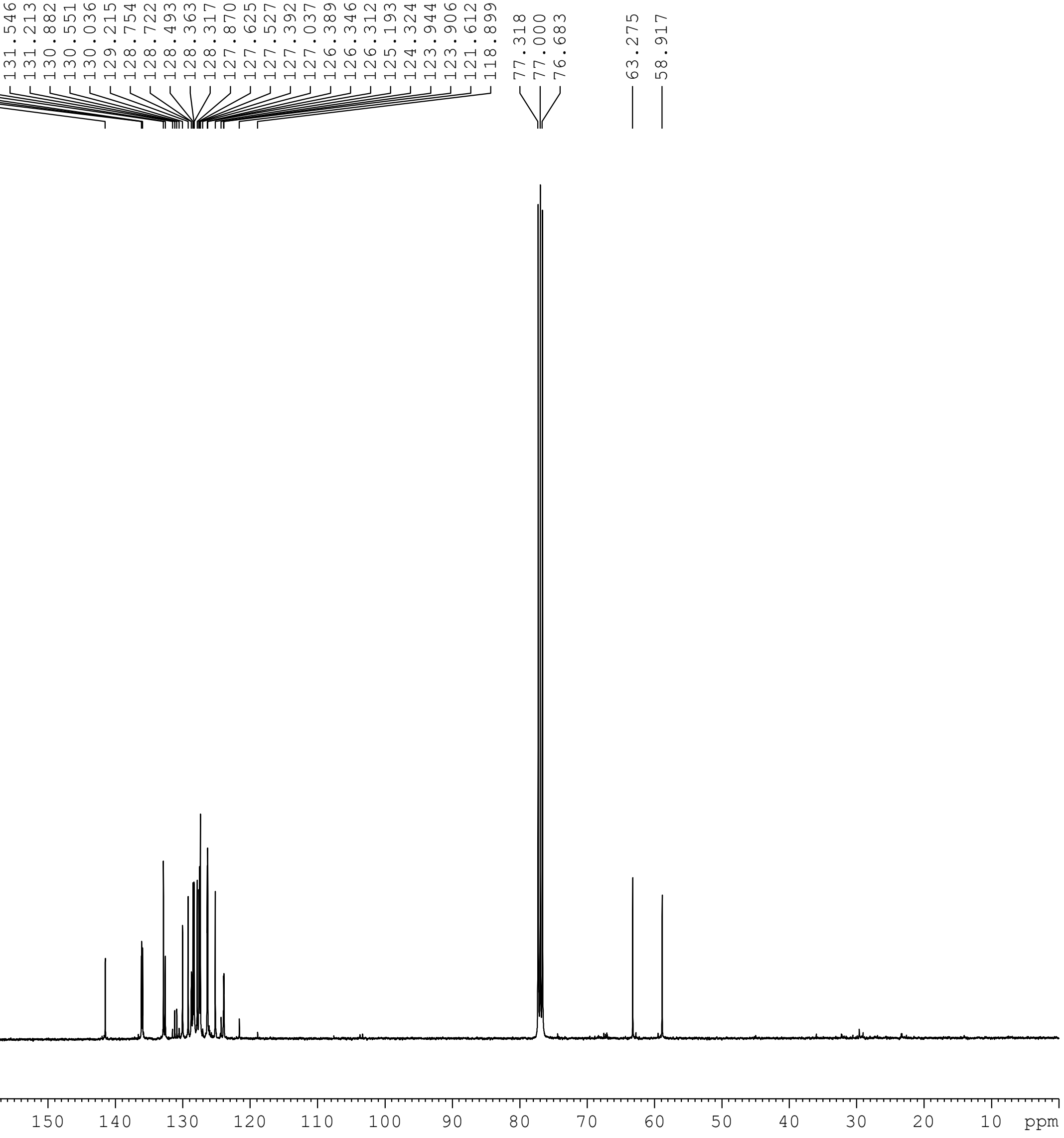

NAME 202407
EXPNO 627
PROCNO 1
Date_ 20240731
Time 21.48
INSTRUM spect
PROBHD 5 mm PABBO BB/
PULPROG zgpg30
TD 32768
SOLVENT CDCl3
NS 10000
DS 0
SWH 24038.461 Hz
FIDRES 0.733596 Hz
AQ 0.6816244 sec
RG 205.92
DW 20.800 usec
DE 6.50 usec
TE 296.2 K
D1 2.00000000 sec
D11 0.03000000 sec
TD0 1

======== CHANNEL f1 ========
SFO1 100.6233329 MHz
NUC1 13C
P1 10.00 usec
SI 32768
SF 100.6127728 MHz
WDW EM
SSB 0
LB 2.00 Hz
GB 0
PC 1.00

^{19}F NMR of **1ih** ($CDCl_3$, 376 MHz)

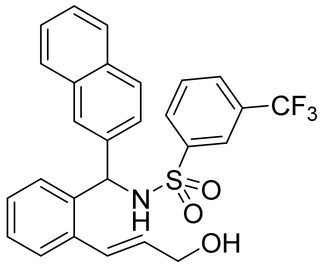

1ih

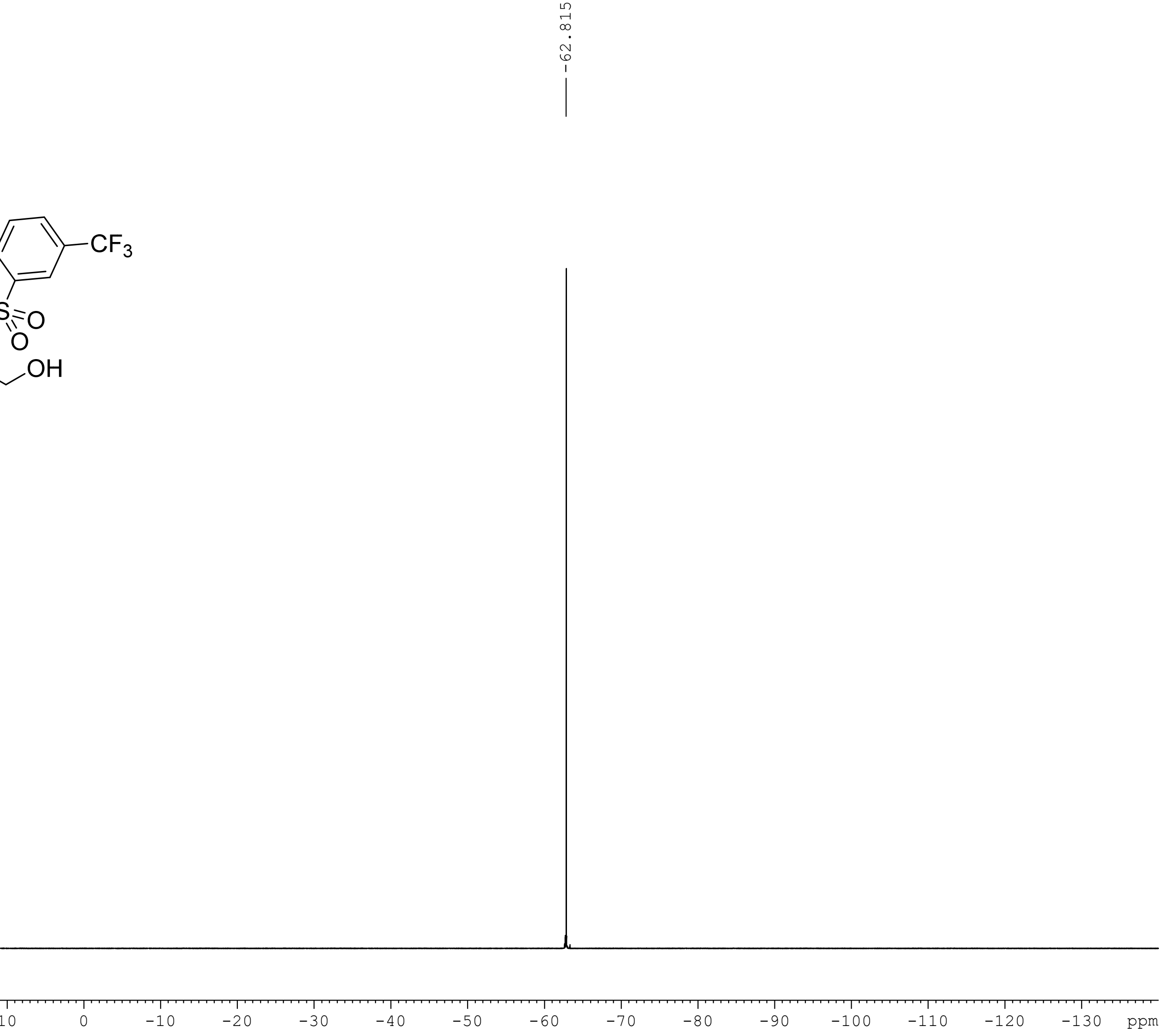

NAME	202407	
EXPNO	576	
PROCNO	1	
Date_	20240727	
Time	16.38	
INSTRUM	spect	
PROBHD	5 mm PABBO BB/	
PULPROG	zg30	
TD	131072	
SOLVENT	CDCl3	
NS	3	
DS	0	
SWH	89285.711	Hz
FIDRES	0.681196	Hz
AQ	0.7340532	sec
RG	205.92	
DW	5.600	usec
DE	6.50	usec
TE	293.4	K
D1	1.00000000	sec
TD0	1	

======== CHANNEL f1 ========

SFO1	376.4757776	MHz
NUC1	19F	
P1	15.00	usec
SI	65536	
SF	376.4983662	MHz
WDW	EM	
SSB	0	
LB	0.30	Hz
GB	0	
PC	1.00	

^{1}H NMR of **1jh** ($CDCl_3$, 400 MHz)

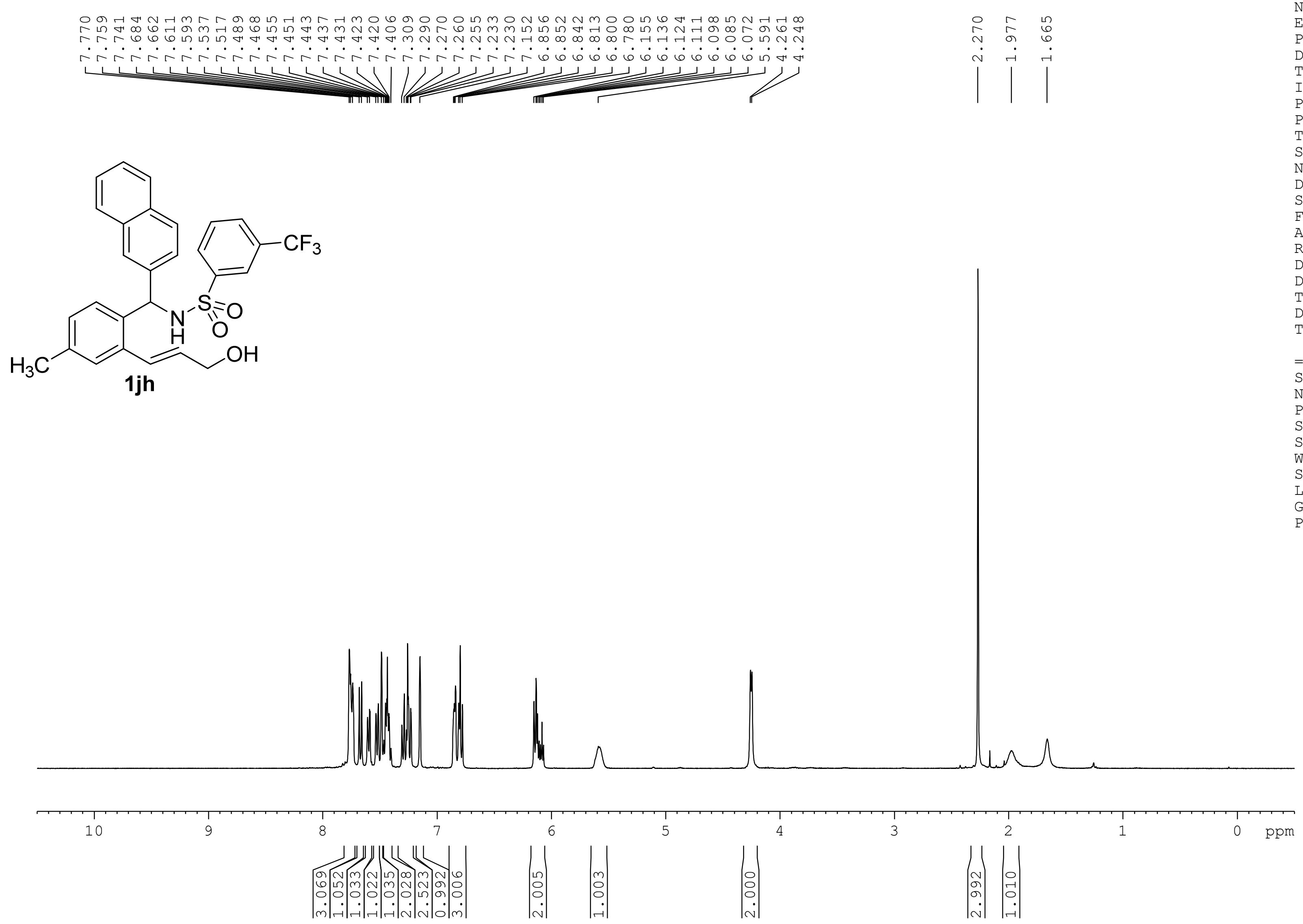

NAME	202408
EXPNO	416
PROCNO	1
Date_	20240826
Time	10.56
INSTRUM	spect
PROBHD	5 mm PABBO BB/
PULPROG	zg30
TD	32768
SOLVENT	CDCl3
NS	20
DS	0
SWH	8012.820 Hz
FIDRES	0.244532 Hz
AQ	2.0447731 sec
RG	205.92
DW	62.400 usec
DE	16.53 usec
TE	295.9 K
D1	2.00000000 sec
TD0	1
======== CHANNEL f1 ========	
SFO1	400.1324008 MHz
NUC1	1H
P1	14.00 usec
SI	16384
SF	400.1300094 MHz
WDW	EM
SSB	0
LB	0.00 Hz
GB	0
PC	1.00

$^{13}C\{^{1}H\}$ NMR of **1jh** ($CDCl_3$, 101 MHz)

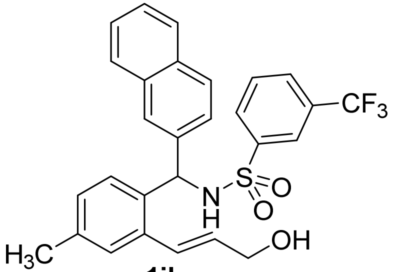

1jh

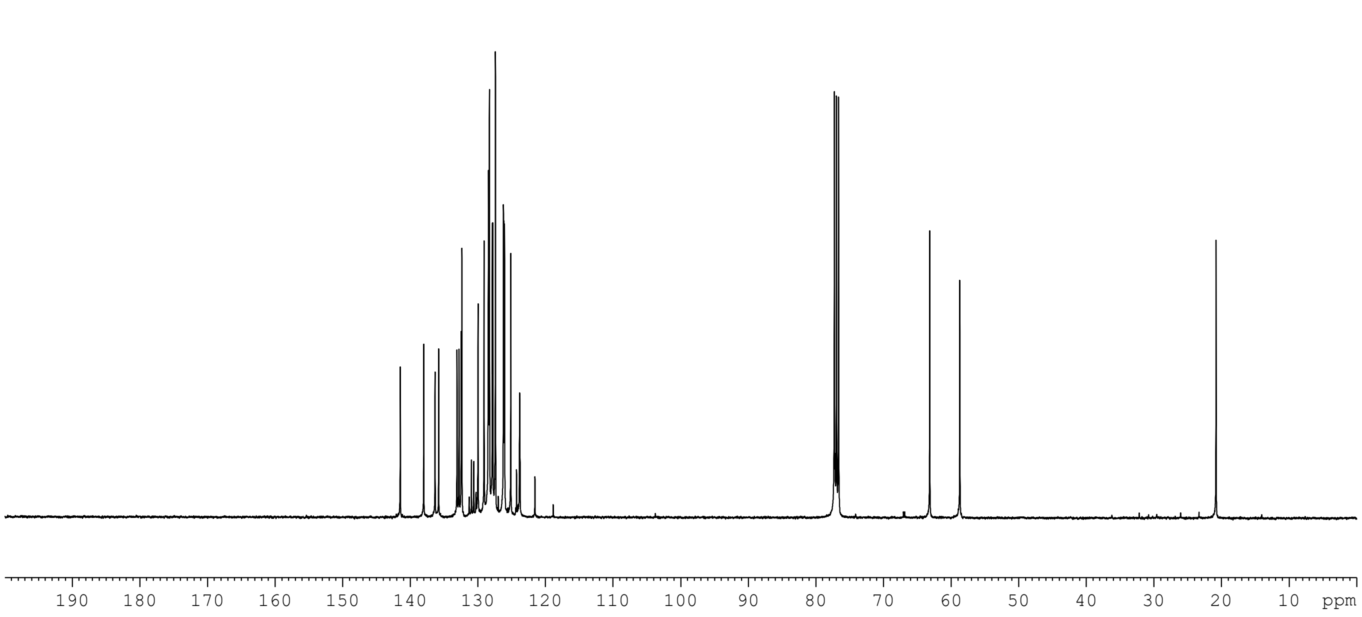

NAME	202408	
EXPNO	411	
PROCNO	1	
Date_	20240826	
Time	0.58	
INSTRUM	spect	
PROBHD	5 mm PABBO BB/	
PULPROG	zgpg30	
TD	32768	
SOLVENT	CDCl3	
NS	8001	
DS	0	
SWH	24038.461	Hz
FIDRES	0.733596	Hz
AQ	0.6816244	sec
RG	205.92	
DW	20.800	usec
DE	6.50	usec
TE	294.1	K
D1	2.00000000	sec
D11	0.03000000	sec
TD0	1	

======== CHANNEL f1 ========

SFO1	100.6233329	MHz
NUC1	13C	
P1	10.00	usec
SI	32768	
SF	100.6127790	MHz
WDW	EM	
SSB	0	
LB	2.00	Hz
GB	0	
PC	1.00	

^{19}F NMR of **1jh** ($CDCl_3$, 376 MHz)

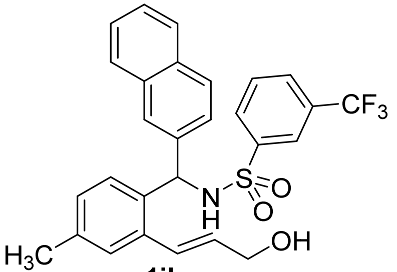

1jh

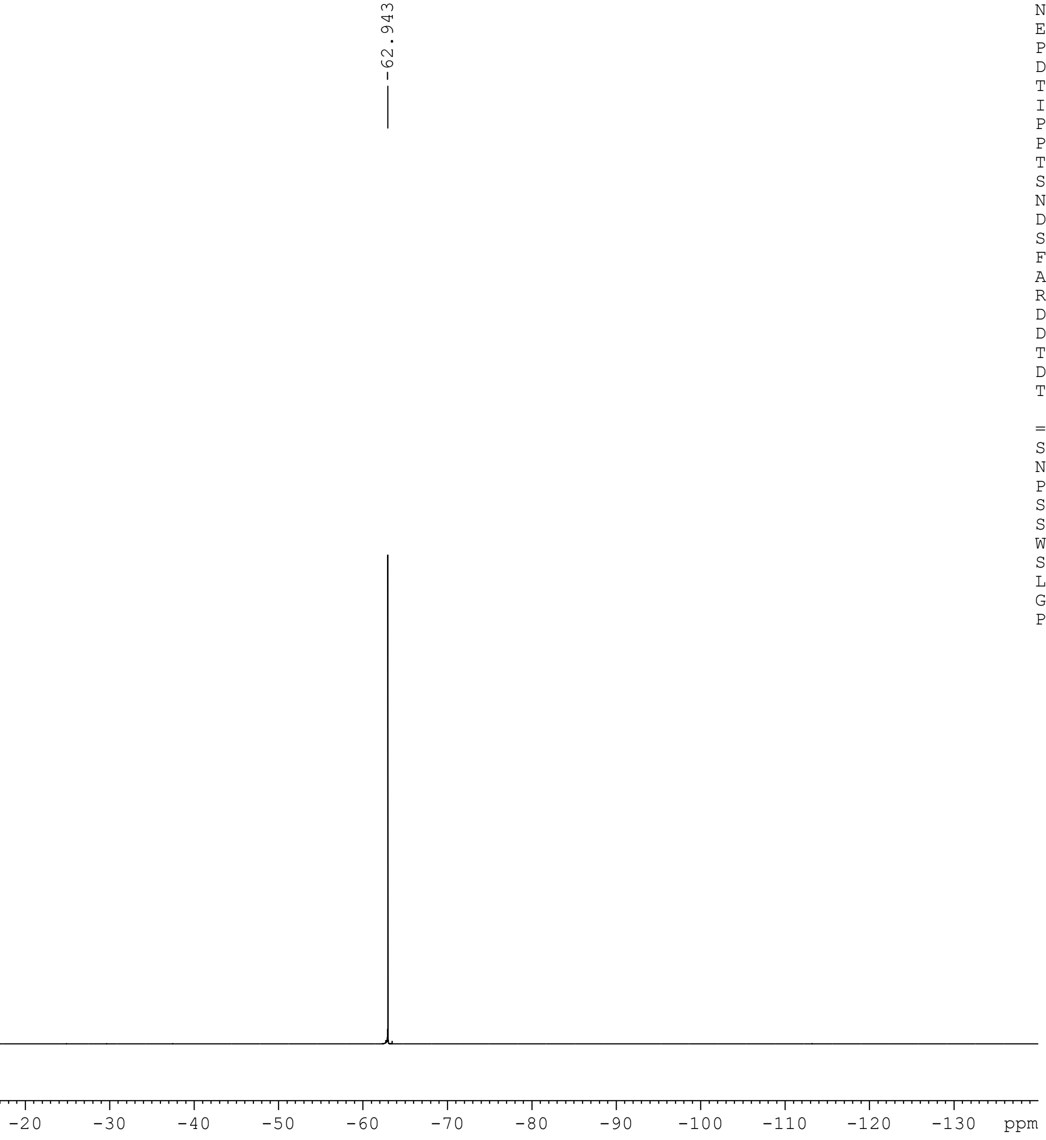


```
NAME              202408
EXPNO                396
PROCNO                 1
Date_           20240824
Time               15.37
INSTRUM            spect
PROBHD   5 mm PABBO BB/
PULPROG             zg30
TD                131072
SOLVENT            CDCl3
NS                    16
DS                     0
SWH           89285.711 Hz
FIDRES         0.681196 Hz
AQ            0.7340532 sec
RG                205.92
DW                 5.600 usec
DE                  6.50 usec
TE                 294.6 K
D1            1.00000000 sec
TD0                    1

======== CHANNEL f1 ========
SFO1         376.4757776 MHz
NUC1                 19F
P1                 15.00 usec
SI                 65536
SF           376.4983662 MHz
WDW                   EM
SSB                    0
LB                  0.30 Hz
GB                     0
PC                  1.00
```

^{1}H NMR of **1kh** ($CDCl_3$, 400 MHz)

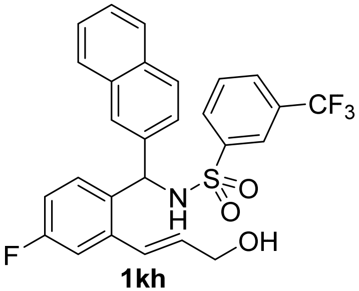

1kh

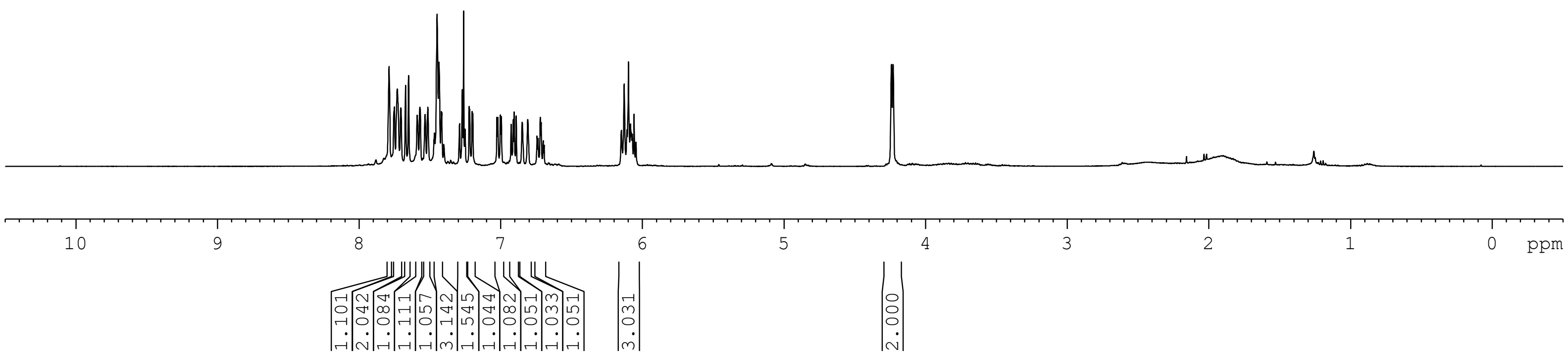

NAME	202409
EXPNO	44
PROCNO	1
Date_	20240912
Time	12.35
INSTRUM	spect
PROBHD	5 mm PABBO BB/
PULPROG	zg30
TD	32768
SOLVENT	CDCl3
NS	30
DS	0
SWH	8012.820 Hz
FIDRES	0.244532 Hz
AQ	2.0447731 sec
RG	115.33
DW	62.400 usec
DE	16.53 usec
TE	291.7 K
D1	2.00000000 sec
TD0	1

======== CHANNEL f1 ========

SFO1	400.1324008 MHz
NUC1	1H
P1	14.00 usec
SI	16384
SF	400.1300096 MHz
WDW	EM
SSB	0
LB	0.00 Hz
GB	0
PC	1.00

$^{13}C\{^1H\}$ NMR of **1kh** ($CDCl_3$, 101 MHz)

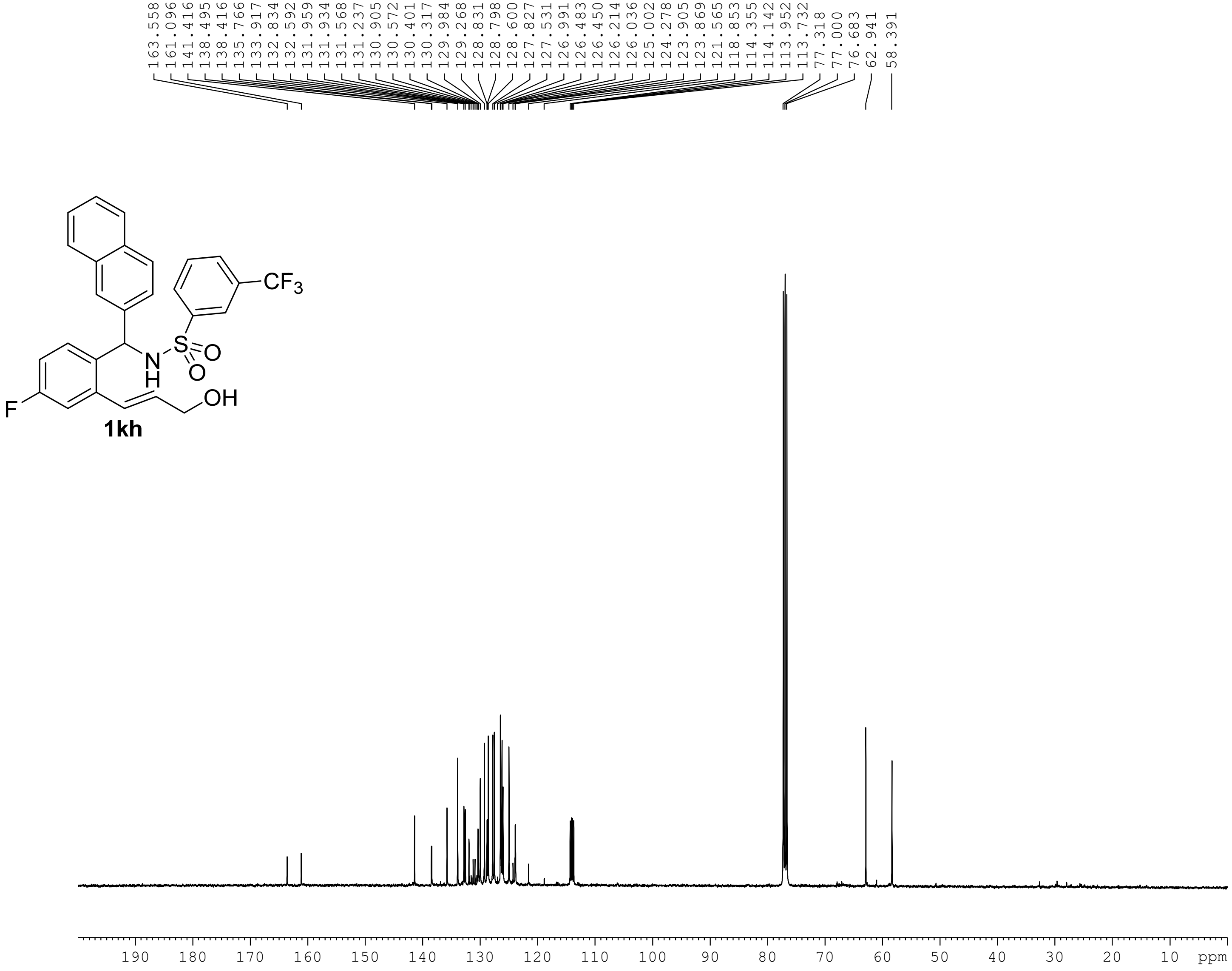


```
NAME              202409
EXPNO                 55
PROCNO                 1
Date_           20240913
Time                9.33
INSTRUM            spect
PROBHD   5 mm PABBO BB/
PULPROG           zgpg30
TD                 32768
SOLVENT            CDCl3
NS                  8000
DS                     0
SWH            24038.461 Hz
FIDRES          0.733596 Hz
AQ             0.6816244 sec
RG                205.92
DW                20.800 usec
DE                  6.50 usec
TE                 293.2 K
D1            2.00000000 sec
D11           0.03000000 sec
TD0                    1

======== CHANNEL f1 ========
SFO1         100.6233329 MHz
NUC1                 13C
P1                 10.00 usec
SI                 32768
SF           100.6127743 MHz
WDW                   EM
SSB                    0
LB                  2.00 Hz
GB                     0
PC                  1.00
```

^{19}F NMR of **1kh** ($CDCl_3$, 376 MHz)

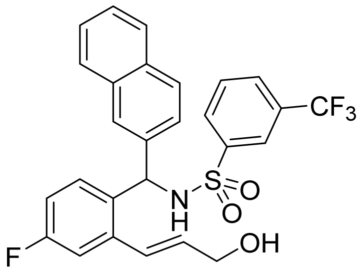

1kh

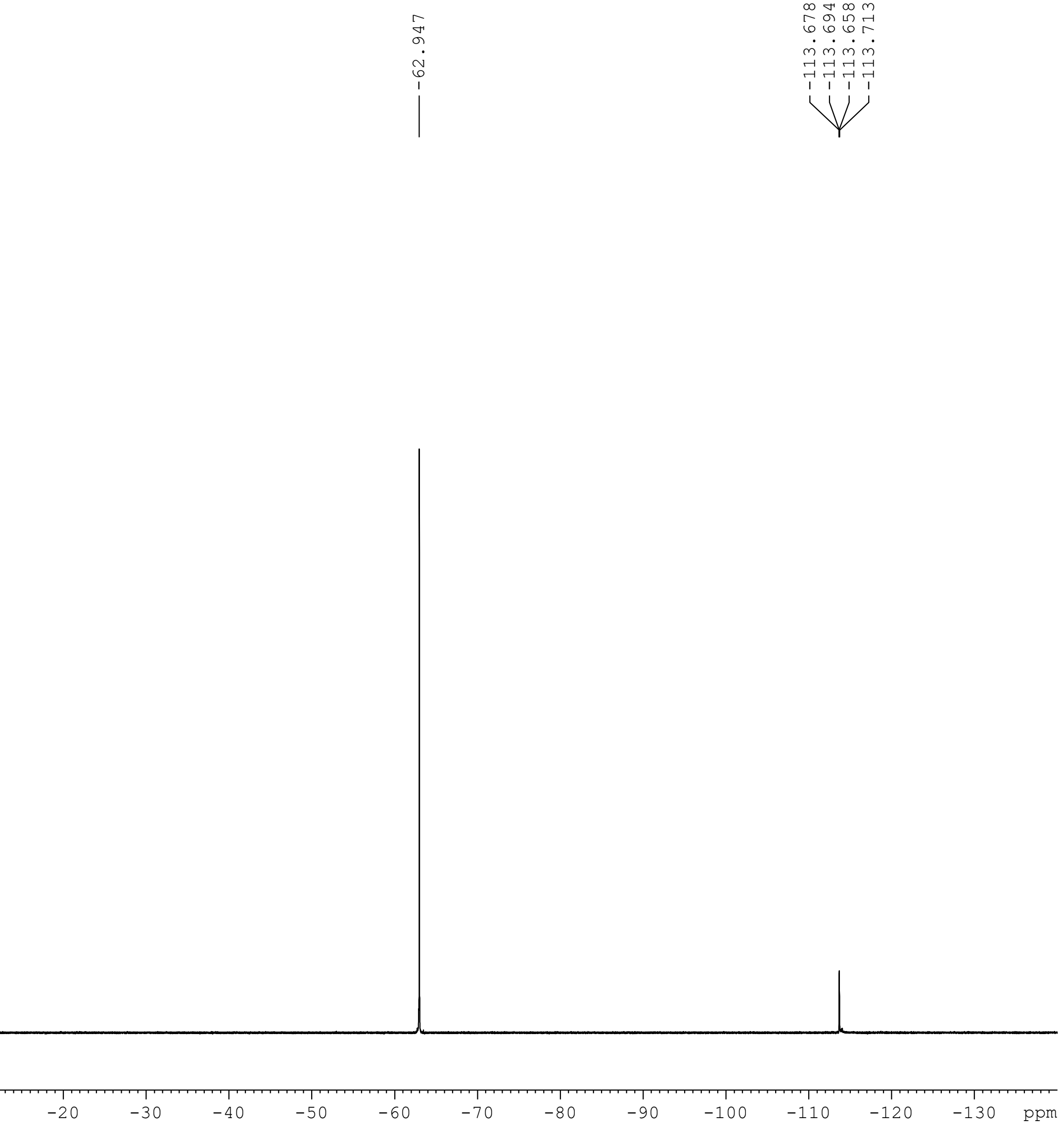


```
NAME             202409
EXPNO                33
PROCNO                1
Date_          20240911
Time              11.53
INSTRUM           spect
PROBHD   5 mm PABBO BB/
PULPROG            zg30
TD               131072
SOLVENT           CDCl3
NS                    1
DS                    0
SWH          89285.711 Hz
FIDRES        0.681196 Hz
AQ           0.7340532 sec
RG               205.92
DW                5.600 usec
DE                 6.50 usec
TE                291.7 K
D1           1.00000000 sec
TD0                   1

======== CHANNEL f1 ========
SFO1       376.4757776 MHz
NUC1                19F
P1                15.00 usec
SI                65536
SF         376.4983662 MHz
WDW                  EM
SSB                   0
LB                 0.30 Hz
GB                    0
PC                 1.00
```

^{1}H NMR of **1lh** ($CDCl_3$, 400 MHz)

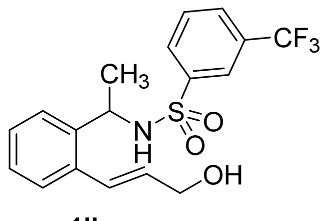

1lh

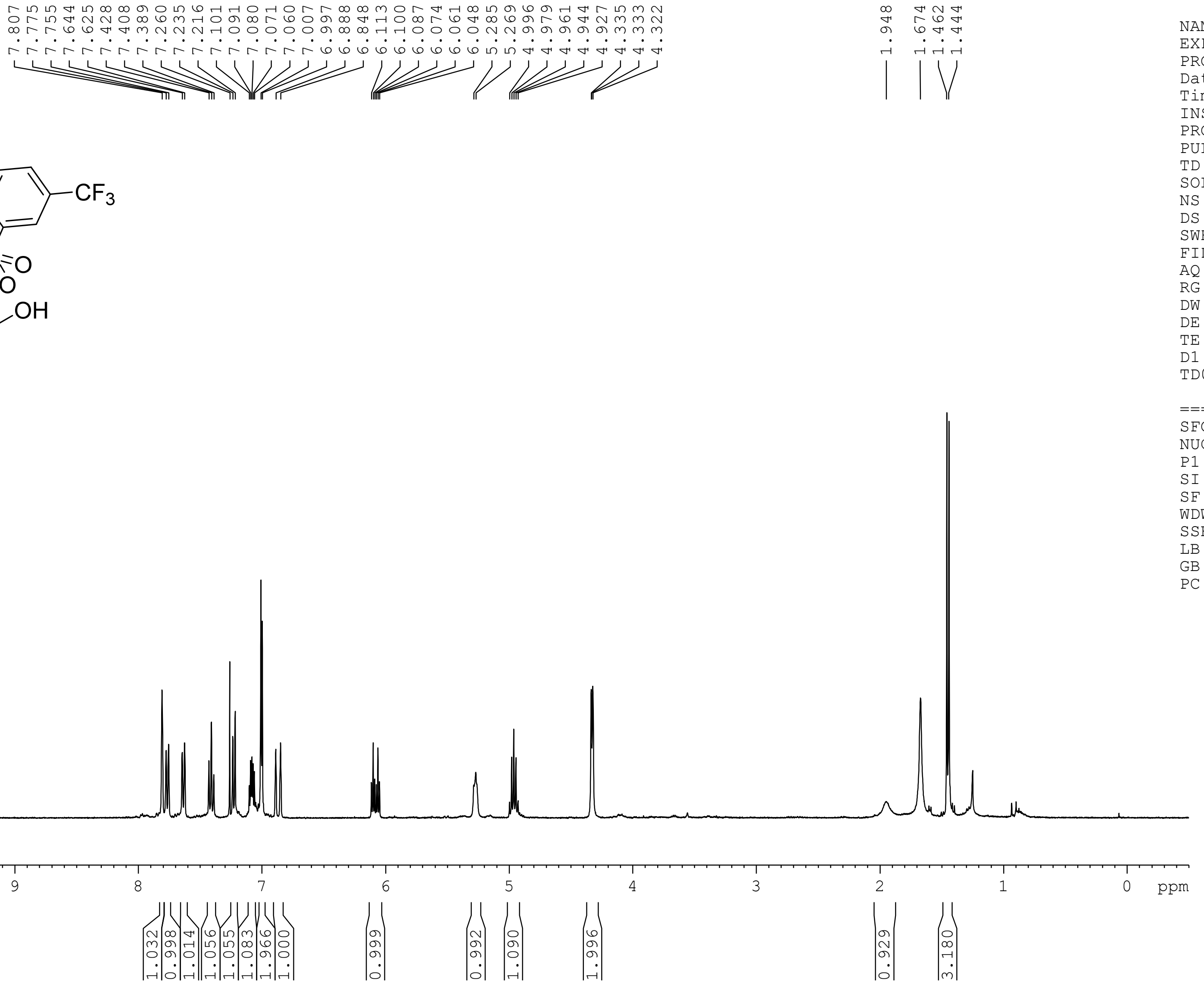

NAME	202406
EXPNO	394
PROCNO	1
Date_	20240625
Time	9.56
INSTRUM	spect
PROBHD	5 mm PABBO BB/
PULPROG	zg30
TD	32768
SOLVENT	CDCl3
NS	9
DS	0
SWH	8012.820 Hz
FIDRES	0.244532 Hz
AQ	2.0447731 sec
RG	205.92
DW	62.400 usec
DE	16.53 usec
TE	295.8 K
D1	2.00000000 sec
TD0	1

======== CHANNEL f1 ========

SFO1	400.1324008 MHz
NUC1	1H
P1	14.00 usec
SI	16384
SF	400.1300096 MHz
WDW	EM
SSB	0
LB	0.00 Hz
GB	0
PC	1.00

$^{13}C\{^{1}H\}$ NMR of **1lh** ($CDCl_3$, 101 MHz)

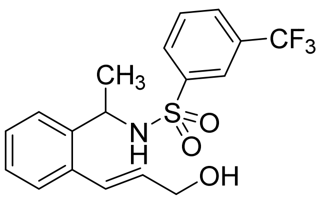

1lh

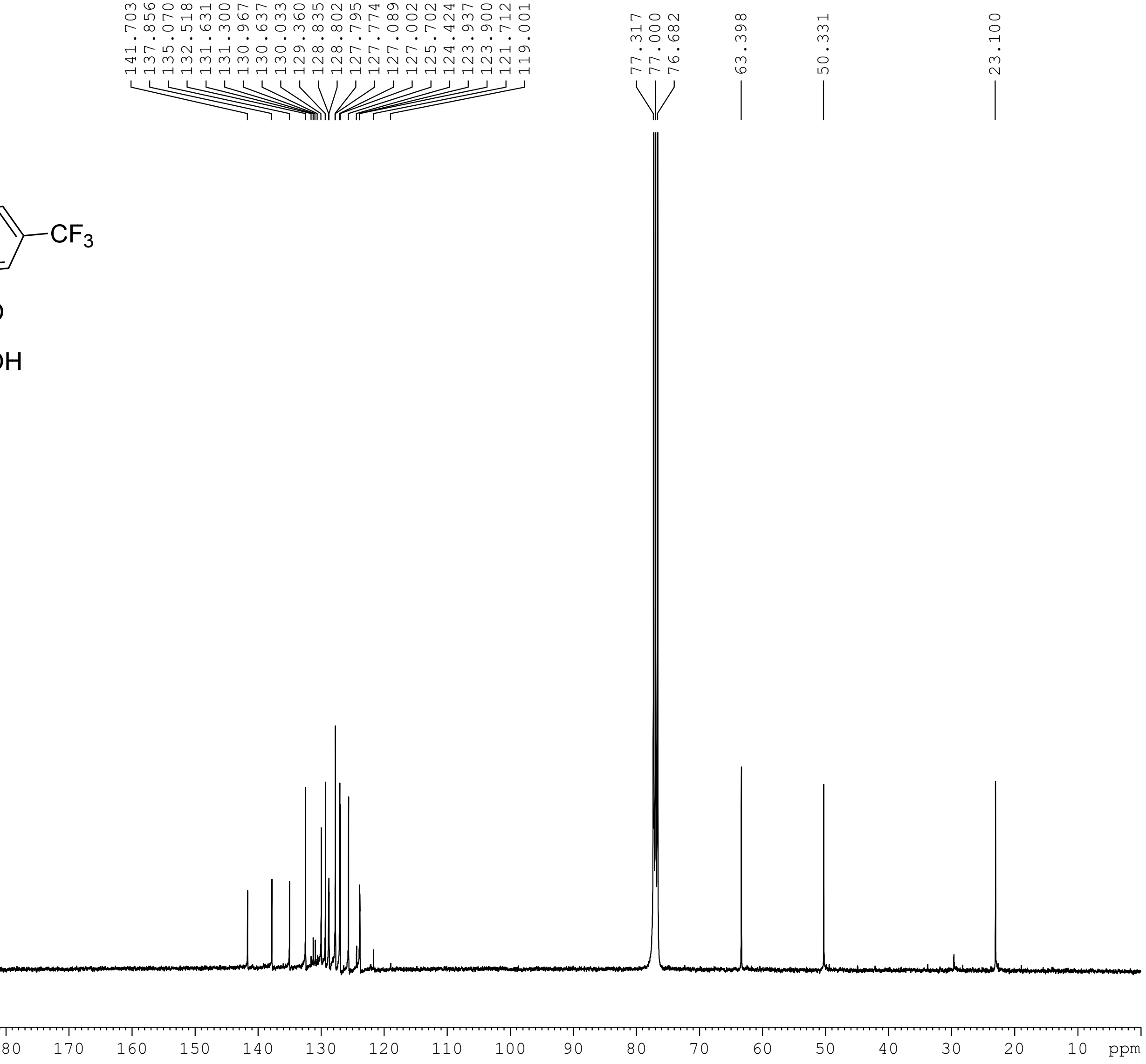

NAME	202409
EXPNO	18
PROCNO	1
Date_	20240908
Time	10.30
INSTRUM	spect
PROBHD	5 mm PABBO BB/
PULPROG	zgpg30
TD	32768
SOLVENT	CDCl3
NS	20000
DS	0
SWH	24038.461 Hz
FIDRES	0.733596 Hz
AQ	0.6816244 sec
RG	205.92
DW	20.800 usec
DE	6.50 usec
TE	292.5 K
D1	2.00000000 sec
D11	0.03000000 sec
TD0	1

======== CHANNEL f1 ========

SFO1	100.6233329 MHz
NUC1	13C
P1	10.00 usec
SI	32768
SF	100.6127725 MHz
WDW	EM
SSB	0
LB	2.00 Hz
GB	0
PC	1.00

^{19}F NMR of **1lh** ($CDCl_3$, 376 MHz)

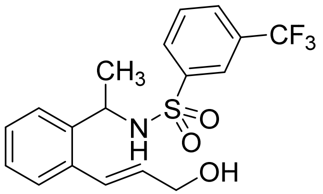

1lh

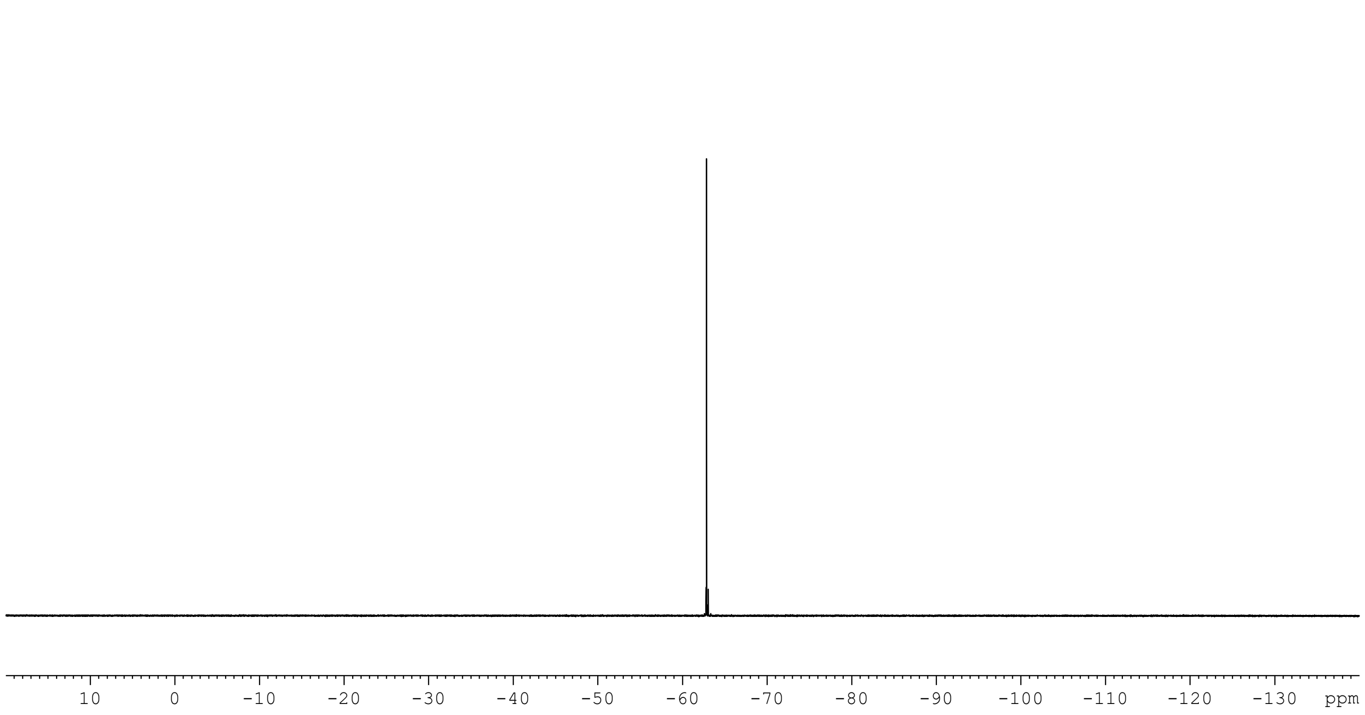


```
NAME              202406
EXPNO                333
PROCNO                 1
Date_           20240623
Time                2.15
INSTRUM            spect
PROBHD   5 mm PABBO BB/
PULPROG             zg30
TD                131072
SOLVENT            CDCl3
NS                    10
DS                     0
SWH            89285.711 Hz
FIDRES          0.681196 Hz
AQ             0.7340532 sec
RG                205.92
DW                 5.600 usec
DE                  6.50 usec
TE                 292.1 K
D1            1.00000000 sec
TD0                    1

======== CHANNEL f1 ========
SFO1         376.4757776 MHz
NUC1                 19F
P1                 15.00 usec
SI                 65536
SF           376.4983662 MHz
WDW                   EM
SSB                    0
LB                  0.30 Hz
GB                     0
PC                  1.00
```

^{1}H NMR of **2aa** ($CDCl_3$, 400 MHz)

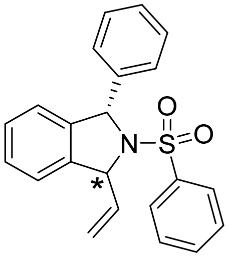

2aa, 1:1.2 dr

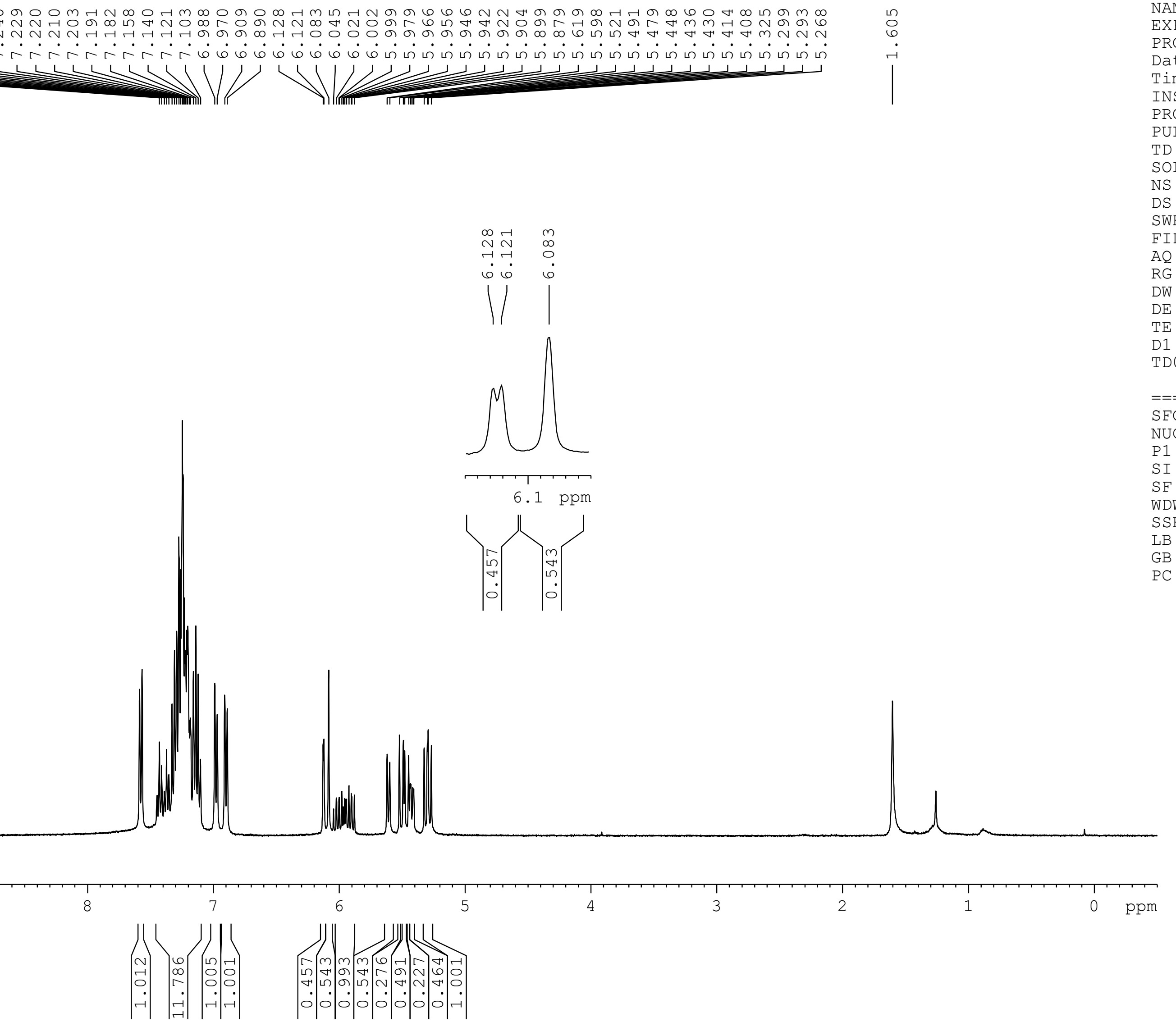


```
NAME              202407
EXPNO                408
PROCNO                 1
Date_           20240718
Time               19.04
INSTRUM            spect
PROBHD   5 mm PABBO BB/
PULPROG             zg30
TD                 32768
SOLVENT            CDCl3
NS                     4
DS                     0
SWH             8012.820 Hz
FIDRES          0.244532 Hz
AQ             2.0447731 sec
RG                137.93
DW                62.400 usec
DE                 16.53 usec
TE                 293.3 K
D1            2.00000000 sec
TD0                    1

======== CHANNEL f1 ========
SFO1         400.1324008 MHz
NUC1                  1H
P1                 14.00 usec
SI                 16384
SF           400.1300100 MHz
WDW                   EM
SSB                    0
LB                  0.00 Hz
GB                     0
PC                  1.00
```

$^{13}C\{^1H\}$ NMR of **2aa** ($CDCl_3$, 101 MHz)

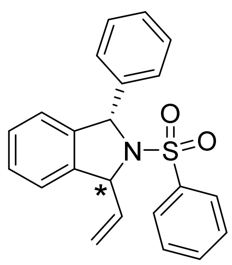

2aa, 1:1.2 dr

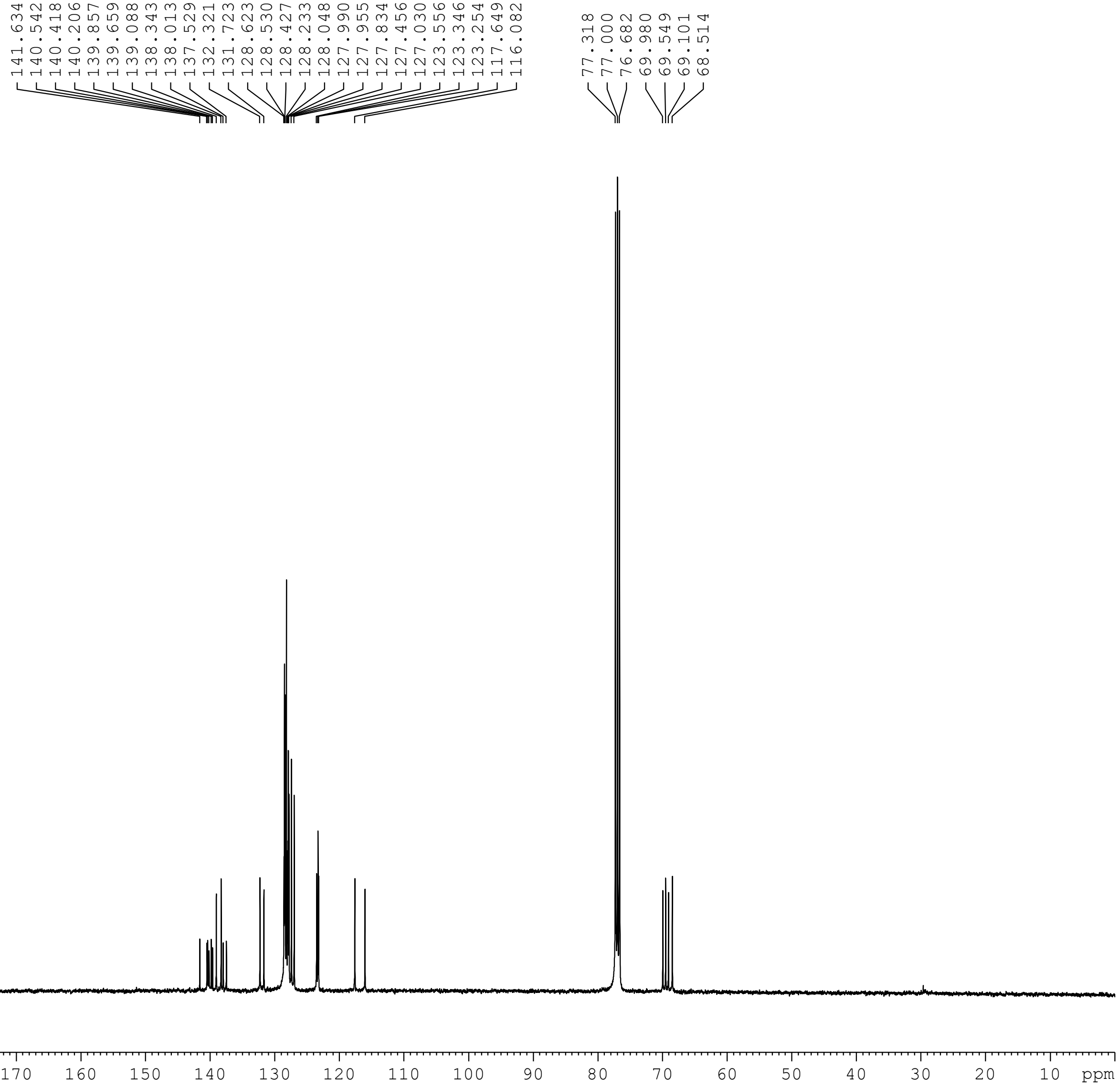


```
NAME              202407
EXPNO                409
PROCNO                 1
Date_           20240719
Time                3.14
INSTRUM            spect
PROBHD   5 mm PABBO BB/
PULPROG          zgpg30
TD                 32768
SOLVENT            CDCl3
NS                  6000
DS                     0
SWH            24038.461 Hz
FIDRES          0.733596 Hz
AQ             0.6816244 sec
RG                205.92
DW                20.800 usec
DE                  6.50 usec
TE                 293.8 K
D1            2.00000000 sec
D11           0.03000000 sec
TD0                    1

======== CHANNEL f1 ========
SFO1         100.6233329 MHz
NUC1                 13C
P1                 10.00 usec
SI                 32768
SF           100.6127742 MHz
WDW                   EM
SSB                    0
LB                  2.00 Hz
GB                     0
PC                  1.00
```

Zoomed-in 2D NMR of **2aa** ($CDCl_3$)

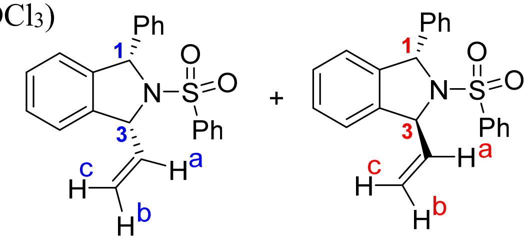

(1*S*,3*R*)-**2aa** (1*S*,3*S*)-**2aa**

COSY

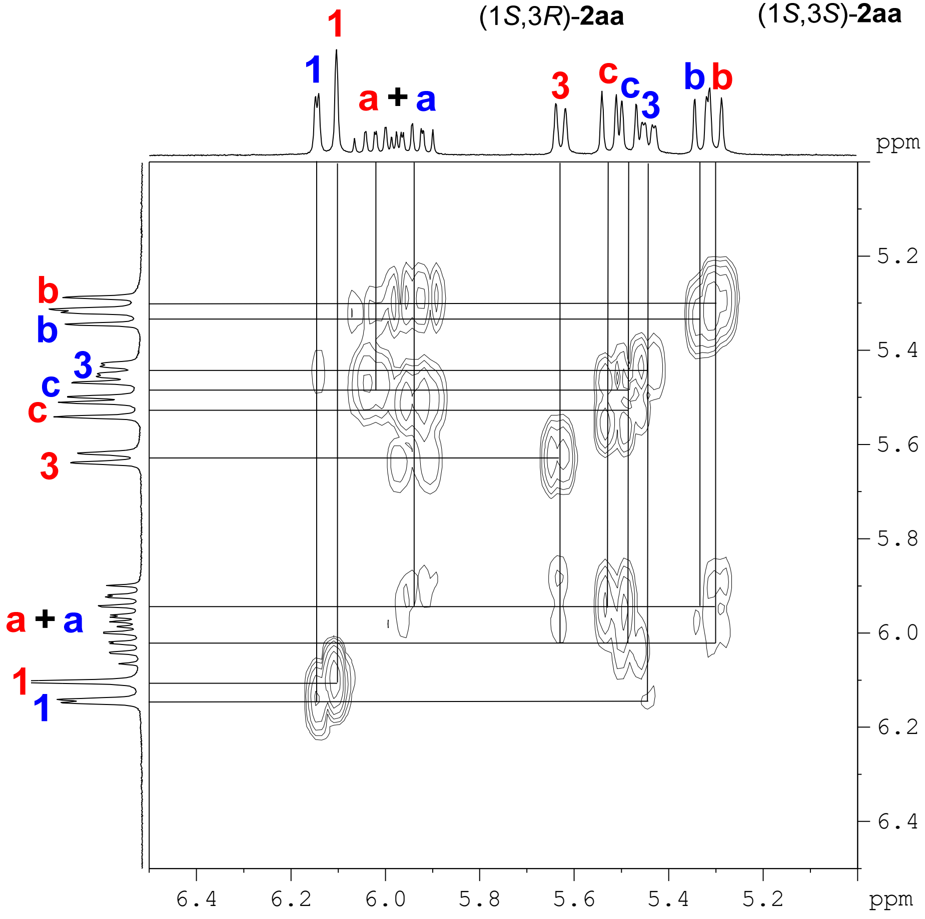

HMQC

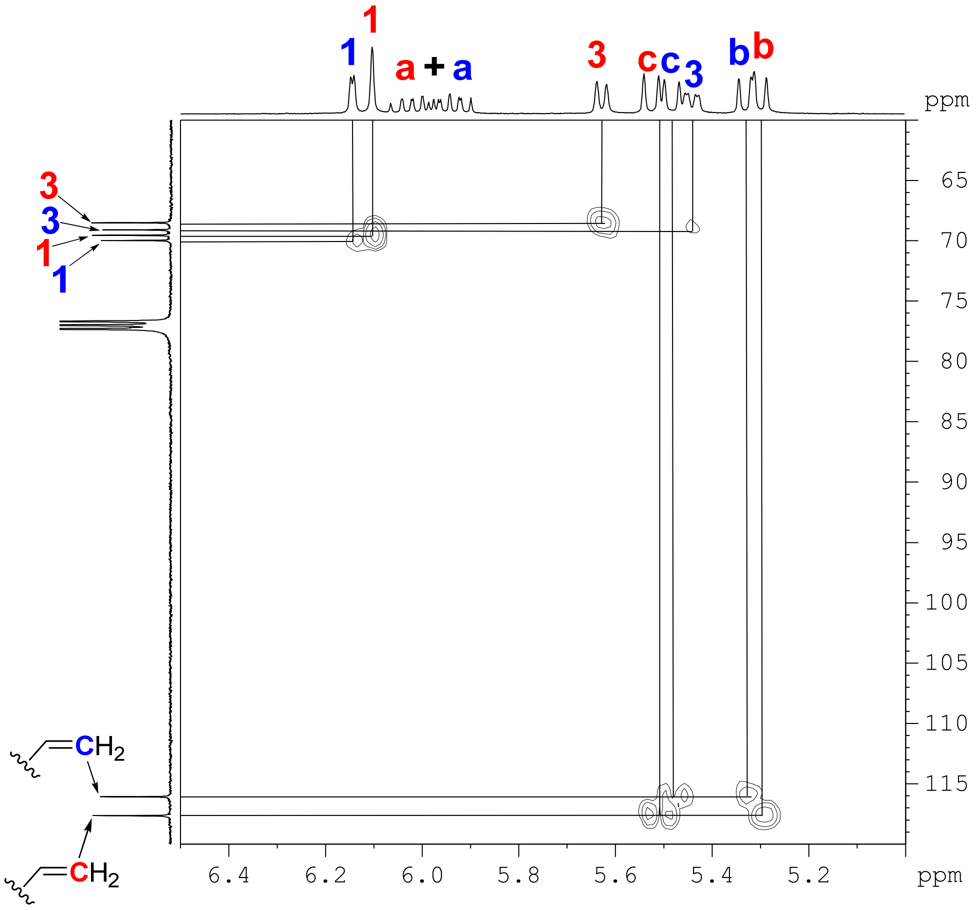

^{1}H NMR of (1*S*,3*R*)-**2aa** (CDCl$_3$, 400 MHz)

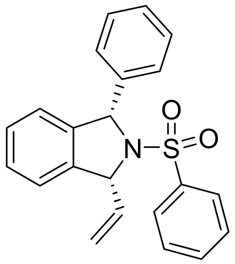

(1*S*,3*R*)-**2aa**, > 20:1 dr, synthesized from **9a**

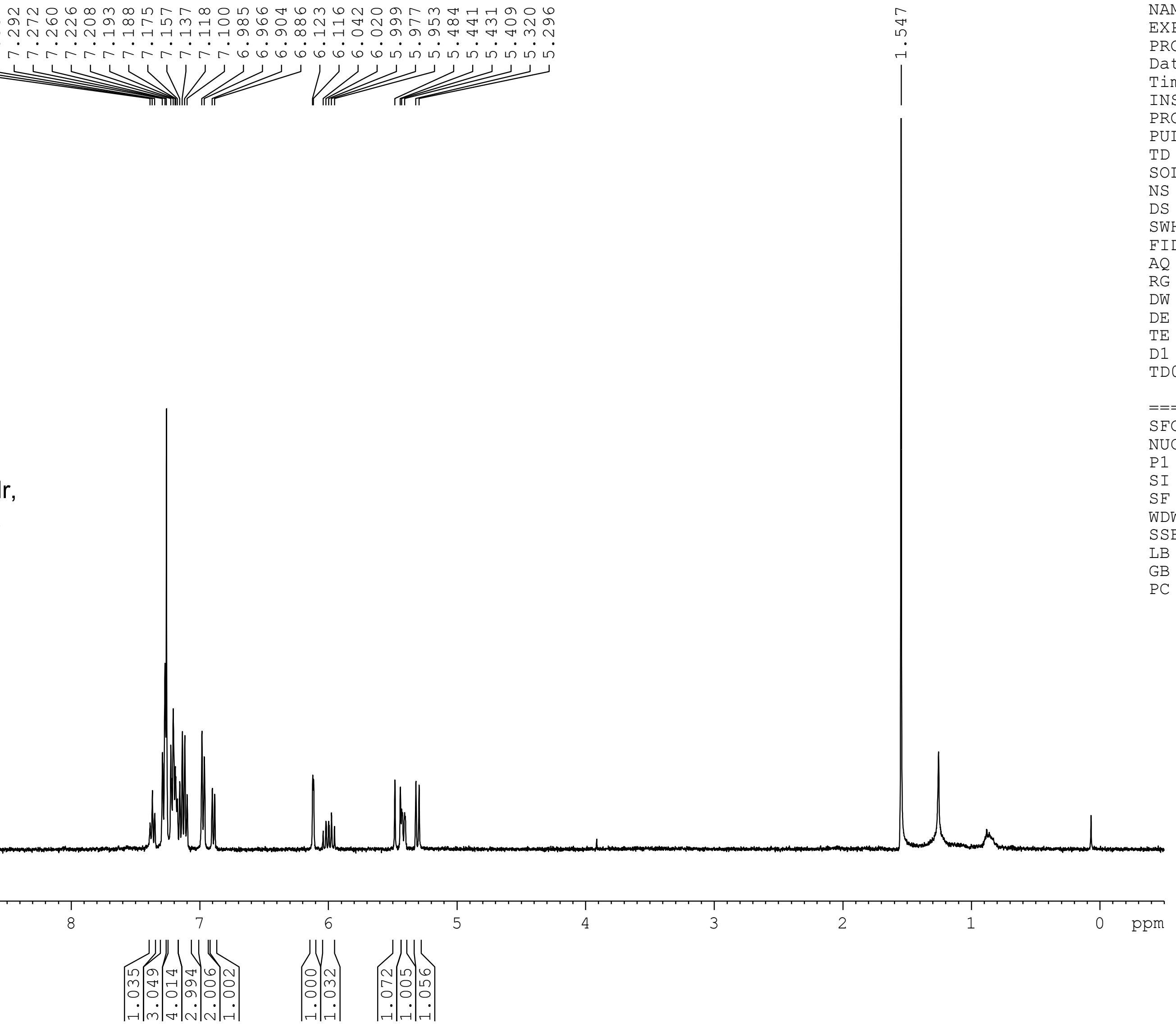

NAME	CCT113
EXPNO	325
PROCNO	1
Date_	20241222
Time	10.42
INSTRUM	spect
PROBHD	5 mm PABBO BB/
PULPROG	zg30
TD	32768
SOLVENT	CDCl3
NS	8
DS	0
SWH	8012.820 Hz
FIDRES	0.244532 Hz
AQ	2.0447731 sec
RG	205.92
DW	62.400 usec
DE	16.53 usec
TE	295.1 K
D1	2.00000000 sec
TD0	1
======== CHANNEL f1 ========	
SFO1	400.1324008 MHz
NUC1	1H
P1	14.00 usec
SI	16384
SF	400.1300092 MHz
WDW	EM
SSB	0
LB	0.00 Hz
GB	0
PC	1.00

$^{13}C\{^1H\}$ NMR of (1*S*,3*R*)-**2aa** (CDCl₃, 101 MHz)

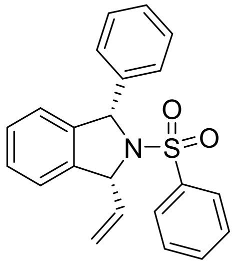

(1*S*,3*R*)-**2aa**, > 20:1 dr,
synthesized from **9a**

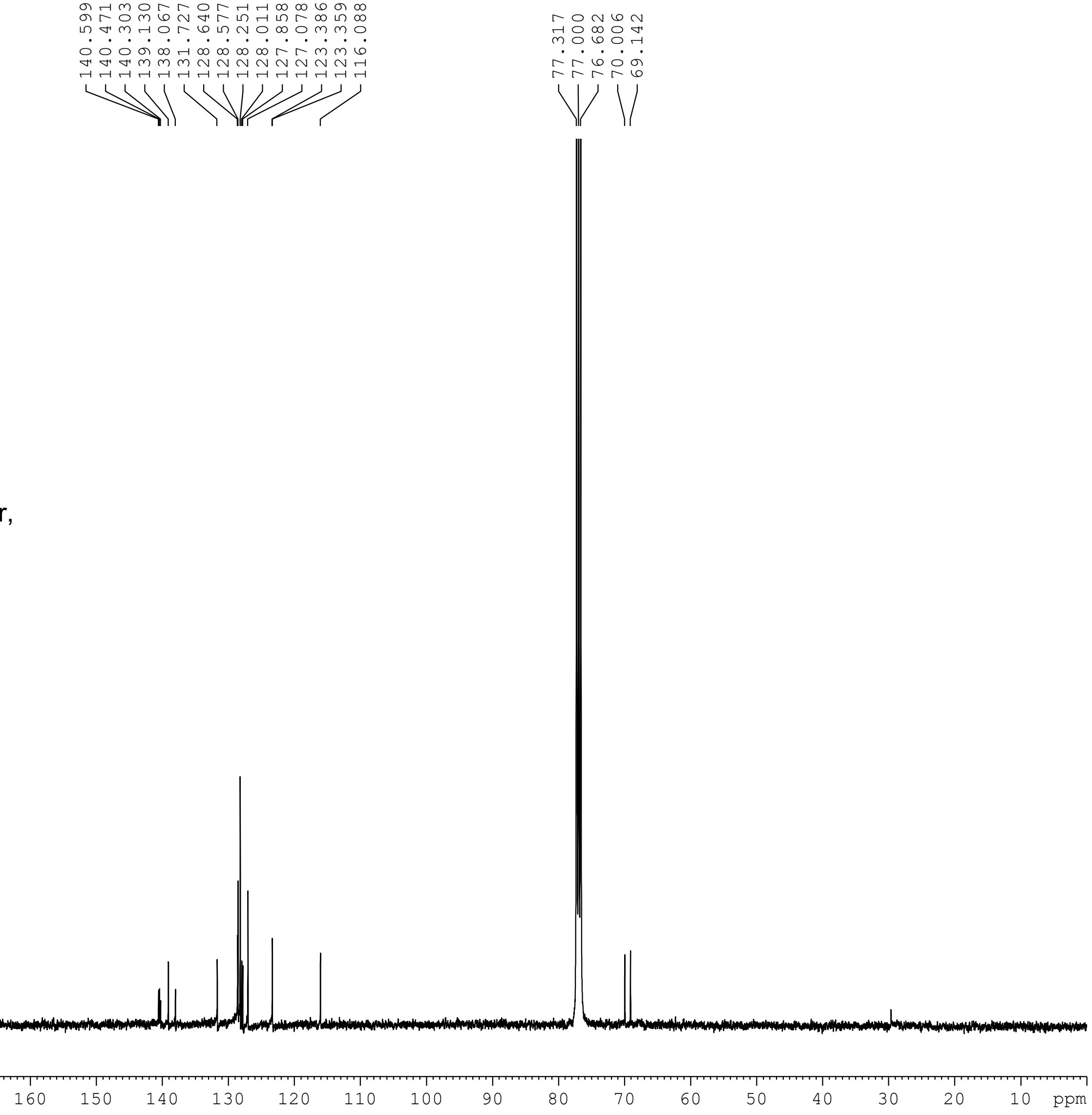

NAME	CCT113	
EXPNO	327	
PROCNO	1	
Date_	20241222	
Time	18.46	
INSTRUM	spect	
PROBHD	5 mm PABBO BB/	
PULPROG	zgpg30	
TD	32768	
SOLVENT	CDCl3	
NS	17235	
DS	0	
SWH	24038.461	Hz
FIDRES	0.733596	Hz
AQ	0.6816244	sec
RG	205.92	
DW	20.800	usec
DE	6.50	usec
TE	295.6	K
D1	2.00000000	sec
D11	0.03000000	sec
TD0	1	
======== CHANNEL f1 ========		
SFO1	100.6233329	MHz
NUC1	13C	
P1	10.00	usec
SI	32768	
SF	100.6127697	MHz
WDW	EM	
SSB	0	
LB	2.00	Hz
GB	0	
PC	1.00	

Zoomed-in 2D NMR of (1*S*,3*R*)-**2aa** ($CDCl_3$)

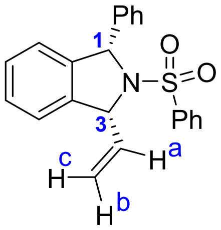

(1*S*,3*R*)-**2aa**, > 20:1 dr, synthesized from **9a**

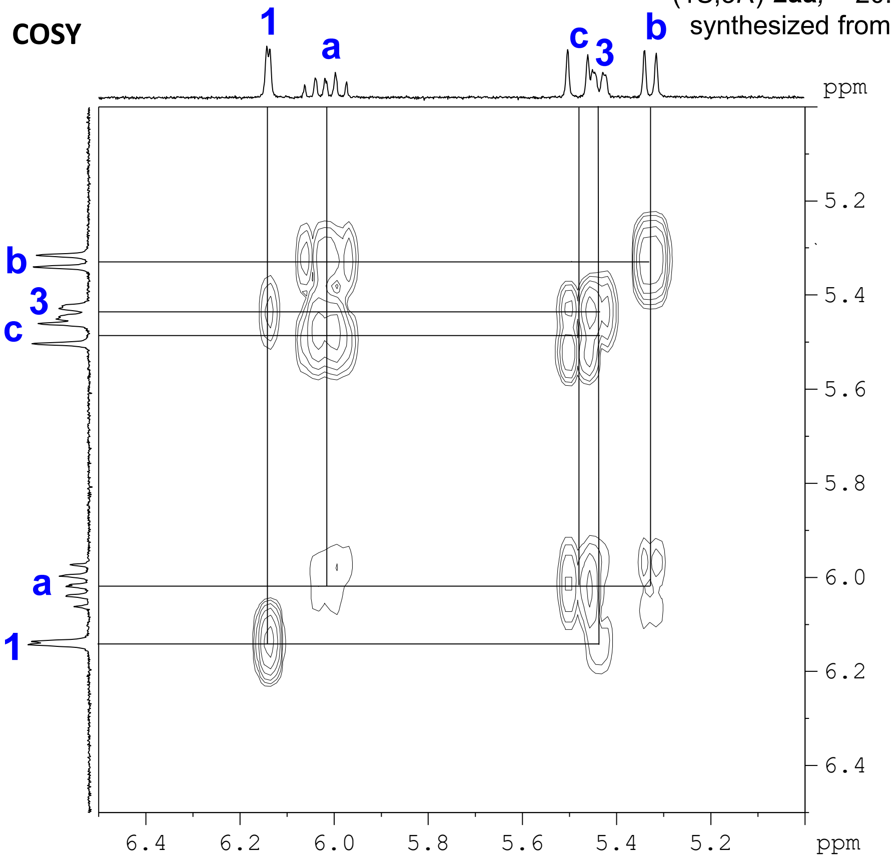

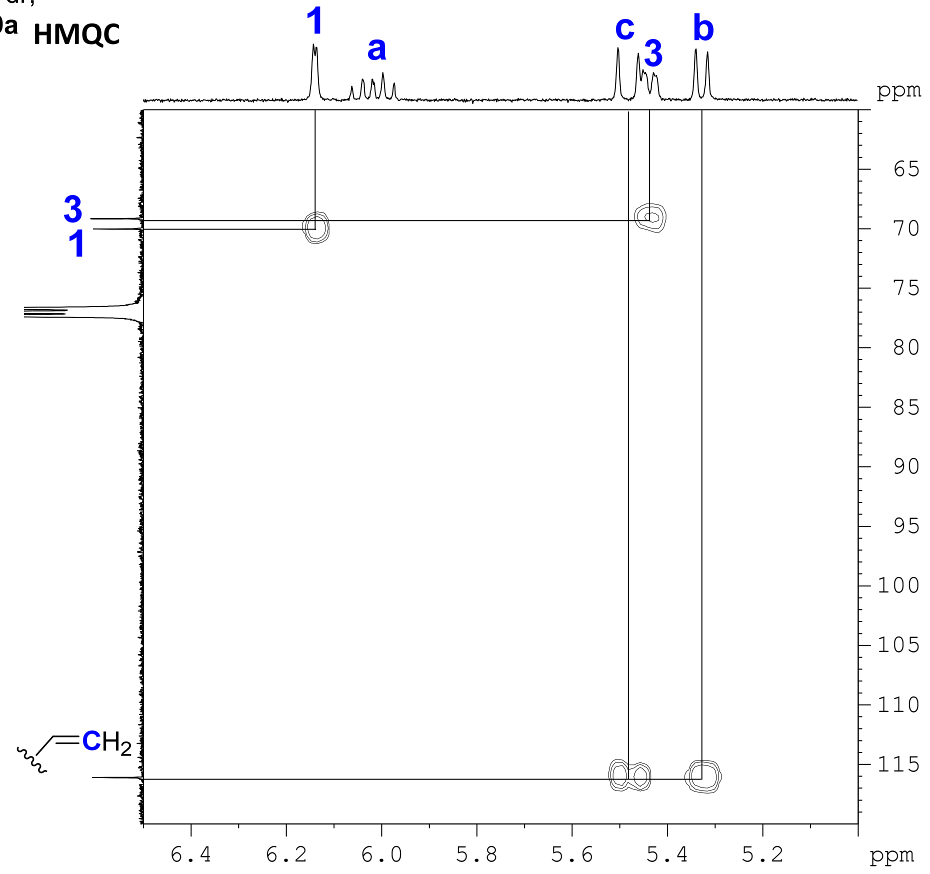

^{1}H NMR of **2ab** ($CDCl_3$, 400 MHz)

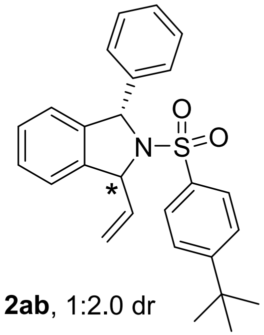

2ab, 1:2.0 dr

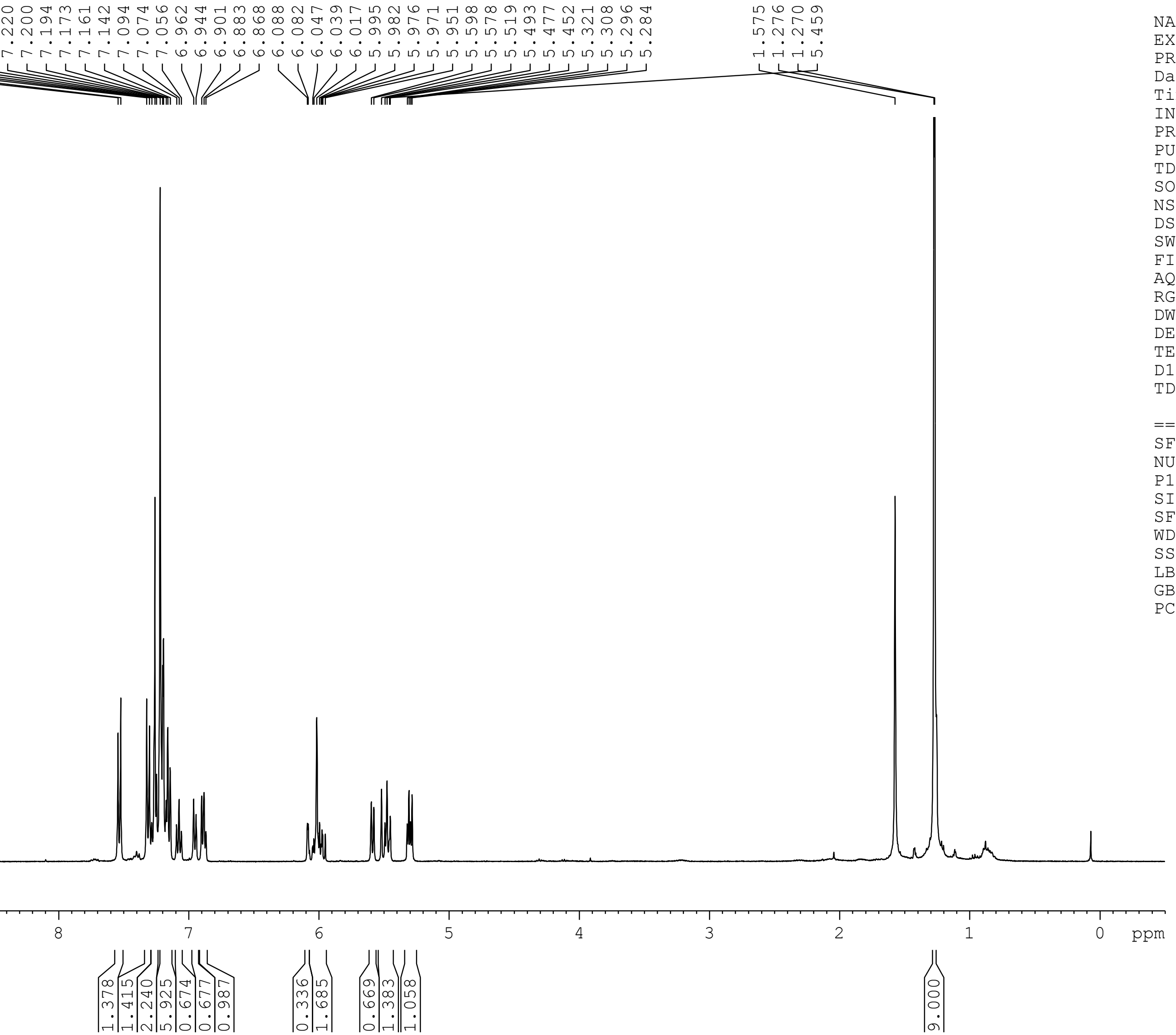

NAME	202405
EXPNO	224
PROCNO	1
Date_	20240513
Time	12.10
INSTRUM	spect
PROBHD	5 mm PABBO BB/
PULPROG	zg30
TD	32768
SOLVENT	CDCl3
NS	30
DS	0
SWH	8012.820 Hz
FIDRES	0.244532 Hz
AQ	2.0447731 sec
RG	205.92
DW	62.400 usec
DE	16.53 usec
TE	294.5 K
D1	2.00000000 sec
TD0	1

======== CHANNEL f1 ========

SFO1	400.1324008 MHz
NUC1	1H
P1	14.00 usec
SI	16384
SF	400.1300096 MHz
WDW	EM
SSB	0
LB	0.00 Hz
GB	0
PC	1.00

$^{13}C\{^{1}H\}$ NMR of **2ab** ($CDCl_3$, 101 MHz)

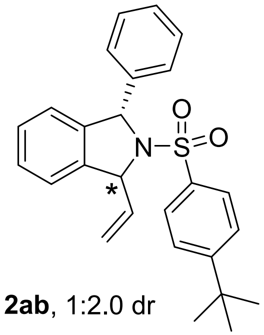

2ab, 1:2.0 dr

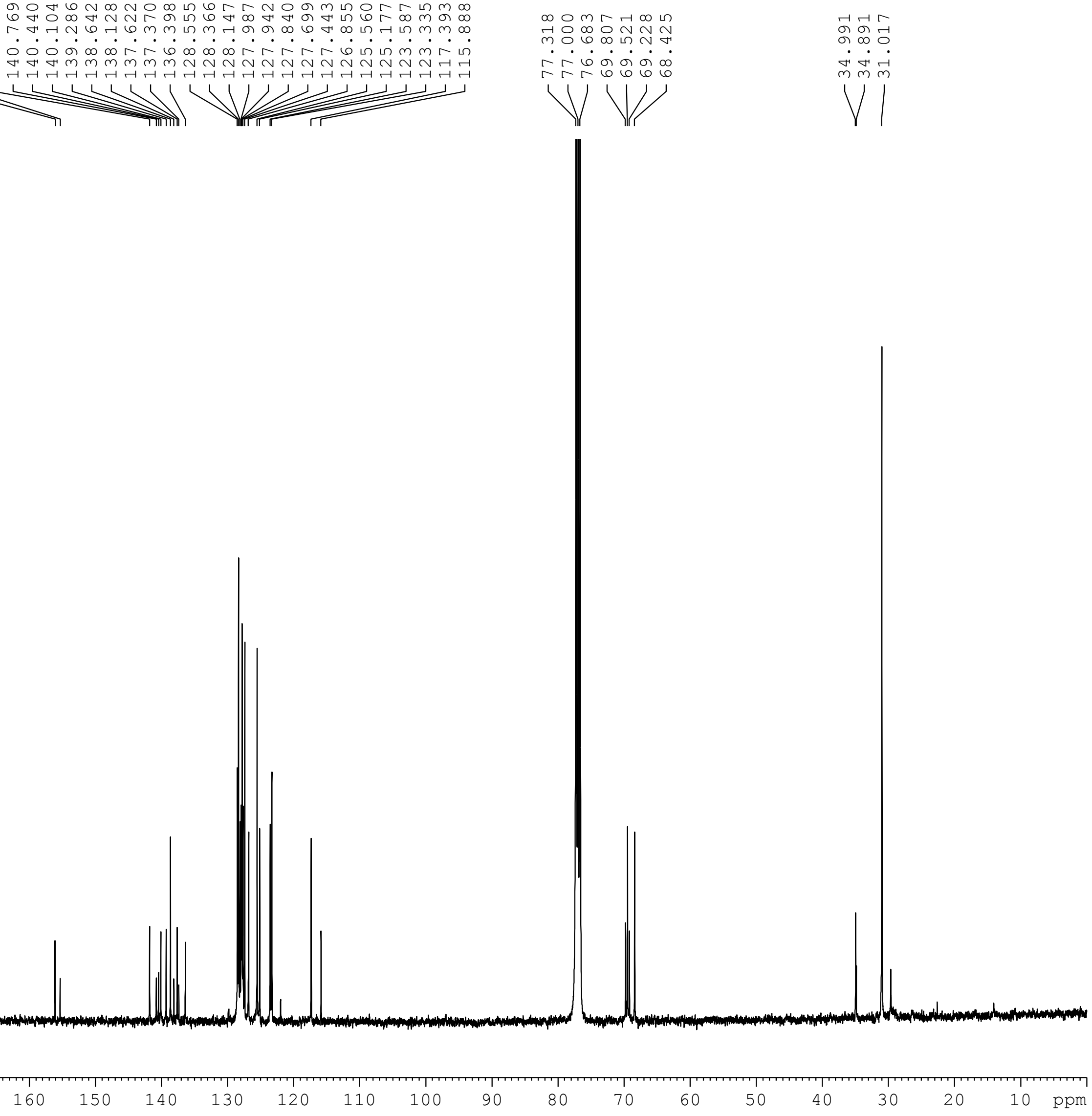


```
NAME               202405
EXPNO                 249
PROCNO                  1
Date_            20240514
Time                21.43
INSTRUM             spect
PROBHD   5 mm PABBO BB/
PULPROG           zgpg30
TD                  32768
SOLVENT             CDCl3
NS                  16000
DS                      0
SWH             24038.461 Hz
FIDRES           0.733596 Hz
AQ              0.6816244 sec
RG                 205.92
DW                 20.800 usec
DE                   6.50 usec
TE                  296.7 K
D1             2.00000000 sec
D11            0.03000000 sec
TD0                     1

======== CHANNEL f1 ========
SFO1         100.6233329 MHz
NUC1                 13C
P1                 10.00 usec
SI                 32768
SF           100.6127709 MHz
WDW                   EM
SSB                    0
LB                  2.00 Hz
GB                     0
PC                  1.00
```

^{1}H NMR of **2ac** ($CDCl_3$, 400 MHz)

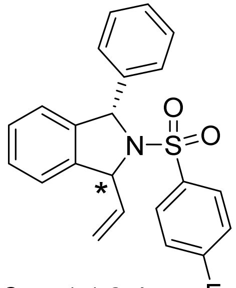

2ac, 1:1.2 dr

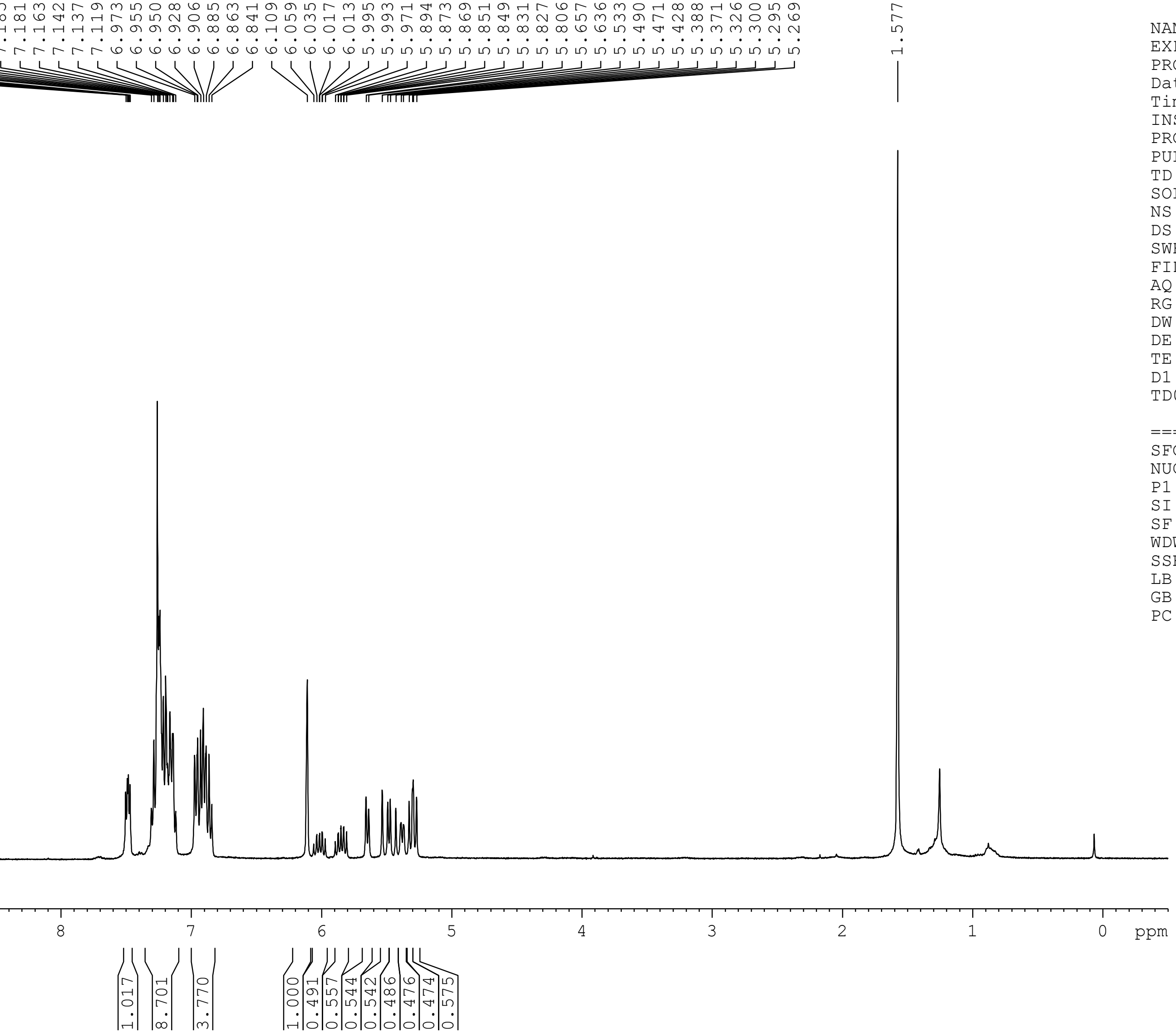

NAME	202405
EXPNO	132
PROCNO	1
Date_	20240507
Time	20.05
INSTRUM	spect
PROBHD	5 mm PABBO BB/
PULPROG	zg30
TD	32768
SOLVENT	CDCl3
NS	30
DS	0
SWH	8012.820 Hz
FIDRES	0.244532 Hz
AQ	2.0447731 sec
RG	205.92
DW	62.400 usec
DE	16.53 usec
TE	294.8 K
D1	2.00000000 sec
TD0	1
======== CHANNEL f1 ========	
SFO1	400.1324008 MHz
NUC1	1H
P1	14.00 usec
SI	16384
SF	400.1300095 MHz
WDW	EM
SSB	0
LB	0.00 Hz
GB	0
PC	1.00

$^{13}C\{^1H\}$ NMR of **2ac** ($CDCl_3$, 101 MHz)

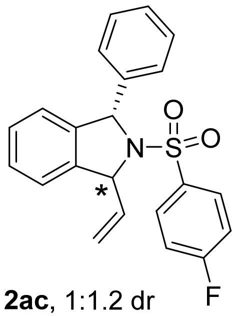

2ac, 1:1.2 dr

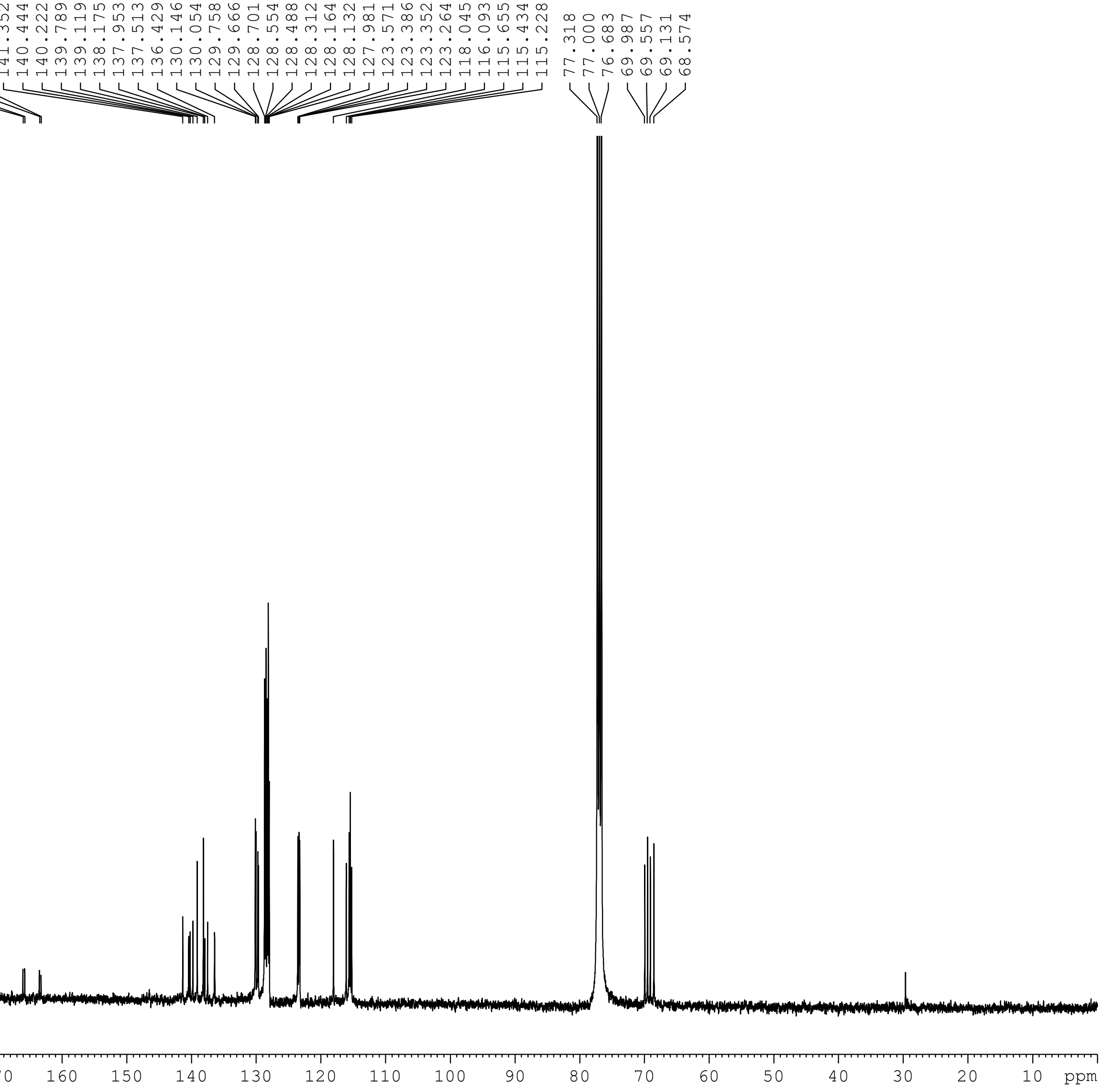

NAME	202405
EXPNO	167
PROCNO	1
Date_	20240509
Time	8.17
INSTRUM	spect
PROBHD	5 mm PABBO BB/
PULPROG	zgpg30
TD	32768
SOLVENT	CDCl3
NS	17171
DS	0
SWH	24038.461 Hz
FIDRES	0.733596 Hz
AQ	0.6816244 sec
RG	205.92
DW	20.800 usec
DE	6.50 usec
TE	296.9 K
D1	2.00000000 sec
D11	0.03000000 sec
TD0	1
======== CHANNEL f1 ========	
SFO1	100.6233329 MHz
NUC1	13C
P1	10.00 usec
SI	32768
SF	100.6127707 MHz
WDW	EM
SSB	0
LB	2.00 Hz
GB	0
PC	1.00

^{19}F NMR of **2ac** ($CDCl_3$, 376 MHz)

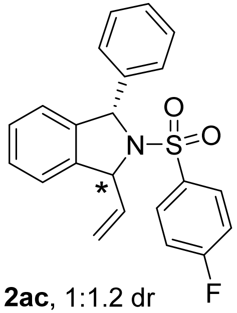

2ac, 1:1.2 dr

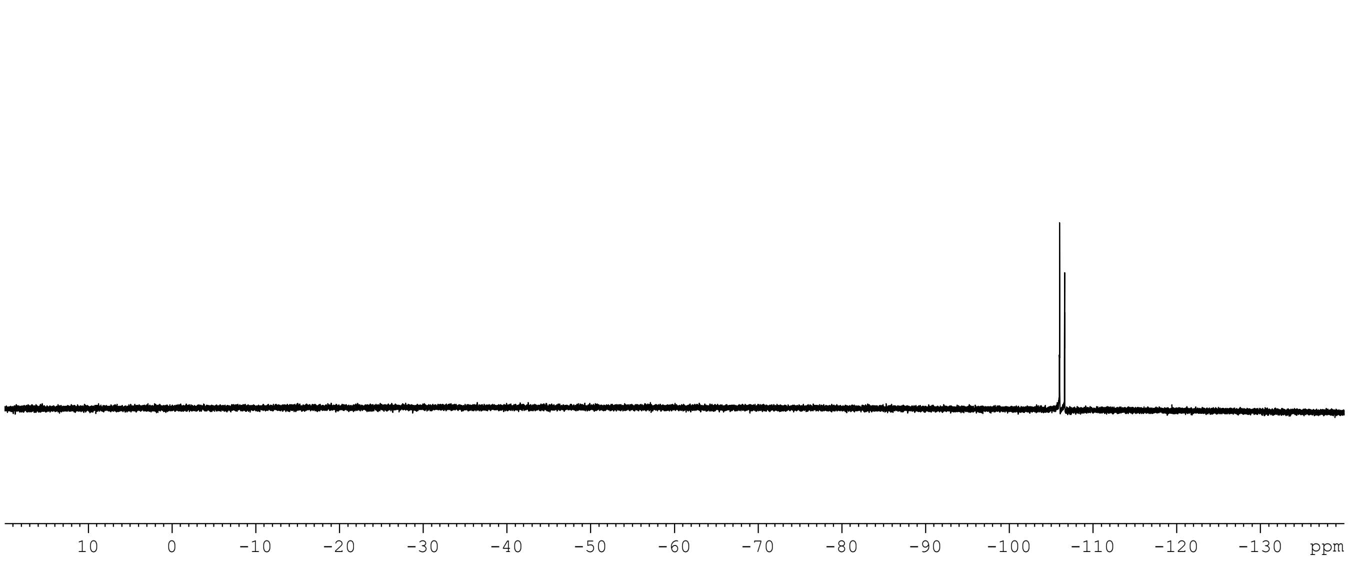

NAME	202405	
EXPNO	166	
PROCNO	1	
Date_	20240508	
Time	20.59	
INSTRUM	spect	
PROBHD	5 mm PABBO BB/	
PULPROG	zg30	
TD	131072	
SOLVENT	CDCl3	
NS	20	
DS	0	
SWH	89285.711	Hz
FIDRES	0.681196	Hz
AQ	0.7340532	sec
RG	205.92	
DW	5.600	usec
DE	6.50	usec
TE	295.8	K
D1	1.00000000	sec
TD0	1	

======== CHANNEL f1 ========

SFO1	376.4757776	MHz
NUC1	19F	
P1	15.00	usec
SI	65536	
SF	376.4983662	MHz
WDW	EM	
SSB	0	
LB	0.30	Hz
GB	0	
PC	1.00	

^{1}H NMR of **2ad** ($CDCl_3$, 400 MHz)

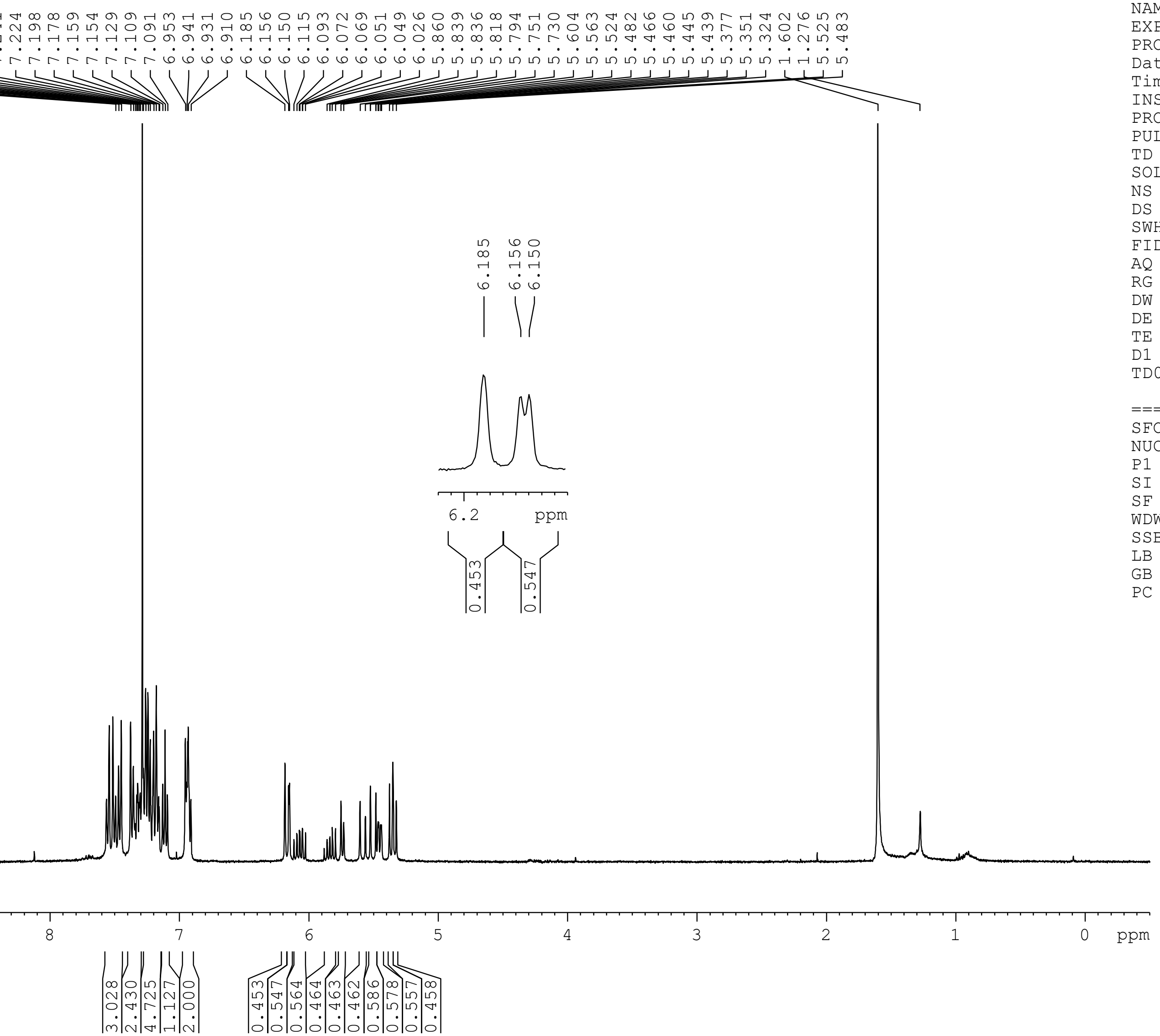

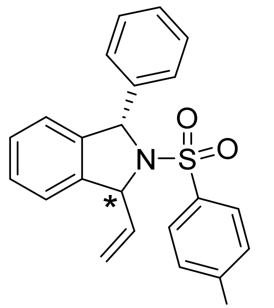

2ad, 1.2:1 dr

NAME	202407
EXPNO	444
PROCNO	1
Date_	20240719
Time	20.10
INSTRUM	spect
PROBHD	5 mm PABBO BB/
PULPROG	zg30
TD	32768
SOLVENT	CDCl3
NS	16
DS	0
SWH	8012.820 Hz
FIDRES	0.244532 Hz
AQ	2.0447731 sec
RG	205.92
DW	62.400 usec
DE	16.53 usec
TE	291.5 K
D1	2.00000000 sec
TD0	1

======== CHANNEL f1 ========

SFO1	400.1324008 MHz
NUC1	1H
P1	14.00 usec
SI	16384
SF	400.1300000 MHz
WDW	EM
SSB	0
LB	0.00 Hz
GB	0
PC	1.00

$^{13}C\{^{1}H\}$ NMR of **2ad** ($CDCl_3$, 101 MHz)

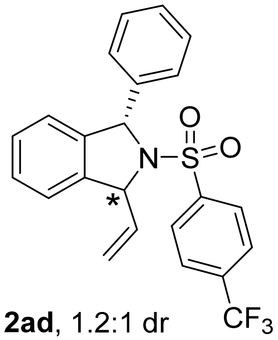

2ad, 1.2:1 dr

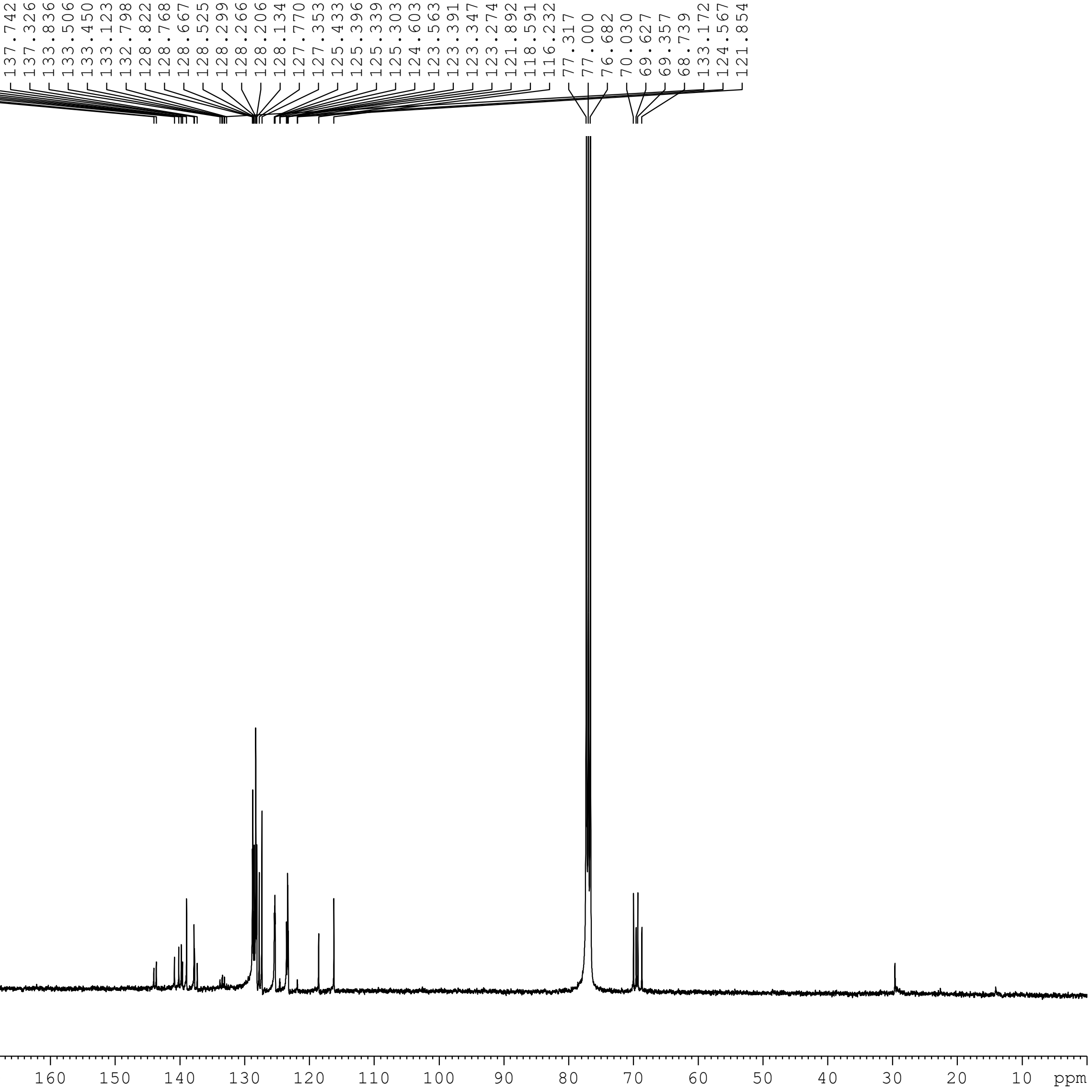

NAME	202407
EXPNO	577
PROCNO	1
Date_	20240728
Time	15.10
INSTRUM	spect
PROBHD	5 mm PABBO BB/
PULPROG	zgpg30
TD	32768
SOLVENT	CDCl3
NS	20000
DS	0
SWH	24038.461 Hz
FIDRES	0.733596 Hz
AQ	0.6816244 sec
RG	205.92
DW	20.800 usec
DE	6.50 usec
TE	294.3 K
D1	2.00000000 sec
D11	0.03000000 sec
TD0	1

======== CHANNEL f1 ========

SFO1	100.6233329 MHz
NUC1	13C
P1	10.00 usec
SI	32768
SF	100.6127721 MHz
WDW	EM
SSB	0
LB	2.00 Hz
GB	0
PC	1.00

^{19}F NMR of **2ad** ($CDCl_3$, 376 MHz)

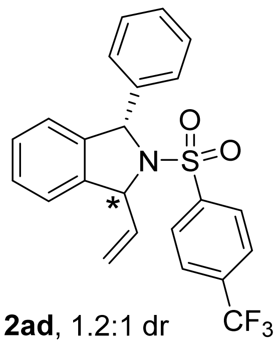

2ad, 1.2:1 dr

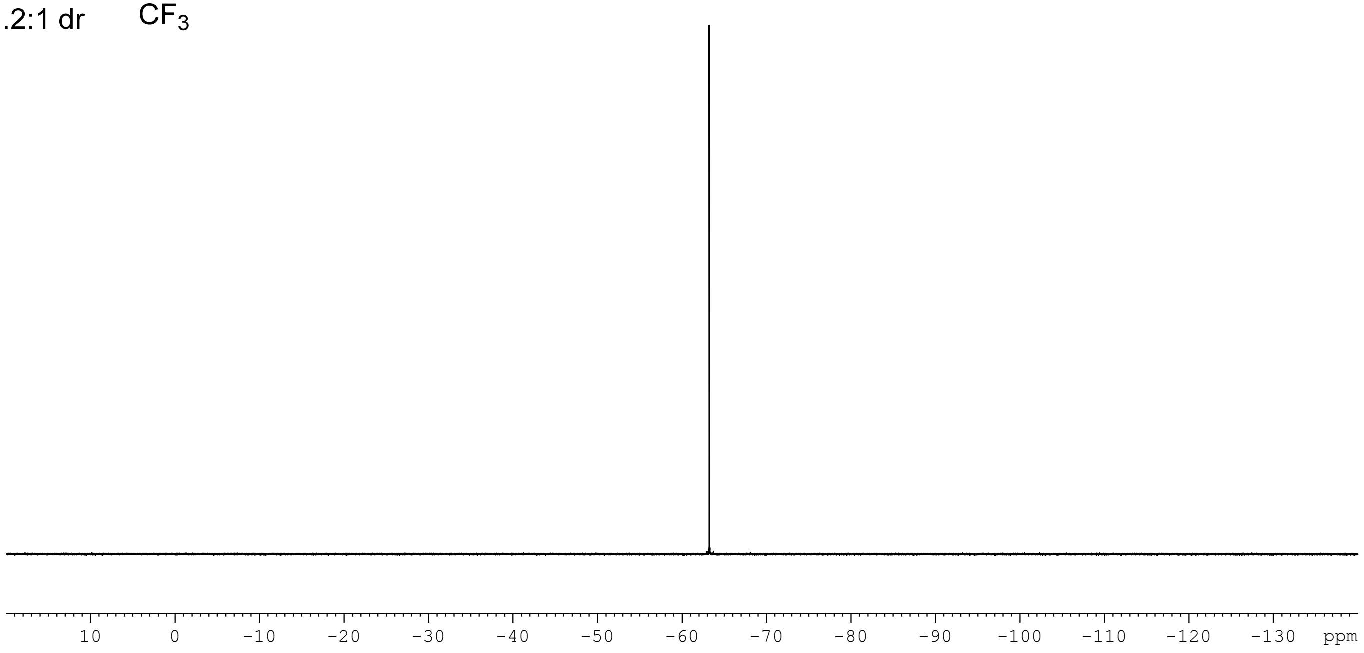

NAME	202405
EXPNO	531
PROCNO	1
Date_	20240530
Time	10.18
INSTRUM	spect
PROBHD	5 mm PABBO BB/
PULPROG	zg30
TD	131072
SOLVENT	CDCl3
NS	19
DS	0
SWH	89285.711 Hz
FIDRES	0.681196 Hz
AQ	0.7340532 sec
RG	205.92
DW	5.600 usec
DE	6.50 usec
TE	296.5 K
D1	1.00000000 sec
TD0	1

======== CHANNEL f1 ========

SFO1	376.4757776 MHz
NUC1	19F
P1	15.00 usec
SI	65536
SF	376.4983662 MHz
WDW	EM
SSB	0
LB	0.30 Hz
GB	0
PC	1.00

^{1}H NMR of **2ae** ($CDCl_3$, 400 MHz)

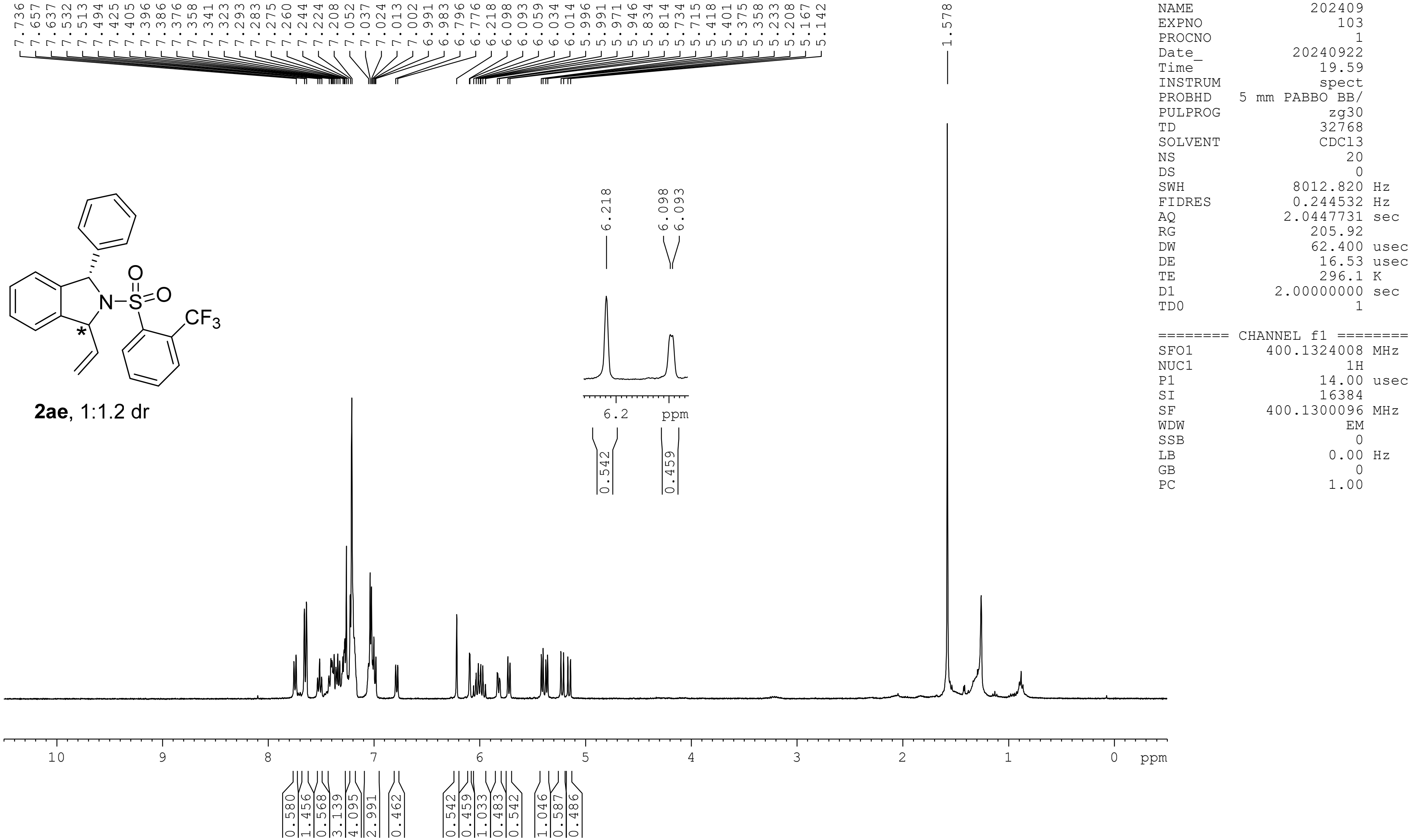

$^{13}C\{^1H\}$ NMR of **2ae** ($CDCl_3$, 101 MHz)

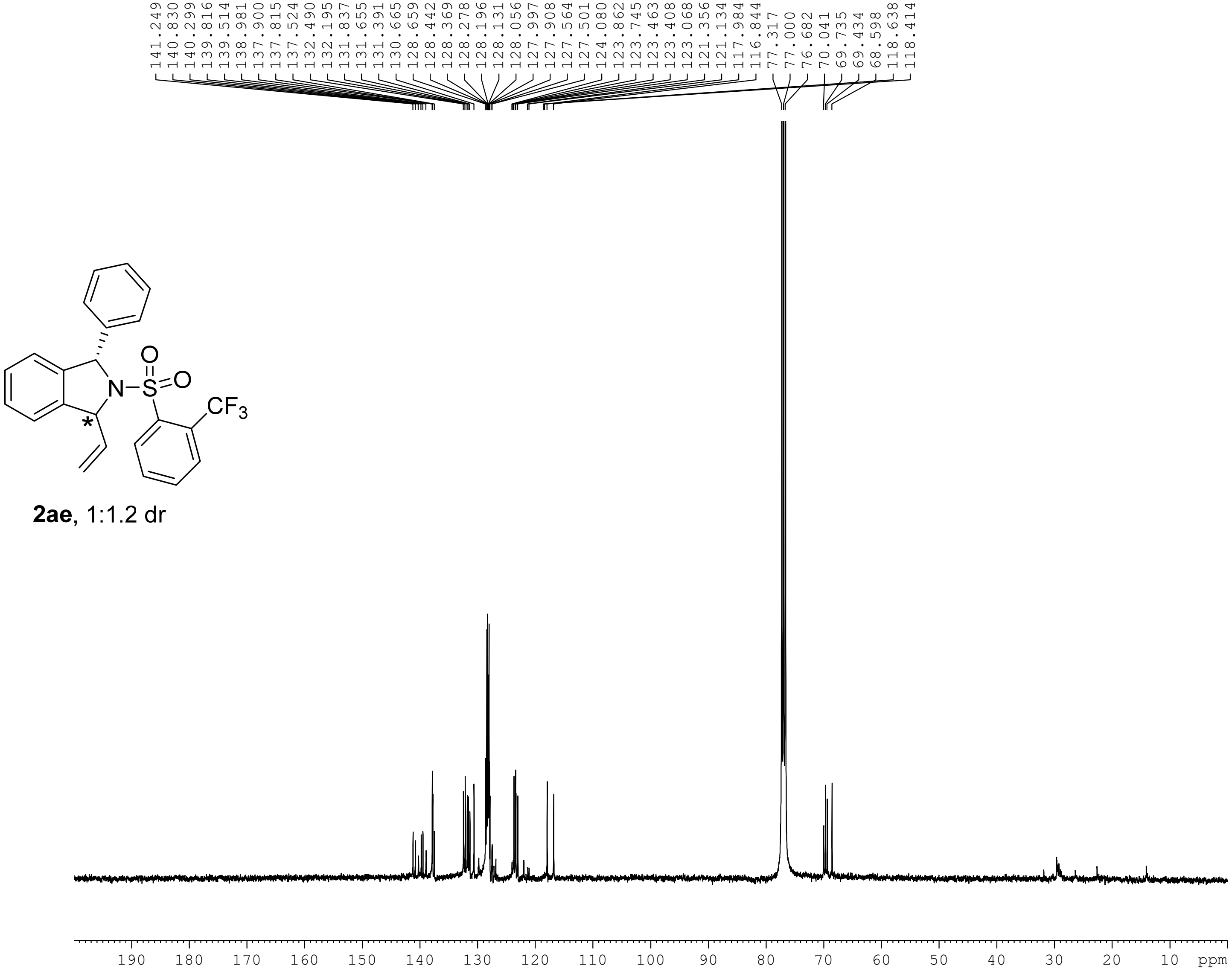

2ae, 1:1.2 dr

NAME	202409
EXPNO	119
PROCNO	1
Date_	20240925
Time	7.24
INSTRUM	spect
PROBHD	5 mm PABBO BB/
PULPROG	zgpg30
TD	32768
SOLVENT	CDCl3
NS	16477
DS	0
SWH	24038.461 Hz
FIDRES	0.733596 Hz
AQ	0.6816244 sec
RG	205.92
DW	20.800 usec
DE	6.50 usec
TE	293.5 K
D1	2.00000000 sec
D11	0.03000000 sec
TD0	1
======== CHANNEL f1 ========	
SFO1	100.6233329 MHz
NUC1	13C
P1	10.00 usec
SI	32768
SF	100.6127725 MHz
WDW	EM
SSB	0
LB	2.00 Hz
GB	0
PC	1.00

^{19}F NMR of **2ae** ($CDCl_3$, 376 MHz)

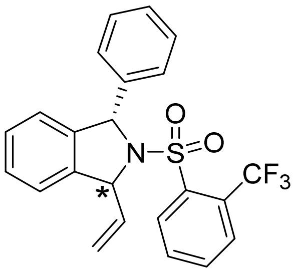

2ae, 1:1.2 dr

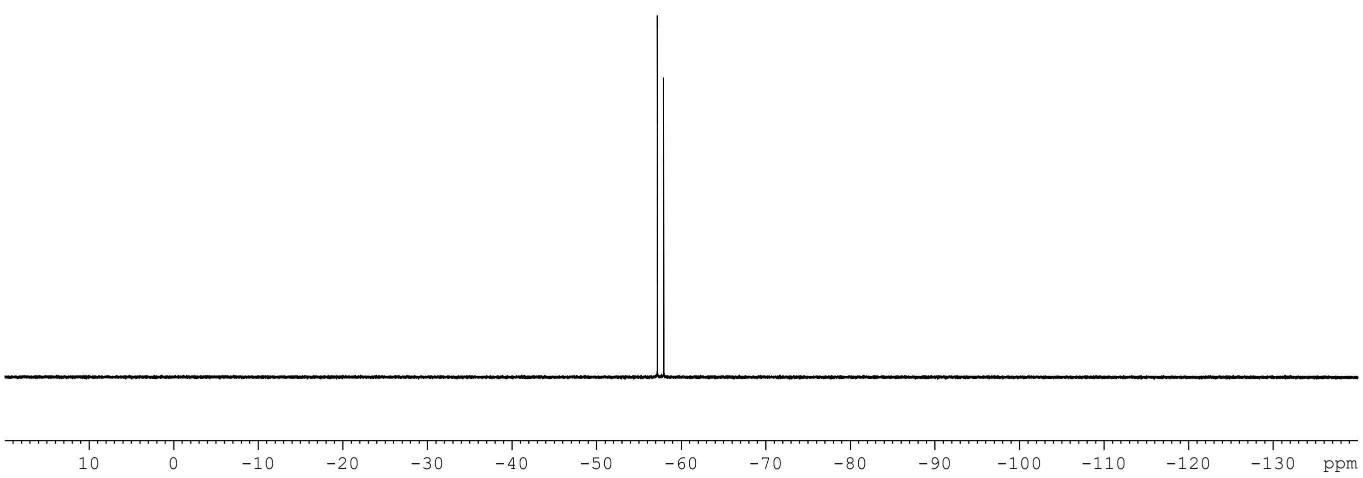

NAME	202409	
EXPNO	118	
PROCNO	1	
Date_	20240924	
Time	21.15	
INSTRUM	spect	
PROBHD	5 mm PABBO BB/	
PULPROG	zg30	
TD	131072	
SOLVENT	CDCl3	
NS	15	
DS	0	
SWH	89285.711	Hz
FIDRES	0.681196	Hz
AQ	0.7340532	sec
RG	205.92	
DW	5.600	usec
DE	6.50	usec
TE	292.4	K
D1	1.00000000	sec
TD0	1	

======== CHANNEL f1 ========

SFO1	376.4757776	MHz
NUC1	19F	
P1	15.00	usec
SI	65536	
SF	376.4983662	MHz
WDW	EM	
SSB	0	
LB	0.30	Hz
GB	0	
PC	1.00	

^{1}H NMR of **2af** ($CDCl_3$, 400 MHz)

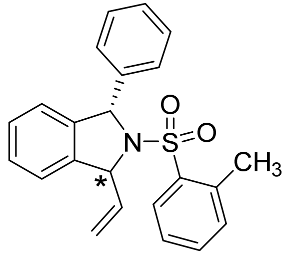

2af, 1:2.0 dr

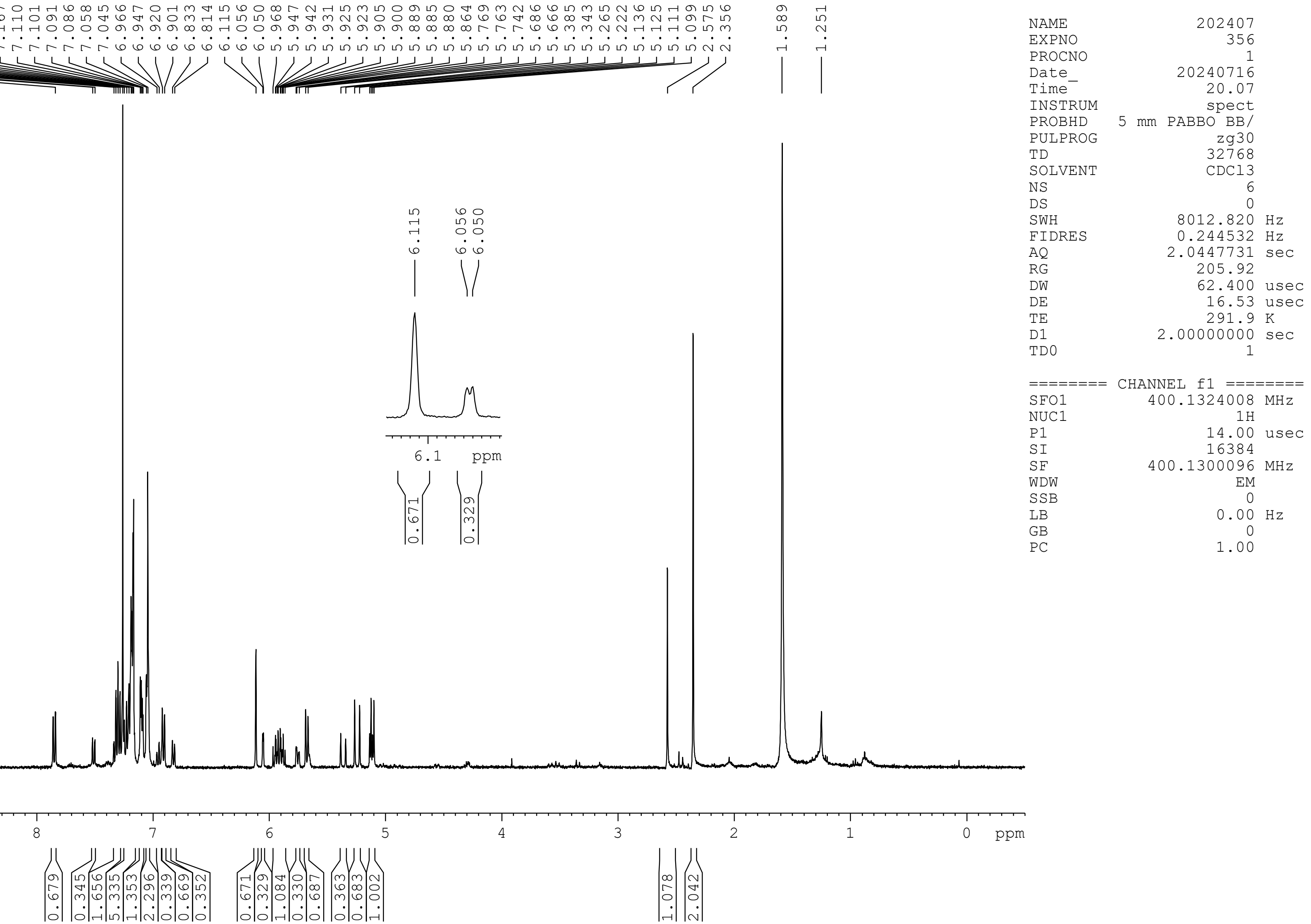

$^{13}C\{^1H\}$ NMR of **2af** ($CDCl_3$, 101 MHz)

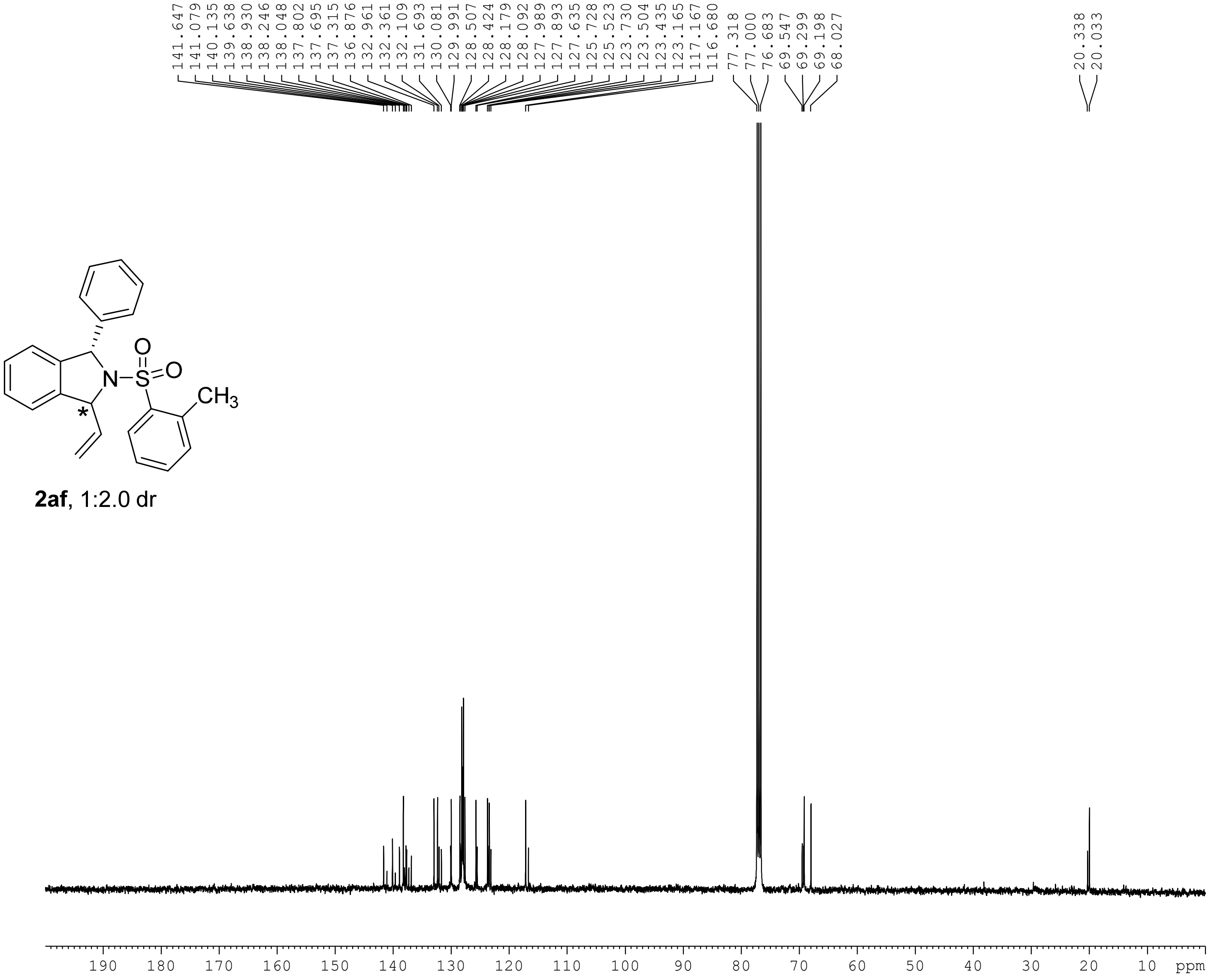

2af, 1:2.0 dr

NAME 202407
EXPNO 251
PROCNO 1
Date_ 20240711
Time 16.32
INSTRUM spect
PROBHD 5 mm PABBO BB/
PULPROG zgpg30
TD 32768
SOLVENT CDCl3
NS 1800
DS 0
SWH 24038.461 Hz
FIDRES 0.733596 Hz
AQ 0.6816244 sec
RG 205.92
DW 20.800 usec
DE 6.50 usec
TE 292.7 K
D1 2.00000000 sec
D11 0.03000000 sec
TD0 1

======== CHANNEL f1 ========
SFO1 100.6233329 MHz
NUC1 13C
P1 10.00 usec
SI 32768
SF 100.6127728 MHz
WDW EM
SSB 0
LB 2.00 Hz
GB 0
PC 1.00

^{1}H NMR of **2ah** ($CDCl_3$, 400 MHz)

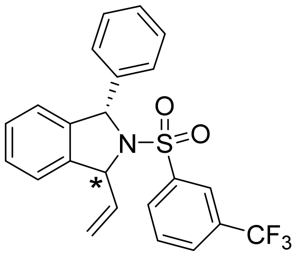

2ah, 1.2:1 dr

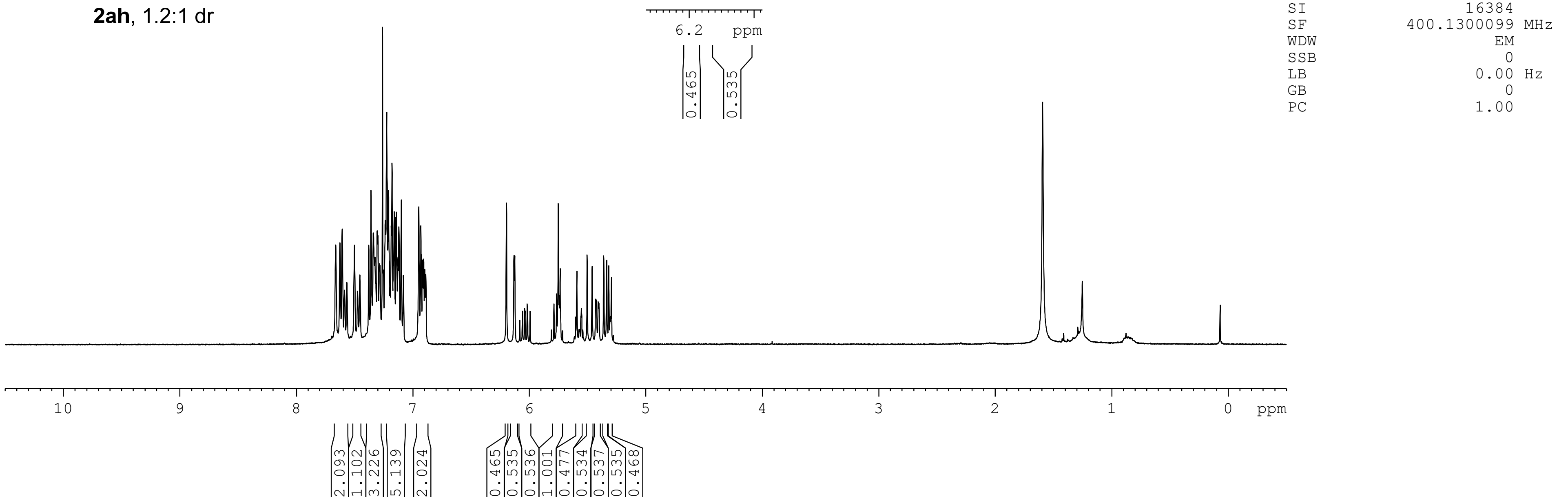

$^{13}C\{^{1}H\}$ NMR of **2ah** ($CDCl_3$, 101 MHz)

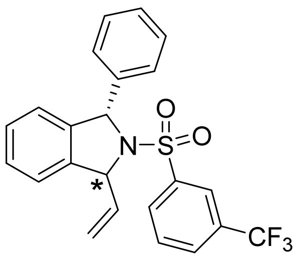

2ah, 1.2:1 dr

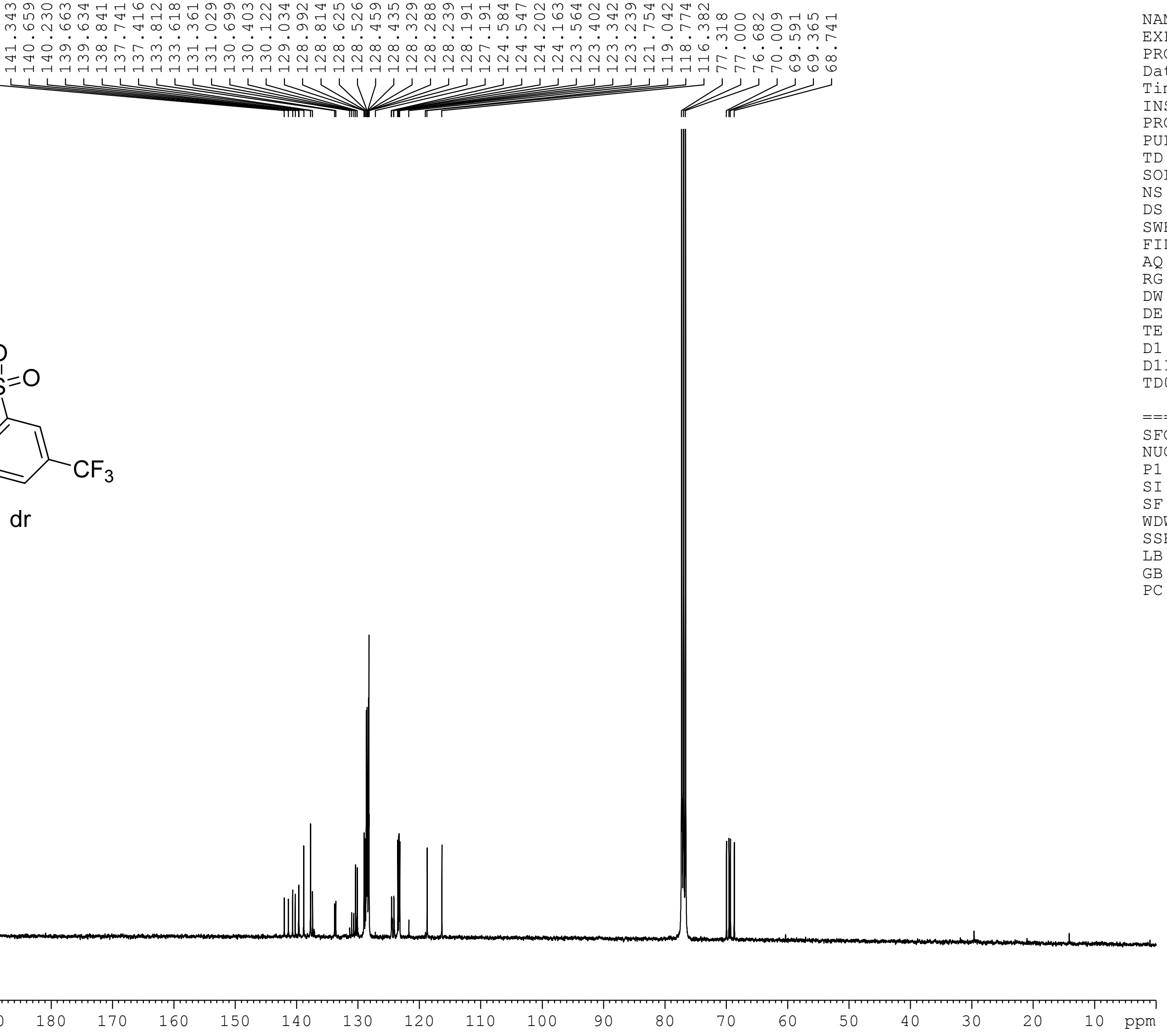

NAME	202404	
EXPNO	283	
PROCNO	1	
Date_	20240420	
Time	18.20	
INSTRUM	spect	
PROBHD	5 mm PABBO BB/	
PULPROG	zgpg30	
TD	32768	
SOLVENT	CDCl3	
NS	15000	
DS	0	
SWH	24038.461	Hz
FIDRES	0.733596	Hz
AQ	0.6816244	sec
RG	205.92	
DW	20.800	usec
DE	6.50	usec
TE	293.0	K
D1	2.00000000	sec
D11	0.03000000	sec
TD0	1	

======== CHANNEL f1 ========

SFO1	100.6233329	MHz
NUC1	13C	
P1	10.00	usec
SI	32768	
SF	100.6127720	MHz
WDW	EM	
SSB	0	
LB	2.00	Hz
GB	0	
PC	1.00	

^{19}F NMR of **2ah** ($CDCl_3$, 376 MHz)

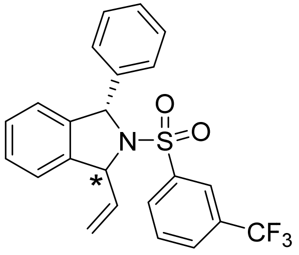

2ah, 1.2:1 dr

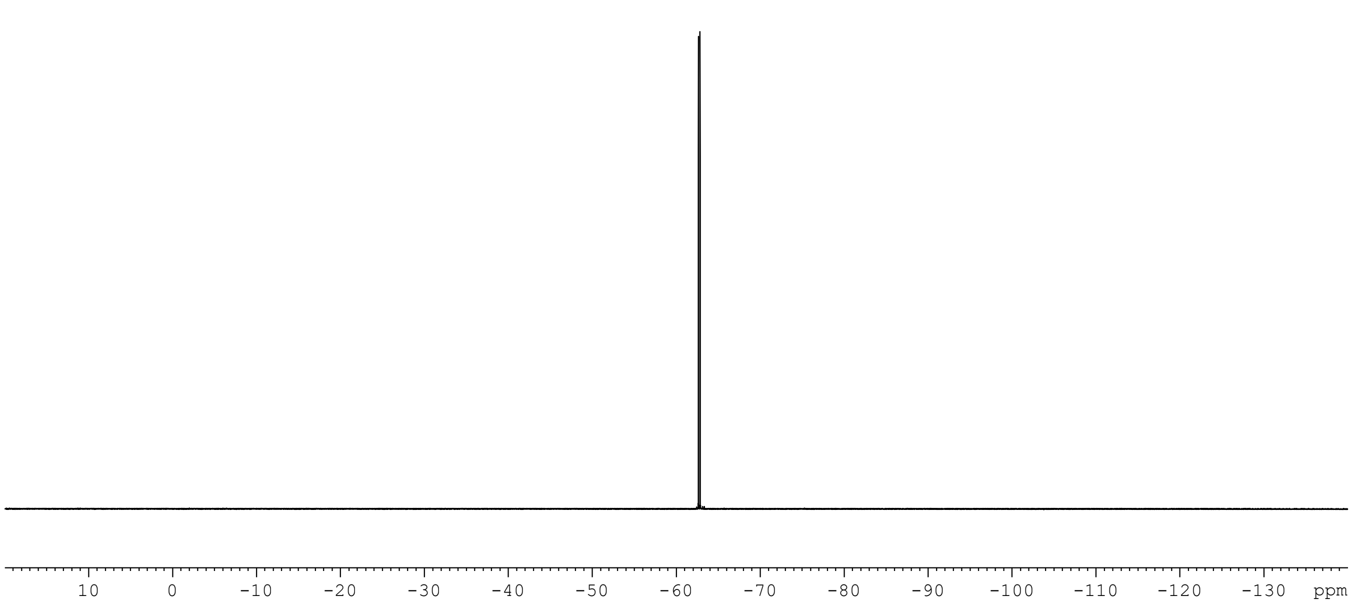


```
NAME              202404
EXPNO                284
PROCNO                 1
Date_           20240421
Time               11.53
INSTRUM            spect
PROBHD   5 mm PABBO BB/
PULPROG             zg30
TD                131072
SOLVENT            CDCl3
NS                    28
DS                     0
SWH            89285.711 Hz
FIDRES          0.681196 Hz
AQ             0.7340532 sec
RG                205.92
DW                 5.600 usec
DE                  6.50 usec
TE                 291.4 K
D1            1.00000000 sec
TD0                    1

======== CHANNEL f1 ========
SFO1         376.4757776 MHz
NUC1                 19F
P1                 15.00 usec
SI                 65536
SF           376.4983662 MHz
WDW                   EM
SSB                    0
LB                  0.30 Hz
GB                     0
PC                  1.00
```


^{1}H NMR of **2bh** ($CDCl_3$, 400 MHz)

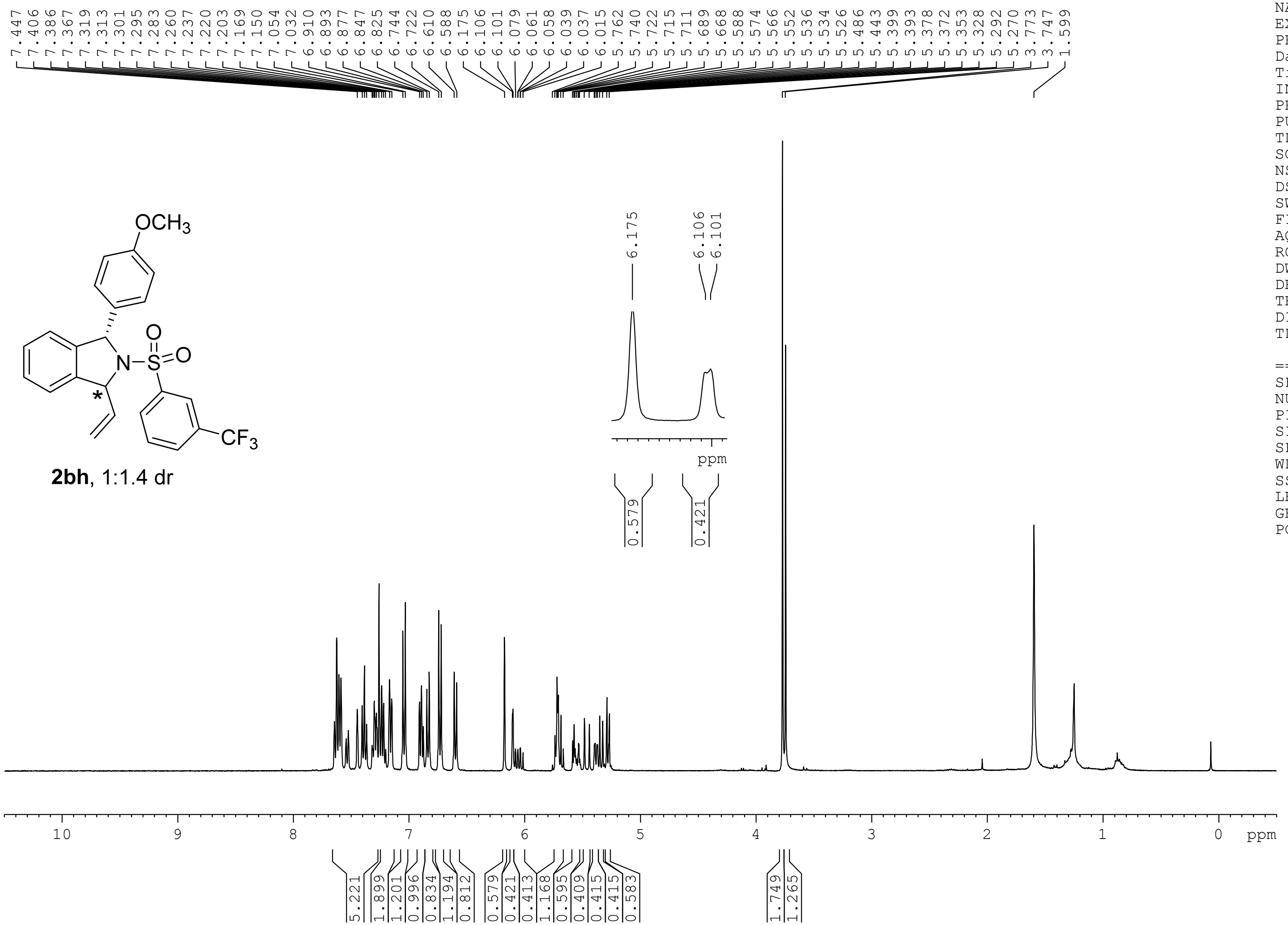

NAME 202408
EXPNO 69
PROCNO 1
Date_ 20240803
Time 16.32
INSTRUM spect
PROBHD 5 mm PABBO BB/
PULPROG zg30
TD 32768
SOLVENT CDCl3
NS 25
DS 0
SWH 8012.820 Hz
FIDRES 0.244532 Hz
AQ 2.0447731 sec
RG 205.92
DW 62.400 usec
DE 16.53 usec
TE 290.3 K
D1 2.00000000 sec
TD0 1

======== CHANNEL f1 ========
SFO1 400.1324008 MHz
NUC1 1H
P1 14.00 usec
SI 16384
SF 400.1300097 MHz
WDW EM
SSB 0
LB 0.00 Hz
GB 0
PC 1.00

$^{13}C\{^1H\}$ NMR of **2bh** ($CDCl_3$, 101 MHz)

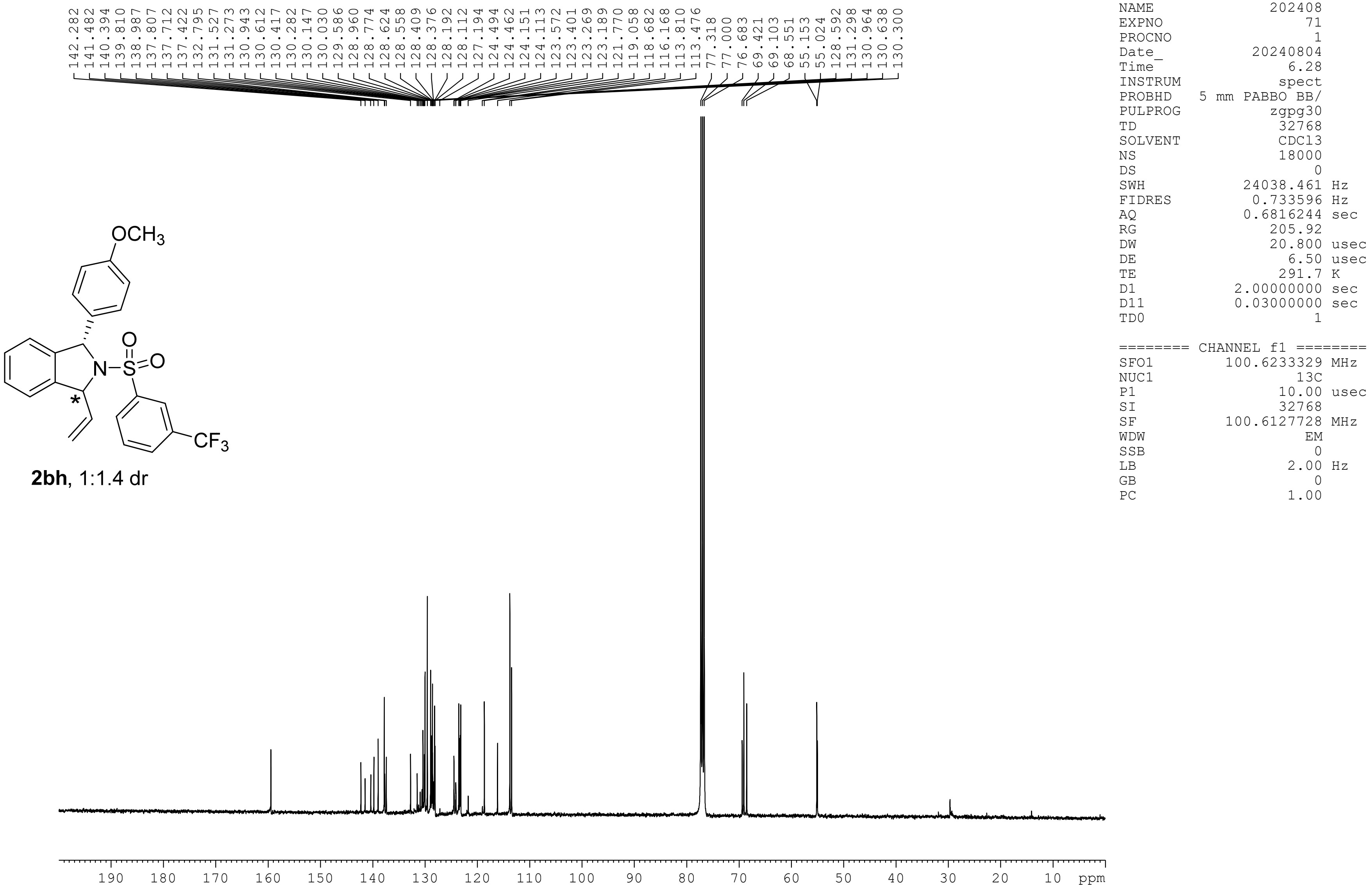

2bh, 1:1.4 dr

^{19}F NMR of **2bh** ($CDCl_3$, 376 MHz)

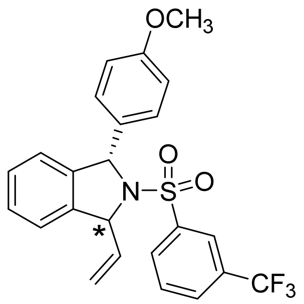

2bh, 1:1.4 dr

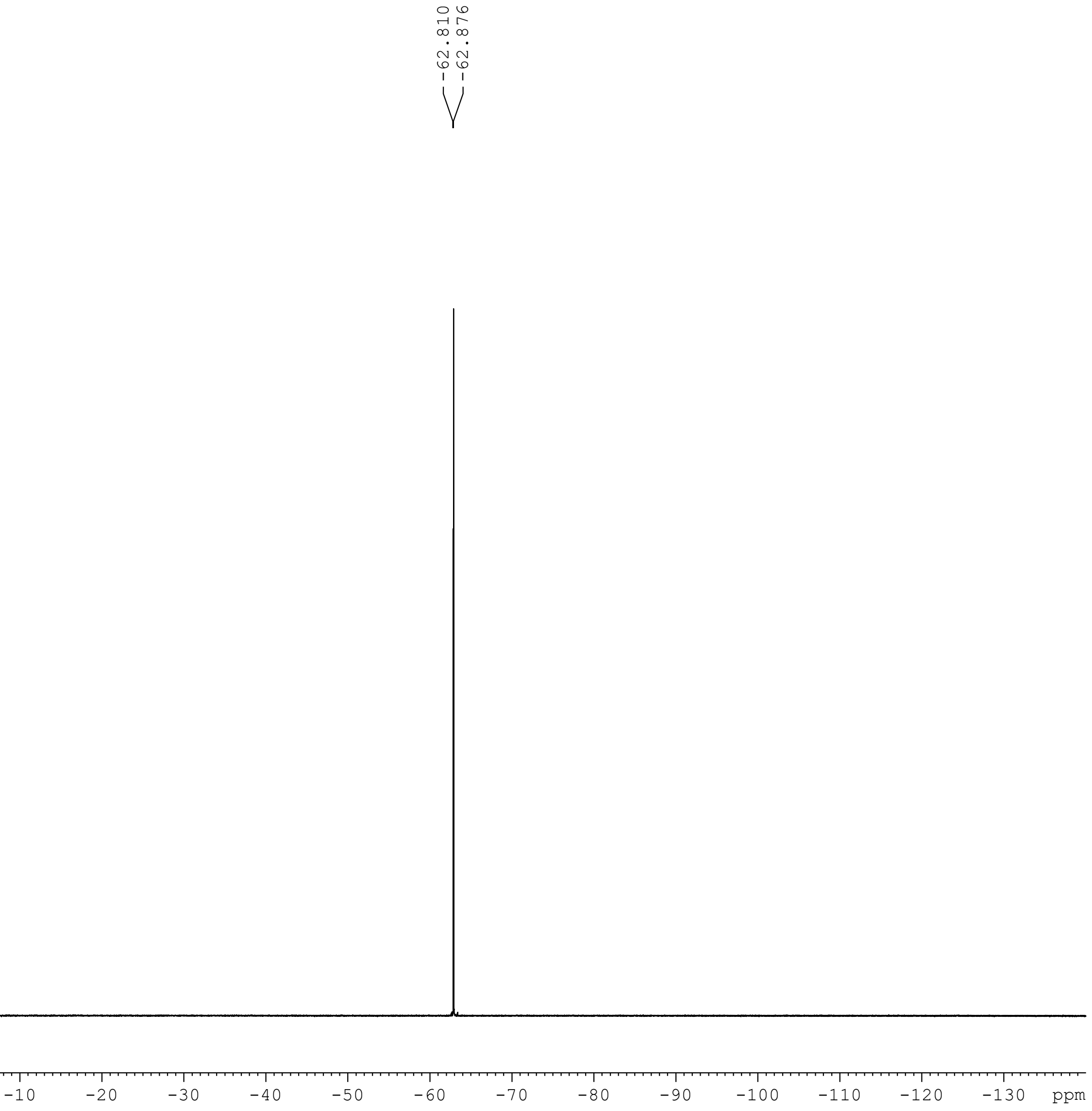


```
NAME               202408
EXPNO                  70
PROCNO                  1
Date_            20240803
Time                16.43
INSTRUM             spect
PROBHD   5 mm PABBO BB/
PULPROG              zg30
TD                 131072
SOLVENT             CDCl3
NS                     20
DS                      0
SWH             89285.711 Hz
FIDRES           0.681196 Hz
AQ              0.7340532 sec
RG                 205.92
DW                  5.600 usec
DE                   6.50 usec
TE                  290.4 K
D1             1.00000000 sec
TD0                     1

======== CHANNEL f1 ========
SFO1          376.4757776 MHz
NUC1                  19F
P1                  15.00 usec
SI                  65536
SF            376.4983662 MHz
WDW                    EM
SSB                     0
LB                   0.30 Hz
GB                      0
PC                   1.00
```

^{1}H NMR of **2ch** ($CDCl_3$, 400 MHz)

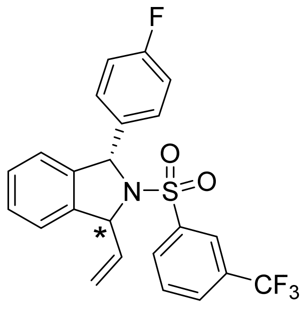

2ch, 1.2:1 dr

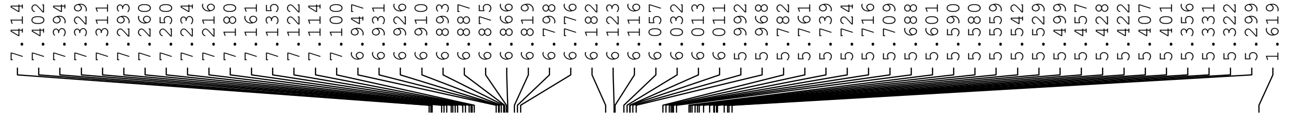

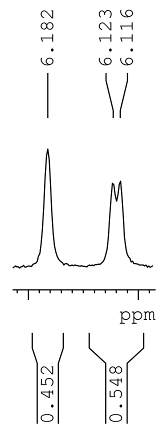

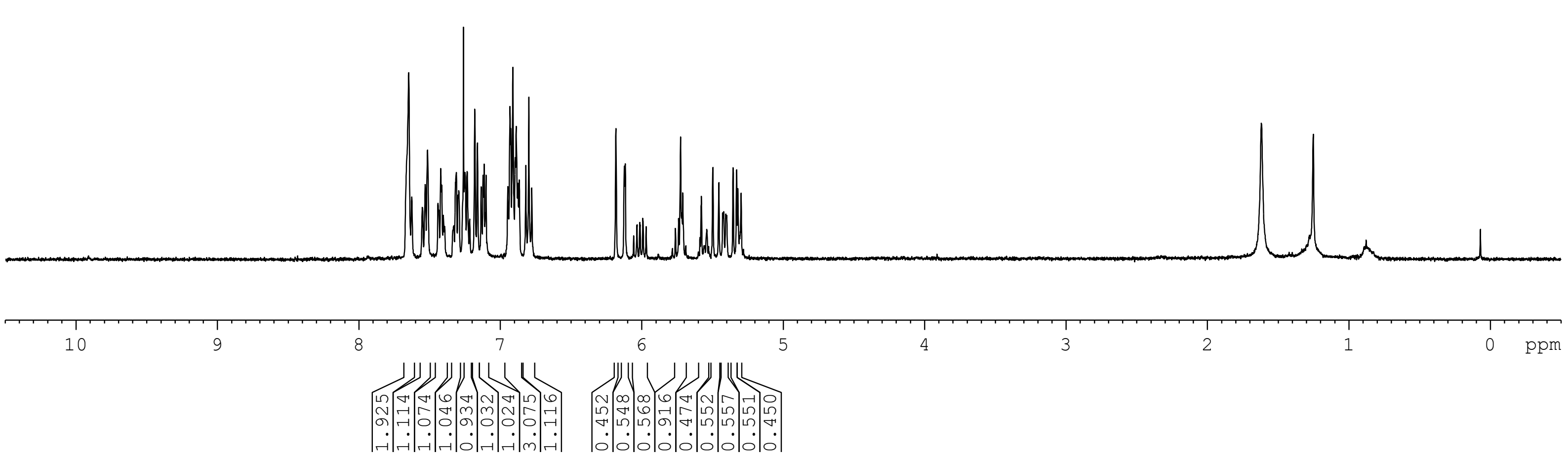

NAME	202407
EXPNO	429
PROCNO	1
Date_	20240719
Time	16.23
INSTRUM	spect
PROBHD	5 mm PABBO BB/
PULPROG	zg30
TD	32768
SOLVENT	CDCl3
NS	30
DS	0
SWH	8012.820 Hz
FIDRES	0.244532 Hz
AQ	2.0447731 sec
RG	205.92
DW	62.400 usec
DE	16.53 usec
TE	291.7 K
D1	2.00000000 sec
TD0	1

======== CHANNEL f1 ========

SFO1	400.1324008 MHz
NUC1	1H
P1	14.00 usec
SI	16384
SF	400.1300096 MHz
WDW	EM
SSB	0
LB	0.00 Hz
GB	0
PC	1.00

$^{13}C\{^{1}H\}$ NMR of **2ch** ($CDCl_3$, 101 MHz)

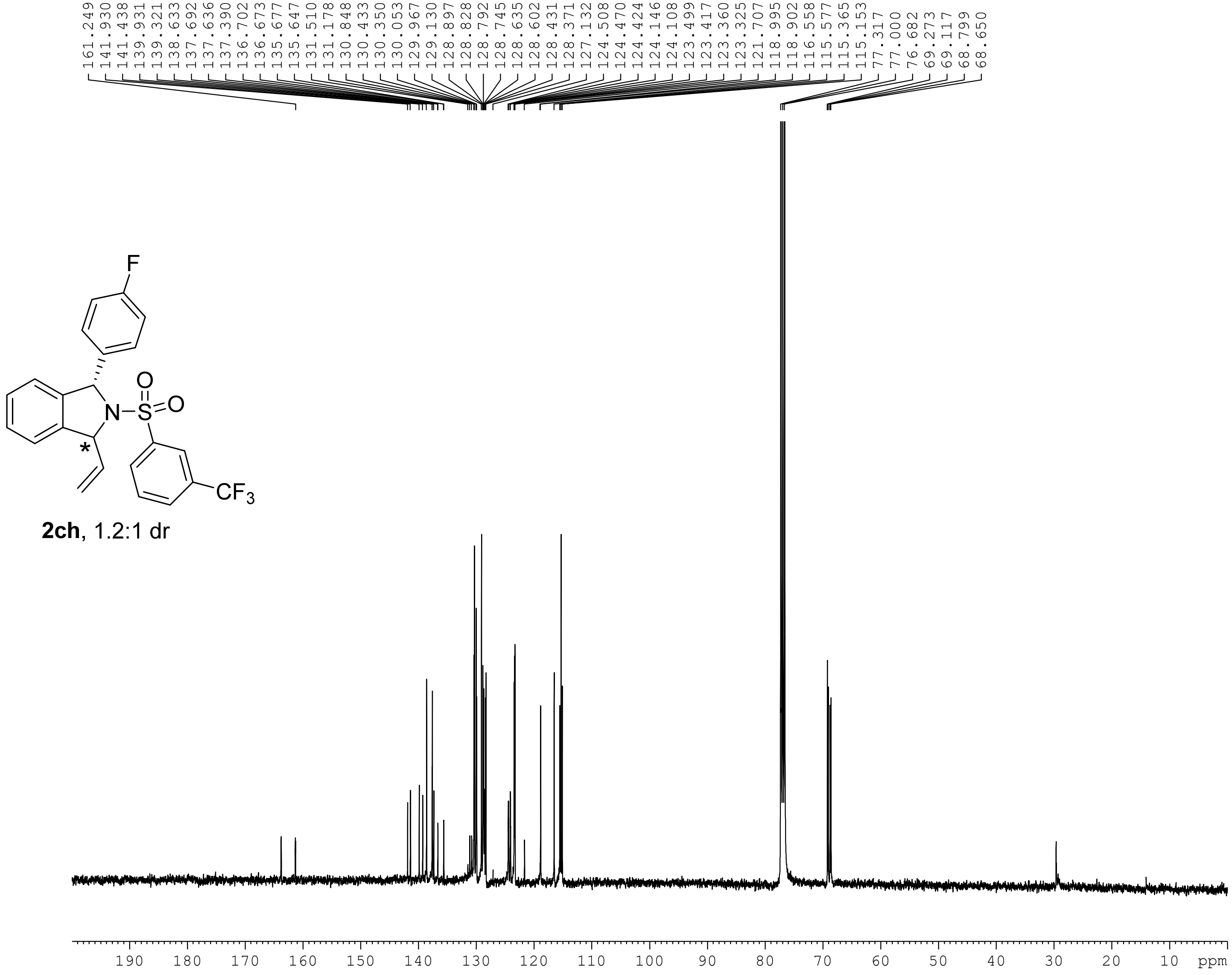

2ch, 1.2:1 dr

NAME	202407
EXPNO	447
PROCNO	1
Date_	20240720
Time	8.22
INSTRUM	spect
PROBHD	5 mm PABBO BB/
PULPROG	zgpg30
TD	32768
SOLVENT	CDCl3
NS	8000
DS	0
SWH	24038.461 Hz
FIDRES	0.733596 Hz
AQ	0.6816244 sec
RG	205.92
DW	20.800 usec
DE	6.50 usec
TE	292.2 K
D1	2.00000000 sec
D11	0.03000000 sec
TD0	1

======== CHANNEL f1 ========

SFO1	100.6233329 MHz
NUC1	13C
P1	10.00 usec
SI	32768
SF	100.6127724 MHz
WDW	EM
SSB	0
LB	2.00 Hz
GB	0
PC	1.00

^{19}F NMR of **2ch** ($CDCl_3$, 376 MHz)

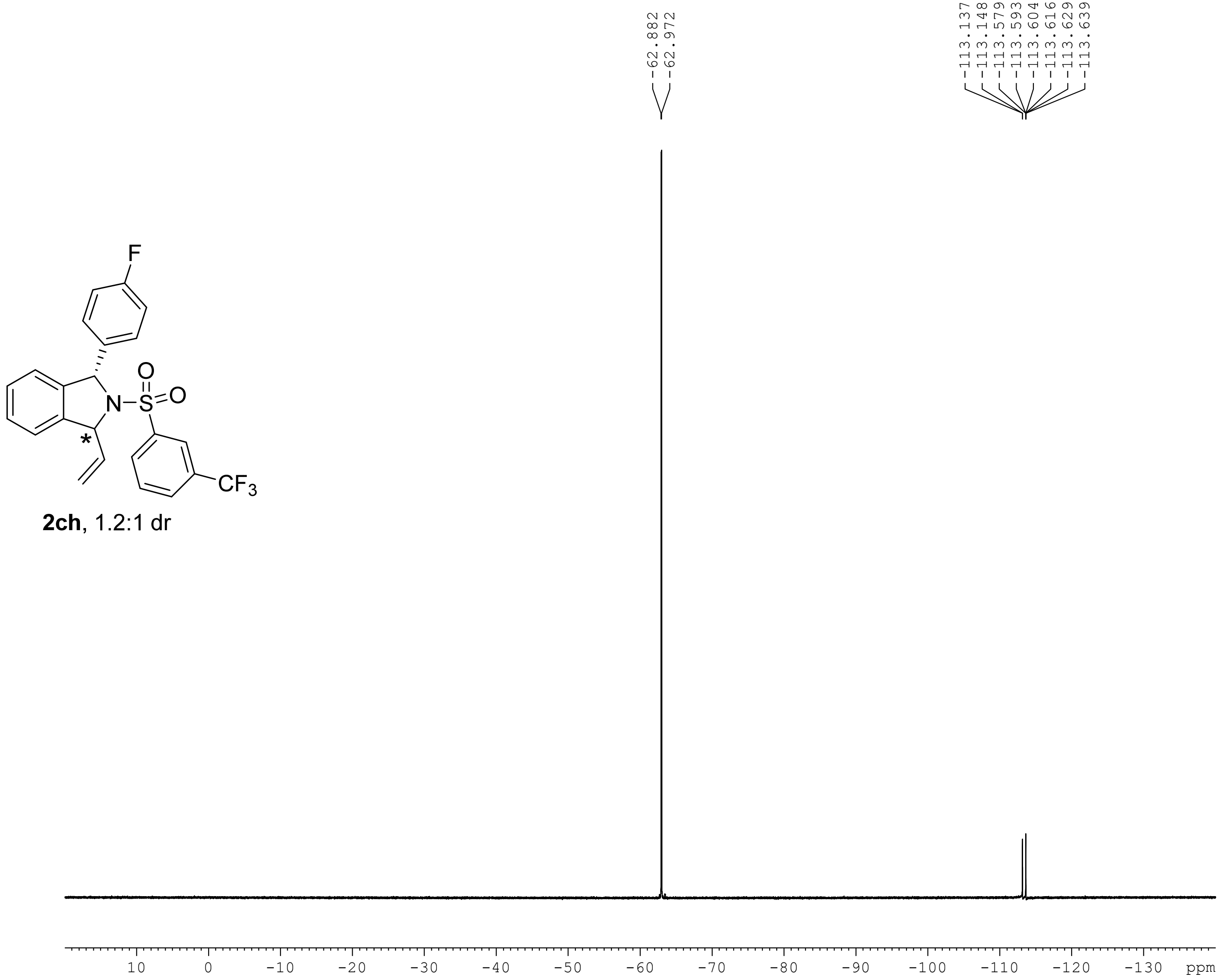

NAME 202407
EXPNO 448
PROCNO 1
Date_ 20240720
Time 8.24
INSTRUM spect
PROBHD 5 mm PABBO BB/
PULPROG zg30
TD 131072
SOLVENT CDCl3
NS 8
DS 0
SWH 89285.711 Hz
FIDRES 0.681196 Hz
AQ 0.7340532 sec
RG 205.92
DW 5.600 usec
DE 6.50 usec
TE 291.4 K
D1 1.00000000 sec
TD0 1

======== CHANNEL f1 ========
SFO1 376.4757776 MHz
NUC1 19F
P1 15.00 usec
SI 65536
SF 376.4983662 MHz
WDW EM
SSB 0
LB 0.30 Hz
GB 0
PC 1.00

^{1}H NMR of **2dh** ($CDCl_3$, 400 MHz)

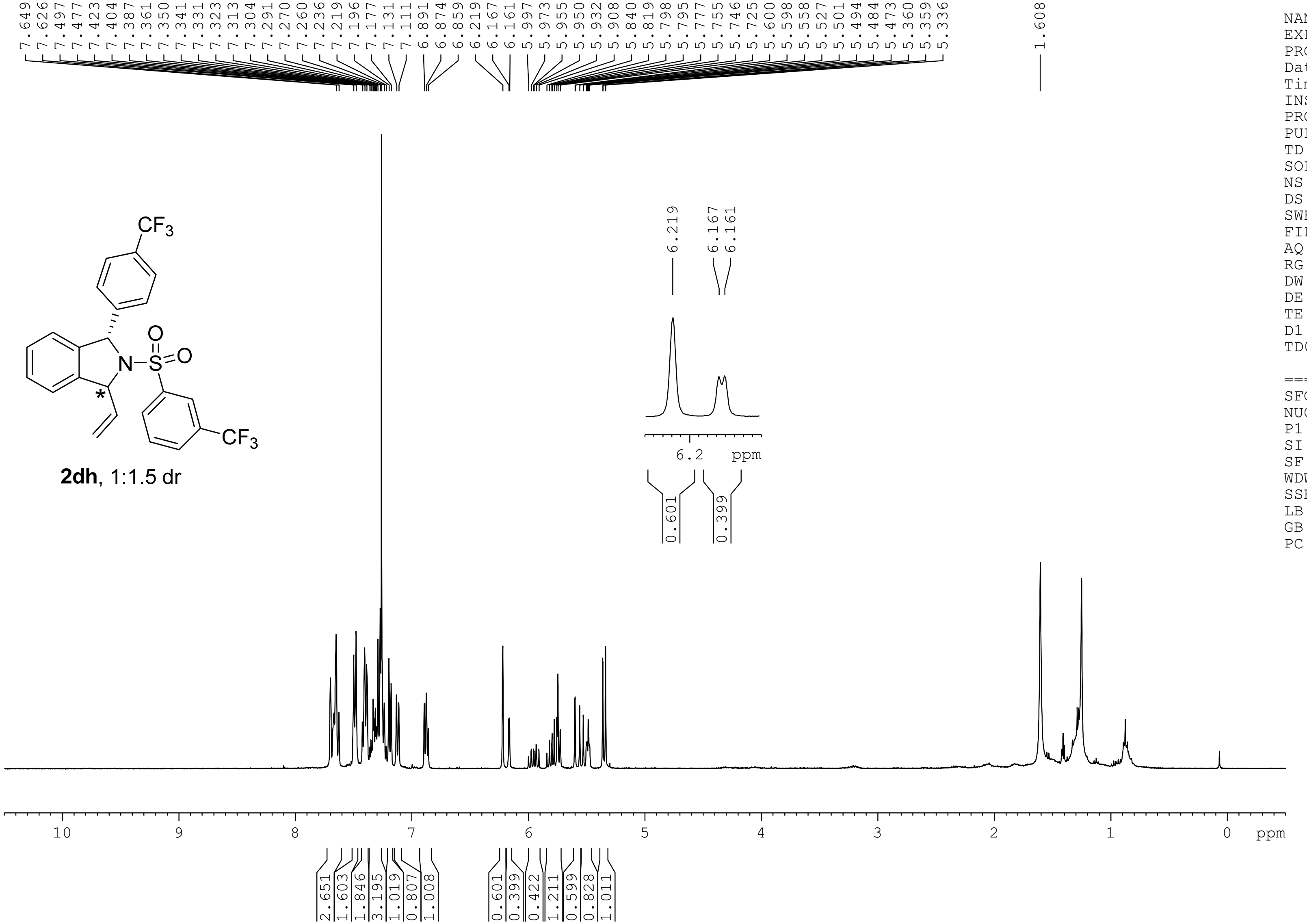


```
NAME              202408
EXPNO                468
PROCNO                 1
Date_           20240829
Time               15.33
INSTRUM            spect
PROBHD   5 mm PABBO BB/
PULPROG             zg30
TD                 32768
SOLVENT            CDCl3
NS                    30
DS                     0
SWH             8012.820 Hz
FIDRES          0.244532 Hz
AQ             2.0447731 sec
RG                205.92
DW                62.400 usec
DE                 16.53 usec
TE                 291.6 K
D1            2.00000000 sec
TD0                    1

======== CHANNEL f1 ========
SFO1         400.1324008 MHz
NUC1                  1H
P1                 14.00 usec
SI                 16384
SF           400.1300096 MHz
WDW                   EM
SSB                    0
LB                  0.00 Hz
GB                     0
PC                  1.00
```

$^{13}C\{^{1}H\}$ NMR of **2dh** ($CDCl_3$, 101 MHz)

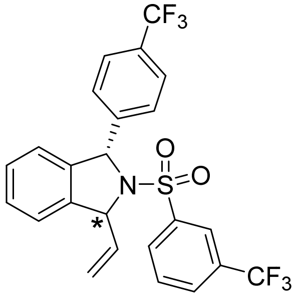

2dh, 1:1.5 dr

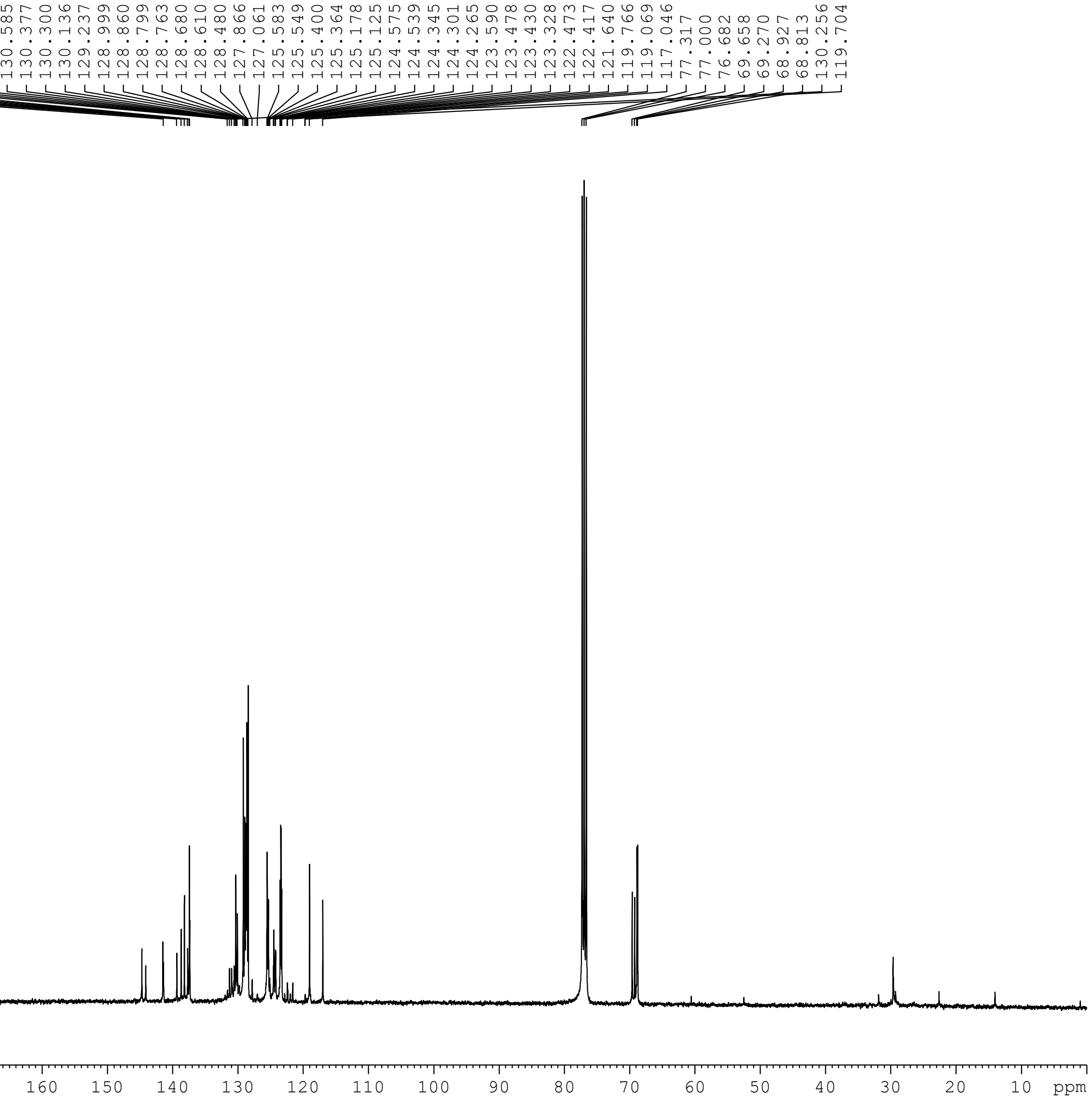

NAME	202409	
EXPNO	54	
PROCNO	1	
Date_	20240913	
Time	3.24	
INSTRUM	spect	
PROBHD	5 mm PABBO BB/	
PULPROG	zgpg30	
TD	32768	
SOLVENT	CDCl3	
NS	10000	
DS	0	
SWH	24038.461	Hz
FIDRES	0.733596	Hz
AQ	0.6816244	sec
RG	205.92	
DW	20.800	usec
DE	6.50	usec
TE	298.5	K
D1	2.00000000	sec
D11	0.03000000	sec
TD0	1	

======== CHANNEL f1 ========

SFO1	100.6233329	MHz
NUC1	13C	
P1	10.00	usec
SI	32768	
SF	100.6127718	MHz
WDW	EM	
SSB	0	
LB	2.00	Hz
GB	0	
PC	1.00	

^{19}F NMR of **2dh** ($CDCl_3$, 376 MHz)

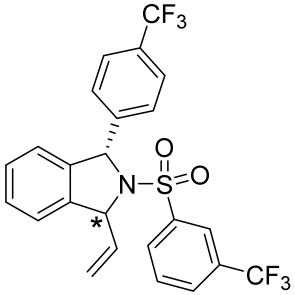

2dh, 1:1.5 dr

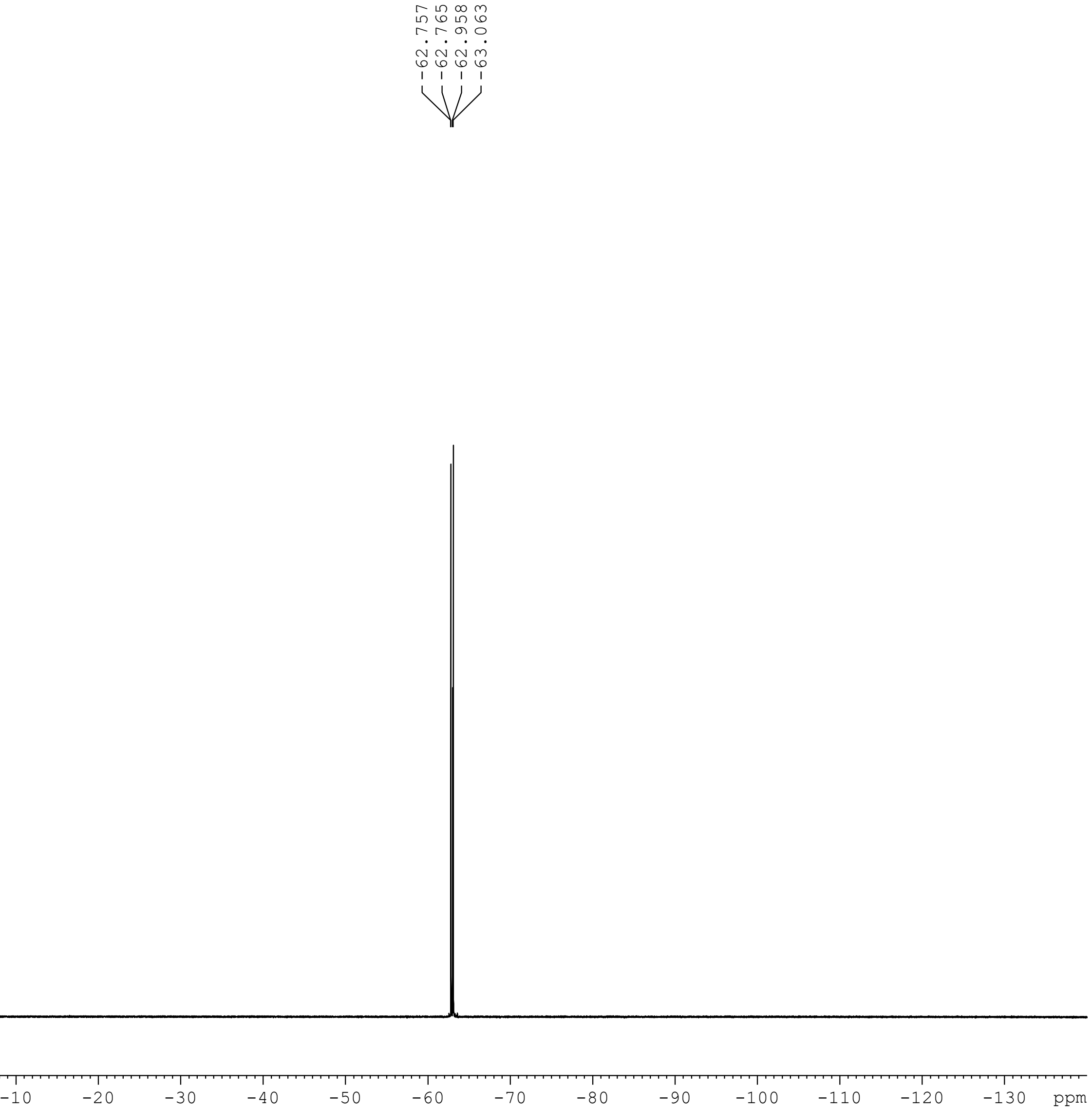

NAME	202408	
EXPNO	473	
PROCNO	1	
Date_	20240829	
Time	15.50	
INSTRUM	spect	
PROBHD	5 mm PABBO BB/	
PULPROG	zg30	
TD	131072	
SOLVENT	CDCl3	
NS	20	
DS	0	
SWH	89285.711	Hz
FIDRES	0.681196	Hz
AQ	0.7340532	sec
RG	205.92	
DW	5.600	usec
DE	6.50	usec
TE	291.6	K
D1	1.00000000	sec
TD0	1	
======== CHANNEL f1 ========		
SFO1	376.4757776	MHz
NUC1	19F	
P1	15.00	usec
SI	65536	
SF	376.4983662	MHz
WDW	EM	
SSB	0	
LB	0.30	Hz
GB	0	
PC	1.00	

^{1}H NMR of **2eh** ($CDCl_3$, 400 MHz)

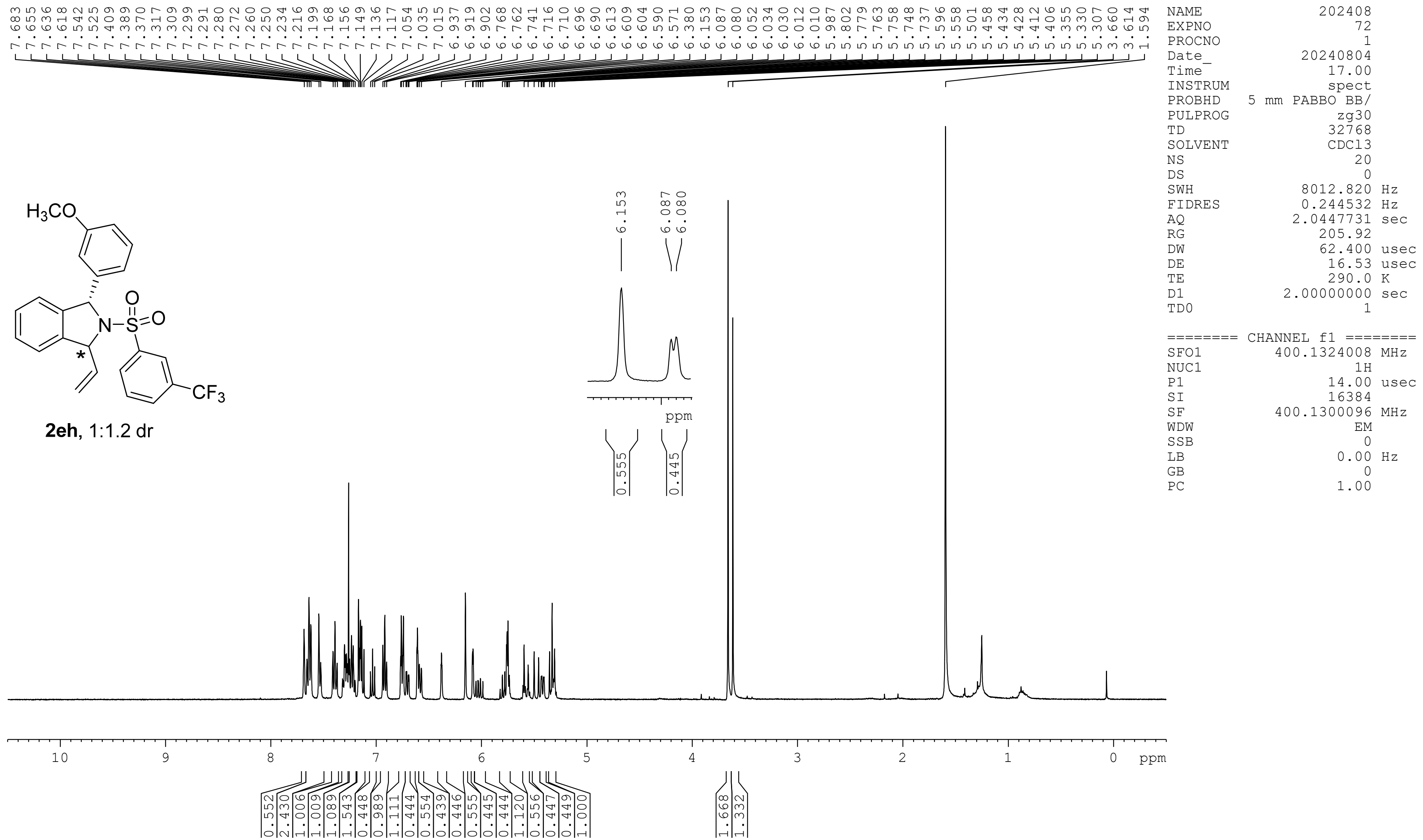

2eh, 1:1.2 dr

$^{13}C\{^{1}H\}$ NMR of **2eh** ($CDCl_3$, 101 MHz)

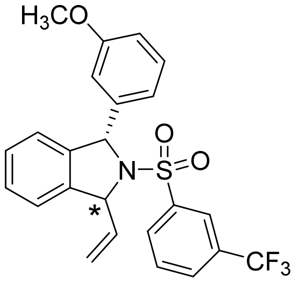

2eh, 1:1.2 dr

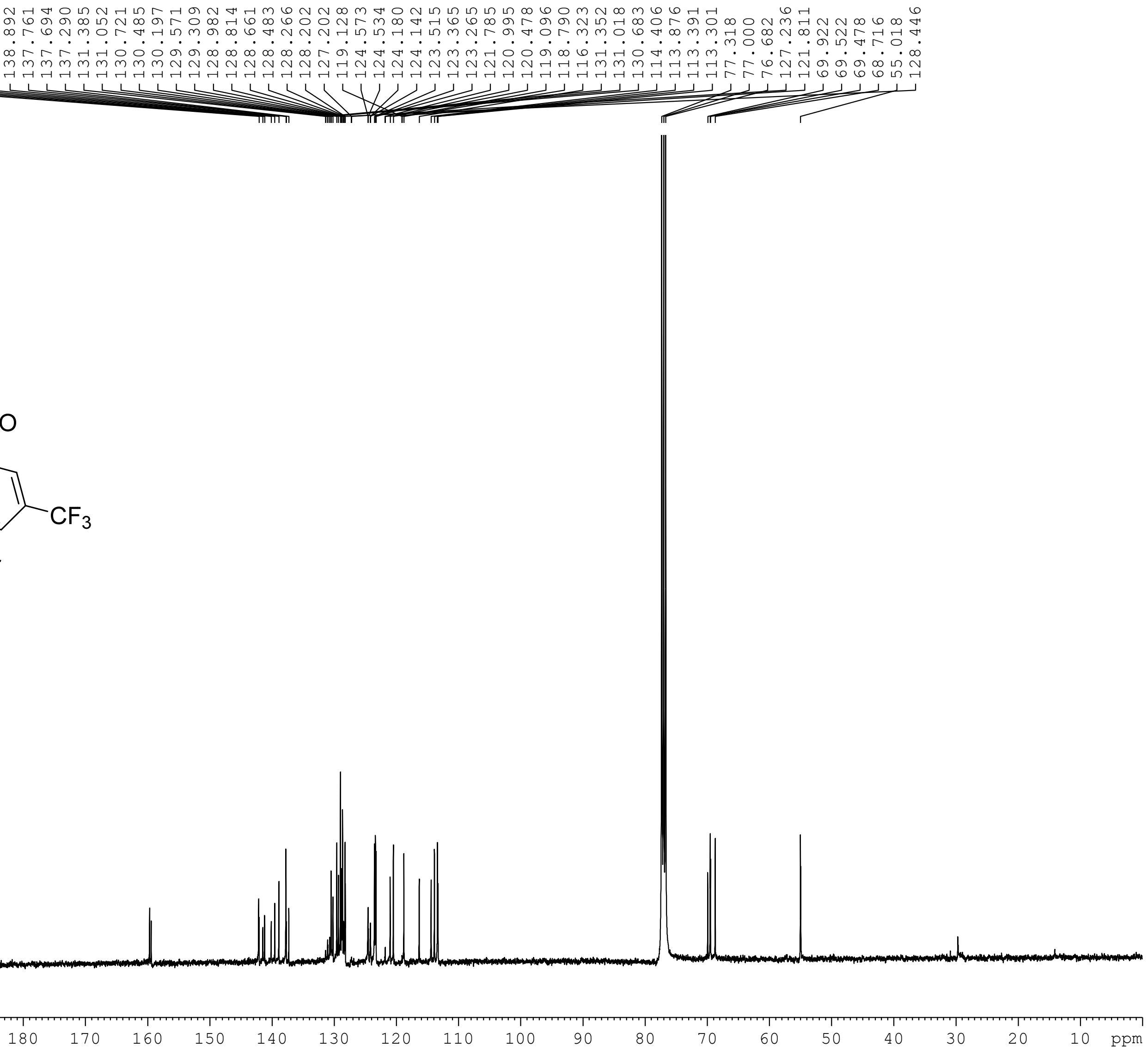

NAME	202408
EXPNO	77
PROCNO	1
Date_	20240805
Time	9.57
INSTRUM	spect
PROBHD	5 mm PABBO BB/
PULPROG	zgpg30
TD	32768
SOLVENT	CDCl3
NS	14563
DS	0
SWH	24038.461 Hz
FIDRES	0.733596 Hz
AQ	0.6816244 sec
RG	205.92
DW	20.800 usec
DE	6.50 usec
TE	297.5 K
D1	2.00000000 sec
D11	0.03000000 sec
TD0	1

======== CHANNEL f1 ========

SFO1	100.6233329 MHz
NUC1	13C
P1	10.00 usec
SI	32768
SF	100.6127706 MHz
WDW	EM
SSB	0
LB	2.00 Hz
GB	0
PC	1.00

^{19}F NMR of **2eh** ($CDCl_3$, 376 MHz)

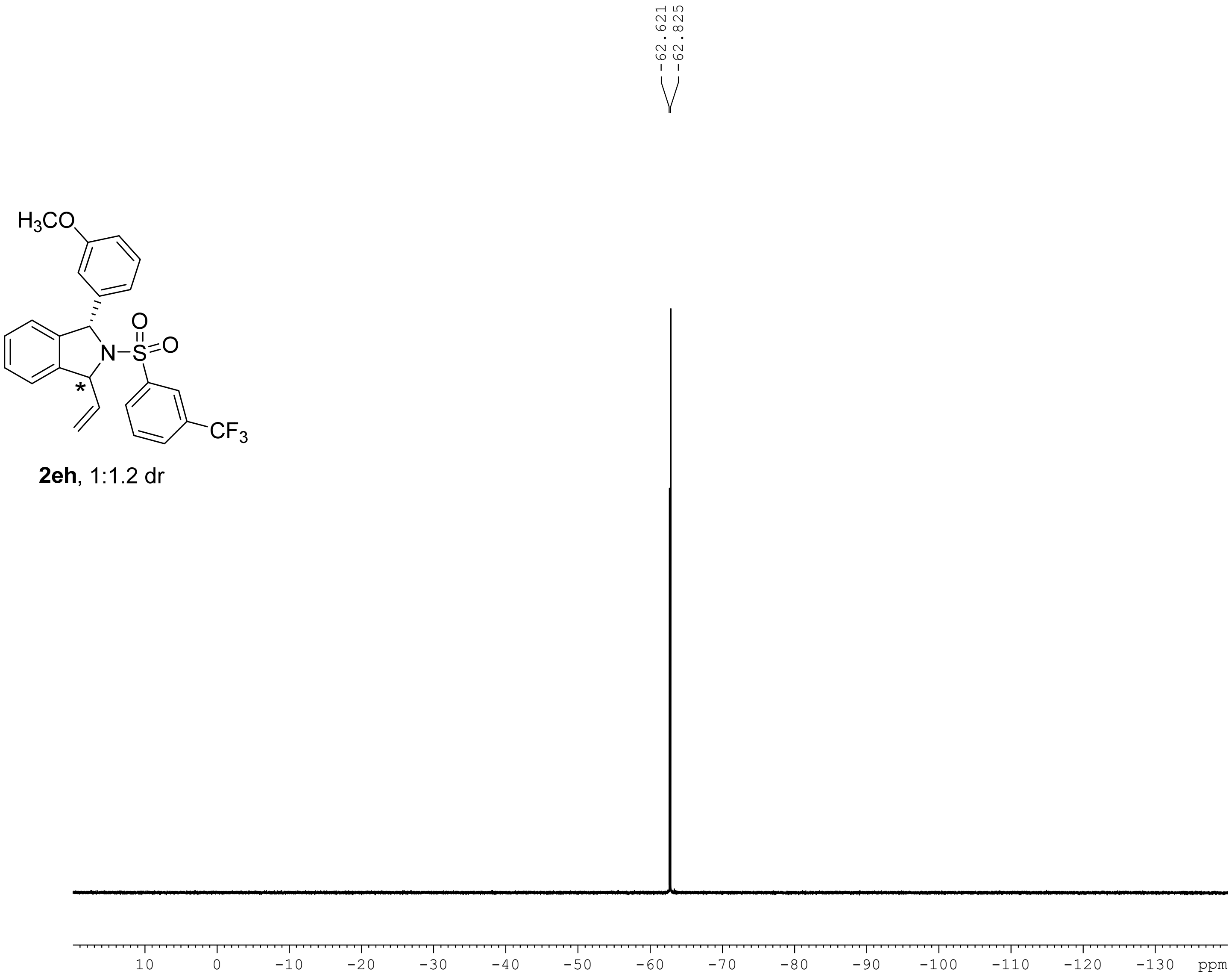

2eh, 1:1.2 dr

NAME	202408
EXPNO	76
PROCNO	1
Date_	20240804
Time	17.53
INSTRUM	spect
PROBHD	5 mm PABBO BB/
PULPROG	zg30
TD	131072
SOLVENT	CDCl3
NS	6
DS	0
SWH	89285.711 Hz
FIDRES	0.681196 Hz
AQ	0.7340532 sec
RG	205.92
DW	5.600 usec
DE	6.50 usec
TE	290.1 K
D1	1.00000000 sec
TD0	1
======== CHANNEL f1 ========	
SFO1	376.4757776 MHz
NUC1	19F
P1	15.00 usec
SI	65536
SF	376.4983662 MHz
WDW	EM
SSB	0
LB	0.30 Hz
GB	0
PC	1.00

^{1}H NMR of **2fh** ($CDCl_3$, 400 MHz)

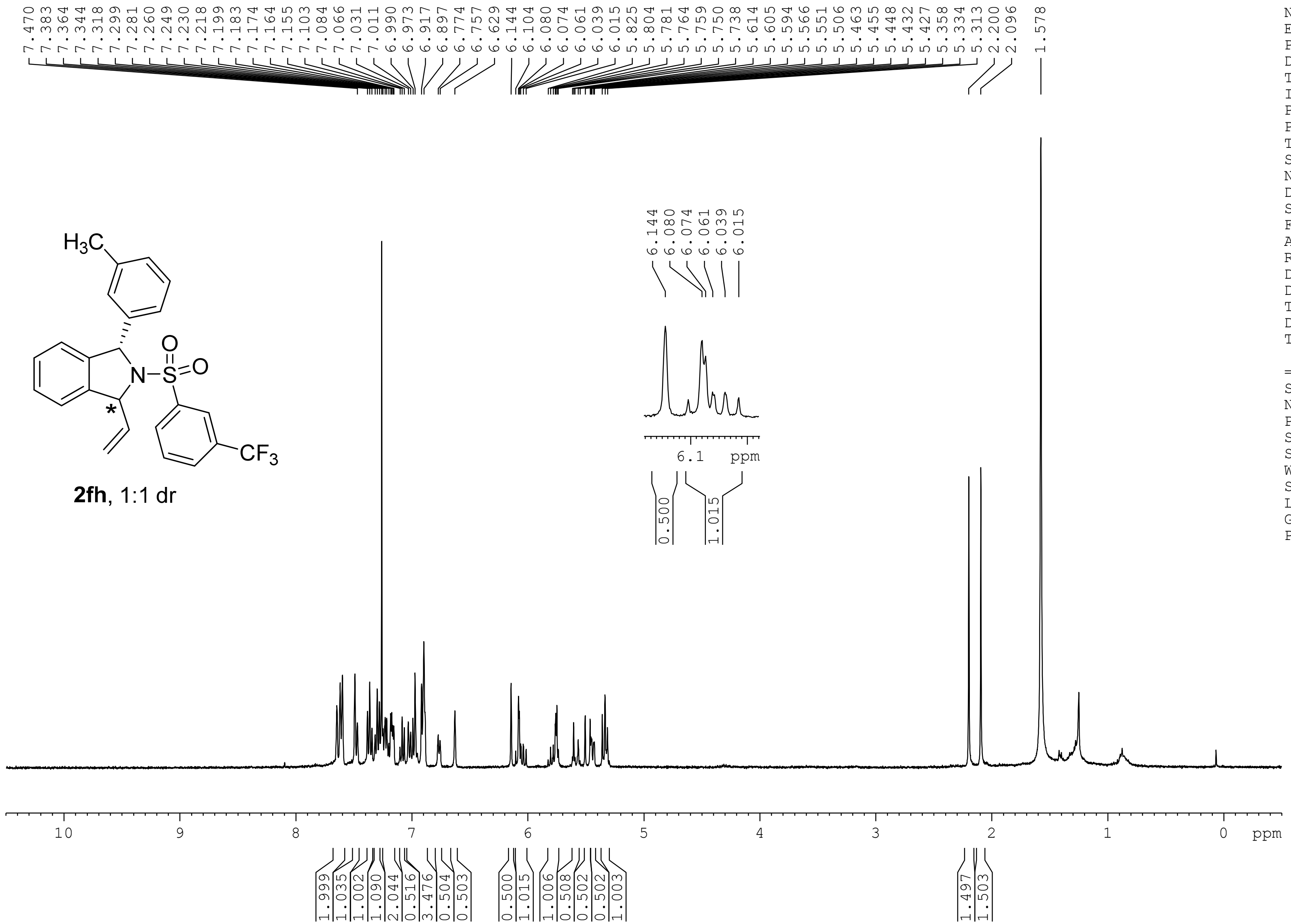

2fh, 1:1 dr

NAME	202408
EXPNO	155
PROCNO	1
Date_	20240808
Time	17.56
INSTRUM	spect
PROBHD	5 mm PABBO BB/
PULPROG	zg30
TD	32768
SOLVENT	CDCl3
NS	16
DS	0
SWH	8012.820 Hz
FIDRES	0.244532 Hz
AQ	2.0447731 sec
RG	205.92
DW	62.400 usec
DE	16.53 usec
TE	291.6 K
D1	2.00000000 sec
TD0	1

======== CHANNEL f1 ========

SFO1	400.1324008 MHz
NUC1	1H
P1	14.00 usec
SI	16384
SF	400.1300100 MHz
WDW	EM
SSB	0
LB	0.00 Hz
GB	0
PC	1.00

$^{13}C\{^{1}H\}$ NMR of **2fh** ($CDCl_3$, 101 MHz)

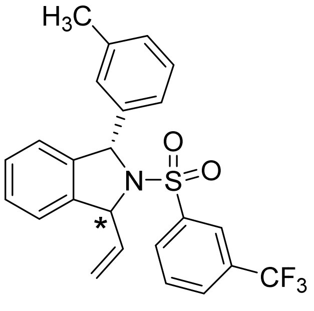

2fh, 1:1 dr

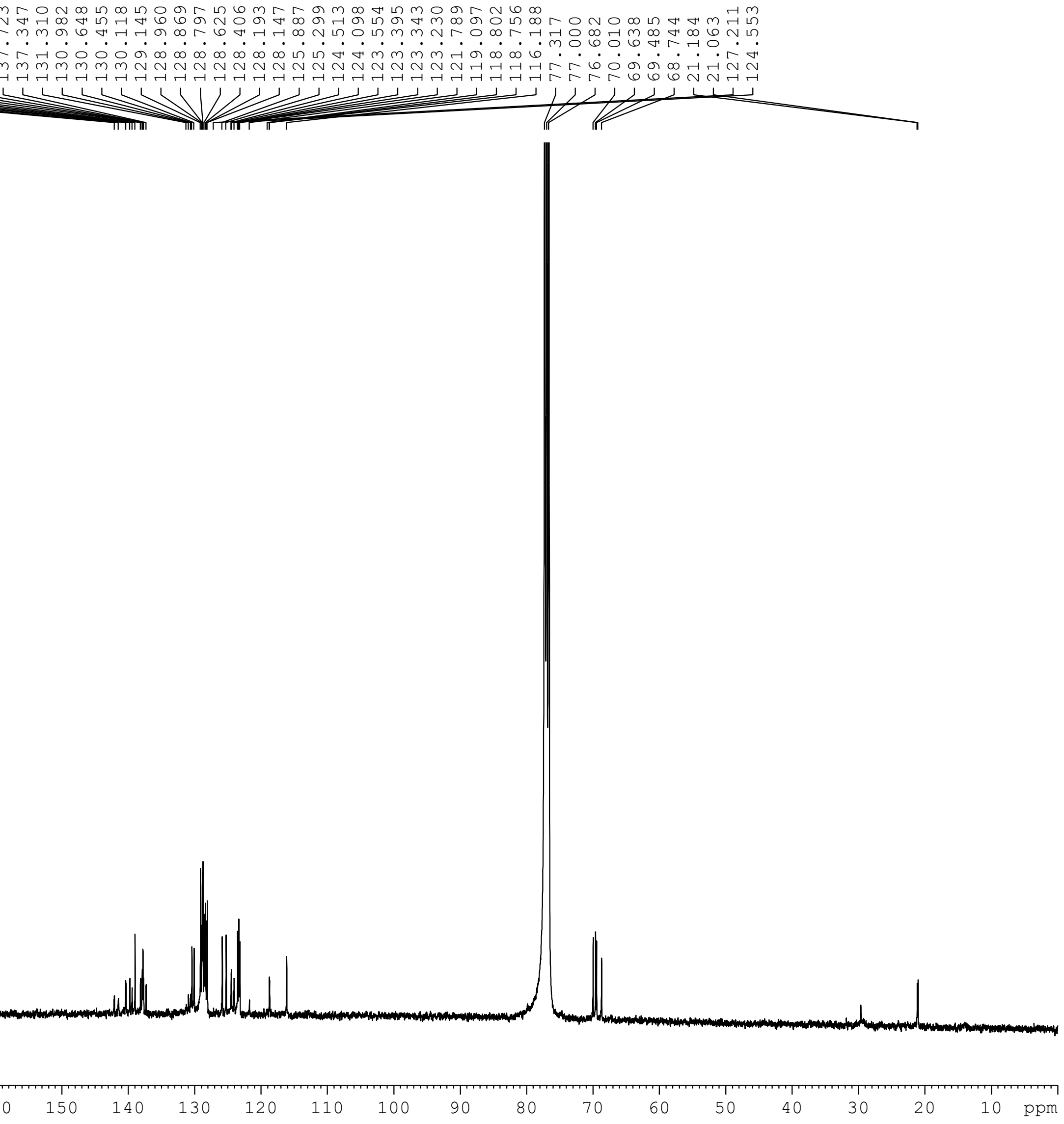

NAME	202408
EXPNO	193
PROCNO	1
Date_	20240908
Time	16.58
INSTRUM	spect
PROBHD	5 mm PABBO BB/
PULPROG	zgpg30
TD	32768
SOLVENT	CDCl3
NS	85220
DS	0
SWH	24038.461 Hz
FIDRES	0.733596 Hz
AQ	0.6816244 sec
RG	205.92
DW	20.800 usec
DE	6.50 usec
TE	290.9 K
D1	2.00000000 sec
D11	0.03000000 sec
TD0	1

======== CHANNEL f1 ========

SFO1	100.6233329 MHz
NUC1	13C
P1	10.00 usec
SI	32768
SF	100.6127704 MHz
WDW	EM
SSB	0
LB	2.00 Hz
GB	0
PC	1.00

^{19}F NMR of **2fh** ($CDCl_3$, 376 MHz)

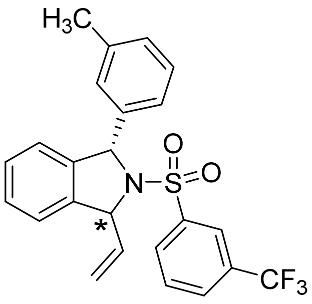

2fh, 1:1 dr

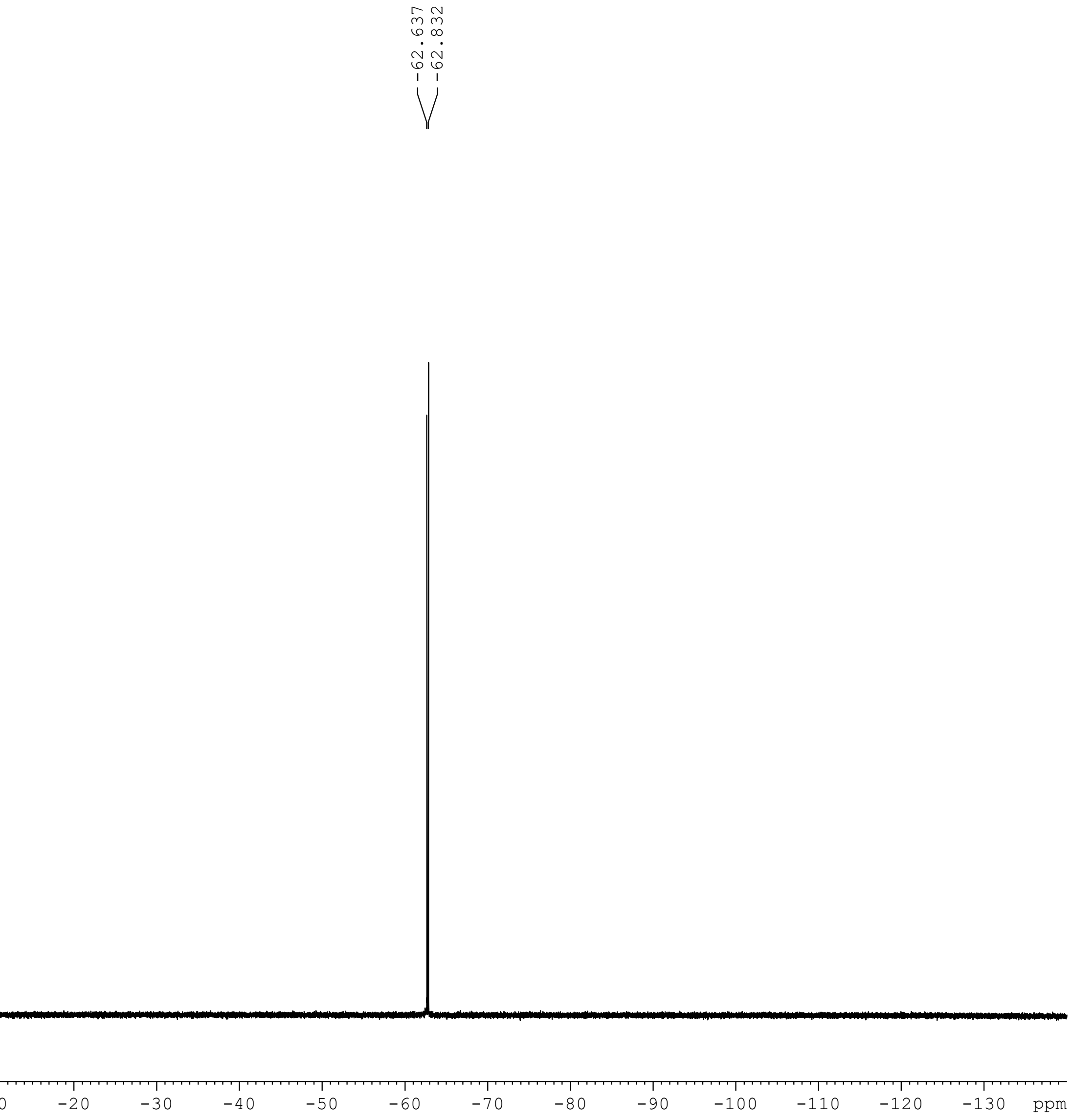


```
NAME              202408
EXPNO                169
PROCNO                 1
Date_           20240809
Time                2.51
INSTRUM            spect
PROBHD   5 mm PABBO BB/
PULPROG             zg30
TD                131072
SOLVENT            CDCl3
NS                    15
DS                     0
SWH            89285.711 Hz
FIDRES          0.681196 Hz
AQ             0.7340532 sec
RG                205.92
DW                 5.600 usec
DE                  6.50 usec
TE                 292.2 K
D1            1.00000000 sec
TD0                    1

======== CHANNEL f1 ========
SFO1         376.4757776 MHz
NUC1                 19F
P1                 15.00 usec
SI                 65536
SF           376.4983662 MHz
WDW                   EM
SSB                    0
LB                  0.30 Hz
GB                     0
PC                  1.00
```

^{1}H NMR of **2gh** ($CDCl_3$, 400 MHz)

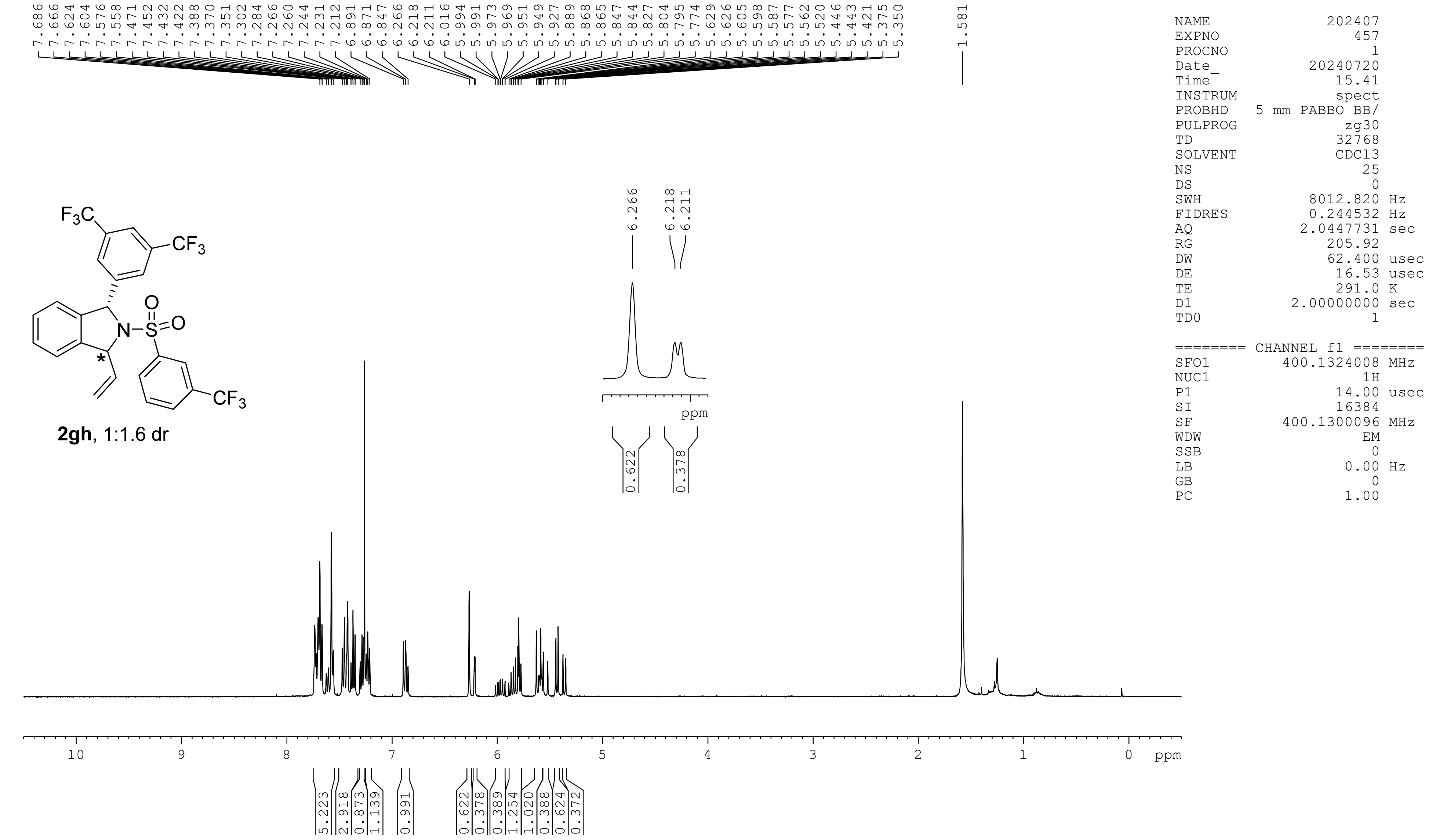

$^{13}C\{^1H\}$ NMR of **2gh** ($CDCl_3$, 101 MHz)

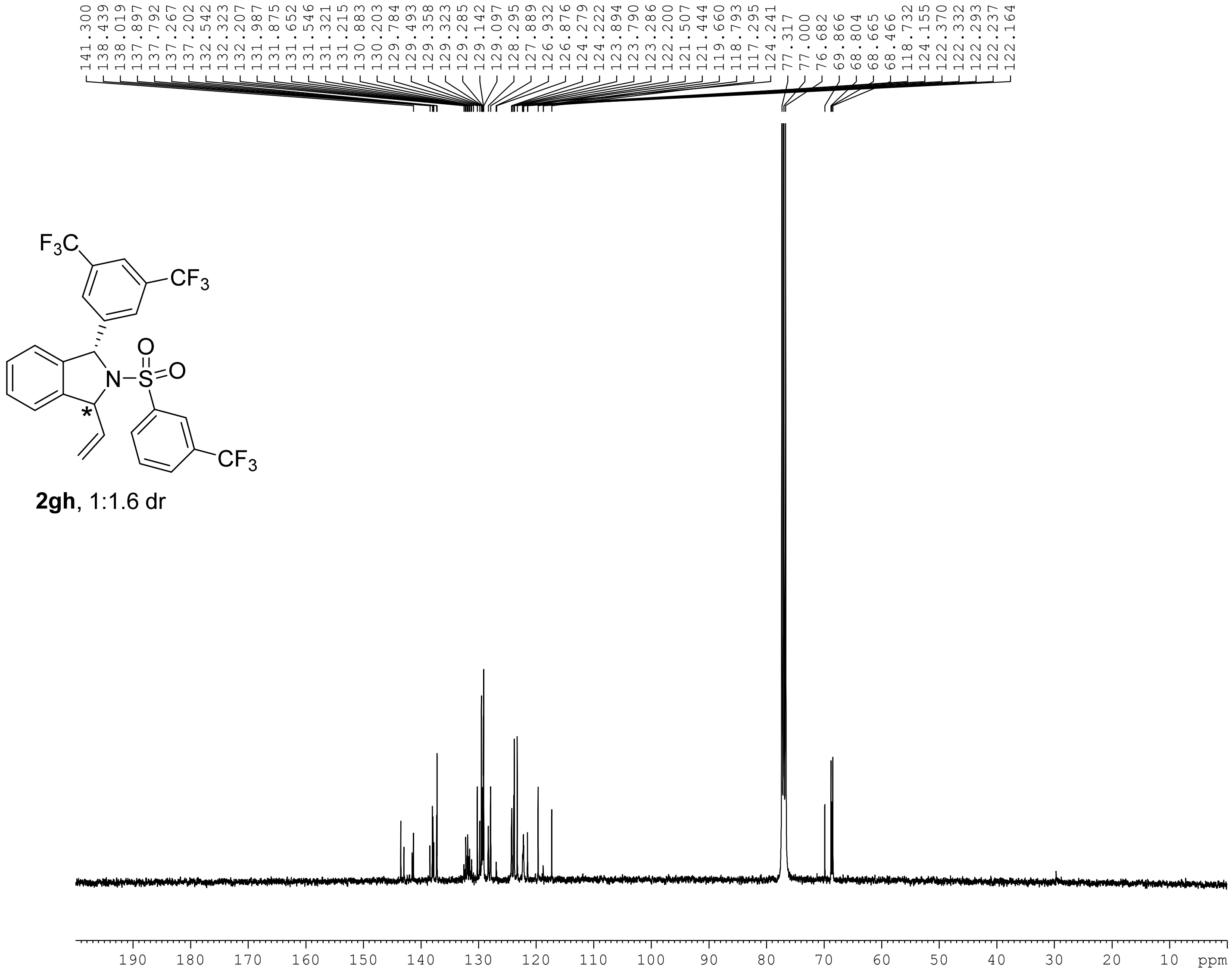

2gh, 1:1.6 dr

NAME	202407	
EXPNO	485	
PROCNO	1	
Date_	20240722	
Time	6.04	
INSTRUM	spect	
PROBHD	5 mm PABBO BB/	
PULPROG	zgpg30	
TD	32768	
SOLVENT	CDCl3	
NS	12000	
DS	0	
SWH	24038.461	Hz
FIDRES	0.733596	Hz
AQ	0.6816244	sec
RG	205.92	
DW	20.800	usec
DE	6.50	usec
TE	292.8	K
D1	2.00000000	sec
D11	0.03000000	sec
TD0	1	
======== CHANNEL f1 ========		
SFO1	100.6233329	MHz
NUC1	13C	
P1	10.00	usec
SI	32768	
SF	100.6127711	MHz
WDW	EM	
SSB	0	
LB	2.00	Hz
GB	0	
PC	1.00	

^{19}F NMR of **2gh** ($CDCl_3$, 376 MHz)

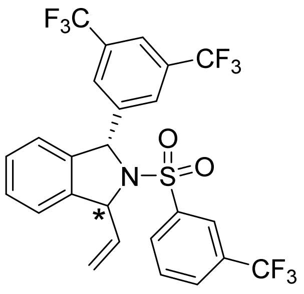

2gh, 1:1.6 dr

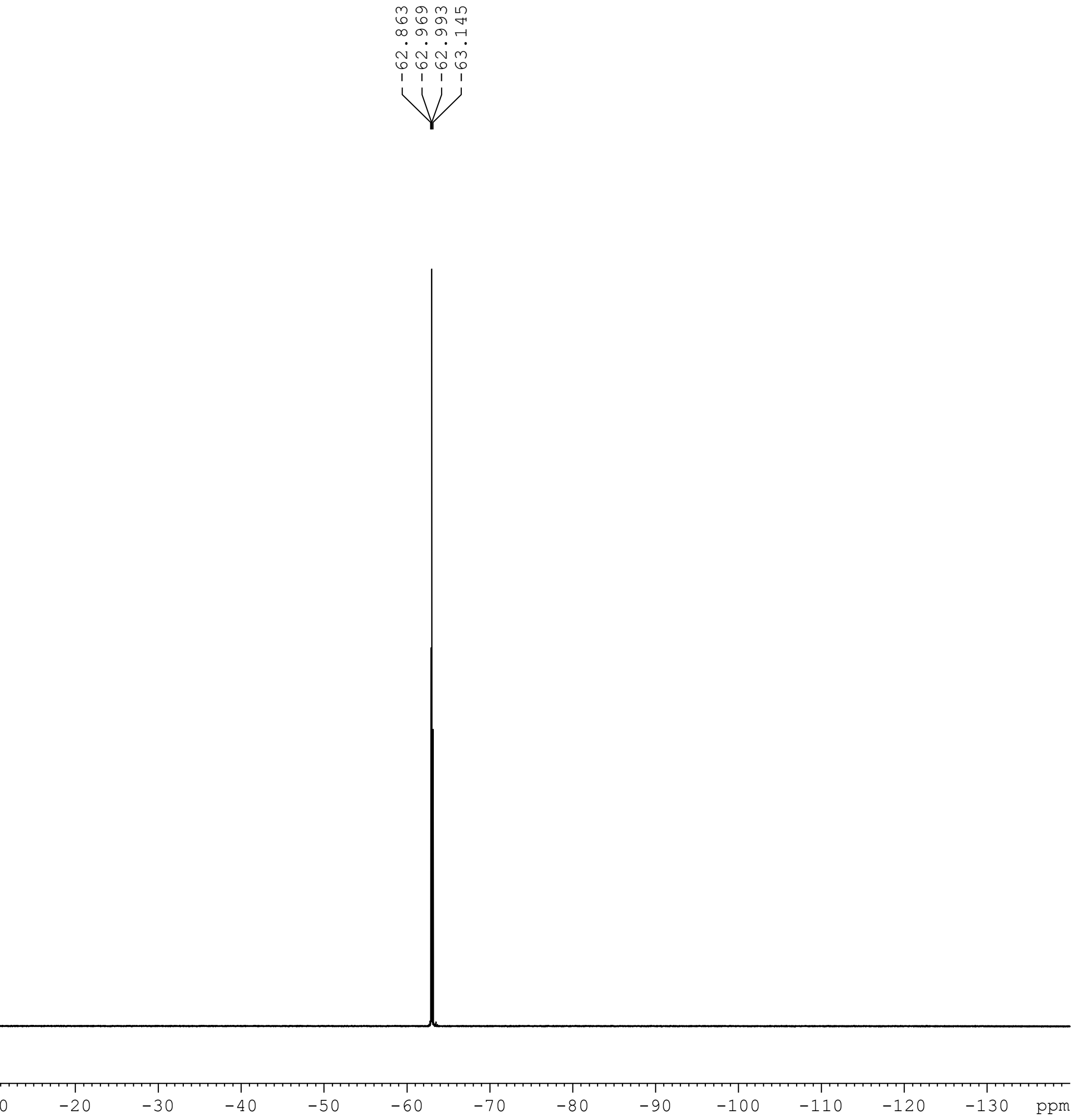


```
NAME              202407
EXPNO                459
PROCNO                 1
Date_           20240720
Time               15.51
INSTRUM            spect
PROBHD   5 mm PABBO BB/
PULPROG             zg30
TD                131072
SOLVENT            CDCl3
NS                    20
DS                     0
SWH           89285.711 Hz
FIDRES         0.681196 Hz
AQ            0.7340532 sec
RG                205.92
DW                 5.600 usec
DE                  6.50 usec
TE                 290.9 K
D1            1.00000000 sec
TD0                    1

======== CHANNEL f1 ========
SFO1         376.4757776 MHz
NUC1                 19F
P1                 15.00 usec
SI                 65536
SF           376.4983662 MHz
WDW                   EM
SSB                    0
LB                  0.30 Hz
GB                     0
PC                  1.00
```

^{1}H NMR of **2hh** ($CDCl_3$, 400 MHz)

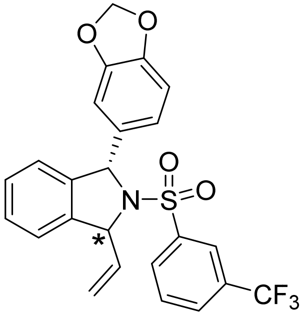

2hh, 1:1.5 dr

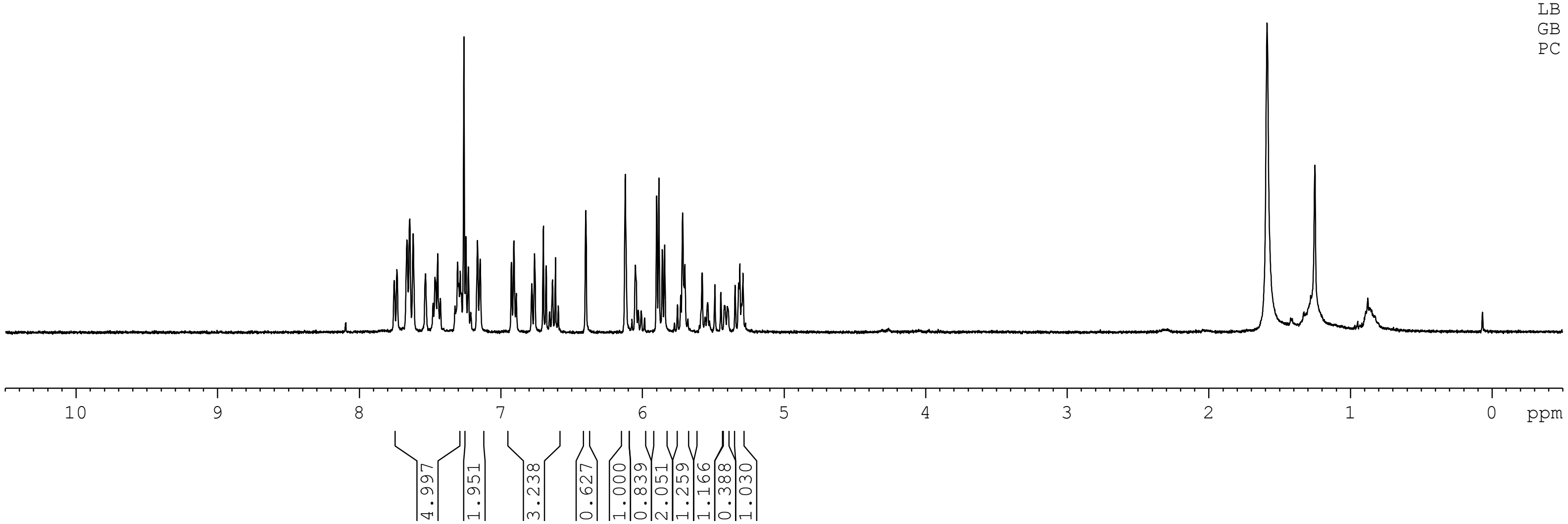

NAME	202408
EXPNO	456
PROCNO	1
Date_	20240828
Time	16.56
INSTRUM	spect
PROBHD	5 mm PABBO BB/
PULPROG	zg30
TD	32768
SOLVENT	CDCl3
NS	8
DS	0
SWH	8012.820 Hz
FIDRES	0.244532 Hz
AQ	2.0447731 sec
RG	205.92
DW	62.400 usec
DE	16.53 usec
TE	292.3 K
D1	2.00000000 sec
TD0	1

======== CHANNEL f1 ========

SFO1	400.1324008 MHz
NUC1	1H
P1	14.00 usec
SI	16384
SF	400.1300095 MHz
WDW	EM
SSB	0
LB	0.00 Hz
GB	0
PC	1.00

$^{13}C\{^{1}H\}$ NMR of **2hh** ($CDCl_3$, 101 MHz)

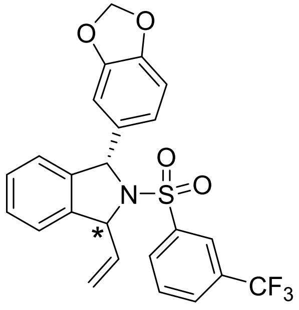

2hh, 1:1.5 dr

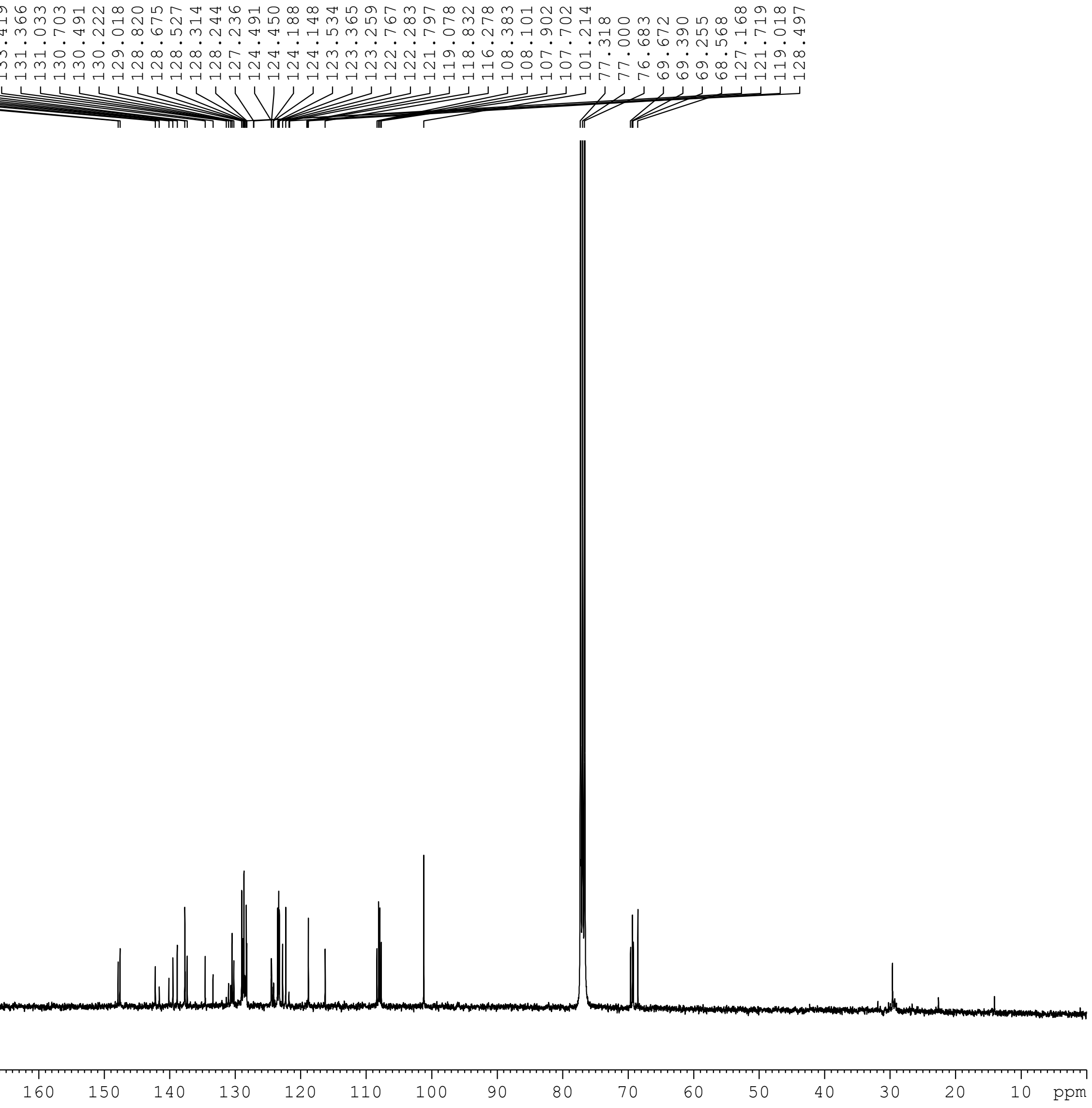

NAME	202408
EXPNO	253
PROCNO	1
Date_	20240814
Time	6.52
INSTRUM	spect
PROBHD	5 mm PABBO BB/
PULPROG	zgpg30
TD	32768
SOLVENT	CDCl3
NS	12618
DS	0
SWH	24038.461 Hz
FIDRES	0.733596 Hz
AQ	0.6816244 sec
RG	205.92
DW	20.800 usec
DE	6.50 usec
TE	292.7 K
D1	2.00000000 sec
D11	0.03000000 sec
TD0	1
======== CHANNEL f1 ========	
SFO1	100.6233329 MHz
NUC1	13C
P1	10.00 usec
SI	32768
SF	100.6127721 MHz
WDW	EM
SSB	0
LB	2.00 Hz
GB	0
PC	1.00

^{19}F NMR of **2hh** ($CDCl_3$, 376 MHz)

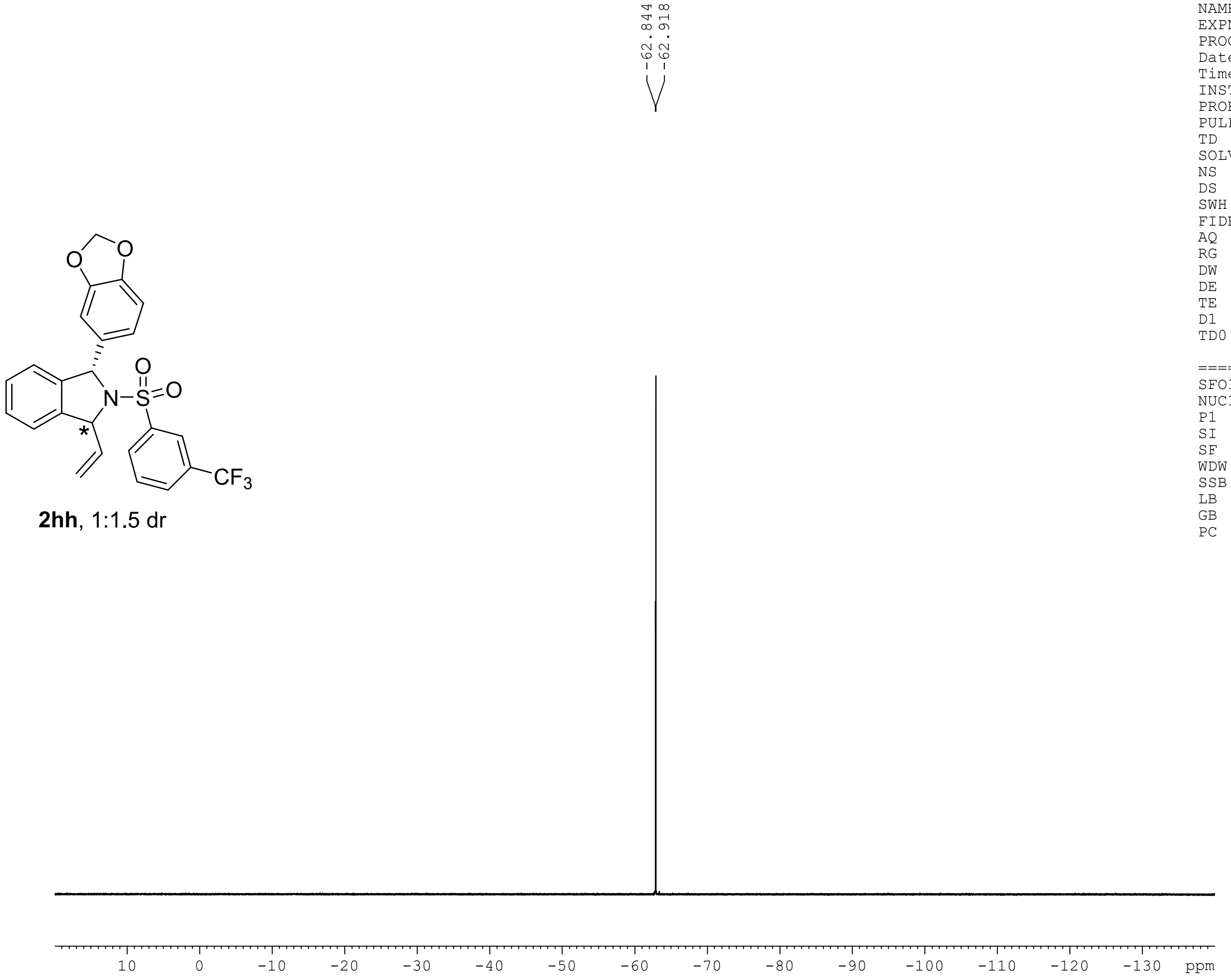

2hh, 1:1.5 dr

NAME	202408
EXPNO	252
PROCNO	1
Date_	20240813
Time	21.15
INSTRUM	spect
PROBHD	5 mm PABBO BB/
PULPROG	zg30
TD	131072
SOLVENT	CDCl3
NS	16
DS	0
SWH	89285.711 Hz
FIDRES	0.681196 Hz
AQ	0.7340532 sec
RG	205.92
DW	5.600 usec
DE	6.50 usec
TE	291.7 K
D1	1.00000000 sec
TD0	1

======== CHANNEL f1 ========

SFO1	376.4757776 MHz
NUC1	19F
P1	15.00 usec
SI	65536
SF	376.4983662 MHz
WDW	EM
SSB	0
LB	0.30 Hz
GB	0
PC	1.00

^{1}H NMR of **2ih** ($CDCl_3$, 400 MHz)

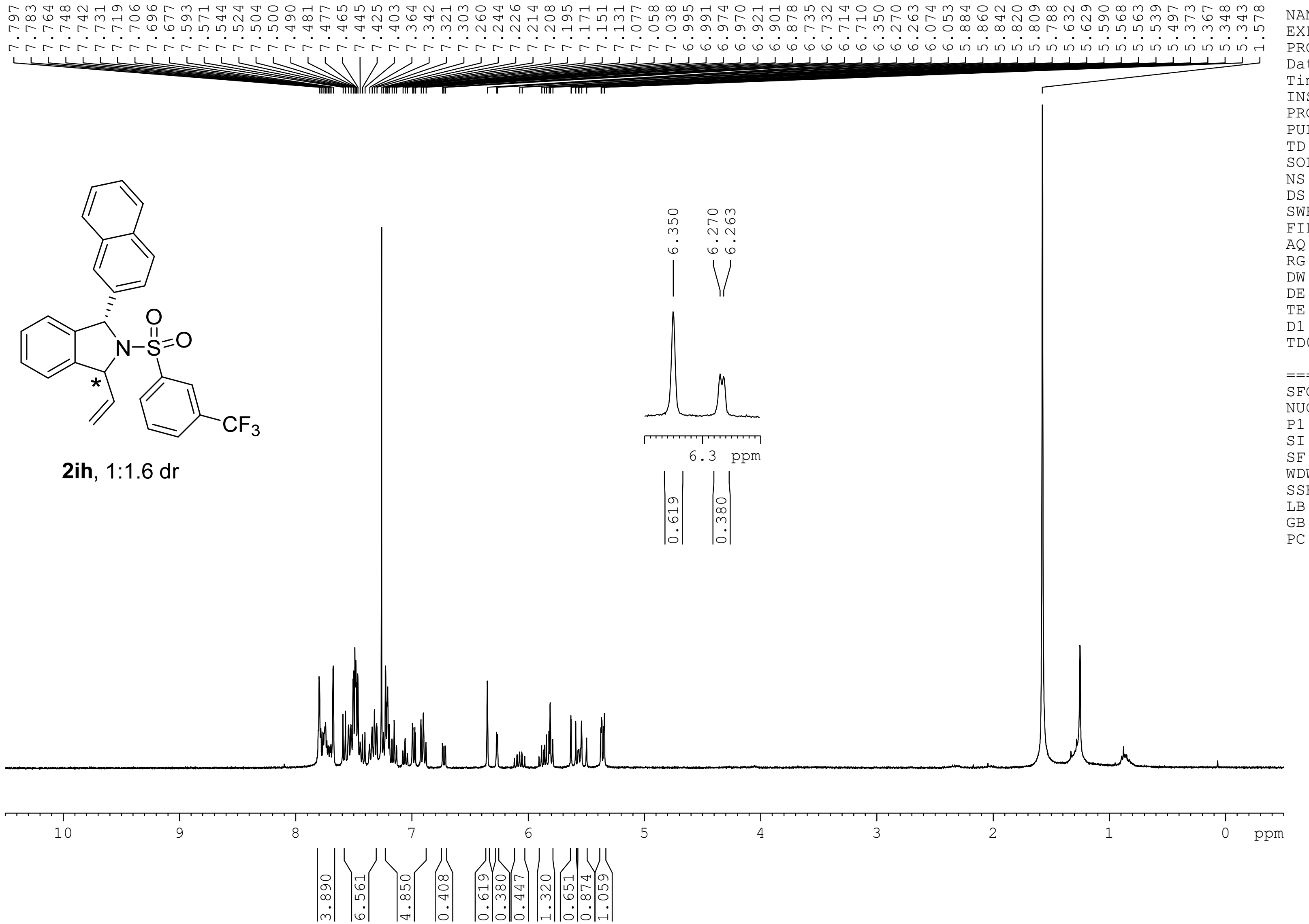

2ih, 1:1.6 dr

NAME	202408
EXPNO	259
PROCNO	1
Date_	20240814
Time	11.20
INSTRUM	spect
PROBHD	5 mm PABBO BB/
PULPROG	zg30
TD	32768
SOLVENT	CDCl3
NS	20
DS	0
SWH	8012.820 Hz
FIDRES	0.244532 Hz
AQ	2.0447731 sec
RG	205.92
DW	62.400 usec
DE	16.53 usec
TE	295.3 K
D1	2.00000000 sec
TD0	1
======== CHANNEL f1 ========	
SFO1	400.1324008 MHz
NUC1	1H
P1	14.00 usec
SI	16384
SF	400.1300096 MHz
WDW	EM
SSB	0
LB	0.00 Hz
GB	0
PC	1.00

$^{13}C\{^{1}H\}$ NMR of **2ih** ($CDCl_3$, 101 MHz)

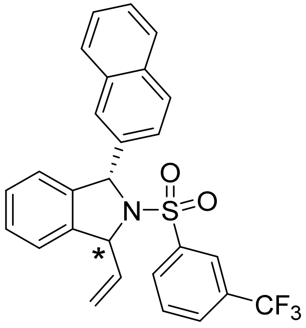

2ih, 1:1.6 dr

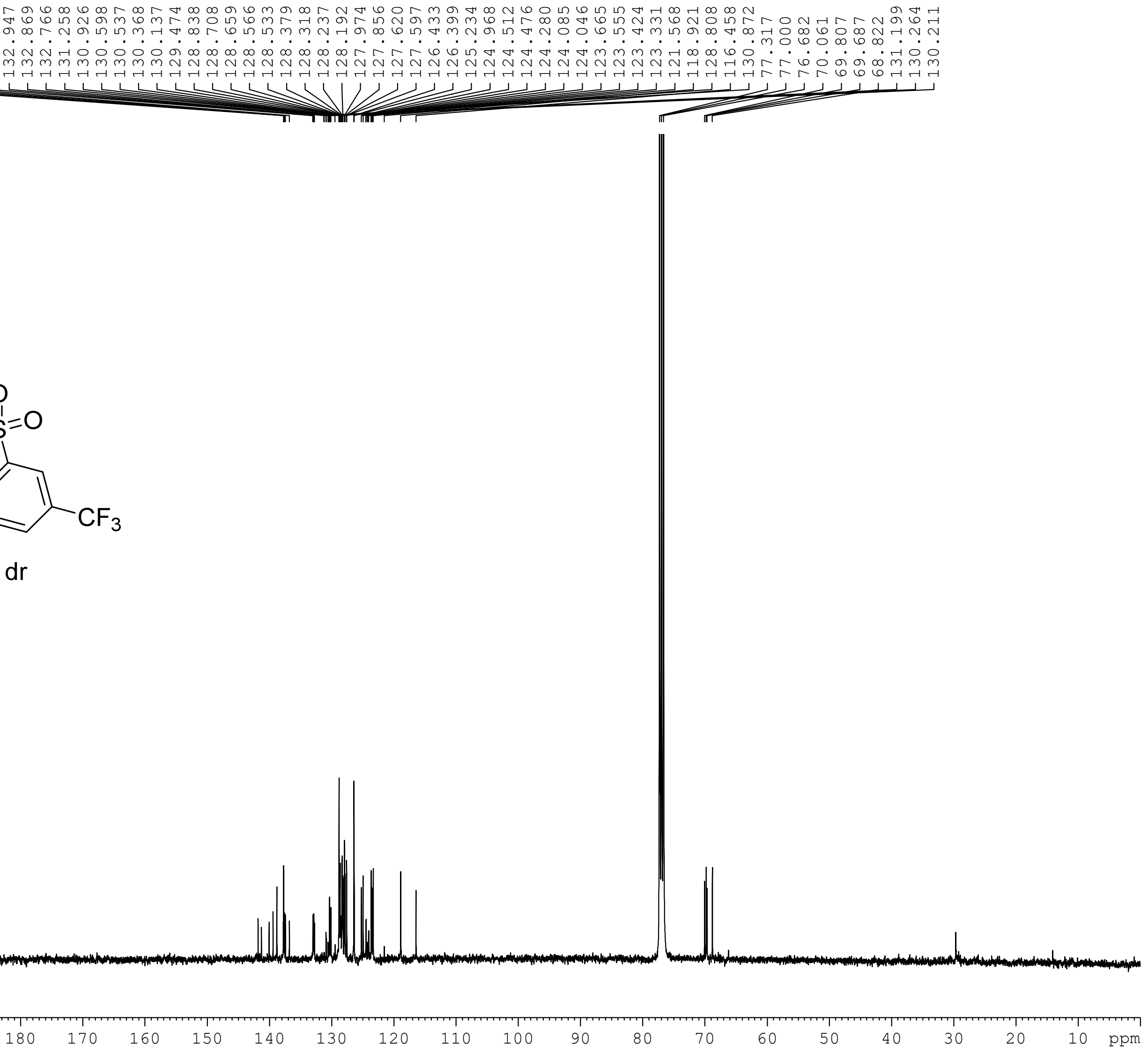

NAME	202408	
EXPNO	34	
PROCNO	1	
Date_	20240802	
Time	8.20	
INSTRUM	spect	
PROBHD	5 mm PABBO BB/	
PULPROG	zgpg30	
TD	32768	
SOLVENT	CDCl3	
NS	9803	
DS	0	
SWH	24038.461	Hz
FIDRES	0.733596	Hz
AQ	0.6816244	sec
RG	205.92	
DW	20.800	usec
DE	6.50	usec
TE	293.1	K
D1	2.00000000	sec
D11	0.03000000	sec
TD0	1	

======== CHANNEL f1 ========

SFO1	100.6233329	MHz
NUC1	13C	
P1	10.00	usec
SI	32768	
SF	100.6127718	MHz
WDW	EM	
SSB	0	
LB	2.00	Hz
GB	0	
PC	1.00	

^{19}F NMR of **2ih** ($CDCl_3$, 376 MHz)

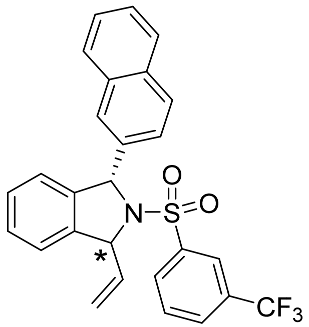

2ih, 1:1.6 dr

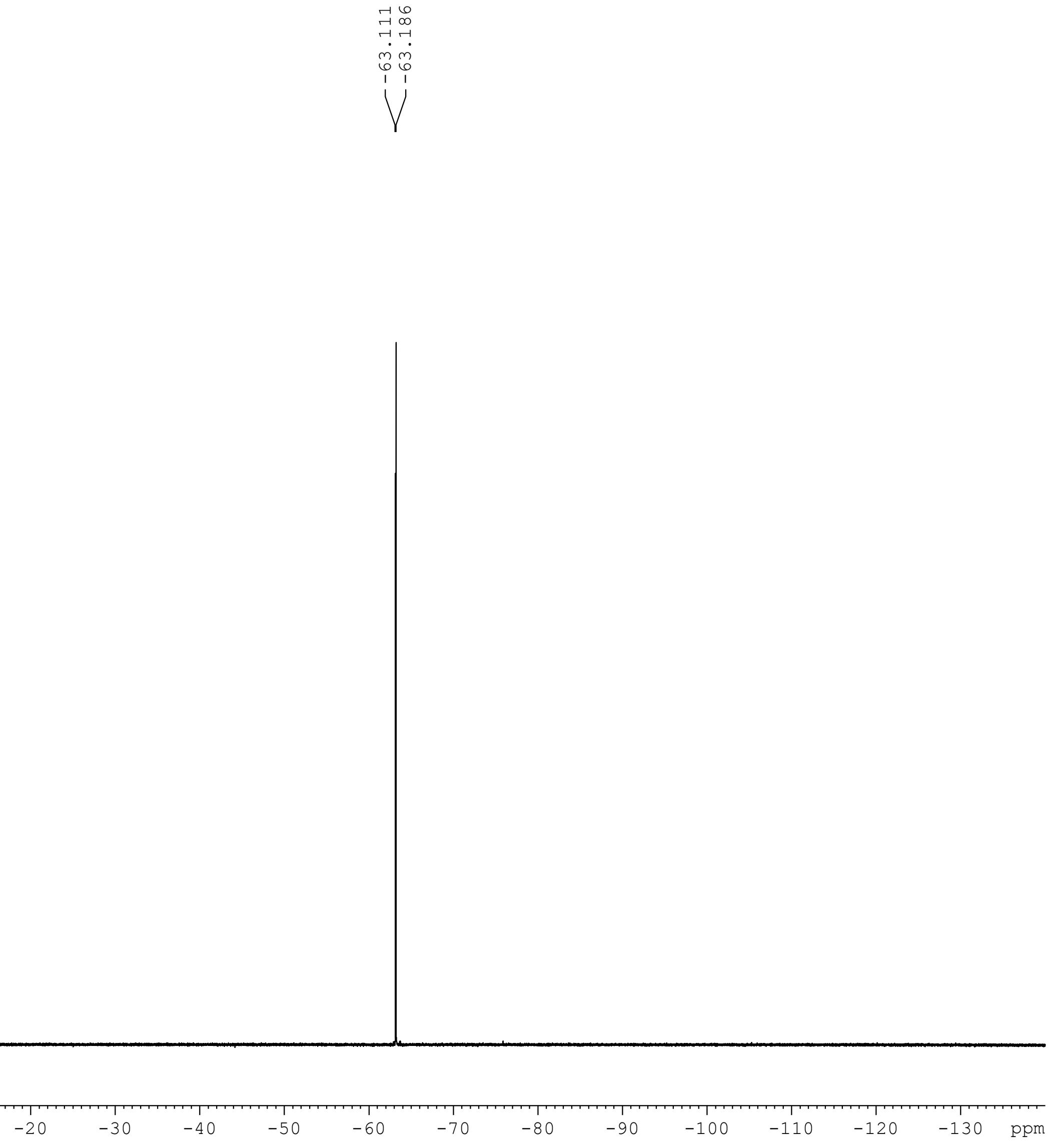

NAME	202408
EXPNO	47
PROCNO	1
Date_	20240802
Time	16.23
INSTRUM	spect
PROBHD	5 mm PABBO BB/
PULPROG	zg30
TD	131072
SOLVENT	CDCl3
NS	16
DS	0
SWH	89285.711 Hz
FIDRES	0.681196 Hz
AQ	0.7340532 sec
RG	205.92
DW	5.600 usec
DE	6.50 usec
TE	291.1 K
D1	1.00000000 sec
TD0	1

======== CHANNEL f1 ========

SFO1	376.4757776 MHz
NUC1	19F
P1	15.00 usec
SI	65536
SF	376.4983662 MHz
WDW	EM
SSB	0
LB	0.30 Hz
GB	0
PC	1.00

^{1}H NMR of (1*S*,3*R*)-**2ih** ($CDCl_3$, 400 MHz)

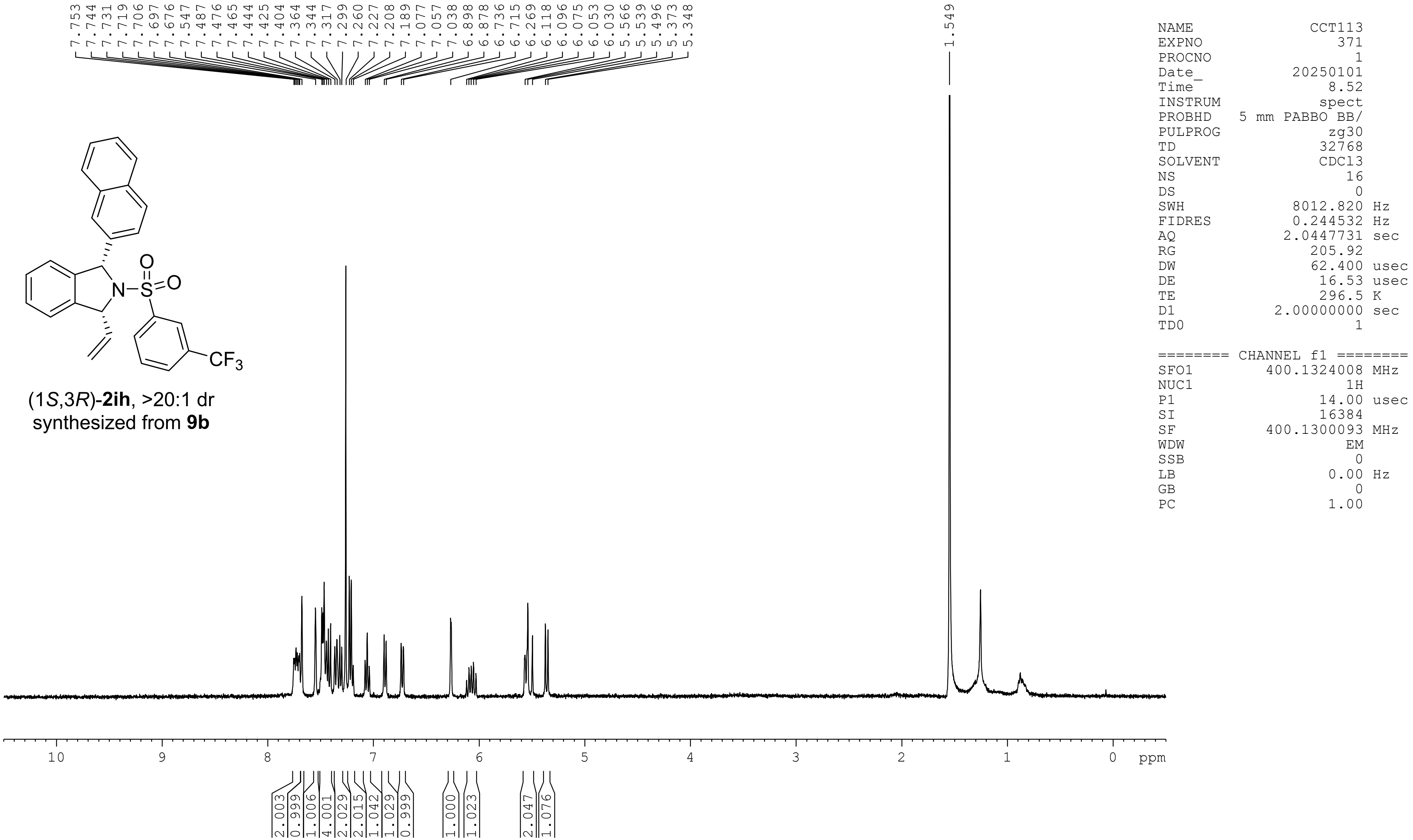

Zoomed-in 2D NMR of (1*S*,3*R*)-**2ih** ($CDCl_3$)

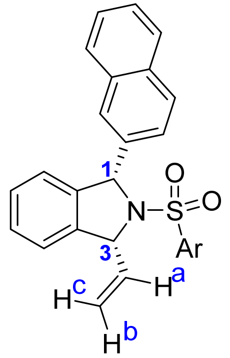

(1*S*,3*R*)-**2ih**, >20:1 dr
synthesized from **9b**
Ar = 3-$CF_3C_6H_4$

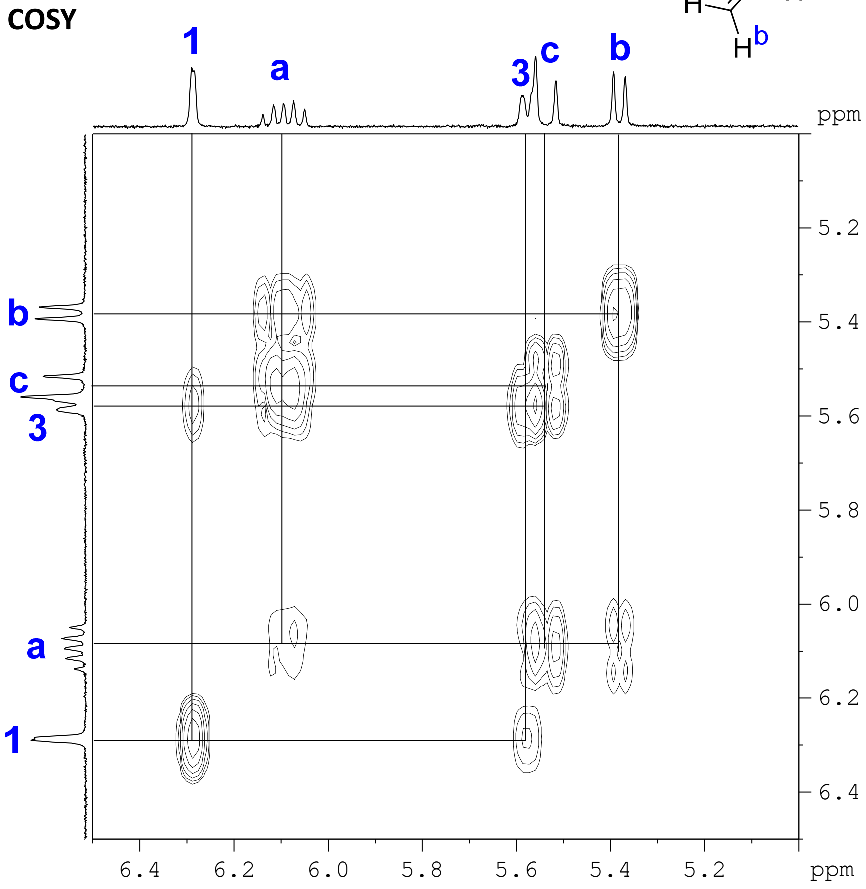

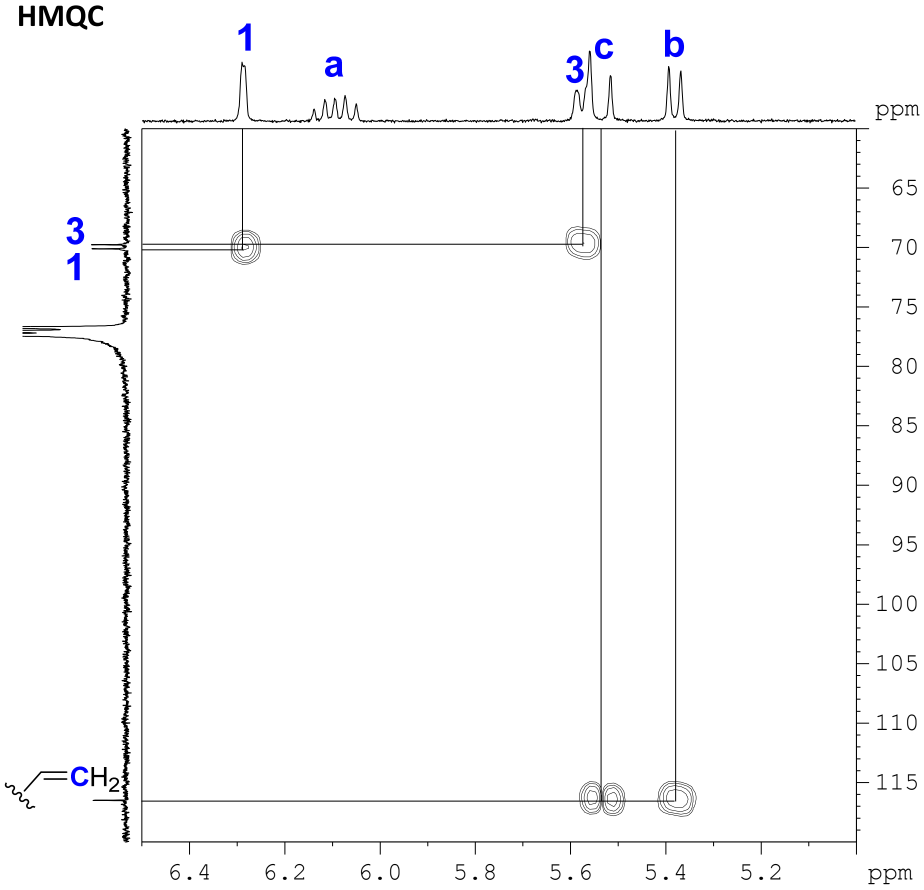

^{1}H NMR of (1*R*,3*S*)-**2ih** ($CDCl_3$, 400 MHz)

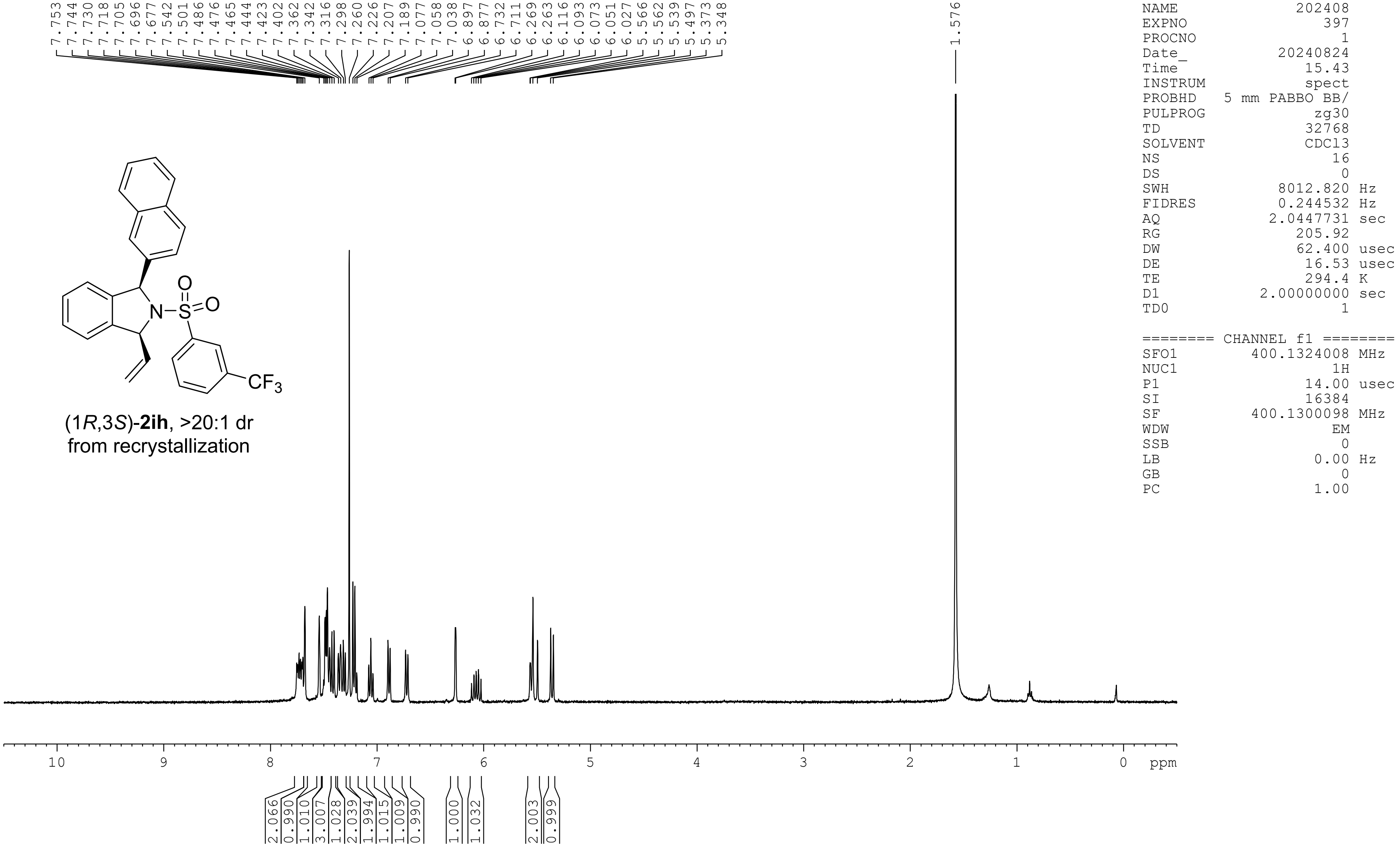

$^{13}C\{^1H\}$ NMR of (1*R*,3*S*)-**2ih** ($CDCl_3$, 176 MHz)

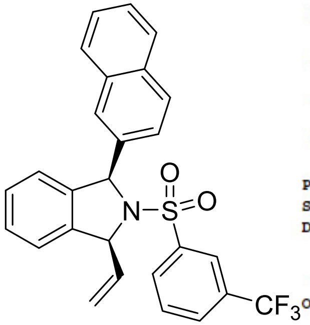

(1*R*,3*S*)-**2ih**, >20:1 dr
from recrystallization

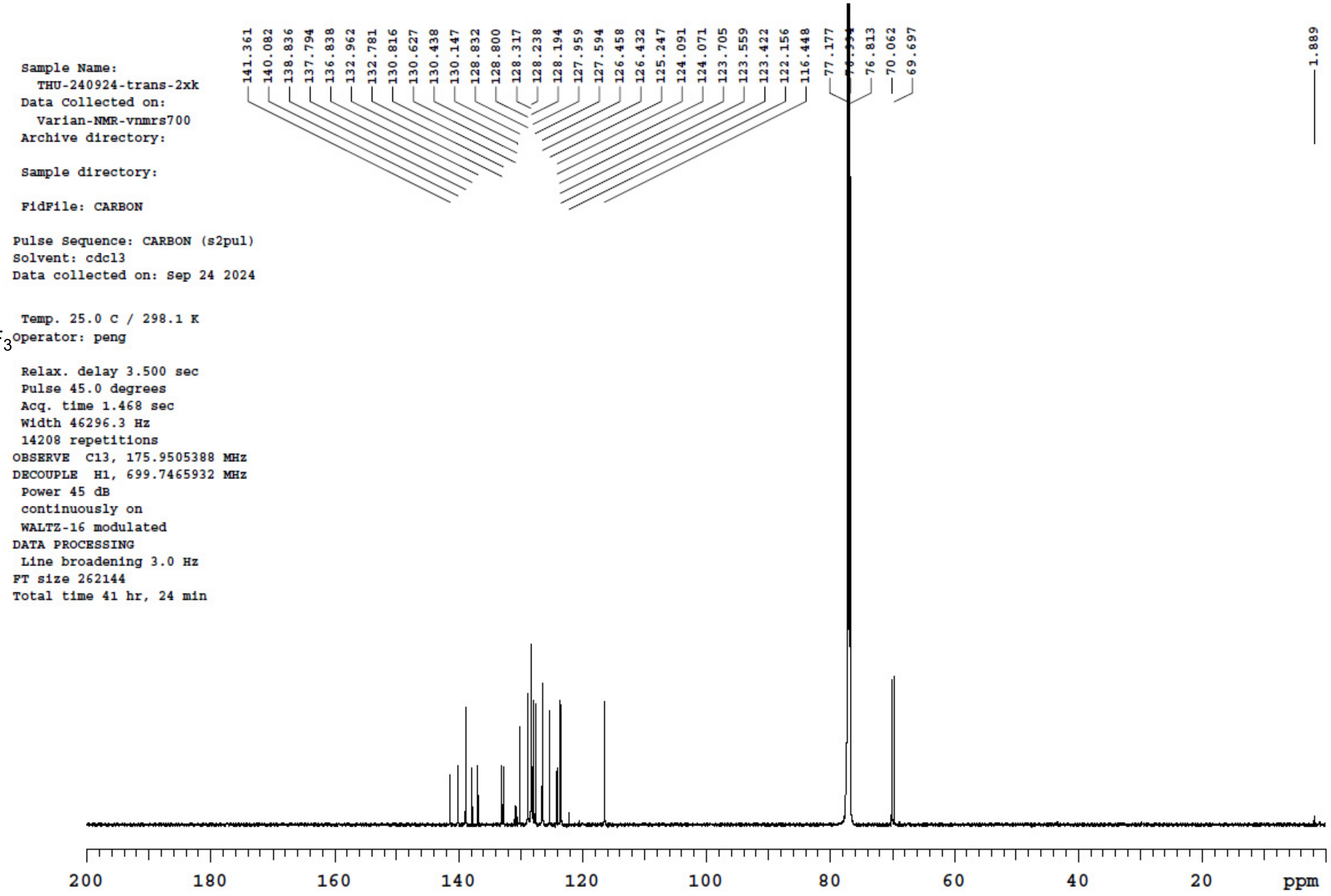

^{19}F NMR of (1*R*,3*S*)-**2ih** ($CDCl_3$, 376 MHz)

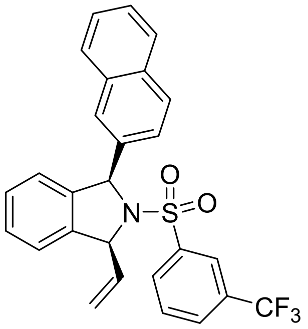

(1*R*,3*S*)-**2ih**, >20:1 dr
from recrystallization

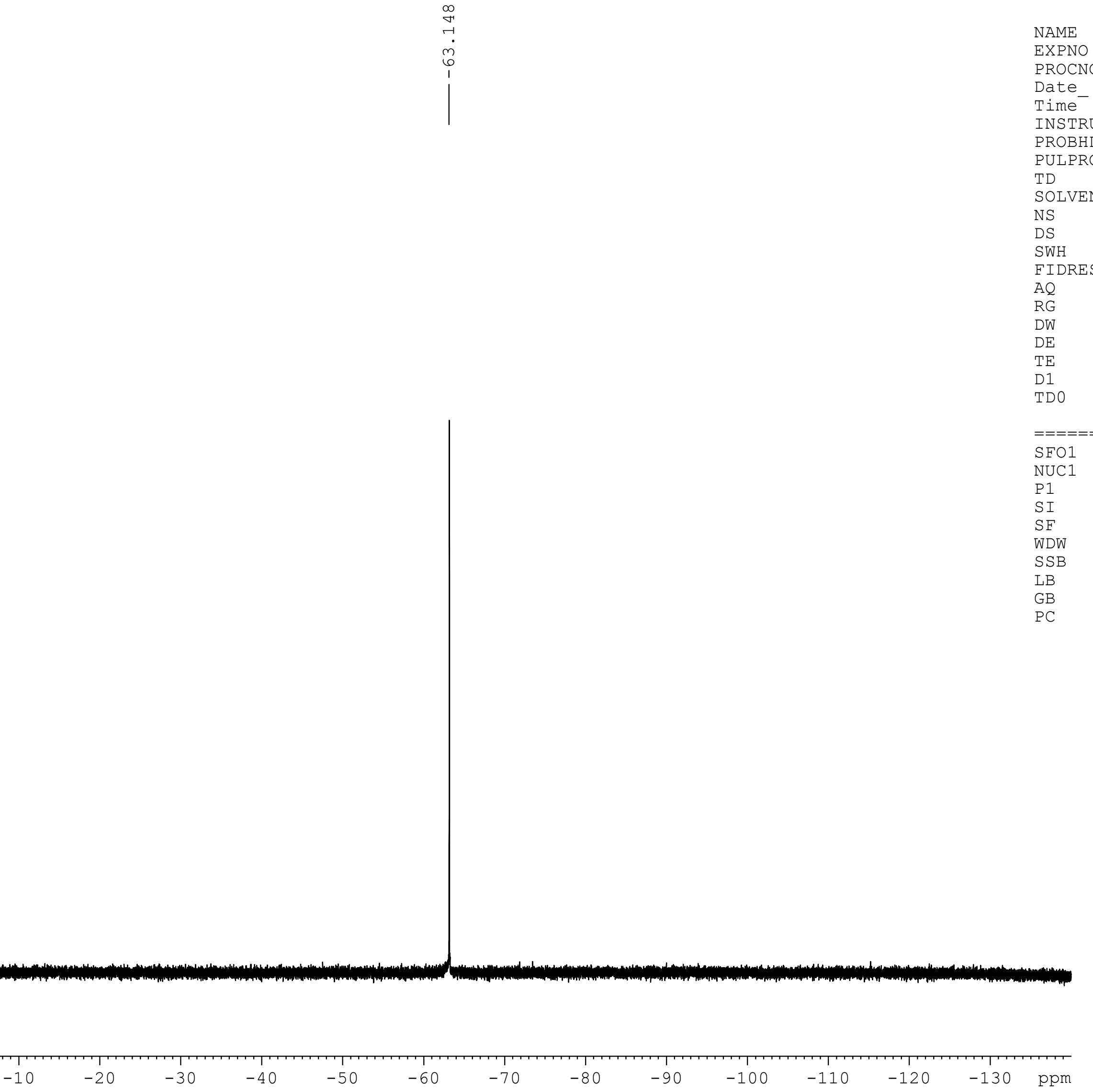

NAME	202408	
EXPNO	442	
PROCNO	1	
Date_	20240828	
Time	4.59	
INSTRUM	spect	
PROBHD	5 mm PABBO BB/	
PULPROG	zg30	
TD	131072	
SOLVENT	CDCl3	
NS	20	
DS	0	
SWH	89285.711	Hz
FIDRES	0.681196	Hz
AQ	0.7340532	sec
RG	205.92	
DW	5.600	usec
DE	6.50	usec
TE	300.2	K
D1	1.00000000	sec
TD0	1	

======== CHANNEL f1 ========

SFO1	376.4757776	MHz
NUC1	19F	
P1	15.00	usec
SI	65536	
SF	376.4983662	MHz
WDW	EM	
SSB	0	
LB	0.30	Hz
GB	0	
PC	1.00	

^{1}H NMR of (1*R*,3*R*)-**2ih** ($CDCl_3$, 400 MHz)

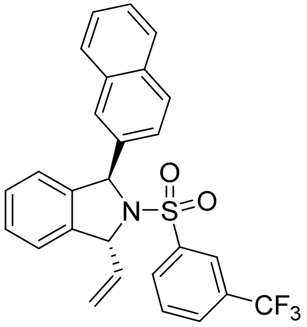

(1*R*,3*R*)-**2ih**, 1:>20 dr
separated from diastereomers in **2ih**

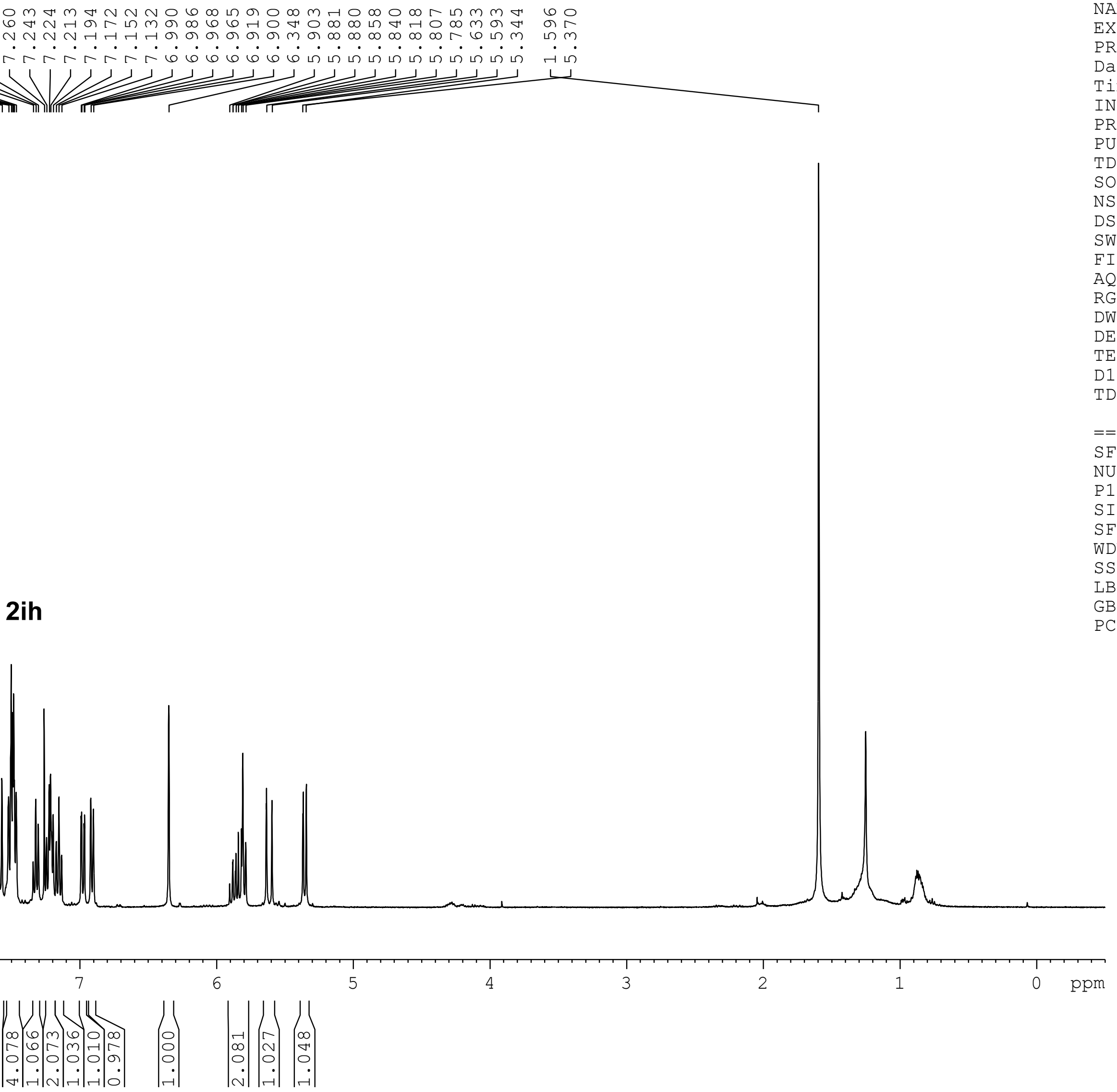

NAME	202409
EXPNO	120
PROCNO	1
Date_	20240925
Time	13.09
INSTRUM	spect
PROBHD	5 mm PABBO BB/
PULPROG	zg30
TD	32768
SOLVENT	CDCl3
NS	30
DS	0
SWH	8012.820 Hz
FIDRES	0.244532 Hz
AQ	2.0447731 sec
RG	205.92
DW	62.400 usec
DE	16.53 usec
TE	291.2 K
D1	2.00000000 sec
TD0	1

======== CHANNEL f1 ========

SFO1	400.1324008 MHz
NUC1	1H
P1	14.00 usec
SI	16384
SF	400.1300104 MHz
WDW	EM
SSB	0
LB	0.00 Hz
GB	0
PC	1.00

$^{13}C\{^{1}H\}$ NMR of (1*R*,3*R*)-**2ih** ($CDCl_3$, 101 MHz)

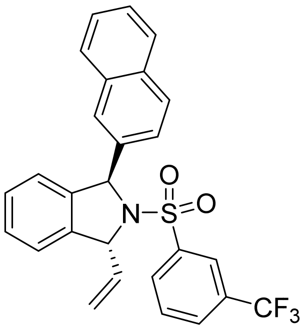

(1*R*,3*R*)-**2ih**, 1:>20 dr
separated from diastereomers in **2ih**

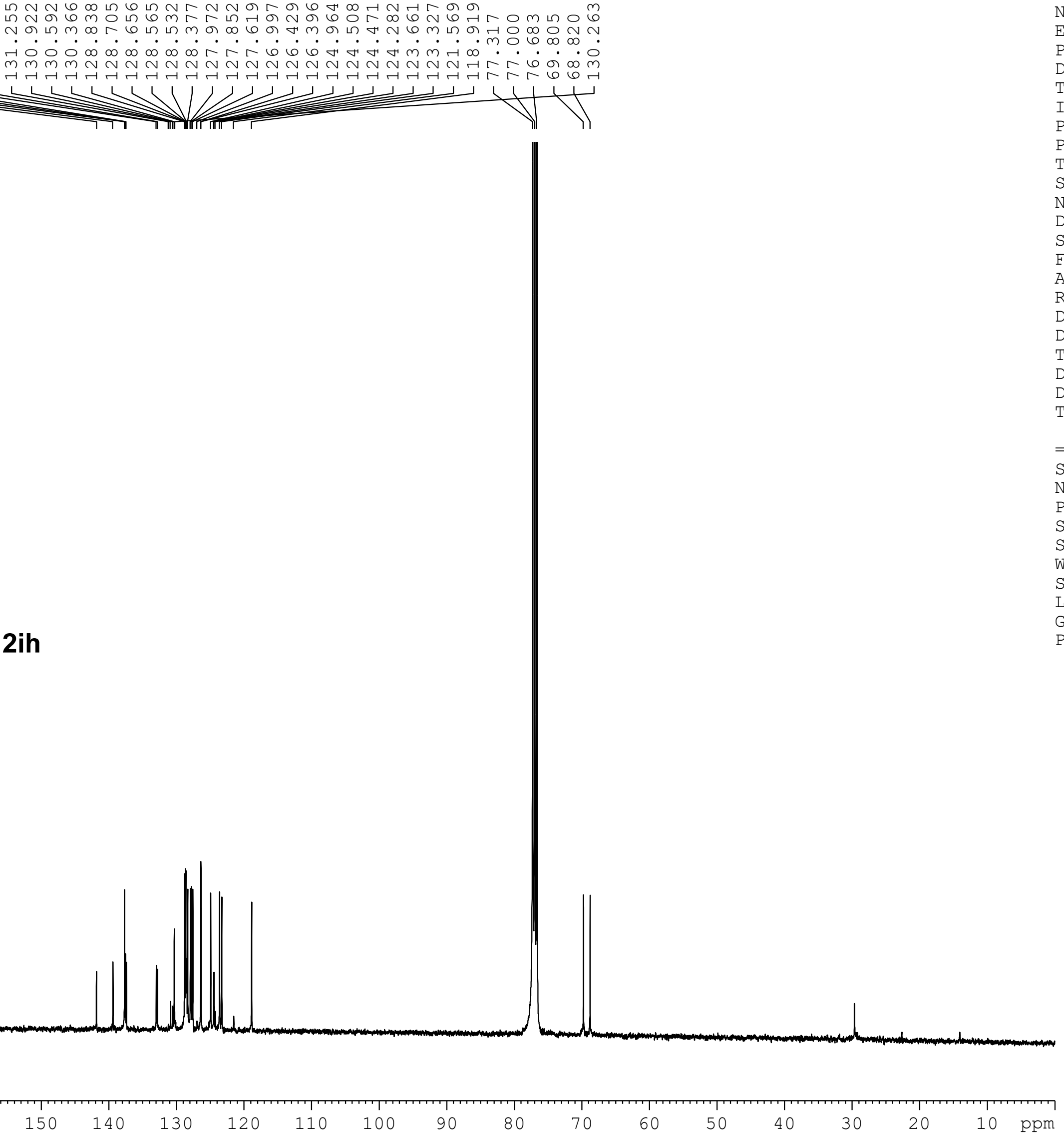

NAME	202409	
EXPNO	122	
PROCNO	1	
Date_	20240925	
Time	20.52	
INSTRUM	spect	
PROBHD	5 mm PABBO BB/	
PULPROG	zgpg30	
TD	32768	
SOLVENT	CDCl3	
NS	16000	
DS	0	
SWH	24038.461	Hz
FIDRES	0.733596	Hz
AQ	0.6816244	sec
RG	205.92	
DW	20.800	usec
DE	6.50	usec
TE	291.0	K
D1	2.00000000	sec
D11	0.03000000	sec
TD0	1	

======== CHANNEL f1 ========

SFO1	100.6233329	MHz
NUC1	13C	
P1	10.00	usec
SI	32768	
SF	100.6127724	MHz
WDW	EM	
SSB	0	
LB	2.00	Hz
GB	0	
PC	1.00	

^{19}F NMR of (1*R*,3*R*)-**2ih** ($CDCl_3$, 376 MHz)

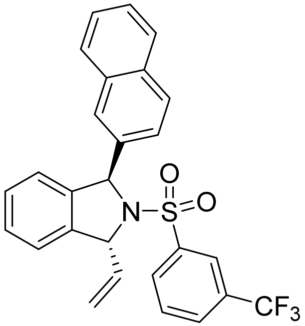

(1*R*,3*R*)-**2ih**, 1:>20 dr
separated from diastereomers in **2ih**

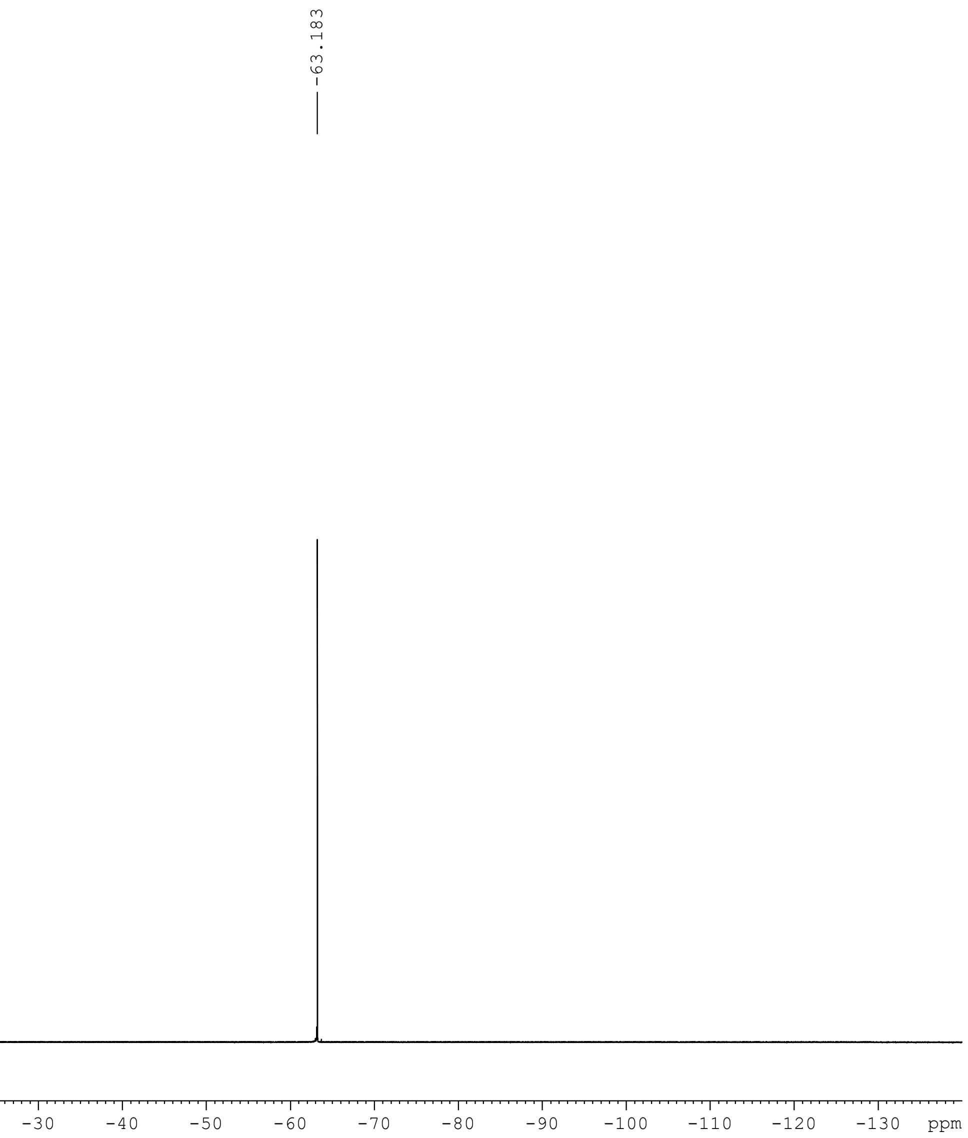

NAME	202409	
EXPNO	121	
PROCNO	1	
Date_	20240925	
Time	13.17	
INSTRUM	spect	
PROBHD	5 mm PABBO BB/	
PULPROG	zg30	
TD	131072	
SOLVENT	CDCl3	
NS	20	
DS	0	
SWH	89285.711	Hz
FIDRES	0.681196	Hz
AQ	0.7340532	sec
RG	205.92	
DW	5.600	usec
DE	6.50	usec
TE	291.4	K
D1	1.00000000	sec
TD0	1	

======== CHANNEL f1 ========

SFO1	376.4757776	MHz
NUC1	19F	
P1	15.00	usec
SI	65536	
SF	376.4983662	MHz
WDW	EM	
SSB	0	
LB	0.30	Hz
GB	0	
PC	1.00	

Zoomed-in 2D NMR of (1*R*,3*R*)-**2ih** ($CDCl_3$)

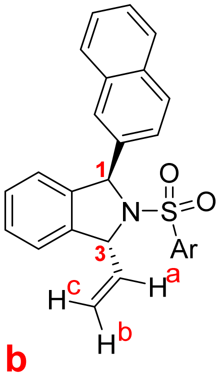

(1*R*,3*R*)-**2ih**, 1:>20 dr
separated from diastereomers in **2ih**
Ar = 3-$CF_3C_6H_4$

COSY

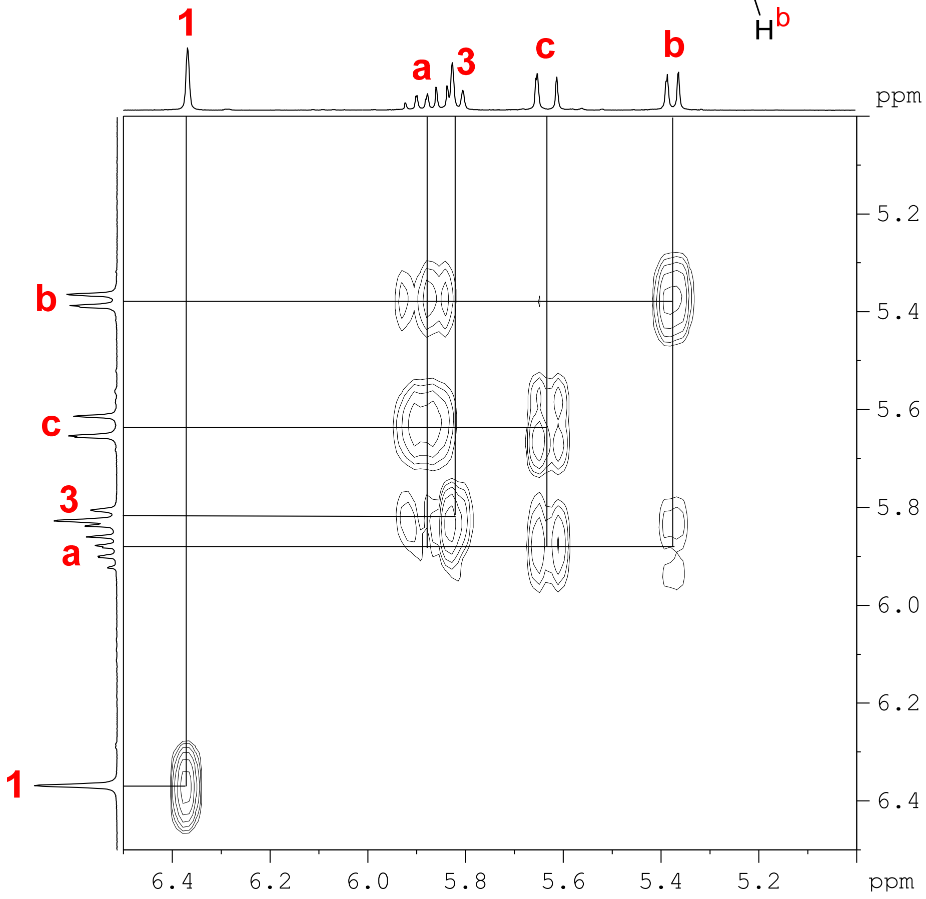

HMQC

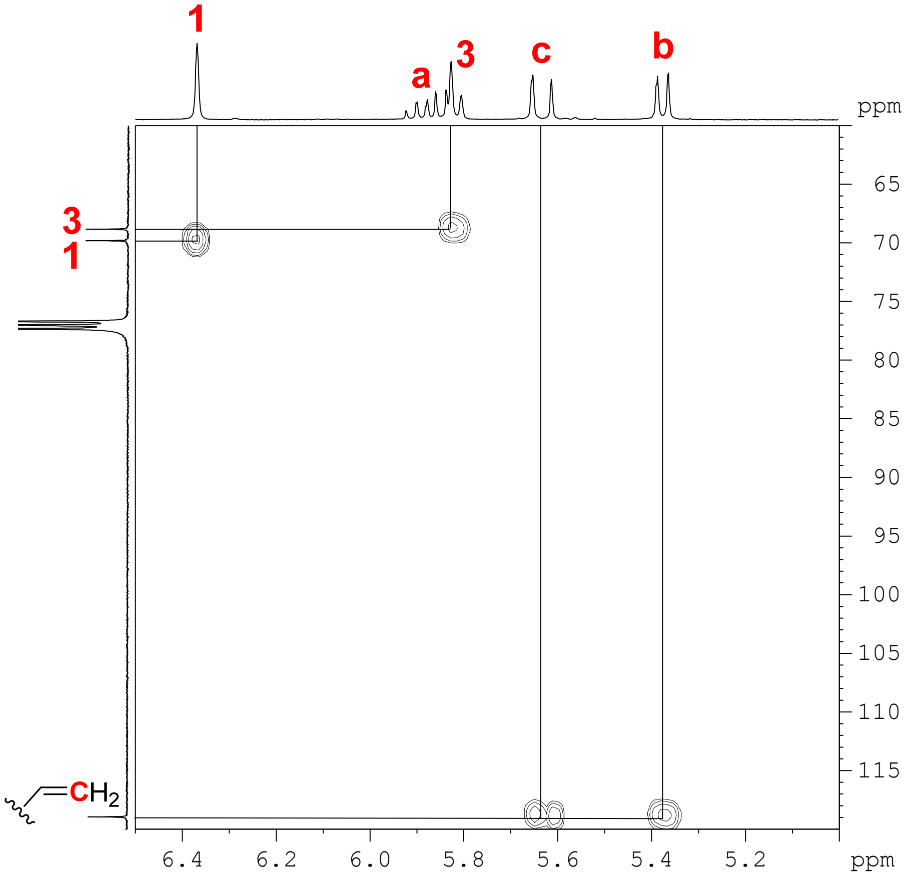

^{1}H NMR of **2jh** ($CDCl_3$, 400 MHz)

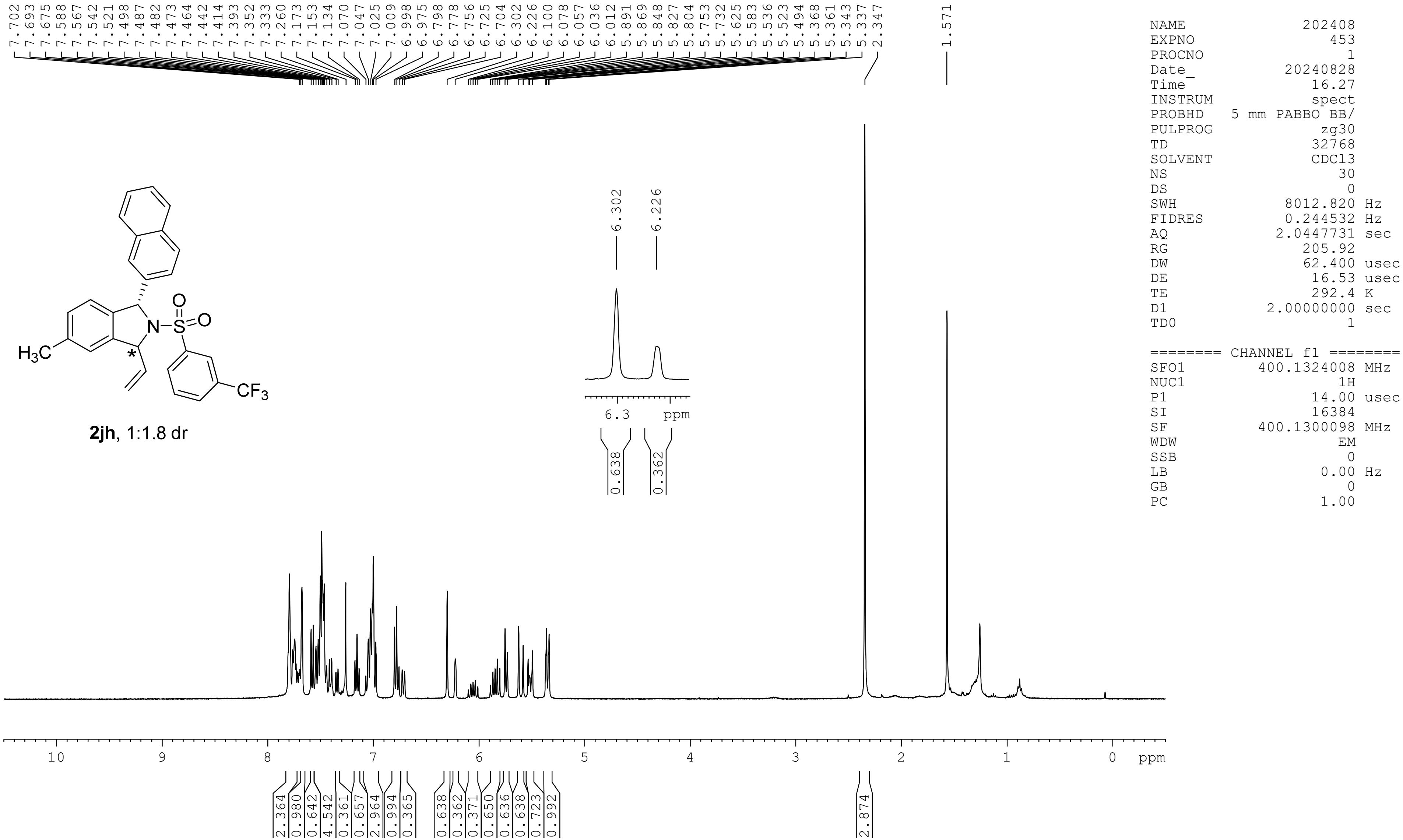

$^{13}C\{^1H\}$ NMR of **2jh** ($CDCl_3$, 101 MHz)

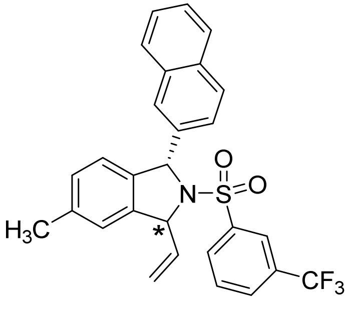

2jh, 1:1.8 dr

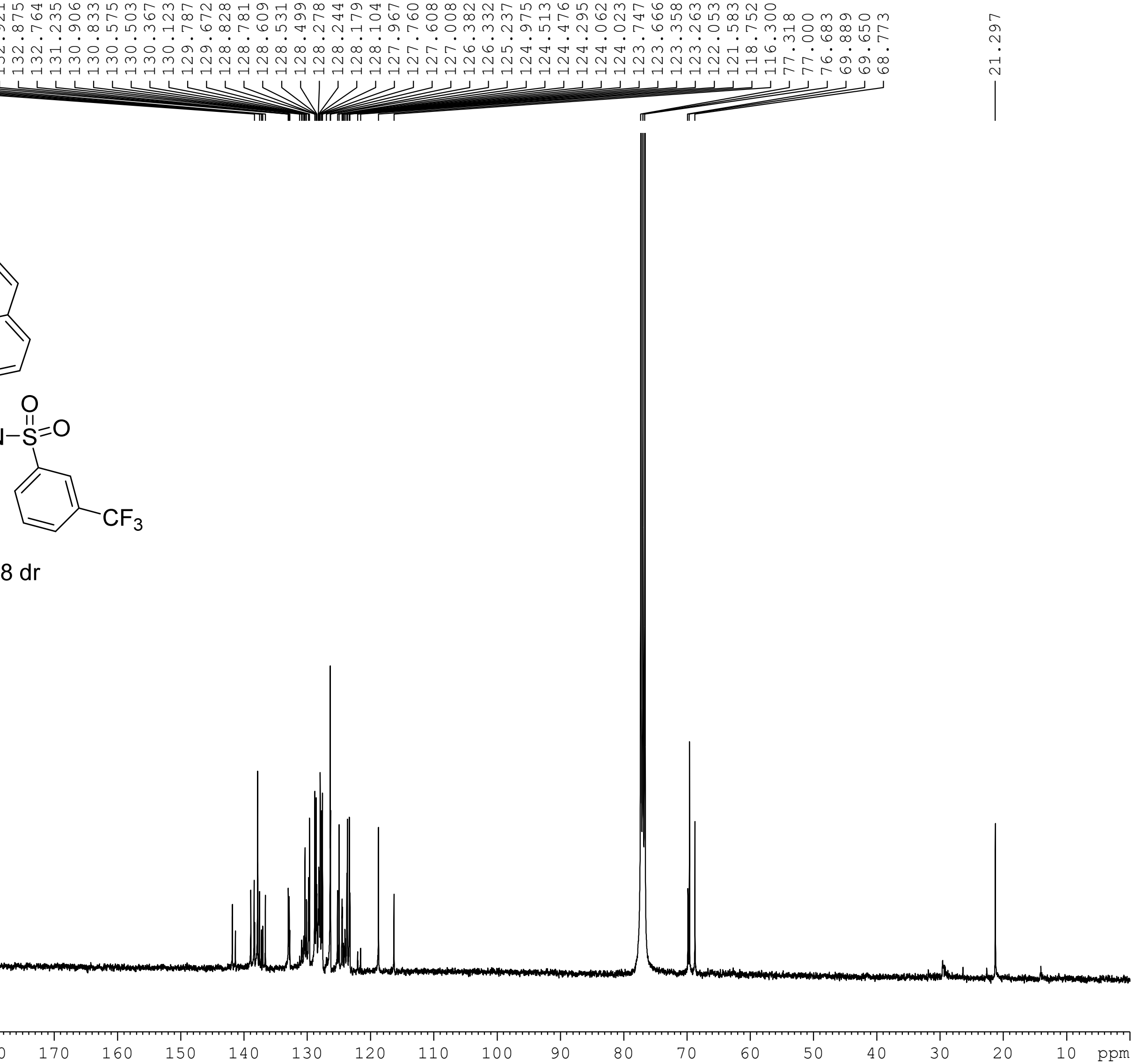

NAME	202408	
EXPNO	480	
PROCNO	1	
Date_	20240830	
Time	22.54	
INSTRUM	spect	
PROBHD	5 mm PABBO BB/	
PULPROG	zgpg30	
TD	32768	
SOLVENT	CDCl3	
NS	20000	
DS	0	
SWH	24038.461	Hz
FIDRES	0.733596	Hz
AQ	0.6816244	sec
RG	205.92	
DW	20.800	usec
DE	6.50	usec
TE	290.9	K
D1	2.00000000	sec
D11	0.03000000	sec
TD0	1	
======== CHANNEL f1 ========		
SFO1	100.6233329	MHz
NUC1	13C	
P1	10.00	usec
SI	32768	
SF	100.6127728	MHz
WDW	EM	
SSB	0	
LB	2.00	Hz
GB	0	
PC	1.00	

^{19}F NMR of **2jh** ($CDCl_3$, 376 MHz)

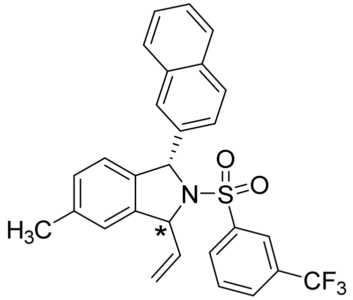

2jh, 1:1.8 dr

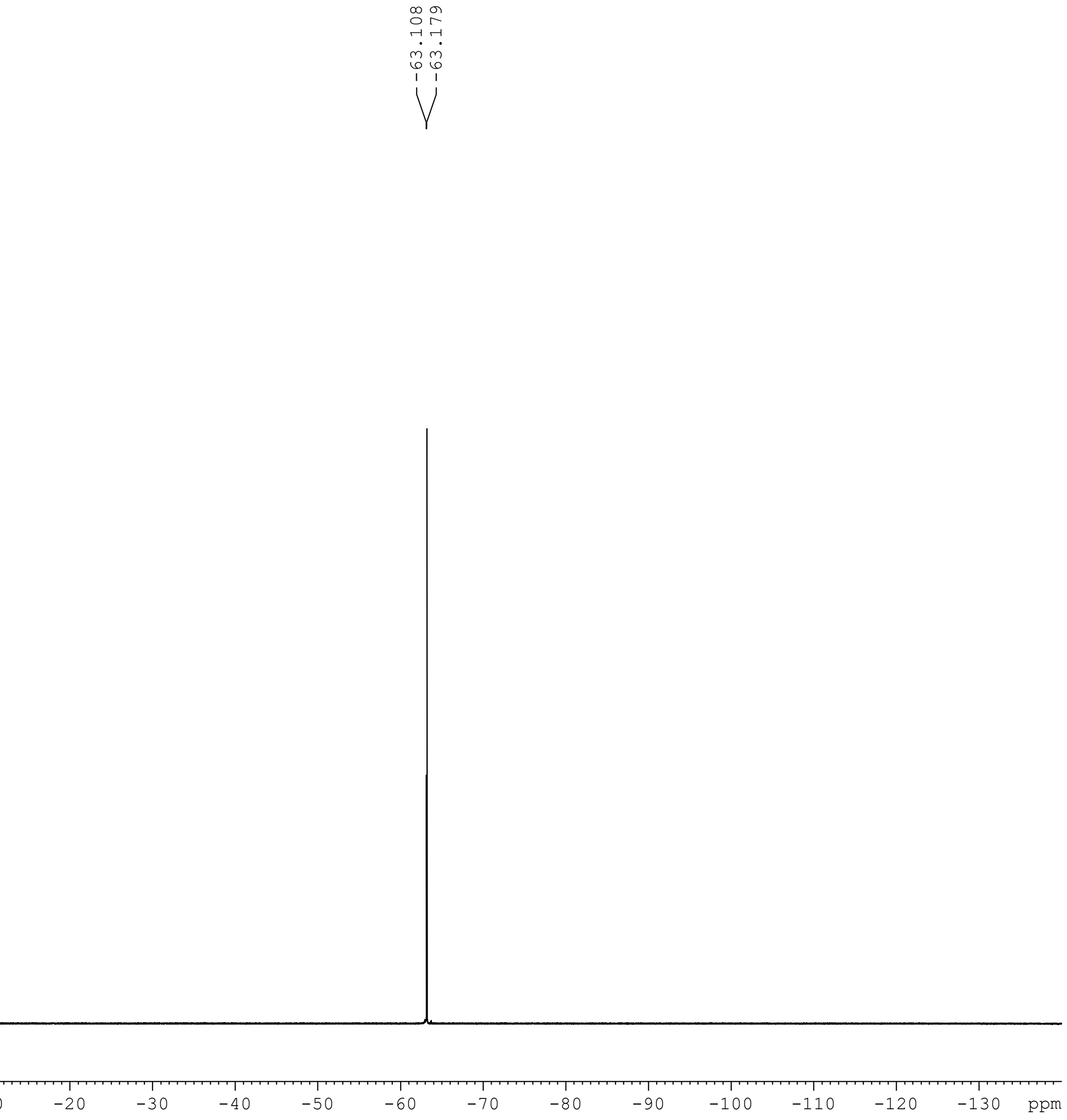

NAME	202408
EXPNO	454
PROCNO	1
Date_	20240828
Time	16.29
INSTRUM	spect
PROBHD	5 mm PABBO BB/
PULPROG	zgig30
TD	131072
SOLVENT	CDCl3
NS	16
DS	0
SWH	89285.711 Hz
FIDRES	0.681196 Hz
AQ	0.7340532 sec
RG	205.92
DW	5.600 usec
DE	6.50 usec
TE	292.6 K
D1	1.00000000 sec
D11	0.03000000 sec
TD0	1

======== CHANNEL f1 ========

SFO1	376.4757776 MHz
NUC1	19F
P1	15.00 usec
SI	65536
SF	376.4983662 MHz
WDW	EM
SSB	0
LB	0.30 Hz
GB	0
PC	1.00

^1H NMR of **2kh** ($CDCl_3$, 400 MHz)

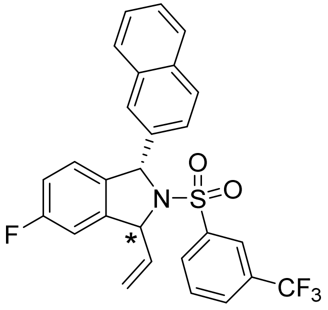

2kh, 1:1.7 dr

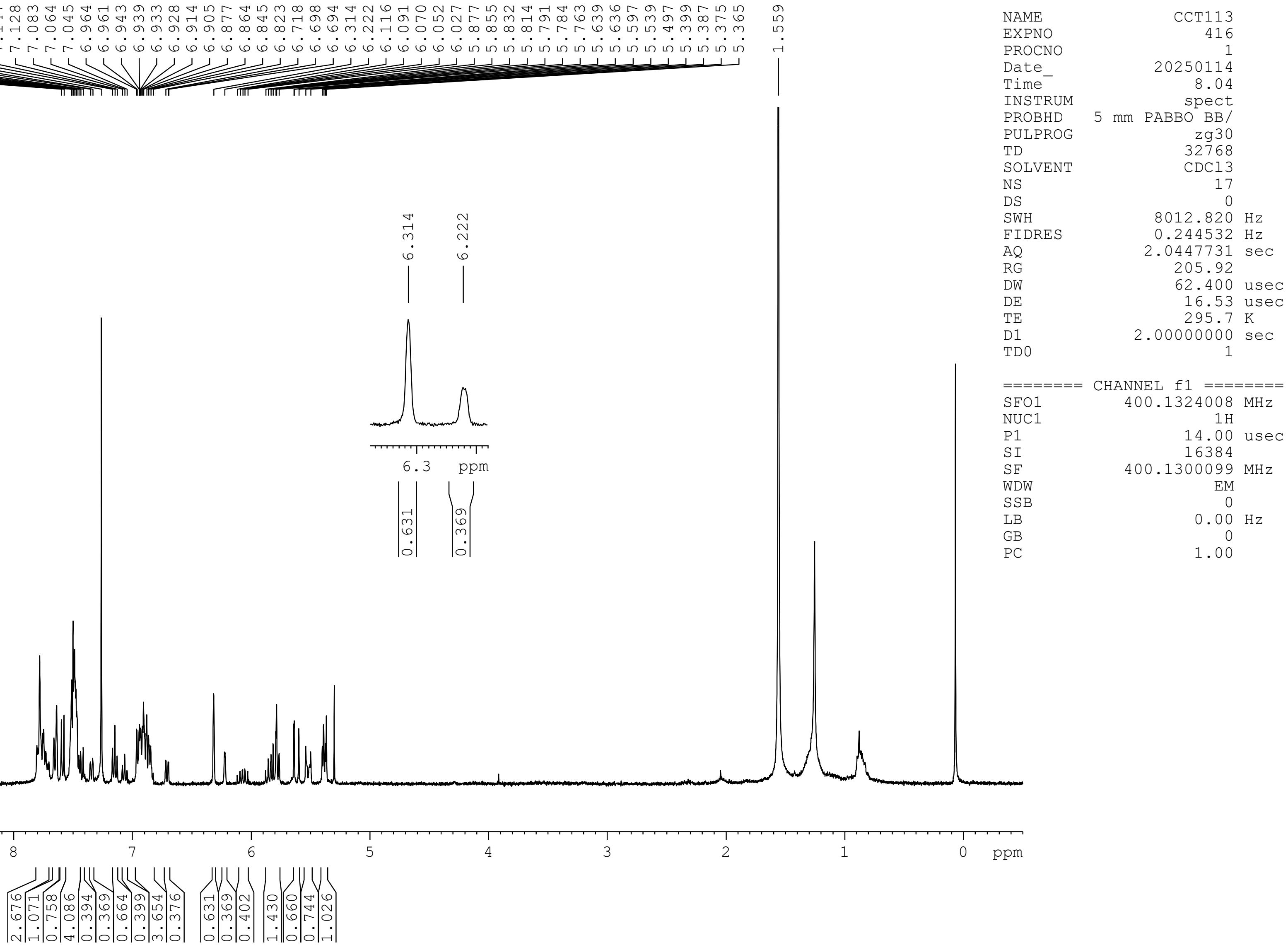

$^{13}C\{^1H\}$ NMR of **2kh** ($CDCl_3$, 101 MHz)

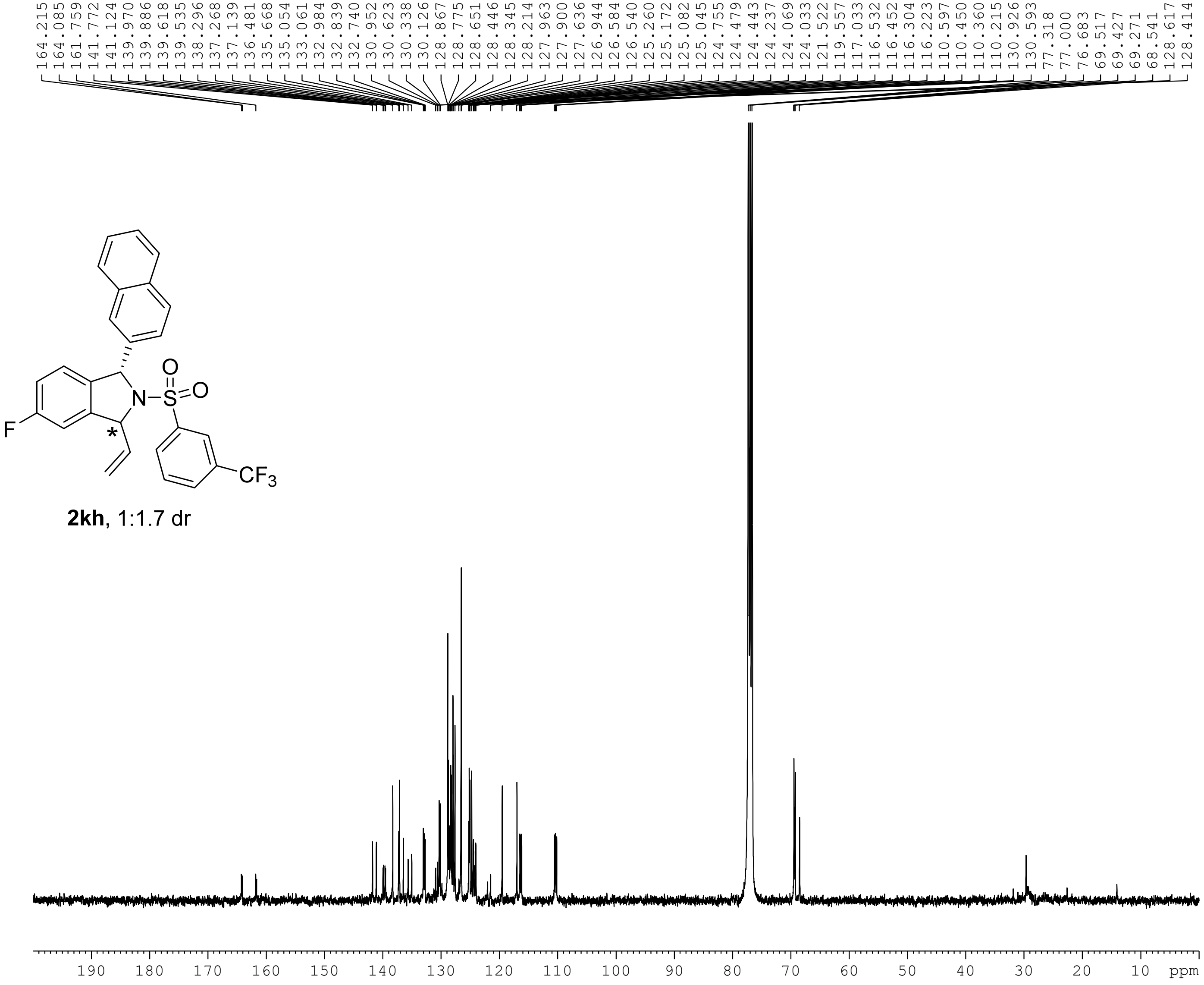

2kh, 1:1.7 dr

NAME	202409	
EXPNO	63	
PROCNO	1	
Date_	20240914	
Time	9.37	
INSTRUM	spect	
PROBHD	5 mm PABBO BB/	
PULPROG	zgpg30	
TD	65536	
SOLVENT	CDCl3	
NS	15500	
DS	0	
SWH	24038.461	Hz
FIDRES	0.366798	Hz
AQ	1.3631988	sec
RG	205.92	
DW	20.800	usec
DE	6.50	usec
TE	291.5	K
D1	2.00000000	sec
D11	0.03000000	sec
TD0	1	

======== CHANNEL f1 ========

SFO1	100.6233329	MHz
NUC1	13C	
P1	10.00	usec
SI	32768	
SF	100.6127723	MHz
WDW	EM	
SSB	0	
LB	2.00	Hz
GB	0	
PC	1.00	

^{19}F NMR of **2kh** ($CDCl_3$, 376 MHz)

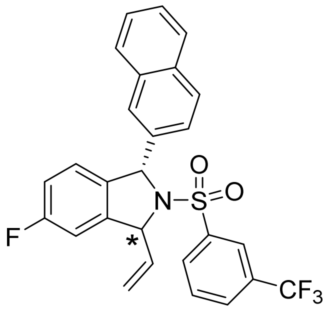

2kh, 1:1.7 dr

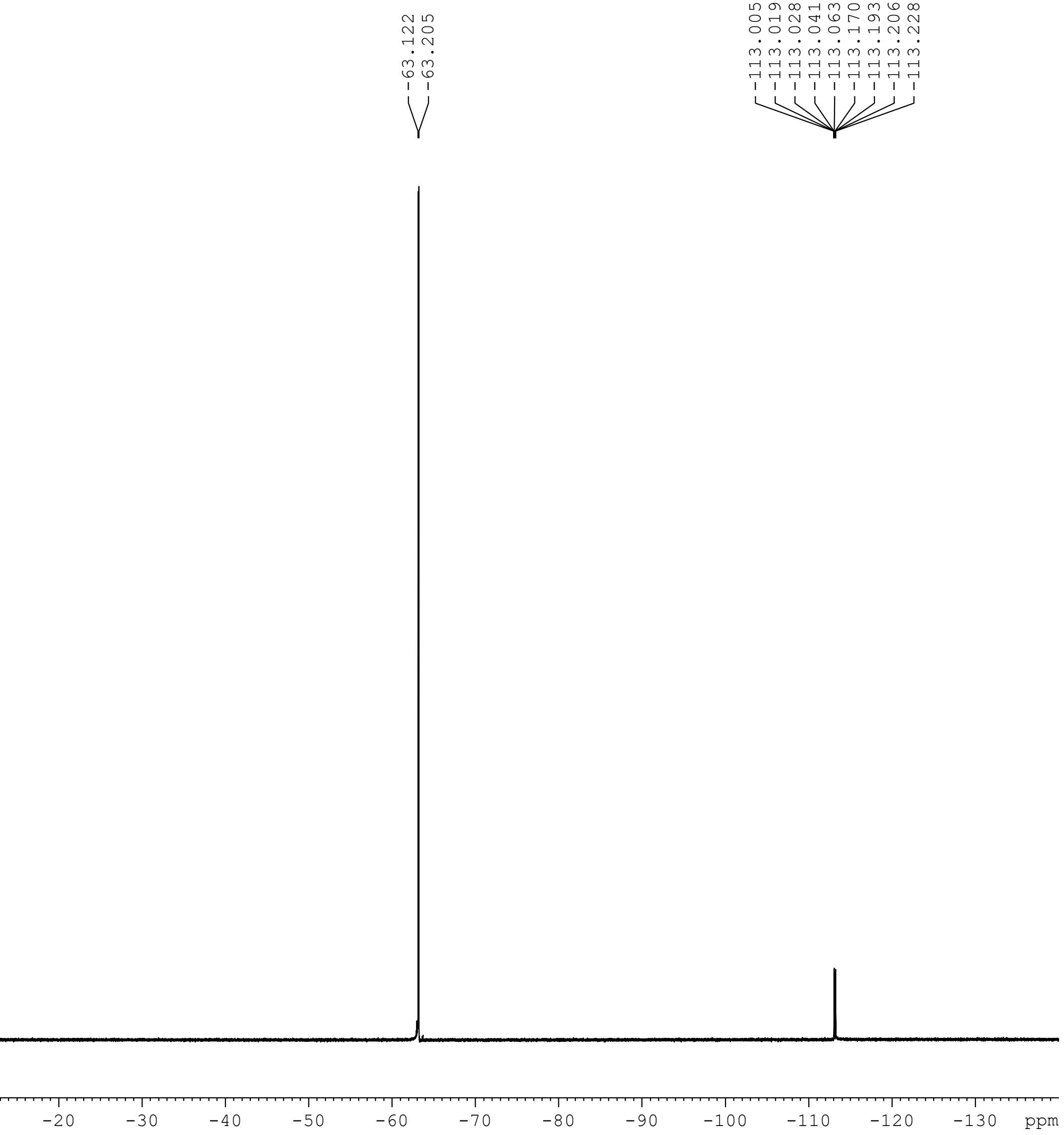

NAME	202409	
EXPNO	65	
PROCNO	1	
Date_	20240914	
Time	14.43	
INSTRUM	spect	
PROBHD	5 mm PABBO BB/	
PULPROG	zg30	
TD	131072	
SOLVENT	CDCl3	
NS	16	
DS	0	
SWH	89285.711	Hz
FIDRES	0.681196	Hz
AQ	0.7340532	sec
RG	205.92	
DW	5.600	usec
DE	6.50	usec
TE	290.1	K
D1	1.00000000	sec
TD0	1	
======== CHANNEL f1 ========		
SFO1	376.4757776	MHz
NUC1	19F	
P1	15.00	usec
SI	65536	
SF	376.4983662	MHz
WDW	EM	
SSB	0	
LB	0.30	Hz
GB	0	
PC	1.00	

^{1}H NMR of **2lh** ($CDCl_3$, 400 MHz)

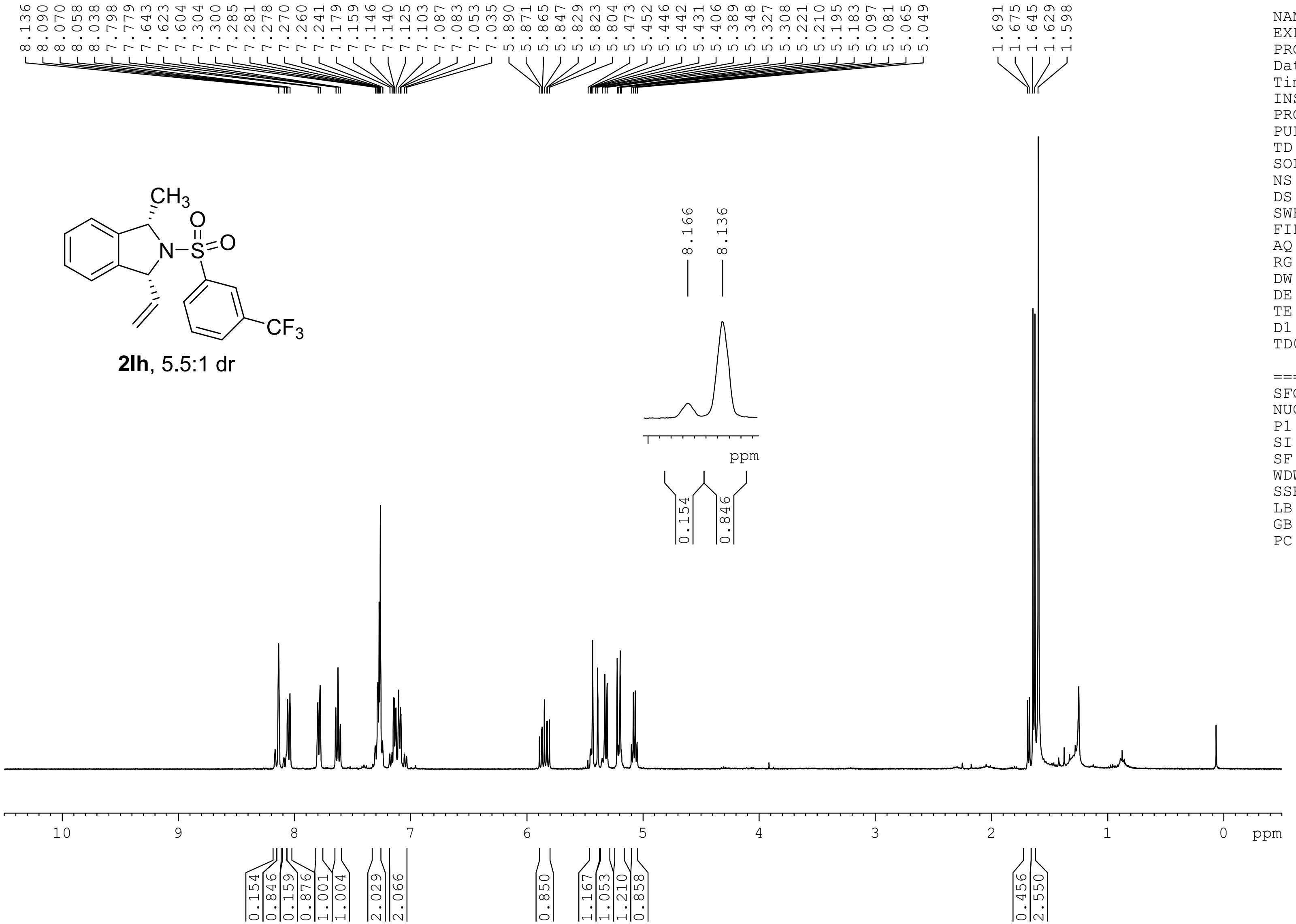

2lh, 5.5:1 dr

NAME 202407
EXPNO 94
PROCNO 1
Date_ 20240703
Time 22.04
INSTRUM spect
PROBHD 5 mm PABBO BB/
PULPROG zg30
TD 32768
SOLVENT CDCl3
NS 16
DS 0
SWH 8012.820 Hz
FIDRES 0.244532 Hz
AQ 2.0447731 sec
RG 205.92
DW 62.400 usec
DE 16.53 usec
TE 290.2 K
D1 2.00000000 sec
TD0 1

======== CHANNEL f1 ========
SFO1 400.1324008 MHz
NUC1 1H
P1 14.00 usec
SI 16384
SF 400.1300096 MHz
WDW EM
SSB 0
LB 0.00 Hz
GB 0
PC 1.00

$^{13}C\{^1H\}$ NMR of **2lh** ($CDCl_3$, 101 MHz)

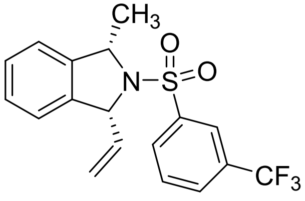

2lh, 5.5:1 dr

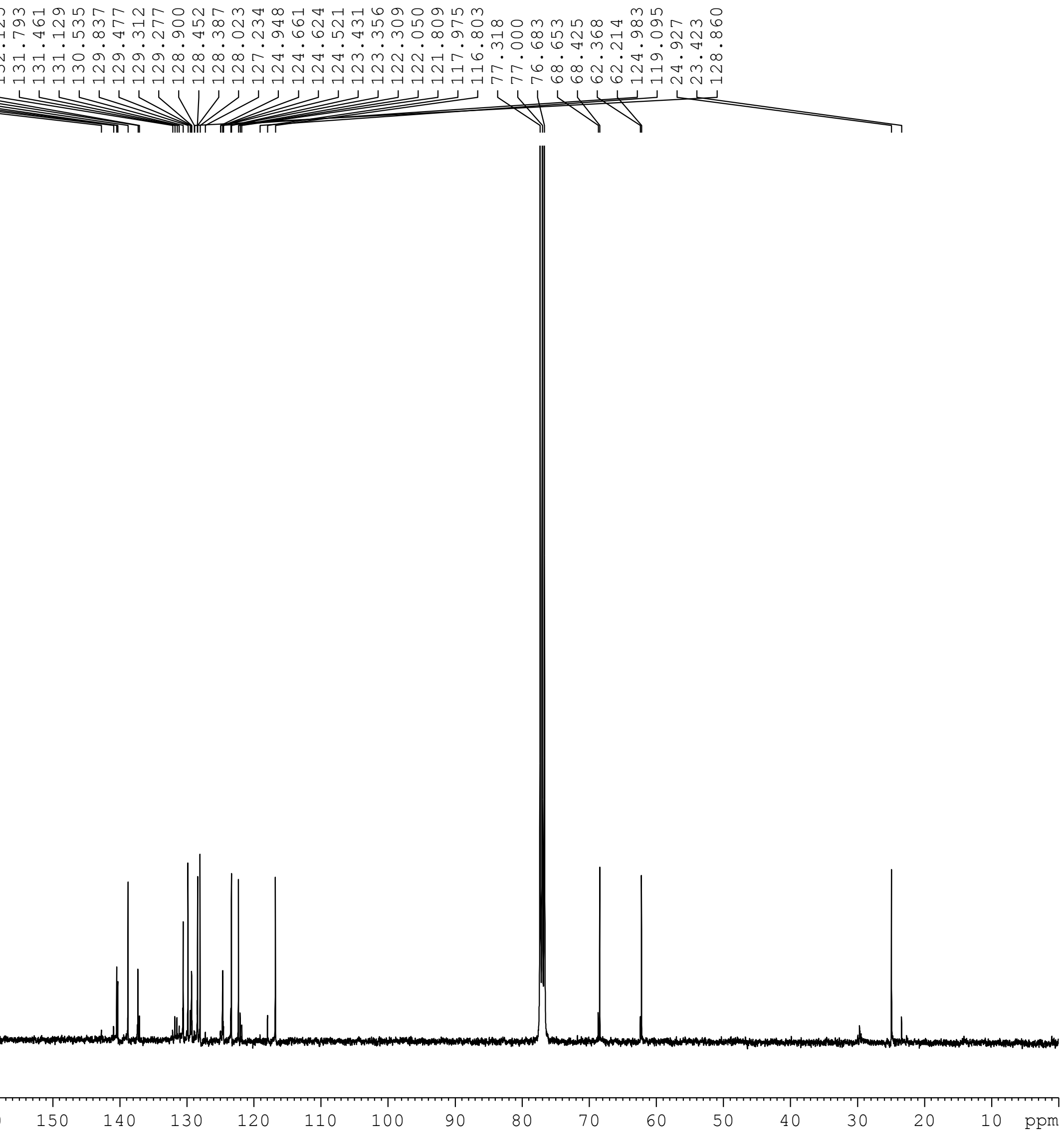

NAME	202407
EXPNO	96
PROCNO	1
Date_	20240704
Time	2.41
INSTRUM	spect
PROBHD	5 mm PABBO BB/
PULPROG	zgpg30
TD	32768
SOLVENT	CDCl3
NS	6000
DS	0
SWH	24038.461 Hz
FIDRES	0.733596 Hz
AQ	0.6816244 sec
RG	205.92
DW	20.800 usec
DE	6.50 usec
TE	291.6 K
D1	2.00000000 sec
D11	0.03000000 sec
TD0	1

======== CHANNEL f1 ========

SFO1	100.6233329 MHz
NUC1	13C
P1	10.00 usec
SI	32768
SF	100.6127722 MHz
WDW	EM
SSB	0
LB	2.00 Hz
GB	0
PC	1.00

^{19}F NMR of **2lh** ($CDCl_3$, 376 MHz)

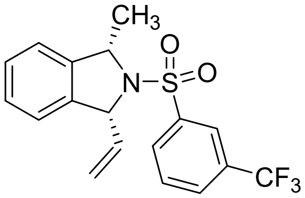

2lh, 5.5:1 dr

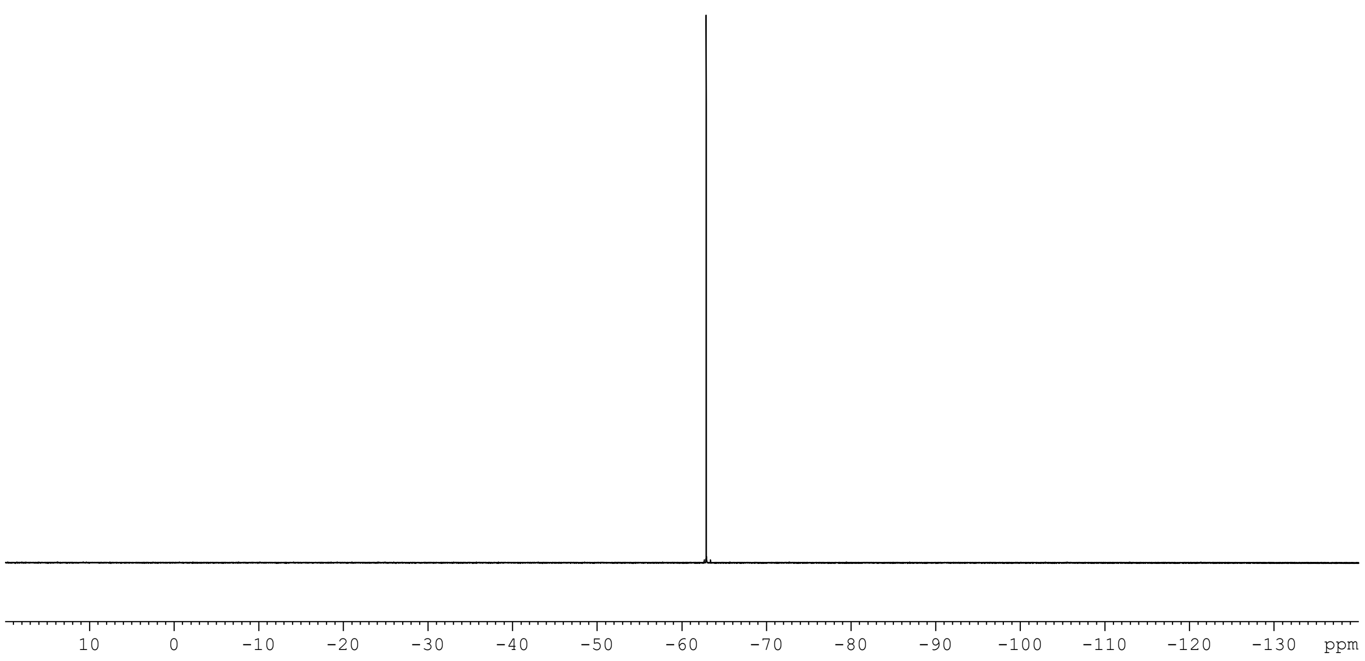


```
NAME              202407
EXPNO                 95
PROCNO                 1
Date_           20240703
Time               22.06
INSTRUM            spect
PROBHD   5 mm PABBO BB/
PULPROG             zg30
TD                131072
SOLVENT            CDCl3
NS                    16
DS                     0
SWH           89285.711 Hz
FIDRES         0.681196 Hz
AQ            0.7340532 sec
RG               205.92
DW                5.600 usec
DE                 6.50 usec
TE                290.2 K
D1           1.00000000 sec
TD0                    1

======== CHANNEL f1 ========
SFO1        376.4757776 MHz
NUC1                 19F
P1                 15.00 usec
SI                 65536
SF          376.4983662 MHz
WDW                   EM
SSB                    0
LB                  0.30 Hz
GB                     0
PC                  1.00
```

^{1}H NMR of (1*S*,3*R*)-**2lh** ($CDCl_3$, 400 MHz)

(1*S*,3*R*)-**2lh**, >20:1 dr
synthesized from **9c**

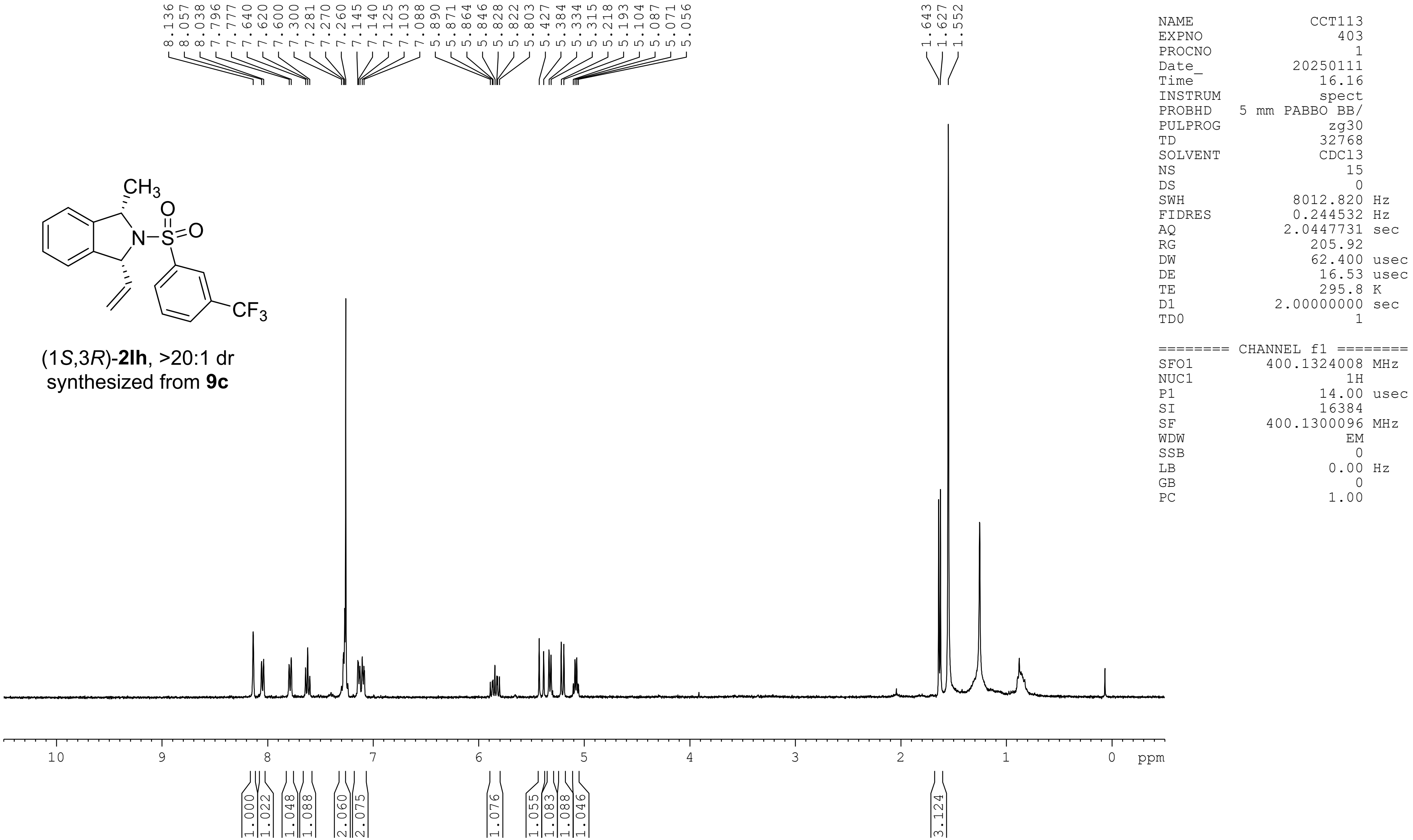

Zoomed-in 2D NMR of (1*S*,3*R*)-**2lh** ($CDCl_3$)

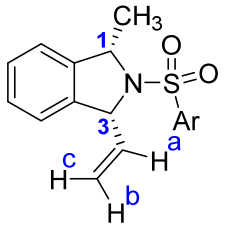

(1*S*,3*R*)-**2lh**, >20:1 dr
synthesized from **9c**
Ar = 3-$CF_3C_6H_4$

COSY

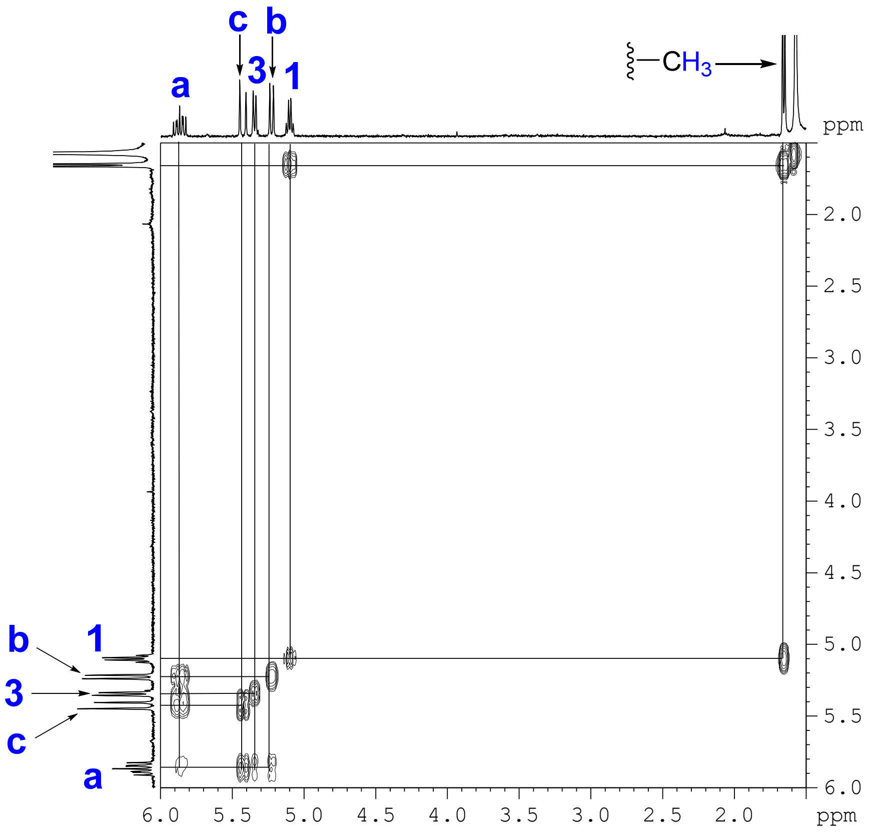

HMQC

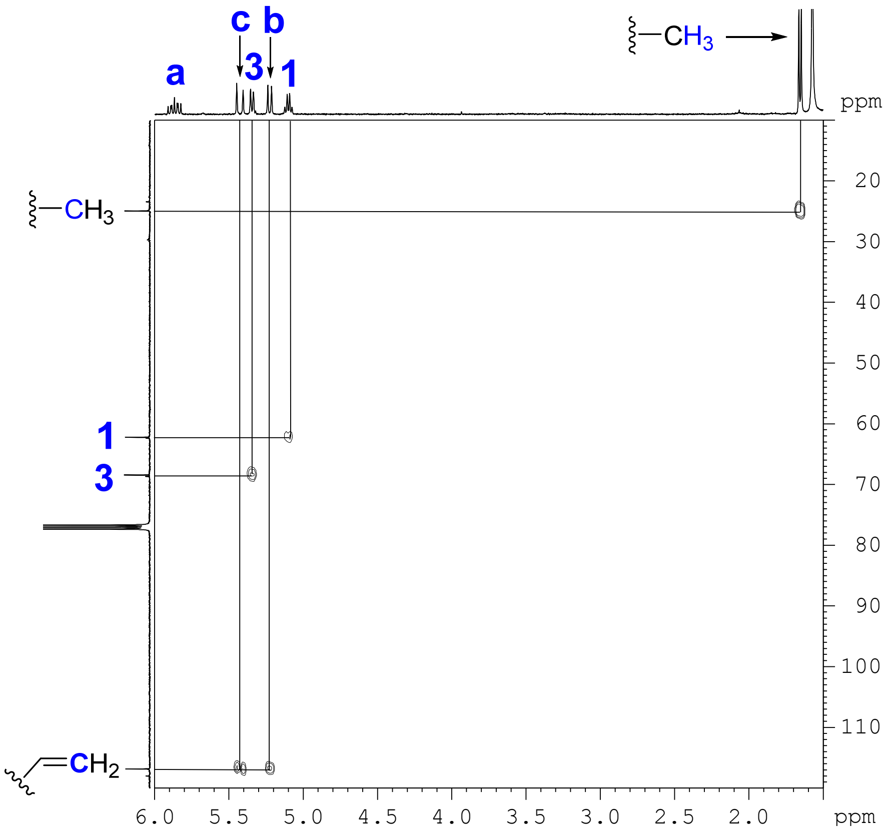

^{1}H NMR of **6** ($CDCl_3$, 400 MHz)

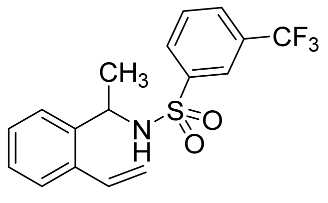

6

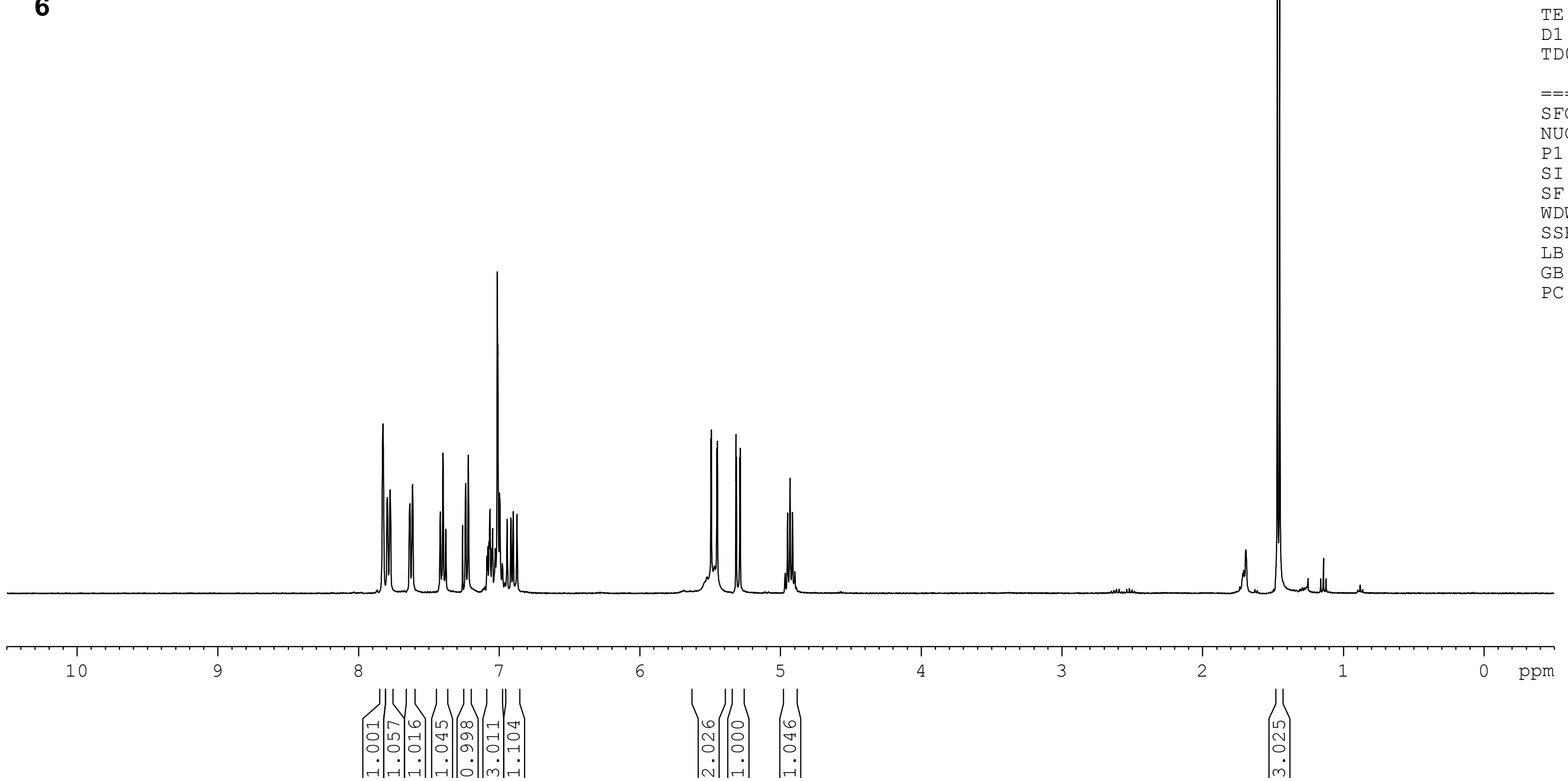

NAME	202406
EXPNO	230
PROCNO	1
Date_	20240619
Time	20.28
INSTRUM	spect
PROBHD	5 mm PABBO BB/
PULPROG	zg30
TD	32768
SOLVENT	CDCl3
NS	7
DS	0
SWH	8012.820 Hz
FIDRES	0.244532 Hz
AQ	2.0447731 sec
RG	101.51
DW	62.400 usec
DE	16.53 usec
TE	291.8 K
D1	2.00000000 sec
TD0	1
======== CHANNEL f1 ========	
SFO1	400.1324008 MHz
NUC1	1H
P1	14.00 usec
SI	16384
SF	400.1300096 MHz
WDW	EM
SSB	0
LB	0.00 Hz
GB	0
PC	1.00

$^{13}C\{^1H\}$ NMR of **6** ($CDCl_3$, 101 MHz)

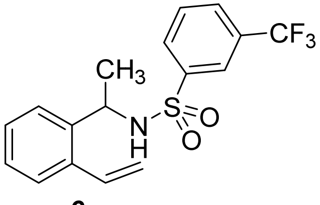

6

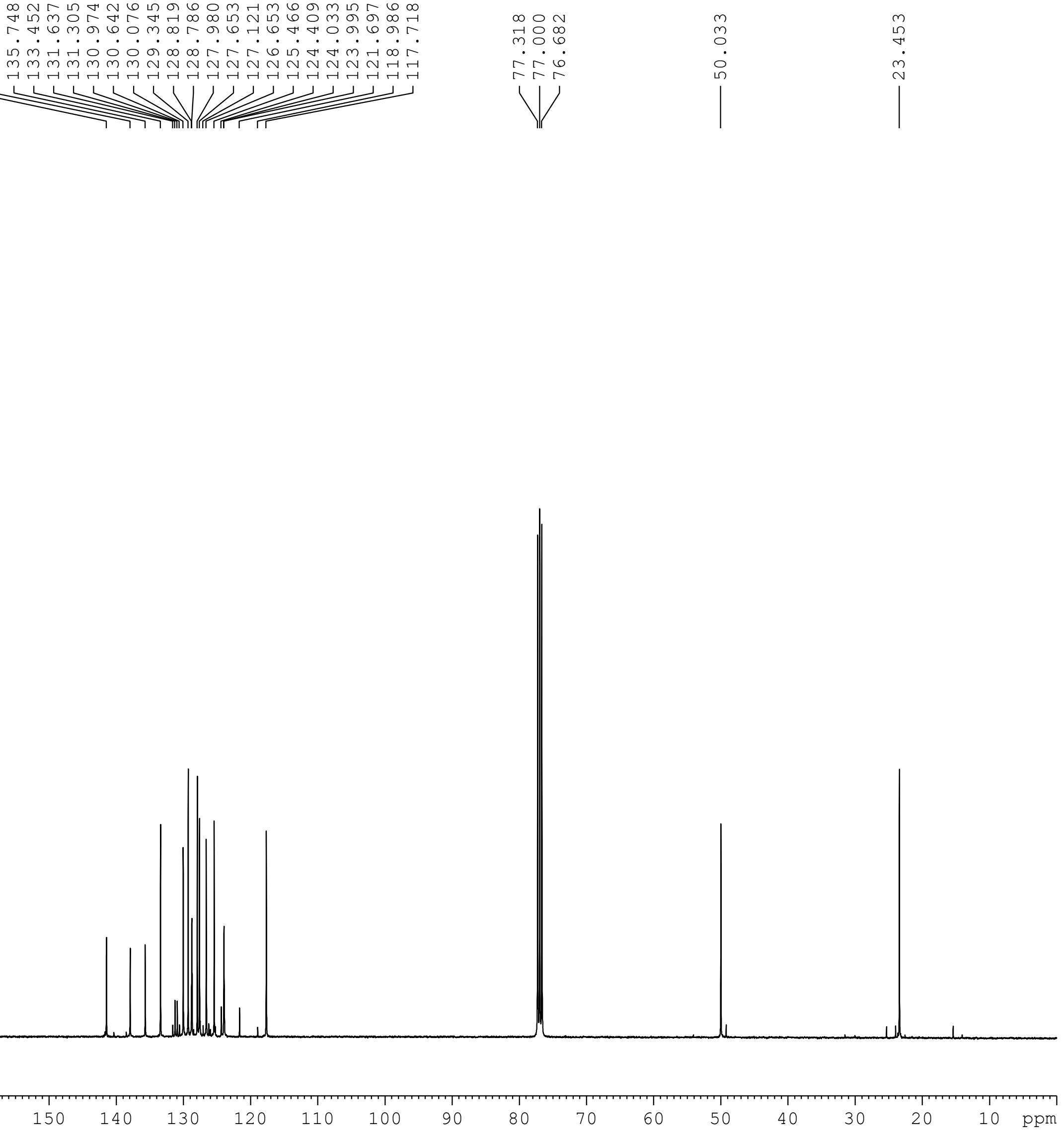

NAME	202406	
EXPNO	234	
PROCNO	1	
Date_	20240619	
Time	22.09	
INSTRUM	spect	
PROBHD	5 mm PABBO BB/	
PULPROG	zgpg30	
TD	32768	
SOLVENT	CDCl3	
NS	14000	
DS	0	
SWH	24038.461	Hz
FIDRES	0.733596	Hz
AQ	0.6816244	sec
RG	205.92	
DW	20.800	usec
DE	6.50	usec
TE	291.5	K
D1	2.00000000	sec
D11	0.03000000	sec
TD0	1	
======== CHANNEL f1 ========		
SFO1	100.6233329	MHz
NUC1	13C	
P1	10.00	usec
SI	32768	
SF	100.6127734	MHz
WDW	EM	
SSB	0	
LB	2.00	Hz
GB	0	
PC	1.00	

^{19}F NMR of **6** ($CDCl_3$, 376 MHz)

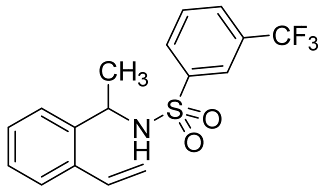

6

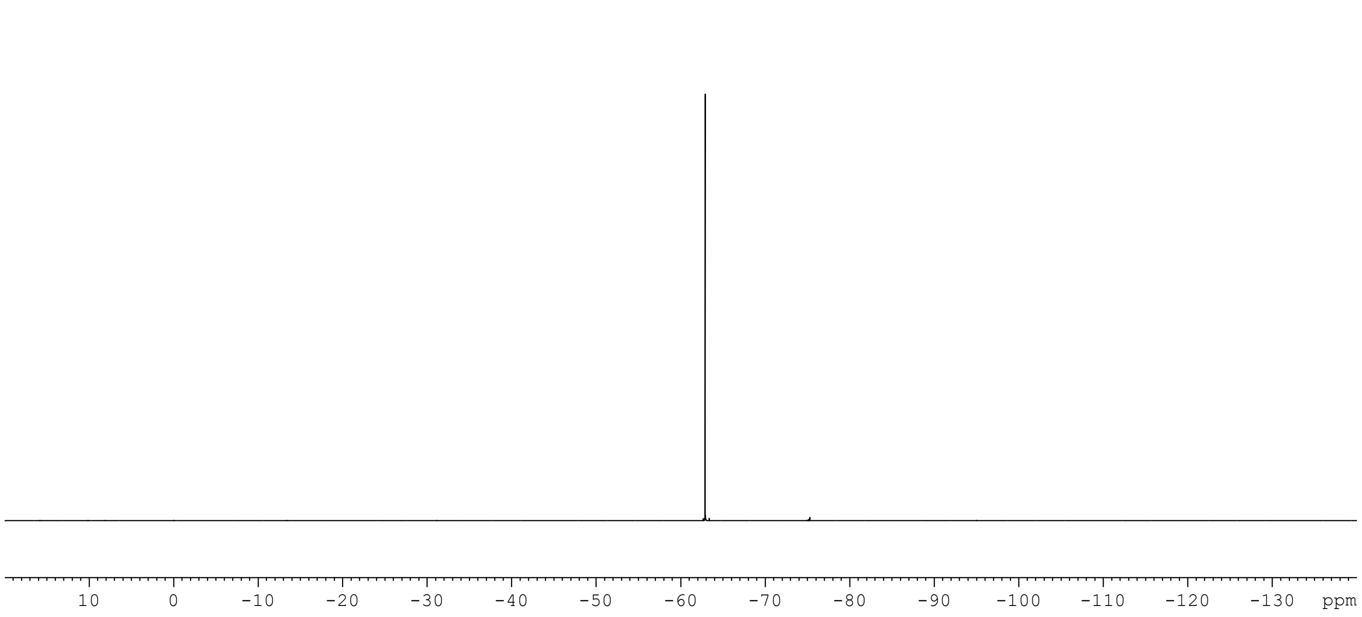


```
NAME              202406
EXPNO                238
PROCNO                 1
Date_           20240620
Time               11.35
INSTRUM            spect
PROBHD   5 mm PABBO BB/
PULPROG             zg30
TD                131072
SOLVENT            CDCl3
NS                    10
DS                     0
SWH            89285.711 Hz
FIDRES          0.681196 Hz
AQ             0.7340532 sec
RG                205.92
DW                 5.600 usec
DE                  6.50 usec
TE                 292.2 K
D1            1.00000000 sec
TD0                    1

======== CHANNEL f1 ========
SFO1         376.4757776 MHz
NUC1                 19F
P1                 15.00 usec
SI                 65536
SF           376.4983662 MHz
WDW                   EM
SSB                    0
LB                  0.30 Hz
GB                     0
PC                  1.00
```

^{1}H NMR of **7** (CD_3OD, 400 MHz)

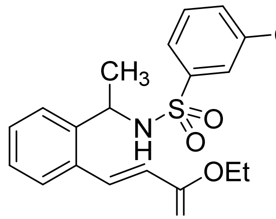

7

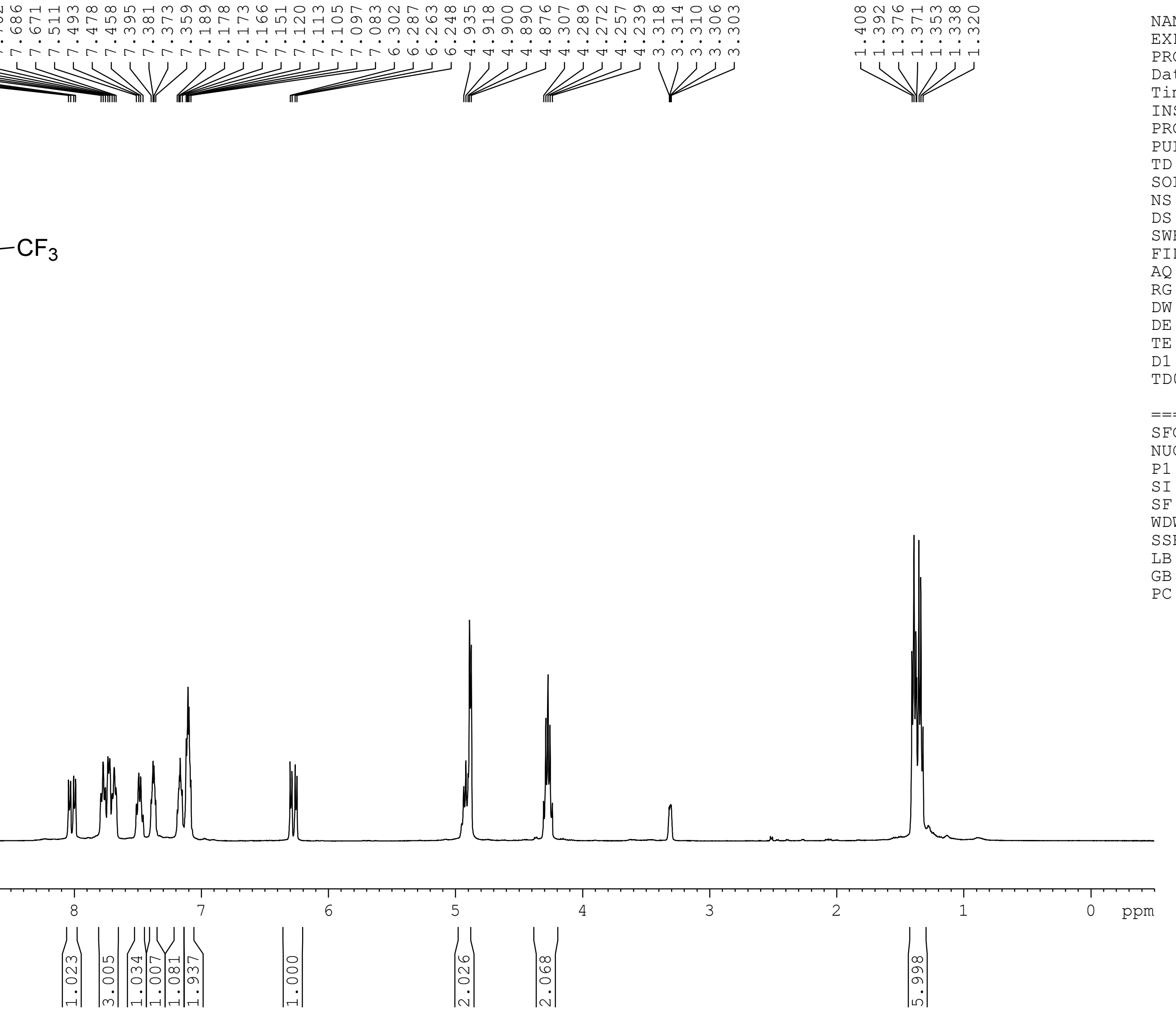

NAME	202406
EXPNO	519
PROCNO	1
Date_	20240628
Time	20.04
INSTRUM	spect
PROBHD	5 mm PABBO BB/
PULPROG	zg30
TD	32768
SOLVENT	MeOD
NS	30
DS	0
SWH	8012.820 Hz
FIDRES	0.244532 Hz
AQ	2.0447731 sec
RG	55.28
DW	62.400 usec
DE	16.53 usec
TE	291.9 K
D1	2.00000000 sec
TD0	1

======== CHANNEL f1 ========

SFO1	400.1324008 MHz
NUC1	1H
P1	14.00 usec
SI	16384
SF	400.1300115 MHz
WDW	EM
SSB	0
LB	0.00 Hz
GB	0
PC	1.00

$^{13}C\{^{1}H\}$ NMR of **7** ($CDCl_3$, 101 MHz)

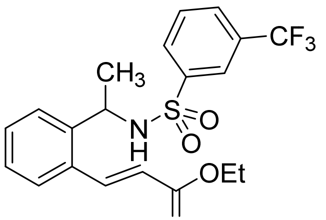

7

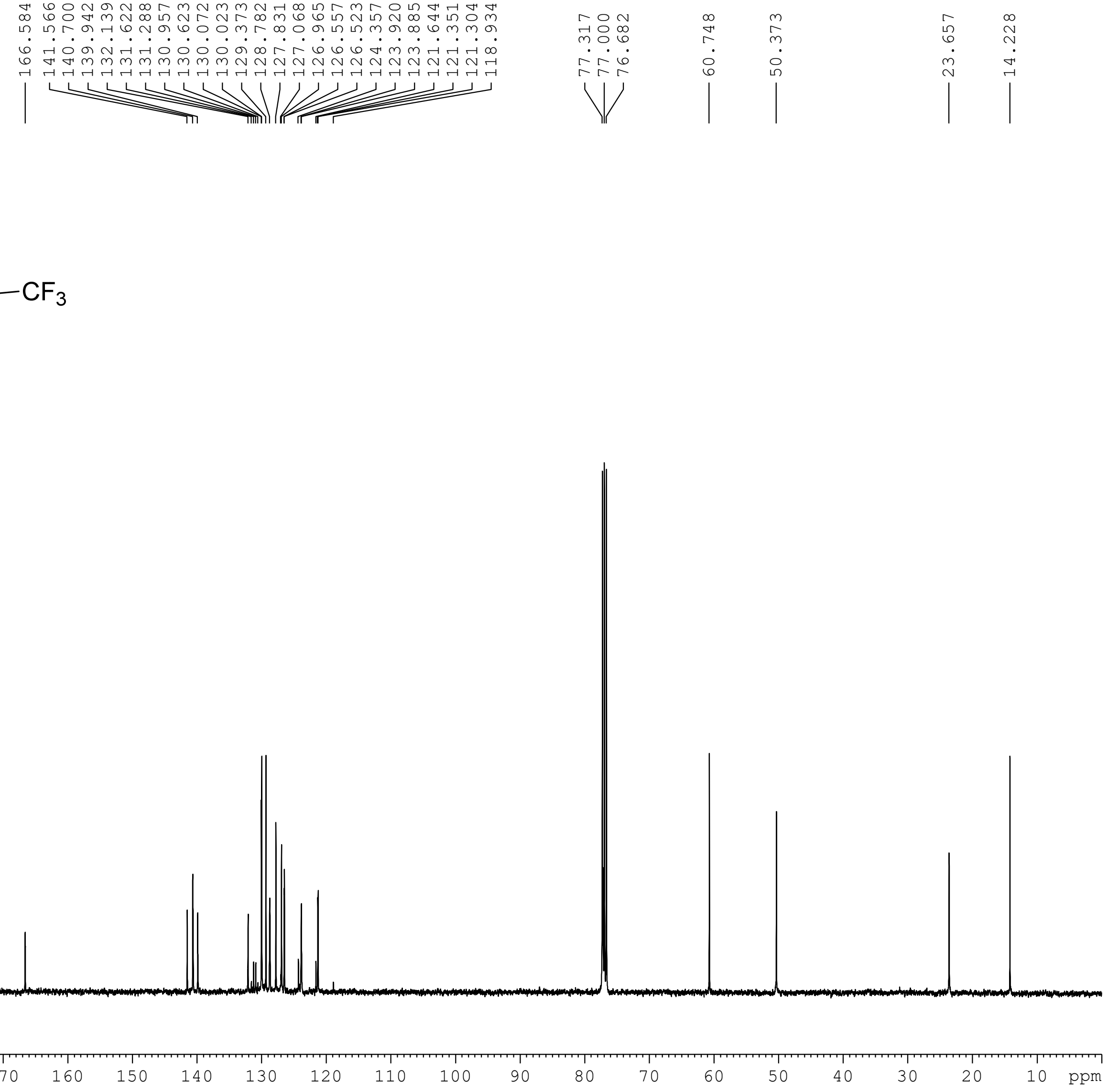

NAME	202406	
EXPNO	292	
PROCNO	1	
Date_	20240622	
Time	0.20	
INSTRUM	spect	
PROBHD	5 mm PABBO BB/	
PULPROG	zgpg30	
TD	32768	
SOLVENT	CDCl3	
NS	4000	
DS	0	
SWH	24038.461	Hz
FIDRES	0.733596	Hz
AQ	0.6816244	sec
RG	205.92	
DW	20.800	usec
DE	6.50	usec
TE	292.9	K
D1	2.00000000	sec
D11	0.03000000	sec
TD0	1	

======== CHANNEL f1 ========

SFO1	100.6233329	MHz
NUC1	13C	
P1	10.00	usec
SI	32768	
SF	100.6127747	MHz
WDW	EM	
SSB	0	
LB	2.00	Hz
GB	0	
PC	1.00	

^{19}F NMR of **7** ($CDCl_3$, 376 MHz)

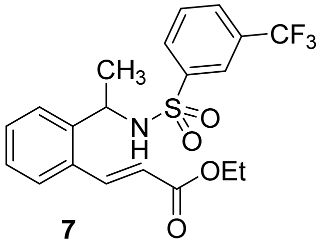

7

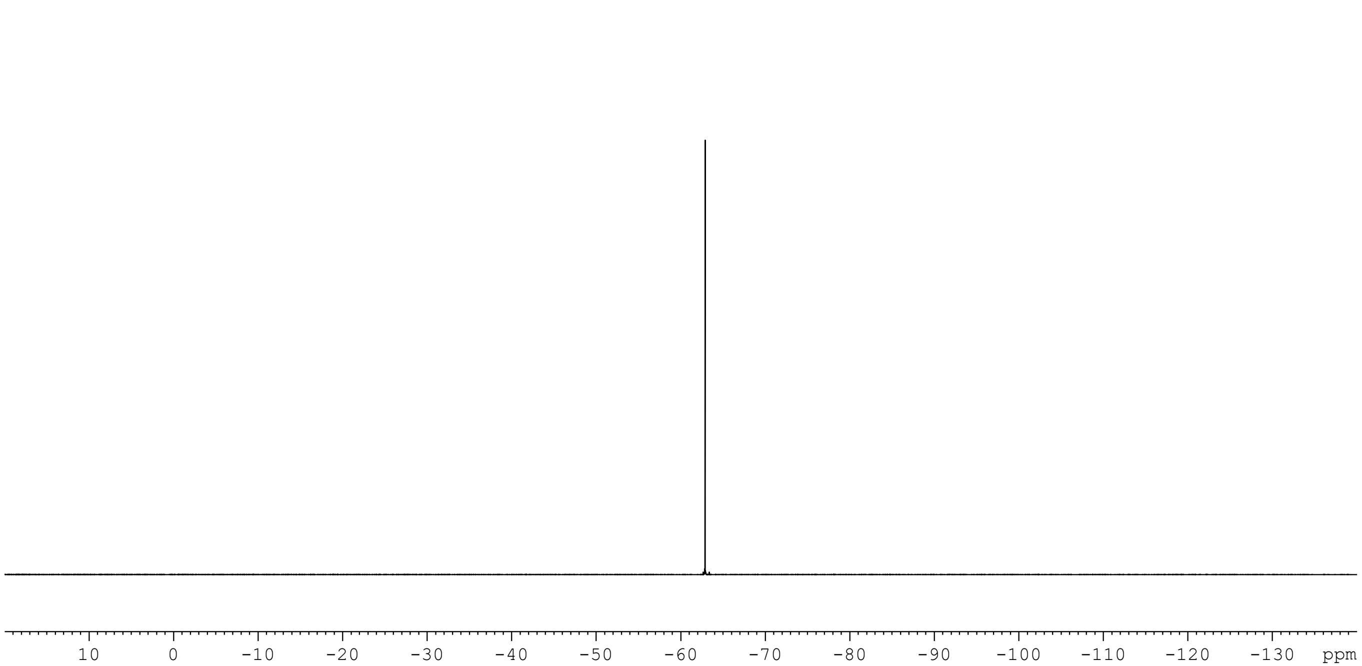


```
NAME                202406
EXPNO                  289
PROCNO                   1
Date_             20240621
Time                 21.46
INSTRUM              spect
PROBHD    5 mm PABBO BB/
PULPROG               zg30
TD                  131072
SOLVENT              CDCl3
NS                      10
DS                       0
SWH              89285.711 Hz
FIDRES            0.681196 Hz
AQ               0.7340532 sec
RG                  205.92
DW                   5.600 usec
DE                    6.50 usec
TE                   291.6 K
D1              1.00000000 sec
TD0                      1

======== CHANNEL f1 ========
SFO1        376.4757776 MHz
NUC1                19F
P1                15.00 usec
SI                65536
SF          376.4983662 MHz
WDW                  EM
SSB                   0
LB                 0.30 Hz
GB                    0
PC                 1.00
```

10. HPLC Data

HPLC of **1aa**

Racemic

<Sample Information>

Data Filename : 230706_1aa_racemic_v2_90;10_2rd_792023_002.lcd
Method Filename : V2_H91_I09_R1_60min.lcm
Batch Filename : 230706_1aa_racemic_v2_90:10_2rd.lcb

<Chromatogram>

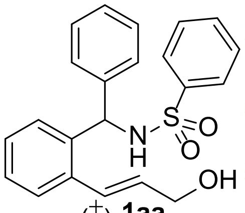

(±)-**1aa**

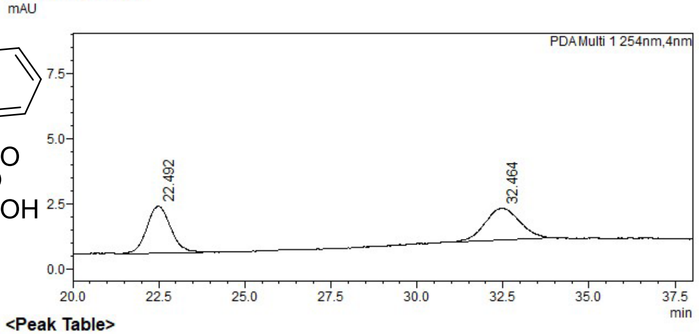

<Peak Table>

PDA Ch1 254nm

Peak#	Ret. Time	Area%
1	22.492	50.485
2	32.464	49.515
Total		100.000

Chiral

BA catalyst: (*R*)-**CPA1**

solvent: DCE

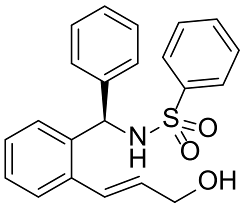

(*R*)-**1aa**

<Sample Information>

Data Filename : 240423_chiral_1aaDCE2h10m(S)_4232024_002.lcd
Method Filename : V2_H91_I09_R1_40min.lcm
Batch Filename : 240423_chiral_1aaDCE2h10m(S).lcb

<Chromatogram>

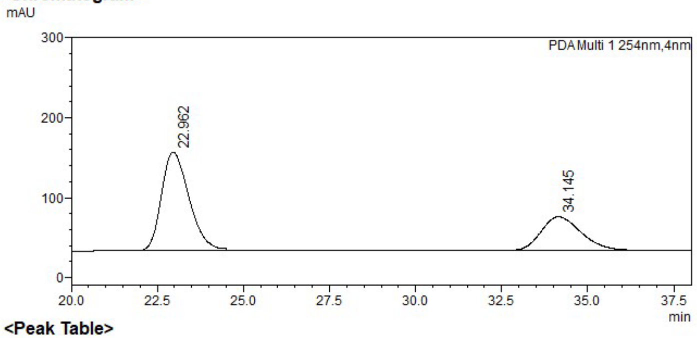

<Peak Table>

PDA Ch1 254nm

Peak#	Ret. Time	Area%
1	22.962	66.844
2	34.145	33.156
Total		100.000

Authentic Compound

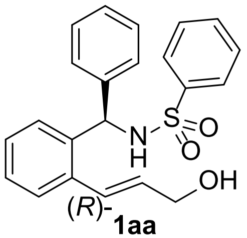

(*R*)-**1aa**

synthesized from **8**

<Sample Information>

Data Filename : 241216-1_20241216_002.lcd
Method Filename : V2_H91_I09_R1_60min.lcm
Batch Filename : 241216-1.lcb

<Chromatogram>

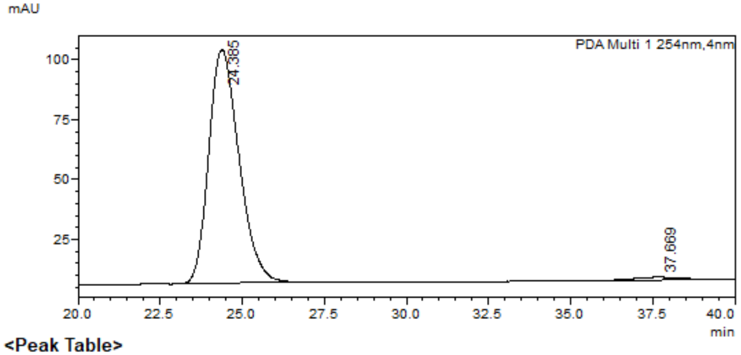

<Peak Table>

PDA Ch1 254nm

Peak#	Ret. Time	Area%
1	24.385	98.375
2	37.669	1.625
Total		100.000

HPLC of **1ab**

Racemic

<Sample Information>

Data Filename : 20230418 V4 IC 7525 4tbu rac S sm_4182023_002.lcd
Method Filename : V4_H75_I25_R1_30min.lcm
Batch Filename : 20230418 V4 IC 7525 4tbu rac S sm.lcb

<Chromatogram>

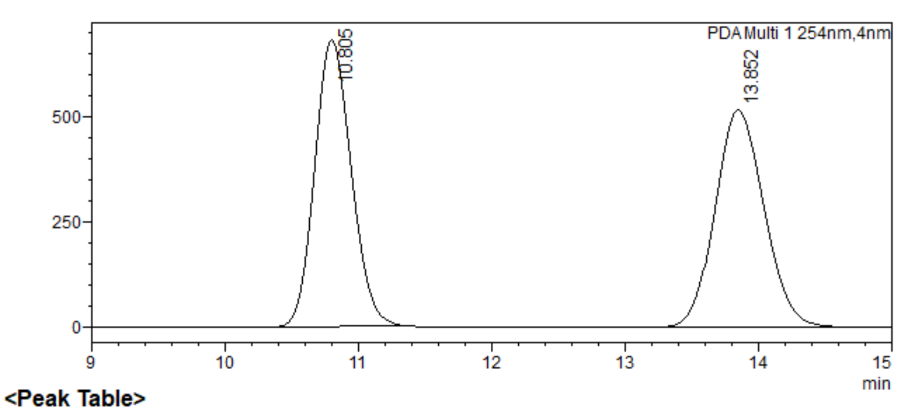

<Peak Table>

PDA Ch1 254nm

Peak#	Ret. Time	Area%
1	10.805	49.931
2	13.852	50.069
Total		100.000

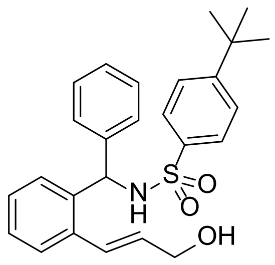

(±)-**1ab**

Chiral

BA catalyst: (*R*)-**CPA1**

Solvent: DCE

<Sample Information>

Data Filename : 240513_chiral_1af(4tbu)DCECPA6_6h15m(S)_5132024_002.lcd
Method Filename : V4_H75_I25_R1_20min.lcm
Batch Filename : 240513_chiral_1af(4tbu)DCECPA6_6h15m(S).lcb

<Chromatogram>

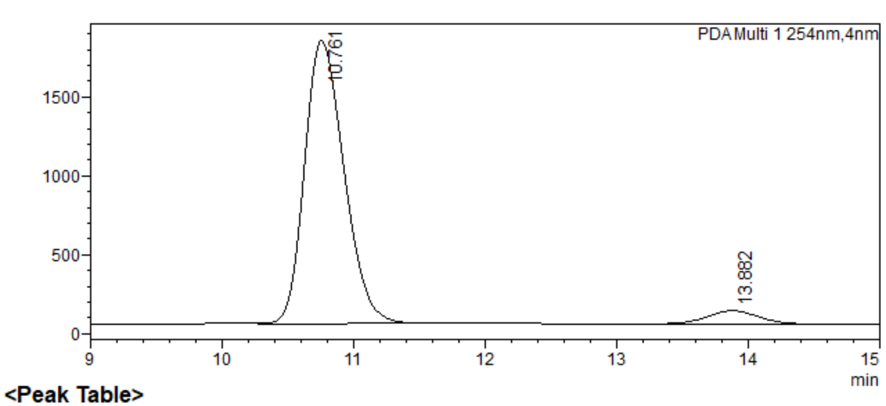

<Peak Table>

PDA Ch1 254nm

Peak#	Ret. Time	Area%
1	10.761	94.168
2	13.882	5.832
Total		100.000

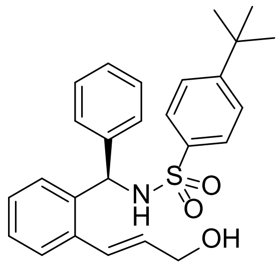

(*R*)-**1ab**

HPLC of **1ac**

Racemic

<Sample Information>

Data Filename : 240508_rac1ah(4F)_ODH85_ADH80_OJ82_582024_002.lcd
Method Filename : V2_H85_I15_R1_30min.lcm
Batch Filename : 240508_rac1ah(4F)_ODH85_ADH80_OJ82.lcb

<Chromatogram>

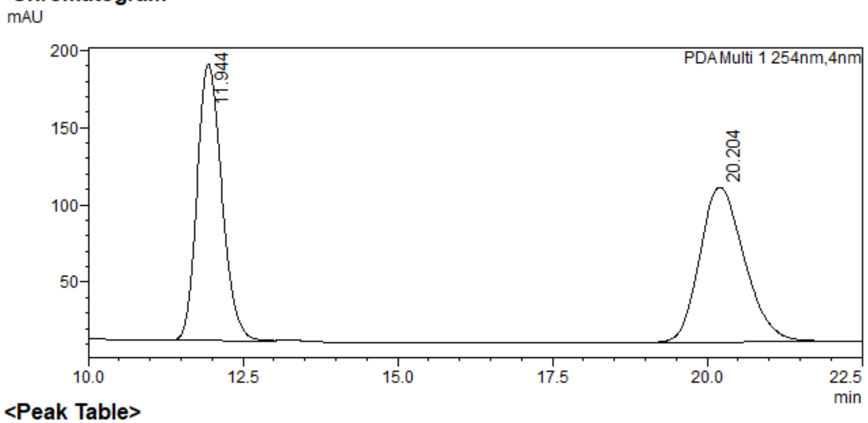

<Peak Table>

PDA Ch1 254nm

Peak#	Ret. Time	Area%
1	11.944	49.791
2	20.204	50.209
Total		100.000

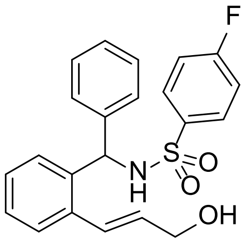

(±)-**1ac**

Chiral

BA catalyst: (*R*)-**CPA1**

Solvent: DCE

<Sample Information>

Data Filename : 240508_chiral_1ah(4-F)DCECPA6_4h30m(S)_582024_002.lcd
Method Filename : V2_H85_I15_R1_30min.lcm
Batch Filename : 240508_chiral_1ah(4-F)DCECPA6_4h30m(S).lcb

<Chromatogram>

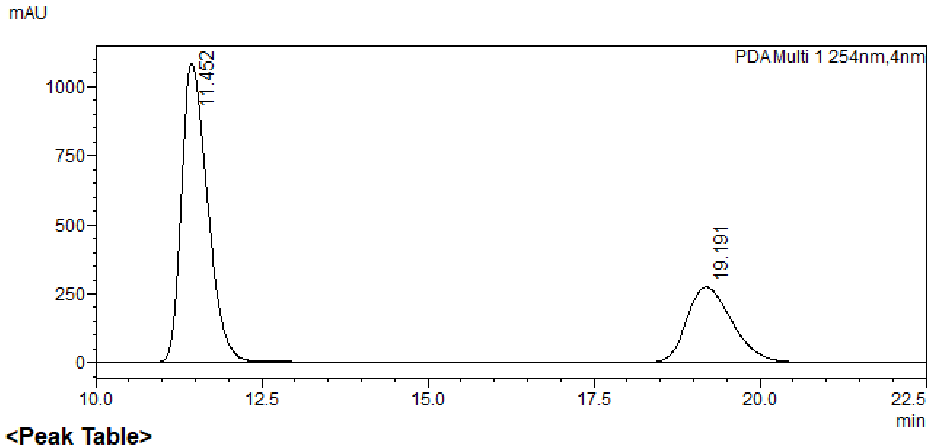

<Peak Table>

PDA Ch1 254nm

Peak#	Ret. Time	Area%
1	11.452	69.448
2	19.191	30.552
Total		100.000

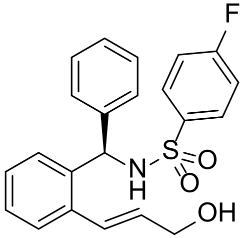

(*R*)-**1ac**

HPLC of **1ad**

Racemic

<Sample Information>

Data Filename : 20230330 V4 IC 9010 4cf3 4ome rac sm_3302023_002.lcd
Method Filename : V4_H90_I10_R1_30min.lcm
Batch Filename : 20230330 V4 IC 9010 4cf3 4ome rac sm.lcb

<Chromatogram>

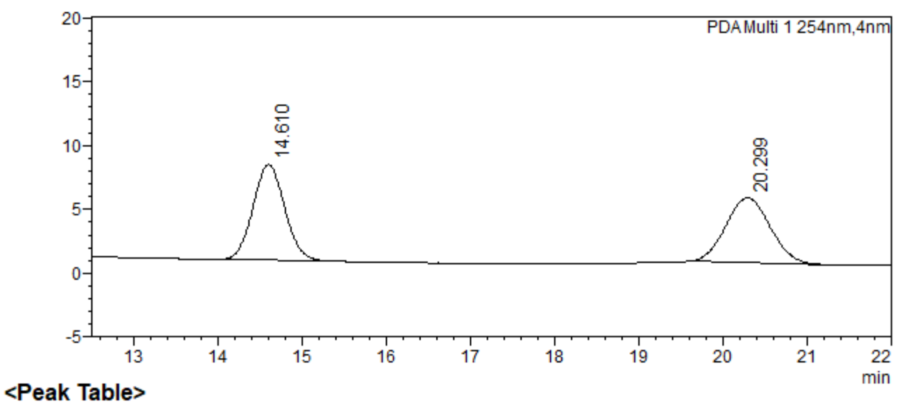

<Peak Table>

PDA Ch1 254nm

Peak#	Ret. Time	Area%
1	14.610	50.960
2	20.299	49.040
Total		100.000

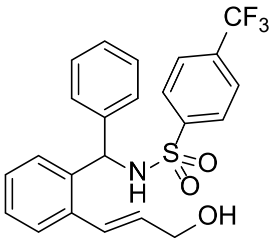

(±)-**1ad**

Chiral

BA catalyst: (*R*)-**CPA1**

Solvent: DCE

<Sample Information>

Data Filename : 240528_chiral_1ai(4-CF3)_DCECPA6_1h55m(S)_5282024_002.lcd
Method Filename : V4_H90_I10_R1_30min.lcm
Batch Filename : 240528_chiral_1ai(4-CF3)_DCECPA6_1h55m(S).lcb

<Chromatogram>

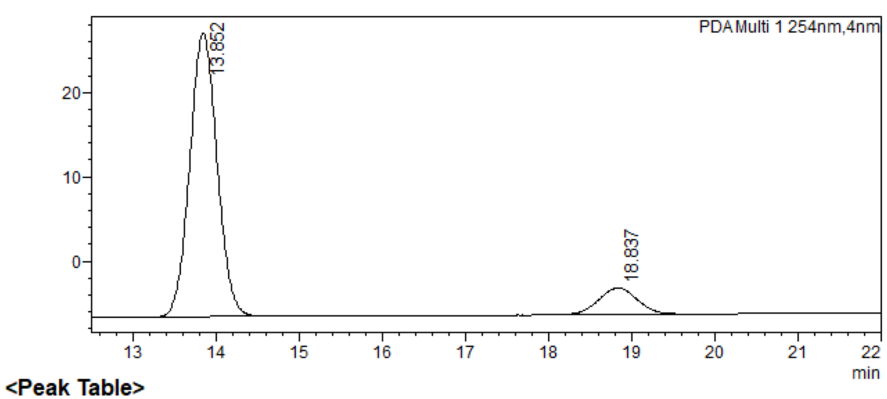

<Peak Table>

PDA Ch1 254nm

Peak#	Ret. Time	Area%
1	13.852	88.397
2	18.837	11.603
Total		100.000

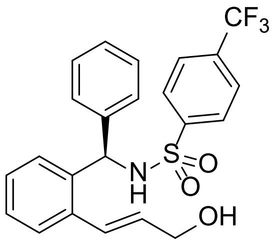

(*R*)-**1ad**

HPLC of **1ae**

Racemic

<Sample Information>

Data Filename : 240920_1ac_H88 I12 30min_9202024_002.lcd
Method Filename : V4_H88_I12_R1_30min.lcm
Batch Filename : 240920_1ac_H88 I12 30min.lcb

<Chromatogram>

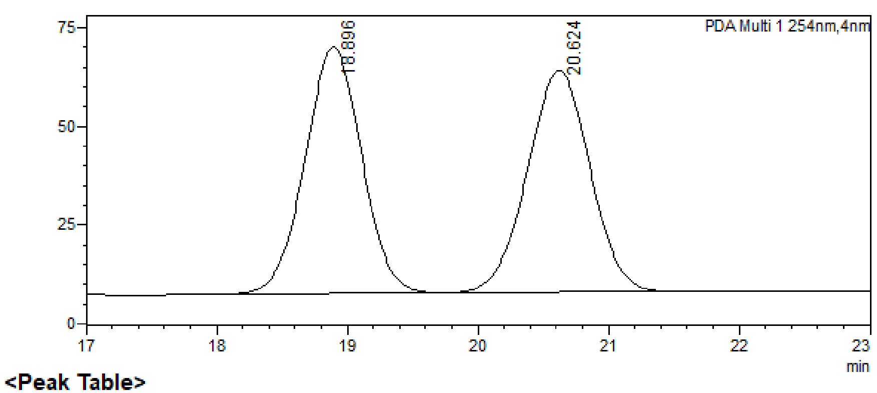

<Peak Table>

PDA Ch1 254nm

Peak#	Ret. Time	Area%
1	18.896	50.189
2	20.624	49.811
Total		100.000

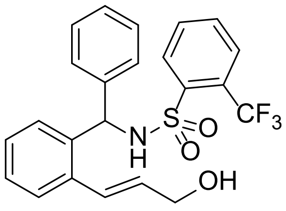

(±)-**1ae**

Chiral

BA catalyst: (*R*)-**CPA1**

Solvent: DCE

<Sample Information>

Data Filename : 240923_chiral_1ac_H88 I12 30min_9232024_002.lcd
Method Filename : V4_H88_I12_R1_30min.lcm
Batch Filename : 240923_chiral_1ac_H88 I12 30min.lcb

<Chromatogram>

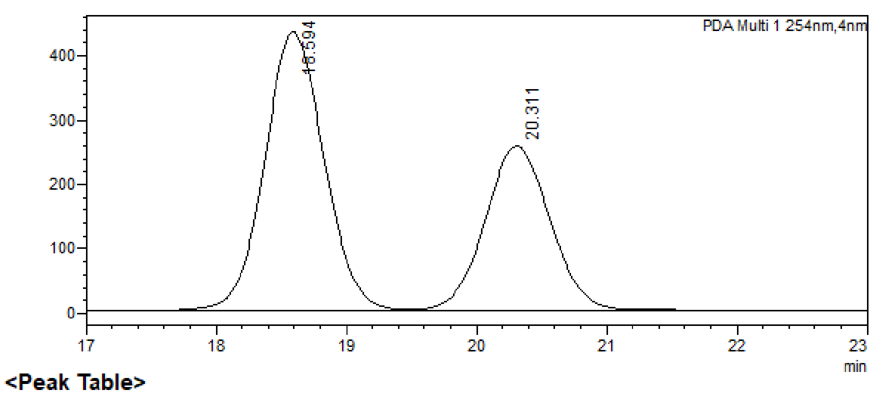

<Peak Table>

PDA Ch1 254nm

Peak#	Ret. Time	Area%
1	18.594	60.769
2	20.311	39.231
Total		100.000

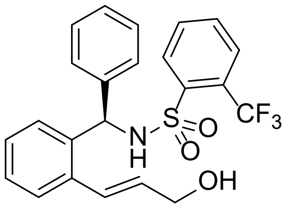

(*R*)-**1ae**

HPLC of **1af**

Racemic

<Sample Information>

Data Filename : 20230323 V2 ODH 9109 nso2ph2me rac sm_3232023_002.lcd
Method Filename : V2_H91_I09_R1_30min.lcm
Batch Filename : 20230323 V2 ODH 9109 nso2ph2me rac sm.lcb

<Chromatogram>

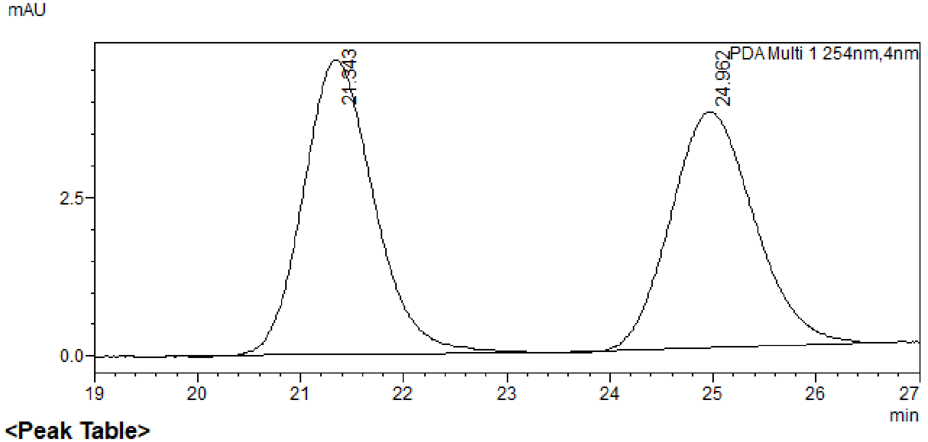

<Peak Table>

PDA Ch1 254nm

Peak#	Ret. Time	Area%
1	21.343	51.308
2	24.962	48.692
Total		100.000

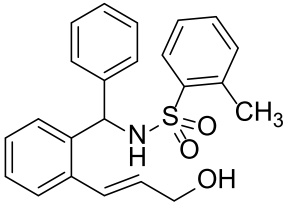

(±)-**1af**

Chiral

BA catalyst: (*R*)-**CPA1**

Solvent: DCE

<Sample Information>

Data Filename : 240516_1ab_DCErt_CPA6_4h40m30mg(S)_5162024_002.lcd
Method Filename : V2_H91_I09_R1_30min.lcm
Batch Filename : 240516_1ab_DCErt_CPA6_4h40m30mg(S).lcb

<Chromatogram>

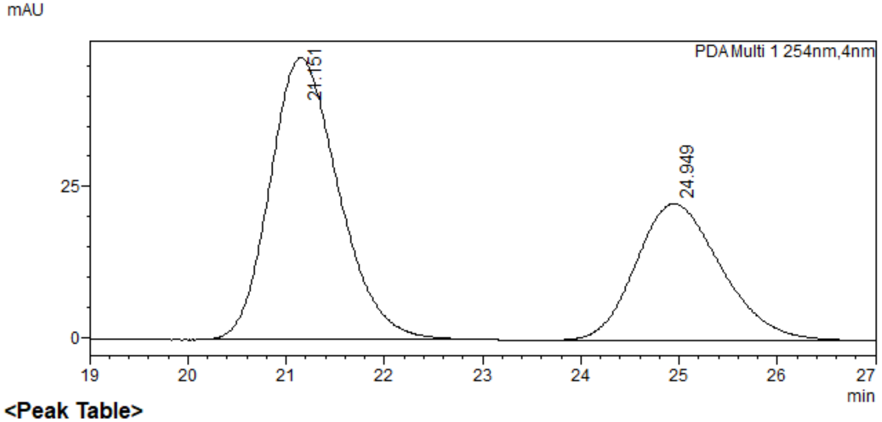

<Peak Table>

PDA Ch1 254nm

Peak#	Ret. Time	Area%
1	21.151	62.509
2	24.949	37.491
Total		100.000

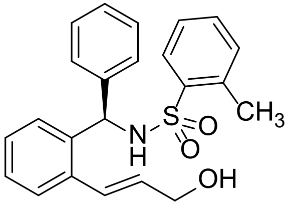

(*R*)-**1af**

HPLC of **1ah**

Racemic

<Sample Information>

Data Filename : 230629_test_1ak_racemic_6302023_005.lcd
Method Filename : V2_H90_I10_R1_30min.lcm
Batch Filename : 230629_test_1ak_racemic.lcb

<Chromatogram>

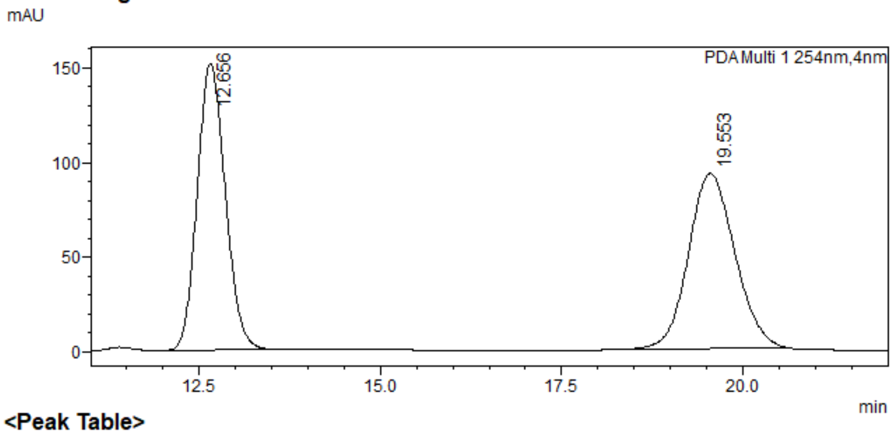

<Peak Table>

PDA Ch1 254nm

Peak#	Ret. Time	Area%
1	12.656	49.597
2	19.553	50.403
Total		100.000

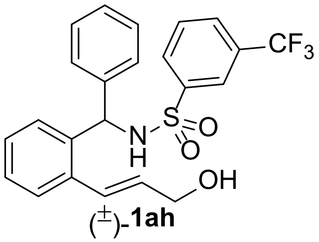

(±)-**1ah**

Chiral

BA catalyst: (*R*)-**CPA1**

Solvent: DCE

<Sample Information>

Data Filename : 240425_chiral_1akDCE3hr(S)_4252024_002.lcd
Method Filename : V2_H90_I10_R1_30min.lcm
Batch Filename : 240425_chiral_1akDCE3hr(S).lcb

<Chromatogram>

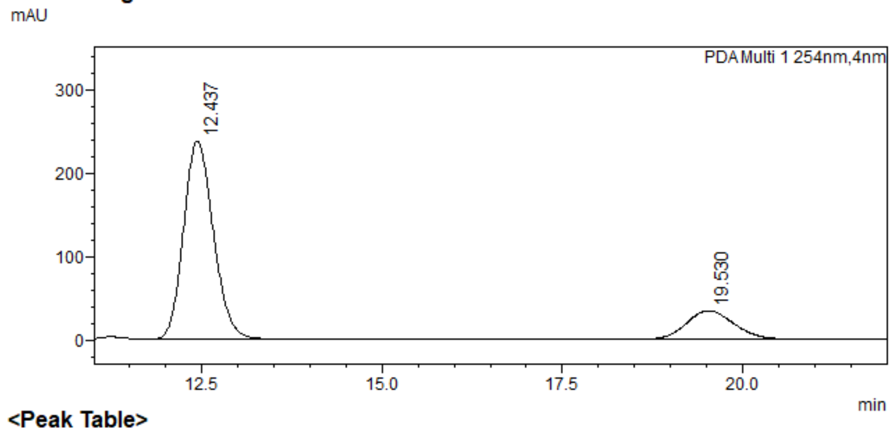

<Peak Table>

PDA Ch1 254nm

Peak#	Ret. Time	Area%
1	12.437	81.248
2	19.530	18.752
Total		100.000

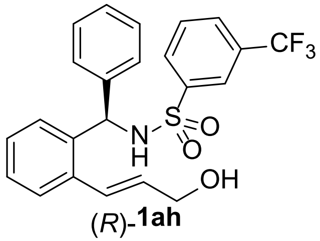

(*R*)-**1ah**

HPLC of **1bh**

Racemic

<Sample Information>

Data Filename : 240727_racemic_1wk(S)_7282024_002.lcd
Method Filename : V5_H88_I12_R1_30min.lcm
Batch Filename : 240727_racemic_1wk(S).lcb

<Chromatogram>

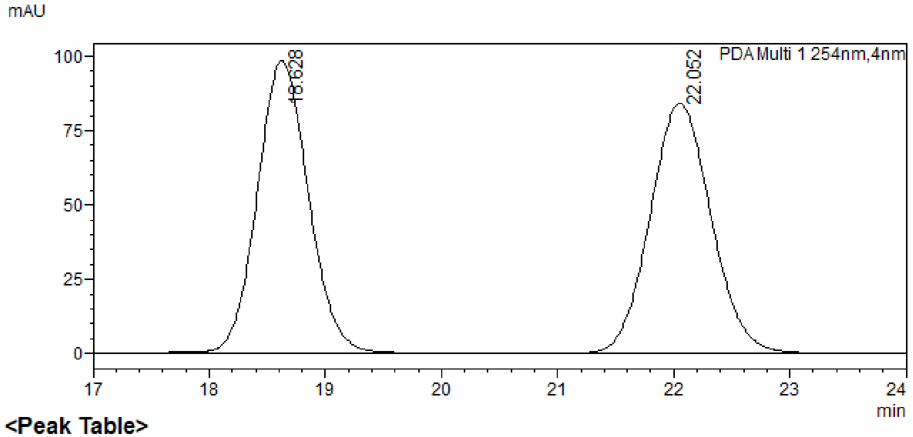

<Peak Table>

PDA Ch1 254nm

Peak#	Ret. Time	Area%
1	18.628	49.540
2	22.052	50.460
Total		100.000

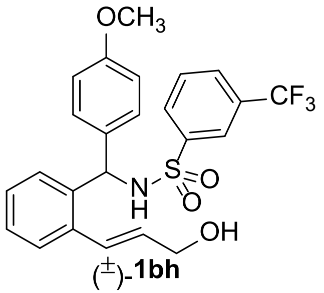

(±)-**1bh**

Chiral

BA catalyst: (*R*)-**CPA1**

Solvent: DCE

<Sample Information>

Data Filename : 240803_chiral_1wk_1h10m(S)_832024_002.lcd
Method Filename : V5_H88_I12_R1_30min.lcm
Batch Filename : 240803_chiral_1wk_1h10m(S).lcb

<Chromatogram>

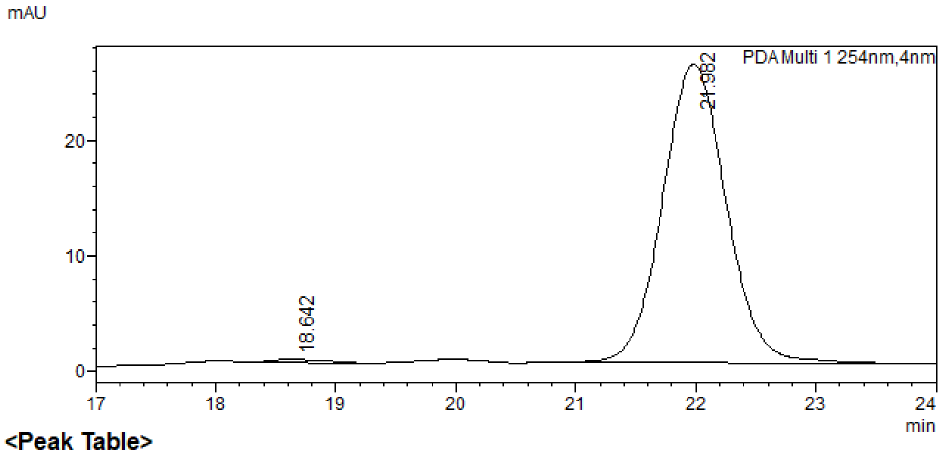

<Peak Table>

PDA Ch1 254nm

Peak#	Ret. Time	Area%
1	18.642	0.840
2	21.982	99.160
Total		100.000

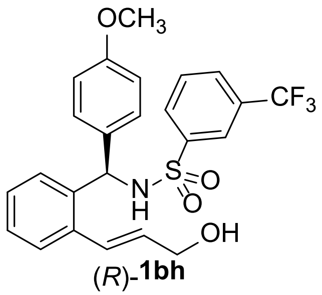

(*R*)-**1bh**

HPLC of **1ch**

Racemic

<Sample Information>

Data Filename : 240710_racemic_1tk(4-FPh)V5ADH8812_2rd_7102024_002.lcd
Method Filename : V5_H88_I12_R1_30min.lcm
Batch Filename : 240710_racemic_1tk(4-FPh)V5ADH8812_2rd.lcb

<Chromatogram>

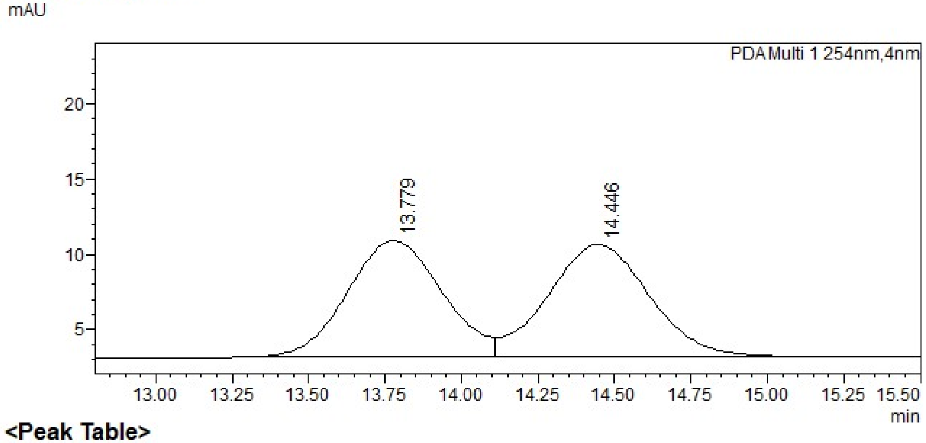

<Peak Table>

PDA Ch1 254nm

Peak#	Ret. Time	Area%
1	13.779	49.466
2	14.446	50.534
Total		100.000

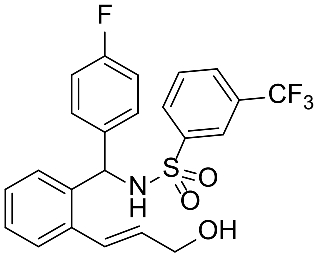

(±)-**1ch**

Chiral

BA catalyst: (*R*)-**CPA1**

Solvent: DCE

<Sample Information>

Data Filename : 240719_chiral_1tk_1h30m(S)_7192024_003.lcd
Method Filename : V5_H88_I12_R1_18min.lcm
Batch Filename : 240719_chiral_1tk_1h30m(S).lcb

<Chromatogram>

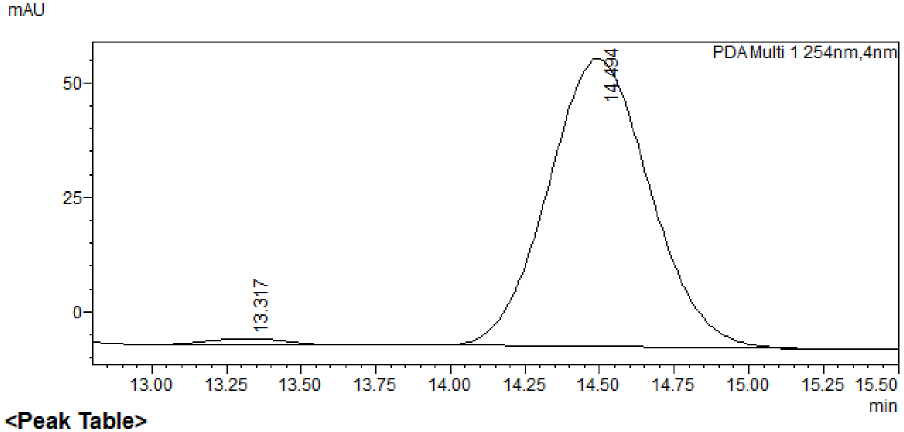

<Peak Table>

PDA Ch1 254nm

Peak#	Ret. Time	Area%
1	13.317	1.585
2	14.494	98.415
Total		100.000

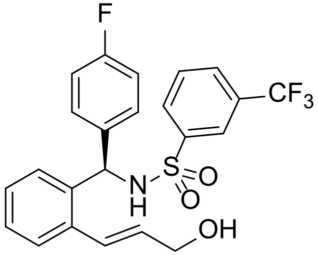

(*R*)-**1ch**

HPLC of **1dh**

Racemic

<Sample Information>

Data Filename : 240826_racemic_1zdk(4-CF3)_ADHV58812_8262024_002.lcd
Method Filename : V5_H88_I12_R1_30min.lcm
Batch Filename : 240826_racemic_1zdk(4-CF3)_ADHV58812.lcb

<Chromatogram>

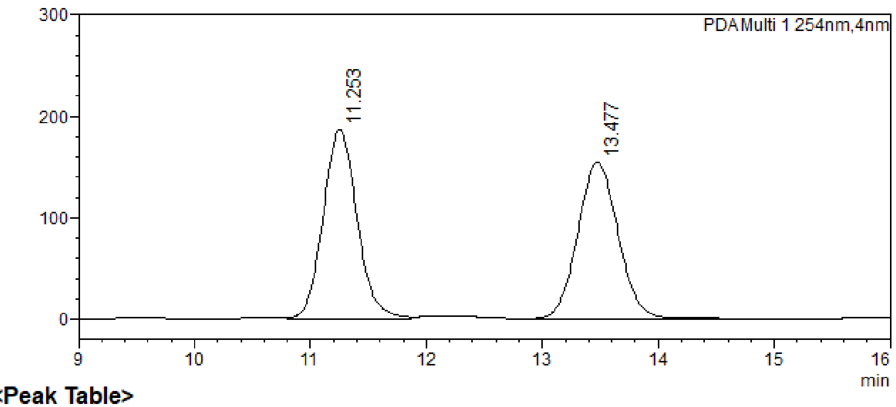

<Peak Table>

PDA Ch1 254nm

Peak#	Ret. Time	Area%
1	11.253	50.409
2	13.477	49.591
Total		100.000

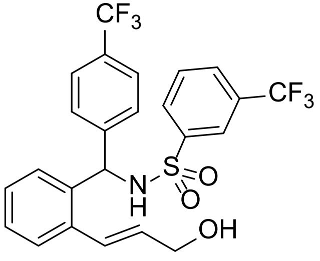

(±)-**1dh**

Chiral

BA catalyst: (*R*)-**CPA1**

Solvent: DCE

<Sample Information>

Data Filename : 240828_1zdk(4-CF3)_8m_30mg(S)_8292024_002.lcd
Method Filename : V5_H88_I12_R1_20min.lcm
Batch Filename : 240828_1zdk(4-CF3)_8m_30mg(S).lcb

<Chromatogram>

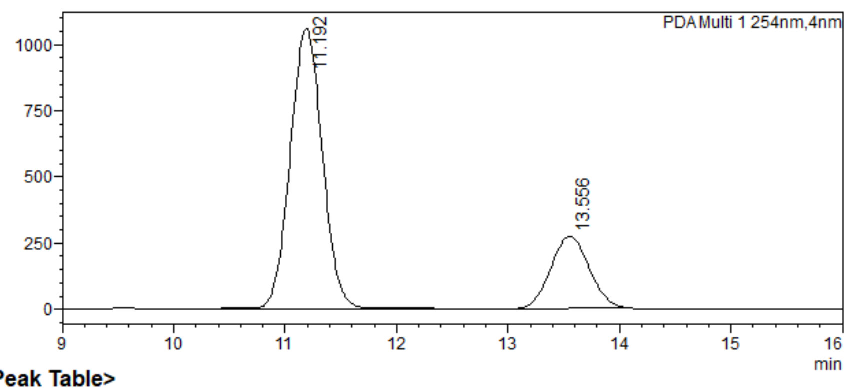

<Peak Table>

PDA Ch1 254nm

Peak#	Ret. Time	Area%
1	11.192	76.449
2	13.556	23.551
Total		100.000

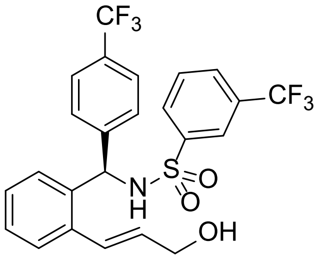

(*R*)-**1dh**

HPLC of **1eh**

Racemic

<Sample Information>

Data Filename : 240801_racemic_1yk(3-OMe)_V5ADH8812_812024_002.lcd
Method Filename : V5_H88_I12_R1_30min.lcm
Batch Filename : 240801_racemic_1yk(3-OMe)_V5ADH8812.lcb

<Chromatogram>

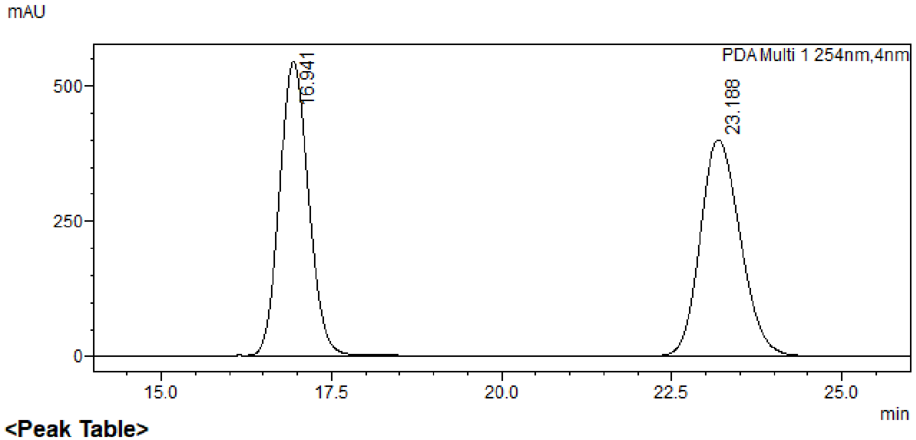

<Peak Table>

PDA Ch1 254nm

Peak#	Ret. Time	Area%
1	16.941	49.973
2	23.188	50.027
Total		100.000

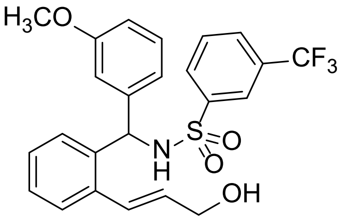

(±)-**1eh**

Chiral

BA catalyst: (*R*)-**CPA1**

Solvent: DCE

<Sample Information>

Data Filename : 240804_chiral_1yk_1h50m(S)_842024_002.lcd
Method Filename : V5_H88_I12_R1_30min.lcm
Batch Filename : 240804_chiral_1yk_1h50m(S).lcb

<Chromatogram>

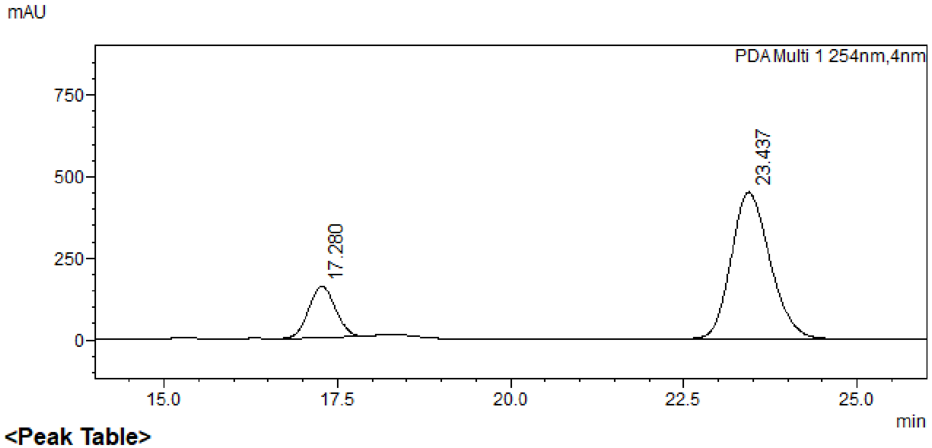

<Peak Table>

PDA Ch1 254nm

Peak#	Ret. Time	Area%
1	17.280	19.440
2	23.437	80.560
Total		100.000

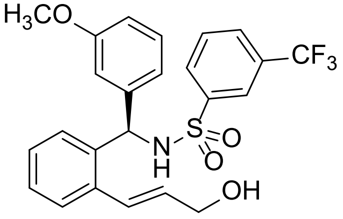

(*R*)-**1eh**

HPLC of **1fh**

Racemic

<Sample Information>

Data Filename : 240802_racemic_1zk(3-Me)_V5ADH8812_822024_002.lcd
Method Filename : V5_H88_I12_R1_30min.lcm
Batch Filename : 240802_racemic_1zk(3-Me)_V5ADH8812.lcb

<Chromatogram>

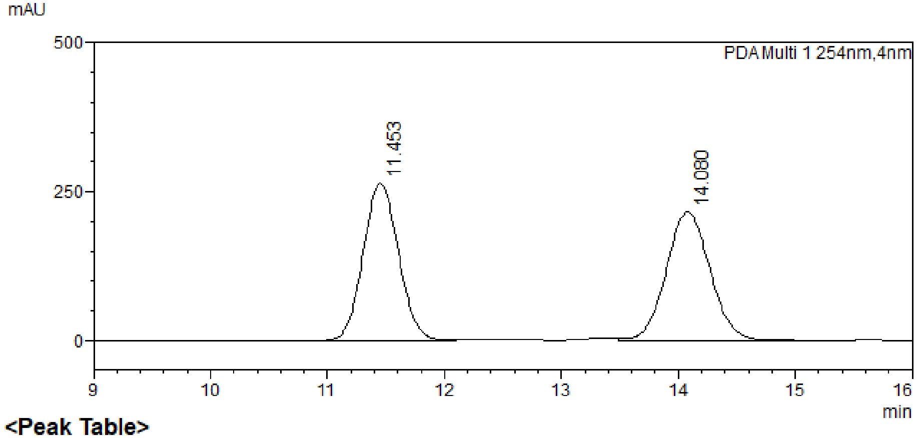

<Peak Table>

PDA Ch1 254nm

Peak#	Ret. Time	Area%
1	11.453	49.796
2	14.080	50.204
Total		100.000

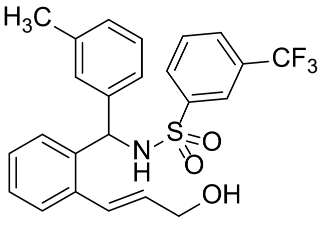

(±)-**1fh**

Chiral

BA catalyst: (*R*)-**CPA1**

Solvent: DCE

<Sample Information>

Data Filename : 240808_chiral_1Zk_3h(s)2rd_882024_002.lcd
Method Filename : V5_H88_I12_R1_30min.lcm
Batch Filename : 240808_chiral_1Zk_3h(s)2rd.lcb

<Chromatogram>

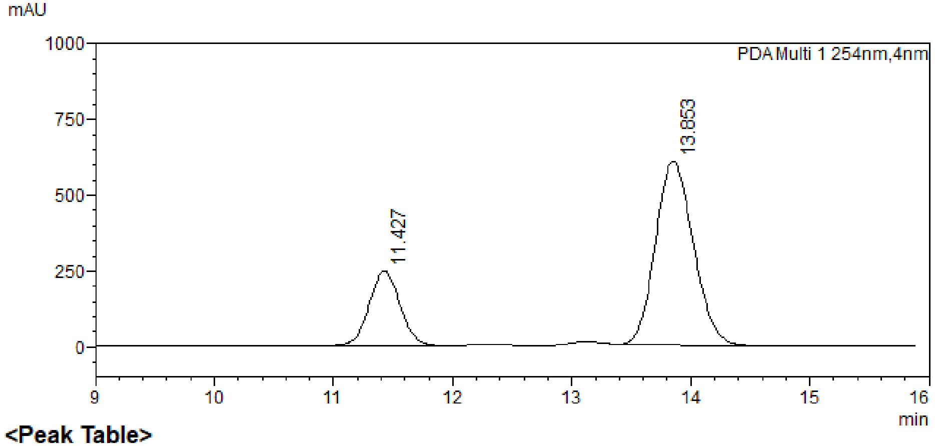

<Peak Table>

PDA Ch1 254nm

Peak#	Ret. Time	Area%
1	11.427	25.862
2	13.853	74.138
Total		100.000

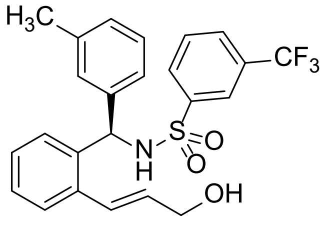

(*R*)-**1fh**

HPLC of **1gh**

Racemic

<Sample Information>

Data Filename : 240718_racemic_1vk(35CF3)_V2ODH9406_7182024_002.lcd
Method Filename : V2_H94_I06_R1_30min.lcm
Batch Filename : 240718_racemic_1vk(35CF3)_V2ODH9406.lcb

<Chromatogram>

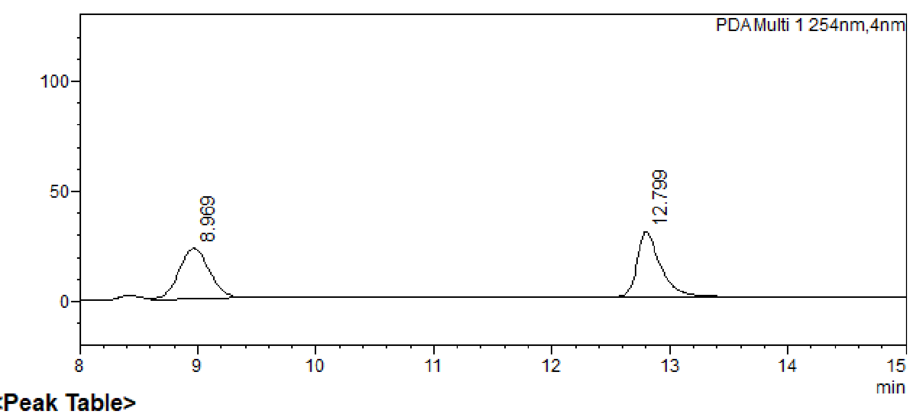

<Peak Table>

PDA Ch1 254nm

Peak#	Ret. Time	Area%
1	8.969	49.627
2	12.799	50.373
Total		100.000

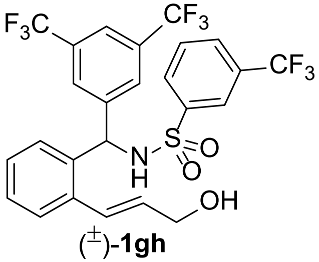

(±)-**1gh**

Chiral

BA catalyst: (*R*)-**CPA1**

Solvent: DCE

<Sample Information>

Data Filename : 240720_chiral_1vk_10min(S)_7202024_002.lcd
Method Filename : V2_H94_I06_R1_18min.lcm
Batch Filename : 240720_chiral_1vk_10min(S).lcb

<Chromatogram>

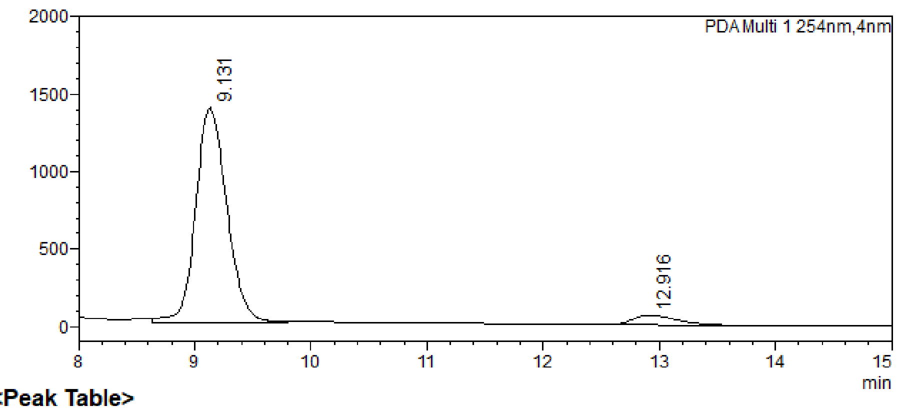

<Peak Table>

PDA Ch1 254nm

Peak#	Ret. Time	Area%
1	9.131	94.147
2	12.916	5.853
Total		100.000

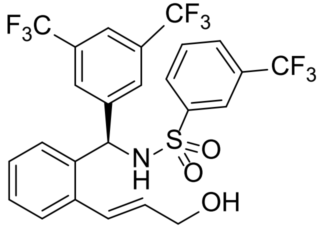

(*R*)-**1gh**

HPLC of **1hh**

Racemic

<Sample Information>

Data Filename : 240809_racemic_1zak(acetal)_ADH98_2rd_892024_002.lcd
Method Filename : V5_H88_I12_R1_45min.lcm
Batch Filename : 240809_racemic_1zak(acetal)_ADH98_2rd.lcb

<Chromatogram>

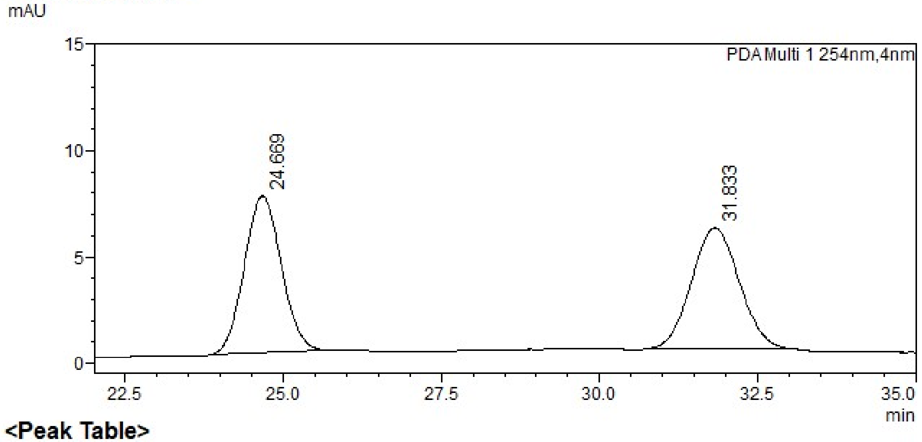

<Peak Table>

PDA Ch1 254nm

Peak#	Ret. Time	Area%
1	24.669	50.174
2	31.833	49.826
Total		100.000

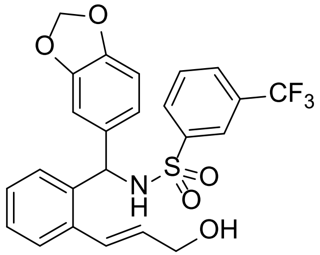

(±)-**1hh**

Chiral

BA catalyst: (*R*)-**CPA1**

Solvent: DCE

<Sample Information>

Data Filename : 240813_chiral_1zak(13benzodioxle)_2h35m(S)_8132024_002.lcd
Method Filename : V5_H88_I12_R1_45min.lcm
Batch Filename : 240813_chiral_1zak(13benzodioxle)_2h35m(S).lcb

<Chromatogram>

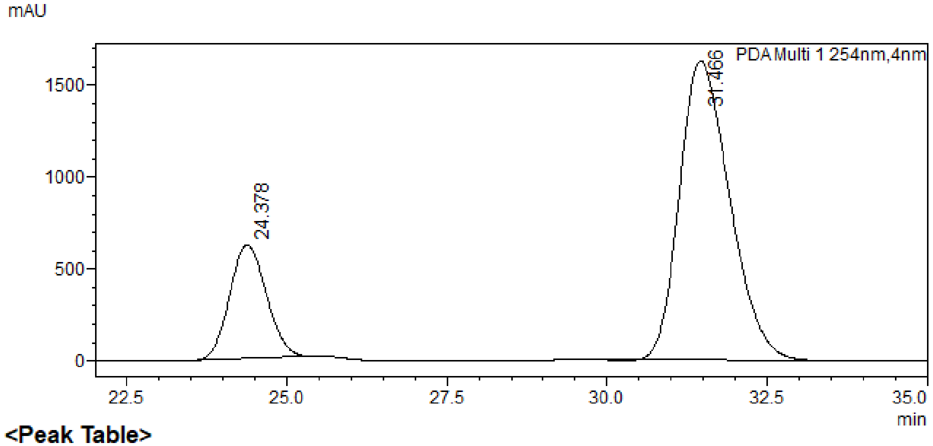

<Peak Table>

PDA Ch1 254nm

Peak#	Ret. Time	Area%
1	24.378	21.512
2	31.466	78.488
Total		100.000

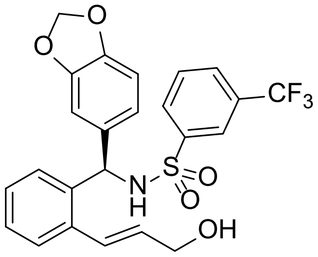

(*R*)-**1hh**

HPLC of **1ih**

Racemic

<Sample Information>

Data Filename : 240729_racemic_1xk(2-naph)_7292024_002.lcd
Method Filename : V5_H88_I12_R1_30min.lcm
Batch Filename : 240729_racemic_1xk(2-naph).lcb

<Chromatogram>

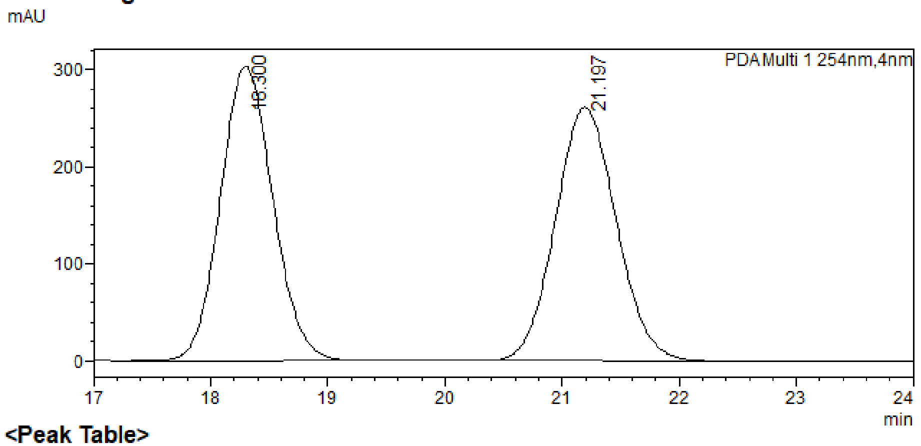

<Peak Table>

PDA Ch1 254nm

Peak#	Ret. Time	Area%
1	18.300	50.048
2	21.197	49.952
Total		100.000

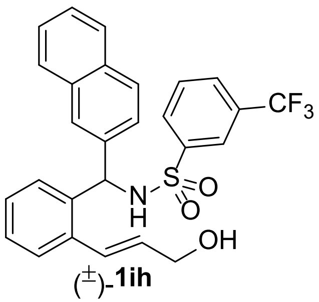

(±)-**1ih**

Chiral

BA catalyst: (*R*)-**CPA1**

Solvent: DCE

<Sample Information>

Data Filename : 240801_chiral_1xk_1h40m(S)_812024_002.lcd
Method Filename : V5_H88_I12_R1_30min.lcm
Batch Filename : 240801_chiral_1xk_1h40m(S).lcb

<Chromatogram>

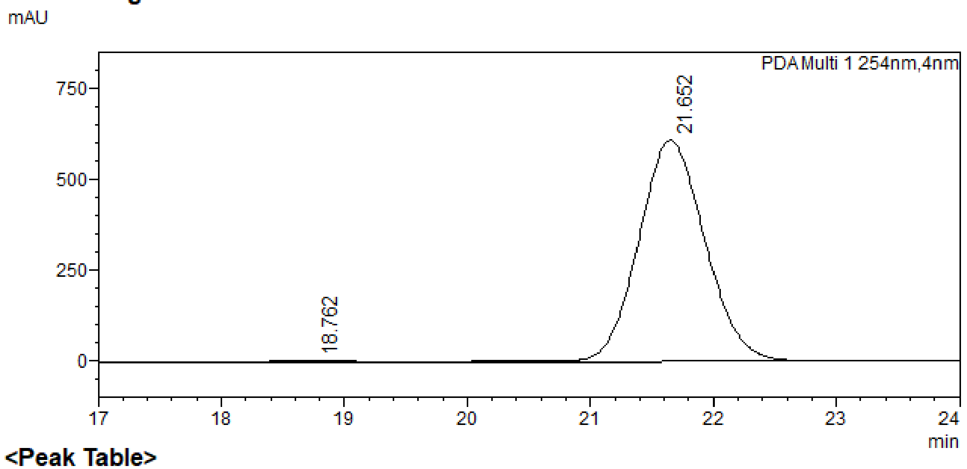

<Peak Table>

PDA Ch1 254nm

Peak#	Ret. Time	Area%
1	18.762	0.466
2	21.652	99.534
Total		100.000

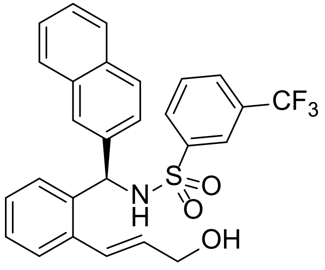

(*R*)-**1ih**

HPLC of **1jh**

Racemic

<Sample Information>

Data Filename : 240824_racemic_1zck(S) -3 H85 I15_8242024_002.lcd
Method Filename : V5_H85_I15_R1_30min.lcm
Batch Filename : 240824_racemic_1zck(S) -3 H85 I15.lcb

<Chromatogram>

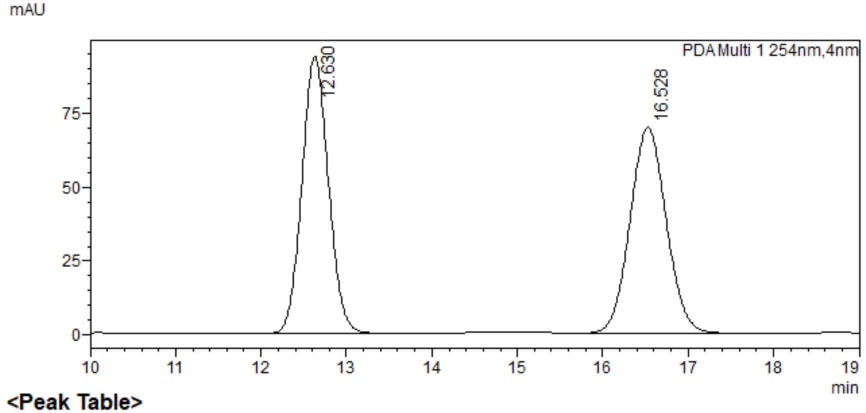

<Peak Table>

PDA Ch1 254nm

Peak#	Ret. Time	Area%
1	12.630	49.629
2	16.528	50.371
Total		100.000

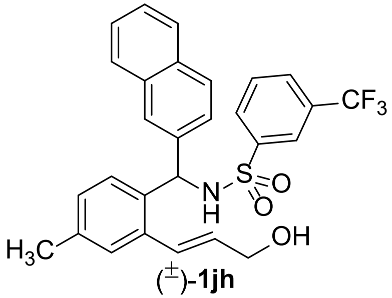

(±)-**1jh**

Chiral

BA catalyst: (*R*)-**CPA1**

Solvent: DCE

<Sample Information>

Data Filename : 240828_chiral_1zck 30mg 20min H85 I15_8282024_002.lcd
Method Filename : V5_H85_I15_R1_30min.lcm
Batch Filename : 240828_chiral_1zck 30mg 20min H85 I15.lcb

<Chromatogram>

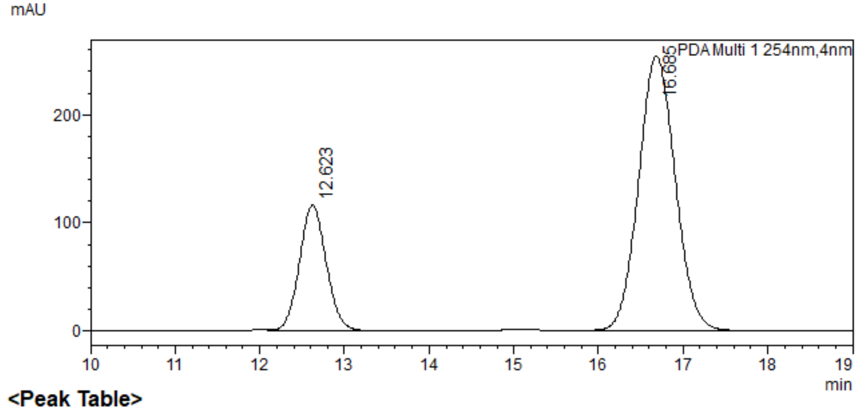

<Peak Table>

PDA Ch1 254nm

Peak#	Ret. Time	Area%
1	12.623	25.085
2	16.685	74.915
Total		100.000

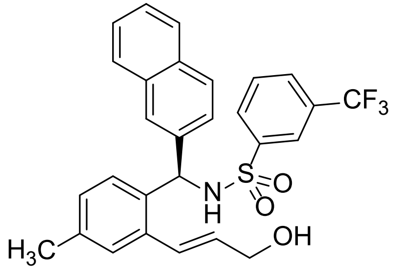

(*R*)-**1jh**

HPLC of **1kh**

Racemic

<Sample Information>

Data Filename : 240910_racemic_1zek(5-F2-NAPH)_V58812_9102024_002.lcd
Method Filename : V5_H88_I12_R1_30min.lcm
Batch Filename : 240910_racemic_1zek(5-F2-NAPH)_V58812.lcb

<Chromatogram>

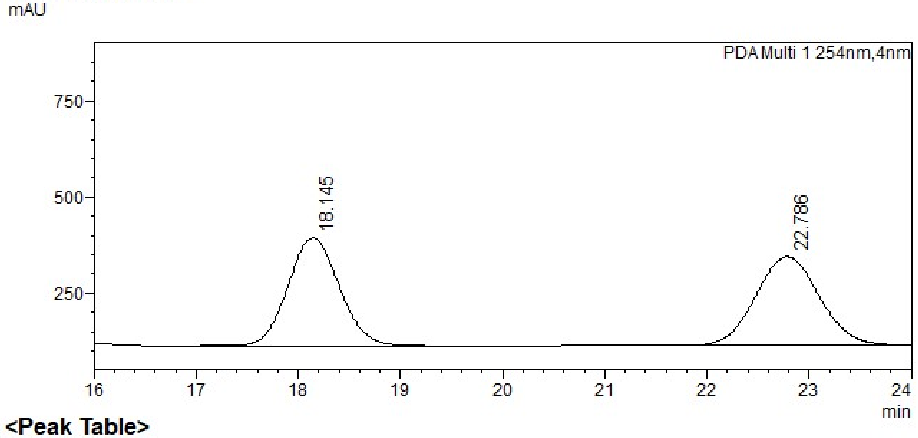

<Peak Table>

PDA Ch1 254nm

Peak#	Ret. Time	Area%
1	18.145	49.774
2	22.786	50.226
Total		100.000

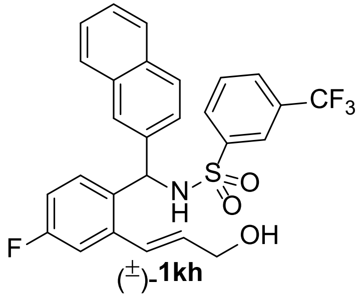

(±)-**1kh**

Chiral

BA catalyst: (*R*)-**CPA1**

Solvent: DCE

<Sample Information>

Data Filename : 250113-1_2025113_002.lcd
Method Filename : V5_H88_I12_R1_30min.lcm
Batch Filename : 250113-1.lcb

<Chromatogram>

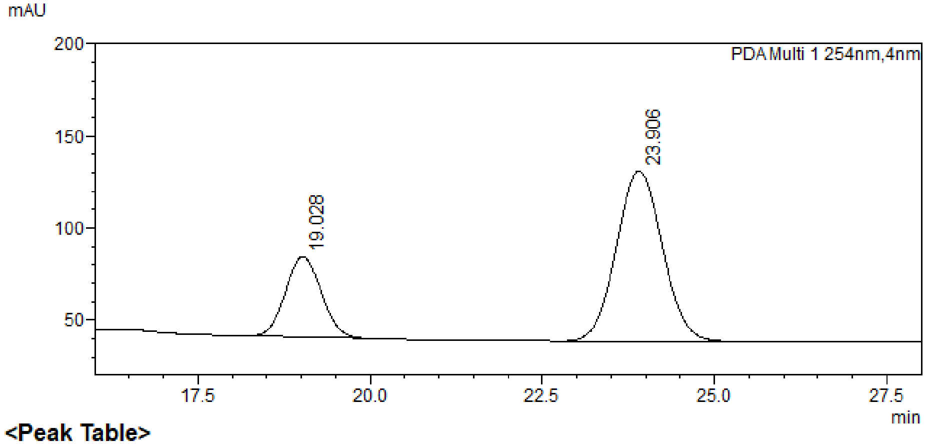

<Peak Table>

PDA Ch1 254nm

Peak#	Ret. Time	Area%
1	19.028	26.967
2	23.906	73.033
Total		100.000

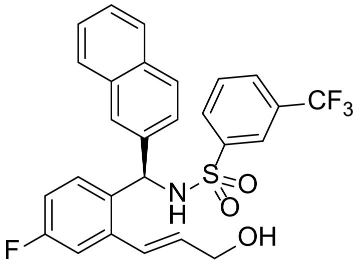

(*R*)-**1kh**

HPLC of **1lh**

Racemic

<Sample Information>

Data Filename : 240622_racemic_1bk_V5ADH8812_6222024_002.lcd
Method Filename : V5_H88_I12_R1_30min.lcm
Batch Filename : 240622_racemic_1bk_V5ADH8812.lcb

<Chromatogram>

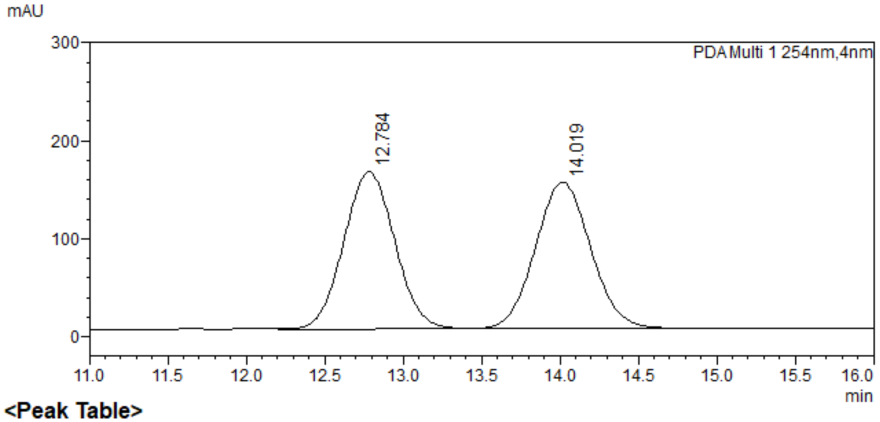

<Peak Table>

PDA Ch1 254nm

Peak#	Ret. Time	Area%
1	12.784	49.691
2	14.019	50.309
Total		100.000

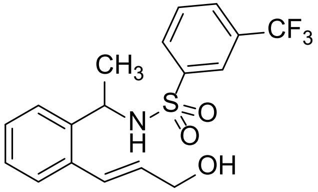

(±)-**1lh**

Chiral

BA catalyst: (*R*)-**CPA1**

Solvent: DCE

<Sample Information>

Data Filename : 240624_chiral_1bk_DCECPA6_2h5m(S)_6242024_002.lcd
Method Filename : V5_H88_I12_R1_30min.lcm
Batch Filename : 240624_chiral_1bk_DCECPA6_2h5m(S).lcb

<Chromatogram>

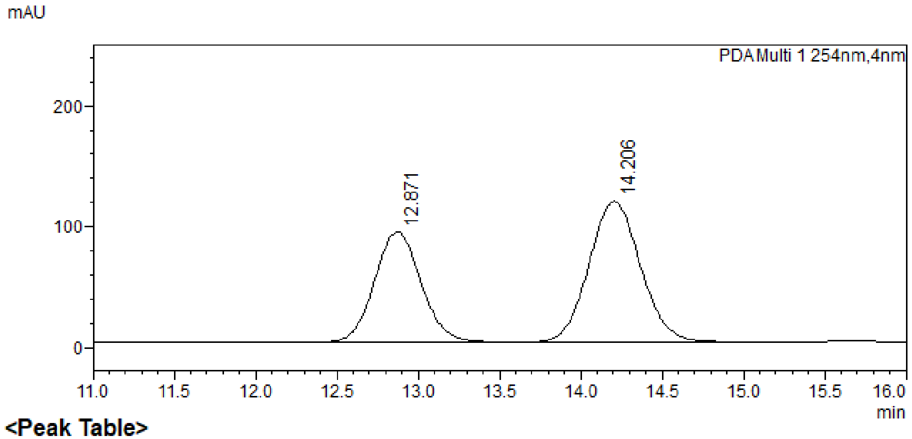

<Peak Table>

PDA Ch1 254nm

Peak#	Ret. Time	Area%
1	12.871	41.444
2	14.206	58.556
Total		100.000

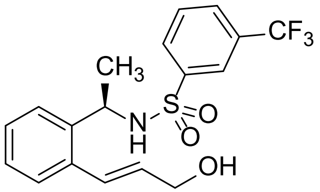

(*R*)-**1lh**

HPLC of **2aa**

Racemic
BA catalyst: DPP
Solvent: DCE

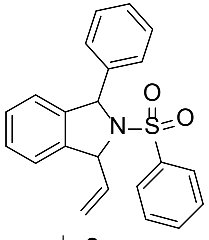

(±)-**2aa**

<Sample Information>

Data Filename : 240415_rac2aa_adh95_OJ94_IC92_4152024_008.lcd
Method Filename : V4_H92_I08_R1_45min.lcm
Batch Filename : 240415_rac2aa_adh95_OJ94_IC92.lcb

<Chromatogram>

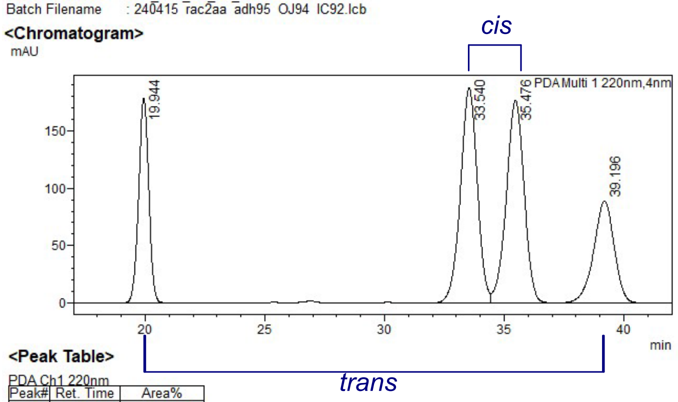

<Peak Table>

PDA Ch1 220nm

Peak#	Ret. Time	Area%
1	19.944	18.202
2	33.540	31.834
3	35.476	31.685
4	39.196	18.279
Total		100.000

Chiral
BA catalyst: (*R*)-**CPA1**
Solvent: DCE

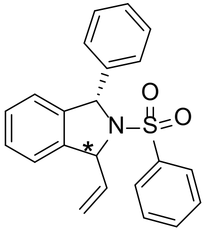

2aa, 1:1.2 dr

<Sample Information>

Data Filename : 240423_chiral_1aaDCE2h10m(P)_4232024_002.lcd
Method Filename : V4_H92_I08_R1_45min.lcm
Batch Filename : 240423_chiral_1aaDCE2h10m(P).lcb

<Chromatogram>

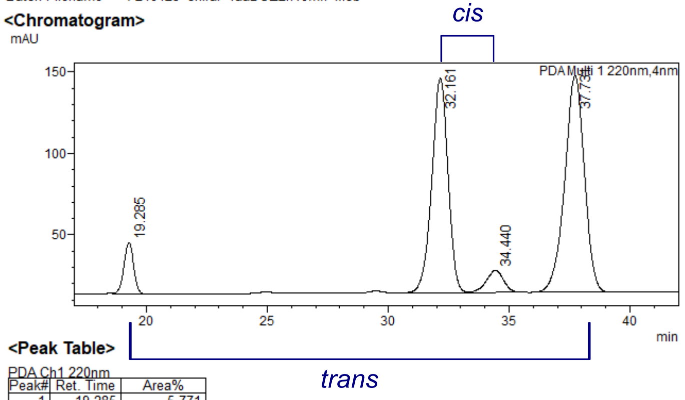

<Peak Table>

PDA Ch1 220nm

Peak#	Ret. Time	Area%
1	19.285	5.771
2	32.161	40.349
3	34.440	4.408
4	37.731	49.472
Total		100.000

Authentic
Compound

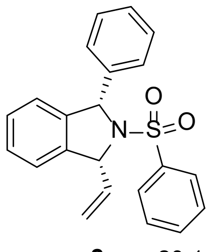

(1*S*,3*R*)-**2aa**, >20:1 dr
synthesized from **9a**

<Sample Information>

Data Filename : 241222-1_20241222_002.lcd
Method Filename : V4_H92_I08_R1_45min.lcm
Batch Filename : 241222-1.lcb

<Chromatogram>

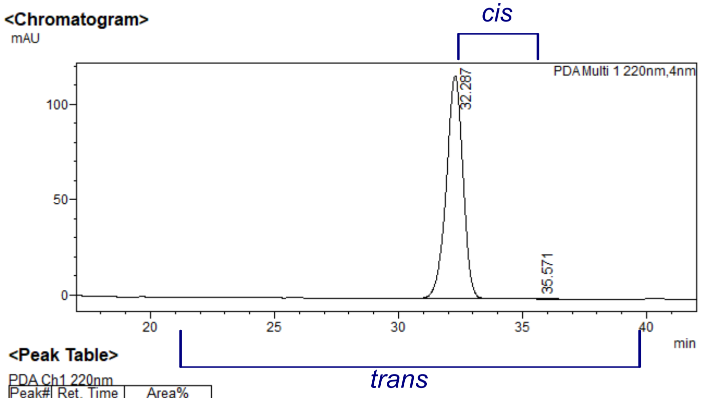

<Peak Table>

PDA Ch1 220nm

Peak#	Ret. Time	Area%
1	32.287	99.391
2	35.571	0.609
Total		100.000

Recrystallization
from **2aa**

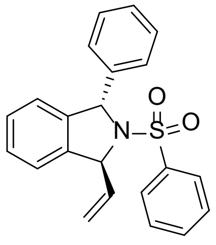

(1*S*,3*S*)-**2aa**, 1:>20 dr

<Sample Information>

Data Filename : 250113_2025113_002.lcd
Method Filename : V4_H92_I08_R1_45min.lcm
Batch Filename : 250113.lcb

<Chromatogram>

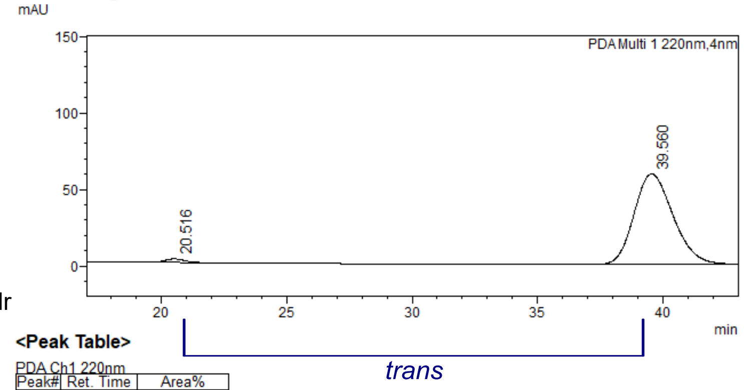

<Peak Table>

PDA Ch1 220nm

Peak#	Ret. Time	Area%
1	20.516	1.888
2	39.560	98.112
Total		100.000

HPLC of **2ab**

Racemic

BA catalyst: DPP

Solvent: DCE

<Sample Information>

Data Filename : 240514_racemic_2af_V5ADH9802_5142024_002.lcd
Method Filename : V5_H98_I02_R1_60min.lcm
Batch Filename : 240514_racemic_2af_V5ADH9802.lcb

<Chromatogram>

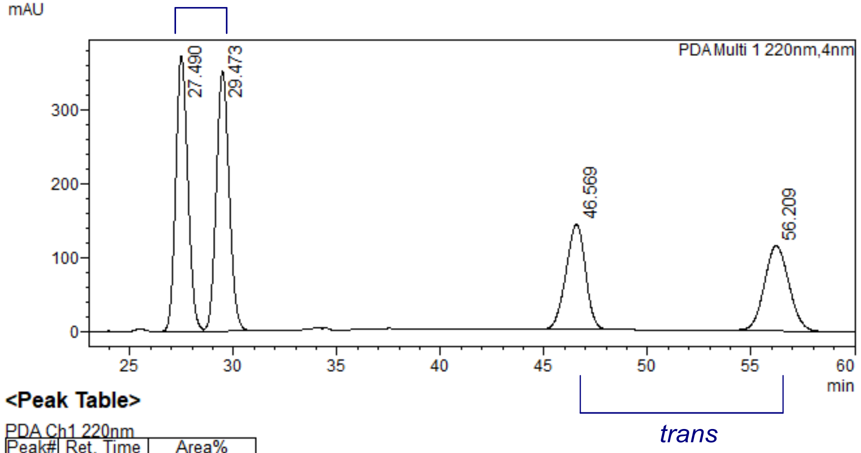

<Peak Table>

PDA Ch1 220nm

Peak#	Ret. Time	Area%
1	27.490	30.852
2	29.473	30.906
3	46.569	19.019
4	56.209	19.224
Total		100.000

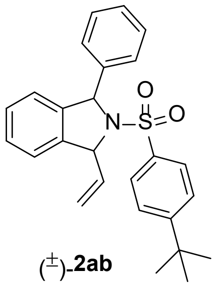

(±)-**2ab**

Chiral

BA catalyst: (*R*)-**CPA1**

Solvent: DCE

<Sample Information>

Data Filename : 240513_chiral_1af(4tbu)DCECPA6_6h15m(P)_5132024_002.lcd
Method Filename : V5_H98_I02_R1_60min.lcm
Batch Filename : 240513_chiral_1af(4tbu)DCECPA6_6h15m(P).lcb

<Chromatogram>

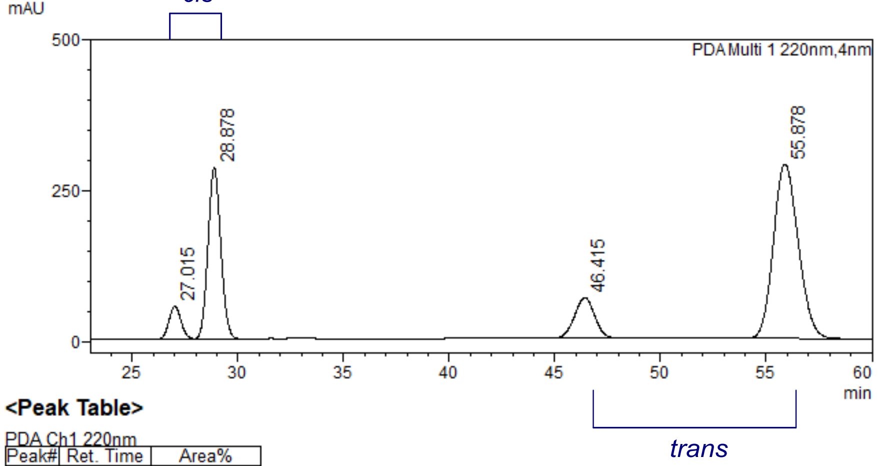

<Peak Table>

PDA Ch1 220nm

Peak#	Ret. Time	Area%
1	27.015	5.226
2	28.878	28.442
3	46.415	10.476
4	55.878	55.855
Total		100.000

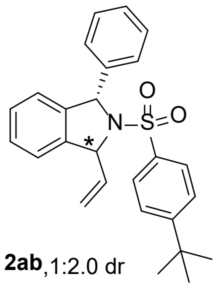

2ab,1:2.0 dr

HPLC of **2ac**

Racemic
BA catalyst: DPP
Solvent: DCE

<Sample Information>

Data Filename : 230614_test_4-fracemic(p)_6142023_005.lcd
Method Filename : V2_H98_I02_R1_30min.lcm
Batch Filename : 230614_test_4-fracemic(p).lcb

<Chromatogram>

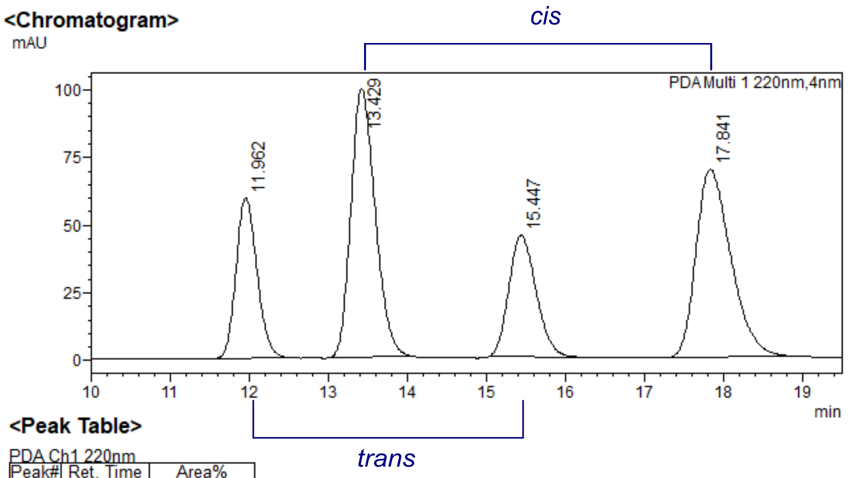

<Peak Table>

PDA Ch1 220nm

Peak#	Ret. Time	Area%
1	11.962	16.981
2	13.429	33.081
3	15.447	16.773
4	17.841	33.165
Total		100.000

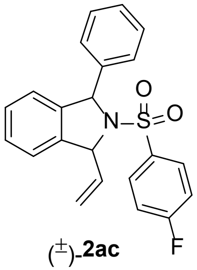

(±)-**2ac**

Chiral
BA catalyst: (*R*)-**CPA1**
Solvent: DCE

<Sample Information>

Data Filename : 240508_chiral_1ah(4-F)DCECPA6_4h30m(P)_582024_002.lcd
Method Filename : V2_H98_I02_R1_30min.lcm
Batch Filename : 240508_chiral_1ah(4-F)DCECPA6_4h30m(P).lcb

<Chromatogram>

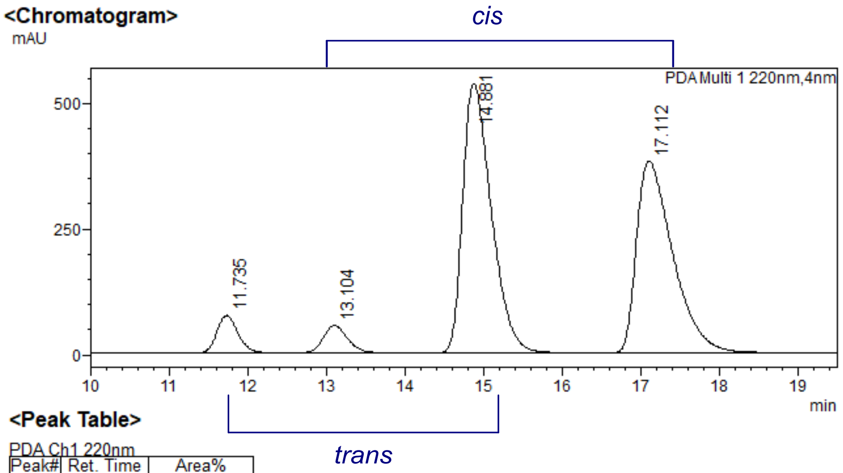

<Peak Table>

PDA Ch1 220nm

Peak#	Ret. Time	Area%
1	11.735	4.951
2	13.104	4.181
3	14.881	47.851
4	17.112	43.017
Total		100.000

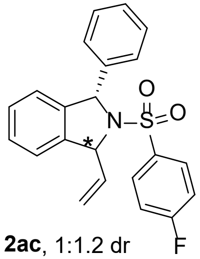

2ac, 1:1.2 dr

HPLC of **2ad**

Racemic

BA catalyst: DPP

Solvent: DCE

<Sample Information>

Data Filename : 240528_racemic_2ai(4-CF3)adh95_oj94_ic92_odh97_5282024_011.lcd
Method Filename : V2_H97_I03_R1_45min.lcm
Batch Filename : 240528_racemic_2ai(4-CF3)adh95_oj94_ic92_odh97.lcb

<Chromatogram>

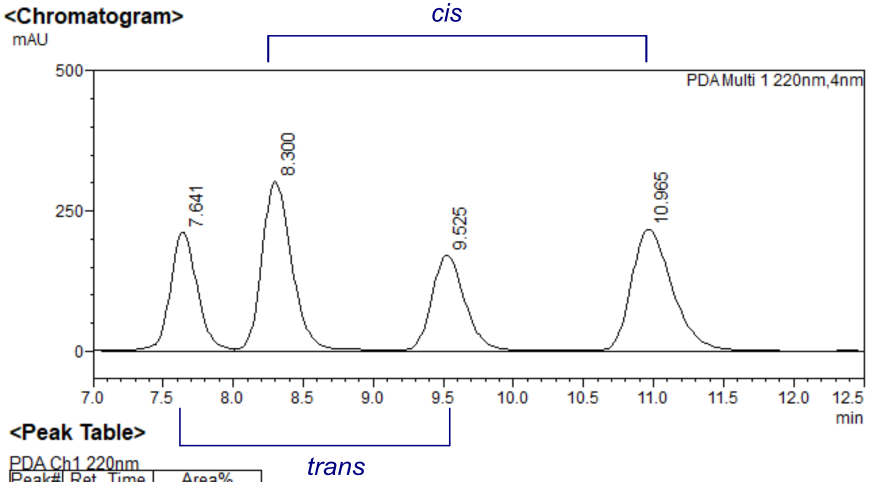

<Peak Table>

PDA Ch1 220nm

Peak#	Ret. Time	Area%
1	7.641	19.484
2	8.300	30.760
3	9.525	19.398
4	10.965	30.358
Total		100.000

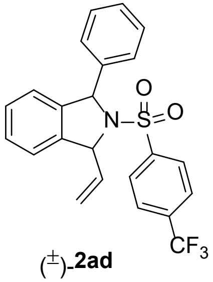

(±)-**2ad**

Chiral

BA catalyst: (*R*)-**CPA1**

Solvent: DCE

<Sample Information>

Data Filename : 240528_chiral_1ai(4-CF3)_DCECPA6_1h55m(P)_5282024_002.lcd
Method Filename : V2_H97_I03_R1_20min.lcm
Batch Filename : 240528_chiral_1ai(4-CF3)_DCECPA6_1h55m(P).lcb

<Chromatogram>

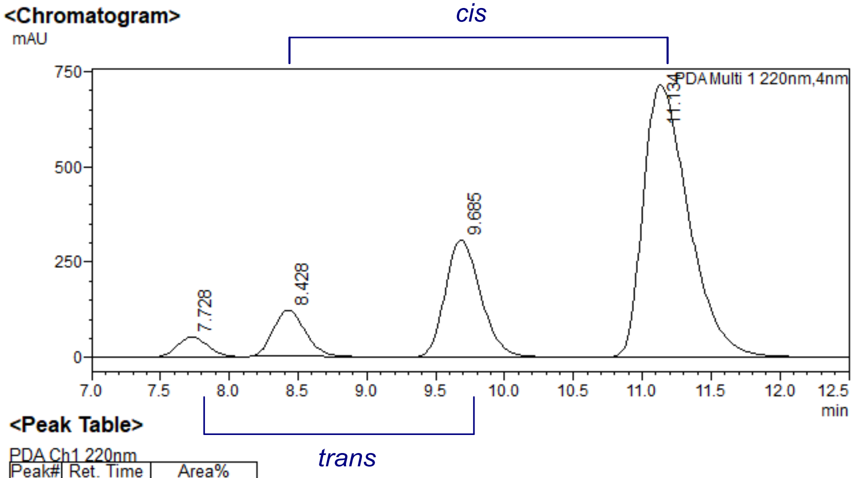

<Peak Table>

PDA Ch1 220nm

Peak#	Ret. Time	Area%
1	7.728	3.254
2	8.428	8.116
3	9.685	22.282
4	11.134	66.348
Total		100.000

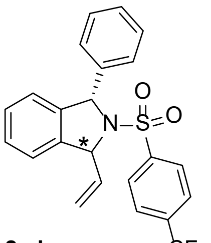

2ad,1.2:1 dr

HPLC of **2ae**

Racemic

BA catalyst: DPP

Solvent: DCE

<Sample Information>

Data Filename : 240417_rac2ac_adh95_80min_4172024_002.lcd
Method Filename : V5_H95_I05_R1_80min.lcm
Batch Filename : 240417_rac2ac_adh95_80min.lcb

<Chromatogram>

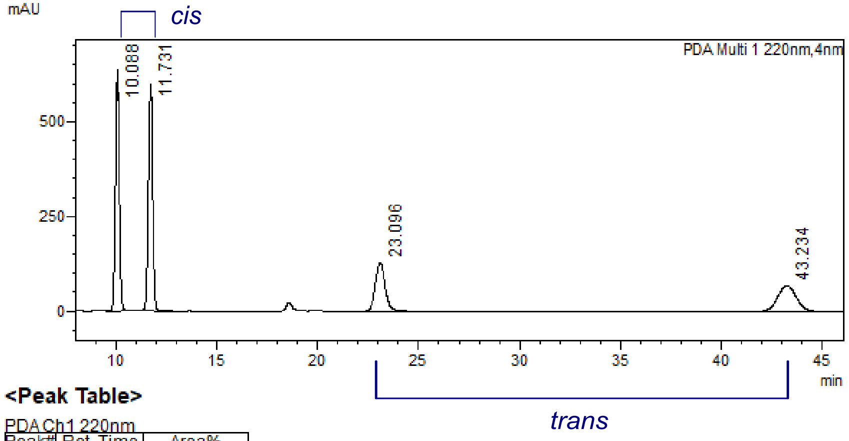

<Peak Table>

PDA Ch1 220nm

Peak#	Ret. Time	Area%
1	10.088	32.925
2	11.731	33.533
3	23.096	16.749
4	43.234	16.793
Total		100.000

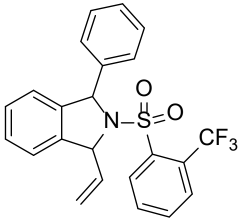

(±)-**2ae**

Chiral

BA catalyst: (*R*)-**CPA1**

Solvent: DCE

<Sample Information>

Data Filename : 240924_chiral_2ac_adh95_60min_9242024_002.lcd
Method Filename : V5_H95_I05_R1_60min.lcm
Batch Filename : 240924_chiral_2ac_adh95_60min.lcb

<Chromatogram>

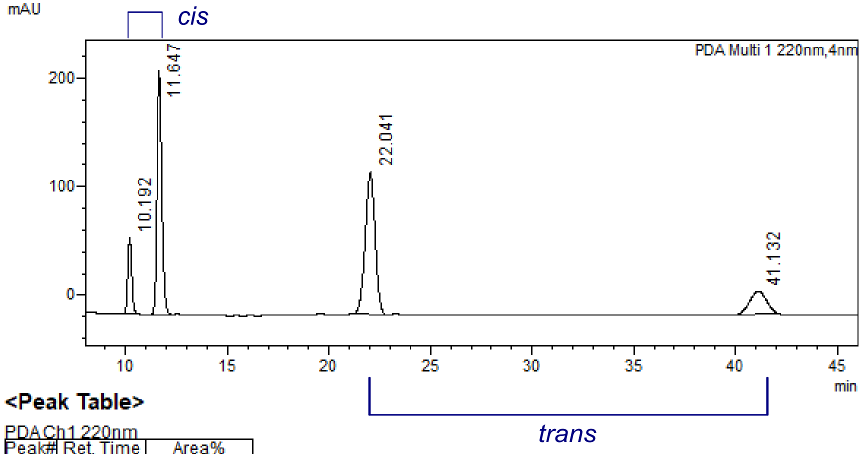

<Peak Table>

PDA Ch1 220nm

Peak#	Ret. Time	Area%
1	10.192	8.984
2	11.647	36.220
3	22.041	43.451
4	41.132	11.344
Total		100.000

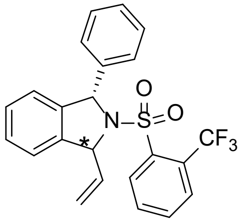

2ae, 1:1.2 dr

HPLC of **2af**

Racemic

BA catalyst: DPP

Solvent: DCE

<Sample Information>

Data Filename : 240415_racemic2AB_ADH95_4152024_002.lcd
Method Filename : V5_H95_I05_R1_40min.lcm
Batch Filename : 240415_racemic2AB_ADH95.lcb

<Chromatogram>

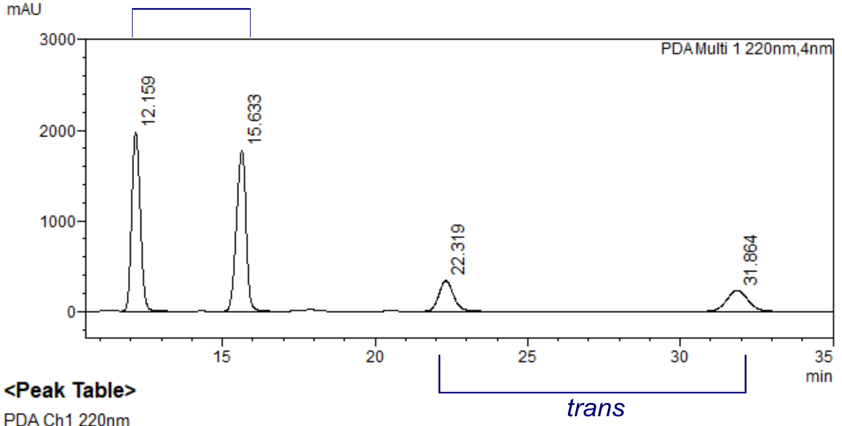

<Peak Table>

PDA Ch1 220nm

Peak#	Ret. Time	Area%
1	12.159	38.441
2	15.633	39.267
3	22.319	11.156
4	31.864	11.136
Total		100.000

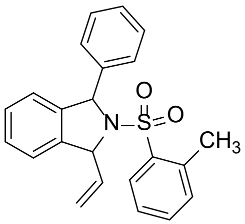

(±)-**2af**

Chiral

BA catalyst: (*R*)-**CPA1**

Solvent: DCE

<Sample Information>

Data Filename : 240516_1ab_DCErt_CPA6_4h40m30mg(P)3rd_5162024_002.lcd
Method Filename : V5_H95_I05_R1_40min.lcm
Batch Filename : 240516_1ab_DCErt_CPA6_4h40m30mg(P)3rd.lcb

<Chromatogram>

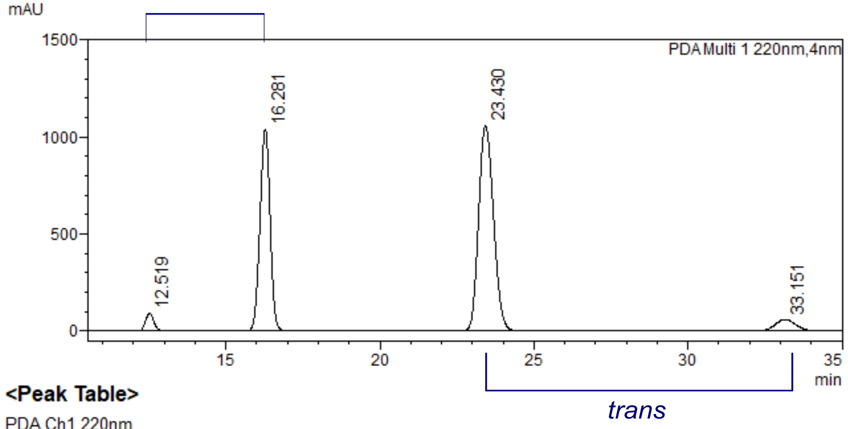

<Peak Table>

PDA Ch1 220nm

Peak#	Ret. Time	Area%
1	12.519	2.684
2	16.281	37.074
3	23.430	55.787
4	33.151	4.454
Total		100.000

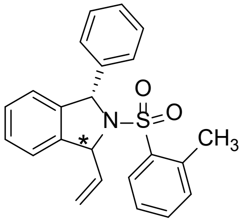

2af, 1:2.0 dr

HPLC of **2ah**

Racemic

BA catalyst: DPP

Solvent: toluene

<Sample Information>

Data Filename : 230722_2ak_racemic_3rd_7222023_005.lcd
Method Filename : V2_H98_I02_R0.8_45min.lcm
Batch Filename : 230722_2ak_racemic_3rd.lcb

<Chromatogram>

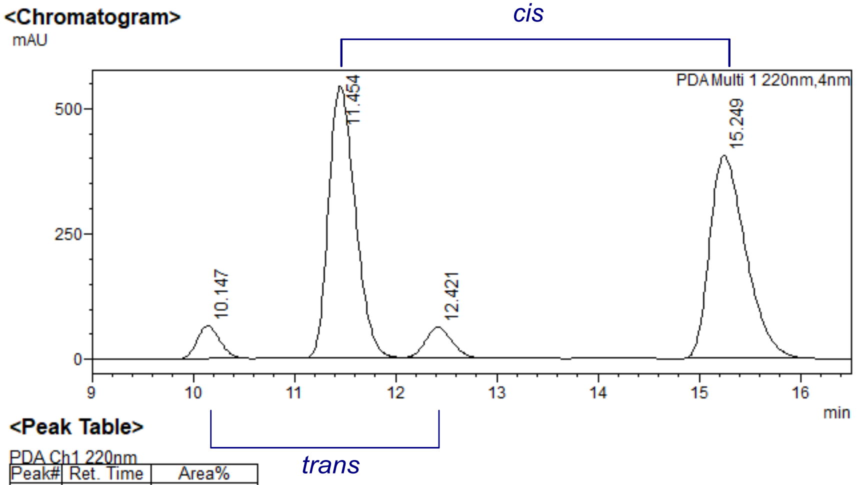

<Peak Table>

PDA Ch1 220nm

Peak#	Ret. Time	Area%
1	10.147	4.510
2	11.454	44.974
3	12.421	4.913
4	15.249	45.603
Total		100.000

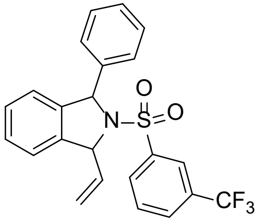

(±)-**2ah**

Chiral

BA catalyst: (*R*)-**CPA1**

Solvent: DCE

<Sample Information>

Data Filename : 240425_chiral_1akDCE3hr(P)_4252024_002.lcd
Method Filename : V2_H98_I02_R0.8_20min.lcm
Batch Filename : 240425_chiral_1akDCE3hr(P).lcb

<Chromatogram>

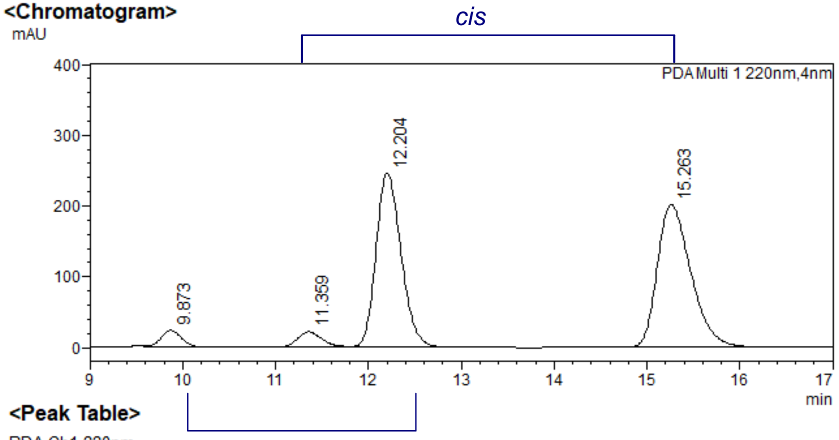

<Peak Table>

PDA Ch1 220nm

Peak#	Ret. Time	Area%
1	9.873	3.471
2	11.359	3.999
3	12.204	44.204
4	15.263	48.325
Total		100.000

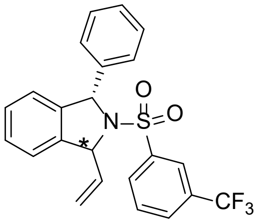

2ah,1.2:1 dr

HPLC of **2bh**

BA catalyst: DPP

Solvent: DCE

<Sample Information>

Data Filename : 240727_racemic_2wk_ADH95_7302024_002.lcd
Method Filename : V5_H95_I05_R1_30min.lcm
Batch Filename : 240727_racemic_2wk_ADH95.lcb

<Chromatogram>

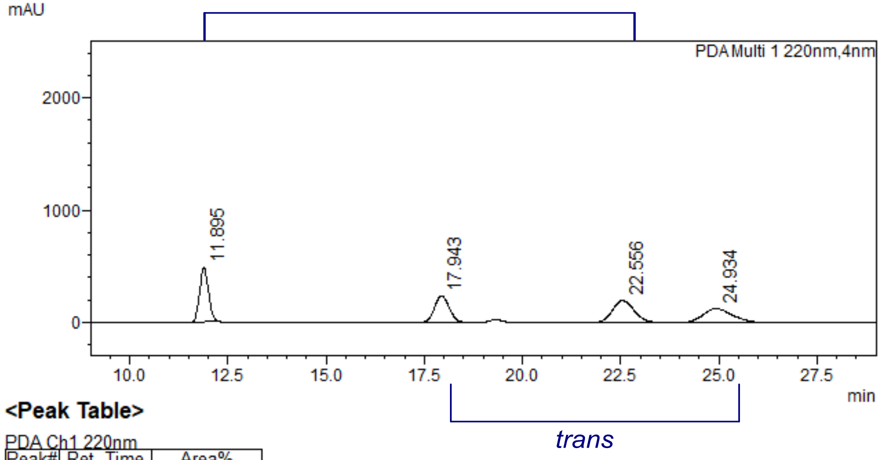

<Peak Table>

PDA Ch1 220nm

Peak#	Ret. Time	Area%
1	11.895	27.127
2	17.943	22.433
3	22.556	27.152
4	24.934	23.288
Total		100.000

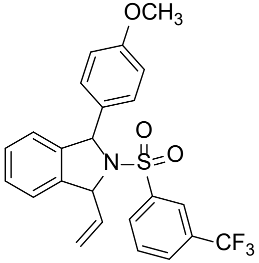

(±)-**2bh**

Chiral

BA catalyst: (*R*)-**CPA1**

Solvent: DCE

<Sample Information>

Data Filename : 240803_chiral_1wk_1h10m(P)_832024_002.lcd
Method Filename : V5_H95_I05_R1_30min.lcm
Batch Filename : 240803_chiral_1wk_1h10m(P).lcb

<Chromatogram>

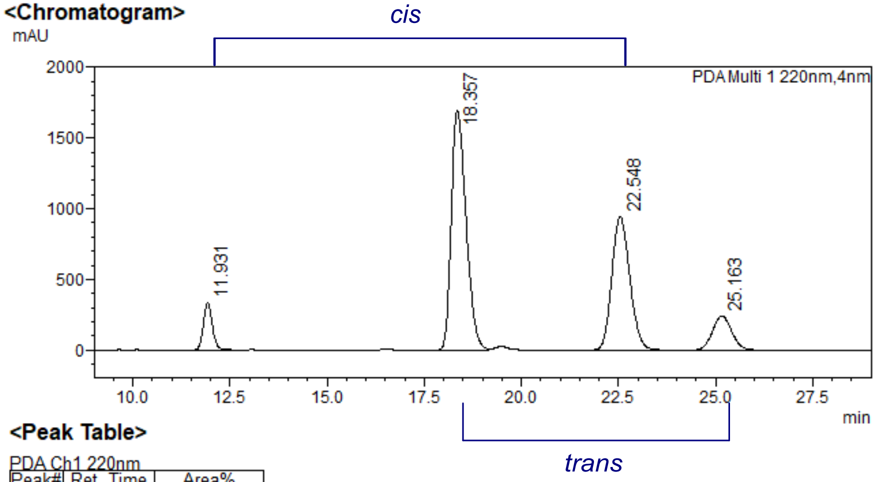

<Peak Table>

PDA Ch1 220nm

Peak#	Ret. Time	Area%
1	11.931	6.112
2	18.357	50.308
3	22.548	34.239
4	25.163	9.341
Total		100.000

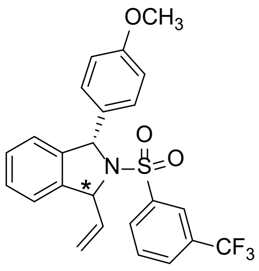

2bh,1:1.4 dr

HPLC of **2ch**

Racemic

BA catalyst: DPP

Solvent: toluene

<Sample Information>

Data Filename : 240710_racemic_2tk(4-FPh)_ADH98_OJ96_IC95_ODH99_7102024_011.lcd
Method Filename : V2_H99_I01_R1_45min.lcm
Batch Filename : 240710_racemic_2tk(4-FPh)_ADH98_OJ96_IC95_ODH99.lcb

<Chromatogram>

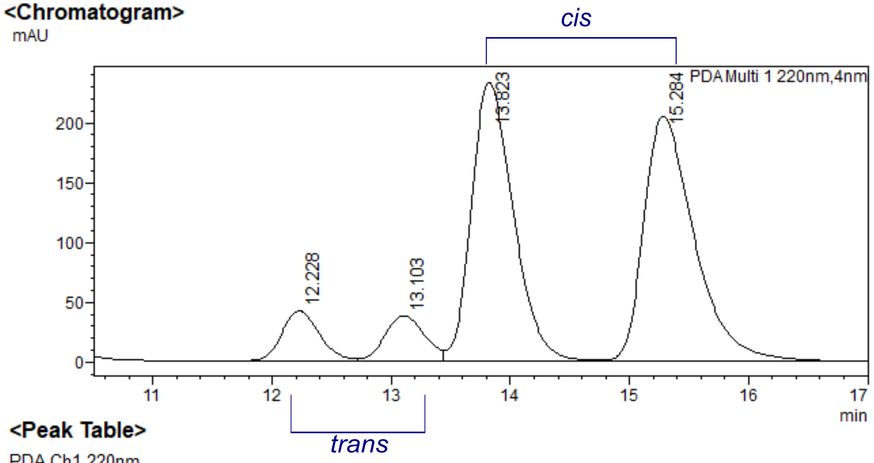

<Peak Table>

PDA Ch1 220nm

Peak#	Ret. Time	Area%
1	12.228	6.864
2	13.103	6.503
3	13.823	41.984
4	15.284	44.649
Total		100.000

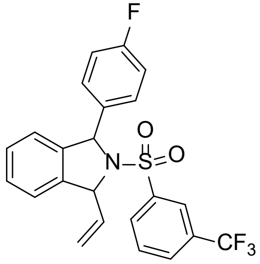

(±)-**2ch**

Chiral

BA catalyst: (*R*)-**CPA1**

Solvent: DCE

<Sample Information>

Data Filename : 240719_chiral_1tk_1h30m(P)2rd_7192024_002.lcd
Method Filename : V2_H99_I01_R1_30min.lcm
Batch Filename : 240719_chiral_1tk_1h30m(P)2rd.lcb

<Chromatogram>

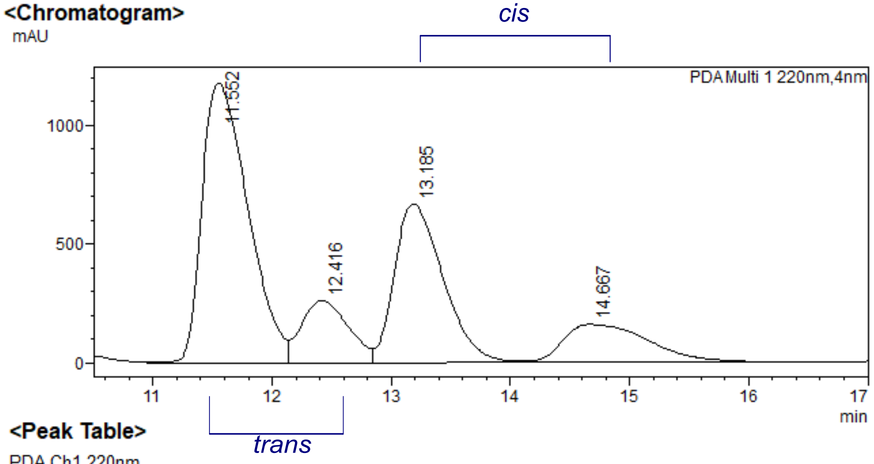

<Peak Table>

PDA Ch1 220nm

Peak#	Ret. Time	Area%
1	11.552	47.942
2	12.416	10.831
3	13.185	28.813
4	14.667	12.415
Total		100.000

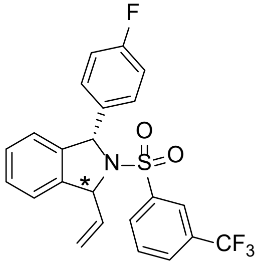

2ch,1.2:1 dr

HPLC of **2dh**

Racemic

BA catalyst: DPP

Solvent: toluene

<Sample Information>

Data Filename	: 240827_racemic_2zdk(4-CF3)_ADH98_2nd_8272024_002.lcd
Method Filename	: V5_H98_I02_R1_35min.lcm
Batch Filename	: 240827_racemic_2zdk(4-CF3)_ADH98_2nd.lcb

<Chromatogram>

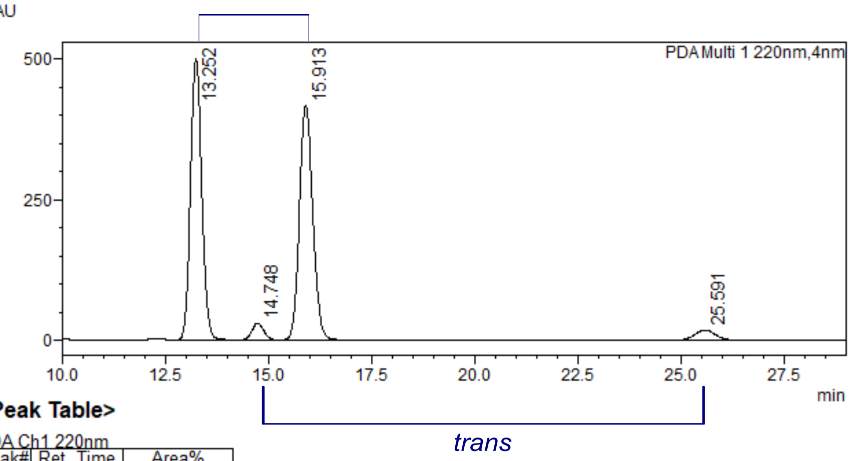

<Peak Table>

PDA Ch1 220nm

Peak#	Ret. Time	Area%
1	13.252	47.168
2	14.748	3.020
3	15.913	46.784
4	25.591	3.028
Total		100.000

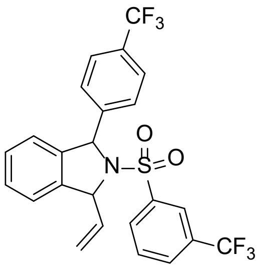

(±)-**2dh**

Chiral

BA catalyst: (*R*)-**CPA1**

Solvent: DCE

<Sample Information>

Data Filename	: 240828_1zdk(4-CF3)_8m_30mg(P)2nd_8292024_002.lcd
Method Filename	: V5_H98_I02_R1_35min.lcm
Batch Filename	: 240828_1zdk(4-CF3)_8m_30mg(P)2nd.lcb

<Chromatogram>

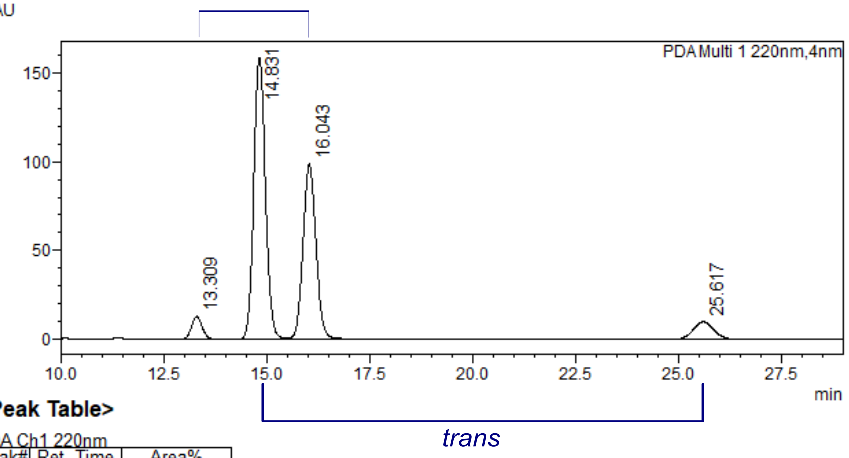

<Peak Table>

PDA Ch1 220nm

Peak#	Ret. Time	Area%
1	13.309	3.892
2	14.831	53.532
3	16.043	36.881
4	25.617	5.695
Total		100.000

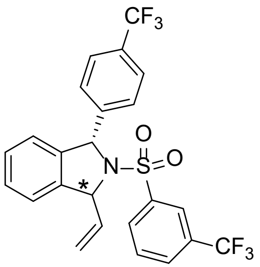

2dh,1:1.5 dr

HPLC of **2eh**

Racemic

BA catalyst: DPP

Solvent: toluene

<Sample Information>

Data Filename : 240802_racemic_2yk_ADH95_822024_002.lcd
Method Filename : V5_H95_I05_R1_30min.lcm
Batch Filename : 240802_racemic_2yk_ADH95.lcb

<Chromatogram>

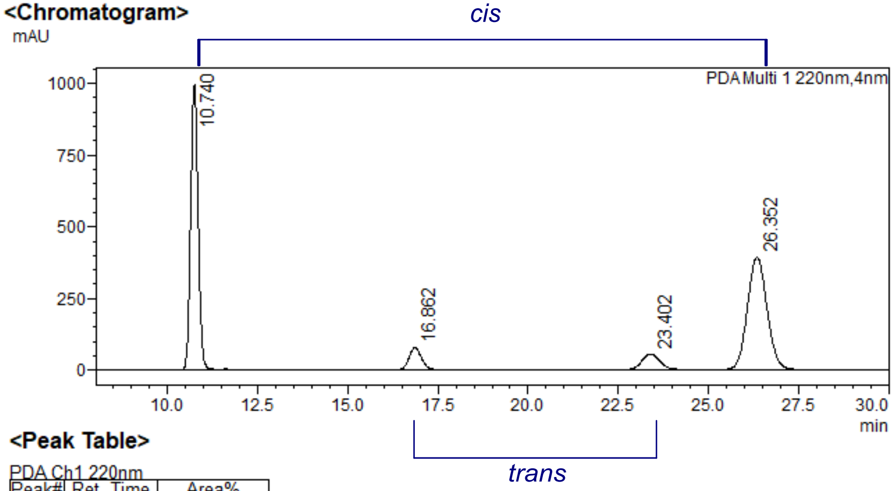

<Peak Table>

PDA Ch1 220nm

Peak#	Ret. Time	Area%
1	10.740	43.834
2	16.862	5.561
3	23.402	5.650
4	26.352	44.955
Total		100.000

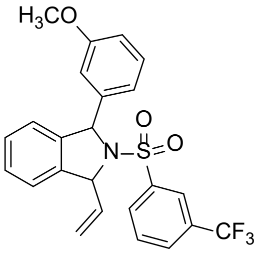

(±)-**2eh**

Chiral

BA catalyst: (*R*)-**CPA1**

Solvent: DCE

<Sample Information>

Data Filename : 240804_chiral_1yk_1h50m(P)_842024_002.lcd
Method Filename : V5_H95_I05_R1_30min.lcm
Batch Filename : 240804_chiral_1yk_1h50m(P).lcb

<Chromatogram>

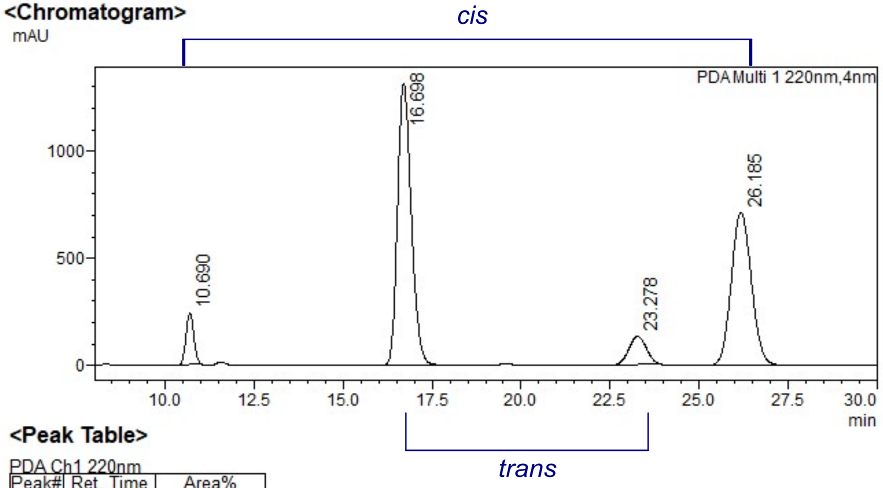

<Peak Table>

PDA Ch1 220nm

Peak#	Ret. Time	Area%
1	10.690	5.096
2	16.698	49.380
3	23.278	6.288
4	26.185	39.235
Total		100.000

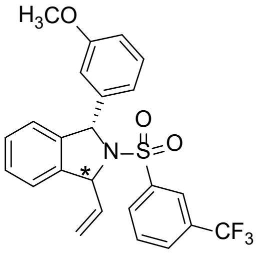

2eh,1:1.2 dr

HPLC of **2fh**

Racemic

BA catalyst: DPP

Solvent: toluene

<Sample Information>

Data Filename : 240803_racemic_2zk(3-Me)OJ96_IC95_ODH99_832024_008.lcd
Method Filename : V2_H99_I01_R1_45min.lcm
Batch Filename : 240803_racemic_2zk(3-Me)OJ96_IC95_ODH99.lcb

<Chromatogram>

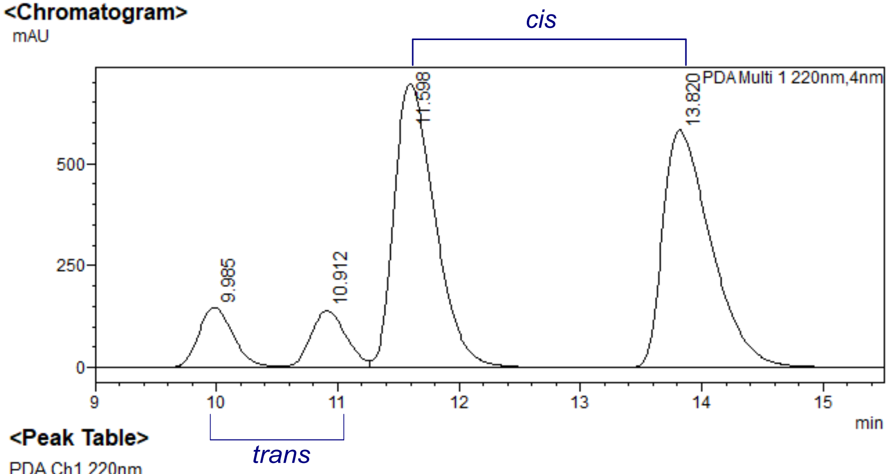

<Peak Table>

PDA Ch1 220nm

Peak#	Ret. Time	Area%
1	9.985	7.679
2	10.912	7.364
3	11.598	42.586
4	13.820	42.372
Total		100.000

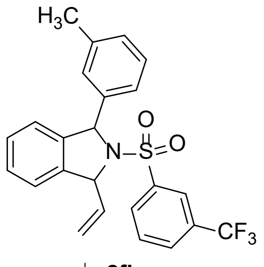

(±)-**2fh**

Chiral

BA catalyst: (*R*)-**CPA1**

Solvent: DCE

<Sample Information>

Data Filename : 240808_chiral_1Zk_3h(P)_882024_002.lcd
Method Filename : V2_H99_I01_R1_20min.lcm
Batch Filename : 240808_chiral_1Zk_3h(P).lcb

<Chromatogram>

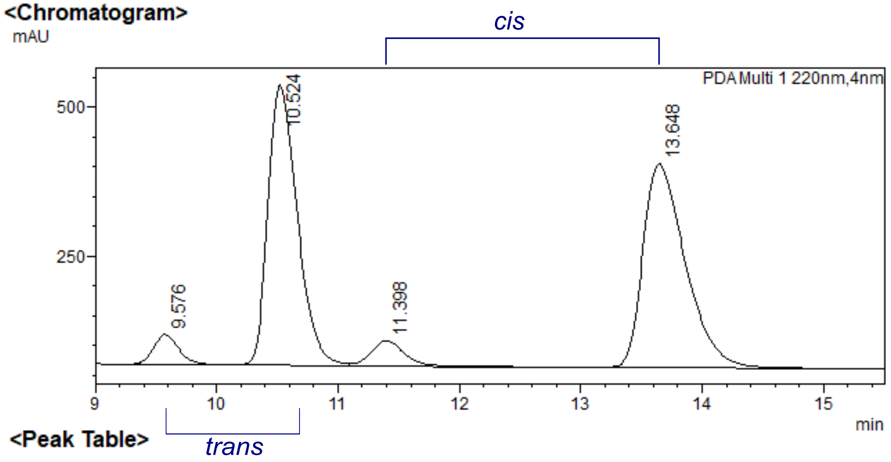

<Peak Table>

PDA Ch1 220nm

Peak#	Ret. Time	Area%
1	9.576	3.992
2	10.524	44.668
3	11.398	4.430
4	13.648	46.909
Total		100.000

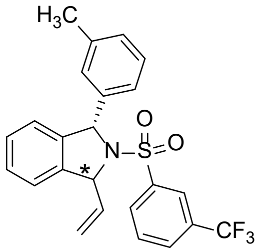

2fh,1:1 dr

HPLC of **2gh**

Racemic

BA catalyst: DPP

Solvent: DCE

<Sample Information>

Data Filename : 240718_racemic_2vk(35CF3)_ADH99_7182024_002.lcd
Method Filename : V5_H99_I01_R1_30min.lcm
Batch Filename : 240718_racemic_2vk(35CF3)_ADH99.lcb

<Chromatogram>

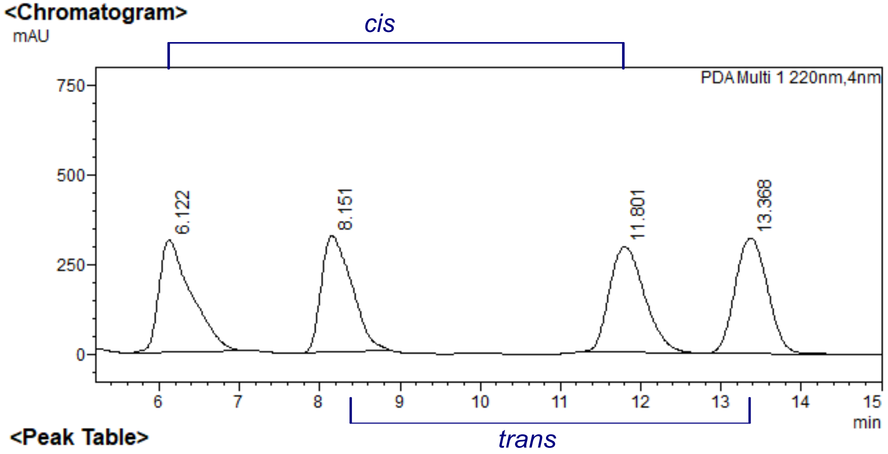

<Peak Table>

PDA Ch1 220nm

Peak#	Ret. Time	Area%
1	6.122	24.912
2	8.151	24.382
3	11.801	25.282
4	13.368	25.424
Total		100.000

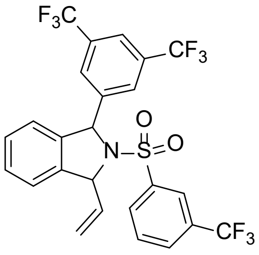

(±)-**2gh**

Chiral

BA catalyst: (*R*)-**CPA1**

Solvent: DCE

<Sample Information>

Data Filename : 240720_chiral_1vk_10min(P)2rd_7212024_002.lcd
Method Filename : V5_H99_I01_R1_30min.lcm
Batch Filename : 240720_chiral_1vk_10min(P)2rd.lcb

<Chromatogram>

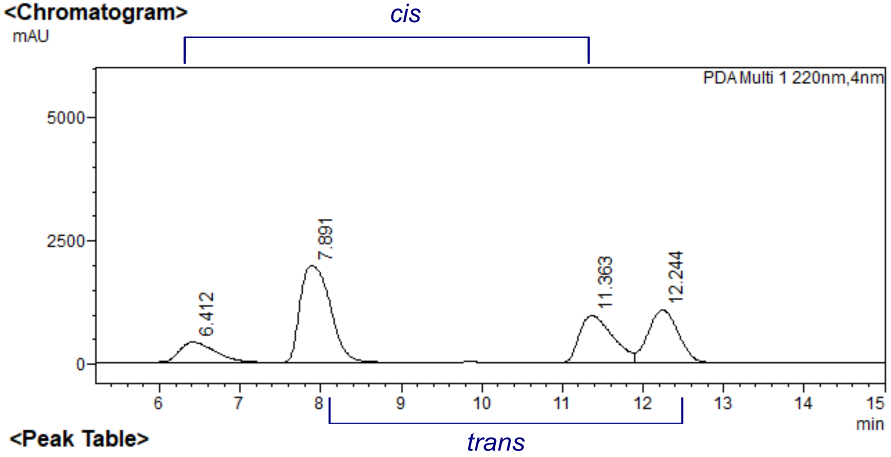

<Peak Table>

PDA Ch1 220nm

Peak#	Ret. Time	Area%
1	6.412	10.771
2	7.891	42.627
3	11.363	23.247
4	12.244	23.355
Total		100.000

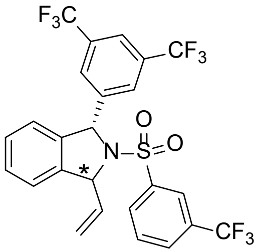

2gh,1:1.6 dr

HPLC of **2hh**

Racemic

BA catalyst: DPP

Solvent: DCE

<Sample Information>

Data Filename : 240809_racemic_2zak(13benzodioxle)_ADH95_3rd_8132024_002.lcd
Method Filename : V5_H95_I05_R1_55min.lcm
Batch Filename : 240809_racemic_2zak(13benzodioxle)_ADH95_3rd.lcb

<Chromatogram>

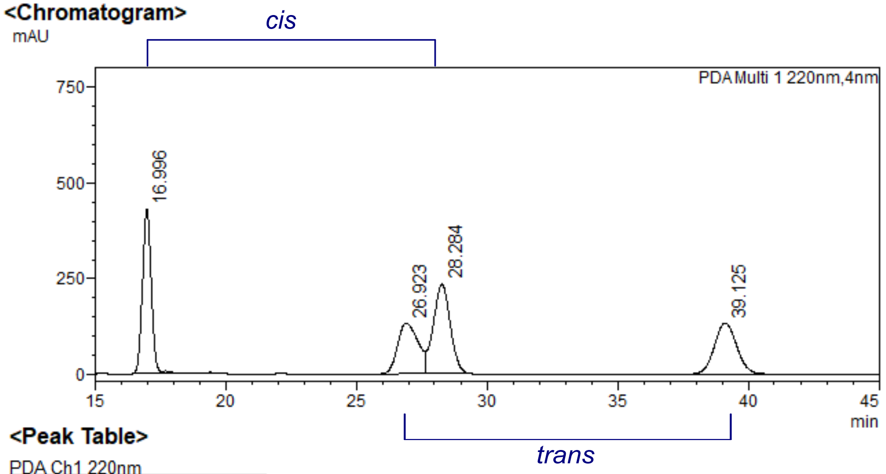

<Peak Table>

PDA Ch1 220nm

Peak#	Ret. Time	Area%
1	16.996	28.175
2	26.923	20.242
3	28.284	29.499
4	39.125	22.084
Total		100.000

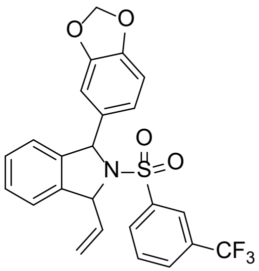

(±)-**2hh**

Chiral

BA catalyst: (*R*)-**CPA1**

Solvent: DCE

<Sample Information>

Data Filename : 240813_chiral_1zak(13benzodioxle)_2h35m(P)_8132024_002.lcd
Method Filename : V5_H95_I05_R1_55min.lcm
Batch Filename : 240813_chiral_1zak(13benzodioxle)_2h35m(P).lcb

<Chromatogram>

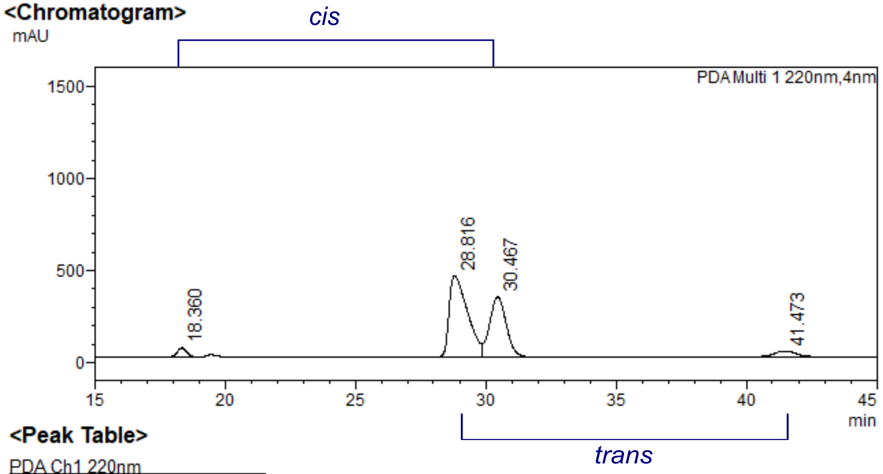

<Peak Table>

PDA Ch1 220nm

Peak#	Ret. Time	Area%
1	18.360	2.967
2	28.816	55.478
3	30.467	36.968
4	41.473	4.587
Total		100.000

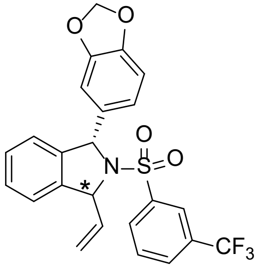

2hh,1:1.5 dr

HPLC of **2ih**

Racemic

BA catalyst: DPP

Solvent: DCE

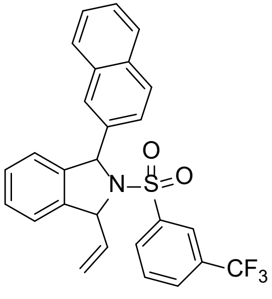

(±)-**2ih**

<Sample Information>

Data Filename : 240730_racemic_2xk(2-naph)_ADH98_80min_7302024_002.lcd
Method Filename : V5_H98_I02_R1_80min.lcm
Batch Filename : 240730_racemic_2xk(2-naph)_ADH98_80min.lcb

<Chromatogram>

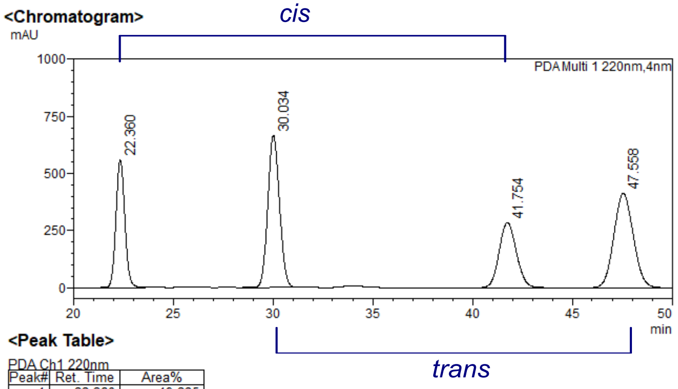

<Peak Table>

PDA Ch1 220nm

Peak#	Ret. Time	Area%
1	22.360	19.285
2	30.034	30.402
3	41.754	19.135
4	47.558	31.178
Total		100.000

Chiral

BA catalyst:

(*R*)-**CPA1**

Solvent: DCE

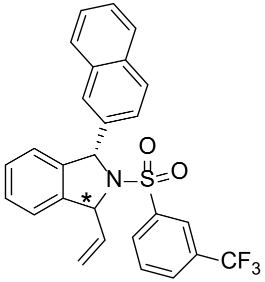

2ih,1:1.6 dr

<Sample Information>

Data Filename : 240802_chiral_1xk_1h40m(P)_812024_002.lcd
Method Filename : V5_H98_I02_R1_60min.lcm
Batch Filename : 240802_chiral_1xk_1h40m(P).lcb

<Chromatogram>

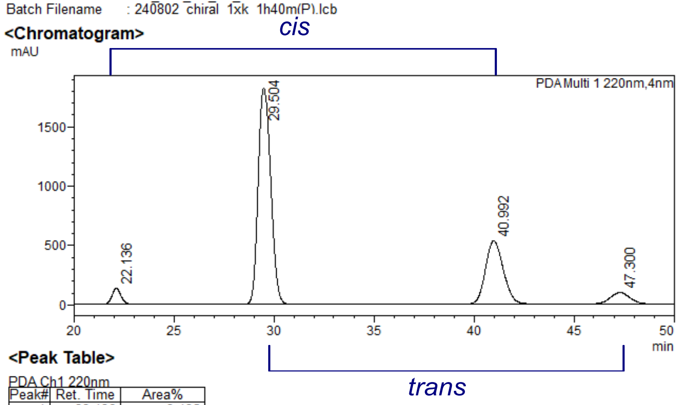

<Peak Table>

PDA Ch1 220nm

Peak#	Ret. Time	Area%
1	22.136	3.435
2	29.504	64.574
3	40.992	26.744
4	47.300	5.247
Total		100.000

Authentic

Compound

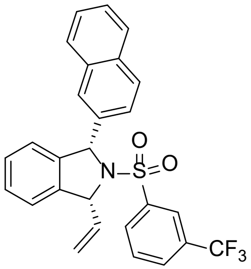

(1*S*,3*R*)-**2ih**, >20:1 dr
synthesized from **9b**

<Sample Information>

Data Filename : 250102_202512_002.lcd
Method Filename : V5_H98_I02_R1_80min.lcm
Batch Filename : 250102.lcb

<Chromatogram>

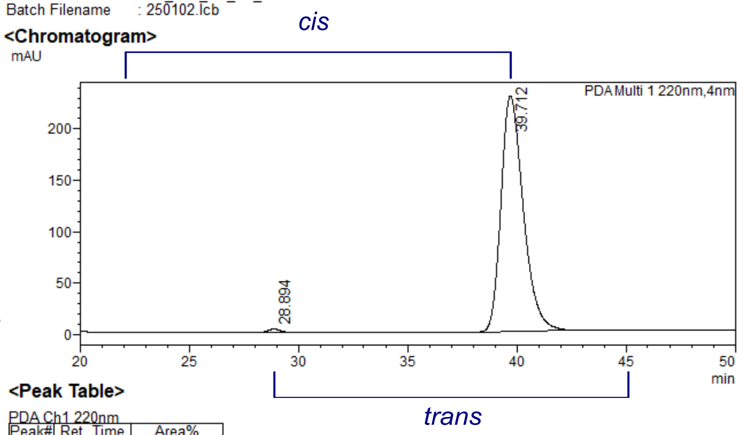

<Peak Table>

PDA Ch1 220nm

Peak#	Ret. Time	Area%
1	28.894	0.735
2	39.712	99.265
Total		100.000

HPLC of **2jh**

Racemic

BA catalyst: DPP

Solvent: DCE

<Sample Information>

Data Filename : 240826_racemic_1zck(P)_IC93 R0.8_8262024_002.lcd
Method Filename : V4_H93_I07_R0.8_50min.lcm
Batch Filename : 240826_racemic_1zck(P)_IC93 R0.8.lcb

<Chromatogram>

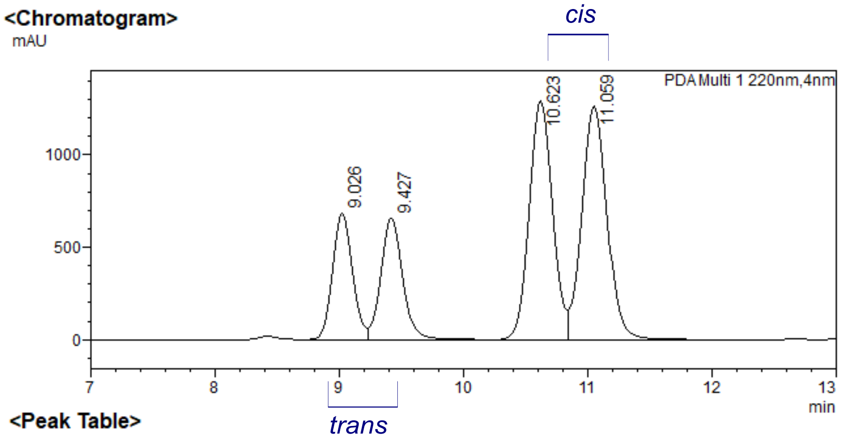

<Peak Table>

PDA Ch1 220nm

Peak#	Ret. Time	Area%
1	9.026	15.263
2	9.427	15.931
3	10.623	33.753
4	11.059	35.053
Total		100.000

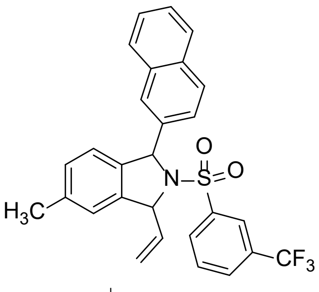

(±)-**2jh**

Chiral

BA catalyst: (*R*)-**CPA1**

Solvent: DCE

<Sample Information>

Data Filename : 240828_chiral_2zck 30mg 20min_IC93 R0.8_8282024_002.lcd
Method Filename : V4_H93_I07_R0.8_30min.lcm
Batch Filename : 240828_chiral_2zck 30mg 20min_IC93 R0.8.lcb

<Chromatogram>

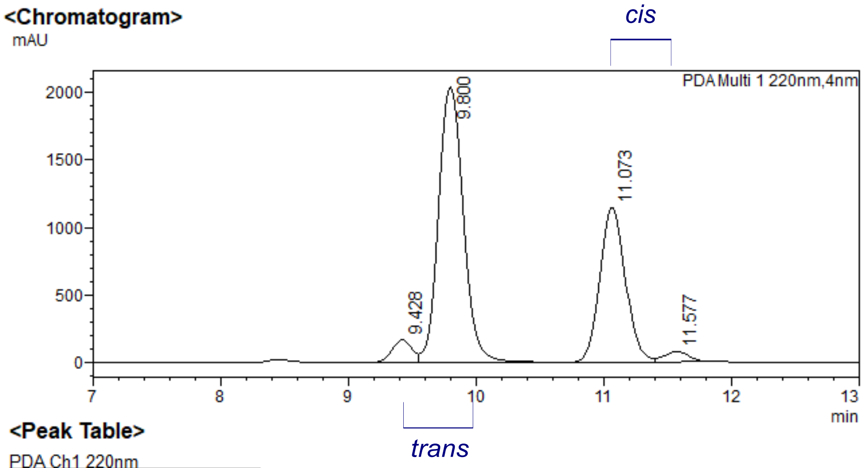

<Peak Table>

PDA Ch1 220nm

Peak#	Ret. Time	Area%
1	9.428	4.038
2	9.800	59.916
3	11.073	33.878
4	11.577	2.168
Total		100.000

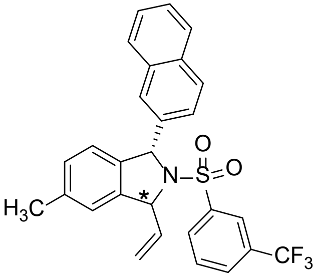

2jh,1:1.8 dr

HPLC of **2kh**

Racemic

BA catalyst: DPP

Solvent: DCE

<Sample Information>

Data Filename : 240911_racemic_2zek(5f2naph)_ADH980260min_9112024_002.lcd
Method Filename : V5_H98_I02_R1_60min.lcm
Batch Filename : 240911_racemic_2zek(5f2naph)_ADH980260min.lcb

<Chromatogram>

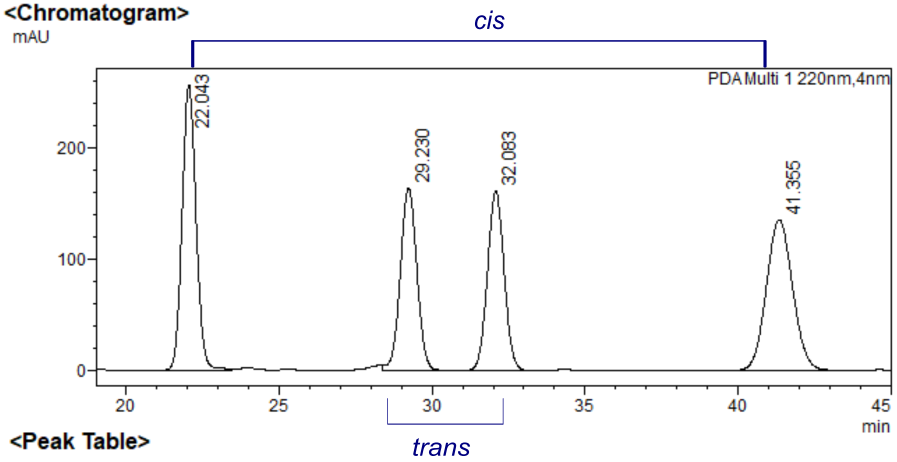

<Peak Table>

PDA Ch1 220nm

Peak#	Ret. Time	Area%
1	22.043	28.571
2	29.230	21.600
3	32.083	21.485
4	41.355	28.344
Total		100.000

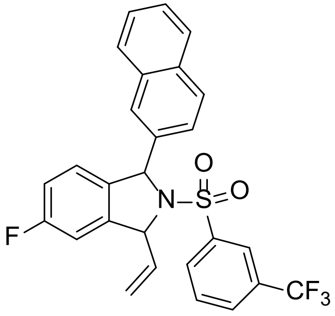

(±)-**2kh**

Chiral

BA catalyst: (*R*)-**CPA1**

Solvent: DCE

<Sample Information>

Data Filename : 250114-2_2025114_002.lcd
Method Filename : V5_H98_I02_R1_50min.lcm
Batch Filename : 250114-2.lcb

<Chromatogram>

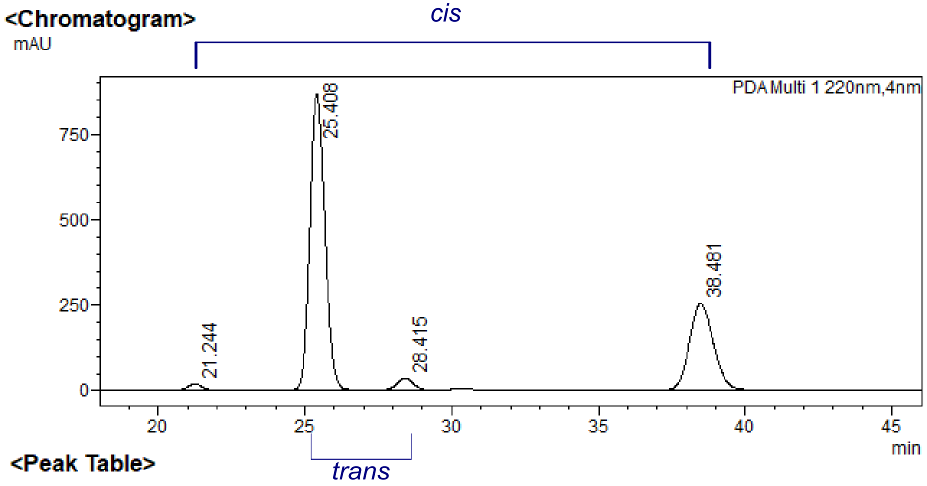

<Peak Table>

PDA Ch1 220nm

Peak#	Ret. Time	Area%
1	21.244	1.230
2	25.408	65.554
3	28.415	2.840
4	38.481	30.376
Total		100.000

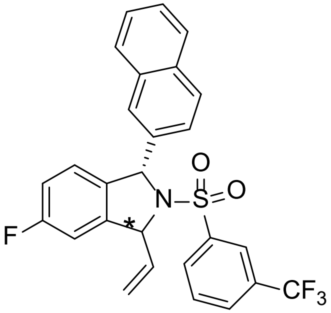

2kh,1:1.7 dr

HPLC of **2lh**

Racemic
BA catalyst: DPP
Solvent: DCE

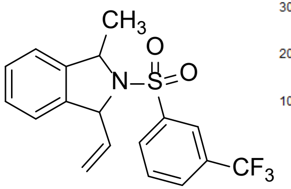

(±)-**2lh**

<Sample Information>

Data Filename : 240622_racemic_2bk_odh99.4_6232024_002.lcd
Method Filename : V2_H99.4_I0.6_R1_30min.lcm
Batch Filename : 240622_racemic_2bk_odh99.4.lcb

<Chromatogram>

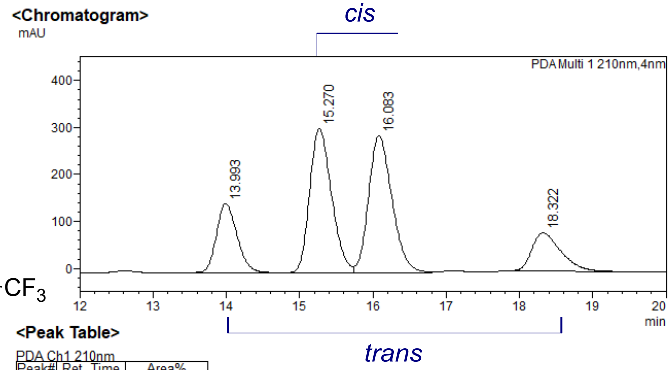

<Peak Table>

PDA Ch1 210nm

Peak#	Ret. Time	Area%
1	13.993	15.951
2	15.270	35.275
3	16.083	35.946
4	18.322	12.828
Total		100.000

Chiral
BA catalyst: (*R*)-**CPA1**
Solvent: DCE

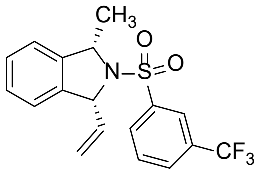

2lh, 5.5:1 dr

<Sample Information>

Data Filename : 240624_chiral_1bk_DCECPA6_2h5m(P)_6242024_002.lcd
Method Filename : V2_H99.4_I0.6_R1_30min.lcm
Batch Filename : 240624_chiral_1bk_DCECPA6_2h5m(P).lcb

<Chromatogram>

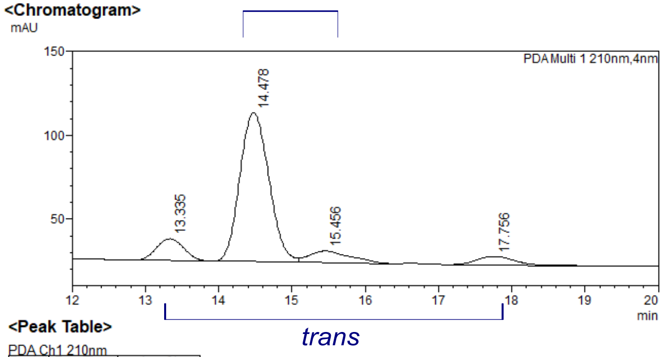

<Peak Table>

PDA Ch1 210nm

Peak#	Ret. Time	Area%
1	13.335	10.460
2	14.478	75.663
3	15.456	8.383
4	17.756	5.494
Total		100.000

Authentic Compound

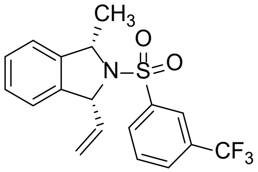

(1*S*,3*R*)-**2lh**, >20:1 dr, synthesized from **9c**

<Sample Information>

Data Filename : 250112-1_2025112_002.lcd
Method Filename : V2_H99.4_I0.6_R1_30min.lcm
Batch Filename : 250112-1.lcb

<Chromatogram>

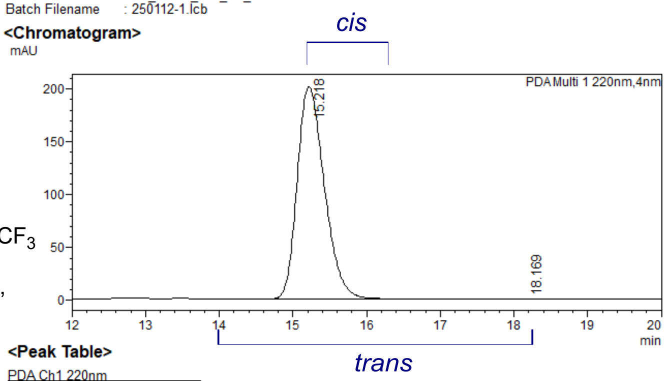

<Peak Table>

PDA Ch1 220nm

Peak#	Ret. Time	Area%
1	15.218	99.911
2	18.169	0.089
Total		100.000

HPLC of **2ih** [Stereodivergent Synthesis of (1*R*,3*R*)-isomer]

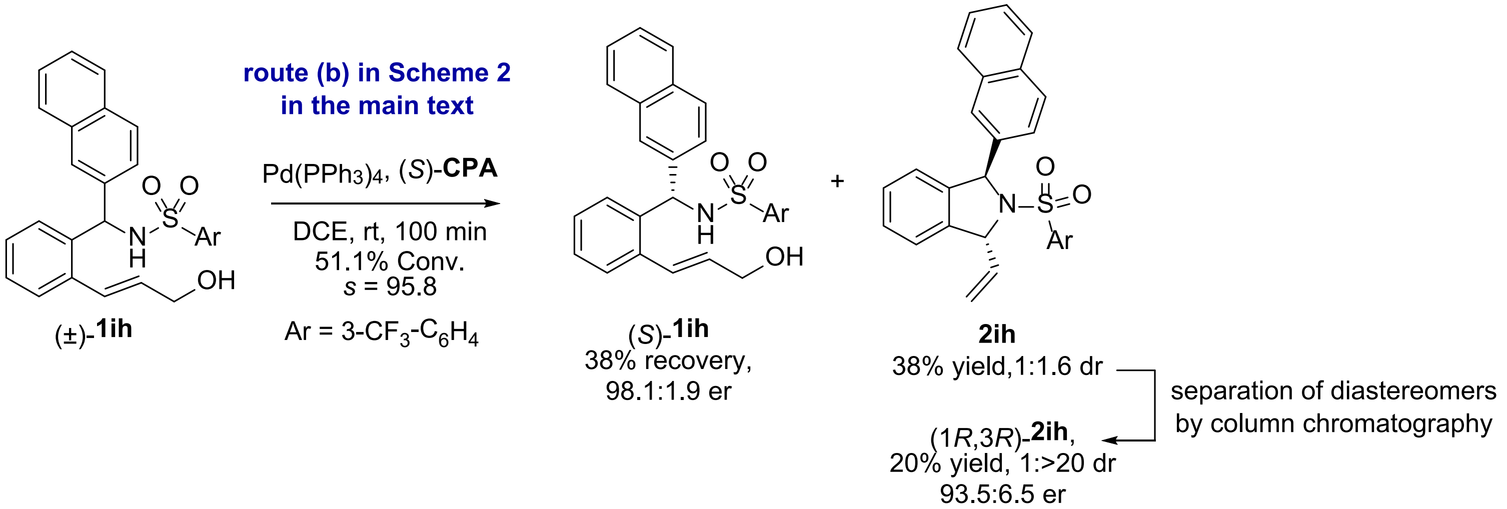

BA catalyst: (*S*)-**CPA1**

Solvent: DCE

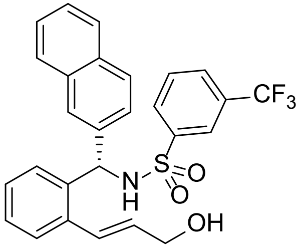

(*S*)-**1ih**

<Sample Information>

Data Filename : 240911_chiral_1XK_1h45MINby scpa_9122024_002.lcd
Method Filename : V5_H88_I12_R1_30min.lcm
Batch Filename : 240911_chiral_1XK_1h45MINby scpa.lcb

<Chromatogram>

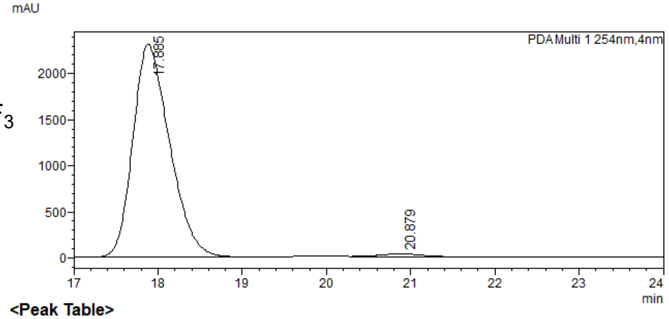

<Peak Table>

PDA Ch1 254nm

Peak#	Ret. Time	Area%
1	17.885	98.085
2	20.879	1.915
Total		100.000

BA catalyst: (*S*)-**CPA1**

Solvent: DCE

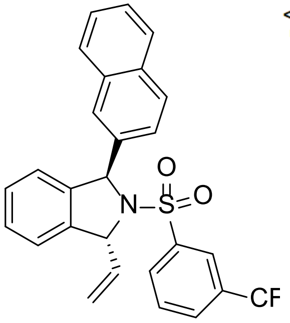

(1*R*,3*R*)-**2ih**
1:>20 dr (*cis*/*trans*)

<Sample Information>

Data Filename : 240911_cis-1R3S_2XK_1h45MINby scpa_9112024_002.lcd
Method Filename : V5_H98_I02_R1_60min.lcm
Batch Filename : 240911_cis-1R3S_2XK_1h45MINby scpa.lcb

<Chromatogram>

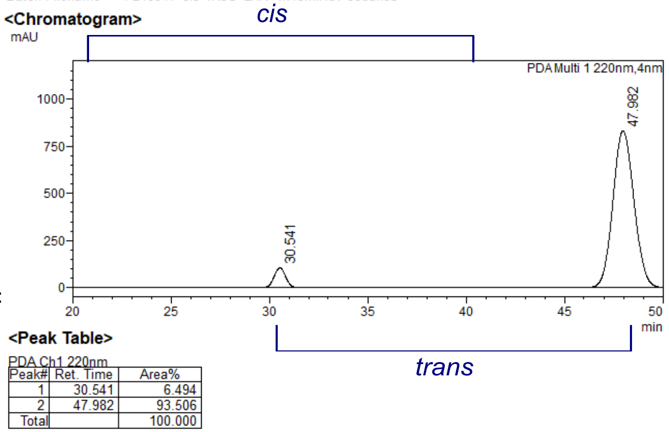

<Peak Table>

PDA Ch1 220nm

Peak#	Ret. Time	Area%
1	30.541	6.494
2	47.982	93.506
Total		100.000

HPLC of **2ih** [Stereodivergent Synthesis of (1*R*,3*S*)-isomer]

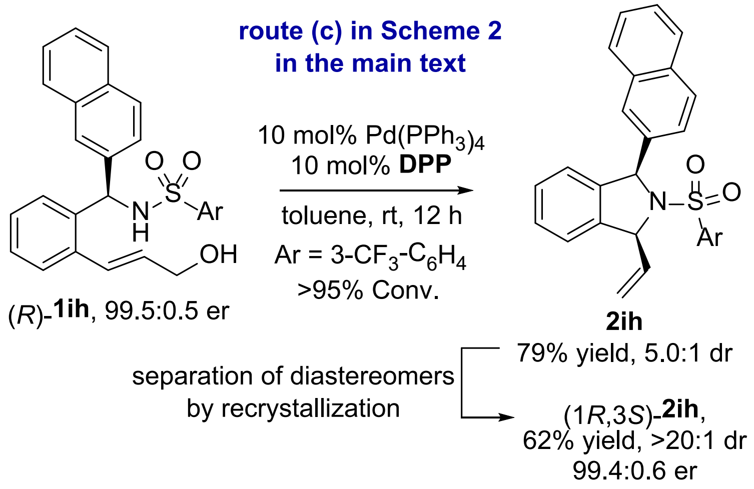

BA catalyst: DPP

Solvent: toluene

<Sample Information>

Data Filename : 240826_trans-(1R3R)2xk(crystal)_8262024_002.lcd
Method Filename : V5_H98_I02_R1_60min.lcm
Batch Filename : 240826_trans-(1R3R)2xk(crystal).lcb

<Chromatogram>

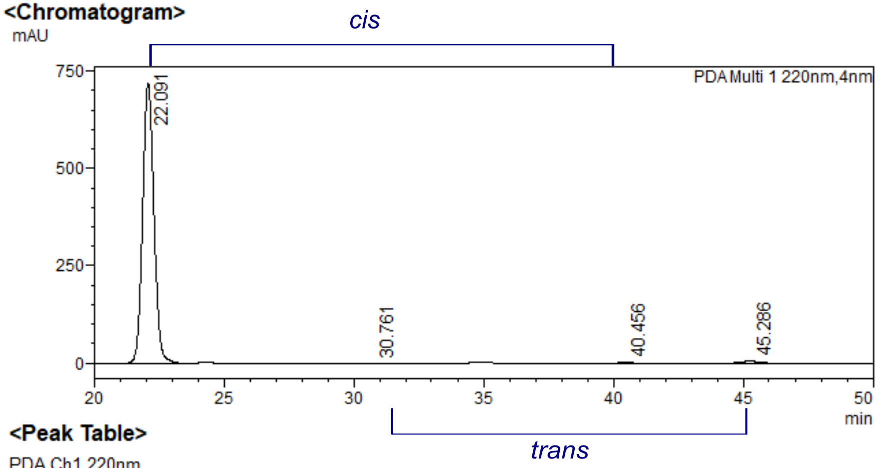

<Peak Table>

PDA Ch1 220nm

Peak#	Ret. Time	Area%
1	22.091	97.718
2	30.761	0.047
3	40.456	0.599
4	45.286	1.636
Total		100.000

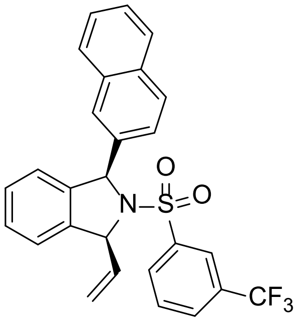

(1*R*,3*S*)-**2ih'**
>20:1 dr (*cis*/*trans*)

HPLC of **2ih** [Stereodivergent Synthesis of (1*S*,3*R*)-isomer]

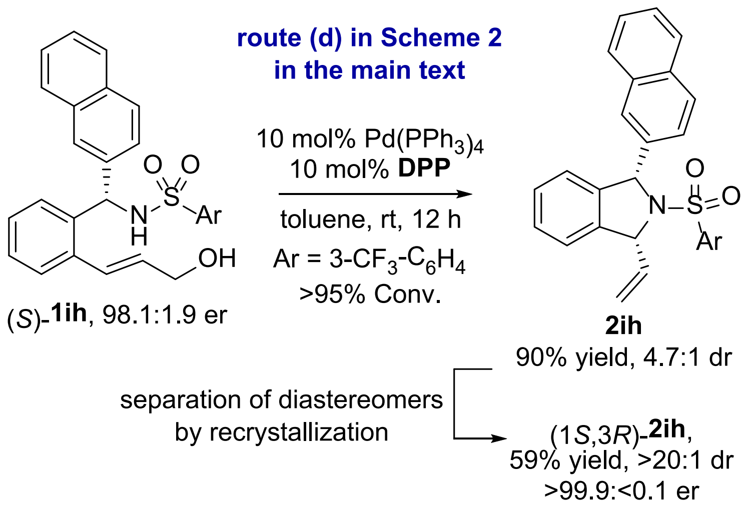

BA catalyst: DPP

Solvent: toluene

<Sample Information>

Data Filename : 240923_trans-1S3S_2XK_crystal2rd_9232024_002.lcd
Method Filename : V5_H98_I02_R1_60min.lcm
Batch Filename : 240923_trans-1S3S_2XK_crystal2rd.lcb

<Chromatogram>

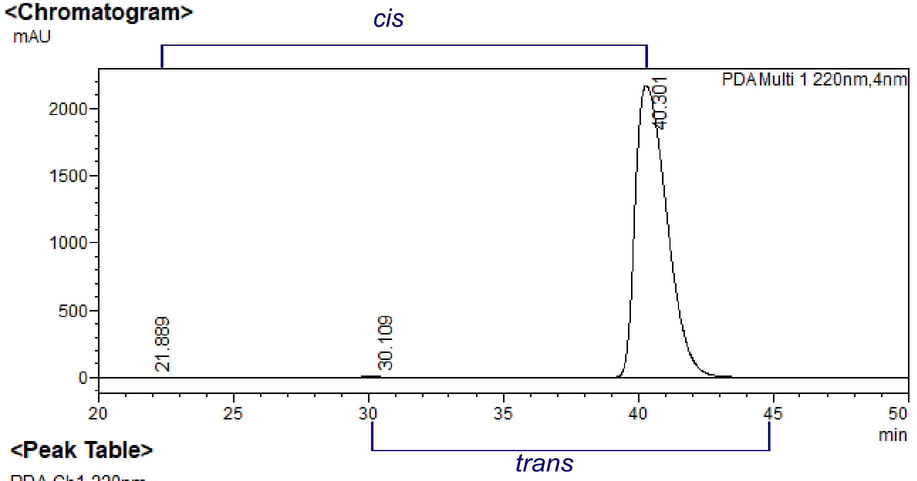

<Peak Table>

PDA Ch1 220nm

Peak#	Ret. Time	Area%
1	21.889	0.008
2	30.109	0.285
3	40.301	99.707
Total		100.000

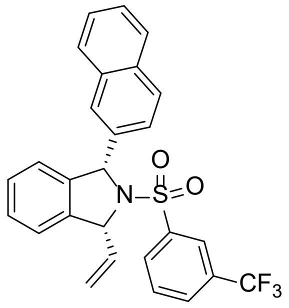

(1*S*,3*R*)-**2ih**'
>20:1 dr (*cis*/*trans*)
